# International Variations in Surgical Morbidity and Mortality Post Gynaecological Oncology Surgery: A Global Gynaecological Oncology Surgical Outcomes Collaborative Led Study (GO SOAR1)

**DOI:** 10.3390/cancers15205001

**Published:** 2023-10-16

**Authors:** Faiza Gaba, Karen Ash, Oleg Blyuss, Nicolò Bizzarri, Paul Kamfwa, Allison Saiz, David Cibula, GO SOAR Collaborators

**Affiliations:** 1 Department of Gynaecological Oncology, Royal London Hospital, Barts Health NHS Trust, London E1 1FR, UK; 2 Institute of Applied Health Sciences, University of Aberdeen, Aberdeen AB24 3FX, UK; 3 Aberdeen Royal Infirmary, NHS Grampian, Aberdeen AB25 2ZN, UK; 4 Wolfson Institute of Population Health, Queen Mary University of London, London EC1M 6BQ, UK; 5 UOC Ginecologia Oncologica, Dipartimento per la Salute della Donna e del Bambino e della Salute Pubblica, Fondazione Policlinico Universitario A. Gemelli, IRCCS, 00168 Rome, Italy; 6 Cancer Diseases Hospital, Lusaka 10101, Zambia; 7 Northwestern University in Chicago, Chicago, IL 60611, USA; 8 Department of Obstetrics and Gynaecology, First Faculty of Medicine, Charles University and General University Hospital, 121 08 Prague, Czech Republic

**Keywords:** surgery, gynaecological oncology, morbidity, mortality, collaborative research

## Abstract

**Simple Summary:**

Little is known about factors contributing to early post-operative morbidity and mortality in low and middle income countries with a paucity of data limiting global efforts to improve gynaecological cancer care. In this multicentre, international prospective cohort study of women undergoing gynaecological oncology surgery, we show that low and middle versus high income countries were associated with similar post-operative major morbidity. Capacity to rescue patients from surgical complications is a tangible opportunity for meaningful intervention.

**Abstract:**

Gynaecological malignancies affect women in low and middle income countries (LMICs) at disproportionately higher rates compared with high income countries (HICs) with little known about variations in access, quality, and outcomes in global cancer care. Our study aims to evaluate international variation in post-operative morbidity and mortality following gynaecological oncology surgery between HIC and LMIC settings. Study design consisted of a multicentre, international prospective cohort study of women undergoing surgery for gynaecological malignancies (NCT04579861). Multilevel logistic regression determined relationships within three-level nested-models of patients within hospitals/countries. We enrolled 1820 patients from 73 hospitals in 27 countries. Minor morbidity (Clavien–Dindo I–II) was 26.5% (178/672) and 26.5% (267/1009), whilst major morbidity (Clavien–Dindo III–V) was 8.2% (55/672) and 7% (71/1009) for LMICs/HICs, respectively. Higher minor morbidity was associated with pre-operative mechanical bowel preparation (OR = 1.474, 95%CI = 1.054–2.061, *p* = 0.023), longer surgeries (OR = 1.253, 95%CI = 1.066–1.472, *p* = 0.006), greater blood loss (OR = 1.274, 95%CI = 1.081–1.502, *p* = 0.004). Higher major morbidity was associated with longer surgeries (OR = 1.37, 95%CI = 1.128–1.664, *p* = 0.002), greater blood loss (OR = 1.398, 95%CI = 1.175–1.664, *p* ≤ 0.001), and seniority of lead surgeon, with junior surgeons three times more likely to have a major complication (OR = 2.982, 95%CI = 1.509–5.894, *p* = 0.002). Of all surgeries, 50% versus 25% were performed by junior surgeons in LMICs/HICs, respectively. We conclude that LMICs and HICs were associated with similar post-operative major morbidity. Capacity to rescue patients from surgical complications is a tangible opportunity for meaningful intervention.

## 1. Introduction

Gynaecological malignancies collectively, after breast cancer, represent the second largest disease burden amongst all female cancers, and by the year 2040 incidence is set to rise by 69% [1]. They continue to affect women in low and middle income countries (LMICs) at disproportionately higher rates in comparison to high income countries (HICs). However, estimates of incidence and distribution by stage are absent for many LMICs, with a paucity of data on variations in access, quality, and outcomes in global cancer care [2].

Irrespective of country development status, surgery is key for cancer cure and palliation. Solid tumours are often untreated in LMICs and this brings major harmful macroeconomic sequelae, with cumulative gross domestic product losses estimated to be as high as 1.2% for 2016–2030 [3]. Cytoreductive surgeries are often extremely invasive, resulting in post-operative morbidity and mortality. Rescuing patients with morbidity from dying is an important focus of surgical quality improvement [4]. Little is known about factors contributing to early post-operative morbidity and mortality in LMICs, with the paucity of data limiting global efforts to improve cancer care. Strategic planning requires detailed and precise information, enabling suitable resource allocation and prioritisation of quality improvement. Real world clinical data and details of hospital resources are required to improve public health enterprises, treatment approaches, and quality improvement interventions [2]. To address these issues, we performed a study to determine international variations in post-operative morbidity and mortality for gynaecological malignancies to act as surrogate markers for the best performance of gynaecological oncology surgical institutions.

## 2. Materials and Methods

This is a multicentre, international, prospective cohort study that has been prospectively registered (NCT04579861). The study was approved and registered with the Quality Improvement & Assurance Team (QIAT) at NHS Grampian (project ID 5009), UK. Both quantitative and qualitative data were collected.

### 2.1. Participants

Investigators included consecutive patients undergoing surgery for ovary/uterus/cervix/vulva/vagina/gestational trophoblastic cancers from a 30-day period (January 2021–November 2022). Inclusion criteria were women aged ≥18 years undergoing curative/palliative surgery for primary/recurrent gynaecological malignancies. Surgical modalities included were open, minimal access (laparoscopic/robotic), and vaginal. Elective and emergency cases were included. Patients were excluded if primary pathology was not a gynaecological malignancy or was benign/borderline disease, and if they had undergone a diagnostic procedure (for example examination under anaesthesia with biopsies, diagnostic hysteroscopy/laparoscopy/cystoscopy/sigmoidoscopy).

All consecutive cases in the selected 30-day period were included. Investigators were required to monitor patients for a minimum of 30 days post-operatively to identify complications. Post-operative follow up was dependent on local clinical pathways (in person, telephone, or review of medical records).

To confirm surgical outcome data collected were representative of the care received in each country, efforts were made to recruit large/medium/small centres performing gynaecological oncology surgery in a 1:1:1 ratio. Centre size was defined according to annual surgical caseload as follows: large >150, medium 75–149, small ≤74. Thresholds were determined as per the European Society of Gynaecological Oncology (ESGO) training centre accreditation criteria [5].

### 2.2. Data Collection and Validation

All 2816 data entry points (patient/surgical/disease characteristics) can be viewed in Supplementary File S1. Local teams uploaded data to a secure custom built Research Electronic Data Capture system database. To ensure data quality, all submitted data were checked centrally and when missing data/discrepancies were identified, local teams were contacted to complete and rectify discrepancies.

Data validation was accomplished in three phases. Firstly, centres self-reported the main processes used to ascertain and follow up patients. Secondly, independent validators (clinicians not part of the local study team) quantitatively reported case ascertainment and confirmed data accuracy. These local validators identified missing eligible patients within the local cohort and collected missing data. Thirdly, local teams were interviewed (face to face, virtually) by the central coordinating study team to qualitatively assess collaborator engagement and data collection practices.

### 2.3. Outcomes

Primary outcome measures were post-operative morbidity (defined as per Clavien–Dindo classification system) and mortality. Secondary outcome measures were designed to describe cancer care quality as a measure of cancer treatment pathways and hospital level care processes. They included unplanned re-intervention (operative/radiological/endoscopic), unplanned readmission, and comparison of current practice against selected tumour specific audit standards derived from ESGO guidelines. The audit standards compared were: (1) surgery performed by a trained gynaecological oncologist/other surgeon (formal training was defined as a competency based curriculum with assessments, informal training as clinical apprenticeship with no structured curriculum/no assessments) and; (2) treatment planned/reviewed at tumour board.

### 2.4. Sample Size

Non-inferiority/equivalence sample size calculation was used to support our null hypothesis that morbidity and mortality following gynaecological oncology surgery between HICs and LMICs are equivalent. Country income groups were determined as per the World Bank classification system. Whilst there is a paucity of data on morbidity and mortality following gynaecological oncology surgery in LMICs, data for HICs suggests 26% morbidity [6] and 2% mortality [7]. For a baseline 26% morbidity incidence in HICs, 450 patients per arm (HIC versus LMIC) allows a 10% point difference to be detected at 90% power, α = 0.05. For a baseline 2% mortality incidence in HICs, 135 patients per arm allows a 10% point difference to be detected at 90% power, α = 0.05. To account for missing data/loss to follow-up, sample size was inflated by 20%. Therefore, a sample size of 1100 (550/arm) at 90% power, α = 0.05, could determine a 10% difference in morbidity/mortality between HICs and LMICs.

### 2.5. Statistical Analysis

Variation across different international health settings was assessed by stratifying countries by World Bank country group classifications (HIC/LMIC). Differences between HICs and LMICs were tested with Fisher’s exact test for categorical variables and with the Wilcoxon rank sum test for continuous variables. Multilevel logistic regression models were constructed to account for case mix (patient, disease, operative characteristics), with population stratification by hospital and country of residence incorporated as random intercepts.

Three-level models (univariable/multivariable/multilevel) were constructed ensuring variables associated with outcome measures in previous studies were accounted for; demographic variables were included in model exploration; population stratification by hospital and country of residence was incorporated as random effects. Final (multilevel) model selection was performed using a criterion-based approach by minimising the Akaike information criterion. Effect estimates are presented as ORs and 95% CIs.

Mediation analysis was performed to further assess the association between length of surgery and different types of complications by two-way decomposition of total effects into direct and indirect effects. Mediator was defined as grade of surgeon (registrar/residents or consultant/attending). Models were adjusted by a set of covariates that included ECOG, age, ASA, elective or emergency surgery, tumour type/stage. All analyses were carried out using R (version 3.6.3).

### 2.6. Qualitative Data

Qualitative data were collected via in-depth semi-structured 1:1 interviews (face to face, virtual) using a topic guide (Supplementary File S2). Transcripts were analysed using an inductive theoretical framework and data managed using NVIVO v12. Two researchers (FG, KS) independently coded all transcripts, following a three-step process: open coding, axial coding, selective coding.

## 3. Results

**Figure 1:**
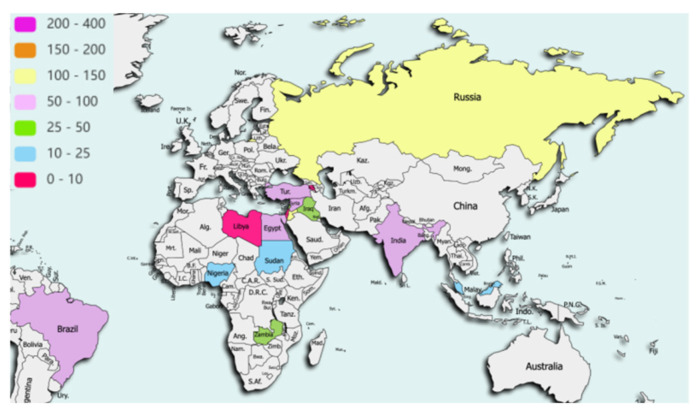
Distribution of cases from Low and Middle Income Countries. Data for 742 patients, from 41 hospitals, in 16 countries were included.

**Figure 2:**
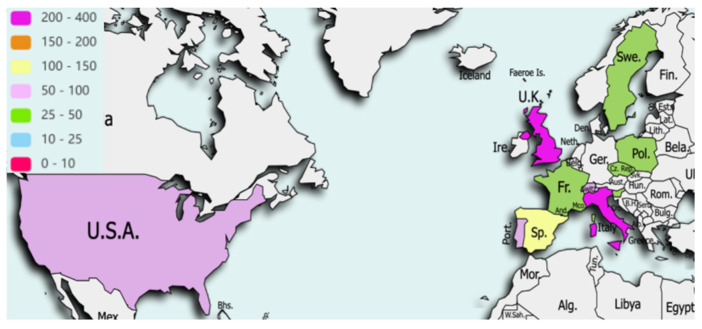
Distribution of cases from High Income Countries. Data for 1078 patients, from 32 hospitals, in 11 countries were included.

Data were collected for 1900 patients, of which 80 (4.2%) did not meet eligibility criteria, leaving 1820 records from 73 hospitals in 27 countries for the final analysis with the following geographical distribution (Figure 1 and Figure 2): 1195 (65.7%) Europe and Central Asia, 338 (18.6%) Middle East and North Africa, 90 (4.9%) Latin America and Caribbean, 62 (3.4%) North America, 69 (3.8%) Sub-Saharan Africa, 54 (3%) South Asia, 12 (0.7%) East Asia and Pacific. Mean follow up was 58.7 and 55.7 days from date of surgery in LMICs/HICs, respectively, and 6.1% (45/742) and 4.8% (52/1078) cases were lost to follow up in LMICs/HICs, respectively.

Table 1 summarizes baseline demographics. Patients from LMICs versus HICs had a poorer ECOG performance status (measure of a patient’s level of functioning in terms of ability to self-care/daily activity/physical ability [8]; performance status ≥3: 2.6% (19/731) versus 0.7% (7/1030), *p* ≤ 0.001), were more likely to undergo emergency surgery (2% (15/738) versus 0.6% (6/1076), *p* = 0.006), and were more likely to undergo open surgery (68.9% (506/734) versus 40.5% (435/1075), *p* ≤ 0.001) versus minimal access surgery (20.9% (153/734) versus 52.8% (567/1075), *p* ≤ 0.001). Distribution of cases per tumour group were: uterus 41.5% (755/1820), ovary 41% (746/1820), cervix 9.1% (165/1820), vulva 7.2% (131/1820), vagina 0.7% (14/1820) and gestational trophoblastic disease 0.5% (9/1820).

Unadjusted, overall morbidity (Clavien–Dindo I–IV) for all tumour groups was 34.7% (233/672) and 33.5% (338/1009), whilst unadjusted mortality was 2.1% (14/672) versus 1% (10/1009) for LMICs versus HICs, respectively. Unadjusted minor morbidity (Clavien–Dindo I–II) for all tumour groups was 26.5% (178/672) and 26.5% (267/1009), whilst unadjusted major morbidity (Clavien–Dindo III–V) was 8.2% (55/672) and 7% (71/1009) for LMICs/HICs, respectively. Table 2 summarises distribution of cancer type, unadjusted morbidity and mortality rates across country income group. Proportion of patients presenting with advanced stage (FIGO III–IV) disease was greater in LMICs versus HICs for uterus/cervix/vulva cancer and for ovary/vagina cancer greater in HICs versus LMICs (Supplementary Table S1). There was a positive correlation between cancer stage and ASA operative risk for patients with vaginal cancer (linear correlation coefficient = 0.62), but no correlation in patients with ovary/uterus/cervix/vulva/gestational trophoblastic disease cancer (Supplementary Table S2). No strong correlation between ECOG performance status and cancer stage was seen. Minor morbidity was higher for uterus/vulva/vaginal cancer and major morbidity higher for uterus/cervix cancer in LMICs versus HICs.

Outcomes were adjusted in three-level models accounting for patient and disease factors nested within hospital and country of treatment (Table 3 and Table 4). Higher minor morbidity (Table 3) was associated with previous laparoscopic surgery (OR = 1.435, 95%CI = 1.046–1.966, *p* = 0.025), COVID-19 positive status (OR = 5.025, 95%CI = 1.262–20.008, *p* = 0.022), pre-operative mechanical bowel preparation (OR = 1.474, 95%CI = 1.054–2.061, *p* = 0.023), longer surgeries (OR = 1.253, 95%CI = 1.066–1.472, *p* = 0.006), greater blood loss (OR = 1.274, 95%CI = 1.081–1.502, *p* = 0.004), and occurrence of intra-operative complications (OR = 2.203, 95%CI = 1.498–3.241, *p* < 0.001). Reduced minor morbidity was associated with minimal access versus open surgery (OR = 0.522, 95%CI = 0.371–0.735, *p* ≤ 0.001).

Higher major morbidity (Table 4) was associated with longer surgeries (OR = 1.37, 95%CI = 1.128–1.664, *p* = 0.002), greater blood loss (OR = 1.398, 95%CI = 1.175–1.664, *p* ≤ 0.001), and seniority of lead surgeon with junior surgeons (residents/registrars) versus senior surgeons (attendings/consultants) three times more likely to have a major complication (OR = 2.982, 95%CI = 1.509–5.894, *p* = 0.002). Overall, 50% versus 25% of all surgeries were performed by junior surgeons in LMICs and HICs respectively.

Unadjusted, intra-operative morbidity for all tumour groups was 14.7% (101/688) and 12.3% (126/1024) for LMICs versus HICs, respectively. Intra-operative morbidity was higher for all tumour groups (except gestational trophoblastic disease) in the LMIC group (Table 2). Higher adjusted intra-operative morbidity (Supplementary Table S3) was associated with previous laparoscopic surgery (OR = 1.6, 95%CI = 1.045–2.45, *p* = 0.031), longer surgeries (OR = 1.48, 95%CI = 1.201–1.824, *p* < 0.001), and greater blood loss (OR = 2.226, 95%CI = 1.784–2.778, *p* < 0.001). Reduced intra-operative morbidity was associated with elective versus emergency surgery (OR = 0.179, 95%CI = 0.042–0.758, *p* = 0.019) and minimal access modality (OR = 0.552, 95%CI = 0.331–0.921, *p* = 0.023).

For every additional hour of intra-operative time, the risk of an intra-operative complication went up by 18% and that of post-operative minor and major complications by 10% and 15%, respectively. Mediation analysis was performed to further assess the association between length of surgery and morbidity through two-way decomposition of total effects into direct and indirect. Mediator was defined as grade of surgeon (trainee registrar/resident or consultant/attending). Using bootstrap resampling (1000 draws), we found no significant average causal mediation effect in any of the analyses.

Unadjusted rates of complete macroscopic cytoreduction were similar for LMICs and HICs for each individual tumour group (Table 2). Higher adjusted rates of complete cytoreduction (Table 5) were associated with a lower ECOG performance status (OR = 0.572, 95%CI = 0.406–0.806, *p* = 0.001) and lower adjusted rates were associated with higher FIGO stage at presentation (OR = 23.316, 95%CI = 10.597–51.297, *p* < 0.001).

There was no association between country income setting and adjusted rates of post-operative or intra-operative morbidity/mortality. Rates of readmission (2.2% (37/692) versus 3.6% (62/1024), *p* = 0.598) and return to theatre (1.1% (19/691) versus 2.2% (38/1026), *p* = 0.337) did not statistically significantly differ between LMICs and HICs.

Supplementary Table S4 summarises tumour specific audit standard outcomes. Proportions of treatment plans discussed at tumour board meetings were similar between LMICs/HICs (82.2% versus 86.2% respectively for all tumour groups). For individual tumour groups, a greater proportion of treatment plans for ovary (92.7% versus 81.8%), cervix (86.1% versus 84.1%) and gestational trophoblastic malignancies (100% versus 66.7%) were discussed at tumour board meeting in HIC settings versus LMIC settings. Overall, in both LMICs/HICs, proportions of surgeries performed by a surgeon formally/informally trained in gynaecological oncology surgery were similar (93.3% versus 89.6% respectively for all tumour groups). However, the proportion of surgeries performed by surgical oncologists/general surgeons was greater in LMICs versus HICs (70% versus 2%). In our cohort, 51.9% (14/27) of HIC centres had surgeons performing gynaecological oncology surgery who had undergone formal training versus 42.9% (15/35) of LMIC centres. Mean length of training was 2.7 years (SD = 0.7, range 2–4) and 3.8 years (SD = 1.2, range 2–6) in HICs/LMICs, respectively.

Supplementary Table S5 summarises centre demographics of hospitals participating in our study and hospitals who formally registered interest but did not participate. Irrespective of country income status, centres that did not participate had a smaller surgical annual case load and were more likely to be in a rural setting, except for non-participating HIC centres, which were all government-run academic/university hospitals. All participating centres had onsite level 2/3 critical care facilities, but fewer non-participating LMIC versus HIC centres had onsite facilities (30% versus 100% respectively). LMIC versus HIC centres, irrespective of study status, were less likely to participate in research studies.

Teams from 29 participating centres (11 LMIC centres from 9 countries, 18 HIC centres from 11 countries) were interviewed. Zero non-participating centres accepted our interview invitation. Interviews lasted 30–60 (mean = 45) min. Three categories of themes emerged: individual, organisational and national. Supplementary Table S6 summarises these interconnected themes.

### 3.1. Individual

Within this category, three themes emerged: altruism, burnout and culture. For LMICs, desire to offer optimum care/treatment to the local population was a strong facilitator for research participation. Burnout and research fatigue was a barrier for HICs due to the perceived overwhelming number of study participation invitations. Conversely, a barrier for LMICs was the culture of research exclusion and not being invited to participate. Supplementary Table S7 summarises other cited facilitators/barriers to research participation. A culture of not seeking medical attention was prevalent amongst LMICs, with a negative impact on surgical morbidity/mortality due to late disease presentations. Reasons cited included lack of personal finances to access healthcare, lack of available facilities and healthcare not being a priority.

### 3.2. Organisational

Three themes emerged: resource limitations, logistics and education. Resource limitations were commonly cited as a having a negative impact on delivery of high quality surgical care and detrimentally impacting morbidity/mortality. For LMICs, whilst level 2/3 care facilities were present, they were often not in use due to a lack of trained staff. LMIC diagnostic pathways were frequently prolonged due to an absence of screening/diagnostic services, resulting in a greater proportion of emergency presentations with advanced stage disease. In both LMICs/HICs, staff shortages (poor retention/emigration) were associated with poorer quality surgical care. For HICs, complicated/lengthy regulatory approval processes were a common barrier to research participation. An absence of curriculum based gynaecological oncology training programmes in LMICs was common. For most LMICs, gynaecological oncology was not considered a separate speciality, with surgeries often performed by general surgeons/surgical oncologists with informal gynaecological oncology training. Conversely, in HICs, gynaecological oncology was often a separate speciality with the presence of either national training programmes or centre specific fellowships. HICs with a national fellowship programme often had a well-established curriculum with training of individuals formally assessed annually. Whereas some European HICs that did not have a curriculum based national training programme had adopted the ESGO training curriculum. A common alternative for many non-ESGO, HIC centres were informal fellowships with no structured curriculum/no annual assessments.

### 3.3. National

Three themes emerged: war, pandemic and policy. Within LMICs in the midst of war, war had a negative impact on surgical morbidity/mortality. Reasons were multi-factorial including destruction of hospital infrastructure, international sanctions, and staff shortages due to emigration. COVID-19 had a negative impact in both LMICs/HICs and had resulted in changes in standard surgical care delivery pathways due to healthcare resource reallocation. National policy on women’s health had an impact on the delivery of surgical care, with some LMICs stating women’s health was not a national priority, resulting in underfunding of care.

## 4. Discussion

In this study, we show unadjusted short term post-operative major morbidity (8.2% versus 7%) and mortality (2.1% versus 1%) to be similar in both LMICs and HICs. Whilst major morbidity was reported to be higher and mortality double in LMICs, the differences in absolute numbers reported are small and may not translate into clinical significance. In our study involving 27 countries, gross domestic product (GDP) per capita ranged from 3.7 billion to 70.2 billion US dollars. Despite the huge economic differences between LMICs and HICs, it is reassuring that major morbidity/mortality was only marginally higher in LMICs. We have, however, identified multiple healthcare system factors that have the potential to improve quality of care and further reduce major morbidity in LMIC settings.

Adequate postgraduate medical training is vital, and when lacking, results in a deficiency of capacity to rescue after major complications. This excess mortality after gynaecological oncology surgery hinders cancer control LMIC efforts, and prevents patients, communities, and economies from realising the benefits of gynaecological cancer specific treatments. Our quantitative data show that in LMICs, 50% of gynaecological oncology surgery is performed by junior surgeons, who have three times the major morbidity rates of senior surgeons, with 70% being performed by non-gynaecological oncology surgeons with qualitative data suggesting that because gynaecological oncology is not a recognised speciality, the majority of LMIC surgeons do not receive adequate gynaecological oncology surgical training. Postgraduate surgical education is one mechanism to increase surgical capacity, and whilst data suggest structured LMIC training programmes exist for general surgery [9], our data show this not to be the case for gynaecological oncology. Our qualitative data propose that this may be due to the lack of prioritisation of women’s health despite the presence of multiple international programmes with the goal of improving women’s health worldwide through investment and prioritisation at national policy levels [10].

Whilst our quantitative data show that all LMIC centres participating in the study had level 2/3 critical care facilities, our qualitative data suggest that these facilities were often not in use due to lack of trained healthcare staff. Staff emigration was another reason for unusable critical care facilities. It is estimated globally that there is a shortage of 2.8 million physicians, with LMICs suffering the brunt of this burden. This unequitable distribution contributes to excess mortality and is exacerbated by physician brain drain, with HICs drawing up to 20% of their physician workforce from LMICs [11].

Longer and bloodier surgeries were independent predictors of increased morbidity (minor and major). Whilst this is in keeping with published literature [12,13], to our knowledge, this is the first study evaluating association with both intra-operative and post-operative minor/major morbidity. Given the adverse consequences of complications, decreased operative times and limiting blood loss should be universal goals for all surgeons, hospitals, and policymakers.

Elective planned surgery (versus emergency) was protective against intra-operative morbidity and is in keeping with general surgery data [14]. In our cohort, 2% versus 0.6% of gynaecological oncology surgery was emergency in LMICs/HICs, respectively. The link between advanced cancer stage and emergency surgery has been widely reported in the literature [15]. Our data suggest reasons for late presentation to be multifactorial. Our quantitative data show a positive correlation between advanced stage and individuals with poorer physical health with published literature reporting increased pre-operative fragility to be associated with a greater risk of major complications [16]. Our qualitative data show that inequalities in access to early detection, screening and deficiencies in patient health education contribute to late stage disease presentation and emergency surgery. The rising burden of advanced stage cancer in LMICs stresses already weak healthcare and economic infrastructure and poses unique challenges. Whilst the WHO recognises that effective management of cancer depends upon early detection, accurate diagnosis, and access to appropriate multimodal therapy, prioritising early detection cannot be assumed to be effective in LMICs, where limited downstream resources may be overwhelmed by inevitable increases in the number of diagnoses [15]. Therefore, in parallel to improving early detection infrastructure, there must be investment in resources to treat advanced stage disease, including investment in training clinicians to manage cases safely and effectively.

Minimal access surgery was found to be an independent variable in reducing intra-operative and minor post-operative morbidity. However, LMICs versus HICs were statistically significantly more likely to undergo open surgery (68.9%, 40.5%, *p* ≤ 0.001) than minimal access surgery (20.9%, 52.8%, *p* ≤ 0.001). Published data suggest that minimal access surgery may be safe, effective, feasible, and cost-effective in LMICs, although it often remains limited in its accessibility, acceptability, and quality [17]. Surgeons, policymakers, and manufacturers should focus on plans for sustainability, training and retention of minimal access surgery providers in LMICs [17].

In keeping with general surgical data [2], advanced stage disease was associated with lower rates of complete cytoreduction, but patients with better performance status were more likely to achieve complete cytoreduction. Patients from LMICs versus HICs had a statistically significantly poorer ECOG performance status and a larger proportion presented with FIGO stage III–IV uterus/cervix/vulva cancers.

In keeping with published data [18,19], our data also suggest a culture of exclusion of LMIC partners from high impact factor international gynaecological oncology research and the overburdening of HIC partners. LMICs account for 85% of the world’s population and 92% of the global disease burden, but only 10% of global funding for health research is devoted to addressing their health challenges [20]. In order for research findings to be truly generalizable and to maximise benefit, there must be LMIC representation.

Our data provide a global snapshot of the morbidity and mortality rates following gynaecological oncology surgery. The results highlight the urgent need for curriculum based gynaecological oncology training programmes and staff retention in LMICs to ensure safe and effective cancer care. With this large dataset, results could be used to coordinate plans and allocate resources to rescue and salvage detrimental effects.

Future research must focus on a detailed account of perioperative care practices, application of strategies to reduce morbidity, and improving access to cancer surgery which remains a barrier to effective healthcare in LMICs. Addressing such factors with interventional trials to build a global evidence base for the delivery of effective cancer surgery will improve overall cancer survival.

Study strengths include a prospective design, in-depth patient level and hospital level data collected from all seven world regions. Over 2000 variables were included, making the established GO SOAR database to our knowledge one of the richest datasets in gynaecological oncology that includes LMIC data. In addition, we collected qualitative data to elicit reasons for trends in observed quantitative data. Reporting of cancer stage, treatment, and outcomes was standardised, enabling comparisons between HIC/LMICs. Data quality was ensured though real time data entry quality assurance. Independent validation verified data accuracy and case ascertainment. Evaluation of LMIC surgical gynaecological oncology care has been hindered by deficiencies in high quality data. Our study adds to closing this knowledge gap and allows meaningful comparisons from multiple income settings.

Limitations include looking at short term post-operative outcomes. The GO SOAR database is continuing to capture HIC/LMIC data on longer term outcomes, including survival, which will help map global trends in gynaecological malignancies. The socioeconomic costs of undergoing cancer treatment are known to be significant [21,22], but were not measured.

## 5. Conclusions

We conclude that LMICs and HICs were associated with similar post-operative major morbidity. However, policymakers must balance investments in early detection and treatment with concurrent improvements in safe perioperative cancer care, including postgraduate training and retention of clinicians to further reduce morbidity in LMIC settings. Without these measures, mortality gains in gynaecological cancer control will not be fully realised.

## Figures and Tables

**Table 1:** Baseline characteristics by country income group.

	LMIC (n = 742)	HIC (n = 1078)	*p* Value
**Mean age (SD, range)**	56.6 years (12.3, 19–86)	61.8 years (13.4, 19–98)	<0.001
**Ethnicity**			
White	22.7% (168/741)	93.6% (999/1067)	<0.001
Asian	10% (74/741)	2.5% (27/1067)	
Black	10% (74/741)	2.2% (24/1067)	
Middle Eastern	52.9% (392/741)	0.7% (7/1067)	
Mixed	4.5% (33/741)	0.2% (2/1067)	
Other	0% (0/741)	0.7% (8/1067)	
**Mean BMI (SD, range)**	29.1 (7.1, 16–76.5)	28 (7.5, 14–77)	<0.001
**ASA**			
1	23.1% (168/726)	12.4% (130/1047)	<0.001
2	57.6% (418/726)	62.7% (656/1047)	
3	17.9% (130/726)	24.4% (255/1047)	
4	1.4% (10/726)	0.6% (6/1047)	
5	0% (0/726)	0% (0/1047)	
6	0% (0/726)	0% (0/1047)	
**ECOG**			
0	47.3% (346/731)	67.6% (696/1030)	<0.001
1	40.5% (296/731)	26.4% (272/1030)	
2	9.6% (70/731)	5.3% (55/1030)	
3	2.3% (17/731)	0.6% (6/1030)	
4	0.3% (2/731)	0.1% (1/1030)	
5	0% (0/731)	0% (0/1030)	
**Comorbidities**			
Yes (any)	64.7% (480/742)	71% (765/1078)	0.005
None	35.3% (262/742)	29% (313/1078)	
**Primary cancer**			
Ovary	38.3% (263/687)	39.1% (373/953)	1.00
Uterus	42.8% (294/687)	45% (429/953)	
Cervix	8.4% (58/687)	9.7% (92/953)	
Vulva	8.2% (56/687)	5.7% (54/953)	
Vagina	1.5% (10/687)	0.3% (3/953)	
GTD	0.9% (6/687)	0.2% (2/953)	
**Recurrent cancer**			
Ovary	52.7% (29/55)	64.8% (81/125)	0.461
Uterus	25.5% (14/55)	14.4% (18/125)	
Cervix	9.1% (5/55)	8% (10/125)	
Vulva	12.7% (7/55)	11.2% (14/125)	
Vagina	0%	0.8% (1/125)	
GTD	0%	0.8% (1/125)	
**COVID-19 status**			
positive	0.9% (7/738)	0.8% (9/1069)	0.025
negative	89.7% (662/738)	93.2% (996/1069)	
not tested	9.3% (69/738)	6% (64/1069)	
**Elective or emergency surgery**			
Elective	98% (723/738)	99.4% (1070/1076)	0.006
Emergency	2% (15/738)	0.6% (6/1076)	
**Surgical modality**			
Laparotomy	68.9% (506/734)	40.5% (435/1075)	<0.001
Laparoscopic	19.8% (145/734)	40.5% (435/1075)	
Robotic	1.1% (8/734)	12.3% (132/1075)	
Vaginal	1.6% (12/734)	0.5% (5/1075)	
Vulval	8.6% (63/734)	6.3% (68/1075)	
**Mean follow up (SD, range)**	58.7 days (53.6, 2–363)	55.7 days (51, 1–355)	0.003
**Lost to follow up**	6.1% (45/742)	4.8% (52/1078)	0.288

LMIC—low and middle income country; HIC—high income country; BMI—body mass index; ASA—American Society of Anesthesiologists physical status classification system; ECOG—Eastern Cooperative Oncology Group performance status scale; GTD—gestational trophoblastic disease; COVID-19—Coronavirus disease; SD—standard deviation.

**Table 2:** Unadjusted morbidity, mortality and complete macroscopic cytoreduction by tumour type and country income group.

		Intra-Operative Morbidity	Overall Post-Operative Morbidity	Minor Post- Operative Morbidity	Major Post-Operative Morbidity	Mortality	Complete Cytoreduction
**LMIC and HIC**	**Ovary** **N = 746**	18.2% (128/702)	41.8% (287/687)	32.6% (224/687)	9.1% (63/687)	1.5% (10/687)	* 89.7% (615/686)
	**Uterus** **N = 755**	9.3% (66/706)	24.7% (172/694)	19.3% (134/694)	5.5% (38/694)	2% (14/694)	96.4% (720/747)
	**Cervix** **N = 165**	15.7% (24/153)	34.7% (52/150)	28% (42/150)	6.7% (10/150)	0% (0/150)	94.3% (150/159)
	**Vulva** **N = 131**	3.9% (5/128)	41.7% (53/127)	29.9% (38/127)	11.8% (15/127)	0% (0/127)	96.9% (126/130)
	**Vagina** **N = 14**	21.4% (3/14)	42.9% (6/14)	42.9% (6/14)	0% (0/14)	0% (0/14)	92.9% (13/14)
	**GTD** **N = 9**	11.1% (1/9)	11.1% (1/9)	11.1% (1/9)	0% (0/9)	0% (0/9)	100% (9/9)
**LMIC**	**Ovary** **N = 292**	19.1% (50/262)	39.4% (100/254)	30.3% (77/254)	9.1% (23/254)	2% (5/254)	* 88.2% (224/254)
	**Uterus** **N = 308**	12.3% (36/293)	25.5% (84/286)	29.4% (64/286)	7% (20/286)	3.1% (9/286)	94.7% (287/303)
	**Cervix** **N = 63**	16.1% (9/56)	32.7% (18/55)	21.8% (12/55)	10.9% (6/55)	0% (0/55)	93.1% (54/58)
	**Vulva** **N = 63**	4.9% (3/61)	42.6% (26/61)	32.8% (20/61)	9.8% (6/61)	0% (0/61)	100% (62/62)
	**Vagina** **N = 10**	30% (3/10)	50% (5/10)	50% (5/10)	0% (0/10)	0% (0/10)	100% (10/10)
	**GTD** **N = 6**	0% (0/6)	0% (0/6)	0% (0/6)	0% (0/6)	0% (0/6)	100% (6/6)
**HIC**	**Ovary** **N = 454**	17.7% (78/440)	43.2% (187/433)	33.9% (147/433)	9.2% (40/433)	1.2% (5/433)	* 90.5% (391/432)
	**Uterus** **N = 447**	7.3% (30/413)	21.6% (88/408)	17.2% (70/408)	4.4% (18/408)	1.2% (5/408)	97.5% (433/444)
	**Cervix** **N = 102**	15.5% (15/97)	35.8% (34/95)	31.6% (30/95)	4.2% (4/95)	0% (0/95)	95% (96/101)
	**Vulva** **N = 68**	3% (2/67)	40.9% (27/66)	27.3% (18/66)	13.6% (9/66)	0% (0/66)	94.1% (64/68)
	**Vagina** **N = 4**	0% (0/4)	25% (1/4)	25% (1/4)	0% (0/4)	0% (0/4)	75% (3/4)
	**GTD** **N = 3**	33.3% (1/3)	33.3% (1/3)	33.3% (1/3)	0% (0/3)	0% (0/3)	100% (3/3)

* Complete cytoreduction rates for ovarian cancer include all primary, interval and delayed cytoreduction surgeries. Overall morbidity = Clavien–Dindo complication stage I–IV; minor morbidity = Clavien–Dindo complication grade I–II; major morbidity = Clavien–Dindo complication grade III–V. LMIC—low and middle income country; HIC—high income country; GTD—gestational trophoblastic disease.

**Table 3:** Adjusted three-level models for predictors of minor morbidity.

	Univariable OR (95%CI)	Multivariable OR (95%CI)	Multivariable Reduced OR (95%CI)	Multilevel OR (95%CI)
**Age**	1.059 (0.939–1.196), *p* = 0.353	1.09 (0.934–1.275), *p* = 0.276		
**Ethnicity**	0.8 (0.624–1.028), *p* = 0.08			
**BMI**	0.967 (0.855–1.09), *p* = 0.589	1.114 (0.965–1.284), *p* = 0.136	1.106 (0.963–1.269), *p* = 0.15	1.09 (0.943–1.261), *p* = 0.244
**ASA**	1.321 (1.095–1.595), *p* = 0.004	1.201 (0.946–1.525), *p* = 0.133	1.228 (0.993–1.52), *p* = 0.058	1.192 (0.95–1.496), *p* = 0.13
**ECOG**	1.115 (0.936–1.324), *p* = 0.219	0.938 (0.75–1.168), *p* = 0.567		
**Comorbidities**	0.839 (0.646–1.085), *p* = 0.185			
**Previous laparotomy**	1.337 (1.046–1.707), *p* = 0.02	1.208 (0.909–1.604), *p* = 0.191	1.232 (0.943–1.608), *p* = 0.125	1.2 (0.906–1.589), *p* = 0.204
**Previous laparoscopy**	1.682 (1.276–2.211), *p* < 0.001	1.454 (1.062–1.984), *p* = 0.019	1.479 (1.091–1.999), *p* = 0.011	1.435 (1.046–1.966), *p* = 0.025
**MDM discussion**	0.83 (0.593–1.176), *p* = 0.287			
**pre-operative imaging**	1.244 (0.787–2.036), *p* = 0.366			
**COVID-19**	6.176 (1.998–22.911), *p* = 0.003	5.136 (1.428–21.165), *p* = 0.015	5.857 (1.615–24.488), *p* = 0.009	5.025 (1.262–20.008), *p* = 0.022
**FIGO stage**	0.415 (0.324–0.53), *p* < 0.001	0.773 (0.568–1.055), *p* = 0.103	0.781 (0.584–1.048), *p* = 0.098	0.76 (0.56–1.031), *p* = 0.077
**Pre-operative mechanical bowel prophylaxis**	1.966 (1.542–2.512), *p* < 0.001	1.398 (1.046–1.868), *p* = 0.023	1.415 (1.08–1.856), *p* = 0.012	1.474 (1.054–2.061), *p* = 0.023
**Intra-operative antibiotics**	1.539 (1.047–2.322), *p* = 0.033	1.299 (0.851–2.028), *p* = 0.236		
**Peri-operative management plan**	0.898 (0.681–1.191), *p* = 0.45			
**Pre-operative haemoglobin**	1 (0.999–1), *p* = 0.232			
**GO surgeon vs. non-GO surgeon**	1.414 (0.914–2.261), *p* = 0.132			
**Trainee vs. consultant**	1.568 (1.014–2.39), *p* = 0.039	1.563 (0.956–2.523), *p* = 0.071	1.561 (0.963–2.494), *p* = 0.066	1.511 (0.884–2.584), *p* = 0.131
**Elective vs. emergency**	0.368 (0.114–1.184), *p* = 0.085			
**WHO checklist**	1.39 (0.891–2.244), *p* = 0.16			
**Length of surgery**	1.588 (1.414–1.788), *p* < 0.001	1.214 (1.042–1.416), *p* = 0.013	1.258 (1.097–1.444), *p* = 0.001	1.253 (1.066–1.472), *p* = 0.006
**Estimated blood loss**	1.713 (1.497–1.973), *p* < 0.001	1.234 (1.059–1.453), *p* = 0.009	1.228 (1.063–1.429), *p* = 0.006	1.274 (1.081–1.502), *p* = 0.004
**ITU recovery**	2.546 (1.944–3.333), *p* < 0.001	1.277 (0.909–1.785), *p* = 0.155		
**HDU recovery**	1.893 (1.401–2.548), *p* < 0.001	1.334 (0.942–1.88), *p* = 0.102		
**Enhanced recovery**	1.147 (0.882–1.499), *p* = 0.31			
**Prophylactic post-operative antibiotics**	1.501 (1.172–1.922), *p* = 0.001	0.93 (0.651–1.323), *p* = 0.687		
**Surgical drain**	2.179 (1.701–2.801), *p* < 0.001	1.135 (0.828–1.555), *p* = 0.432		
**Urinary catheter**	2.042 (1.245–3.538), *p* = 0.007	1.246 (0.71–2.289), *p* = 0.46		
**Complete cytoreduction**	0.571 (0.362–0.911), *p* = 0.017	0.93 (0.546–1.607), *p* = 0.791		
**WBI**	1.022 (0.801–1.308), *p* = 0.861	1.087 (0.761–1.556), *p* = 0.648	1.132 (0.845–1.519), *p* = 0.407	1.256 (0.703–2.244), *p* = 0.441
**Surgical modality**	0.405 (0.31–0.527), *p* < 0.001	0.58 (0.409–0.82), *p* = 0.002	0.502 (0.367–0.683), *p* < 0.001	0.522 (0.371–0.735), *p* < 0.001
**Centre size**	1.133 (0.873–1.465), *p* = 0.343			
**Intra-operative complication**	3.677 (2.677–5.059), *p* < 0.001	2.043 (1.415–2.945), *p* < 0.001	2.11 (1.471–3.021), *p* < 0.001	2.203 (1.498–3.241), *p* < 0.001
**Recurrence vs. primary surgery**	1.662 (1.151–2.379), *p* = 0.006	1.126 (0.728–1.73), *p* = 0.59		
**Primary tumour**				
**Cervix**	0.848 (0.55–0.397), *p* = 0.443	1.583 (0.973–2.55), *p* = 0.061		
**Uterus**	0.522 (0.55–0.397), *p* < 0.001	1.136 (0.796–1.624), *p* = 0.484		
**GTD**	0.344 (0.55–0.397), *p* = 0.324	0.876 (0.045–5.631), *p* = 0.905		
**Vagina**	1.473 (0.55–0.397), *p* = 0.514	1.515 (0.353–6.145), *p* = 0.562		
**Vulva**	1.031 (0.55–0.397), *p* = 0.9	1.801 (1.022–3.139), *p* = 0.039		

Adjusted three-level models (univariable, multivariable, multilevel) for predictors of a Clavien–Dindo complication grade I–II. N = 1350 with 366 events. LMIC—low and middle income country; HIC—high income country; BMI—body mass index; ASA—American Society of Anesthesiologists physical status classification system; ECOG—Eastern Cooperative Oncology Group performance status scale; GTD—gestational trophoblastic disease; COVID-19—Coronavirus disease; WHO—World Health Organization; MIS—Minimally invasive surgery (laparoscopy/robotic surgery); FIGO—The International Federation of Gynecology and Obstetrics; WBI—World Bank Institute. Age: linear variable; ethnicity: Caucasian vs. non-Caucasian; BMI: linear variable; ASA: linear variable; ECOG: linear variable; comorbidities: no comorbidity vs. presence of one or more comorbidities; previous laparotomy: yes vs. no; previous laparoscopic abdominal surgery: yes vs. no; MDM discussion: yes vs. no; pre-operative imaging: yes vs. no; pre-operative COVID-19 status: positive vs. negative/not tested; FIGO stage: I–II vs. III–IV; pre-operative mechanical prophylaxis: yes vs. no; intra-operative antibiotics: yes vs. no; peri-operative management plan: yes vs. no; pre-operative haemoglobin: linear variable; GO surgeon vs. non-GO surgeon; trainee vs. consultant: registrar/resident vs. attending/consultant; elective vs. emergency; WHO checklist: yes vs. no; length of surgery: linear variable; estimated blood loss: linear variable; ITU recovery: yes vs. no; HDU recovery: yes vs. no; enhanced recovery: yes vs. no; prophylactic post-operative antibiotics: yes vs. no; surgical drain: yes vs. no; indwelling urinary catheter: yes vs. no; complete macroscopic cytoreduction: yes vs. no; WBI: HIC vs. LMIC; surgical modality: laparoscopic/robotic vs. laparotomy/MIS converted to laparotomy; centre size: small/medium vs. large; intra-operative complication: yes vs. no; recurrence surgery vs. primary surgery; cervix: cervix vs. ovary; uterus: uterus vs. ovary; GTD: GTD vs. ovary; vagina: vagina vs. ovary; vulva: vulva vs. ovary.

**Table 4:** Adjusted three-level models for predictors of major morbidity.

	Univariable OR (95%CI)	Multivariable OR (95%CI)	Multivariable Reduced OR (95%CI)	Multilevel OR (95%CI)
**Age**	1.082 (0.889–1.323), *p* = 0.439	1.086 (0.873–1.359), *p* = 0.464		
**Ethnicity**	0.918 (0.613–1.393), *p* = 0.683			
**BMI**	0.737 (0.581–0.919), *p* = 0.009	0.781 (0.603–0.993), *p* = 0.051	0.779 (0.605–0.986), *p* = 0.045	0.778 (0.605–1.001), *p* = 0.051
**ASA**	1.112 (0.82–1.507), *p* = 0.492			
**ECOG**	1.316 (1.004–1.701), *p* = 0.04	1.176 (0.864–1.581), *p* = 0.291	1.238 (0.924–1.637), *p* = 0.143	1.226 (0.895–1.681), *p* = 0.205
**Comorbidities**	0.996 (0.651–1.5), *p* = 0.985			
**Previous laparotomy**	1.434 (0.964–2.125), *p* = 0.073			
**Previous laparoscopy**	1.291 (0.815–1.994), *p* = 0.261			
**MDM discussion**	0.968 (0.564–1.773), *p* = 0.91			
**pre-operative imaging**	1.783 (0.784–5.138), *p* = 0.218			
**COVID-19**	0.948 (0.052–4.881), *p* = 0.96	0.506 (0.027–2.91), *p* = 0.531		
**FIGO stage**	0.466 (0.313–0.691), *p* < 0.001	0.92 (0.572–1.487), *p* = 0.731		
**Pre-operative mechanical bowel prophylaxis**	1.22 (0.824–1.808), *p* = 0.32			
**Intra-operative antibiotics**	1.29 (0.706–2.598), *p* = 0.439			
**Peri-operative management plan**	0.845 (0.548–1.337), *p* = 0.458			
**Pre-operative haemoglobin**	0.818 (0.467–1.096), *p* = 0.374			
**GO surgeon vs. non-GO surgeon**	0.786 (0.434–1.55), *p* = 0.455			
**Trainee vs. consultant**	2.17 (1.165–3.805), *p* = 0.01	2.583 (1.334–4.749), *p* = 0.003	2.568 (1.341–4.669), *p* = 0.003	2.982 (1.509–5.894), *p* = 0.002
**Elective vs. emergency**	0.435 (0.113–2.851), *p* = 0.286			
**WHO checklist**	1.67 (0.777–4.35), *p* = 0.235			
**Length of surgery**	1.568 (1.343–1.825), *p* < 0.001	1.301 (1.064–1.58), *p* = 0.009	1.351 (1.123–1.615), *p* = 0.001	1.37 (1.128–1.664), *p* = 0.002
**Estimated blood loss**	1.556 (1.346–1.808), *p* < 0.001	1.298 (1.093–1.546), *p* = 0.003	1.346 (1.146–1.584), *p* < 0.001	1.398 (1.175–1.664), *p* < 0.001
**ITU recovery**	2.542 (1.685–3.804), *p* < 0.001	1.246 (0.751–2.038), *p* = 0.387		
**HDU recovery**	1.185 (0.703–1.915), *p* = 0.504			
**Enhanced recovery**	1.153 (0.753–1.811), *p* = 0.522			
**Prophylactic post-operative antibiotics**	1.36 (0.908–2.021), *p* = 0.131			
**Surgical drain**	2.209 (1.463–3.401), *p* < 0.001	1.353 (0.836–2.207), *p* = 0.22	1.421 (0.894–2.284), *p* = 0.141	1.608 (0.949–2.726), *p* = 0.078
**Urinary catheter**	1.963 (0.864–5.647), *p* = 0.15			
**Complete cytoreduction**	0.491 (0.266–0.979), *p* = 0.031	0.804 (0.397–1.737), *p* = 0.558		
**WBI**	0.906 (0.611–1.353), *p* = 0.625	0.745 (0.471–1.179), *p* = 0.207	0.764 (0.497–1.179), *p* = 0.222	1.067 (0.521–2.186), *p* = 0.86
**Surgical modality**	0.664 (0.433–1), *p* = 0.054			
**Centre size**	0.702 (0.437–1.093), *p* = 0.128			
**Intra-operative complication**	2.352 (1.463–3.688), *p* < 0.001	1.165 (0.676–1.954), *p* = 0.571		
**Recurrence vs. primary surgery**	1.039 (0.529–1.873), *p* = 0.904	0.884 (0.437–1.655), *p* = 0.715		
**Primary tumour**	Excluded because no complications in vagina/GTD tumours.			

Adjusted three-level models (univariable, multivariable, multilevel) for predictors of a Clavien–Dindo complication grade III–V. N = 1350 with 109 events. LMIC—low and middle income country; HIC—high income country; BMI—body mass index; ASA—American Society of Anesthesiologists physical status classification system; ECOG—Eastern Cooperative Oncology Group performance status scale; GTD—gestational trophoblastic disease; COVID-19—Coronavirus disease; WHO—World Health Organization; MIS: Minimally invasive surgery (laparoscopy/robotic surgery); FIGO—The International Federation of Gynecology and Obstetrics; WBI—World Bank Institute. Age: linear variable; ethnicity: Caucasian vs. non-Caucasian; BMI: linear variable; ASA: linear variable; ECOG: linear variable; comorbidities: no comorbidity vs. presence of one or more comorbidities; previous laparotomy: yes vs. no; previous laparoscopic abdominal surgery: yes vs. no; MDM discussion: yes vs. no; pre-operative imaging: yes vs. no; pre-operative COVID-19 status: positive vs. negative/not tested; FIGO stage: I–II vs. III–IV; pre-operative mechanical prophylaxis: yes vs. no; intra-operative antibiotics: yes vs. no; peri-operative management plan: yes vs. no; pre-operative haemoglobin: linear variable; GO surgeon vs. non-GO surgeon; trainee vs. consultant: registrar/resident vs. attending/consultant; elective vs. emergency; WHO checklist: yes vs. no; length of surgery: linear variable; estimated blood loss: linear variable; ITU recovery: yes vs. no; HDU recovery: yes vs. no; enhanced recovery: yes vs. no; prophylactic post-operative antibiotics: yes vs. no; surgical drain: yes vs. no; indwelling urinary catheter: yes vs. no; complete macroscopic cytoreduction: yes vs. no; WBI: HIC vs. LMIC; surgical modality: laparoscopic/robotic vs. laparotomy/MIS converted to laparotomy; centre size: small/medium vs. large; intra-operative complication: yes vs. no; recurrence surgery vs. primary surgery.

**Table 5:** Adjusted three-level models for predictors of tumour resectability.

	Univariable OR (95%CI)	Multivariable OR (95%CI)	Multivariable Reduced OR (95%CI)	Multilevel OR (95%CI)
**Age**	0.803 (0.634–1.009), *p* = 0.064	0.986 (0.755–1.278), *p* = 0.919		
**Ethnicity**	1.045 (0.649–1.653), *p* = 0.852			
**BMI**	1.27 (0.996–1.652), *p* = 0.064	1.114 (0.85–1.489), *p* = 0.45		
**ASA**	0.588 (0.417–0.829), *p* = 0.002	0.809 (0.535–1.221), *p* = 0.313		
**ECOG**	0.525 (0.402–0.691), *p* < 0.001	0.589 (0.417–0.837), *p* = 0.003	0.529 (0.392–0.717), *p* < 0.001	0.572 (0.406–0.806), *p* = 0.001
**Comorbidities**	1.091 (0.683–1.786), *p* = 0.721			
**MDT discussion**	1.215 (0.63–2.172), *p* = 0.534			
**Pre-operative imaging**	0.285 (0.046–0.922), *p* = 0.083			
**FIGO stage**	20.766 (10.187–49.923), *p* < 0.001	20.932 (9.974–51.317), *p* < 0.001	20.197 (9.873–48.676), *p* < 0.001	23.316 (10.597–51.297), *p* < 0.001
**GO surgeon vs. non-GO surgeon**	1.778 (0.893–3.264), *p* = 0.079			
**Trainee vs. consultant**	0.558 (0.291–1.184), *p* = 0.1			
**Length of surgery**	0.808 (0.674–0.985), *p* = 0.027	1.19 (0.932–1.552), *p* = 0.179		
**Estimated blood loss**	0.753 (0.648–0.879), *p* < 0.001	0.936 (0.776–1.146), *p* = 0.5		
**WBI**	0.937 (0.589–1.471), *p* = 0.781	0.926 (0.542–1.562), *p* = 0.775	0.88 (0.529–1.44), *p* = 0.615	0.989 (0.418–2.338), *p* = 0.98
**Centre size**	1.396 (0.847–2.396), *p* = 0.206			

Adjusted three-level models (univariable, multivariable, multilevel) for predictors of complete macroscopic cytoreduction. N = 1350 (83 complete cytoreduction, 1267 incomplete cytoreduction). LMIC—low and middle income country; HIC—high income country; BMI—body mass index; ASA—American Society of Anesthesiologists physical status classification system; ECOG—Eastern Cooperative Oncology Group performance status scale; MDT—multidisciplinary team/tumour board; FIGO—The International Federation of Gynecology and Obstetrics; WBI—World Bank Institute. Age: linear variable; ethnicity: Caucasian vs. non-Caucasian; BMI: linear variable; ASA: linear variable; ECOG: linear variable; comorbidities: no comorbidity vs. presence of one or more comorbidities; previous laparotomy: yes vs. no; previous laparoscopic abdominal surgery: yes vs. no; MDT discussion: yes vs. no; pre-operative imaging: yes vs. no; FIGO stage: I-II vs. III-IV; pre-operative mechanical prophylaxis: yes vs. no; intra-operative antibiotics: yes vs. no; peri-operative management plan: yes vs. no; pre-operative haemoglobin: linear variable; GO surgeon vs. non-GO surgeon; trainee vs. consultant: registrar/resident vs. attending/consultant; length of surgery: linear variable; estimated blood loss: linear variable; WBI: HIC vs. LMIC; centre size: small/medium vs. large.

## Data Availability

Relevant anonymised data can be obtained on reasonable request from the corresponding author.

## Supplementary Materials

The following supporting information can be downloaded at: https://www.mdpi.com/article/10.3390/cancers15205001/s1, Table S1: Proportion of patients by cancer stage per tumour and country income group. Table S2: Linear correlation between cancer stage per tumour group and operative risk and performance status. Table S3: Adjusted three-level models for predictors of intra-operative morbidity. Table S4: Adherence to tumour specific audit standards by country income group. Table S5: Centre demographics per income country setting and study participation status. Table S6: Themes associated with research, training, surgical morbidity and mortality. Table S7: Facilitators and barriers to research participation. Supplementary File S1: GO SOAR database data entry points. Supplementary File S2: Topic guide for qualitative sub-study: GO SOAR1. Supplementary File S3: GO SOAR Collaborators.
