# Supplementary material for: International Variations in Surgical Morbidity and Mortality Post Gynaecological Oncology Surgery: A Global Gynaecological Oncology Surgical Outcomes Collaborative Led Study (GO SOAR1)
Source: Cancers (Basel). 2023 Oct 16;15(20):5001. doi: 10.3390/cancers15205001 (PMC10605858; doi:10.3390/cancers15205001)
Supplement: Supplementary file 1 [file cancers-15-05001-s001.zip › Supplementary_File_S1.pdf]

Data Dictionary Codebook

09/01/2023 12:29pm

#	Variable / Field Name	Field Label *Field Note*	Field Attributes (Field Type, Validation, Choices, Calculations, etc.)
Instrument: **Registration** (registration) Enabled as survey			
1	[record_id]	Record ID	text

2	[received_by]	Country<br>*Initials*	dropdown, Required

1	Afghanistan
2	Albania
3	Algeria
4	Andorra
5	Angola
6	Antigua and Barbuda
7	Argentina
8	Armenia
9	Australia
10	Austria
11	Azerbaijan
12	The Bahamas
13	Bahrain
14	Bangladesh
15	Barbados
16	Belarus
17	Belgium
18	Belize
19	Benin
20	Bhutan
21	Bolivia
22	Bosnia and Herzegovina
23	Botswana
24	Brazil
25	Brunei
26	Bulgaria
27	Burkina Faso
28	Burundi
29	Cabo Verde
30	Cambodia
31	Cameroon
32	Canada
33	Central African Republic
34	Chad
35	Chile
36	China
37	Colombia
38	Comoros
Congo	Republic of the
39	Costa Rica
40	Côte d'Ivoire
41	Croatia
42	Cuba
43	Cyprus
44	Czech Republic
45	Denmark
46	Djibouti
47	Dominica
48	Dominican Republic
49	East Timor (Timor-Leste)
50	Ecuador
51	Egypt

52	El Salvador
53	Equatorial Guinea
54	Eritrea
55	Estonia
56	Eswatini
57	Ethiopia
58	Fiji
59	Finland
60	France
61	Gabon
62	The Gambia
63	Georgia
64	Germany
65	Ghana
66	Greece
67	Grenada
68	Guatemala
69	Guinea
70	Guinea-Bissau
71	Guyana
72	Haiti
73	Honduras
74	Hungary
75	Iceland
76	India
77	Indonesia
78	Iran
79	Iraq
80	Ireland
81	Israel
82	Italy
83	Jamaica
84	Japan
85	Jordan
86	Kazakhstan
87	Kenya
88	Kiribati
Korea	South
89	Kosovo
90	Kuwait
91	Kyrgyzstan
92	Laos
93	Latvia
94	Lebanon
95	Lesotho
96	Liberia
97	Libya
98	Liechtenstein
99	Lithuania
100	Luxembourg
101	Madagascar
102	Malawi
103	Malaysia

104	Maldives
105	Mali
106	Malta
107	Marshall Islands
108	Mauritania
109	Mauritius
110	Mexico
Micronesia	Federated States of
111	Moldova
112	Monaco
113	Mongolia
114	Montenegro
115	Morocco
116	Mozambique
117	Myanmar (Burma)
118	Namibia
119	Nauru
120	Nepal
121	Netherlands
122	New Zealand
123	Nicaragua
124	Niger
125	Nigeria
126	North Macedonia
127	Norway
128	Oman
129	Pakistan
130	Palau
131	Panama
132	Papua New Guinea
133	Paraguay
134	Peru
135	Philippines
136	Poland
137	Portugal
138	Qatar
139	Romania
140	Russia
141	Rwanda
142	Saint Kitts and Nevis
143	Saint Lucia
144	Saint Vincent and the Grenadines
145	Samoa
146	San Marino
147	Sao Tome and Principe
148	Saudi Arabia
149	Senegal
150	Serbia
151	Seychelles
152	Sierra Leone
153	Singapore
154	Slovakia
155	Slovenia

			156 Solomon Islands<br>157 Somalia<br>158 South Africa<br>159 Spain<br>160 Sri Lanka<br>161 Sudan<br>Sudan South<br>162 Suriname<br>163 Sweden<br>164 Switzerland<br>165 Syria<br>166 Taiwan<br>167 Tajikistan<br>168 Tanzania<br>169 Thailand<br>170 Togo<br>171 Tonga<br>172 Trinidad and Tobago<br>173 Tunisia<br>174 Turkey<br>175 Turkmenistan<br>176 Tuvalu<br>177 Uganda<br>178 Ukraine<br>179 United Arab Emirates<br>180 United Kingdom<br>181 United States<br>182 Uruguay<br>183 Uzbekistan<br>184 Vanuatu<br>185 Vatican City<br>186 Venezuela<br>187 Vietnam<br>188 Yemen<br>189 Zambia<br>190 Zimbabwe<br>191 Palestine
3	[site_name]	Healthcare facility name	text, Required, Identifier
4	[hcf_address]	Healthcare facility address	text, Required, Identifier
5	[pi]	Principal investigator<br>*First and Last Name*	text, Required, Identifier
6	[phone_number]	Contact Phone Number	text, Required, Identifier
7	[email]	Contact Email	text (email), Required, Identifier
8	[registration_complete]	Section Header: *Form Status*<br>Complete?	dropdown<br>0 Incomplete<br>1 Unverified<br>2 Complete

Instrument: **Eligibility and Feasibility** (eligibility_and_feasibility) Enabled as survey

9	[site_type]	Type of institution	dropdown, Required<br>1 Government health centre<br>2 Private health centre<br>3 Government district/rural hospital<br>4 Private district/rural hospital<br>5 Government academic/university hospital<br>6 Private academic/university hospital<br>7 Government provincial tertiary hospital<br>8 Private provincial tertiary hospital<br>9 NGO/Mission Hospital
10	[go_op]	Gynaecological cancers operated on (select all that apply)	checkbox, Required<br>1 go_op___1 Primary tubo-ovarian/peritoneal<br>2 go_op___2 Primary endometrial<br>3 go_op___3 Primary cervical<br>4 go_op___4 Primary vulval<br>5 go_op___5 Primary vaginal<br>6 go_op___6 Primary gestational trophoblastic<br>7 go_op___7 Recurrent tubo-ovarian/peritoneal<br>8 go_op___8 Recurrent endometrial<br>9 go_op___9 Recurrent cervical<br>10 go_op___10 Recurrent vulval<br>11 go_op___11 Recurrent vaginal<br>12 go_op___12 Recurrent gestational trophoblastic<br>13 go_op___13 None of the above
11	[typ_surg]	Type of gynaecological oncology surgery performed (select all that apply)	checkbox, Required<br>1 typ_surg___1 Elective<br>2 typ_surg___2 Emergency<br>3 typ_surg___3 Curative<br>4 typ_surg___4 Palliative<br>5 typ_surg___5 None of the above - no gynaecological oncology surgery performed
12	[surg_mod]	Gynaecological oncology surgical treatment modalities (select all that apply)	checkbox, Required<br>1 surg_mod___1 Open<br>2 surg_mod___2 Laparoscopic<br>3 surg_mod___3 Robotic<br>4 surg_mod___4 Vaginal<br>5 surg_mod___5 None of the above - no gynaecological oncology surgery performed
13	[tot_new]	Total number of new referrals received/new cases seen per year	text (number), Required
14	[numb_op]	Total number of gynaecological oncology surgeries performed per year	text (number), Required
15	[numb_oc]	Number of tubo-ovarian/primary peritoneal cancer surgeries performed per year	text (number), Required
16	[numb_ec]	Number of endometrial cancer surgeries performed per year	text (number), Required
17	[numb_cc]	Number of cervical cancer surgeries performed per year	text (number), Required
18	[numb_vu]	Number of vulval cancer surgeries performed per year	text (number), Required
19	[numb_vag]	Number of vaginal cancer surgeries performed per year	text (number), Required
20	[numb_gtd]	Number of gestational trophoblastic cancer surgeries performed per year	text (number), Required
21	[beds]	Number of gynaecological oncology inpatient beds	text (number), Required
22	[numb_cons]	Number of certified/accredited consultant gynaecological oncology specialists	text (number), Required

23	[numb_fell]	Number of gynaecological oncology fellows/trainees	text (number), Required
24	[hdu_itu_yn]	High dependency unit/intensive care unit facilities available on site	yesno, Required<br>1 Yes<br>0 No
25	[res]	Does your hospital/unit/centre participate in gynaecological oncology clinical trials/research studies?	yesno, Required<br>1 Yes<br>0 No
26	[approval]	I confirm that I have obtained all the necessary approvals from my local review body to take part in this study. I take sole responsibility for this.	checkbox, Required<br>1 approval___1 Yes
27	[app_doc]	Please upload approval documentation from your local review body	file, Required, Identifier
28	[cons_data]	I agree that I/my team will accurately enter gynaecological oncology surgical outcome data from my institution for a minimum of 30 consecutive days	checkbox, Required, Identifier<br>1 cons_data___1 Yes
29	[train]	I agree to ensure I/my team read all training material provided by the Global Gynaecological Oncology Surgical Outcomes Collaborative central coordinating team	checkbox, Required<br>1 train___1 Yes
30	[poc_name]	Name of local point of contact	text, Required
31	[poc_email]	Email address of local point of contact	text (email), Required, Identifier
32	[signature]	Signature	file (signature), Required
33	[eligibility_and_feasibility_complete]	Section Header: *Form Status*<br>Complete?	dropdown<br>0 Incomplete<br>1 Unverified<br>2 Complete
Instrument: **World Health Organization Tool for Situational Analysis to Assess Emergency and Essential Surgical Care** (world_health_organization_tool_for_situational_ana) Enabled as survey			
34	[txt_who]	Population served by this health care facility	text (number)
35	[numb_beds]	Number of beds	text (number)
36	[adms]	Number of total admissions in one year	text (number)
37	[op]	Number of total outpatients in one year	text (number)
38	[or]	Number of total functioning operating rooms (major & minor)	text (number)
39	[pat]	Number of patients at this facility requiring major & minor surgical (including obs/gyn) procedures per year	text (number)
40	[child]	Number of children (aged less than 15 years) at this facility requiring surgical procedures per year	text (number)
41	[refer]	Number of patients to this facility that you refer for surgical intervention to a higher level facility per year	text (number)
42	[travel]	How far (in km) does the average patient travel to get to your health facility for surgical services?	text (number)
43	[ss_travel]	If you do not provide surgical services, how far (in km) does the average patient travel to access surgical services?	text (number)
44	[o2]	Do you have oxygen cylinder or concentrator supply with mask and tubing?	radio (Matrix)<br>1 Not available<br>2 Sometimes<br>3 All the time
45	[h2o]	Do you have running water?	radio (Matrix)<br>1 Not available<br>2 Sometimes<br>3 All the time
46	[elec]	Do you have an electricity source/operational power generator?	radio (Matrix)<br>1 Not available<br>2 Sometimes<br>3 All the time

47	[anaes]	Do you have a functioning anaesthesia machine?	radio (Matrix)<br>1 Not available<br>2 Sometimes<br>3 All the time
48	[mr]	Do you keep medical records?	radio (Matrix)<br>1 Not available<br>2 Sometimes<br>3 All the time
49	[emer_care]	Do you have an area designated for Emergency care?	radio (Matrix)<br>1 Not available<br>2 Sometimes<br>3 All the time
50	[po_care]	Do you have an area designated for Postoperative care?	radio (Matrix)<br>1 Not available<br>2 Sometimes<br>3 All the time
51	[guide]	Do you have management guidelines available for Emergency care?	radio (Matrix)<br>1 Not available<br>2 Sometimes<br>3 All the time
52	[mx_gude_surg]	Do you have management guidelines available for Surgery?	radio (Matrix)<br>1 Not available<br>2 Sometimes<br>3 All the time
53	[mx_uide_anaes]	Do you have management guidelines available for Anaesthesia?	radio (Matrix)<br>1 Not available<br>2 Sometimes<br>3 All the time
54	[mx_guide_pain]	Do you have management guidelines available for Pain Relief?	radio (Matrix)<br>1 Not available<br>2 Sometimes<br>3 All the time
55	[bld_bnk]	Do you have a blood bank available at the facility?	radio (Matrix)<br>1 Not available<br>2 Sometimes<br>3 All the time
56	[hb_urine]	Do you have a facility to test haemoglobin & urine?	radio (Matrix)<br>1 Not available<br>2 Sometimes<br>3 All the time
57	[xray]	Do you have a functioning X-ray machine available?	radio (Matrix)<br>1 Not available<br>2 Sometimes<br>3 All the time
58	[oxim]	Do you have a functioning pulse oximeter available?	radio (Matrix)<br>1 Not available<br>2 Sometimes<br>3 All the time
59	[surg_ft]	Number of full time surgeons (qualified)	text (number)
60	[surg_pt]	Number of part time surgeons (qualified)	text (number)
61	[anaes_ft]	Number of full time anaesthesiologist physicians (qualified)	text (number)
62	[anaes_pt]	Number of part time anaesthesiologist physicians (qualified)	text (number)
63	[obgyn_ft]	Number of full time obstetricians/gynecologists (qualified)	text (number)

64	[obgyn_pt]	Number of part time obstetricians/gynecologists (qualified)	text (number)
65	[doc_surg_ft]	Number of full time general doctors providing surgery (including obstetrics)	text (number)
66	[doc_surg_pt]	Number of part time general doctors providing surgery (including obstetrics)	text (number)
67	[doc_anaes_ft]	Number of full time general doctors providing anaesthesia	text (number)
68	[doc_anaes_pt]	Number of part time general doctors providing anaesthesia	text (number)
69	[ncmo_anaes_ft]	Number of full time nurses/clinical medical officers providing anaesthesia	text (number)
70	[ncmo_anaes_pt]	Number of part time nurses/clinical medical officers providing anaesthesia	text (number)
71	[cmo_surg_ft]	Number of full time clinical medical officers providing surgery (including obstetrics)	text (number)
72	[cmo_surg_pt]	Number of part time clinical medical officers providing surgery (including obstetrics)	text (number)
73	[parmw_ft]	Number of full time paramedics/midwives	text (number)
74	[parmw_pt]	Number of part time paramedics/midwives	text (number)
75	[ofr]	Section Header: *Do you perform these procedures*<br>Obstetric fistula repair	radio (Matrix)<br>1 Yes we perform these procedures<br>2 No we do not perform these procedures
76	[tl]	Tubal ligation/vasectomy	radio (Matrix)<br>1 Yes we perform these procedures<br>2 No we do not perform these procedures
77	[bx]	Biopsy (lymph node, mass, other)	radio (Matrix)<br>1 Yes we perform these procedures<br>2 No we do not perform these procedures
78	[app]	Appendectomy	radio (Matrix)<br>1 Yes we perform these procedures<br>2 No we do not perform these procedures
79	[hern]	Hernia repair (strangulated, elective, congenital)	radio (Matrix)<br>1 Yes we perform these procedures<br>2 No we do not perform these procedures
80	[hyd]	Hydrocelectomy	radio (Matrix)<br>1 Yes we perform these procedures<br>2 No we do not perform these procedures
81	[cyst]	Cystostomy	radio (Matrix)<br>1 Yes we perform these procedures<br>2 No we do not perform these procedures
82	[usd]	Urethral stricture dilatation	radio (Matrix)<br>1 Yes we perform these procedures<br>2 No we do not perform these procedures
83	[laparot]	Laparotomy (uterine rupture, ectopic pregnancy, acute abdomen, intestinal obstruction, perforation, injuries)	radio (Matrix)<br>1 Yes we perform these procedures<br>2 No we do not perform these procedures
84	[mc]	Male circumcision	radio (Matrix)<br>1 Yes we perform these procedures<br>2 No we do not perform these procedures
85	[nsurg]	Neonatal surgery: abdominal wall defect, colostomy imperforate anus, intussusceptions	radio (Matrix)<br>1 Yes we perform these procedures<br>2 No we do not perform these procedures
86	[clrpr]	Cleft lip repair	radio (Matrix)<br>1 Yes we perform these procedures<br>2 No we do not perform these procedures

87	[club]	Clubfoot repair	radio (Matrix)<br>1 Yes we perform these procedures<br>2 No we do not perform these procedures
88	[skn_g]	Contracture release/skin grafting	radio (Matrix)<br>1 Yes we perform these procedures<br>2 No we do not perform these procedures
89	[frac_clo]	Closed treatment of fracture	radio (Matrix)<br>1 Yes we perform these procedures<br>2 No we do not perform these procedures
90	[frac_op]	Open treatment of fracture	radio (Matrix)<br>1 Yes we perform these procedures<br>2 No we do not perform these procedures
91	[jnt]	Joint dislocation treatment	radio (Matrix)<br>1 Yes we perform these procedures<br>2 No we do not perform these procedures
92	[ostm]	Drainage of osteomyelitis/septic arthritis	radio (Matrix)<br>1 Yes we perform these procedures<br>2 No we do not perform these procedures
93	[amptn]	Amputation	radio (Matrix)<br>1 Yes we perform these procedures<br>2 No we do not perform these procedures
94	[cs]	Cataract surgery	radio (Matrix)<br>1 Yes we perform these procedures<br>2 No we do not perform these procedures
95	[reg_anaes]	Regional anaesthesia blocks	radio (Matrix)<br>1 Yes we perform these procedures<br>2 No we do not perform these procedures
96	[sp_anaes]	Spinal anaesthesia	radio (Matrix)<br>1 Yes we perform these procedures<br>2 No we do not perform these procedures
97	[ket_anaes]	Ketamine intravenous anaesthesia	radio (Matrix)<br>1 Yes we perform these procedures<br>2 No we do not perform these procedures
98	[ga_anaes]	General anaesthesia inhalational	radio (Matrix)<br>1 Yes we perform these procedures<br>2 No we do not perform these procedures
99	[resus]	Resuscitation (airway, hemorrhage, peripheral percutaneous intravenous access, peripheral venous cut down)	radio (Matrix)<br>1 Yes we perform these procedures<br>2 No we do not perform these procedures
100	[ct_air]	Cricothyroidotomy/Tracheostomy	radio (Matrix)<br>1 Yes we perform these procedures<br>2 No we do not perform these procedures
101	[cti]	Chest tube insertion	radio (Matrix)<br>1 Yes we perform these procedures<br>2 No we do not perform these procedures
102	[fb]	Removal of foreign body (throat/eye/ear/nose)	radio (Matrix)<br>1 Yes we perform these procedures<br>2 No we do not perform these procedures
103	[abm]	Acute burn management	radio (Matrix)<br>1 Yes we perform these procedures<br>2 No we do not perform these procedures

104	[iandd]	Incision & drainage of abscess	radio (Matrix)<br>1 Yes we perform these procedures<br>2 No we do not perform these procedures
105	[sut]	Suturing (for wounds, episiotomy, cervical & vaginal lacerations)	radio (Matrix)<br>1 Yes we perform these procedures<br>2 No we do not perform these procedures
106	[wb]	Wound debridement	radio (Matrix)<br>1 Yes we perform these procedures<br>2 No we do not perform these procedures
107	[lscs]	Caesarean section	radio (Matrix)<br>1 Yes we perform these procedures<br>2 No we do not perform these procedures
108	[dandc]	Dilatation & curettage/vacuum extraction (obstetrics/gyn)	radio (Matrix)<br>1 Yes we perform these procedures<br>2 No we do not perform these procedures
109	[resus2]	Section Header: *Do you refer these procedures*<br>Resuscitation (airway, hemorrhage, peripheral percutaneous intravenous access, peripheral venous cut down)	radio (Matrix)<br>1 Yes<br>2 No
110	[ct_air2]	Cricothyroidotomy/Tracheostomy	radio (Matrix)<br>1 Yes<br>2 No
111	[cti2]	Chest tube insertion	radio (Matrix)<br>1 Yes<br>2 No
112	[fb2]	Removal of foreign body (throat/eye/ear/nose)	radio (Matrix)<br>1 Yes<br>2 No
113	[abm2]	Acute burn management	radio (Matrix)<br>1 Yes<br>2 No
114	[id2]	Incision & drainage of abscess	radio (Matrix)<br>1 Yes<br>2 No
115	[sut2]	Suturing (for wounds, episiotomy, cervical & vaginal lacerations)	radio (Matrix)<br>1 Yes<br>2 No
116	[wd2]	Wound debridement	radio (Matrix)<br>1 Yes<br>2 No
117	[cs2]	Caesarean section	radio (Matrix)<br>1 Yes<br>2 No
118	[dac2]	Dilatation & curettage/vacuum extraction (obstetrics/gyn)	radio (Matrix)<br>1 Yes<br>2 No
119	[ofr2]	Obstetric fistula repair	radio (Matrix)<br>1 Yes<br>2 No
120	[tlv2]	Tubal ligation/vasectomy	radio (Matrix)<br>1 Yes<br>2 No

121	[bx2]	Biopsy (lymph node, mass, other)	radio (Matrix)<br>1 Yes<br>2 No
122	[app2]	Appendectomy	radio (Matrix)<br>1 Yes<br>2 No
123	[hr2]	Hernia repair (strangulated, elective, congenital)	radio (Matrix)<br>1 Yes<br>2 No
124	[hyd2]	Hydrocelectomy	radio (Matrix)<br>1 Yes<br>2 No
125	[cyst2]	Cystostomy	radio (Matrix)<br>1 Yes<br>2 No
126	[usd2]	Urethral stricture dilatation	radio (Matrix)<br>1 Yes<br>2 No
127	[lap2]	Laparotomy (uterine rupture, ectopic pregnancy, acute abdomen, intestinal obstruction, perforation, injuries)	radio (Matrix)<br>1 Yes<br>2 No
128	[mc2]	Male circumcision	radio (Matrix)<br>1 Yes<br>2 No
129	[ns2]	Neonatal surgery: abdominal wall defect, colostomy imperforate anus, intussusceptions	radio (Matrix)<br>1 Yes<br>2 No
130	[clr2]	Cleft lip repair	radio (Matrix)<br>1 Yes<br>2 No
131	[clbft2]	Clubfoot repair	radio (Matrix)<br>1 Yes<br>2 No
132	[sg2]	Contracture release/skin grafting	radio (Matrix)<br>1 Yes<br>2 No
133	[clfrac2]	Closed treatment of fracture	radio (Matrix)<br>1 Yes<br>2 No
134	[opfrc2]	Open treatment of fracture	radio (Matrix)<br>1 Yes<br>2 No
135	[jntdis2]	Joint dislocation treatment	radio (Matrix)<br>1 Yes<br>2 No
136	[ost2]	Drainage of osteomyelitis/septic arthritis	radio (Matrix)<br>1 Yes<br>2 No
137	[amp2]	Amputation	radio (Matrix)<br>1 Yes<br>2 No

138	[csurg2]	Cataract surgery	radio (Matrix)<br>1 Yes<br>2 No
139	[rab2]	Regional anaesthesia blocks	radio (Matrix)<br>1 Yes<br>2 No
140	[sa2]	Spinal anaesthesia	radio (Matrix)<br>1 Yes<br>2 No
141	[ka2]	Ketamine intravenous anaesthesia	radio (Matrix)<br>1 Yes<br>2 No
142	[ga2]	General anaesthesia inhalational	radio (Matrix)<br>1 Yes<br>2 No
143	[refer_y]<br>Show the field ONLY if:<br>[resus2] = '1'	If you refer is it due to: (select all that apply)	checkbox<br>1 refer_y___1 Lack of skills<br>2 refer_y___2 Non functioning equipment<br>3 refer_y___3 Lack of supplies/drugs
144	[refer_y_2]<br>Show the field ONLY if:<br>[ct_air2] = '1'	If you refer is it due to: (select all that apply)	checkbox<br>1 refer_y_2___1 Lack of skills<br>2 refer_y_2___2 Non functioning equipment<br>3 refer_y_2___3 Lack of supplies/drugs
145	[refer_y_3]<br>Show the field ONLY if:<br>[cti2] = '1'	If you refer is it due to: (select all that apply)	checkbox<br>1 refer_y_3___1 Lack of skills<br>2 refer_y_3___2 Non functioning equipment<br>3 refer_y_3___3 Lack of supplies/drugs
146	[refer_y_4]<br>Show the field ONLY if:<br>[fb2] = '1'	If you refer is it due to: (select all that apply)	checkbox<br>1 refer_y_4___1 Lack of skills<br>2 refer_y_4___2 Non functioning equipment<br>3 refer_y_4___3 Lack of supplies/drugs
147	[refer_y_5]<br>Show the field ONLY if:<br>[abm2] = '1'	If you refer is it due to: (select all that apply)	checkbox<br>1 refer_y_5___1 Lack of skills<br>2 refer_y_5___2 Non functioning equipment<br>3 refer_y_5___3 Lack of supplies/drugs
148	[refer_y_6]<br>Show the field ONLY if:<br>[id2] = '1'	If you refer is it due to: (select all that apply)	checkbox<br>1 refer_y_6___1 Lack of skills<br>2 refer_y_6___2 Non functioning equipment<br>3 refer_y_6___3 Lack of supplies/drugs
149	[refer_y_7]<br>Show the field ONLY if:<br>[sut2] = '1'	If you refer is it due to: (select all that apply)	checkbox<br>1 refer_y_7___1 Lack of skills<br>2 refer_y_7___2 Non functioning equipment<br>3 refer_y_7___3 Lack of supplies/drugs
150	[refer_y_8]<br>Show the field ONLY if:<br>[wd2] = '1'	If you refer is it due to: (select all that apply)	checkbox<br>1 refer_y_8___1 Lack of skills<br>2 refer_y_8___2 Non functioning equipment<br>3 refer_y_8___3 Lack of supplies/drugs
151	[refer_y_9]<br>Show the field ONLY if:<br>[cs2] = '1'	If you refer is it due to: (select all that apply)	checkbox<br>1 refer_y_9___1 Lack of skills<br>2 refer_y_9___2 Non functioning equipment<br>3 refer_y_9___3 Lack of supplies/drugs

152	[refer_y_10] Show the field ONLY if: [dac2] = '1'	If you refer is it due to: (select all that apply)	checkbox<br>1 refer_y_10___1 Lack of skills<br>2 refer_y_10___2 Non functioning equipment<br>3 refer_y_10___3 Lack of supplies/drugs
153	[refer_y_11] Show the field ONLY if: [ofr2] = '1'	If you refer is it due to: (select all that apply)	checkbox<br>1 refer_y_11___1 Lack of skills<br>2 refer_y_11___2 Non functioning equipment<br>3 refer_y_11___3 Lack of supplies/drugs
154	[refer_y_12] Show the field ONLY if: [tlv2] = '1'	If you refer is it due to: (select all that apply)	checkbox<br>1 refer_y_12___1 Lack of skills<br>2 refer_y_12___2 Non functioning equipment<br>3 refer_y_12___3 Lack of supplies/drugs
155	[refer_y_13] Show the field ONLY if: [bx2] = '1'	If you refer is it due to: (select all that apply)	checkbox<br>1 refer_y_13___1 Lack of skills<br>2 refer_y_13___2 Non functioning equipment<br>3 refer_y_13___3 Lack of supplies/drugs
156	[refer_y_14] Show the field ONLY if: [app2] = '1'	If you refer is it due to: (select all that apply)	checkbox<br>1 refer_y_14___1 Lack of skills<br>2 refer_y_14___2 Non functioning equipment<br>3 refer_y_14___3 Lack of supplies/drugs
157	[refer_y_15] Show the field ONLY if: [hr2] = '1'	If you refer is it due to: (select all that apply)	checkbox<br>1 refer_y_15___1 Lack of skills<br>2 refer_y_15___2 Non functioning equipment<br>3 refer_y_15___3 Lack of supplies/drugs
158	[refer_y_16] Show the field ONLY if: [hyd2] = '1'	If you refer is it due to: (select all that apply)	checkbox<br>1 refer_y_16___1 Lack of skills<br>2 refer_y_16___2 Non functioning equipment<br>3 refer_y_16___3 Lack of supplies/drugs
159	[refer_y_17] Show the field ONLY if: [cyst2] = '1'	If you refer is it due to: (select all that apply)	checkbox<br>1 refer_y_17___1 Lack of skills<br>2 refer_y_17___2 Non functioning equipment<br>3 refer_y_17___3 Lack of supplies/drugs
160	[refer_y_18] Show the field ONLY if: [usd2] = '1'	If you refer is it due to: (select all that apply)	checkbox<br>1 refer_y_18___1 Lack of skills<br>2 refer_y_18___2 Non functioning equipment<br>3 refer_y_18___3 Lack of supplies/drugs
161	[refer_y_19] Show the field ONLY if: [lap2] = '1'	If you refer is it due to: (select all that apply)	checkbox<br>1 refer_y_19___1 Lack of skills<br>2 refer_y_19___2 Non functioning equipment<br>3 refer_y_19___3 Lack of supplies/drugs
162	[refer_y_20] Show the field ONLY if: [mc2] = '1'	If you refer is it due to: (select all that apply)	checkbox<br>1 refer_y_20___1 Lack of skills<br>2 refer_y_20___2 Non functioning equipment<br>3 refer_y_20___3 Lack of supplies/drugs
163	[refer_y_21] Show the field ONLY if: [ns2] = '1'	If you refer is it due to: (select all that apply)	checkbox<br>1 refer_y_21___1 Lack of skills<br>2 refer_y_21___2 Non functioning equipment<br>3 refer_y_21___3 Lack of supplies/drugs
164	[refer_y_22] Show the field ONLY if: [clr2] = '1'	If you refer is it due to: (select all that apply)	checkbox<br>1 refer_y_22___1 Lack of skills<br>2 refer_y_22___2 Non functioning equipment<br>3 refer_y_22___3 Lack of supplies/drugs

165	[refer_y_23]<br>Show the field ONLY if:<br>[clbft2] = '1'	If you refer is it due to: (select all that apply)	checkbox<br>1 refer_y_23___1 Lack of skills<br>2 refer_y_23___2 Non functioning equipment<br>3 refer_y_23___3 Lack of supplies/drugs
166	[refer_y_24]<br>Show the field ONLY if:<br>[sg2] = '1'	If you refer is it due to: (select all that apply)	checkbox<br>1 refer_y_24___1 Lack of skills<br>2 refer_y_24___2 Non functioning equipment<br>3 refer_y_24___3 Lack of supplies/drugs
167	[refer_y_25]<br>Show the field ONLY if:<br>[clfrac2] = '1'	If you refer is it due to: (select all that apply)	checkbox<br>1 refer_y_25___1 Lack of skills<br>2 refer_y_25___2 Non functioning equipment<br>3 refer_y_25___3 Lack of supplies/drugs
168	[refer_y_26]<br>Show the field ONLY if:<br>[opfrc2] = '1'	If you refer is it due to: (select all that apply)	checkbox<br>1 refer_y_26___1 Lack of skills<br>2 refer_y_26___2 Non functioning equipment<br>3 refer_y_26___3 Lack of supplies/drugs
169	[refer_y_27]<br>Show the field ONLY if:<br>[jntdis2] = '1'	If you refer is it due to: (select all that apply)	checkbox<br>1 refer_y_27___1 Lack of skills<br>2 refer_y_27___2 Non functioning equipment<br>3 refer_y_27___3 Lack of supplies/drugs
170	[refer_y_28]<br>Show the field ONLY if:<br>[ost2] = '1'	If you refer is it due to: (select all that apply)	checkbox<br>1 refer_y_28___1 Lack of skills<br>2 refer_y_28___2 Non functioning equipment<br>3 refer_y_28___3 Lack of supplies/drugs
171	[refer_y_29]<br>Show the field ONLY if:<br>[amp2] = '1'	If you refer is it due to: (select all that apply)	checkbox<br>1 refer_y_29___1 Lack of skills<br>2 refer_y_29___2 Non functioning equipment<br>3 refer_y_29___3 Lack of supplies/drugs
172	[refer_y_30]<br>Show the field ONLY if:<br>[csurg2] = '1'	If you refer is it due to: (select all that apply)	checkbox<br>1 refer_y_30___1 Lack of skills<br>2 refer_y_30___2 Non functioning equipment<br>3 refer_y_30___3 Lack of supplies/drugs
173	[refer_y_31]<br>Show the field ONLY if:<br>[rab2] = '1'	If you refer is it due to: (select all that apply)	checkbox<br>1 refer_y_31___1 Lack of skills<br>2 refer_y_31___2 Non functioning equipment<br>3 refer_y_31___3 Lack of supplies/drugs
174	[refer_y_32]<br>Show the field ONLY if:<br>[sa2] = '1'	If you refer is it due to: (select all that apply)	checkbox<br>1 refer_y_32___1 Lack of skills<br>2 refer_y_32___2 Non functioning equipment<br>3 refer_y_32___3 Lack of supplies/drugs
175	[refer_y_33]<br>Show the field ONLY if:<br>[ka2] = '1'	If you refer is it due to: (select all that apply)	checkbox<br>1 refer_y_33___1 Lack of skills<br>2 refer_y_33___2 Non functioning equipment<br>3 refer_y_33___3 Lack of supplies/drugs
176	[refer_y_34]<br>Show the field ONLY if:<br>[ga2] = '1'	If you refer is it due to: (select all that apply)	checkbox<br>1 refer_y_34___1 Lack of skills<br>2 refer_y_34___2 Non functioning equipment<br>3 refer_y_34___3 Lack of supplies/drugs
177	[resus_bv_ad]	Resuscitator bag valve & mask (adult)	radio (Matrix)<br>1 Absent<br>2 Available with frequent shortages or difficulties<br>3 Fully available for all patients all the time

178	[resus_bv_ped]	Resuscitator bag valve & mask (pediatric)	radio (Matrix)<br>1 Absent<br>2 Available with frequent shortages or difficulties<br>3 Fully available for all patients all the time
179	[steth]	Stethoscope	radio (Matrix)<br>1 Absent<br>2 Available with frequent shortages or difficulties<br>3 Fully available for all patients all the time
180	[sucpum]	Suction pump (manual or electric) with catheter	radio (Matrix)<br>1 Absent<br>2 Available with frequent shortages or difficulties<br>3 Fully available for all patients all the time
181	[bp]	Blood pressure measuring equipment	radio (Matrix)<br>1 Absent<br>2 Available with frequent shortages or difficulties<br>3 Fully available for all patients all the time
182	[ther]	Thermometer	radio (Matrix)<br>1 Absent<br>2 Available with frequent shortages or difficulties<br>3 Fully available for all patients all the time
183	[scalp]	Scalpel with blades	radio (Matrix)<br>1 Absent<br>2 Available with frequent shortages or difficulties<br>3 Fully available for all patients all the time
184	[ret]	Retractor	radio (Matrix)<br>1 Absent<br>2 Available with frequent shortages or difficulties<br>3 Fully available for all patients all the time
185	[sci]	Scissors	radio (Matrix)<br>1 Absent<br>2 Available with frequent shortages or difficulties<br>3 Fully available for all patients all the time
186	[oro_ad]	Oropharyngeal airway (adult size)	radio (Matrix)<br>1 Absent<br>2 Available with frequent shortages or difficulties<br>3 Fully available for all patients all the time
187	[oro_ped]	Oropharyngeal airway (pediatric size)	radio (Matrix)<br>1 Absent<br>2 Available with frequent shortages or difficulties<br>3 Fully available for all patients all the time
188	[for_art]	Forceps, artery	radio (Matrix)<br>1 Absent<br>2 Available with frequent shortages or difficulties<br>3 Fully available for all patients all the time
189	[glov_str]	Gloves (sterile)	radio (Matrix)<br>1 Absent<br>2 Available with frequent shortages or difficulties<br>3 Fully available for all patients all the time
190	[glov_ex]	Gloves (examination)	radio (Matrix)<br>1 Absent<br>2 Available with frequent shortages or difficulties<br>3 Fully available for all patients all the time

191	[nh]	Needle holder	radio (Matrix)<br>1 Absent<br>2 Available with frequent shortages or difficulties<br>3 Fully available for all patients all the time
192	[vag_spec]	Vaginal speculum	radio (Matrix)<br>1 Absent<br>2 Available with frequent shortages or difficulties<br>3 Fully available for all patients all the time
193	[ng]	Nasogastric tubes	radio (Matrix)<br>1 Absent<br>2 Available with frequent shortages or difficulties<br>3 Fully available for all patients all the time
194	[light]	Light source (lamp & flash light)	radio (Matrix)<br>1 Absent<br>2 Available with frequent shortages or difficulties<br>3 Fully available for all patients all the time
195	[iv_set]	Intravenous fluid infusion set	radio (Matrix)<br>1 Absent<br>2 Available with frequent shortages or difficulties<br>3 Fully available for all patients all the time
196	[iv_can]	Intravenous cannulas/scalp vein infusion set	radio (Matrix)<br>1 Absent<br>2 Available with frequent shortages or difficulties<br>3 Fully available for all patients all the time
197	[syr]	Syringes with needles (disposable)	radio (Matrix)<br>1 Absent<br>2 Available with frequent shortages or difficulties<br>3 Fully available for all patients all the time
198	[desp]	Sharps disposal container	radio (Matrix)<br>1 Absent<br>2 Available with frequent shortages or difficulties<br>3 Fully available for all patients all the time
199	[tourn]	Tourniquet	radio (Matrix)<br>1 Absent<br>2 Available with frequent shortages or difficulties<br>3 Fully available for all patients all the time
200	[ns]	Needles & sutures	radio (Matrix)<br>1 Absent<br>2 Available with frequent shortages or difficulties<br>3 Fully available for all patients all the time
201	[splint]	Splints for arm, leg	radio (Matrix)<br>1 Absent<br>2 Available with frequent shortages or difficulties<br>3 Fully available for all patients all the time
202	[cath]	Urinary catheters (Foleys disposable)	radio (Matrix)<br>1 Absent<br>2 Available with frequent shortages or difficulties<br>3 Fully available for all patients all the time
203	[disp]	Waste disposal container	radio (Matrix)<br>1 Absent<br>2 Available with frequent shortages or difficulties<br>3 Fully available for all patients all the time

204	[fm]	Face masks	radio (Matrix)<br>1 Absent<br>2 Available with frequent shortages or difficulties<br>3 Fully available for all patients all the time
205	[ep]	Eye protection	radio (Matrix)<br>1 Absent<br>2 Available with frequent shortages or difficulties<br>3 Fully available for all patients all the time
206	[gwn]	Protective gowns/aprons	radio (Matrix)<br>1 Absent<br>2 Available with frequent shortages or difficulties<br>3 Fully available for all patients all the time
207	[soap]	Soap	radio (Matrix)<br>1 Absent<br>2 Available with frequent shortages or difficulties<br>3 Fully available for all patients all the time
208	[mf_ad]	Magill forceps (adult)	radio (Matrix)<br>1 Absent<br>2 Available with frequent shortages or difficulties<br>3 Fully available for all patients all the time
209	[mf_ped]	Magill forceps (pediatric)	radio (Matrix)<br>1 Absent<br>2 Available with frequent shortages or difficulties<br>3 Fully available for all patients all the time
210	[et_ad]	Endotracheal tubes (adult)	radio (Matrix)<br>1 Absent<br>2 Available with frequent shortages or difficulties<br>3 Fully available for all patients all the time
211	[et_ped]	Endotracheal tubes (pediatric)	radio (Matrix)<br>1 Absent<br>2 Available with frequent shortages or difficulties<br>3 Fully available for all patients all the time
212	[iv_bg]	IV infusor bags	radio (Matrix)<br>1 Absent<br>2 Available with frequent shortages or difficulties<br>3 Fully available for all patients all the time
213	[chst_tub_equip]	Chest tubes insertion equipment	radio (Matrix)<br>1 Absent<br>2 Available with frequent shortages or difficulties<br>3 Fully available for all patients all the time
214	[lmb_ad]	Laryngoscope Macintosh blades with bulbs & batteries (adult)	radio (Matrix)<br>1 Absent<br>2 Available with frequent shortages or difficulties<br>3 Fully available for all patients all the time
215	[lmb_ped]	Laryngoscope Macintosh blades with bulbs & batteries (pediatric)	radio (Matrix)<br>1 Absent<br>2 Available with frequent shortages or difficulties<br>3 Fully available for all patients all the time
216	[cric_set]	Cricothyroidotomy set	radio (Matrix)<br>1 Absent<br>2 Available with frequent shortages or difficulties<br>3 Fully available for all patients all the time

217	[ster]	Sterilizer	radio (Matrix)<br>1 Absent<br>2 Available with frequent shortages or difficulties<br>3 Fully available for all patients all the time
218	[world_health_organization_tool_for_situational_ana_complete]	Section Header: *Form Status*<br>Complete?	dropdown<br>0 Incomplete<br>1 Unverified<br>2 Complete
Instrument: **Data validation stage 1** (data_validation_stage_1)			
219	[txt1]	This form is to be completed by investigators who collect/enter data.	descriptive
220	[pi_valid_1]	Methods used for identifying patients operated on in local centre/unit/hospital (select all that apply)	checkbox<br>1 pi_valid_1___1 Theatre logbook/system review<br>2 pi_valid_1___2 Planned operating lists<br>3 pi_valid_1___3 Ward lists<br>4 pi_valid_1___4 Handover lists<br>5 pi_valid_1___5 Staff memory<br>6 pi_valid_1___6 Other
221	[ps_pi_valid_1]<br>Show the field ONLY if:<br>[pi_valid_1(6)] = '1'	Please state	text
222	[fu_dat_val1]	Methods used to follow up patients during a 30 day time period post surgery (select all that apply)	checkbox<br>1 fu_dat_val1___1 Clinic review<br>2 fu_dat_val1___2 Community/home review<br>3 fu_dat_val1___3 No contact after discharge<br>4 fu_dat_val1___4 Still inpatient<br>5 fu_dat_val1___5 Telephone review<br>6 fu_dat_val1___6 Reviewing hospital records for readmissions<br>7 fu_dat_val1___7 Reviewing emergency department lists for readmissions<br>8 fu_dat_val1___8 Contacting general/family practitioners to enquire about complications managed in primary care<br>9 fu_dat_val1___9 Other
223	[ps_fu_dat_val1]<br>Show the field ONLY if:<br>[fu_dat_val1(9)] = '1'	Please state	text
224	[data_validation_stage_1_complete]	Section Header: *Form Status*<br>Complete?	dropdown<br>0 Incomplete<br>1 Unverified<br>2 Complete
Instrument: **Data validation stage 2** (data_validation_stage_2)			
225	[txt2]	This form is to be completed by independent investigators from the local site not involved in data collection/entry	descriptive
226	[case_numb_val2]	Total number of cases identified as eligible for data entry in the 30 day data collection period	text (number)
227	[age_valid2]	Patient age data available	dropdown<br>1 All cases (100%)<br>2 Most cases (80-99%)<br>3 Some cases (50-79%)<br>4 Not enough cases (30-49%)<br>5 Hardly any cases (0-29%)

228	[bmi_valid2]	Body mass index data available	dropdown<br>1 All cases (100%)<br>2 Most cases (80-99%)<br>3 Some cases (50-79%)<br>4 Not enough cases (30-49%)<br>5 Hardly any cases (0-29%)
229	[ecog_valid2]	ECOG performance status data available	dropdown<br>1 All cases (100%)<br>2 Most cases (80-99%)<br>3 Some cases (50-79%)<br>4 Not enough cases (30-49%)<br>5 Hardly any cases (0-29%)
230	[urg_valid2]	Surgical urgency data available	dropdown<br>1 All cases (100%)<br>2 Most cases (80-99%)<br>3 Some cases (50-79%)<br>4 Not enough cases (30-49%)<br>5 Hardly any cases (0-29%)
231	[anaes_valid]	Mode of anaesthesia data available	dropdown<br>1 All cases (100%)<br>2 Most cases (80-99%)<br>3 Some cases (50-79%)<br>4 Not enough cases (30-49%)<br>5 Hardly any cases (0-29%)
232	[surgmod_valid2]	Operative modality/approach data available	dropdown<br>1 All cases (100%)<br>2 Most cases (80-99%)<br>3 Some cases (50-79%)<br>4 Not enough cases (30-49%)<br>5 Hardly any cases (0-29%)
233	[surg_int_valid2]	Surgical aim/intent data available	dropdown<br>1 All cases (100%)<br>2 Most cases (80-99%)<br>3 Some cases (50-79%)<br>4 Not enough cases (30-49%)<br>5 Hardly any cases (0-29%)
234	[resect_valid2]	Resectability data available	dropdown<br>1 All cases (100%)<br>2 Most cases (80-99%)<br>3 Some cases (50-79%)<br>4 Not enough cases (30-49%)<br>5 Hardly any cases (0-29%)
235	[iop_valid2]	Intra-operative morbidity data available	dropdown<br>1 All cases (100%)<br>2 Most cases (80-99%)<br>3 Some cases (50-79%)<br>4 Not enough cases (30-49%)<br>5 Hardly any cases (0-29%)

236	[morb_valid2]	30 day post-operative morbidity data available	dropdown<br>1 All cases (100%)<br>2 Most cases (80-99%)<br>3 Some cases (50-79%)<br>4 Not enough cases (30-49%)<br>5 Hardly any cases (0-29%)
237	[mort_valid2]	30 day post-operative mortality data available	dropdown<br>1 All cases (100%)<br>2 Most cases (80-99%)<br>3 Some cases (50-79%)<br>4 Not enough cases (30-49%)<br>5 Hardly any cases (0-29%)
238	[data_validation_stage_2_complete]	Section Header: *Form Status*<br>Complete?	dropdown<br>0 Incomplete<br>1 Unverified<br>2 Complete
Instrument: **Education and training** (education_and_training)			
239	[go_training]	Do you have a formal national gynaecological oncology training programme available in your country?	yesno<br>1 Yes<br>0 No
240	[yrs_train]<br>Show the field ONLY if:<br>[go_training] = '1'	How long is the training programme in years?	text (number)
241	[accredit_yn]	Is your hospital accredited for performing gynaecological oncology surgery by national/international organisations?	yesno<br>1 Yes<br>0 No
242	[accred_org_name]<br>Show the field ONLY if:<br>[accredit_yn] = '1'	Name of accrediting organisation (select all that apply)	checkbox<br>1 accred_org_name___1 ESGO (European Society of Gynaecological Oncology)<br>2 accred_org_name___2 SGO (Society of Gynecologic Oncology)<br>3 accred_org_name___3 RCOG (Royal College of Obstetricians and Gynaecologists)<br>5 accred_org_name___5 IGCS (International Gynecologic Cancer Society)<br>4 accred_org_name___4 Other
243	[ps]<br>Show the field ONLY if:<br>[accred_org_name(4)] = '1'	Please state	text
244	[go_surg_train]	Have all surgeons performing gynaecological oncology surgery in your centre/unit undergone formal training as part of a national training programme or other formal local training?	yesno<br>1 Yes<br>0 No
245	[education_and_training_complete]	Section Header: *Form Status*<br>Complete?	dropdown<br>0 Incomplete<br>1 Unverified<br>2 Complete
Instrument: **Ovarian cancer (initial presentation)** (ovarian_cancer_initial_presentation)			
246	[dat_oc]	Date of primary diagnosis	text (date_dmy)
247	[age]	Section Header: *DEMOGRAPHICS*<br>Age of patient	text (number)

248	[eth_oc]	Ethnicity	dropdown<br>1 White (British, Australian, North American, European, other white)<br>2 Asian (South East Asian, Far East Asian, other Asian)<br>3 Black (African, Caribbean, other Black)<br>4 Middle Eastern (Arab, North African, Turkish)<br>5 Mixed (multiple ethnic backgrounds)<br>6 Other
249	[ps_ethn]<br>Show the field ONLY if:<br>[eth_oc] = '6'	Please state	text
250	[bmi_oc]	Body mass index (kg/m2)	text (number)
251	[asa_oc]	Section Header: *PRE-OPERATIVE*<br>American Society of Anesthesiologists (ASA) physical status	dropdown<br>1 ASA 1: A normal healthy patient. Example: Fit, non-obese (BMI under 30), a nonsmoking patient with good exercise tolerance.<br>2 ASA 2: A patient with a mild systemic disease. Example: Patient with no functional limitations and a well-controlled disease (e.g., treated hypertension, obesity with BMI under 35, frequent social drinker or is a cigarette smoker).<br>3 ASA 3: A patient with a severe systemic disease that is not life-threatening. Example: Patient with some functional limitation as a result of disease (e.g., poorly treated hypertension or diabetes, morbid obesity, chronic renal failure, a bronchospastic disease with intermittent exacerbation, stable angina, implanted pacemaker).<br>4 ASA 4: A patient with a severe systemic disease that is a constant threat to life. Example: Patient with functional limitation from severe, life-threatening disease (e.g., unstable angina, poorly controlled COPD, symptomatic CHF, recent (less than three months ago) myocardial infarction or stroke.<br>5 ASA 5: A moribund patient who is not expected to survive without the operation. The patient is not expected to survive beyond the next 24 hours without surgery. Examples: ruptured abdominal aortic aneurysm, massive trauma, and extensive intracranial hemorrhage with mass effect.<br>6 ASA 6: A brain-dead patient whose organs are being removed with the intention of transplanting them into another patient.
252	[ecog_oc]	ECOG (Eastern Cooperative Oncology Group) performance status	dropdown<br>1 Grade 0 - Fully active, able to carry on all pre-disease performance without restriction<br>2 Grade 1 - Restricted in physically strenuous activity but ambulatory and able to carry out work of a light or sedentary nature, e.g., light house work, office work<br>3 Grade 2 - Ambulatory and capable of all selfcare but unable to carry out any work activities; up and about more than 50% of waking hours<br>4 Grade 3 - Capable of only limited selfcare; confined to bed or chair more than 50% of waking hours<br>5 Grade 4 - Completely disabled; cannot carry on any selfcare; totally confined to bed or chair<br>6 Grade 5 - Dead

253	[comorb]	Co-morbidities (select all that apply)	checkbox<br>1 comorb___1 Cardiovascular<br>2 comorb___2 Respiratory<br>3 comorb___3 Urogenital<br>4 comorb___4 Endocrine<br>5 comorb___5 Neurological<br>6 comorb___6 Haematological<br>7 comorb___7 Psychiatric<br>8 comorb___8 Gastroenterology<br>9 comorb___9 Rheumatology and musculoskeletal<br>10 comorb___10 Oncology<br>11 comorb___11 None
254	[cvd_oc]<br>Show the field ONLY if:<br>[comorb(1)] = '1'	Select all that apply	checkbox<br>1 cvd_oc___1 Hypertension<br>2 cvd_oc___2 Coronary heart disease / ischaemic heart disease<br>3 cvd_oc___3 Myocardial infarction<br>4 cvd_oc___4 Heart failure<br>5 cvd_oc___5 Arrhythmia<br>6 cvd_oc___6 Heart valve disease<br>7 cvd_oc___7 Venous insufficiency<br>8 cvd_oc___8 Peripheral vascular disease<br>9 cvd_oc___9 Stroke<br>10 cvd_oc___10 Transient ischaemic attack<br>11 cvd_oc___11 Other
255	[comorb_resp_oc]<br>Show the field ONLY if:<br>[comorb(2)] = '1'	Select all that apply	checkbox<br>1 comorb_resp_oc___1 Asthma<br>2 comorb_resp_oc___2 Emphysema (COPD)<br>3 comorb_resp_oc___3 Chronic bronchitis (COPD)<br>4 comorb_resp_oc___4 Pulmonary fibrosis<br>5 comorb_resp_oc___5 Sleep apnoea<br>6 comorb_resp_oc___6 Bronchiectasis<br>7 comorb_resp_oc___7 Cystic fibrosis<br>8 comorb_resp_oc___8 Tuberculosis<br>9 comorb_resp_oc___9 Pneumonia<br>10 comorb_resp_oc___10 Other
256	[comorb_urog_oc]<br>Show the field ONLY if:<br>[comorb(3)] = '1'	Select all that apply	checkbox<br>1 comorb_urog_oc___1 Chronic kidney disease (not requiring haemodialysis)<br>2 comorb_urog_oc___2 Chronic kidney disease (requiring haemodialysis)<br>3 comorb_urog_oc___3 Polycystic kidney disease<br>4 comorb_urog_oc___4 Glomerulonephropathy<br>5 comorb_urog_oc___5 Glomerulonephritis<br>6 comorb_urog_oc___6 Urinary tract infection<br>7 comorb_urog_oc___7 Other
257	[comorb_endo_oc]<br>Show the field ONLY if:<br>[comorb(4)] = '1'	Select all that apply	checkbox<br>1 comorb_endo_oc___1 Type 1 diabetes mellitus<br>2 comorb_endo_oc___2 Type 2 diabetes mellitus<br>3 comorb_endo_oc___3 Hyperthyroidism<br>4 comorb_endo_oc___4 Hypothyroidism<br>5 comorb_endo_oc___5 Other

258	[comorb_neuro_oc]<br>Show the field ONLY if:<br>[comorb(5)] = '1'	Select all that apply	checkbox<br>1 comorb_neuro_oc___1 Parkinsons disease<br>2 comorb_neuro_oc___2 Myasthenia gravis<br>3 comorb_neuro_oc___3 Dementia<br>4 comorb_neuro_oc___4 Epilepsy<br>5 comorb_neuro_oc___5 Other
259	[comorb_haem_oc]<br>Show the field ONLY if:<br>[comorb(6)] = '1'	Select all that apply	checkbox<br>1 comorb_haem_oc___1 Iron deficiency anaemia<br>2 comorb_haem_oc___2 Folate deficience anaemia<br>3 comorb_haem_oc___3 Vitamin B12 deficiency anaemia<br>4 comorb_haem_oc___4 Pernicious anaemia<br>5 comorb_haem_oc___5 Haemolytic anaemia<br>6 comorb_haem_oc___6 Aplastic anaemia<br>7 comorb_haem_oc___7 Thalassemia<br>8 comorb_haem_oc___8 Sickle cell disease<br>9 comorb_haem_oc___9 Disseminated intravascular coagulation (DIC)<br>10 comorb_haem_oc___10 Factor V Leiden<br>11 comorb_haem_oc___11 Thrombocytosis<br>12 comorb_haem_oc___12 Idiopathic thrombocytopenic purpura (ITP)<br>13 comorb_haem_oc___13 Haemophilia<br>14 comorb_haem_oc___14 Von Willebrand disease<br>15 comorb_haem_oc___15 Antiphospholipid syndrome<br>16 comorb_haem_oc___16 Thrombocytopenia<br>17 comorb_haem_oc___17 Thrombotic thrombocytopenic purpura<br>18 comorb_haem_oc___18 Deep vein thrombosis<br>19 comorb_haem_oc___19 Pulmonary embolism<br>20 comorb_haem_oc___20 Other
260	[comorb_pscy_oc]<br>Show the field ONLY if:<br>[comorb(7)] = '1'	Select all that apply	checkbox<br>1 comorb_pscy_oc___1 Anxiety<br>2 comorb_pscy_oc___2 Depression<br>3 comorb_pscy_oc___3 Learning difficulties<br>4 comorb_pscy_oc___4 Personality disorder<br>5 comorb_pscy_oc___5 Schizophrenia<br>6 comorb_pscy_oc___6 Bipolar disorder<br>7 comorb_pscy_oc___7 Autism<br>8 comorb_pscy_oc___8 Other

261	[comorb_gi_oc]<br>Show the field ONLY if:<br>[comorb(8)] = '1'	Select all that apply	checkbox<br>1 comorb_gi_oc___1 Crohn's disease<br>2 comorb_gi_oc___2 Ulcerative colitis<br>3 comorb_gi_oc___3 Irritable bowel syndrome<br>4 comorb_gi_oc___4 Celiac Disease<br>5 comorb_gi_oc___5 Peptic ulcer disease<br>6 comorb_gi_oc___6 Gallstones<br>7 comorb_gi_oc___7 Pancreatitis<br>8 comorb_gi_oc___8 Diverticulitis<br>9 comorb_gi_oc___9 Non-Alcoholic Fatty Liver Disease<br>10 comorb_gi_oc___10 Alcoholic fatty liver disease<br>11 comorb_gi_oc___11 Viral hepatitis<br>12 comorb_gi_oc___12 Liver failure<br>13 comorb_gi_oc___13 Other
262	[comorb_msk_oc]<br>Show the field ONLY if:<br>[comorb(9)] = '1'	Select all that apply	checkbox<br>1 comorb_msk_oc___1 Osteoarthritis<br>2 comorb_msk_oc___2 Rheumatoid arthritis<br>3 comorb_msk_oc___3 Osteoporosis<br>4 comorb_msk_oc___4 Lupus<br>5 comorb_msk_oc___5 Spondyloarthropathies<br>6 comorb_msk_oc___6 Sjogren's syndrome<br>7 comorb_msk_oc___7 Gout<br>8 comorb_msk_oc___8 Other
263	[comorb_onc_oc]<br>Show the field ONLY if:<br>[comorb(10)] = '1'	Select all that apply	checkbox<br>1 comorb_onc_oc___1 Breast cancer<br>2 comorb_onc_oc___2 Lung cancer<br>3 comorb_onc_oc___3 Hepatobiliary cancer<br>4 comorb_onc_oc___4 Brain cancer<br>5 comorb_onc_oc___5 Leukemia<br>6 comorb_onc_oc___6 Lymphoma<br>7 comorb_onc_oc___7 Osteosarcoma<br>8 comorb_onc_oc___8 Other
264	[lap_oc]	Previous laparoscopic abdominal surgery	yesno<br>1 Yes<br>0 No
265	[laparot_oc]	Previous laparotomy	yesno<br>1 Yes<br>0 No
266	[mdt_oc]	Discussed at multidisciplinary team meeting/tumour board?	yesno<br>1 Yes<br>0 No

267	[mdt_tm_oc]<br>Show the field ONLY if:<br>[mdt_oc] = '1'	Team members making up multidisciplinary team/tumour board (select all that apply)	checkbox<br>1 mdt_tm_oc___1 Certified gynaecological oncology surgeon<br>2 mdt_tm_oc___2 Trained surgeon dedicated to the management of gynaecologic cancer (accounting for more than 50% of his/her practice) or having completed an ESGO-accredited fellowship<br>3 mdt_tm_oc___3 Radiologist<br>4 mdt_tm_oc___4 Pathologist<br>5 mdt_tm_oc___5 Physician certified to deliver chemotherapy (a gynaecologic oncologist in countries where the subspecialty is structured and/or a medical oncologist with special interest in gynaecologic oncology)<br>6 mdt_tm_oc___6 Clinical oncologist (trained to administer radiotherapy)
268	[mdt_oc_no]<br>Show the field ONLY if:<br>[mdt_oc] = '0'	Reason not discussed (select all that apply)	checkbox<br>1 mdt_oc_no___1 No multidisciplinary team meeting/tumour board<br>2 mdt_oc_no___2 Other
269	[ps_mdt_oc_no]<br>Show the field ONLY if:<br>[mdt_oc_no(2)] = '1'	Please state	text
270	[bx_oc]	Diagnostic biopsy performed?	yesno<br>1 Yes<br>0 No
271	[bx_no_reas]<br>Show the field ONLY if:<br>[bx_oc] = '0'	Reason not performed (select all that apply)	checkbox<br>1 bx_no_reas___1 Biopsy not technically feasible<br>2 bx_no_reas___2 Patient refused<br>3 bx_no_reas___3 Patient unable to afford<br>4 bx_no_reas___4 No facilities to perform biopsy<br>5 bx_no_reas___5 Other
272	[ps_bx_no_reas]<br>Show the field ONLY if:<br>[bx_no_reas(5)] = '1'	Please state	text
273	[tm_oc]	Tumour markers tested (select all that apply)	checkbox<br>1 tm_oc___1 CA125<br>2 tm_oc___2 CA 19-9<br>3 tm_oc___3 CEA<br>4 tm_oc___4 Inhibin A<br>5 tm_oc___5 Inhibin B<br>6 tm_oc___6 AFP<br>7 tm_oc___7 HCG<br>8 tm_oc___8 LDH
274	[ca125_val]<br>Show the field ONLY if:<br>[tm_oc(1)] = '1'	Enter value	text
275	[ca19_9_val]<br>Show the field ONLY if:<br>[tm_oc(2)] = '1'	Enter value	text
276	[cea_val]<br>Show the field ONLY if:<br>[tm_oc(3)] = '1'	Enter value	text

277	[inhibin_val]<br>Show the field ONLY if:<br>[tm_oc(4)] = '1'	Enter value	text
278	[inhibin_val_2]<br>Show the field ONLY if:<br>[tm_oc(5)] = '1'	Enter value	text
279	[afp_val]<br>Show the field ONLY if:<br>[tm_oc(6)] = '1'	Enter value	text
280	[hcg_val]<br>Show the field ONLY if:<br>[tm_oc(7)] = '1'	Enter value	text
281	[ldh_val_1]<br>Show the field ONLY if:<br>[tm_oc(8)] = '1'	Enter value	text
282	[uss]	Ultrasound performed at diagnosis?	yesno<br>1 Yes<br>0 No
283	[uss_oc]<br>Show the field ONLY if:<br>[uss] = '1'	Ultrasound results at diagnosis (select all that apply)	checkbox<br>1 uss_oc___1 B1 Unilocular<br>2 uss_oc___2 B2 Presence of solid components with largest diameter < 7mm<br>3 uss_oc___3 B3 Presence of acoustic shadows<br>4 uss_oc___4 B4 Smooth multilocular tumor with largest diameter < 100 mm<br>5 uss_oc___5 B5 No blood flow (colour score 1)<br>6 uss_oc___6 M1 Irregular solid tumor<br>7 uss_oc___7 M2 Presence of ascites<br>8 uss_oc___8 M3 At least 4 papillary structures<br>9 uss_oc___9 M4 Irregular multilocular-solid tumor with largest diameter >100 mm<br>10 uss_oc___10 M5 Very strong blood flow (colour score 4)<br>11 uss_oc___11 Ascites
284	[uss_nt_reas_oc]<br>Show the field ONLY if:<br>[uss] = '0'	Reason ultrasound not performed at diagnosis (select all that apply)	checkbox<br>1 uss_nt_reas_oc___1 No access to ultrasound machine<br>2 uss_nt_reas_oc___2 Patient unable to afford<br>3 uss_nt_reas_oc___3 Patient refused<br>4 uss_nt_reas_oc___4 Other imaging performed<br>5 uss_nt_reas_oc___5 Other
285	[ps_uss_nt_reas_oc]<br>Show the field ONLY if:<br>[uss_nt_reas_oc(5)] = '1'	Please state	text
286	[rmi]	Risk of malignancy index I score calculated	yesno<br>1 Yes<br>0 No
287	[rmi_val]<br>Show the field ONLY if:<br>[rmi] = '1'	Enter value	text
288	[ct_yn]	Staging computerised tomography (CT) performed at diagnosis?	yesno<br>1 Yes<br>0 No

289	[ct_oc]<br>Show the field ONLY if:<br>[ct_yn] = '1'	Staging computerised tomography (CT) results at diagnosis (select all that apply)	checkbox

1	ct_oc___1	Ascites
2	ct_oc___2	Unilateral ovarian mass < 5cm
3	ct_oc___3	Unilateral ovarian mass 5-10cm
4	ct_oc___4	Unilateral ovarian mass 10-20cm
5	ct_oc___5	Unilateral ovarian mass >20cm
6	ct_oc___6	Bilateral ovarian mass < 5cm
7	ct_oc___7	Bilateral ovarian mass 5-10cm
8	ct_oc___8	Bilateral ovarian mass 10-20cm
9	ct_oc___9	Bilateral ovarian mass >20cm
10	ct_oc___10	Fallopian tube invasion
11	ct_oc___11	Uterus invasion
12	ct_oc___12	Peritoneal deposits in pouch of douglas
13	ct_oc___13	Para rectal nodules
14	ct_oc___14	Sigmoid nodules
15	ct_oc___15	Rectal and sigmoid diffuse serosal invasion
16	ct_oc___16	Rectal and sigmoid diffuse wall invasion
17	ct_oc___17	Rectal and sigmoid diffuse luminal invasion
18	ct_oc___18	Pelvic vascular side wall invasion
19	ct_oc___19	Ureteric invasion
20	ct_oc___20	Bony and muscular pelvic side wall invasion
21	ct_oc___21	Diffuse presacral disease
22	ct_oc___22	Pelvic lymphadenopathy
23	ct_oc___23	Abdominal lymphadenopathy
24	ct_oc___24	Retroperitoneal lymphadenopathy
25	ct_oc___25	High retroperitoneal nodes at or above the coeliac axis
26	ct_oc___26	Diffuse large volume mesenteric disease
27	ct_oc___27	Root of small bowel mesentry disease
53	ct_oc___53	small bowel serosal metastasis
52	ct_oc___52	paracolic gutter metastasis
28	ct_oc___28	Stranding, nodular, diffuse omental cake
29	ct_oc___29	Involvement of transverse mesocolon
30	ct_oc___30	Perisplenic subcapsular disease
31	ct_oc___31	Splenic hilar disease
32	ct_oc___32	Subdiaphragmatic disease
33	ct_oc___33	Gastro-splenic ligament invasion
34	ct_oc___34	Lesser omentum disease
35	ct_oc___35	Gastric and duodenal serosa disease
36	ct_oc___36	Hepatic portal disease
37	ct_oc___37	Lesser sac disease
38	ct_oc___38	Coeliac lymphadenopathy
39	ct_oc___39	Falciform ligament disease
40	ct_oc___40	Morrisons Pouch and gall bladder fossa disease
41	ct_oc___41	Parenchymal liver metastasis or invasive surface implants

			42 ct_oc___42 Peritoneal and serosal disease along the ascending colon<br>43 ct_oc___43 Appendiceal mass<br>44 ct_oc___44 Diaphragm metastasis<br>45 ct_oc___45 Pleural effusion with pleural infiltration<br>46 ct_oc___46 Paracardiac nodes<br>47 ct_oc___47 Axillary nodes<br>48 ct_oc___48 Supraclavicular nodes<br>49 ct_oc___49 Inguinal nodes<br>51 ct_oc___51 Pancreatic invasion<br>50 ct_oc___50 Other
290	[ps_ct_oc]<br>Show the field ONLY if:<br>[ct_oc(50)] = '1'	Please state	text
291	[no_ct]<br>Show the field ONLY if:<br>[ct_yn] = '0'	Reason computerised tomography (CT) not performed at diagnosis (select all that apply)	checkbox<br>1 no_ct___1 No access to CT machine<br>2 no_ct___2 Patient unable to afford<br>3 no_ct___3 Patient refused<br>4 no_ct___4 Other imaging performed<br>5 no_ct___5 Allergic reaction to contrast<br>6 no_ct___6 Chronic kidney disease<br>7 no_ct___7 Other
292	[ps_no_ct]<br>Show the field ONLY if:<br>[no_ct(7)] = '1'	Please state	text
293	[mri_yn]	Magnetic resonance imaging (MRI) performed at diagnosis?	yesno<br>1 Yes<br>0 No

294	[mri_oc]<br>Show the field ONLY if:<br>[mri_yn] = '1'	MRI results at diagnosis (select all that apply)	checkbox

1	mri_oc___1	Ascites
2	mri_oc___2	Unilateral ovarian mass < 5cm
3	mri_oc___3	Unilateral ovarian mass 5-10cm
4	mri_oc___4	Unilateral ovarian mass 10-20cm
5	mri_oc___5	Unilateral ovarian mass >20cm
6	mri_oc___6	Bilateral ovarian mass < 5cm
7	mri_oc___7	Bilateral ovarian mass 5-10cm
8	mri_oc___8	Bilateral ovarian mass 10-20cm
9	mri_oc___9	Bilateral ovarian mass >20cm
10	mri_oc___10	Fallopian tube invasion
11	mri_oc___11	Uterus invasion
12	mri_oc___12	Peritoneal deposits in pouch of douglas
13	mri_oc___13	Para rectal nodules
14	mri_oc___14	Sigmoid nodules
15	mri_oc___15	Rectal and sigmoid diffuse serosal invasion
16	mri_oc___16	Rectal and sigmoid diffuse wall invasion
17	mri_oc___17	Rectal and sigmoid diffuse luminal invasion
18	mri_oc___18	Pelvic vascular side wall invasion
19	mri_oc___19	Ureteric invasion
20	mri_oc___20	Bony and muscular pelvic side wall invasion
21	mri_oc___21	Diffuse presacral disease
22	mri_oc___22	Pelvic lymphadenopathy
23	mri_oc___23	Abdominal lymphadenopathy
24	mri_oc___24	Retroperitoneal lymphadenopathy
25	mri_oc___25	High retroperitoneal nodes at or above the coeliac axis
26	mri_oc___26	Diffuse large volume mesenteric disease
27	mri_oc___27	Root of small bowel mesentry disease
53	mri_oc___53	small bowel serosal metastasis
52	mri_oc___52	paracolic gutter metastasis
28	mri_oc___28	Stranding, nodular, diffuse omental cake
29	mri_oc___29	Involvement of transverse mesocolon
30	mri_oc___30	Perisplenic subcapsular disease
31	mri_oc___31	Splenic hilar disease
32	mri_oc___32	Subdiaphragmatic disease
33	mri_oc___33	Gastro-splenic ligament invasion
34	mri_oc___34	Lesser omentum disease
35	mri_oc___35	Gastric and duodenal serosa disease
36	mri_oc___36	Hepatic portal disease
37	mri_oc___37	Lesser sac disease
38	mri_oc___38	Coeliac lymphadenopathy
39	mri_oc___39	Falciform ligament disease
40	mri_oc___40	Morrisons Pouch and gall bladder fossa disease
41	mri_oc___41	Parenchymal liver metastasis or invasive surface implants

			42 mri_oc___42 Peritoneal and serosal disease along the ascending colon<br>43 mri_oc___43 Appendiceal mass<br>44 mri_oc___44 Diaphragm metastasis<br>45 mri_oc___45 Pleural effusion with pleural infiltration<br>46 mri_oc___46 Paracardiac nodes<br>47 mri_oc___47 Axillary nodes<br>48 mri_oc___48 Supraclavicular nodes<br>49 mri_oc___49 Inguinal nodes<br>51 mri_oc___51 Pancreatic invasion<br>50 mri_oc___50 Other
295	[ps_mri_oc]<br>Show the field ONLY if:<br>[mri_oc(50)] = '1'	Please state	text
296	[mr1_nt_reason_oc]<br>Show the field ONLY if:<br>[mri_yn] = '0'	Reason MRI not performed at diagnosis (select all that apply)	checkbox<br>1 mr1_nt_reason_oc___1 No access to MRI machine<br>2 mr1_nt_reason_oc___2 Patient unable to afford<br>3 mr1_nt_reason_oc___3 Patient refused<br>4 mr1_nt_reason_oc___4 Claustrophobia<br>5 mr1_nt_reason_oc___5 Other imaging performed<br>6 mr1_nt_reason_oc___6 Extreme morbid obesity<br>7 mr1_nt_reason_oc___7 Other
297	[ps_mri1_nt_reas_oc]<br>Show the field ONLY if:<br>[mr1_nt_reason_oc(7)] = '1'	Please state	text
298	[pet_yn]	Positron emission tomography (PET) performed at diagnosis?	yesno<br>1 Yes<br>0 No

299	[pet_oc]<br>Show the field ONLY if:<br>[pet_yn] = '1'	Positron emission tomography (PET) results at diagnosis (select all that apply)	checkbox<br>1 | pet_oc___1 | FDG-avid uptake in ovary(s)<br>2 | pet_oc___2 | FDG-avid uptake in fallopian tube(s)<br>3 | pet_oc___3 | FDG-avid uptake in uterus<br>4 | pet_oc___4 | FDG-avid uptake in peritoneal pouch of douglas<br>5 | pet_oc___5 | FDG-avid uptake in para rectal nodules<br>6 | pet_oc___6 | FDG-avid uptake in sigmoid nodules<br>7 | pet_oc___7 | FDG-avid uptake in rectum<br>8 | pet_oc___8 | FDG-avid uptake in sigmoid<br>9 | pet_oc___9 | FDG-avid uptake in pelvic vascular side wall<br>10 | pet_oc___10 | FDG-avid uptake in ureters<br>11 | pet_oc___11 | FDG-avid uptake in bony and muscular pelvic side wall<br>12 | pet_oc___12 | FDG-avid uptake in sacrum<br>13 | pet_oc___13 | FDG-avid uptake in pelvic lymph nodes<br>14 | pet_oc___14 | FDG-avid uptake in para-aortic lymph nodes<br>15 | pet_oc___15 | FDG-avid uptake in retroperitoneal lymph nodes<br>16 | pet_oc___16 | FDG-avid uptake in mesentry<br>17 | pet_oc___17 | FDG-avid uptake in omentum<br>18 | pet_oc___18 | FDG-avid uptake in transverse mesocolon<br>19 | pet_oc___19 | FDG-avid uptake in spleen<br>20 | pet_oc___20 | FDG-avid uptake in gastro-splenic ligament<br>21 | pet_oc___21 | FDG-avid uptake in lesser omentum<br>22 | pet_oc___22 | FDG-avid uptake in stomach<br>23 | pet_oc___23 | FDG-avid uptake in duodenum<br>24 | pet_oc___24 | FDG-avid uptake in liver<br>25 | pet_oc___25 | FDG-avid uptake in lesser sac<br>26 | pet_oc___26 | FDG-avid uptake in Falciform ligament<br>27 | pet_oc___27 | FDG-avid uptake in gall bladder<br>28 | pet_oc___28 | FDG-avid uptake in ascending colon<br>29 | pet_oc___29 | FDG-avid uptake in appendix<br>30 | pet_oc___30 | FDG-avid uptake in diaphragm<br>31 | pet_oc___31 | FDG-avid uptake in lung(s)<br>32 | pet_oc___32 | FDG-avid uptake in pericardium<br>34 | pet_oc___34 | FDG-avid uptake in pancreas<br>33 | pet_oc___33 | Other
300	[ps_pet_oc]<br>Show the field ONLY if:<br>[pet_oc(33)] = '1'	Please state	text

301	[pet_nt_reason_oc]<br>Show the field ONLY if:<br>[pet_yn] = '0'	Reason positron emission tomography (PET) not performed at diagnosis (select all that apply)	checkbox<br>1 pet_nt_reason_oc___1 No access to PET machine<br>2 pet_nt_reason_oc___2 Patient unable to afford<br>3 pet_nt_reason_oc___3 Patient refused<br>4 pet_nt_reason_oc___4 Uncontrolled diabetes mellitus<br>5 pet_nt_reason_oc___5 Other imaging performed<br>6 pet_nt_reason_oc___6 Other
302	[ps_nt_reas_oc]<br>Show the field ONLY if:<br>[pet_nt_reason_oc(6)] = '1'	Please state	text
303	[oc_stage]	Clinical FIGO Stage (i.e. preoperative staging taking into account imaging, clinical examination)	dropdown<br>1 1A - Tumor limited to 1 ovary, capsule intact, no tumor on surface, negative washings<br>2 1B - Tumor involves both ovaries otherwise like IA<br>3 1C1 - Tumor limited to one or both ovaries with surgical spill<br>4 1C2 - Tumor limited to one or both ovaries with capsule rupture before surgery or tumor on ovarian surface<br>5 IC3 - Tumor limited to one or both ovaries with malignant cells in the ascites or peritoneal washings<br>6 IIA - Tumor involves 1 or both ovaries with extension and/or implant on uterus and/or Fallopian tubes<br>7 IIB - Tumor involves 1 or both ovaries with extension to other pelvic intraperitoneal tissues<br>8 IIIA (i) - Tumor involves 1 or both ovaries with positive retroperitoneal lymph nodes ≤10 mm<br>9 IIIA (ii) - Tumor involves 1 or both ovaries with positive retroperitoneal lymph nodes >10 mm<br>10 IIIB - Tumor involves 1 or both ovaries with macroscopic, extrapelvic, peritoneal metastasis ≤ 2 cm ± positive retroperitoneal lymph nodes. Includes extension to capsule of liver/spleen<br>11 IIIC - Tumor involves 1 or both ovaries with macroscopic, extrapelvic, peritoneal metastasis > 2 cm ± positive retroperitoneal lymph nodes. Includes extension to capsule of liver/spleen<br>12 IVA - Tumor involves 1 or both ovaries with pleural effusion with positive cytology<br>13 IVB - Tumor involves 1 or both ovaries with hepatic and/or splenic parenchymal metastasis, metastasis to extraabdominal organs (including inguinal lymph nodes and lymph nodes outside of the abdominal cavity)
304	[nact_oc]	Neoadjuvant (before surgery) chemotherapy	yesno<br>1 Yes<br>0 No

305	[chem_reg1_nact]<br>Show the field ONLY if:<br>[nact_oc] = '1'	Chemotherapy regimen FIRST line (select all that apply)	checkbox<br>1 chem_reg1_nact___1 Platinum (for example cisplatin, carboplatin)<br>2 chem_reg1_nact___2 Taxane (for example paclitaxel)<br>3 chem_reg1_nact___3 Bevacuzimab<br>4 chem_reg1_nact___4 Clinical trial regimens<br>5 chem_reg1_nact___5 PARP inhibitors (for example olaparib)<br>6 chem_reg1_nact___6 Other
306	[ps_chem_reg1_nact]<br>Show the field ONLY if:<br>[chem_reg1_nact(6)] = '1'	Please state	text
307	[numb_cyc_nact]<br>Show the field ONLY if:<br>[nact_oc] = '1'	Total number of cycles received	text (number)
308	[nact_no_reas]<br>Show the field ONLY if:<br>[nact_oc] = '0'	Reason neoadjuvant chemotherapy not administered (select all that apply)	checkbox<br>1 nact_no_reas___1 Poor performance status<br>2 nact_no_reas___2 Bowel obstruction<br>3 nact_no_reas___3 No access<br>4 nact_no_reas___4 Patient unable to afford<br>5 nact_no_reas___5 Patient refused<br>6 nact_no_reas___6 Not clinically indicated - upfront surgery<br>7 nact_no_reas___7 Other
309	[ps_nact_no_reas]<br>Show the field ONLY if:<br>[nact_no_reas(7)] = '1'	Please state	text
310	[cov_oc]	COVID-19 status	dropdown<br>1 Positive<br>2 Negative<br>3 Not tested
311	[cov_no_oc]<br>Show the field ONLY if:<br>[cov_oc] = '3'	If not tested select reason (select all that apply)	checkbox<br>1 cov_no_oc___1 Not clinically indicated (asymptomatic)<br>2 cov_no_oc___2 Not clinical protocol to routinely test all patients prior to surgery<br>3 cov_no_oc___3 Patient refused<br>4 cov_no_oc___4 Patient unable to afford test<br>6 cov_no_oc___6 Testing not available locally<br>5 cov_no_oc___5 Other
312	[ps_cov_no_oc]<br>Show the field ONLY if:<br>[cov_no_oc(5)] = '1'	Please state	text
313	[bp_oc]	Bowel preparation pre-operatively	yesno<br>1 Yes<br>0 No
314	[peri_op_mx_oc]	Peri-operative management plan in place? (Non- exhaustive list: pre-operative haemoglobin optimisation and iron deficit correction; correction of denutrition and immunonutrition; fluid management; pain management; prevention of post-operative nausea and vomiting).	yesno<br>1 Yes<br>0 No
315	[hb_oc]	Pre-operative Haemoglobin (g/L)	text (number)
316	[wbc_oc]	Pre-operative White Cell Count (10^9/L)	text (number)
317	[plat_oc]	Pre-operative Platelets (10^9/L)	text (number)
318	[pt_oc]	Pre-operative PT (Secs)	text (number)
319	[inr_oc]	Pre-operative INR (Ratio)	text (number)

320	[aptt_oc]	Pre-operative APTT (Secs)	text (number)
321	[fib_oc]	Pre-operative Fibrinogen (g/L)	text (number)
322	[alb_oc]	Pre-operative Albumin (g/L)	text (number)
323	[na_oc]	Pre-operative Sodium (mmol/L)	text (number)
324	[k_oc]	Pre-operative Potassium (mmol/L)	text (number)
325	[urea_oc]	Pre-operative Urea (mmol/L)	text (number)
326	[creat_oc]	Pre-operative Creatinine (μmol/L)	text
327	[egfr_oc]	Pre-operative eGFR - Estimated Glomerular Filtration Rate (mL/min)	text
328	[bili_oc]	Pre-operative Bilirubin total (μmol/L)	text (number)
329	[aat_oc]	Pre-operative Alanine Aminotransaminase (IU/L)	text (number)
330	[alp_oc]	Pre-operative Alkaline Phosphatase (IU/L)	text (number)
331	[ast_oc]	Pre-operative Aspartate Aminotransaminase (IU/L)	text (number)
332	[crp_oc]	Pre-operative CRP - C-reactive protein (mg/L)	text (number)
333	[datsurg_oc]	Section Header: *SURGERY*<br>Date of surgery	text (date_dmy)
334	[anaes_oc]	Anaesthesia (select all that apply)	checkbox<br>1 anaes_oc___1 General<br>2 anaes_oc___2 Spinal<br>3 anaes_oc___3 Epidural
335	[el_em]	Was the surgery an elective or emergency case?	dropdown<br>1 Elective<br>2 Emergency
336	[surg_mod_oc]	Surgical modality	dropdown<br>1 Laparotomy<br>2 Laparoscopic<br>3 Robotic<br>4 Laparoscopic converted to laparotomy<br>5 Robotic converted to laparotomy
337	[ds]	Surgery	dropdown<br>1 Primary debulking - upfront surgery prior to chemotherapy<br>2 Interval debulking - surgery performed after three to four cycles of neoadjuvant chemotherapy/ induction chemotherapy<br>3 Delayed debulking - surgery performed after six cycles of neoadjuvant chemotherapy/ induction chemotherapy
338	[surg_aim]	Surgical aim	dropdown<br>1 Curative<br>2 Curative attempted but then abandoned (i.e. open/close laparotomy)<br>3 Palliative
339	[surgeon_pos]	Lead surgeon performing procedure	dropdown<br>1 Certified consultant/specialist gynaecological oncologist<br>2 Fellow/trainee gynaecological oncologist<br>3 Trained surgeon dedicated to the management of gynaecologic cancer (accounting for more than 50% of his/her practice) or having completed an ESGO accredited fellowship.<br>4 General surgeon<br>5 Surgical oncologist<br>6 Other

340	[ps_surgeon_pos]<br>Show the field ONLY if:<br>[surgeon_pos] = '6'	Please state	text
341	[who_oc]	WHO surgical safety checklist used	yesno<br>1 Yes<br>0 No
342	[resect_oc]<br>Show the field ONLY if:<br>[surg_aim] = '1'	Resectibility achieved (determined at surgery)	dropdown<br>1 R0 resection no visible residual macroscopic disease<br>2 R1 resection < 10 mm visible residual lesions<br>3 R2 resection residual lesions visible of >10 mm or more
343	[r1_reas_oc]<br>Show the field ONLY if:<br>[resect_oc] = '2'	Reason(s) for not achieving complete cytoreduction (select all that apply)	checkbox<br>1 r1_reas_oc___1 Disease more extensive than that shown on imaging<br>2 r1_reas_oc___2 Technically unable to resect<br>3 r1_reas_oc___3 Patient became medically unstable intra-operatively and decision made to not proceed further with surgery<br>4 r1_reas_oc___4 Other
344	[ps_r1_reas_oc]<br>Show the field ONLY if:<br>[r1_reas_oc(4)] = '1'	Please state	text
345	[r2_reas_oc]<br>Show the field ONLY if:<br>[resect_oc] = '3'	Reason(s) for not achieving complete cytoreduction (select all that apply)	checkbox<br>1 r2_reas_oc___1 Disease more extensive than that shown on imaging<br>2 r2_reas_oc___2 Technically unable to resect<br>3 r2_reas_oc___3 Patient became medically unstable intra-operatively and decision made to not proceed further with surgery<br>4 r2_reas_oc___4 Other
346	[ps_r2_reas_oc]<br>Show the field ONLY if:<br>[r2_reas_oc(4)] = '1'	Please state	text

347	[loc_r1_oc]<br>Show the field ONLY if:<br>[resect_oc] = '2'	Location of residual disease (select all that apply)	checkbox<br>1 loc_r1_oc___1 Ovary<br>2 loc_r1_oc___2 Fallopian tube<br>3 loc_r1_oc___3 Uterus<br>4 loc_r1_oc___4 Peritoneal deposits<br>5 loc_r1_oc___5 Para rectal nodules<br>6 loc_r1_oc___6 Sigmoid nodules<br>7 loc_r1_oc___7 Rectal and sigmoid diffuse serosal invasion<br>8 loc_r1_oc___8 Rectal and sigmoid diffuse wall invasion<br>9 loc_r1_oc___9 Rectal and sigmoid diffuse luminal invasion<br>10 loc_r1_oc___10 Pelvic vascular side wall invasion<br>11 loc_r1_oc___11 Ureteric invasion<br>12 loc_r1_oc___12 Bony and muscular pelvic side wall invasion<br>13 loc_r1_oc___13 Diffuse presacral disease<br>14 loc_r1_oc___14 Pelvic lymphadenopathy<br>15 loc_r1_oc___15 Abdominal lymphadenopathy<br>16 loc_r1_oc___16 Retroperitoneal lymphadenopathy<br>17 loc_r1_oc___17 High retroperitoneal nodes at or above the coeliac axis<br>18 loc_r1_oc___18 Diffuse large volume mesenteric disease<br>19 loc_r1_oc___19 Root of small bowel mesentry disease<br>45 loc_r1_oc___45 small bowel serosal metastasis<br>44 loc_r1_oc___44 paracolic gutter metastasis<br>20 loc_r1_oc___20 Stranding, nodular, diffuse omental cake<br>21 loc_r1_oc___21 Involvement of transverse mesocolon<br>22 loc_r1_oc___22 Perisplenic subcapsular disease<br>23 loc_r1_oc___23 Splenic hilar disease<br>24 loc_r1_oc___24 Subdiaphragmatic disease<br>25 loc_r1_oc___25 Gastro-splenic ligament invasion<br>26 loc_r1_oc___26 Lesser omentum disease<br>27 loc_r1_oc___27 Gastric and duodenal serosa disease<br>28 loc_r1_oc___28 Hepatic portal disease<br>29 loc_r1_oc___29 Lesser sac disease<br>30 loc_r1_oc___30 Coeliac lymphadenopathy<br>31 loc_r1_oc___31 Falciform ligament disease<br>32 loc_r1_oc___32 Morrisons Pouch and gall bladder fossa disease<br>33 loc_r1_oc___33 Parenchymal liver metastasis or invasive surface implants<br>34 loc_r1_oc___34 Peritoneal and serosal disease along the ascending colon<br>35 loc_r1_oc___35 Appendiceal mass<br>36 loc_r1_oc___36 Diaphragm metastasis<br>37 loc_r1_oc___37 Pleural effusion with pleural infiltration<br>38 loc_r1_oc___38 Paracardiac nodes

			39	loc_r1_oc___39	Axillary nodes
			40	loc_r1_oc___40	Supraclavicular nodes
			41	loc_r1_oc___41	Inguinal nodes
			43	loc_r1_oc___43	Pancreas
			42	loc_r1_oc___42	Other
348	[ps_loc_r1_oc]<br>Show the field ONLY if:<br>[loc_r1_oc(42)] = '1'	Please state	text		

349	[loc_r2_oc]<br>Show the field ONLY if:<br>[resect_oc] = '3'	Location of residual disease (select all that apply)	checkbox

1	loc_r2_oc___1	Ovary
2	loc_r2_oc___2	Fallopian tube
3	loc_r2_oc___3	Uterus
4	loc_r2_oc___4	Peritoneal deposits
5	loc_r2_oc___5	Para rectal nodules
6	loc_r2_oc___6	Sigmoid nodules
7	loc_r2_oc___7	Rectal and sigmoid diffuse serosal invasion
8	loc_r2_oc___8	Rectal and sigmoid diffuse wall invasion
9	loc_r2_oc___9	Rectal and sigmoid diffuse luminal invasion
10	loc_r2_oc___10	Pelvic vascular side wall invasion
11	loc_r2_oc___11	Ureteric invasion
12	loc_r2_oc___12	Bony and muscular pelvic side wall invasion
13	loc_r2_oc___13	Diffuse presacral disease
14	loc_r2_oc___14	Pelvic lymphadenopathy
15	loc_r2_oc___15	Abdominal lymphadenopathy
16	loc_r2_oc___16	Retroperitoneal lymphadenopathy
17	loc_r2_oc___17	High retroperitoneal nodes at or above the coeliac axis
18	loc_r2_oc___18	Diffuse large volume mesenteric disease
19	loc_r2_oc___19	Root of small bowel mesentry disease
45	loc_r2_oc___45	small bowel serosal metastasis
44	loc_r2_oc___44	paracolic gutter metastasis
20	loc_r2_oc___20	Stranding, nodular, diffuse omental cake
21	loc_r2_oc___21	Involvement of transverse mesocolon
22	loc_r2_oc___22	Perisplenic subcapsular disease
23	loc_r2_oc___23	Splenic hilar disease
24	loc_r2_oc___24	Subdiaphragmatic disease
25	loc_r2_oc___25	Gastro-splenic ligament invasion
26	loc_r2_oc___26	Lesser omentum disease
27	loc_r2_oc___27	Gastric and duodenal serosa disease
28	loc_r2_oc___28	Hepatic portal disease
29	loc_r2_oc___29	Lesser sac disease
30	loc_r2_oc___30	Coeliac lymphadenopathy
31	loc_r2_oc___31	Falciform ligament disease
32	loc_r2_oc___32	Morrisons Pouch and gall bladder fossa disease
33	loc_r2_oc___33	Parenchymal liver metastasis or invasive surface implants
34	loc_r2_oc___34	Peritoneal and serosal disease along the ascending colon
35	loc_r2_oc___35	Appendiceal mass
36	loc_r2_oc___36	Diaphragm metastasis
37	loc_r2_oc___37	Pleural effusion with pleural infiltration
38	loc_r2_oc___38	Paracardiac nodes

			39	loc_r2_oc___39	Axillary nodes
			40	loc_r2_oc___40	Supraclavicular nodes
			41	loc_r2_oc___41	Inguinal nodes
			43	loc_r2_oc___43	Pancreas
			42	loc_r2_oc___42	Other
350	[ps_loc_r2_oc]<br>Show the field ONLY if: [loc_r2_oc(42)] = '1'	Please state	text		

351	[stagsurg_oc]	Findings at surgery (select all that apply)	checkbox

1	stagsurg_oc___1	Ascites
2	stagsurg_oc___2	Unilateral ovarian mass < 5cm
3	stagsurg_oc___3	Unilateral ovarian mass 5-10cm
4	stagsurg_oc___4	Unilateral ovarian mass 10-20cm
5	stagsurg_oc___5	Unilateral ovarian mass >20cm
6	stagsurg_oc___6	Bilateral ovarian mass < 5cm
7	stagsurg_oc___7	Bilateral ovarian mass 5-10cm
8	stagsurg_oc___8	Bilateral ovarian mass 10-20cm
9	stagsurg_oc___9	Bilateral ovarian mass >20cm
10	stagsurg_oc___10	Fallopian tube invasion
11	stagsurg_oc___11	Uterus invasion
12	stagsurg_oc___12	Peritoneal deposits in pouch of douglas
13	stagsurg_oc___13	Para rectal nodules
14	stagsurg_oc___14	Sigmoid nodules
15	stagsurg_oc___15	Rectal and sigmoid diffuse serosal invasion
16	stagsurg_oc___16	Rectal and sigmoid diffuse wall invasion
17	stagsurg_oc___17	Rectal and sigmoid diffuse luminal invasion
18	stagsurg_oc___18	Pelvic vascular side wall invasion
19	stagsurg_oc___19	Ureteric invasion
20	stagsurg_oc___20	Bony and muscular pelvic side wall invasion
21	stagsurg_oc___21	Diffuse presacral disease
22	stagsurg_oc___22	Pelvic lymphadenopathy
23	stagsurg_oc___23	Abdominal lymphadenopathy
24	stagsurg_oc___24	Retroperitoneal lymphadenopathy
25	stagsurg_oc___25	High retroperitoneal nodes at or above the coeliac axis
26	stagsurg_oc___26	Diffuse large volume mesenteric disease
27	stagsurg_oc___27	Root of small bowel mesentry disease
54	stagsurg_oc___54	small bowel serosal metastasis
53	stagsurg_oc___53	paracolic gutter metastasis
28	stagsurg_oc___28	Stranding, nodular, diffuse omental cake
29	stagsurg_oc___29	Involvement of transverse mesocolon
30	stagsurg_oc___30	Perisplenic subcapsular disease
31	stagsurg_oc___31	Splenic hilar disease
32	stagsurg_oc___32	Subdiaphragmatic disease
33	stagsurg_oc___33	Gastro-splenic ligament invasion
34	stagsurg_oc___34	Lesser omentum disease

35	stagsurg_oc___35	Gastric and duodenal serosa disease
36	stagsurg_oc___36	Hepatic portal disease
37	stagsurg_oc___37	Lesser sac disease
38	stagsurg_oc___38	Coeliac lymphadenopathy
39	stagsurg_oc___39	Falciform ligament disease
40	stagsurg_oc___40	Morrisons Pouch and gall bladder fossa disease
41	stagsurg_oc___41	Parenchymal liver metastasis or invasive surface implants
42	stagsurg_oc___42	Peritoneal and serosal disease along the ascending colon
43	stagsurg_oc___43	Appendiceal mass
44	stagsurg_oc___44	Diaphragm metastasis
45	stagsurg_oc___45	Pleural effusion with pleural infiltration
46	stagsurg_oc___46	Paracardiac nodes
47	stagsurg_oc___47	Axillary nodes
48	stagsurg_oc___48	Supraclavicular nodes
49	stagsurg_oc___49	Inguinal nodes
51	stagsurg_oc___51	Pancreatic invasion
50	stagsurg_oc___50	Other
52	stagsurg_oc___52	No visible disease

352	[ps_stag_surg]<br>Show the field ONLY if:<br>[stagsurg_oc(50)] = '1'	Please state	text

<table>
<tr><td>353</td><td>[tis_oc]</td><td>Tissue removed</td><td>checkbox
<table>
<tr><td>1</td><td>tis_oc___1</td><td>peritoneal washings</td></tr>
<tr><td>2</td><td>tis_oc___2</td><td>uterus</td></tr>
<tr><td>3</td><td>tis_oc___3</td><td>cervix</td></tr>
<tr><td>4</td><td>tis_oc___4</td><td>x1 fallopian tube</td></tr>
<tr><td>5</td><td>tis_oc___5</td><td>x2 fallopian tube</td></tr>
<tr><td>6</td><td>tis_oc___6</td><td>x1 ovary</td></tr>
<tr><td>7</td><td>tis_oc___7</td><td>x2 ovaries</td></tr>
<tr><td>8</td><td>tis_oc___8</td><td>peritoneum</td></tr>
<tr><td>9</td><td>tis_oc___9</td><td>spleen</td></tr>
<tr><td>10</td><td>tis_oc___10</td><td>liver capsule</td></tr>
<tr><td>11</td><td>tis_oc___11</td><td>liver parenchyma</td></tr>
<tr><td>12</td><td>tis_oc___12</td><td>gall bladder</td></tr>
<tr><td>13</td><td>tis_oc___13</td><td>stomach</td></tr>
<tr><td>14</td><td>tis_oc___14</td><td>diaphragm resection/stripping</td></tr>
<tr><td>15</td><td>tis_oc___15</td><td>pelvic lymph nodes</td></tr>
<tr><td>16</td><td>tis_oc___16</td><td>para-aortic lymph nodes</td></tr>
<tr><td>17</td><td>tis_oc___17</td><td>inguinal lymph nodes</td></tr>
<tr><td>18</td><td>tis_oc___18</td><td>omentum</td></tr>
<tr><td>19</td><td>tis_oc___19</td><td>rectum</td></tr>
<tr><td>20</td><td>tis_oc___20</td><td>sigmoid</td></tr>
<tr><td>21</td><td>tis_oc___21</td><td>ascending colon</td></tr>
<tr><td>22</td><td>tis_oc___22</td><td>descending colon</td></tr>
<tr><td>23</td><td>tis_oc___23</td><td>transverse colon</td></tr>
<tr><td>24</td><td>tis_oc___24</td><td>appendix</td></tr>
<tr><td>25</td><td>tis_oc___25</td><td>duodenum</td></tr>
<tr><td>26</td><td>tis_oc___26</td><td>jejunum</td></tr>
<tr><td>27</td><td>tis_oc___27</td><td>ileum</td></tr>
<tr><td>28</td><td>tis_oc___28</td><td>bladder</td></tr>
<tr><td>29</td><td>tis_oc___29</td><td>ureter</td></tr>
<tr><td>30</td><td>tis_oc___30</td><td>kidney</td></tr>
<tr><td>32</td><td>tis_oc___32</td><td>pancreas</td></tr>
<tr><td>33</td><td>tis_oc___33</td><td>pelvic peritoneal stripping</td></tr>
<tr><td>34</td><td>tis_oc___34</td><td>abdominal peritoneal stripping</td></tr>
<tr><td>31</td><td>tis_oc___31</td><td>other</td></tr>
</table></td></tr>
<tr><td>354</td><td>[paln_dis_oc]
Show the field ONLY if:
[tis_oc(16)] = '1'</td><td>Select all that apply</td><td>checkbox
<table>
<tr><td>1</td><td>paln_dis_oc___1</td><td>Infra-renal</td></tr>
<tr><td>2</td><td>paln_dis_oc___2</td><td>Supra-renal</td></tr>
</table></td></tr>
<tr><td>355</td><td>[ps_tis_oc]
Show the field ONLY if:
[tis_oc(31)] = '1'</td><td>Please state</td><td>text</td></tr>
<tr><td>356</td><td>[li_stom_oc]</td><td>large bowel stoma</td><td>yesno
<table>
<tr><td>1</td><td>Yes</td></tr>
<tr><td>0</td><td>No</td></tr>
</table></td></tr>
<tr><td>357</td><td>[pb_li_stom]
Show the field ONLY if:
[li_stom_oc] = '1'</td><td>Performed by (select all that apply)</td><td>checkbox
<table>
<tr><td>1</td><td>pb_li_stom___1</td><td>Gynaecological oncologist</td></tr>
<tr><td>2</td><td>pb_li_stom___2</td><td>Colorectal surgeon</td></tr>
<tr><td>3</td><td>pb_li_stom___3</td><td>Urologist</td></tr>
<tr><td>4</td><td>pb_li_stom___4</td><td>Hepatobiliary surgeon</td></tr>
<tr><td>5</td><td>pb_li_stom___5</td><td>Cardiothoracic surgeon</td></tr>
<tr><td>6</td><td>pb_li_stom___6</td><td>General surgeon</td></tr>
<tr><td>7</td><td>pb_li_stom___7</td><td>Other</td></tr>
</table></td></tr>
</table>

358	[ps_pb_li_stom]<br>Show the field ONLY if:<br>[pb_li_stom(7)] = '1'	Please state	text
359	[li_anast_oc]	large bowel anastamosis	yesno<br>1 Yes<br>0 No
360	[anast_typ_oc]<br>Show the field ONLY if:<br>[li_anast_oc] = '1'	Select type of anastamosis	dropdown<br>1 colo-colic<br>2 colo-rectal<br>3 colo-anal
361	[pb_li]<br>Show the field ONLY if:<br>[li_anast_oc] = '1'	Performed by (select all that apply)	checkbox<br>1 pb_li___1 Gynaecological oncologist<br>2 pb_li___2 Colorectal surgeon<br>3 pb_li___3 Urologist<br>4 pb_li___4 Hepatobiliary surgeon<br>5 pb_li___5 Cardiothoracic surgeon<br>6 pb_li___6 General surgeon<br>7 pb_li___7 Other
362	[ps_pb_li]<br>Show the field ONLY if:<br>[pb_li(7)] = '1'	Please state	text
363	[si_stom_oc]	small bowel stoma	yesno<br>1 Yes<br>0 No
364	[ss_oc]<br>Show the field ONLY if:<br>[si_stom_oc] = '1'	Stoma site	dropdown<br>1 Duodenum<br>2 Jejunum<br>3 Ileum
365	[pb_st_stom]<br>Show the field ONLY if:<br>[si_stom_oc] = '1'	Performed by (select all that apply)	checkbox<br>1 pb_st_stom___1 Gynaecological oncologist<br>2 pb_st_stom___2 Colorectal surgeon<br>3 pb_st_stom___3 Urologist<br>4 pb_st_stom___4 Hepatobiliary surgeon<br>5 pb_st_stom___5 Cardiothoracic surgeon<br>6 pb_st_stom___6 General surgeon<br>7 pb_st_stom___7 Other
366	[ps_pb_st_stom]<br>Show the field ONLY if:<br>[pb_st_stom(7)] = '1'	Please state	text
367	[si_anast_oc]	Small bowel anastamosis	yesno<br>1 Yes<br>0 No
368	[pb_si]<br>Show the field ONLY if:<br>[si_anast_oc] = '1'	Performed by (select all that apply)	checkbox<br>1 pb_si___1 Gynaecological oncologist<br>2 pb_si___2 Colorectal surgeon<br>3 pb_si___3 Urologist<br>4 pb_si___4 Hepatobiliary surgeon<br>5 pb_si___5 Cardiothoracic surgeon<br>6 pb_si___6 General surgeon<br>7 pb_si___7 Other
369	[ps_pb_si]<br>Show the field ONLY if:<br>[pb_si(7)] = '1'	Please state	text

370	[mf_oc]	Mucous fistula	yesno<br>1 Yes<br>0 No
371	[pb_mf]<br>Show the field ONLY if:<br>[mf_oc] = '1'	Performed by (select all that apply)	checkbox<br>1 pb_mf___1 Gynaecological oncologist<br>2 pb_mf___2 Colorectal surgeon<br>3 pb_mf___3 Urologist<br>4 pb_mf___4 Hepatobiliary surgeon<br>5 pb_mf___5 Cardiothoracic surgeon<br>6 pb_mf___6 General surgeon<br>7 pb_mf___7 Other
372	[ps_pb_mf]<br>Show the field ONLY if:<br>[pb_mf(7)] = '1'	Please state	text
373	[urostomy_oc]	Urostomy/ileal conduit	yesno<br>1 Yes<br>0 No
374	[pb_ic]<br>Show the field ONLY if:<br>[urostomy_oc] = '1'	Performed by (select all that apply)	checkbox<br>1 pb_ic___1 Gynaecological oncologist<br>2 pb_ic___2 Colorectal surgeon<br>3 pb_ic___3 Urologist<br>4 pb_ic___4 Hepatobiliary surgeon<br>5 pb_ic___5 Cardiothoracic surgeon<br>6 pb_ic___6 General surgeon<br>7 pb_ic___7 Other
375	[ps_pb_ic]<br>Show the field ONLY if:<br>[pb_ic(7)] = '1'	Please state	text
376	[uret_imp_oc]	Ureteric reimplantation	yesno<br>1 Yes<br>0 No
377	[perfo_oc_surg]<br>Show the field ONLY if:<br>[uret_imp_oc] = '1'	Performed by (select all that apply)	checkbox<br>1 perfo_oc_surg___1 Gynaecological oncologist<br>2 perfo_oc_surg___2 Colorectal surgeon<br>3 perfo_oc_surg___3 Urologist<br>4 perfo_oc_surg___4 Hepatobiliary surgeon<br>5 perfo_oc_surg___5 Cardiothoracic surgeon<br>6 perfo_oc_surg___6 General surgeon<br>7 perfo_oc_surg___7 Other
378	[ps_perfo_oc_surg]<br>Show the field ONLY if:<br>[perfo_oc_surg(7)] = '1'	Please state	text
379	[tuu_oc]	Trans uretero-ureteric anastamosis	yesno<br>1 Yes<br>0 No
380	[pb_tuu_oc]<br>Show the field ONLY if:<br>[tuu_oc] = '1'	Performed by (select all that apply)	checkbox<br>1 pb_tuu_oc___1 Gynaecological oncologist<br>2 pb_tuu_oc___2 Colorectal surgeon<br>3 pb_tuu_oc___3 Urologist<br>4 pb_tuu_oc___4 Hepatobiliary surgeon<br>5 pb_tuu_oc___5 Cardiothoracic surgeon<br>6 pb_tuu_oc___6 General surgeon<br>7 pb_tuu_oc___7 Other

381	[ps_pb_tuu_oc]<br>Show the field ONLY if:<br>[pb_tuu_oc(7)] = '1'	Please state	text
382	[nephrostomy_oc]	Nephrostomy	yesno<br>1 Yes<br>0 No
383	[pb_neph]<br>Show the field ONLY if:<br>[nephrostomy_oc] = '1'	Performed by (select all that apply)	checkbox<br>1 pb_neph___1 Gynaecological oncologist<br>2 pb_neph___2 Colorectal surgeon<br>3 pb_neph___3 Urologist<br>4 pb_neph___4 Hepatobiliary surgeon<br>5 pb_neph___5 Cardiothoracic surgeon<br>6 pb_neph___6 General surgeon<br>7 pb_neph___7 Other
384	[ps_pb_neph]<br>Show the field ONLY if:<br>[pb_neph(7)] = '1'	Please state	text
385	[hipec_yn]	Hyperthermic Intraperitoneal Chemotherapy (HIPEC)	yesno<br>1 Yes<br>0 No
386	[hipec_no_reas]<br>Show the field ONLY if:<br>[hipec_yn] = '0'	Reason Hyperthermic Intraperitoneal Chemotherapy (HIPEC) not administered	checkbox<br>1 hipec_no_reas___1 No access to HIPEC<br>2 hipec_no_reas___2 Not standardised practice<br>3 hipec_no_reas___3 Patient unable to afford<br>4 hipec_no_reas___4 Patient refused<br>5 hipec_no_reas___5 Other
387	[ps_hipec_no_reas]<br>Show the field ONLY if:<br>[hipec_no_reas(5)] = '1'	Please state	text
388	[abx_oc]	Intra-operative antibiotics	yesno<br>1 Yes<br>0 No
389	[surg_time_oc]	Length of time of surgery	text (time)
390	[itu_oc]	Section Header: *POST-OPERATIVE*<br>Post-operative recovery in intensive care unit	yesno<br>1 Yes<br>0 No
391	[itu_dys_oc]<br>Show the field ONLY if:<br>[itu_oc] = '1'	Number of days in intensive care unit	text (number)
392	[hdu_oc]	Post-operative recovery in high dependency unit	yesno<br>1 Yes<br>0 No
393	[hdu_dys_oc]<br>Show the field ONLY if:<br>[hdu_oc] = '1'	Number of days in high dependency unit	text (number)
394	[er_oc]	Enhanced recovery?	yesno<br>1 Yes<br>0 No
395	[po_abx_oc]	Prophylactic post operative antibiotics	yesno<br>1 Yes<br>0 No
396	[drn_oc]	Post operative surgical drain	yesno<br>1 Yes<br>0 No

397	[drn_dys_oc]<br>Show the field ONLY if:<br>[drn_oc] = '1'	Number of days in situ	text (number)
398	[uc_oc]	Post operative urinary catheter	yesno<br>1 Yes<br>0 No
399	[uc_dys_oc]<br>Show the field ONLY if:<br>[uc_oc] = '1'	Number of days in situ	text (number)
400	[hb_oc_2]	Post-operative Haemoglobin (g/L) - please state first post-operative result	text (number)
401	[wbc_oc_2]	Post-operative White Cell Count (10^9/L) - please state first post-operative result	text (number)
402	[plat_oc_2]	Post-operative Platelets (10^9/L) - please state first post-operative result	text (number)
403	[pt_oc_2]	Post-operative PT (Secs) - please state first post-operative result	text (number)
404	[inr_oc_2]	Post-operative INR (Ratio) - please state first post-operative result	text (number)
405	[aptt_oc_2]	Post-operative APTT (Secs) - please state first post-operative result	text (number)
406	[fib_oc_2]	Post-operative Fibrinogen (g/L) - please state first post-operative result	text (number)
407	[alb_oc_2]	Post-operative Albumin (g/L) - please state first post-operative result	text (number)
408	[na_oc_2]	Post-operative Sodium (mmol/L) - please state first post-operative result	text (number)
409	[k_oc_2]	Post-operative Potassium (mmol/L) - please state first post-operative result	text (number)
410	[urea_oc_2]	Post-operative Urea (mmol/L) - please state first post-operative result	text (number)
411	[creat_oc_2]	Post-operative Creatinine (μmol/L) - please state first post-operative result	text
412	[egfr_oc_2]	Post-operative eGFR - Estimated Glomerular Filtration Rate (mL/min) - please state first post-operative result	text
413	[bili_oc_2]	Post-operative Bilirubin total (μmol/L) - please state first post-operative result	text (number)
414	[aat_oc_2]	Post-operative Alanine Aminotransaminase (IU/L) - please state first post-operative result	text (number)
415	[alp_oc_2]	Post-operative Alkaline Phosphatase (IU/L) - please state first post-operative result	text (number)
416	[ast_oc_2]	Post-operative Aspartate Aminotransaminase (IU/L) - please state first post-operative result	text (number)
417	[crp_oc_2]	Post-operative CRP - C-reactive protein (mg/L) - please state first post-operative result	text (number)
418	[oc_hist]	Histologic type	dropdown<br>1 High grade serous<br>2 Low grade serous<br>3 Endometrioid<br>4 Mucinous<br>5 Clear cell<br>6 Carcinosarcoma<br>7 Transitional cell/malignant Brenner<br>8 Germ cell<br>9 Sex cord stromal<br>10 Borderline<br>11 Metastatic/Krukenberg<br>12 Undifferentiated<br>13 Mixed epithelial<br>14 Benign

419	[resec_marg_oc]	Histological confirmed resection margins	dropdown<br>1 < 1 mm / tumour on margin<br>2 1-5 mm (NO tumour on margin)<br>3 >5 mm Margins confirmed clear<br>4 Unknown, not available in this hospital<br>5 Not applicable - benign
420	[crs_oc]	Chemotherapy response score following neoadjuvant chemotherapy (NACT) and interval debulking surgery/delayed debulking surgery (IDS/DDS) for tubo-ovarian high grade serous carcinoma	dropdown<br>1 CRS1 (no response/minimal response)<br>2 CRS2 (partial response)<br>3 CRS3 (complete response/near-complete response)<br>4 Data not available<br>5 Not applicable - patient did not have NACT and underwent primary debulking surgery instead<br>6 Not applicable - benign
421	[oc_stage_2]	Final surgical FIGO Stage (i.e. postoperative staging taking into account surgical and pathology findings)	dropdown<br>1 1A - Tumor limited to 1 ovary, capsule intact, no tumor on surface, negative washings<br>2 1B - Tumor involves both ovaries otherwise like IA<br>3 1C1 - Tumor limited to one or both ovaries with surgical spill<br>4 1C2 - Tumor limited to one or both ovaries with capsule rupture before surgery or tumor on ovarian surface<br>5 IC3 - Tumor limited to one or both ovaries with malignant cells in the ascites or peritoneal washings<br>6 IIA - Tumor involves 1 or both ovaries with extension and/or implant on uterus and/or Fallopian tubes<br>7 IIB - Tumor involves 1 or both ovaries with extension to other pelvic intraperitoneal tissues<br>8 IIIA (i) - Tumor involves 1 or both ovaries with positive retroperitoneal lymph nodes ≤10 mm<br>9 IIIA (ii) - Tumor involves 1 or both ovaries with positive retroperitoneal lymph nodes >10 mm<br>10 IIIB - Tumor involves 1 or both ovaries with macroscopic, extrapelvic, peritoneal metastasis ≤ 2 cm ± positive retroperitoneal lymph nodes. Includes extension to capsule of liver/spleen<br>11 IIIC - Tumor involves 1 or both ovaries with macroscopic, extrapelvic, peritoneal metastasis > 2 cm ± positive retroperitoneal lymph nodes. Includes extension to capsule of liver/spleen<br>12 IVA - Tumor involves 1 or both ovaries with pleural effusion with positive cytology<br>13 IVB - Tumor involves 1 or both ovaries with hepatic and/or splenic parenchymal metastasis, metastasis to extraabdominal organs (including inguinal lymph nodes and lymph nodes outside of the abdominal cavity)<br>14 Not applicable - benign
422	[p53]	Section Header: *Immunohistochemistry*<br>p53	radio (Matrix)<br>1 Positive<br>2 Negative<br>3 Not tested
423	[p16]	p16	radio (Matrix)<br>1 Positive<br>2 Negative<br>3 Not tested

424	[er]	ER	radio (Matrix)<br>1 Positive<br>2 Negative<br>3 Not tested
425	[pr]	PR	radio (Matrix)<br>1 Positive<br>2 Negative<br>3 Not tested
426	[ck7]	CK7	radio (Matrix)<br>1 Positive<br>2 Negative<br>3 Not tested
427	[ck20]	CK20	radio (Matrix)<br>1 Positive<br>2 Negative<br>3 Not tested
428	[cdx]	CDX	radio (Matrix)<br>1 Positive<br>2 Negative<br>3 Not tested
429	[wt1]	WT1	radio (Matrix)<br>1 Positive<br>2 Negative<br>3 Not tested
430	[ca125]	Ca125	radio (Matrix)<br>1 Positive<br>2 Negative<br>3 Not tested
431	[cea]	CEA	radio (Matrix)<br>1 Positive<br>2 Negative<br>3 Not tested
432	[vim]	Vimentin	radio (Matrix)<br>1 Positive<br>2 Negative<br>3 Not tested
433	[cal]	Calretinin	radio (Matrix)<br>1 Positive<br>2 Negative<br>3 Not tested
434	[inh]	Inhibin	radio (Matrix)<br>1 Positive<br>2 Negative<br>3 Not tested
435	[hnf1]	HNF1-β	radio (Matrix)<br>1 Positive<br>2 Negative<br>3 Not tested
436	[hcg]	HCG	radio (Matrix)<br>1 Positive<br>2 Negative<br>3 Not tested

437	[afp]	AFP	radio (Matrix)<br>1 Positive<br>2 Negative<br>3 Not tested
438	[arid1a]	ARID1A	radio (Matrix)<br>1 Positive<br>2 Negative<br>3 Not tested
439	[gcdpf]	GCDFP-15	radio (Matrix)<br>1 Positive<br>2 Negative<br>3 Not tested
440	[pax8]	PAX8	radio (Matrix)<br>1 Positive<br>2 Negative<br>3 Not tested
441	[bcat]	β-catenin	radio (Matrix)<br>1 Positive<br>2 Negative<br>3 Not tested
442	[ca199]	CA 19-9	radio (Matrix)<br>1 Positive<br>2 Negative<br>3 Not tested
443	[pw_oc]	Section Header: *Cytology*<br>Peritoneal washings	radio (Matrix)<br>1 Malignant cells<br>2 Normal<br>3 Insufficient<br>4 Not done
444	[asc_oc]	Ascites	radio (Matrix)<br>1 Malignant cells<br>2 Normal<br>3 Insufficient<br>4 Not done
445	[pf_oc]	Pleural fluid	radio (Matrix)<br>1 Malignant cells<br>2 Normal<br>3 Insufficient<br>4 Not done
446	[gt_oc]	Genetic testing performed	yesno<br>1 Yes<br>0 No
447	[gs_gt_oc]<br>Show the field ONLY if:<br>[gt_oc] = '1'	Type of genetic testing (select all that apply)	checkbox<br>1 gs_gt_oc___1 Somatic<br>2 gs_gt_oc___2 Germline
448	[brca1]<br>Show the field ONLY if:<br>[gs_gt_oc(1)] = '1'	Section Header: *Somatic genetic testing results*<br>BRCA1	radio (Matrix)<br>1 Positive<br>2 Negative<br>3 Not tested
449	[brca2]<br>Show the field ONLY if:<br>[gs_gt_oc(1)] = '1'	BRCA2	radio (Matrix)<br>1 Positive<br>2 Negative<br>3 Not tested

450	[rad51c]<br>Show the field ONLY if:<br>[gs_gt_oc(1)] = '1'	RAD51C	radio (Matrix)<br>1 Positive<br>2 Negative<br>3 Not tested
451	[rad51d]<br>Show the field ONLY if:<br>[gs_gt_oc(1)] = '1'	RAD51D	radio (Matrix)<br>1 Positive<br>2 Negative<br>3 Not tested
452	[brip1]<br>Show the field ONLY if:<br>[gs_gt_oc(1)] = '1'	BRIP1	radio (Matrix)<br>1 Positive<br>2 Negative<br>3 Not tested
453	[palb2]<br>Show the field ONLY if:<br>[gs_gt_oc(1)] = '1'	PALB2	radio (Matrix)<br>1 Positive<br>2 Negative<br>3 Not tested
454	[mlh1_oc]<br>Show the field ONLY if:<br>[gs_gt_oc(1)] = '1'	MLH1	radio (Matrix)<br>1 Positive<br>2 Negative<br>3 Not tested
455	[msh2_oc]<br>Show the field ONLY if:<br>[gs_gt_oc(1)] = '1'	MSH2	radio (Matrix)<br>1 Positive<br>2 Negative<br>3 Not tested
456	[msh6_oc]<br>Show the field ONLY if:<br>[gs_gt_oc(1)] = '1'	MSH6	radio (Matrix)<br>1 Positive<br>2 Negative<br>3 Not tested
457	[braca1_g]<br>Show the field ONLY if:<br>[gs_gt_oc(2)] = '1'	Section Header: *Germline genetic testing results*<br>BRCA1	radio (Matrix)<br>1 Positive<br>2 Negative<br>3 Not tested
458	[brca2_g]<br>Show the field ONLY if:<br>[gs_gt_oc(2)] = '1'	BRCA2	radio (Matrix)<br>1 Positive<br>2 Negative<br>3 Not tested
459	[rad51c_g]<br>Show the field ONLY if:<br>[gs_gt_oc(2)] = '1'	RAD51C	radio (Matrix)<br>1 Positive<br>2 Negative<br>3 Not tested
460	[rad51d_g]<br>Show the field ONLY if:<br>[gs_gt_oc(2)] = '1'	RAD51D	radio (Matrix)<br>1 Positive<br>2 Negative<br>3 Not tested
461	[brip1_g]<br>Show the field ONLY if:<br>[gs_gt_oc(2)] = '1'	BRIP1	radio (Matrix)<br>1 Positive<br>2 Negative<br>3 Not tested
462	[palb2_g]<br>Show the field ONLY if:<br>[gs_gt_oc(2)] = '1'	PALB2	radio (Matrix)<br>1 Positive<br>2 Negative<br>3 Not tested

463	[mlh1_g]<br>Show the field ONLY if:<br>[gs_gt_oc(2)] = '1'	MLH1	radio (Matrix)<br>1 Positive<br>2 Negative<br>3 Not tested
464	[msh2_g]<br>Show the field ONLY if:<br>[gs_gt_oc(2)] = '1'	MSH2	radio (Matrix)<br>1 Positive<br>2 Negative<br>3 Not tested
465	[msh6_g]<br>Show the field ONLY if:<br>[gs_gt_oc(2)] = '1'	MSH6	radio (Matrix)<br>1 Positive<br>2 Negative<br>3 Not tested
466	[pgt_no_reas]<br>Show the field ONLY if:<br>[gt_oc] = '0'	Reason genetic testing not performed (select all that apply)	checkbox<br>1 pgt_no_reas___1 Not available<br>2 pgt_no_reas___2 Does not meet clinical criteria for genetic testing<br>3 pgt_no_reas___3 Patient unable to afford<br>4 pgt_no_reas___4 Patient refused<br>5 pgt_no_reas___5 Other
467	[ps_pgt_no_reas]<br>Show the field ONLY if:<br>[pgt_no_reas(5)] = '1'	Please state	text
468	[chemo_oc]	Adjuvant (after surgery) chemotherapy	yesno<br>1 Yes<br>0 No
469	[chemo_reg_oc]<br>Show the field ONLY if:<br>[chemo_oc] = '1'	Chemotherapy regimen FIRST LINE (select all that apply)	checkbox<br>1 chemo_reg_oc___1 Platinum (for example cisplatin, carboplatin)<br>2 chemo_reg_oc___2 Taxane (for example paclitaxel)<br>3 chemo_reg_oc___3 Bevacuzimab<br>4 chemo_reg_oc___4 Clinical trial regimens<br>5 chemo_reg_oc___5 PARP inhibitors (for example olaparib)<br>6 chemo_reg_oc___6 Other
470	[ps_chemo_reg_oc]<br>Show the field ONLY if:<br>[chemo_reg_oc(6)] = '1'	Please state	text
471	[chem_numb]<br>Show the field ONLY if:<br>[chemo_oc] = '1'	Number of cycles planned	text (number, Min: 1)
472	[chemo_no_reas]<br>Show the field ONLY if:<br>[chemo_oc] = '0'	Reason adjuvant chemotherapy not administered (select all that apply)	checkbox<br>1 chemo_no_reas___1 Poor performance status<br>2 chemo_no_reas___2 No access<br>3 chemo_no_reas___3 Patient unable to afford<br>4 chemo_no_reas___4 Patient refused<br>5 chemo_no_reas___5 Other
473	[ps_chemo_no_reas]<br>Show the field ONLY if:<br>[chemo_no_reas(5)] = '1'	Please state	text
474	[ovarian_cancer_initial_presentation_complete]	Section Header: *Form Status*<br>Complete?	dropdown<br>0 Incomplete<br>1 Unverified<br>2 Complete

Instrument: **Ovarian cancer (recurrence)** (ovarian_cancer_recurrence)

475	[date_oc_initial]	Section Header: *PREVIOUS TREATMENT RECEIVED AT PRIMARY DIAGNOSIS*<br>Date of primary/initial diagnosis of ovarian cancer	text (date_dmy)
476	[prim_tx_oc]	Previous treatment at primary/initial diagnosis (select all that apply)	checkbox<br>1 prim_tx_oc___1 Primary debulking surgery<br>2 prim_tx_oc___2 Interval debulking surgery<br>3 prim_tx_oc___3 Delayed debulking surgery<br>4 prim_tx_oc___4 Adjuvant chemotherapy<br>5 prim_tx_oc___5 Neoadjuvant chemotherapy<br>6 prim_tx_oc___6 Radiotherapy<br>7 prim_tx_oc___7 Immunotherapy
477	[dat_pds]<br>Show the field ONLY if:<br>[prim_tx_oc(1)] = '1'	Date of primary debulking surgery	text (date_dmy)
478	[dat_ids]<br>Show the field ONLY if:<br>[prim_tx_oc(2)] = '1'	Date of interval debulking surgery	text (date_dmy)
479	[dat_dds]<br>Show the field ONLY if:<br>[prim_tx_oc(3)] = '1'	Date of delayed debulking surgery	text (date_dmy)
480	[dat_chemo]<br>Show the field ONLY if:<br>[prim_tx_oc(4)] = '1'	Date of adjuvant chemotherapy following primary diagnosis	text (date_dmy)
481	[dat_nact]<br>Show the field ONLY if:<br>[prim_tx_oc(5)] = '1'	Date of neo-adjuvant chemotherapy following primary diagnosis	text (date_dmy)
482	[dat_rt_2]<br>Show the field ONLY if:<br>[prim_tx_oc(6)] = '1'	Date of radiotherapy following primary diagnosis	text (date_dmy)
483	[dat_imm_3]<br>Show the field ONLY if:<br>[prim_tx_oc(7)] = '1'	Date of immunotherapy following primary diagnosis	text (date_dmy)
484	[dat_rec]	Date of recurrence diagnosis	text (date_dmy)
485	[age_v2]	Section Header: *DEMOGRAPHICS*<br>Age of patient	text (number)
486	[eth_oc_v2]	Ethnicity	dropdown<br>1 White (British, Australian, North American, European, other white)<br>2 Asian (South East Asian, Far East Asian, other Asian)<br>3 Black (African, Caribbean, other Black)<br>4 Middle Eastern (Arab, North African, Turkish)<br>5 Mixed (multiple ethnic backgrounds)<br>6 Other
487	[ps_ethn_v2]<br>Show the field ONLY if:<br>[eth_oc_v2] = '6'	Please state	text
488	[bmi_oc_rec]	Body mass index (kg/m2)	text (number)

489	[oc_stage_v2]	Section Header: *PRE-OPERATIVE*<br>FIGO Stage	dropdown<br>1 — 1A - Tumor limited to 1 ovary, capsule intact, no tumor on surface, negative washings<br>2 — 1B - Tumor involves both ovaries otherwise like IA<br>3 — 1C1 - Tumor limited to one or both ovaries with surgical spill<br>4 — 1C2 - Tumor limited to one or both ovaries with capsule rupture before surgery or tumor on ovarian surface<br>5 — IC3 - Tumor limited to one or both ovaries with malignant cells in the ascites or peritoneal washings<br>6 — IIA - Tumor involves 1 or both ovaries with extension and/or implant on uterus and/or Fallopian tubes<br>7 — IIB - Tumor involves 1 or both ovaries with extension to other pelvic intraperitoneal tissues<br>8 — IIIA (i) - Tumor involves 1 or both ovaries with positive retroperitoneal lymph nodes ≤10 mm<br>9 — IIIA (ii) - Tumor involves 1 or both ovaries with positive retroperitoneal lymph nodes >10 mm<br>10 — IIIB - Tumor involves 1 or both ovaries with macroscopic, extrapelvic, peritoneal metastasis ≤ 2 cm ± positive retroperitoneal lymph nodes. Includes extension to capsule of liver/spleen<br>11 — IIIC - Tumor involves 1 or both ovaries with macroscopic, extrapelvic, peritoneal metastasis > 2 cm ± positive retroperitoneal lymph nodes. Includes extension to capsule of liver/spleen<br>12 — IVA - Tumor involves 1 or both ovaries with pleural effusion with positive cytology<br>13 — IVB - Tumor involves 1 or both ovaries with hepatic and/or splenic parenchymal metastasis, metastasis to extraabdominal organs (including inguinal lymph nodes and lymph nodes outside of the abdominal cavity)

490	[asa_oc_v2]	American Society of Anesthesiologists (ASA) physical status	dropdown<br>1 ASA 1: A normal healthy patient. Example: Fit, non-obese (BMI under 30), a nonsmoking patient with good exercise tolerance.<br>2 ASA 2: A patient with a mild systemic disease. Example: Patient with no functional limitations and a well-controlled disease (e.g., treated hypertension, obesity with BMI under 35, frequent social drinker or is a cigarette smoker).<br>3 ASA 3: A patient with a severe systemic disease that is not life-threatening. Example: Patient with some functional limitation as a result of disease (e.g., poorly treated hypertension or diabetes, morbid obesity, chronic renal failure, a bronchospastic disease with intermittent exacerbation, stable angina, implanted pacemaker).<br>4 ASA 4: A patient with a severe systemic disease that is a constant threat to life. Example: Patient with functional limitation from severe, life-threatening disease (e.g., unstable angina, poorly controlled COPD, symptomatic CHF, recent (less than three months ago) myocardial infarction or stroke.<br>5 ASA 5: A moribund patient who is not expected to survive without the operation. The patient is not expected to survive beyond the next 24 hours without surgery. Examples: ruptured abdominal aortic aneurysm, massive trauma, and extensive intracranial hemorrhage with mass effect.<br>6 ASA 6: A brain-dead patient whose organs are being removed with the intention of transplanting them into another patient.
491	[ecog_oc_rec]	ECOG (Eastern Cooperative Oncology Group) performance status	dropdown<br>1 Grade 0 - Fully active, able to carry on all pre-disease performance without restriction<br>2 Grade 1 - Restricted in physically strenuous activity but ambulatory and able to carry out work of a light or sedentary nature, e.g., light house work, office work<br>3 Grade 2 - Ambulatory and capable of all selfcare but unable to carry out any work activities; up and about more than 50% of waking hours<br>4 Grade 3 - Capable of only limited selfcare; confined to bed or chair more than 50% of waking hours<br>5 Grade 4 - Completely disabled; cannot carry on any selfcare; totally confined to bed or chair<br>6 Grade 5 - Dead
492	[comorb_oc2]	Comorbidities	checkbox<br>1 comorb_oc2___1 Cardiovascular<br>2 comorb_oc2___2 Respiratory<br>3 comorb_oc2___3 Urogenital<br>4 comorb_oc2___4 Endocrine<br>5 comorb_oc2___5 Neurological<br>6 comorb_oc2___6 Haematological<br>7 comorb_oc2___7 Psychiatric<br>8 comorb_oc2___8 Gastroenterology<br>9 comorb_oc2___9 Rheumatology and musculoskeletal<br>10 comorb_oc2___10 Oncology<br>11 comorb_oc2___11 None

493	[comorb_cvd_oc2]<br>Show the field ONLY if:<br>[comorb_oc2(1)] = '1'	Select all that apply	checkbox<br>1 comorb_cvd_oc2___1 Hypertension<br>2 comorb_cvd_oc2___2 Coronary heart disease / ischaemic heart disease<br>3 comorb_cvd_oc2___3 Myocardial infarction<br>4 comorb_cvd_oc2___4 Heart failure<br>5 comorb_cvd_oc2___5 Arrhythmia<br>6 comorb_cvd_oc2___6 Heart valve disease<br>7 comorb_cvd_oc2___7 Venous insufficiency<br>8 comorb_cvd_oc2___8 Peripheral vascular disease<br>9 comorb_cvd_oc2___9 Stroke<br>10 comorb_cvd_oc2___10 Transient ischaemic attack<br>11 comorb_cvd_oc2___11 Other
494	[comorb_resp_oc2]<br>Show the field ONLY if:<br>[comorb_oc2(2)] = '1'	Select all that apply	checkbox<br>1 comorb_resp_oc2___1 Asthma<br>2 comorb_resp_oc2___2 Emphysema (COPD)<br>3 comorb_resp_oc2___3 Chronic bronchitis (COPD)<br>4 comorb_resp_oc2___4 Pulmonary fibrosis<br>5 comorb_resp_oc2___5 Sleep apnoea<br>6 comorb_resp_oc2___6 Bronchiectasis<br>7 comorb_resp_oc2___7 Cystic fibrosis<br>8 comorb_resp_oc2___8 Tuberculosis<br>9 comorb_resp_oc2___9 Pneumonia<br>10 comorb_resp_oc2___10 Other
495	[comorb_uro_oc2]<br>Show the field ONLY if:<br>[comorb_oc2(3)] = '1'	Select all that apply	checkbox<br>1 comorb_uro_oc2___1 Chronic kidney disease (not requiring haemodialysis)<br>2 comorb_uro_oc2___2 Chronic kidney disease (requiring haemodialysis)<br>3 comorb_uro_oc2___3 Polycystic kidney disease<br>4 comorb_uro_oc2___4 Glomerulonephropathy<br>5 comorb_uro_oc2___5 Glomerulonephritis<br>6 comorb_uro_oc2___6 Urinary tract infection<br>7 comorb_uro_oc2___7 Other
496	[comorb_endo_oc2]<br>Show the field ONLY if:<br>[comorb_oc2(4)] = '1'	Select all that apply	checkbox<br>1 comorb_endo_oc2___1 Type 1 diabetes mellitus<br>2 comorb_endo_oc2___2 Type 2 diabetes mellitus<br>3 comorb_endo_oc2___3 Hyperthyroidism<br>4 comorb_endo_oc2___4 Hypothyroidism<br>5 comorb_endo_oc2___5 Other
497	[comorb_neuro_oc2]<br>Show the field ONLY if:<br>[comorb_oc2(5)] = '1'	Select all that apply	checkbox<br>1 comorb_neuro_oc2___1 Parkinsons disease<br>2 comorb_neuro_oc2___2 Myasthenia gravis<br>3 comorb_neuro_oc2___3 Dementia<br>4 comorb_neuro_oc2___4 Epilepsy<br>5 comorb_neuro_oc2___5 Other

498	[comorb_haem_oc2]<br>Show the field ONLY if:<br>[comorb_oc2(6)] = '1'	Select all that apply	checkbox<br>1 comorb_haem_oc2___1 Iron deficiency anaemia<br>2 comorb_haem_oc2___2 Folate deficience anaemia<br>3 comorb_haem_oc2___3 Vitamin B12 deficiency anaemia<br>4 comorb_haem_oc2___4 Pernicious anaemia<br>5 comorb_haem_oc2___5 Haemolytic anaemia<br>6 comorb_haem_oc2___6 Aplastic anaemia<br>7 comorb_haem_oc2___7 Thalassemia<br>8 comorb_haem_oc2___8 Sickle cell disease<br>9 comorb_haem_oc2___9 Disseminated intravascular coagulation (DIC)<br>10 comorb_haem_oc2___10 Factor V Leiden<br>11 comorb_haem_oc2___11 Thrombocytosis<br>12 comorb_haem_oc2___12 Idiopathic thrombocytopenic purpura (ITP)<br>13 comorb_haem_oc2___13 Haemophilia<br>14 comorb_haem_oc2___14 Von Willebrand disease<br>15 comorb_haem_oc2___15 Antiphospholipid syndrome<br>16 comorb_haem_oc2___16 Thrombocytopenia<br>17 comorb_haem_oc2___17 Thrombotic thrombocytopenic purpura<br>18 comorb_haem_oc2___18 Deep vein thrombosis<br>19 comorb_haem_oc2___19 Pulmonary embolism<br>20 comorb_haem_oc2___20 Other
499	[comorb_pscy_oc2]<br>Show the field ONLY if:<br>[comorb_oc2(7)] = '1'	Select all that apply	checkbox<br>1 comorb_pscy_oc2___1 Anxiety<br>2 comorb_pscy_oc2___2 Depression<br>3 comorb_pscy_oc2___3 Learning difficulties<br>4 comorb_pscy_oc2___4 Personality disorder<br>5 comorb_pscy_oc2___5 Schizophrenia<br>6 comorb_pscy_oc2___6 Bipolar disorder<br>7 comorb_pscy_oc2___7 Autism<br>8 comorb_pscy_oc2___8 Other

500	[comorb_gi_oc2]<br>Show the field ONLY if:<br>[comorb_oc2(8)] = '1'	Select all that apply	checkbox<br>1 comorb_gi_oc2___1 Crohn's disease<br>2 comorb_gi_oc2___2 Ulcerative colitis<br>3 comorb_gi_oc2___3 Irritable bowel syndrome<br>4 comorb_gi_oc2___4 Celiac Disease<br>5 comorb_gi_oc2___5 Peptic ulcer disease<br>6 comorb_gi_oc2___6 Gallstones<br>7 comorb_gi_oc2___7 Pancreatitis<br>8 comorb_gi_oc2___8 Diverticulitis<br>9 comorb_gi_oc2___9 Non-Alcoholic Fatty Liver Disease<br>10 comorb_gi_oc2___10 Alcoholic fatty liver disease<br>11 comorb_gi_oc2___11 Viral hepatitis<br>12 comorb_gi_oc2___12 Liver failure<br>13 comorb_gi_oc2___13 Other
501	[comorb_msk_oc2]<br>Show the field ONLY if:<br>[comorb_oc2(9)] = '1'	Select all that apply	checkbox<br>1 comorb_msk_oc2___1 Osteoarthritis<br>2 comorb_msk_oc2___2 Rheumatoid arthritis<br>3 comorb_msk_oc2___3 Osteoporosis<br>4 comorb_msk_oc2___4 Lupus<br>5 comorb_msk_oc2___5 Spondyloarthropathies<br>6 comorb_msk_oc2___6 Sjogren's syndrome<br>7 comorb_msk_oc2___7 Gout<br>8 comorb_msk_oc2___8 Other
502	[comorb_onc_oc_2]<br>Show the field ONLY if:<br>[comorb_oc2(10)] = '1'	Select all that apply	checkbox<br>1 comorb_onc_oc_2___1 Breast cancer<br>2 comorb_onc_oc_2___2 Lung cancer<br>3 comorb_onc_oc_2___3 Hepatobiliary cancer<br>4 comorb_onc_oc_2___4 Brain cancer<br>5 comorb_onc_oc_2___5 Leukemia<br>6 comorb_onc_oc_2___6 Lymphoma<br>7 comorb_onc_oc_2___7 Osteosarcoma<br>8 comorb_onc_oc_2___8 Other
503	[lap_oc2]	Previous laparoscopic abdominal surgery	yesno<br>1 Yes<br>0 No
504	[opn_oc2]	Previous laparotomy	yesno<br>1 Yes<br>0 No
505	[mdt_oc_v2]	Discussed at multidisciplinary team meeting/tumour board?	yesno<br>1 Yes<br>0 No
506	[ps_mdt_oc_no_v2]<br>Show the field ONLY if:<br>[mdt_oc_no_v2(2)] = '1'	Please state	text

507	[mdt_tm_oc2]<br>Show the field ONLY if:<br>[mdt_oc_v2] = '1'	Members of multidisciplinary team/tumour board (select all that apply)	checkbox<br>1 mdt_tm_oc2___1 Certified gynaecological oncology surgeon<br>2 mdt_tm_oc2___2 Trained surgeon dedicated to the management of gynaecologic cancer (accounting for more than 50% of his/her practice) or having completed an ESGO-accredited fellowship<br>3 mdt_tm_oc2___3 Radiologist<br>4 mdt_tm_oc2___4 Pathologist<br>5 mdt_tm_oc2___5 Physician certified to deliver chemotherapy (a gynaecologic oncologist in countries where the subspecialty is structured and/or a medical oncologist with special interest in gynaecologic oncology)<br>6 mdt_tm_oc2___6 Clinical oncologist (trained to administer radiotherapy)
508	[mdt_oc_no_v2]<br>Show the field ONLY if:<br>[mdt_oc_v2] = '0'	Reason not discussed (select all that apply)	checkbox<br>1 mdt_oc_no_v2___1 No multidisciplinary team meeting/tumour board<br>2 mdt_oc_no_v2___2 Other
509	[bx_oc_v2]	Diagnostic biopsy performed?	yesno<br>1 Yes<br>0 No
510	[bx_no_reas_v2]<br>Show the field ONLY if:<br>[bx_oc_v2] = '0'	Reason not performed (select all that apply)	checkbox<br>1 bx_no_reas_v2___1 Biopsy not technically feasible<br>2 bx_no_reas_v2___2 Patient refused<br>3 bx_no_reas_v2___3 Patient unable to afford<br>4 bx_no_reas_v2___4 No facilities to perform biopsy<br>5 bx_no_reas_v2___5 Other
511	[ps_bx_no_reas_v2]<br>Show the field ONLY if:<br>[bx_no_reas_v2(5)] = '1'	Please state	text
512	[tm_oc_v2]	Tumour markers tested at recurrence diagnosis (select all that apply)	checkbox<br>1 tm_oc_v2___1 CA125<br>2 tm_oc_v2___2 CA 19-9<br>3 tm_oc_v2___3 CEA<br>4 tm_oc_v2___4 Inhibin A<br>5 tm_oc_v2___5 Inhibin B<br>6 tm_oc_v2___6 AFP<br>7 tm_oc_v2___7 HCG<br>8 tm_oc_v2___8 LDH
513	[ca125_val_v2]<br>Show the field ONLY if:<br>[tm_oc_v2(1)] = '1'	Enter value	text
514	[ca19_9_val_v2]<br>Show the field ONLY if:<br>[tm_oc_v2(2)] = '1'	Enter value	text
515	[cea_val_v2]<br>Show the field ONLY if:<br>[tm_oc_v2(3)] = '1'	Enter value	text
516	[inhibin_val_v2]<br>Show the field ONLY if:<br>[tm_oc_v2(4)] = '1'	Enter value	text

517	[inhibin_val_v3]<br>Show the field ONLY if:<br>[tm_oc_v2(5)] = '1'	Enter value	text
518	[afp_val_v2]<br>Show the field ONLY if:<br>[tm_oc_v2(6)] = '1'	Enter value	text
519	[hcg_val_v2]<br>Show the field ONLY if:<br>[tm_oc_v2(7)] = '1'	Enter value	text
520	[ldh_val_v3]<br>Show the field ONLY if:<br>[tm_oc_v2(8)] = '1'	Enter value	text
521	[uss_v2]	Ultrasound performed at recurrence diagnosis?	yesno<br>1 Yes<br>0 No
522	[uss_nt_reas_oc_v2]<br>Show the field ONLY if:<br>[uss_v2] = '0'	Reason ultrasound not performed at recurrence diagnosis (select all that apply)	checkbox<br>1 uss_nt_reas_oc_v2___1 No access to ultrasound machine<br>2 uss_nt_reas_oc_v2___2 Patient unable to afford<br>3 uss_nt_reas_oc_v2___3 Patient refused<br>4 uss_nt_reas_oc_v2___4 Other imaging performed<br>5 uss_nt_reas_oc_v2___5 Other
523	[ps_uss_nt_reas_oc_v2]<br>Show the field ONLY if:<br>[uss_nt_reas_oc_v2(5)] = '1'	Please state	text
524	[ct_yn_v2]	Staging computerised tomography (CT) performed at recurrence diagnosis?	yesno<br>1 Yes<br>0 No

525	[ct_oc_v2]<br>Show the field ONLY if:<br>[ct_yn_v2] = '1'	Staging computerised tomography (CT) results at recurrence diagnosis (select all that apply)	checkbox

1	ct_oc_v2___1	Ascites
2	ct_oc_v2___2	Pelvic mass post hysterectomy
3	ct_oc_v2___3	Peritoneal deposits in pouch of douglas
4	ct_oc_v2___4	Para rectal nodules
5	ct_oc_v2___5	Sigmoid nodules
6	ct_oc_v2___6	Rectal and sigmoid diffuse serosal invasion
7	ct_oc_v2___7	Rectal and sigmoid diffuse wall invasion
8	ct_oc_v2___8	Rectal and sigmoid diffuse luminal invasion
9	ct_oc_v2___9	Pelvic vascular side wall invasion
10	ct_oc_v2___10	Ureteric invasion
11	ct_oc_v2___11	Bony and muscular pelvic side wall invasion
12	ct_oc_v2___12	Diffuse presacral disease
13	ct_oc_v2___13	Pelvic lymphadenopathy
14	ct_oc_v2___14	Abdominal lymphadenopathy
15	ct_oc_v2___15	Retroperitoneal lymphadenopathy
16	ct_oc_v2___16	High retroperitoneal nodes at or above the coeliac axis
17	ct_oc_v2___17	Diffuse large volume mesenteric disease
18	ct_oc_v2___18	Root of small bowel mesentry disease
44	ct_oc_v2___44	small bowel serosal metastasis
43	ct_oc_v2___43	paracolic gutter metastasis
19	ct_oc_v2___19	Stranding, nodular, diffuse omental cake
20	ct_oc_v2___20	Involvement of transverse mesocolon
21	ct_oc_v2___21	Perisplenic subcapsular disease
22	ct_oc_v2___22	Splenic hilar disease
23	ct_oc_v2___23	Subdiaphragmatic disease
24	ct_oc_v2___24	Gastro-splenic ligament invasion
25	ct_oc_v2___25	Lesser omentum disease
26	ct_oc_v2___26	Gastric and duodenal serosa disease
27	ct_oc_v2___27	Hepatic portal disease
28	ct_oc_v2___28	Lesser sac disease
29	ct_oc_v2___29	Coeliac lymphadenopathy
30	ct_oc_v2___30	Falciform ligament disease
31	ct_oc_v2___31	Morrisons Pouch and gall bladder fossa disease
32	ct_oc_v2___32	Parenchymal liver metastasis or invasive surface implants
33	ct_oc_v2___33	Peritoneal and serosal disease along the ascending colon
34	ct_oc_v2___34	Appendiceal mass
35	ct_oc_v2___35	Diaphragm metastasis
36	ct_oc_v2___36	Pleural effusion with pleural infiltration
37	ct_oc_v2___37	Paracardiac nodes
38	ct_oc_v2___38	Axillary nodes

			39 ct_oc_v2___39 Supraclavicular nodes<br>40 ct_oc_v2___40 Inguinal nodes<br>42 ct_oc_v2___42 Pancreatic invasion<br>41 ct_oc_v2___41 Other
526	[ps_ct_oc_v2]<br>Show the field ONLY if:<br>[ct_oc_v2(41)] = '1'	Please state	text
527	[no_ct_v2]<br>Show the field ONLY if:<br>[ct_yn_v2] = '0'	Reason computerised tomography (CT) not performed at recurrence diagnosis (select all that apply)	checkbox<br>1 no_ct_v2___1 No access to CT machine<br>2 no_ct_v2___2 Patient unable to afford<br>3 no_ct_v2___3 Patient refused<br>4 no_ct_v2___4 Other imaging performed<br>5 no_ct_v2___5 Allergic reaction to contrast<br>6 no_ct_v2___6 Chronic kidney disease<br>7 no_ct_v2___7 Other
528	[ps_no_ct_v2]<br>Show the field ONLY if:<br>[no_ct_v2(7)] = '1'	Please state	text
529	[mri_yn_v2]	Magnetic resonance imaging (MRI) performed at recurrence diagnosis?	yesno<br>1 Yes<br>0 No

530	[mri_oc_v2]<br>Show the field ONLY if:<br>[mri_yn_v2] = '1'	MRI results at recurrence diagnosis (select all that apply)	checkbox

1	mri_oc_v2___1	Ascites
2	mri_oc_v2___2	Pelvic mass post hysterectomy
3	mri_oc_v2___3	Peritoneal deposits in pouch of douglas
4	mri_oc_v2___4	Para rectal nodules
5	mri_oc_v2___5	Sigmoid nodules
6	mri_oc_v2___6	Rectal and sigmoid diffuse serosal invasion
7	mri_oc_v2___7	Rectal and sigmoid diffuse wall invasion
8	mri_oc_v2___8	Rectal and sigmoid diffuse luminal invasion
9	mri_oc_v2___9	Pelvic vascular side wall invasion
10	mri_oc_v2___10	Ureteric invasion
11	mri_oc_v2___11	Bony and muscular pelvic side wall invasion
12	mri_oc_v2___12	Diffuse presacral disease
13	mri_oc_v2___13	Pelvic lymphadenopathy
14	mri_oc_v2___14	Abdominal lymphadenopathy
15	mri_oc_v2___15	Retroperitoneal lymphadenopathy
16	mri_oc_v2___16	High retroperitoneal nodes at or above the coeliac axis
17	mri_oc_v2___17	Diffuse large volume mesenteric disease
18	mri_oc_v2___18	Root of small bowel mesentry disease
44	mri_oc_v2___44	small bowel serosal metastasis
43	mri_oc_v2___43	paracolic gutter metastasis
19	mri_oc_v2___19	Stranding, nodular, diffuse omental cake
20	mri_oc_v2___20	Involvement of transverse mesocolon
21	mri_oc_v2___21	Perisplenic subcapsular disease
22	mri_oc_v2___22	Splenic hilar disease
23	mri_oc_v2___23	Subdiaphragmatic disease
24	mri_oc_v2___24	Gastro-splenic ligament invasion
25	mri_oc_v2___25	Lesser omentum disease
26	mri_oc_v2___26	Gastric and duodenal serosa disease
27	mri_oc_v2___27	Hepatic portal disease
28	mri_oc_v2___28	Lesser sac disease
29	mri_oc_v2___29	Coeliac lymphadenopathy
30	mri_oc_v2___30	Falciform ligament disease
31	mri_oc_v2___31	Morrisons Pouch and gall bladder fossa disease
32	mri_oc_v2___32	Parenchymal liver metastasis or invasive surface implants
33	mri_oc_v2___33	Peritoneal and serosal disease along the ascending colon
34	mri_oc_v2___34	Appendiceal mass
35	mri_oc_v2___35	Diaphragm metastasis
36	mri_oc_v2___36	Pleural effusion with pleural infiltration

			37 mri_oc_v2___37 Paracardiac nodes<br>38 mri_oc_v2___38 Axillary nodes<br>39 mri_oc_v2___39 Supraclavicular nodes<br>40 mri_oc_v2___40 Inguinal nodes<br>42 mri_oc_v2___42 Pancreatic invasion<br>41 mri_oc_v2___41 Other
531	[ps_mri_oc_v2]<br>Show the field ONLY if:<br>[mri_oc_v2(41)] = '1'	Please state	text
532	[mr1_nt_reason_oc_v2]<br>Show the field ONLY if:<br>[mri_yn_v2] = '0'	Reason MRI not performed at recurrence diagnosis (select all that apply)	checkbox<br>1 mr1_nt_reason_oc_v2___1 No access to MRI machine<br>2 mr1_nt_reason_oc_v2___2 Patient unable to afford<br>3 mr1_nt_reason_oc_v2___3 Patient refused<br>4 mr1_nt_reason_oc_v2___4 Claustrophobia<br>5 mr1_nt_reason_oc_v2___5 Other imaging performed<br>6 mr1_nt_reason_oc_v2___6 Extreme morbid obesity<br>7 mr1_nt_reason_oc_v2___7 Other
533	[ps_mri1_nt_reas_oc_v2]<br>Show the field ONLY if:<br>[mr1_nt_reason_oc_v2(7)] = '1'	Please state	text
534	[pet_yn_v2]	Positron emission tomography (PET) performed at recurrence diagnosis?	yesno<br>1 Yes<br>0 No

535	[pet_oc_v2]<br>Show the field ONLY if:<br>[pet_yn_v2] = '1'	Positron emission tomography (PET) results at recurrence diagnosis (select all that apply)	checkbox<br>1 pet_oc_v2___1 FDG-avid uptake in peritoneal pouch of douglas<br>2 pet_oc_v2___2 FDG-avid uptake in para rectal nodules<br>3 pet_oc_v2___3 FDG-avid uptake in sigmoid nodules<br>4 pet_oc_v2___4 FDG-avid uptake in rectum<br>5 pet_oc_v2___5 FDG-avid uptake in sigmoid<br>6 pet_oc_v2___6 FDG-avid uptake in pelvic vascular side wall<br>7 pet_oc_v2___7 FDG-avid uptake in ureters<br>8 pet_oc_v2___8 FDG-avid uptake in bony and muscular pelvic side wall<br>9 pet_oc_v2___9 FDG-avid uptake in sacrum<br>10 pet_oc_v2___10 FDG-avid uptake in pelvic lymph nodes<br>11 pet_oc_v2___11 FDG-avid uptake in para-aortic lymph nodes<br>12 pet_oc_v2___12 FDG-avid uptake in retroperitoneal lymph nodes<br>13 pet_oc_v2___13 FDG-avid uptake in mesentry<br>14 pet_oc_v2___14 FDG-avid uptake in omentum<br>15 pet_oc_v2___15 FDG-avid uptake in transverse mesocolon<br>16 pet_oc_v2___16 FDG-avid uptake in spleen<br>17 pet_oc_v2___17 FDG-avid uptake in gastro-splenic ligament<br>18 pet_oc_v2___18 FDG-avid uptake in lesser omentum<br>19 pet_oc_v2___19 FDG-avid uptake in stomach<br>20 pet_oc_v2___20 FDG-avid uptake in duodenum<br>21 pet_oc_v2___21 FDG-avid uptake in liver<br>22 pet_oc_v2___22 FDG-avid uptake in lesser sac<br>23 pet_oc_v2___23 FDG-avid uptake in Falciform ligament<br>24 pet_oc_v2___24 FDG-avid uptake in gall bladder<br>25 pet_oc_v2___25 FDG-avid uptake in ascending colon<br>26 pet_oc_v2___26 FDG-avid uptake in appendix<br>27 pet_oc_v2___27 FDG-avid uptake in diaphragm<br>28 pet_oc_v2___28 FDG-avid uptake in lung(s)<br>29 pet_oc_v2___29 FDG-avid uptake in pericardium<br>31 pet_oc_v2___31 FDG-avid uptake pancreas<br>30 pet_oc_v2___30 Other
536	[ps_pet_oc_v2]<br>Show the field ONLY if:<br>[pet_oc_v2(30)] = '1'	Please state	text

537	[pet_nt_reason_oc_v2]<br>Show the field ONLY if:<br>[pet_yn_v2] = '0'	Reason positron emission tomography (PET) not performed at recurrence diagnosis (select all that apply)	checkbox<br>1 pet_nt_reason_oc_v2___1 No access to PET machine<br>2 pet_nt_reason_oc_v2___2 Patient unable to afford<br>3 pet_nt_reason_oc_v2___3 Patient refused<br>4 pet_nt_reason_oc_v2___4 Uncontrolled diabetes mellitus<br>5 pet_nt_reason_oc_v2___5 Other imaging performed<br>6 pet_nt_reason_oc_v2___6 Other
538	[ps_nt_reas_oc_v2]<br>Show the field ONLY if:<br>[pet_nt_reason_oc_v2(6)] = '1'	Please state	text
539	[cov_oc_v2]	COVID-19 status	dropdown<br>1 Positive<br>2 Negative<br>3 Not tested
540	[cov_no_oc_v2]<br>Show the field ONLY if:<br>[cov_oc_v2] = '3'	If not tested select reason (select all that apply)	checkbox<br>1 cov_no_oc_v2___1 Not clinically indicated (asymptomatic)<br>2 cov_no_oc_v2___2 Not clinical protocol to routinely test all patients prior to surgery<br>3 cov_no_oc_v2___3 Patient refused<br>4 cov_no_oc_v2___4 Patient unable to afford test<br>6 cov_no_oc_v2___6 Testing not available locally<br>5 cov_no_oc_v2___5 Other
541	[ps_cov_no_oc_v2]<br>Show the field ONLY if:<br>[cov_no_oc_v2(5)] = '1'	Please state	text
542	[bp_oc2]	Bowel preparation pre-operatively	yesno<br>1 Yes<br>0 No
543	[peri_op_oc2]	Peri-operative management plan in place? (Non- exhaustive list: pre-operative haemoglobin optimisation and iron deficit correction; correction of denutrition and immunonutrition; fluid management; pain management; prevention of post-operative nausea and vomiting).	yesno<br>1 Yes<br>0 No
544	[hb_oc2]	Pre-operative Haemoglobin (g/L)	text (number)
545	[wcc_oc2]	Pre-operative White Cell Count (10^9/L)	text (number)
546	[plts_oc2]	Pre-operative Platelets (10^9/L)	text (number)
547	[pt_oc2]	Pre-operative PT (Secs)	text (number)
548	[inr_oc2]	Pre-operative INR (ratio)	text (number)
549	[aptt_oc2]	Pre-operative APTT (Secs)	text (number)
550	[fib_oc2]	Pre-operative Fibrinogen (g/L)	text (number)
551	[alb_oc2]	Pre-operative Albumin (g/L)	text (number)
552	[na_oc2]	Pre-operative Sodium (mmol/L)	text (number)
553	[k_oc2]	Pre-operative Potassium (mmol/L)	text (number)
554	[urea_oc2]	Pre-operative Urea (mmol/L)	text (number)
555	[creat_oc2]	Pre-operative Creatinine (μmol/L)	text
556	[egfr_oc2]	Pre-operative eGFR - Estimated Glomerular Filtration Rate (mL/min)	text
557	[bili_oc2]	Pre-operative Bilirubin total (μmol/L)	text (number)
558	[aat_oc2]	Pre-operative Alanine Aminotransaminase (IU/L)	text (number)
559	[alp_oc2]	Pre-operative Alkaline Phosphatase (IU/L)	text (number)
560	[ast_oc2]	Pre-operative Aspartate Aminotransaminase (IU/L)	text (number)
561	[crp_oc2]	Pre-operative C-Reactive Protein - CRP (mg/L)	text (number)

562	[datsurg_oc_v2]	Section Header: *SURGERY*<br>Date of recurrence surgery	text (date_dmy)
563	[anaes_oc_v2]	Anaesthesia (select all that apply)	checkbox<br>1 anaes_oc_v2___1 General<br>2 anaes_oc_v2___2 Spinal<br>3 anaes_oc_v2___3 Epidural
564	[el_em_v2]	Was the surgery an elective or emergency case?	dropdown<br>1 Elective<br>2 Emergency
565	[surg_mod_oc_v2]	Surgical modality	dropdown<br>1 Laparotomy<br>2 Laparoscopic<br>3 Robotic<br>4 Laparoscopic converted to laparotomy<br>5 Robotic converted to laparotomy
566	[surg_aim_v2]	Surgical aim	dropdown<br>1 Curative<br>2 Curative attempted but then abandoned (i.e. open/close laparotomy)<br>3 Palliative
567	[surgeon_pos_v2]	Lead surgeon performing procedure	dropdown<br>1 Certified consultant/specialist gynaecological oncologist<br>2 Fellow/trainee gynaecological oncologist<br>3 Trained surgeon dedicated to the management of gynaecologic cancer (accounting for more than 50% of his/her practice) or having completed an ESGO accredited fellowship.<br>4 General surgeon<br>5 Surgical oncologist<br>6 Other
568	[ps_surgeon_pos_v2]<br>Show the field ONLY if:<br>[surgeon_pos_v2] = '6'	Please state	text
569	[who_oc2]	WHO surgical safety checklist used	yesno<br>1 Yes<br>0 No
570	[cyto_resect_oc2]	Complete macroscopic cytoreduction at recurrence surgery	yesno<br>1 Yes<br>0 No
571	[cyto_resect_no_oc2]<br>Show the field ONLY if:<br>[cyto_resect_oc2] = '0'	Reason(s) for not achieving complete cytoreduction at recurrence surgery (select all that apply)	checkbox<br>1 cyto_resect_no_oc2___1 Disease more extensive than that shown on imaging<br>2 cyto_resect_no_oc2___2 Technically unable to resect<br>3 cyto_resect_no_oc2___3 Patient became medically unstable intra-operatively and decision made to not proceed further with surgery<br>4 cyto_resect_no_oc2___4 Other
572	[ps_cyto_resect_no_oc2]<br>Show the field ONLY if:<br>[cyto_resect_no_oc2(4)] = '1'	Please state	text

573	[resid_dis_siz_oc2]<br>Show the field ONLY if:<br>[cyto_resect_oc2] = '0'	Size of residual disease (in millimetres)	dropdown<br>1 1-2.5<br>2 2.6-5<br>3 5.1-7.5<br>4 7.6-10<br>5 >10

574	[loc_resid_dis_oc2]<br>Show the field ONLY if:<br>[cyto_resect_oc2] = '0'	Location of residual disease (select all that apply)	checkbox		
			1	loc_resid_dis_oc2___1	Peritoneal deposits
			2	loc_resid_dis_oc2___2	Para rectal nodules
			3	loc_resid_dis_oc2___3	Sigmoid nodules
			4	loc_resid_dis_oc2___4	Rectal and sigmoid diffuse serosal invasion
			5	loc_resid_dis_oc2___5	Rectal and sigmoid diffuse wall invasion
			6	loc_resid_dis_oc2___6	Rectal and sigmoid diffuse luminal invasion
			7	loc_resid_dis_oc2___7	Pelvic vascular side wall invasion
			8	loc_resid_dis_oc2___8	Ureteric invasion
			9	loc_resid_dis_oc2___9	Bony and muscular pelvic side wall invasion
			10	loc_resid_dis_oc2___10	Diffuse presacral disease
			11	loc_resid_dis_oc2___11	Pelvic lymphadenopathy
			12	loc_resid_dis_oc2___12	Abdominal lymphadenopathy
			13	loc_resid_dis_oc2___13	Retroperitoneal lymphadenopathy
			14	loc_resid_dis_oc2___14	High retroperitoneal nodes at or above the coeliac axis
			15	loc_resid_dis_oc2___15	Diffuse large volume mesenteric disease
			16	loc_resid_dis_oc2___16	Root of small bowel mesentry disease
			41	loc_resid_dis_oc2___41	small bowel serosal metastasis
			42	loc_resid_dis_oc2___42	paracolic gutter metastasis
			17	loc_resid_dis_oc2___17	Stranding, nodular, diffuse omental cake
			18	loc_resid_dis_oc2___18	Involvement of transverse mesocolon
			19	loc_resid_dis_oc2___19	Perisplenic subcapsular disease
			20	loc_resid_dis_oc2___20	Splenic hilar disease
			21	loc_resid_dis_oc2___21	Subdiaphragmatic disease
			22	loc_resid_dis_oc2___22	Gastro-splenic ligament invasion
			23	loc_resid_dis_oc2___23	Lesser omentum disease
			24	loc_resid_dis_oc2___24	Gastric and duodenal serosa disease
			25	loc_resid_dis_oc2___25	Hepatic portal disease
			26	loc_resid_dis_oc2___26	Lesser sac disease
			27	loc_resid_dis_oc2___27	Coeliac lymphadenopathy
			28	loc_resid_dis_oc2___28	Falciform ligament disease
			29	loc_resid_dis_oc2___29	Morrisons Pouch and gall bladder fossa disease
			30	loc_resid_dis_oc2___30	Parenchymal liver metastasis or invasive surface implants

			<table><tr><td>31</td><td>loc_resid_dis_oc2___31</td><td>Peritoneal and serosal disease along the ascending colon</td></tr><tr><td>32</td><td>loc_resid_dis_oc2___32</td><td>Appendiceal mass</td></tr><tr><td>33</td><td>loc_resid_dis_oc2___33</td><td>Diaphragm metastasis</td></tr><tr><td>34</td><td>loc_resid_dis_oc2___34</td><td>Pleural effusion with pleural infiltration</td></tr><tr><td>35</td><td>loc_resid_dis_oc2___35</td><td>Paracardiac nodes</td></tr><tr><td>36</td><td>loc_resid_dis_oc2___36</td><td>Axillary nodes</td></tr><tr><td>37</td><td>loc_resid_dis_oc2___37</td><td>Supraclavicular nodes</td></tr><tr><td>38</td><td>loc_resid_dis_oc2___38</td><td>Inguinal nodes</td></tr><tr><td>40</td><td>loc_resid_dis_oc2___40</td><td>Pancreas</td></tr><tr><td>39</td><td>loc_resid_dis_oc2___39</td><td>Other</td></tr></table>
575	[ps_loc_resid_dis_oc2]<br>Show the field ONLY if:<br>[loc_resid_dis_oc2(39)] = '1'	Please state	text

576	[stagsurg_oc_v2]	Findings at surgery (select all that apply)	checkbox

1	stagsurg_oc_v2___1	Ascites
2	stagsurg_oc_v2___2	Pelvic mass post hysterectomy
3	stagsurg_oc_v2___3	Peritoneal deposits in pouch of douglas
4	stagsurg_oc_v2___4	Para rectal nodules
5	stagsurg_oc_v2___5	Sigmoid nodules
6	stagsurg_oc_v2___6	Rectal and sigmoid diffuse serosal invasion
7	stagsurg_oc_v2___7	Rectal and sigmoid diffuse wall invasion
8	stagsurg_oc_v2___8	Rectal and sigmoid diffuse luminal invasion
9	stagsurg_oc_v2___9	Pelvic vascular side wall invasion
10	stagsurg_oc_v2___10	Ureteric invasion
11	stagsurg_oc_v2___11	Bony and muscular pelvic side wall invasion
12	stagsurg_oc_v2___12	Diffuse presacral disease
13	stagsurg_oc_v2___13	Pelvic lymphadenopathy
14	stagsurg_oc_v2___14	Abdominal lymphadenopathy
15	stagsurg_oc_v2___15	Retroperitoneal lymphadenopathy
16	stagsurg_oc_v2___16	High retroperitoneal nodes at or above the coeliac axis
17	stagsurg_oc_v2___17	Diffuse large volume mesenteric disease
18	stagsurg_oc_v2___18	Root of small bowel mesentry disease
44	stagsurg_oc_v2___44	small bowel serosal metastasis
45	stagsurg_oc_v2___45	paracolic gutter metastasis
19	stagsurg_oc_v2___19	Stranding, nodular, diffuse omental cake
20	stagsurg_oc_v2___20	Involvement of transverse mesocolon
21	stagsurg_oc_v2___21	Perisplenic subcapsular disease
22	stagsurg_oc_v2___22	Splenic hilar disease
23	stagsurg_oc_v2___23	Subdiaphragmatic disease
24	stagsurg_oc_v2___24	Gastro-splenic ligament invasion
25	stagsurg_oc_v2___25	Lesser omentum disease
26	stagsurg_oc_v2___26	Gastric and duodenal serosa disease
27	stagsurg_oc_v2___27	Hepatic portal disease
28	stagsurg_oc_v2___28	Lesser sac disease
29	stagsurg_oc_v2___29	Coeliac lymphadenopathy
30	stagsurg_oc_v2___30	Falciform ligament disease
31	stagsurg_oc_v2___31	Morrisons Pouch and gall bladder fossa disease
32	stagsurg_oc_v2___32	Parenchymal liver metastasis or invasive surface implants

			33 stagsurg_oc_v2___33 Peritoneal and serosal disease along the ascending colon<br>34 stagsurg_oc_v2___34 Appendiceal mass<br>35 stagsurg_oc_v2___35 Diaphragm disease<br>36 stagsurg_oc_v2___36 Pleural effusion with pleural infiltration<br>37 stagsurg_oc_v2___37 Paracardiac nodes<br>38 stagsurg_oc_v2___38 Axillary nodes<br>39 stagsurg_oc_v2___39 Supraclavicular nodes<br>40 stagsurg_oc_v2___40 Inguinal nodes<br>42 stagsurg_oc_v2___42 Pancreatic invasion<br>41 stagsurg_oc_v2___41 Other<br>43 stagsurg_oc_v2___43 No visible disease
577	[ps_stag_surg_v2]<br>Show the field ONLY if: [stagsurg_oc_v2(41)] = '1'	Please state	text
578	[tis_oc_v2]	Tissue removed	checkbox<br>1 tis_oc_v2___1 peritoneal washings<br>2 tis_oc_v2___2 peritoneum<br>3 tis_oc_v2___3 spleen<br>4 tis_oc_v2___4 liver capsule<br>5 tis_oc_v2___5 liver parenchyma<br>6 tis_oc_v2___6 gall bladder<br>7 tis_oc_v2___7 stomach<br>8 tis_oc_v2___8 diaphragm resection/stripping<br>9 tis_oc_v2___9 pelvic lymph nodes<br>10 tis_oc_v2___10 para-aortic lymph nodes<br>11 tis_oc_v2___11 inguinal lymph nodes<br>12 tis_oc_v2___12 omentum<br>13 tis_oc_v2___13 rectum<br>14 tis_oc_v2___14 sigmoid<br>15 tis_oc_v2___15 ascending colon<br>16 tis_oc_v2___16 descending colon<br>17 tis_oc_v2___17 transverse colon<br>18 tis_oc_v2___18 appendix<br>19 tis_oc_v2___19 duodenum<br>20 tis_oc_v2___20 jejunum<br>21 tis_oc_v2___21 ileum<br>22 tis_oc_v2___22 bladder<br>23 tis_oc_v2___23 ureter<br>24 tis_oc_v2___24 kidney<br>26 tis_oc_v2___26 pancreas<br>27 tis_oc_v2___27 pelvic peritoneal stripping<br>28 tis_oc_v2___28 abdominal peritoneal stripping<br>25 tis_oc_v2___25 other
579	[paln_dis_oc2]<br>Show the field ONLY if: [tis_oc_v2(10)] = '1'	Select all that apply	checkbox<br>1 paln_dis_oc2___1 Infra-renal<br>2 paln_dis_oc2___2 Supra-renal
580	[ps_tis_oc_v2]<br>Show the field ONLY if: [tis_oc_v2(25)] = '1'	Please state	text

581	[li_stom_oc_v2]	large bowel stoma	yesno<br>1 Yes<br>0 No
582	[pb_li_stom_v2]<br>Show the field ONLY if:<br>[li_stom_oc_v2] = '1'	Performed by (select all that apply)	checkbox<br>1 pb_li_stom_v2___1 Gynaecological oncologist<br>2 pb_li_stom_v2___2 Colorectal surgeon<br>3 pb_li_stom_v2___3 Urologist<br>4 pb_li_stom_v2___4 Hepatobiliary surgeon<br>5 pb_li_stom_v2___5 Cardiothoracic surgeon<br>6 pb_li_stom_v2___6 General surgeon<br>7 pb_li_stom_v2___7 Other
583	[ps_pb_li_stom_v2]<br>Show the field ONLY if:<br>[pb_li_stom_v2(7)] = '1'	Please state	text
584	[li_anast_oc_v2]	large bowel anastamosis	yesno<br>1 Yes<br>0 No
585	[col_anast_typ_oc2]<br>Show the field ONLY if:<br>[li_anast_oc_v2] = '1'	Type of anastamosis	dropdown<br>1 colo-colic<br>2 colo-rectal<br>3 colo-anal
586	[pb_li_v2]<br>Show the field ONLY if:<br>[li_anast_oc_v2] = '1'	Performed by (select all that apply)	checkbox<br>1 pb_li_v2___1 Gynaecological oncologist<br>2 pb_li_v2___2 Colorectal surgeon<br>3 pb_li_v2___3 Urologist<br>4 pb_li_v2___4 Hepatobiliary surgeon<br>5 pb_li_v2___5 Cardiothoracic surgeon<br>6 pb_li_v2___6 General surgeon<br>7 pb_li_v2___7 Other
587	[ps_pb_li_v2]<br>Show the field ONLY if:<br>[pb_li_v2(7)] = '1'	Please state	text
588	[si_stom_oc_v2]	small bowel stoma	yesno<br>1 Yes<br>0 No
589	[ss_oc_2]<br>Show the field ONLY if:<br>[si_stom_oc_v2] = '1'	Site of stoma	dropdown<br>1 Duodenum<br>2 Jejunum<br>3 Ileum
590	[pb_st_stom_v2]<br>Show the field ONLY if:<br>[si_stom_oc_v2] = '1'	Performed by (select all that apply)	checkbox<br>1 pb_st_stom_v2___1 Gynaecological oncologist<br>2 pb_st_stom_v2___2 Colorectal surgeon<br>3 pb_st_stom_v2___3 Urologist<br>4 pb_st_stom_v2___4 Hepatobiliary surgeon<br>5 pb_st_stom_v2___5 Cardiothoracic surgeon<br>6 pb_st_stom_v2___6 General surgeon<br>7 pb_st_stom_v2___7 Other
591	[ps_pb_st_stom_v2]<br>Show the field ONLY if:<br>[pb_st_stom_v2(7)] = '1'	Please state	text
592	[si_anast_oc_v2]	Small bowel anastamosis	yesno<br>1 Yes<br>0 No

593	[pb_si_v2]<br>Show the field ONLY if:<br>[si_anast_oc_v2] = '1'	Performed by (select all that apply)	checkbox<br>1 pb_si_v2___1 Gynaecological oncologist<br>2 pb_si_v2___2 Colorectal surgeon<br>3 pb_si_v2___3 Urologist<br>4 pb_si_v2___4 Hepatobiliary surgeon<br>5 pb_si_v2___5 Cardiothoracic surgeon<br>6 pb_si_v2___6 General surgeon<br>7 pb_si_v2___7 Other
594	[ps_pb_si_v2]<br>Show the field ONLY if:<br>[pb_si_v2(7)] = '1'	Please state	text
595	[mf_oc_v2]	Mucous fistula	yesno<br>1 Yes<br>0 No
596	[pb_mf_v2]<br>Show the field ONLY if:<br>[mf_oc_v2] = '1'	Performed by (select all that apply)	checkbox<br>1 pb_mf_v2___1 Gynaecological oncologist<br>2 pb_mf_v2___2 Colorectal surgeon<br>3 pb_mf_v2___3 Urologist<br>4 pb_mf_v2___4 Hepatobiliary surgeon<br>5 pb_mf_v2___5 Cardiothoracic surgeon<br>6 pb_mf_v2___6 General surgeon<br>7 pb_mf_v2___7 Other
597	[ps_pb_mf_v2]<br>Show the field ONLY if:<br>[pb_mf_v2(7)] = '1'	Please state	text
598	[urostomy_oc_v2]	Urostomy/ileal conduit	yesno<br>1 Yes<br>0 No
599	[pb_ic_v2]<br>Show the field ONLY if:<br>[urostomy_oc_v2] = '1'	Performed by (select all that apply)	checkbox<br>1 pb_ic_v2___1 Gynaecological oncologist<br>2 pb_ic_v2___2 Colorectal surgeon<br>3 pb_ic_v2___3 Urologist<br>4 pb_ic_v2___4 Hepatobiliary surgeon<br>5 pb_ic_v2___5 Cardiothoracic surgeon<br>6 pb_ic_v2___6 General surgeon<br>7 pb_ic_v2___7 Other
600	[ps_pb_ic_v2]<br>Show the field ONLY if:<br>[pb_ic_v2(7)] = '1'	Please state	text
601	[tuu_oc2]	Trans uretero-ureteric anastamosis	yesno<br>1 Yes<br>0 No
602	[pb_tuu_oc2]<br>Show the field ONLY if:<br>[tuu_oc2] = '1'	Performed by (Select all that apply)	checkbox<br>1 pb_tuu_oc2___1 Gynaecological oncologist<br>2 pb_tuu_oc2___2 Colorectal surgeon<br>3 pb_tuu_oc2___3 Urologist<br>4 pb_tuu_oc2___4 Hepatobiliary surgeon<br>5 pb_tuu_oc2___5 Cardiothoracic surgeon<br>6 pb_tuu_oc2___6 General surgeon<br>7 pb_tuu_oc2___7 Other
603	[ps_pb_tuu_oc2]<br>Show the field ONLY if:<br>[pb_tuu_oc2(7)] = '1'	Please state	text

604	[uret_imp_oc_v2]	Ureteric reimplantation	yesno<br>1 Yes<br>0 No
605	[perfo_oc_surg_v2]<br>Show the field ONLY if:<br>[uret_imp_oc_v2] = '1'	Performed by (select all that apply)	checkbox<br>1 perfo_oc_surg_v2___1 Gynaecological oncologist<br>2 perfo_oc_surg_v2___2 Colorectal surgeon<br>3 perfo_oc_surg_v2___3 Urologist<br>4 perfo_oc_surg_v2___4 Hepatobiliary surgeon<br>5 perfo_oc_surg_v2___5 Cardiothoracic surgeon<br>6 perfo_oc_surg_v2___6 General surgeon<br>7 perfo_oc_surg_v2___7 Other
606	[ps_perfo_oc_surg_v2]<br>Show the field ONLY if:<br>[perfo_oc_surg_v2(7)] = '1'	Please state	text
607	[nephrostomy_oc_v2]	Nephrostomy	yesno<br>1 Yes<br>0 No
608	[pb_neph_v2]<br>Show the field ONLY if:<br>[nephrostomy_oc_v2] = '1'	Performed by (select all that apply)	checkbox<br>1 pb_neph_v2___1 Gynaecological oncologist<br>2 pb_neph_v2___2 Colorectal surgeon<br>3 pb_neph_v2___3 Urologist<br>4 pb_neph_v2___4 Hepatobiliary surgeon<br>5 pb_neph_v2___5 Cardiothoracic surgeon<br>6 pb_neph_v2___6 General surgeon<br>7 pb_neph_v2___7 Other
609	[ps_pb_neph_v2]<br>Show the field ONLY if:<br>[pb_neph_v2(7)] = '1'	Please state	text
610	[hipec_yn_v2]	Hyperthermic Intraperitoneal Chemotherapy (HIPEC)	yesno<br>1 Yes<br>0 No
611	[hipec_no_reas_v2]<br>Show the field ONLY if:<br>[hipec_yn_v2] = '0'	Reason Hyperthermic Intraperitoneal Chemotherapy (HIPEC) not administered	checkbox<br>1 hipec_no_reas_v2___1 No access to HIPEC<br>2 hipec_no_reas_v2___2 Not standardised practice<br>3 hipec_no_reas_v2___3 Patient unable to afford<br>4 hipec_no_reas_v2___4 Patient refused<br>5 hipec_no_reas_v2___5 Other
612	[ps_hipec_no_reas_v2]<br>Show the field ONLY if:<br>[hipec_no_reas_v2(5)] = '1'	Please state	text
613	[abx_oc2]	Intraoperative antibiotics	yesno<br>1 Yes<br>0 No
614	[surg_time_oc_v2]	Length of time of surgery	text (time)
615	[itu_oc2]	Section Header: *POST-OPERATIVE*<br>Post-operative recovery in intensive care unit	yesno<br>1 Yes<br>0 No
616	[itu_dys_oc2]<br>Show the field ONLY if:<br>[itu_oc2] = '1'	Number of days in intensive care unit	text (number)
617	[hdu_oc2]	Post-operative recovery in high dependency unit	yesno<br>1 Yes<br>0 No

618	[hdu_dys_oc2]<br>Show the field ONLY if:<br>[hdu_oc2] = '1'	Number of days in high dependency unit	text (number)
619	[er_roc]	Enhanced recovery?	yesno<br>1 Yes<br>0 No
620	[po_abx_oc2]	Prophylactic post operative antibiotics	yesno<br>1 Yes<br>0 No
621	[drn_oc2]	Post operative surgical drain	yesno<br>1 Yes<br>0 No
622	[drn_dys_oc2]<br>Show the field ONLY if:<br>[drn_oc2] = '1'	Number of days in situ	text (number)
623	[uc_oc2]	Post operative urinary catheter	yesno<br>1 Yes<br>0 No
624	[cath_dys_oc3]<br>Show the field ONLY if:<br>[uc_oc2] = '1'	Number of days in situ	text (number)
625	[phb_oc2]	Post-operative Haemoglobin (g/L) - please state first post-operative result	text (number)
626	[pwbc_oc2]	Post-operative White Cell Count (10^9/L) - please state first post-operative result	text (number)
627	[pplat_oc2]	Post-operative Platelets (10^9/L) - please state first post-operative result	text (number)
628	[ppt_oc2]	Post-operative PT (Secs) - please state first post-operative result	text (number)
629	[pinr_oc2]	Post-operative INR (Ratio) - please state first post-operative result	text (number)
630	[paptt_oc2]	Post-operative APTT (Secs) - please state first post-operative result	text (number)
631	[pfib_oc2]	Post-operative Fibrinogen (g/L) - please state first post-operative result	text (number)
632	[palb_oc2]	Post-operative Albumin (g/L) - please state first post-operative result	text (number)
633	[pna_oc2]	Post-operative Sodium (mmol/L) - please state first post-operative result	text (number)
634	[pk_oc2]	Post-operative Potassium (mmol/L) - please state first post-operative result	text (number)
635	[purea_oc2]	Post-operative Urea (mmol/L) - please state first post-operative result	text (number)
636	[pcreat_oc2]	Post-operative Creatinine (μmol/L) - please state first post-operative result	text
637	[pegfr_oc2]	Post-operative eGFR - Estimated Glomerular Filtration Rate (mL/min) - please state first post-operative result	text
638	[pbili_oc2]	Post-operative Bilirubin total (μmol/L) - please state first post-operative result	text (number)
639	[paat_oc2]	Post-operative Alanine Aminotransaminase (IU/L) - please state first post-operative result	text (number)
640	[palp_oc2]	Post-operative Alkaline Phosphatase (IU/L) - please state first post-operative result	text (number)
641	[past_oc2]	Post-operative Aspartate Aminotransaminase (IU/L) - please state first post-operative result	text (number)
642	[pcrp_oc2]	Post-operative CRP - C-reactive protein (mg/L) - please state first post-operative result	text (number)

643	[oc_hist_v2]	Histologic type	dropdown<br>1 High grade serous<br>2 Low grade serous<br>3 Endometrioid<br>4 Mucinous<br>5 Clear cell<br>6 Carcinosarcoma<br>7 Transitional cell/malignant Brenner<br>8 Germ cell<br>9 Sex cord stromal<br>10 Borderline<br>11 Metastatic/Krukenberg<br>12 Undifferentiated<br>13 Mixed epithelial
644	[resect_oc2]	Histological confirmed resection margins following recurrence surgery	dropdown<br>1 < 1 mm / tumour on margin<br>2 1-5 mm (NO tumour on margin)<br>3 >5 mm Margins confirmed clear<br>4 Unknown, not available in this hospital
645	[p53_v2]	Section Header: *Immunohistochemistry*<br>p53	radio (Matrix)<br>1 Positive<br>2 Negative<br>3 Not tested
646	[p16_v2]	p16	radio (Matrix)<br>1 Positive<br>2 Negative<br>3 Not tested
647	[er_v2]	ER	radio (Matrix)<br>1 Positive<br>2 Negative<br>3 Not tested
648	[pr_v2]	PR	radio (Matrix)<br>1 Positive<br>2 Negative<br>3 Not tested
649	[ck7_v2]	CK7	radio (Matrix)<br>1 Positive<br>2 Negative<br>3 Not tested
650	[ck20_v2]	CK20	radio (Matrix)<br>1 Positive<br>2 Negative<br>3 Not tested
651	[cdx_v2]	CDX	radio (Matrix)<br>1 Positive<br>2 Negative<br>3 Not tested
652	[wt1_v2]	WT1	radio (Matrix)<br>1 Positive<br>2 Negative<br>3 Not tested

653	[ca125_v2]	Ca125	radio (Matrix)<br>1 Positive<br>2 Negative<br>3 Not tested
654	[cea_v2]	CEA	radio (Matrix)<br>1 Positive<br>2 Negative<br>3 Not tested
655	[vim_v2]	Vimentin	radio (Matrix)<br>1 Positive<br>2 Negative<br>3 Not tested
656	[cal_v2]	Calretinin	radio (Matrix)<br>1 Positive<br>2 Negative<br>3 Not tested
657	[inh_v2]	Inhibin	radio (Matrix)<br>1 Positive<br>2 Negative<br>3 Not tested
658	[hnf1_v2]	HNF1-β	radio (Matrix)<br>1 Positive<br>2 Negative<br>3 Not tested
659	[hcg_v2]	HCG	radio (Matrix)<br>1 Positive<br>2 Negative<br>3 Not tested
660	[afp_v2]	AFP	radio (Matrix)<br>1 Positive<br>2 Negative<br>3 Not tested
661	[arid1a_v2]	ARID1A	radio (Matrix)<br>1 Positive<br>2 Negative<br>3 Not tested
662	[gcdpf_v2]	GCDFP-15	radio (Matrix)<br>1 Positive<br>2 Negative<br>3 Not tested
663	[pax8_v2]	PAX8	radio (Matrix)<br>1 Positive<br>2 Negative<br>3 Not tested
664	[bcat_v2]	β-catenin	radio (Matrix)<br>1 Positive<br>2 Negative<br>3 Not tested
665	[ca199_v2]	CA 19-9	radio (Matrix)<br>1 Positive<br>2 Negative<br>3 Not tested

666	[pw_oc_v2]	Section Header: *Cytology*<br>Peritoneal washings	radio (Matrix)<br>1 Malignant cells<br>2 Normal<br>3 Insufficient<br>4 Not done
667	[asc_oc_v2]	Ascites	radio (Matrix)<br>1 Malignant cells<br>2 Normal<br>3 Insufficient<br>4 Not done
668	[pf_oc_v2]	Pleural fluid	radio (Matrix)<br>1 Malignant cells<br>2 Normal<br>3 Insufficient<br>4 Not done
669	[gt_oc_v2]	Genetic testing performed?	yesno<br>1 Yes<br>0 No
670	[sg_gt_2]<br>Show the field ONLY if:<br>[gt_oc_v2] = '1'	Type of genetic testing (select all that apply)	checkbox<br>1 sg_gt_2___1 Somatic<br>2 sg_gt_2___2 Germline
671	[brca1_v2]<br>Show the field ONLY if:<br>[sg_gt_2(1)] = '1'	Section Header: *Somatic genetic testing results*<br>BRCA1	radio (Matrix)<br>1 Positive<br>2 Negative<br>3 Not tested
672	[brca2_v2]<br>Show the field ONLY if:<br>[sg_gt_2(1)] = '1'	BRCA2	radio (Matrix)<br>1 Positive<br>2 Negative<br>3 Not tested
673	[rad51c_v2]<br>Show the field ONLY if:<br>[sg_gt_2(1)] = '1'	RAD51C	radio (Matrix)<br>1 Positive<br>2 Negative<br>3 Not tested
674	[rad51d_v2]<br>Show the field ONLY if:<br>[sg_gt_2(1)] = '1'	RAD51D	radio (Matrix)<br>1 Positive<br>2 Negative<br>3 Not tested
675	[brip1_v2]<br>Show the field ONLY if:<br>[sg_gt_2(1)] = '1'	BRIP1	radio (Matrix)<br>1 Positive<br>2 Negative<br>3 Not tested
676	[palb2_v2]<br>Show the field ONLY if:<br>[sg_gt_2(1)] = '1'	PALB2	radio (Matrix)<br>1 Positive<br>2 Negative<br>3 Not tested
677	[mlh1_oc_v2]<br>Show the field ONLY if:<br>[sg_gt_2(1)] = '1'	MLH1	radio (Matrix)<br>1 Positive<br>2 Negative<br>3 Not tested
678	[msh2_oc_v2]<br>Show the field ONLY if:<br>[sg_gt_2(1)] = '1'	MSH2	radio (Matrix)<br>1 Positive<br>2 Negative<br>3 Not tested

679	[msh6_oc_v2]<br>Show the field ONLY if:<br>[sg_gt_2(1)] = '1'	MSH6	radio (Matrix)<br>1 Positive<br>2 Negative<br>3 Not tested
680	[brca1_g_gt2]<br>Show the field ONLY if:<br>[sg_gt_2(2)] = '1'	Section Header: *Germline genetic testing results*<br>BRCA1	radio (Matrix)<br>1 Positive<br>2 Negative<br>3 Not tested
681	[brca2_g_gt2]<br>Show the field ONLY if:<br>[sg_gt_2(2)] = '1'	BRCA2	radio (Matrix)<br>1 Positive<br>2 Negative<br>3 Not tested
682	[rad51c_g_gt2]<br>Show the field ONLY if:<br>[sg_gt_2(2)] = '1'	RAD51C	radio (Matrix)<br>1 Positive<br>2 Negative<br>3 Not tested
683	[rad51d_g_gt2]<br>Show the field ONLY if:<br>[sg_gt_2(2)] = '1'	RAD51D	radio (Matrix)<br>1 Positive<br>2 Negative<br>3 Not tested
684	[brip1_g_gt2]<br>Show the field ONLY if:<br>[sg_gt_2(2)] = '1'	BRIP1	radio (Matrix)<br>1 Positive<br>2 Negative<br>3 Not tested
685	[palb2_g_gt2]<br>Show the field ONLY if:<br>[sg_gt_2(2)] = '1'	PALB2	radio (Matrix)<br>1 Positive<br>2 Negative<br>3 Not tested
686	[mlh1_g_gt2]<br>Show the field ONLY if:<br>[sg_gt_2(2)] = '1'	MLH1	radio (Matrix)<br>1 Positive<br>2 Negative<br>3 Not tested
687	[msh2_g_gt2]<br>Show the field ONLY if:<br>[sg_gt_2(2)] = '1'	MSH2	radio (Matrix)<br>1 Positive<br>2 Negative<br>3 Not tested
688	[msh6_g_gt2]<br>Show the field ONLY if:<br>[sg_gt_2(2)] = '1'	MSH6	radio (Matrix)<br>1 Positive<br>2 Negative<br>3 Not tested
689	[pgt_no_reas_v2]<br>Show the field ONLY if:<br>[gt_oc_v2] = '0'	Reason genetic testing not performed (select all that apply)	checkbox<br>1 pgt_no_reas_v2___1 Not available<br>2 pgt_no_reas_v2___2 Does not meet clinical criteria for genetic testing<br>3 pgt_no_reas_v2___3 Patient unable to afford<br>4 pgt_no_reas_v2___4 Patient refused<br>5 pgt_no_reas_v2___5 Other
690	[ps_pgt_no_reas_v2]<br>Show the field ONLY if:<br>[pgt_no_reas_v2(5)] = '1'	Please state	text
691	[chemo_oc_v2]	Chemotherapy administered following diagnosis of recurrence	yesno<br>1 Yes<br>0 No

692	[chemo_reg_oc_v2]<br>Show the field ONLY if:<br>[chemo_oc_v2] = '1'	Chemotherapy regimen following diagnosis of recurrence (select all that apply)	checkbox<br>1 chemo_reg_oc_v2___1 Platinum (for example cisplatin, carboplatin)<br>2 chemo_reg_oc_v2___2 Taxane (for example paclitaxel)<br>3 chemo_reg_oc_v2___3 Bevacuzimab<br>4 chemo_reg_oc_v2___4 Clinical trial regimens<br>5 chemo_reg_oc_v2___5 PARP inhibitors (for example olaparib)<br>6 chemo_reg_oc_v2___6 Other
693	[ps_chemo_reg_oc_v2]<br>Show the field ONLY if:<br>[chemo_reg_oc_v2(6)] = '1'	Please state	text
694	[chem_numb_v2]<br>Show the field ONLY if:<br>[chemo_oc_v2] = '1'	Number of cycles planned	text (number, Min: 1)
695	[chemo_no_reas_v2]<br>Show the field ONLY if:<br>[chemo_oc_v2] = '0'	Reason chemotherapy not administered (select all that apply)	checkbox<br>1 chemo_no_reas_v2___1 Poor performance status<br>2 chemo_no_reas_v2___2 No access<br>3 chemo_no_reas_v2___3 Patient unable to afford<br>4 chemo_no_reas_v2___4 Patient refused<br>5 chemo_no_reas_v2___5 Other
696	[ps_chemo_no_reas_v2]<br>Show the field ONLY if:<br>[chemo_no_reas_v2(5)] = '1'	Please state	text
697	[ovarian_cancer_recurrence_complete]	Section Header: *Form Status*<br>Complete?	dropdown<br>0 Incomplete<br>1 Unverified<br>2 Complete
Instrument: **Uterine Cancer (initial presentation)** (endometrial_cancer_initial_presentation)			
698	[dat_dx_ec]	Section Header: *DEMOGRAPHICS*<br>Date of primary diagnosis	text (date_dmy)
699	[age_ec]	Age of patient	text (number)
700	[eth_ec]	Ethnicity	dropdown<br>1 White (British, Australian, North American, European, other white)<br>2 Asian (South East Asian, Far East Asian, other Asian)<br>3 Black (African, Caribbean, other Black)<br>4 Middle Eastern (Arab, North African, Turkish)<br>5 Mixed (multiple ethnic background)<br>6 Other
701	[ps_eth_ec]<br>Show the field ONLY if:<br>[eth_ec] = '6'	Please state	text
702	[bmi_ec]	Body mass index (kg/m2)	text (number)
703	[hrt_ec]	History of unopposed oestrogen use (i.e. systemic oestrogen only hormone replacement therapy)?	yesno<br>1 Yes<br>0 No
704	[ein_ec]	History of atypical hyperplasia/endometrial intraepithelial neoplasia?	yesno<br>1 Yes<br>0 No

705	[asa_ec]	Section Header: *PRE-OPERATIVE*<br>American Society of Anesthesiologists (ASA) physical status	dropdown<br>1 ASA 1: A normal healthy patient. Example: Fit, non-obese (BMI under 30), a nonsmoking patient with good exercise tolerance.<br>2 ASA 2: A patient with a mild systemic disease. Example: Patient with no functional limitations and a well-controlled disease (e.g., treated hypertension, obesity with BMI under 35, frequent social drinker or is a cigarette smoker).<br>3 ASA 3: A patient with a severe systemic disease that is not life-threatening. Example: Patient with some functional limitation as a result of disease (e.g., poorly treated hypertension or diabetes, morbid obesity, chronic renal failure, a bronchospastic disease with intermittent exacerbation, stable angina, implanted pacemaker).<br>4 ASA 4: A patient with a severe systemic disease that is a constant threat to life. Example: Patient with functional limitation from severe, life-threatening disease (e.g., unstable angina, poorly controlled COPD, symptomatic CHF, recent (less than three months ago) myocardial infarction or stroke.<br>5 ASA 5: A moribund patient who is not expected to survive without the operation. The patient is not expected to survive beyond the next 24 hours without surgery. Examples: ruptured abdominal aortic aneurysm, massive trauma, and extensive intracranial hemorrhage with mass effect.<br>6 ASA 6: A brain-dead patient whose organs are being removed with the intention of transplanting them into another patient.
706	[ecog_ec]	ECOG (Eastern Cooperative Oncology Group) performance status	dropdown<br>1 Grade 0 - Fully active, able to carry on all pre-disease performance without restriction<br>2 Grade 1 - Restricted in physically strenuous activity but ambulatory and able to carry out work of a light or sedentary nature, e.g., light house work, office work<br>3 Grade 2 - Ambulatory and capable of all selfcare but unable to carry out any work activities; up and about more than 50% of waking hours<br>4 Grade 3 - Capable of only limited selfcare; confined to bed or chair more than 50% of waking hours<br>5 Grade 4 - Completely disabled; cannot carry on any selfcare; totally confined to bed or chair<br>6 Grade 5 - Dead
707	[comorb_ec]	Co-morbidities	checkbox<br>1 comorb_ec___1 Cardiovascular<br>2 comorb_ec___2 Respiratory<br>3 comorb_ec___3 Urogenital<br>4 comorb_ec___4 Endocrine<br>5 comorb_ec___5 Neurological<br>6 comorb_ec___6 Haematological<br>7 comorb_ec___7 Psychiatric<br>8 comorb_ec___8 Gastroenterology<br>9 comorb_ec___9 Rheumatology and musculoskeletal<br>10 comorb_ec___10 Oncology<br>11 comorb_ec___11 None

708	[cvd_ec]<br>Show the field ONLY if:<br>[comorb_ec(1)] = '1'	Select all that apply	checkbox<br>1 cvd_ec___1 Hypertension<br>2 cvd_ec___2 Coronary heart disease / ischaemic heart disease<br>3 cvd_ec___3 Myocardial infarction<br>4 cvd_ec___4 Heart failure<br>5 cvd_ec___5 Arrhythmia<br>6 cvd_ec___6 Heart valve disease<br>7 cvd_ec___7 Venous insufficiency<br>8 cvd_ec___8 Peripheral vascular disease<br>9 cvd_ec___9 Stroke<br>10 cvd_ec___10 Transient ischaemic attack<br>11 cvd_ec___11 Other
709	[comorb_resp_ec]<br>Show the field ONLY if:<br>[comorb_ec(2)] = '1'	Select all that apply	checkbox<br>1 comorb_resp_ec___1 Asthma<br>2 comorb_resp_ec___2 Emphysema (COPD)<br>3 comorb_resp_ec___3 Chronic bronchitis (COPD)<br>4 comorb_resp_ec___4 Pulmonary fibrosis<br>5 comorb_resp_ec___5 Sleep apnoea<br>6 comorb_resp_ec___6 Bronchiectasis<br>7 comorb_resp_ec___7 Cystic fibrosis<br>8 comorb_resp_ec___8 Tuberculosis<br>9 comorb_resp_ec___9 Pneumonia<br>10 comorb_resp_ec___10 Other
710	[comorb_uro_ec]<br>Show the field ONLY if:<br>[comorb_ec(3)] = '1'	Select all that apply	checkbox<br>1 comorb_uro_ec___1 Chronic kidney disease (not requiring haemodialysis)<br>2 comorb_uro_ec___2 Chronic kidney disease (requiring haemodialysis)<br>3 comorb_uro_ec___3 Polycystic kidney disease<br>4 comorb_uro_ec___4 Glomerulonephropathy<br>5 comorb_uro_ec___5 Glomerulonephritis<br>6 comorb_uro_ec___6 Urinary tract infection<br>7 comorb_uro_ec___7 Other
711	[comorb_endo_ec]<br>Show the field ONLY if:<br>[comorb_ec(4)] = '1'	Select all that apply	checkbox<br>1 comorb_endo_ec___1 Type 1 diabetes mellitus<br>2 comorb_endo_ec___2 Type 2 diabetes mellitus<br>3 comorb_endo_ec___3 Hyperthyroidism<br>4 comorb_endo_ec___4 Hypothyroidism<br>5 comorb_endo_ec___5 Other
712	[comorb_neuro_ec]<br>Show the field ONLY if:<br>[comorb_ec(5)] = '1'	Select all that apply	checkbox<br>1 comorb_neuro_ec___1 Parkinsons disease<br>2 comorb_neuro_ec___2 Myasthenia gravis<br>3 comorb_neuro_ec___3 Dementia<br>4 comorb_neuro_ec___4 Epilepsy<br>5 comorb_neuro_ec___5 Other

713	[comorb_haem_ec]<br>Show the field ONLY if:<br>[comorb_ec(6)] = '1'	Select all that apply	checkbox<br>1 comorb_haem_ec___1 Iron deficiency anaemia<br>2 comorb_haem_ec___2 Folate deficience anaemia<br>3 comorb_haem_ec___3 Vitamin B12 deficiency anaemia<br>4 comorb_haem_ec___4 Pernicious anaemia<br>5 comorb_haem_ec___5 Haemolytic anaemia<br>6 comorb_haem_ec___6 Aplastic anaemia<br>7 comorb_haem_ec___7 Thalassemia<br>8 comorb_haem_ec___8 Sickle cell disease<br>9 comorb_haem_ec___9 Disseminated intravascular coagulation (DIC)<br>10 comorb_haem_ec___10 Factor V Leiden<br>11 comorb_haem_ec___11 Thrombocytosis<br>12 comorb_haem_ec___12 Idiopathic thrombocytopenic purpura (ITP)<br>13 comorb_haem_ec___13 Haemophilia<br>14 comorb_haem_ec___14 Von Willebrand disease<br>15 comorb_haem_ec___15 Antiphospholipid syndrome<br>16 comorb_haem_ec___16 Thrombocytopenia<br>17 comorb_haem_ec___17 Thrombotic thrombocytopenic purpura<br>18 comorb_haem_ec___18 Deep vein thrombosis<br>19 comorb_haem_ec___19 Pulmonary embolism<br>20 comorb_haem_ec___20 Other
714	[comorb_pscy_ec]<br>Show the field ONLY if:<br>[comorb_ec(7)] = '1'	Select all that apply	checkbox<br>1 comorb_pscy_ec___1 Anxiety<br>2 comorb_pscy_ec___2 Depression<br>3 comorb_pscy_ec___3 Learning difficulties<br>4 comorb_pscy_ec___4 Personality disorder<br>5 comorb_pscy_ec___5 Schizophrenia<br>6 comorb_pscy_ec___6 Bipolar disorder<br>7 comorb_pscy_ec___7 Autism<br>8 comorb_pscy_ec___8 Other
715	[comorb_gi_ec]<br>Show the field ONLY if:<br>[comorb_ec(8)] = '1'	Select all that apply	checkbox<br>1 comorb_gi_ec___1 Crohn's disease<br>2 comorb_gi_ec___2 Ulcerative colitis<br>3 comorb_gi_ec___3 Irritable bowel syndrome<br>4 comorb_gi_ec___4 Celiac Disease<br>5 comorb_gi_ec___5 Peptic ulcer disease<br>6 comorb_gi_ec___6 Gallstones<br>7 comorb_gi_ec___7 Pancreatitis<br>8 comorb_gi_ec___8 Diverticulitis<br>9 comorb_gi_ec___9 Non-Alcoholic Fatty Liver Disease<br>10 comorb_gi_ec___10 Alcoholic fatty liver disease<br>11 comorb_gi_ec___11 Viral hepatitis<br>12 comorb_gi_ec___12 Liver failure<br>13 comorb_gi_ec___13 Other

716	[comorb_msk_ec]<br>Show the field ONLY if:<br>[comorb_ec(9)] = '1'	Select all that apply	checkbox<br>1 comorb_msk_ec___1 Osteoarthritis<br>2 comorb_msk_ec___2 Rheumatoid arthritis<br>3 comorb_msk_ec___3 Osteoporosis<br>4 comorb_msk_ec___4 Lupus<br>5 comorb_msk_ec___5 Spondyloarthropathies<br>6 comorb_msk_ec___6 Sjogren's syndrome<br>7 comorb_msk_ec___7 Gout<br>8 comorb_msk_ec___8 Other
717	[comorb_onc_ec]<br>Show the field ONLY if:<br>[comorb_ec(10)] = '1'	Select all that apply	checkbox<br>1 comorb_onc_ec___1 Breast cancer<br>2 comorb_onc_ec___2 Lung cancer<br>3 comorb_onc_ec___3 Hepatobiliary cancer<br>4 comorb_onc_ec___4 Brain cancer<br>5 comorb_onc_ec___5 Leukemia<br>6 comorb_onc_ec___6 Lymphoma<br>7 comorb_onc_ec___7 Osteosarcoma<br>8 comorb_onc_ec___8 Other
718	[lap_ec]	Previous laparoscopic abdominal surgery	yesno<br>1 Yes<br>0 No
719	[opn_ec]	Previous laparotomy	yesno<br>1 Yes<br>0 No
720	[mdt_ec]	Discussed at multidisciplinary team meeting/tumour board?	yesno<br>1 Yes<br>0 No
721	[mdt_tm_ec]<br>Show the field ONLY if:<br>[mdt_ec] = '1'	Multidisciplinary team/tumour board team members (select all that apply)	checkbox<br>1 mdt_tm_ec___1 Certified gynaecological oncology surgeon<br>2 mdt_tm_ec___2 Trained surgeon dedicated to the management of gynaecologic cancer or having completed an ESGO-accredited fellowship<br>3 mdt_tm_ec___3 Radiologist<br>4 mdt_tm_ec___4 Radiation/clinical oncologist (administering radiotherapy)<br>5 mdt_tm_ec___5 Medical oncologist (administering chemotherapy)<br>6 mdt_tm_ec___6 Pathologist
722	[mdt_ec_no]<br>Show the field ONLY if:<br>[mdt_ec] = '0'	Reason not discussed (select all that apply)	checkbox<br>1 mdt_ec_no___1 No multidisciplinary team meeting/tumour board<br>2 mdt_ec_no___2 Other
723	[ps_mdt_ec_no]<br>Show the field ONLY if:<br>[mdt_ec_no(2)] = '1'	Please state	text
724	[uss_ec]	Ultrasound scan performed at diagnosis?	yesno<br>1 Yes<br>0 No
725	[et_mm]<br>Show the field ONLY if:<br>[uss_ec] = '1'	Endometrial thickness in millimetres (mm)	text (number)

726	[uss_no_reas]<br>Show the field ONLY if:<br>[uss_ec] = '0'	Reason ultrasound not performed at diagnosis (select all that apply)	checkbox<br>1 uss_no_reas___1 No access to ultrasound machine<br>2 uss_no_reas___2 Patient unable to afford<br>3 uss_no_reas___3 Patient refused<br>4 uss_no_reas___4 Other imaging performed<br>5 uss_no_reas___5 Other
727	[ps_uss_no_reas]<br>Show the field ONLY if:<br>[uss_no_reas(5)] = '1'	Please state	text
728	[hystero_ec]	Diagnostic hysteroscopy performed and endometrial biopsy obtained at diagnosis?	yesno<br>1 Yes<br>0 No
729	[ebx_ec]<br>Show the field ONLY if:<br>[hystero_ec] = '0'	Endometrial biopsy obtained without hysteroscopy at diagnosis?	yesno<br>1 Yes<br>0 No
730	[bx_ec_no_reas]<br>Show the field ONLY if:<br>[ebx_ec] = '0'	Reason endometrial biopsy not performed (Select all that apply)	checkbox<br>1 bx_ec_no_reas___1 Biopsy not technically feasible<br>2 bx_ec_no_reas___2 Patient refused<br>3 bx_ec_no_reas___3 Patient unable to afford<br>4 bx_ec_no_reas___4 No facilities to perform biopsy<br>5 bx_ec_no_reas___5 Other
731	[ps_bx_ec_no_reas]<br>Show the field ONLY if:<br>[bx_ec_no_reas(5)] = '1'	Please state	text
732	[mri_ec]	Magnetic resonance imaging (MRI) pelvis/abdominal-pelvis performed at diagnosis?	yesno<br>1 Yes<br>0 No

<table>
<tr><td>733</td><td>[mri_find_ec]
Show the field ONLY if:
[mri_ec] = '1'</td><td>MRI pelvis/abdominal-pelvis findings at diagnosis (select all that apply)</td><td>checkbox<table>
<tr><td>1</td><td>mri_find_ec___1</td><td>Tumour confined to endometrium</td></tr>
<tr><td>2</td><td>mri_find_ec___2</td><td>Tumour invading < 50% of myometrium</td></tr>
<tr><td>3</td><td>mri_find_ec___3</td><td>Tumour invading >50% of myometrium</td></tr>
<tr><td>4</td><td>mri_find_ec___4</td><td>x1 tubo-ovarian invasion</td></tr>
<tr><td>5</td><td>mri_find_ec___5</td><td>x2 tubo-ovarian invasion</td></tr>
<tr><td>6</td><td>mri_find_ec___6</td><td>Cervical invasion</td></tr>
<tr><td>7</td><td>mri_find_ec___7</td><td>Vaginal invasion</td></tr>
<tr><td>8</td><td>mri_find_ec___8</td><td>Parametrial invasion</td></tr>
<tr><td>9</td><td>mri_find_ec___9</td><td>Pelvic sidewall invasion</td></tr>
<tr><td>10</td><td>mri_find_ec___10</td><td>Pelvic lymphadenopathy</td></tr>
<tr><td>11</td><td>mri_find_ec___11</td><td>Inguinal lymphadenopathy</td></tr>
<tr><td>12</td><td>mri_find_ec___12</td><td>Para-aortic lymphadenopathy</td></tr>
<tr><td>13</td><td>mri_find_ec___13</td><td>x1 hydroureter</td></tr>
<tr><td>14</td><td>mri_find_ec___14</td><td>x2 hydroureter</td></tr>
<tr><td>15</td><td>mri_find_ec___15</td><td>Ureteric invasion</td></tr>
<tr><td>16</td><td>mri_find_ec___16</td><td>x1 hydronephrosis</td></tr>
<tr><td>17</td><td>mri_find_ec___17</td><td>x2 hydronephrosis</td></tr>
<tr><td>18</td><td>mri_find_ec___18</td><td>Bladder invasion</td></tr>
<tr><td>19</td><td>mri_find_ec___19</td><td>Para rectal nodules</td></tr>
<tr><td>20</td><td>mri_find_ec___20</td><td>Sigmoid nodules</td></tr>
<tr><td>21</td><td>mri_find_ec___21</td><td>Rectal and sigmoid diffuse serosal invasion</td></tr>
<tr><td>22</td><td>mri_find_ec___22</td><td>Rectal and sigmoid diffuse wall invasion</td></tr>
<tr><td>23</td><td>mri_find_ec___23</td><td>Rectal and sigmoid diffuse luminal invasion</td></tr>
<tr><td>24</td><td>mri_find_ec___24</td><td>Peritoneal nodules</td></tr>
<tr><td>25</td><td>mri_find_ec___25</td><td>Omental disease</td></tr>
<tr><td>26</td><td>mri_find_ec___26</td><td>Ascites</td></tr>
<tr><td>27</td><td>mri_find_ec___27</td><td>Splenic invasion</td></tr>
<tr><td>28</td><td>mri_find_ec___28</td><td>Liver invasion</td></tr>
<tr><td>29</td><td>mri_find_ec___29</td><td>Other</td></tr>
</table></td></tr>
<tr><td>734</td><td>[ps_mri_find_ec]
Show the field ONLY if:
[mri_find_ec(29)] = '1'</td><td>Please state</td><td>text</td></tr>
<tr><td>735</td><td>[mri_ec_no]
Show the field ONLY if:
[mri_ec] = '0'</td><td>Reason MRI pelvis/abdominal-pelvis not performed at diagnosis (select all that apply)</td><td>checkbox<table>
<tr><td>1</td><td>mri_ec_no___1</td><td>No access to MRI machine</td></tr>
<tr><td>2</td><td>mri_ec_no___2</td><td>Patient unable to afford</td></tr>
<tr><td>3</td><td>mri_ec_no___3</td><td>Patient refused</td></tr>
<tr><td>4</td><td>mri_ec_no___4</td><td>Claustrophobia</td></tr>
<tr><td>5</td><td>mri_ec_no___5</td><td>Other imaging performed</td></tr>
<tr><td>6</td><td>mri_ec_no___6</td><td>Extreme morbid obesity</td></tr>
<tr><td>7</td><td>mri_ec_no___7</td><td>Other</td></tr>
</table></td></tr>
<tr><td>736</td><td>[pc_mri_ec_no]
Show the field ONLY if:
[mri_ec_no(7)] = '1'</td><td>Please state</td><td>text</td></tr>
<tr><td>737</td><td>[ct_ec]</td><td>Staging computerised tomography (CT) performed at diagnosis?</td><td>yesno<table>
<tr><td>1</td><td>Yes</td></tr>
<tr><td>0</td><td>No</td></tr>
</table></td></tr>
</table>

738	[ct_ec_find]<br>Show the field ONLY if:<br>[ct_ec] = '1'	Staging CT findings at diagnosis (select all that apply)	checkbox<br>1 ct_ec_find___1 Invasion of uterus<br>2 ct_ec_find___2 x1 tubo-ovarian invasion<br>3 ct_ec_find___3 x2 tubo-ovarian invasion<br>4 ct_ec_find___4 Cervical invasion<br>5 ct_ec_find___5 Vaginal invasion<br>6 ct_ec_find___6 Parametrial invasion<br>7 ct_ec_find___7 Pelvic sidewall invasion<br>8 ct_ec_find___8 Pelvic lymphadenopathy<br>9 ct_ec_find___9 Inguinal lymphadenopathy<br>10 ct_ec_find___10 Para-aortic lymphadenopathy<br>11 ct_ec_find___11 x1 hydroureter<br>12 ct_ec_find___12 x2 hydroureter<br>13 ct_ec_find___13 Ureteric invasion<br>14 ct_ec_find___14 x1 hydronephrosis<br>15 ct_ec_find___15 x2 hydronephrosis<br>16 ct_ec_find___16 Bladder invasion<br>17 ct_ec_find___17 Pleural effusion<br>18 ct_ec_find___18 Lung nodules<br>19 ct_ec_find___19 Para rectal nodules<br>20 ct_ec_find___20 Sigmoid nodules<br>21 ct_ec_find___21 Rectal and sigmoid diffuse serosal invasion<br>22 ct_ec_find___22 Rectal and sigmoid diffuse wall invasion<br>23 ct_ec_find___23 Rectal and sigmoid diffuse luminal invasion<br>24 ct_ec_find___24 Peritoneal nodules<br>25 ct_ec_find___25 Omental disease<br>26 ct_ec_find___26 Ascites<br>27 ct_ec_find___27 Splenic invasion<br>28 ct_ec_find___28 Liver invasion<br>29 ct_ec_find___29 Other
739	[ps_ct_ec_find]<br>Show the field ONLY if:<br>[ct_ec_find(29)] = '1'	Please state	text
740	[ct_ec_no]<br>Show the field ONLY if:<br>[ct_ec] = '0'	Reason staging CT not performed at diagnosis (select all that apply)	checkbox<br>1 ct_ec_no___1 No access to CT machine<br>2 ct_ec_no___2 Patient unable to afford<br>3 ct_ec_no___3 Patient refused<br>4 ct_ec_no___4 Other imaging performed<br>5 ct_ec_no___5 Allergic reaction to contrast<br>6 ct_ec_no___6 Chronic kidney disease<br>7 ct_ec_no___7 Other
741	[ps_ct_ec_no]<br>Show the field ONLY if:<br>[ct_ec_no(7)] = '1'	Please state	text
742	[petct_ec]	Positron emission tomogrophy-computerised tomography (PET-CT) performed at diagnosis?	yesno<br>1 Yes<br>0 No

743	[petct_find]<br>Show the field ONLY if:<br>[petct_ec] = '1'	PET-CT findings at diagnosis (select all that apply)	checkbox<br>1 petct_find___1 FDG-avid uptake in uterus<br>2 petct_find___2 FDG-avid uptake in fallopian tubes<br>3 petct_find___3 FDG-avid uptake ovaries<br>4 petct_find___4 FDG-avid uptake in cervix<br>5 petct_find___5 FDG-avid uptake in vagina<br>6 petct_find___6 FDG-avid uptake in parametrium<br>7 petct_find___7 FDG-avid uptake in pelvic side wall<br>8 petct_find___8 FDG-avid uptake in pelvic lymph nodes<br>9 petct_find___9 FDG-avid uptake in inguinal lymph nodes<br>10 petct_find___10 FDG-avid uptake in para-aortic nodes<br>11 petct_find___11 FDG-avid uptake in x1 ureter<br>12 petct_find___12 FDG-avid uptake in x2 ureters<br>13 petct_find___13 FDG-avid uptake in x1 kidney<br>14 petct_find___14 FDG-avid uptake in x2 kidneys<br>15 petct_find___15 FDG-avid uptake in bladder<br>16 petct_find___16 FDG-avid uptake in lungs<br>17 petct_find___17 FDG-avid uptake in rectum<br>18 petct_find___18 FDG-avid uptake in sigmoid<br>19 petct_find___19 FDG-avid uptake in rectal nodules<br>20 petct_find___20 FDG-avid uptake in sigmoid nodules<br>21 petct_find___21 FDG-avid uptake peritoneal nodules<br>22 petct_find___22 FDG-avid uptake omentum<br>23 petct_find___23 Other
744	[ps_petct_find]<br>Show the field ONLY if:<br>[petct_find(23)] = '1'	Please state	text
745	[petct_no_ec]<br>Show the field ONLY if:<br>[petct_ec] = '0'	Reason PET-CT not performed at diagnosis (select all that apply)	checkbox<br>1 petct_no_ec___1 No access to PET machine<br>2 petct_no_ec___2 Patient unable to afford<br>3 petct_no_ec___3 Patient refused<br>4 petct_no_ec___4 Uncontrolled diabetes mellitus<br>5 petct_no_ec___5 Other imaging performed<br>6 petct_no_ec___6 Not clinical protocol<br>7 petct_no_ec___7 Other
746	[ps_petct_no_ec]<br>Show the field ONLY if:<br>[petct_no_ec(7)] = '1'	Please state	text

747	[figo_ec]	Clinical FIGO Stage (i.e. preoperative staging taking into account imaging, clinical examination)	dropdown<br>1 1A Limited to endometrium or involves less than half of the myometrium (ENDOMETRIUM/CARCINOSARCOMA)<br>2 IB Invasion of half or more of the myometrium (ENDOMETRIUM/CARCINOSARCOMA)<br>3 II Invasion of the cervical stroma but no extension outside the uterus (ENDOMETRIUM/CARCINOSARCOMA)<br>4 IIIA Invasion of serosa, adnexa, or both (direct extension or metastasis) (ENDOMETRIUM/CARCINOSARCOMA)<br>5 IIIB Metastases or direct spread to the vagina and/or spread to the parametria (ENDOMETRIUM/CARCINOSARCOMA)<br>6 IIIC1 Metastases to pelvic lymph nodes (ENDOMETRIUM/CARCINOSARCOMA)<br>7 IIIC2 Metastases to para-aortic lymph nodes, with or without metastases to pelvic lymph nodes (ENDOMETRIUM/CARCINOSARCOMA)<br>8 IVA Invasion of the bladder, intestinal mucosa, or both (ENDOMETRIUM/CARCINOSARCOMA)<br>9 IVB Distant metastases, including metastases to the inguinal lymph nodes, lungs, liver, or bone and intraperitoneal disease (ENDOMETRIUM/CARCINOSARCOMA)<br>10 IA Tumour limited to uterus < 5cm (LEIOMYOSARCOMA)<br>11 IB Tumour limited to uterus >5cm (LEIOMYOSARCOMA)<br>12 IIA Adnexal involvement (LEIOMYOSARCOMA)<br>13 IIB Tumour extends to extra-uterine pelvic tissue (LEIOMYOSARCOMA)<br>14 IIIA Tumour invades abdominal tissues, 1 site (LEIOMYOSARCOMA)<br>15 IIIB Tumour invades abdominal tissues, > 1 site (LEIOMYOSARCOMA)<br>16 IIIC Metastasis to pelvic and/or para-aortic lymph nodes (LEIOMYOSARCOMA)<br>17 IVA Tumour invades bladder and/or rectum (LEIOMYOSARCOMA)<br>18 IVB Distant metastasis (LEIOMYOSARCOMA)<br>19 IA Tumour limited to endometrium/endocervix with no myometrial invasion (ENDOMETRIAL STROMAL SARCOMA/ADENOSARCOMA)<br>20 IB Less than or equal to half myometrial invasion (ENDOMETRIAL STROMAL SARCOMA/ADENOSARCOMA)<br>21 IC More than half myometrial invasion (ENDOMETRIAL STROMAL SARCOMA/ADENOSARCOMA)<br>22 IIA Adnexal involvement (ENDOMETRIAL STROMAL SARCOMA/ADENOSARCOMA)<br>23 IIB Tumour extends to extrauterine pelvic tissue (ENDOMETRIAL STROMAL SARCOMA/ADENOSARCOMA)<br>24 IIIA Tumour invades abdominal tissues, 1 site (ENDOMETRIAL STROMAL SARCOMA/ADENOSARCOMA)<br>25 IIIB Tumour invades abdominal tissues, > 1 site (ENDOMETRIAL STROMAL SARCOMA/ADENOSARCOMA)<br>26 IIIC Metastasis to pelvic and/or para-aortic lymph nodes (ENDOMETRIAL STROMAL SARCOMA/ADENOSARCOMA)

			27 IVA Tumour invades bladder and/or rectum (ENDOMETRIAL STROMAL SARCOMA/ADENOSARCOMA)<br>28 IVB Distant metastasis (ENDOMETRIAL STROMAL SARCOMA/ADENOSARCOMA)
748	[nact_ec]	Neoadjuvant (before surgery) chemotherapy	yesno<br>1 Yes<br>0 No
749	[first_chem_ec]<br>Show the field ONLY if:<br>[nact_ec] = '1'	Chemotherapy regimen FIRST line (select all that apply)	checkbox<br>1 first_chem_ec___1 Platinum (for example cisplatin, carboplatin)<br>2 first_chem_ec___2 Taxane (for example paclitaxel)<br>3 first_chem_ec___3 Bevacuzimab<br>4 first_chem_ec___4 Doxorubicin<br>5 first_chem_ec___5 Clinical trial regimens<br>6 first_chem_ec___6 Aromatase inhibitors (for example anastrazole/letrozole)<br>7 first_chem_ec___7 Tamoxifen<br>8 first_chem_ec___8 Other
750	[ps_first_chem_ec]<br>Show the field ONLY if:<br>[first_chem_ec(8)] = '1'	Please state	text
751	[nact_numb_ec]<br>Show the field ONLY if:<br>[nact_ec] = '1'	Total number of cycles received	text
752	[nact_no_ec]<br>Show the field ONLY if:<br>[nact_ec] = '0'	Reason neoadjuvant chemotherapy not administered (select all that apply)	checkbox<br>1 nact_no_ec___1 Poor performance status<br>2 nact_no_ec___2 Bowel obstruction<br>3 nact_no_ec___3 No access<br>4 nact_no_ec___4 Patient unable to afford<br>5 nact_no_ec___5 Patient refused<br>6 nact_no_ec___6 Not clinically indicated - upfront surgery<br>7 nact_no_ec___7 Other
753	[ps_nact_no_ec]<br>Show the field ONLY if:<br>[nact_no_ec(7)] = '1'	Please state	text
754	[cov_ec]	COVID-19 status	dropdown<br>1 Positive<br>2 Negative<br>3 Not tested
755	[cov_no_ec]<br>Show the field ONLY if:<br>[cov_ec] = '3'	If not tested select reason (select all that apply)	checkbox<br>1 cov_no_ec___1 Not clinically indicated (asymptomatic)<br>2 cov_no_ec___2 Not clinical protocol to routinely test all patients prior to surgery<br>3 cov_no_ec___3 Patient refused<br>4 cov_no_ec___4 Patient unable to afford test<br>6 cov_no_ec___6 Testing not available locally<br>5 cov_no_ec___5 Other
756	[ps_cov_no_ec]<br>Show the field ONLY if:<br>[cov_no_ec(5)] = '1'	Please state	text

757	[bp_ec]	Bowel preparation pre-operatively	yesno<br>1 Yes<br>0 No
758	[peri_op_ec]	Peri-operative management plan in place? (Non- exhaustive list: pre-operative haemoglobin optimisation and iron deficit correction; correction of denutrition and immunonutrition; fluid management; pain management; prevention of post-operative nausea and vomiting).	yesno<br>1 Yes<br>0 No
759	[hb_ec]	Pre-operative Haemoglobin (g/L)	text (number)
760	[wcc_ec]	Pre-operative White Cell Count (10^9/L)	text (number)
761	[plt_ec]	Pre-operative Platelets (10^9/L)	text (number)
762	[pt_ec]	Pre-operative PT (Secs)	text (number)
763	[inr_ec]	Pre-operative INR (ratio)	text (number)
764	[aptt_ec]	Pre-operative APTT (Secs)	text (number)
765	[fib_ec]	Pre-operative Fibrinogen (g/L)	text (number)
766	[alb_ec]	Pre-operative Albumin (g/L)	text (number)
767	[na_ec]	Pre-operative Sodium (mmol/L)	text (number)
768	[k_ec]	Pre-operative Potassium (mmol/L)	text (number)
769	[ur_ec]	Pre-operative Urea (mmol/L)	text (number)
770	[creat_ec]	Pre-operative Creatinine (μmol/L)	text (number)
771	[egfr_ec]	Pre-operative eGFR - estimated Glomerular Filtration Rate (mL/min)	text
772	[bili_ec]	Pre-operative Bilirubin total (μmol/L)	text (number)
773	[alt_ec]	Pre-operative Alanine Aminotransaminase (IU/L)	text (number)
774	[alp_ec]	Pre-operative Alkaline Phosphatase (IU/L)	text (number)
775	[ast_ec]	Pre-operative Aspartate Aminotransaminase (IU/L)	text (number)
776	[crp_ec]	Pre-operative CRP - C-reactive protein (mg/L)	text (number)
777	[datsurg_ec]	Section Header: *SURGERY*<br>Date of surgery	text (date_dmy)
778	[anaes_ec]	Anaesthesia (select all that apply)	checkbox<br>1 anaes_ec___1 General<br>2 anaes_ec___2 Epidural<br>3 anaes_ec___3 Spinal
779	[elem_ec]	Was the surgery an elective or emergency case?	dropdown<br>1 Elective<br>2 Emergency
780	[sm_ec]	Surgical modality	dropdown<br>1 Laparotomy<br>2 Laparoscopic<br>3 Robotic<br>4 Vaginal<br>5 Laparoscopic converted to laparotomy<br>6 Robotic converted to laparotomy<br>7 Vaginal converted to laparotomy
781	[si_ec]	Surgical aim	dropdown<br>1 Curative<br>2 Curative attempted but then abandoned (i.e. open/close laparotomy)<br>3 Palliative

782	[lead_surg_ec]	Lead surgeon performing surgery	dropdown<br>1 Certified consultant/specialist gynaecological oncologist<br>2 Fellow/trainee gynaecological oncologist<br>3 Trained surgeon dedicated to the management of gynaecologic cancer or having completed an ESGO accredited fellowship.<br>4 General surgeon<br>5 Surgical oncologist<br>6 Other
783	[ps_lead_surg_ec]<br>Show the field ONLY if:<br>[lead_surg_ec] = '6'	Please state	text
784	[who_ec]	WHO surgical safety checklist used	yesno<br>1 Yes<br>0 No
785	[cyto_ec1]	Complete macroscopic cytoreduction at surgery	yesno<br>1 Yes<br>0 No
786	[cytored_no_ec]<br>Show the field ONLY if:<br>[cyto_ec1] = '0'	Reason(s) complete cytoreduction not achieved (select all that apply)	checkbox<br>1 cytored_no_ec___1 Disease more extensive than that shown on imaging<br>2 cytored_no_ec___2 Technically unable to resect<br>3 cytored_no_ec___3 Patient became medically unstable intra-operatively and decision made to not proceed further with surgery<br>4 cytored_no_ec___4 Other
787	[ps_cytoreduc_no_ec]<br>Show the field ONLY if:<br>[cytored_no_ec(4)] = '1'	Please state	text
788	[siz_resid_mm_ec]<br>Show the field ONLY if:<br>[cyto_ec1] = '0'	Size of residual disease (in millimetres)	dropdown<br>1 1-2.5<br>2 2.6-5<br>3 5.1-7.5<br>4 7.6-10<br>5 >10

789	[loc_resid_dis_ec]<br>Show the field ONLY if:<br>[cyto_ec1] = '0'	Location of residual disease (select all that apply)	checkbox<br>1 loc_resid_dis_ec___1 uterus<br>2 loc_resid_dis_ec___2 fallopian tube(s)<br>3 loc_resid_dis_ec___3 ovary(s)<br>4 loc_resid_dis_ec___4 cervix<br>5 loc_resid_dis_ec___5 vagina<br>6 loc_resid_dis_ec___6 parametrium<br>7 loc_resid_dis_ec___7 pelvic side wall<br>8 loc_resid_dis_ec___8 pelvic lymph nodes<br>9 loc_resid_dis_ec___9 inguinal lymph nodes<br>10 loc_resid_dis_ec___10 para-aortic nodes<br>11 loc_resid_dis_ec___11 ureter(s)<br>12 loc_resid_dis_ec___12 kidney(s)<br>13 loc_resid_dis_ec___13 bladder<br>14 loc_resid_dis_ec___14 rectum<br>15 loc_resid_dis_ec___15 sigmoid<br>16 loc_resid_dis_ec___16 rectal nodules<br>17 loc_resid_dis_ec___17 sigmoid nodules<br>18 loc_resid_dis_ec___18 peritoneal nodules<br>19 loc_resid_dis_ec___19 omentum<br>20 loc_resid_dis_ec___20 Other
790	[ps_loc_resid_dis_ec]<br>Show the field ONLY if:<br>[loc_resid_dis_ec(20)] = '1'	Please state	text
791	[surgfind_ec]	Findings at surgery (select all that apply)	checkbox<br>1 surgfind_ec___1 Uterus invasion<br>2 surgfind_ec___2 Cervical invasion<br>3 surgfind_ec___3 x1 tubo-ovarian invasion<br>4 surgfind_ec___4 x2 tubo-ovarian invasion<br>5 surgfind_ec___5 Parametrial invasion<br>6 surgfind_ec___6 Pelvic sidewall invasion<br>7 surgfind_ec___7 Pelvic lymphadenopathy<br>8 surgfind_ec___8 Inguinal lymphadenopathy<br>9 surgfind_ec___9 Para-aortic lymphadenopathy<br>10 surgfind_ec___10 Bladder invasion<br>11 surgfind_ec___11 x1 hydroureter<br>12 surgfind_ec___12 x2 hydroureter<br>13 surgfind_ec___13 Ureteric invasion<br>14 surgfind_ec___14 x1 hydronephrosis<br>15 surgfind_ec___15 x2 hydronephrosis<br>16 surgfind_ec___16 Para rectal nodules<br>17 surgfind_ec___17 Sigmoid nodules<br>18 surgfind_ec___18 Rectal and sigmoid diffuse serosal invasion<br>19 surgfind_ec___19 Rectal and sigmoid diffuse wall invasion<br>20 surgfind_ec___20 Rectal and sigmoid diffuse luminal invasion<br>21 surgfind_ec___21 Peritoneal nodules<br>22 surgfind_ec___22 Omental disease<br>23 surgfind_ec___23 Ascites<br>24 surgfind_ec___24 Other<br>25 surgfind_ec___25 No visible disease

792	[ps_surgfind_ec]<br>Show the field ONLY if:<br>[surgfind_ec(24)] = '1'	Please state	text
793	[tr_ec]	Tissue removed (select all that apply)	checkbox<br>1 | tr_ec___1 | Uterus<br>2 | tr_ec___2 | Cervix<br>3 | tr_ec___3 | x1 fallopian tube<br>4 | tr_ec___4 | x2 fallopian tubes<br>5 | tr_ec___5 | x1 ovary<br>6 | tr_ec___6 | x2 ovaries<br>7 | tr_ec___7 | Upper vagina<br>8 | tr_ec___8 | Parametrium<br>9 | tr_ec___9 | Pelvic lymph nodes<br>10 | tr_ec___10 | Para-aortic lymph nodes<br>11 | tr_ec___11 | Inguinal lymph nodes<br>12 | tr_ec___12 | Rectum<br>13 | tr_ec___13 | Sigmoid<br>14 | tr_ec___14 | Bladder<br>15 | tr_ec___15 | Ureter<br>16 | tr_ec___16 | Kidney<br>17 | tr_ec___17 | Peritoneum<br>18 | tr_ec___18 | Omentum<br>19 | tr_ec___19 | Other
794	[paln_dis_ec]<br>Show the field ONLY if:<br>[tr_ec(10)] = '1'	Select all that apply	checkbox<br>1 | paln_dis_ec___1 | Infra-renal<br>2 | paln_dis_ec___2 | Supra-renal
795	[ps_tr_ec]<br>Show the field ONLY if:<br>[tr_ec(19)] = '1'	Please state	text
796	[sln_yn_ec]	Sentinel lymph nodes mapping	yesno<br>1 | Yes<br>0 | No
797	[sln_no_ec]<br>Show the field ONLY if:<br>[sln_yn_ec] = '0'	Reason not done (select all that apply)	checkbox<br>1 | sln_no_ec___1 | No facility<br>2 | sln_no_ec___2 | Not local protocol<br>3 | sln_no_ec___3 | Clinicians not trained<br>4 | sln_no_ec___4 | Patient refused<br>5 | sln_no_ec___5 | Patient unable to afford<br>6 | sln_no_ec___6 | Other
798	[ps_sln_no_ec]<br>Show the field ONLY if:<br>[sln_no_ec(6)] = '1'	Please state	text
799	[inj_sln_ec]<br>Show the field ONLY if:<br>[sln_yn_ec] = '1'	Site of injection (select all that apply)	checkbox<br>1 | inj_sln_ec___1 | Cervix<br>2 | inj_sln_ec___2 | Hysteroscopy<br>3 | inj_sln_ec___3 | Fundus<br>4 | inj_sln_ec___4 | Other
800	[ps_inj_sln_ec]<br>Show the field ONLY if:<br>[inj_sln_ec(4)] = '1'	Please state	text

801	[trcr_sln_ec]<br>Show the field ONLY if:<br>[sln_yn_ec] = '1'	Tracer	dropdown<br>1 Indocyanine green<br>2 Methylene blue<br>3 Technetium<br>4 Other
802	[ps_trcr_sln_ec]<br>Show the field ONLY if:<br>[trcr_sln_ec] = '4'	Please state	text
803	[sitmap_sln_ec]<br>Show the field ONLY if:<br>[sln_yn_ec] = '1'	Site of mapping (select all that apply)	checkbox<br>1 sitmap_sln_ec___1 right pelvic lymph node<br>2 sitmap_sln_ec___2 left pelvic lymph node<br>3 sitmap_sln_ec___3 para-aortic<br>4 sitmap_sln_ec___4 presacral
804	[r_pln_ec]<br>Show the field ONLY if:<br>[sitmap_sln_ec(1)] = '1'	Results	dropdown<br>1 Positive<br>2 Negative
805	[l_pln_ec]<br>Show the field ONLY if:<br>[sitmap_sln_ec(2)] = '1'	Results	dropdown<br>1 Positive<br>2 Negative
806	[paln_ec]<br>Show the field ONLY if:<br>[sitmap_sln_ec(3)] = '1'	Results	dropdown<br>1 Positive<br>2 Negative
807	[psln_ec]<br>Show the field ONLY if:<br>[sitmap_sln_ec(4)] = '1'	Results	dropdown<br>1 Positive<br>2 Negative
808	[si_stom_ec]	Small bowel stoma	yesno<br>1 Yes<br>0 No
809	[ss_ec]<br>Show the field ONLY if:<br>[si_stom_ec] = '1'	Site of stoma	dropdown<br>1 Duodenum<br>2 Jejunum<br>3 Ileum
810	[pb_si_stom_ec]<br>Show the field ONLY if:<br>[si_stom_ec] = '1'	Performed by (select all that apply)	checkbox<br>1 pb_si_stom_ec___1 Gynaecological oncologist<br>2 pb_si_stom_ec___2 Colorectal surgeon<br>3 pb_si_stom_ec___3 Urologist<br>4 pb_si_stom_ec___4 General surgeon<br>5 pb_si_stom_ec___5 Other
811	[ps_pb_si_stom_ec]<br>Show the field ONLY if:<br>[pb_si_stom_ec(5)] = '1'	Please state	text
812	[si_anast_ec]	Small bowel anastamosis	yesno<br>1 Yes<br>0 No
813	[pb_si_anast_ec]<br>Show the field ONLY if:<br>[si_anast_ec] = '1'	Performed by (select all that apply)	checkbox<br>1 pb_si_anast_ec___1 Gynaecological oncologist<br>2 pb_si_anast_ec___2 Colorectal surgeon<br>3 pb_si_anast_ec___3 Urologist<br>4 pb_si_anast_ec___4 General surgeon<br>5 pb_si_anast_ec___5 Other
814	[ps_pb_si_anast_ec]<br>Show the field ONLY if:<br>[pb_si_anast_ec(5)] = '1'	Please state	text

815	[listom_ec]	Large bowel stoma	yesno<br>1 Yes<br>0 No
816	[pb_listom_ec]<br>Show the field ONLY if:<br>[listom_ec] = '1'	Performed by (please state)	checkbox<br>1 pb_listom_ec___1 Gynaecological oncologist<br>2 pb_listom_ec___2 Colorectal surgeon<br>3 pb_listom_ec___3 Urologist<br>4 pb_listom_ec___4 General surgeon<br>5 pb_listom_ec___5 Other
817	[ps_pb_listom_ec]<br>Show the field ONLY if:<br>[pb_listom_ec(5)] = '1'	Please state	text
818	[lianas_ec]	Large bowel anastamosis	yesno<br>1 Yes<br>0 No
819	[pb_lianas_ec]<br>Show the field ONLY if:<br>[lianas_ec] = '1'	Performed by (select all that apply)	checkbox<br>1 pb_lianas_ec___1 Gynaecological oncologist<br>2 pb_lianas_ec___2 Colorectal surgeon<br>3 pb_lianas_ec___3 Urologist<br>4 pb_lianas_ec___4 General surgeon<br>5 pb_lianas_ec___5 Other
820	[ps_pb_lianas_ec]<br>Show the field ONLY if:<br>[pb_lianas_ec(5)] = '1'	Please state	text
821	[uro_ec]	Urostomy/ileal conduit	yesno<br>1 Yes<br>0 No
822	[pb_uro_ec]<br>Show the field ONLY if:<br>[uro_ec] = '1'	Performed by (select all that apply)	checkbox<br>1 pb_uro_ec___1 Gynaecological oncologist<br>2 pb_uro_ec___2 Colorectal surgeon<br>3 pb_uro_ec___3 Urologist<br>4 pb_uro_ec___4 General surgeon<br>5 pb_uro_ec___5 Other
823	[ps_pb_uro_ec]<br>Show the field ONLY if:<br>[pb_uro_ec(5)] = '1'	Please state	text
824	[tuu_ec]	Trans uretero-ureteric anastamosis	yesno<br>1 Yes<br>0 No
825	[pb_tuu_ec]<br>Show the field ONLY if:<br>[tuu_ec] = '1'	Performed by (select all that apply)	checkbox<br>1 pb_tuu_ec___1 Gynaecological oncologist<br>2 pb_tuu_ec___2 Colorectal surgeon<br>3 pb_tuu_ec___3 Urologist<br>4 pb_tuu_ec___4 General surgeon<br>5 pb_tuu_ec___5 Other
826	[ps_pb_tuu_ec]<br>Show the field ONLY if:<br>[pb_tuu_ec(5)] = '1'	Please state	text
827	[ui_ec]	Ureteric reimplantation	yesno<br>1 Yes<br>0 No

828	[pb_ui_ec]<br>Show the field ONLY if:<br>[ui_ec] = '1'	Performed by (select all that apply)	checkbox<br>1 pb_ui_ec___1 Gynaecological oncologist<br>2 pb_ui_ec___2 Colorectal surgeon<br>3 pb_ui_ec___3 Urologist<br>4 pb_ui_ec___4 General surgeon<br>5 pb_ui_ec___5 Other
829	[ps_pb_ui_ec]<br>Show the field ONLY if:<br>[pb_ui_ec(5)] = '1'	Please state	text
830	[neph_ec]	Nephrostomy	yesno<br>1 Yes<br>0 No
831	[aliedsurg_ec]<br>Show the field ONLY if:<br>[neph_ec] = '1'	Performed by (select all that apply)	checkbox<br>1 aliedsurg_ec___1 Gynaecological oncologist<br>2 aliedsurg_ec___2 Colorectal surgeon<br>3 aliedsurg_ec___3 Urologist<br>4 aliedsurg_ec___4 General surgeon<br>5 aliedsurg_ec___5 Other
832	[ps_alied_surg_ec]<br>Show the field ONLY if:<br>[aliedsurg_ec(5)] = '1'	Please state	text
833	[abx_ec]	Intraoperative antibiotics	yesno<br>1 Yes<br>0 No
834	[tim_ec]	Length of time of surgery	text (time)
835	[itu_ec]	Section Header: *POST-OPERATIVE*<br>Post-operative recovery in intensive care unit	yesno<br>1 Yes<br>0 No
836	[dys_itu_ec]<br>Show the field ONLY if:<br>[itu_ec] = '1'	Number of days in intensive care unit	text (number)
837	[hdu_ec]	Post-operative recovery in high dependency unit	yesno<br>1 Yes<br>0 No
838	[dys_hdu_ec]<br>Show the field ONLY if:<br>[hdu_ec] = '1'	Number of days in high dependency unit	text (number)
839	[er_ec]	Enhanced recovery?	yesno<br>1 Yes<br>0 No
840	[po_abx_ec]	Prophylactic post operative antibiotics	yesno<br>1 Yes<br>0 No
841	[drn_ec]	Post operative surgical drain	yesno<br>1 Yes<br>0 No
842	[drn_dys_ec]<br>Show the field ONLY if:<br>[drn_ec] = '1'	Number of days in situ	text (number)
843	[uc_ec]	Post operative urinary catheter	yesno<br>1 Yes<br>0 No
844	[cath_dys_ec]<br>Show the field ONLY if:<br>[uc_ec] = '1'	Number of days in situ	text (number)

845	[phb_ec]	Post-operative Haemoglobin (g/L) - please state first post-operative result	text (number)
846	[pwbc_ec]	Post-operative White Cell Count (10^9/L) - please state first post-operative result	text (number)
847	[pplat_ec]	Post-operative Platelets (10^9/L) - please state first post-operative result	text (number)
848	[ppt_ec]	Post-operative PT (Secs) - please state first post-operative result	text (number)
849	[pinr_ec]	Post-operative INR (Ratio) - please state first post-operative result	text (number)
850	[paptt_ec]	Post-operative APTT (Secs) - please state first post-operative result	text (number)
851	[pfib_ec]	Post-operative Fibrinogen (g/L) - please state first post-operative result	text (number)
852	[palb_ec]	Post-operative Albumin (g/L) - please state first post-operative result	text (number)
853	[pna_ec]	Post-operative Sodium (mmol/L) - please state first post-operative result	text (number)
854	[pk_ec]	Post-operative Potassium (mmol/L) - please state first post-operative result	text (number)
855	[purea_ec]	Post-operative Urea (mmol/L) - please state first post-operative result	text (number)
856	[pcreat_ec]	Post-operative Creatinine (μmol/L) - please state first post-operative result	text
857	[pegfr_ec]	Post-operative eGFR - Estimated Glomerular Filtration Rate (mL/min) - please state first post-operative result	text
858	[pbili_ec]	Post-operative Bilirubin total (μmol/L) - please state first post-operative result	text (number)
859	[paat_ec]	Post-operative Alanine Aminotransaminase (IU/L) - please state first post-operative result	text (number)
860	[palp_ec]	Post-operative Alkaline Phosphatase (IU/L) - please state first post-operative result	text (number)
861	[past_ec]	Post-operative Aspartate Aminotransaminase (IU/L) - please state first post-operative result	text (number)
862	[pcrp_ec]	Post-operative CRP - C-reactive protein (mg/L) - please state first post-operative result	text (number)
863	[hist_ec]	Histologic type	dropdown<br>1 Endometrioid<br>2 Mucinous<br>3 Serous<br>4 Clear cell<br>5 Squamous cell<br>6 Undifferentiated<br>7 Mixed<br>8 Carcinosarcoma<br>9 Leiomyosarcoma<br>10 Benign
864	[resec_ec]	Histological confirmed resection margins	dropdown<br>1 < 1 mm / tumour on margin<br>2 1-5 mm (NO tumour on margin)<br>3 >5 mm Margins confirmed clear<br>4 Unknown, not available in this hospital<br>5 Not applicable - benign
865	[grade_ec]<br>Show the field ONLY if:<br>[hist_ec] = '1'	Tumour grade	dropdown<br>1 I<br>2 II<br>3 III<br>4 Not applicable - benign

866	[lvsi_ec]	Lymphovascular space invasion (LVSI)	dropdown<br>1 No<br>2 Focal<br>3 Substantial<br>4 Not applicable - benign
867	[molec_ec]	Molecular classification	dropdown<br>1 ultramutated/with pathogenic POLE mutations<br>2 hypermutated with MSI/MMRd (loss of MMR protein immunoreactivity)<br>3 high copy number/p53abn (p53 mutant immunoreactive pattern)<br>4 low copy number/NSMP (retained MMR protein immunoreactivity, and p53 wild-type immunoreactive pattern)<br>5 multiple classifier (any combination of markers included in the previous categories)<br>6 not classified<br>7 Not applicable - benign
868	[mlh1]	Section Header: *Immunohistochemistry*<br>MLH1	radio (Matrix)<br>1 Positive<br>2 Negative<br>3 Not tested
869	[msh2]	MSH2	radio (Matrix)<br>1 Positive<br>2 Negative<br>3 Not tested
870	[msh6]	MSH6	radio (Matrix)<br>1 Positive<br>2 Negative<br>3 Not tested
871	[pms2]	PMS2	radio (Matrix)<br>1 Positive<br>2 Negative<br>3 Not tested
872	[pten]	PTEN	radio (Matrix)<br>1 Positive<br>2 Negative<br>3 Not tested
873	[er_ihc]	ER	radio (Matrix)<br>1 Positive<br>2 Negative<br>3 Not tested
874	[pr_ihc]	PR	radio (Matrix)<br>1 Positive<br>2 Negative<br>3 Not tested
875	[p53_ihc]	p53	radio (Matrix)<br>1 Positive<br>2 Negative<br>3 Not tested
876	[p16_ihc]	p16	radio (Matrix)<br>1 Positive<br>2 Negative<br>3 Not tested

877	[her2]	HER2	radio (Matrix)<br>1 Positive<br>2 Negative<br>3 Not tested
878	[hmga2]	HMGA2	radio (Matrix)<br>1 Positive<br>2 Negative<br>3 Not tested
879	[cea_ihc]	CEA	radio (Matrix)<br>1 Positive<br>2 Negative<br>3 Not tested
880	[vim_ihc]	Vimentin	radio (Matrix)<br>1 Positive<br>2 Negative<br>3 Not tested
881	[bcat_ihc]	β-catenin	radio (Matrix)<br>1 Positive<br>2 Negative<br>3 Not tested
882	[arid1a_ihc]	ARID1A	radio (Matrix)<br>1 Positive<br>2 Negative<br>3 Not tested
883	[pax2_ihc]	PAX2	radio (Matrix)<br>1 Positive<br>2 Negative<br>3 Not tested
884	[wt1_ihc]	WT-1	radio (Matrix)<br>1 Positive<br>2 Negative<br>3 Not tested

885	[figo_ec_2]	Final surgical FIGO stage (i.e. postoperative staging taking into account surgical and pathology findings)	dropdown

1	1A Limited to endometrium or involves less than half of the myometrium (ENDOMETRIUM/CARCINOSARCOMA)
2	IB Invasion of half or more of the myometrium (ENDOMETRIUM/CARCINOSARCOMA)
3	II Invasion of the cervical stroma but no extension outside the uterus (ENDOMETRIUM/CARCINOSARCOMA)
4	IIIA Invasion of serosa, adnexa, or both (direct extension or metastasis) (ENDOMETRIUM/CARCINOSARCOMA)
5	IIIB Metastases or direct spread to the vagina and/or spread to the parametria (ENDOMETRIUM/CARCINOSARCOMA)
6	IIIC1 Metastases to pelvic lymph nodes (ENDOMETRIUM/CARCINOSARCOMA)
7	IIIC2 Metastases to para-aortic lymph nodes, with or without metastases to pelvic lymph nodes (ENDOMETRIUM/CARCINOSARCOMA)
8	IVA Invasion of the bladder, intestinal mucosa, or both (ENDOMETRIUM/CARCINOSARCOMA)
9	IVB Distant metastases, including metastases to the inguinal lymph nodes, lungs, liver, or bone and intraperitoneal disease (ENDOMETRIUM/CARCINOSARCOMA)
10	IA Tumour limited to uterus < 5cm (LEIOMYOSARCOMA)
11	IB Tumour limited to uterus >5cm (LEIOMYOSARCOMA)
12	IIA Adnexal involvement (LEIOMYOSARCOMA)
13	IIB Tumour extends to extra-uterine pelvic tissue (LEIOMYOSARCOMA)
14	IIIA Tumour invades abdominal tissues, 1 site (LEIOMYOSARCOMA)
15	IIIB Tumour invades abdominal tissues, > 1 site (LEIOMYOSARCOMA)
16	IIIC Metastasis to pelvic and/or para-aortic lymph nodes (LEIOMYOSARCOMA)
17	IVA Tumour invades bladder and/or rectum (LEIOMYOSARCOMA)
18	IVB Distant metastasis (LEIOMYOSARCOMA)
19	IA Tumour limited to endometrium/endocervix with no myometrial invasion (ENDOMETRIAL STROMAL SARCOMA/ADENOSARCOMA)
20	IB Less than or equal to half myometrial invasion (ENDOMETRIAL STROMAL SARCOMA/ADENOSARCOMA)
21	IC More than half myometrial invasion (ENDOMETRIAL STROMAL SARCOMA/ADENOSARCOMA)
22	IIA Adnexal involvement (ENDOMETRIAL STROMAL SARCOMA/ADENOSARCOMA)
23	IIB Tumour extends to extrauterine pelvic tissue (ENDOMETRIAL STROMAL SARCOMA/ADENOSARCOMA)
24	IIIA Tumour invades abdominal tissues, 1 site (ENDOMETRIAL STROMAL SARCOMA/ADENOSARCOMA)
25	IIIB Tumour invades abdominal tissues, > 1 site (ENDOMETRIAL STROMAL SARCOMA/ADENOSARCOMA)
26	IIIC Metastasis to pelvic and/or para-aortic lymph nodes (ENDOMETRIAL STROMAL SARCOMA/ADENOSARCOMA)

			27 IVA Tumour invades bladder and/or rectum (ENDOMETRIAL STROMAL SARCOMA/ADENOSARCOMA)<br>28 IVB Distant metastasis (ENDOMETRIAL STROMAL SARCOMA/ADENOSARCOMA)<br>29 Not applicable - benign
886	[gt_ec]	Genetic testing performed?	yesno<br>1 Yes<br>0 No
887	[brca1_ec]<br>Show the field ONLY if:<br>[gt_ec] = '1'	Section Header: *Genetic testing*<br>BRCA1	radio (Matrix)<br>1 Positive<br>2 Negative<br>3 Not tested
888	[brca2_ec]<br>Show the field ONLY if:<br>[gt_ec] = '1'	BRCA2	radio (Matrix)<br>1 Positive<br>2 Negative<br>3 Not tested
889	[mlh1_ec]<br>Show the field ONLY if:<br>[gt_ec] = '1'	MLH1	radio (Matrix)<br>1 Positive<br>2 Negative<br>3 Not tested
890	[msh2_ec]<br>Show the field ONLY if:<br>[gt_ec] = '1'	MSH2	radio (Matrix)<br>1 Positive<br>2 Negative<br>3 Not tested
891	[msh6_ec]<br>Show the field ONLY if:<br>[gt_ec] = '1'	MSH6	radio (Matrix)<br>1 Positive<br>2 Negative<br>3 Not tested
892	[pms2_ec]<br>Show the field ONLY if:<br>[gt_ec] = '1'	PMS2	radio (Matrix)<br>1 Positive<br>2 Negative<br>3 Not tested
893	[no_gt_reas_ec]<br>Show the field ONLY if:<br>[gt_ec] = '0'	Reason genetic testing not performed (select all that apply)	checkbox<br>1 no_gt_reas_ec___1 Not available<br>5 no_gt_reas_ec___5 Does not meet clinical criteria for genetic testing<br>2 no_gt_reas_ec___2 Patient unable to afford<br>4 no_gt_reas_ec___4 Patient refused<br>3 no_gt_reas_ec___3 Other
894	[ps_no_gt_reas_ec]<br>Show the field ONLY if:<br>[no_gt_reas_ec(3)] = '1'	Please state	text
895	[immuno_ec]	Immunotherapy	yesno<br>1 Yes<br>0 No
896	[adjct_ec]	Adjuvant (after surgery) chemotherapy	yesno<br>1 Yes<br>0 No

897	[chem1_ec]<br>Show the field ONLY if:<br>[adjct_ec] = '1'	Chemotherapy regimen FIRST line (Select all that apply)	checkbox<br>1 chem1_ec___1 Platinum (for example cisplatin, carboplatin)<br>2 chem1_ec___2 Taxane (for example paclitaxel)<br>3 chem1_ec___3 Bevacuzimab<br>4 chem1_ec___4 Doxorubicin<br>5 chem1_ec___5 Clinical trial regimens<br>6 chem1_ec___6 Aromatase inhibitors (for example anastrazole/letrozole)<br>7 chem1_ec___7 Tamoxifen<br>8 chem1_ec___8 Other
898	[ps_chem1_ec]<br>Show the field ONLY if:<br>[chem1_ec(8)] = '1'	Please state	text
899	[chem_numb_ec]<br>Show the field ONLY if:<br>[adjct_ec] = '1'	Number of cycles planned	text (number)
900	[no_adjct_ec]<br>Show the field ONLY if:<br>[adjct_ec] = '0'	Reason adjuvant chemotherapy not administered (select all that apply)	checkbox<br>1 no_adjct_ec___1 Poor performance status<br>2 no_adjct_ec___2 No access<br>3 no_adjct_ec___3 Patient unable to afford<br>4 no_adjct_ec___4 Patient refused<br>5 no_adjct_ec___5 Not clinically indicated<br>6 no_adjct_ec___6 Other
901	[ps_no_adj_ec]<br>Show the field ONLY if:<br>[no_adjct_ec(6)] = '1'	Please state	text
902	[rt_ec]	External beam radiotherapy administered	yesno<br>1 Yes<br>0 No
903	[rt_y_ec]<br>Show the field ONLY if:<br>[rt_ec] = '1'	Administered to (select all that apply)	checkbox<br>1 rt_y_ec___1 pelvis<br>2 rt_y_ec___2 para-aortic strip<br>3 rt_y_ec___3 pelvis and para-aortic strip<br>4 rt_y_ec___4 other
904	[ps_rt_y_ec]<br>Show the field ONLY if:<br>[rt_y_ec(4)] = '1'	Please state	text
905	[rt_dos_ec]<br>Show the field ONLY if:<br>[rt_ec] = '1'	Dose (Gray - Gy)	text (number)
906	[rt_no_ec]<br>Show the field ONLY if:<br>[rt_ec] = '0'	Reason external beam radiotherapy not administered (select all that apply)	checkbox<br>1 rt_no_ec___1 Poor performance status<br>2 rt_no_ec___2 No access<br>3 rt_no_ec___3 Patient unable to afford<br>4 rt_no_ec___4 Patient refused<br>5 rt_no_ec___5 Not clinically indicated<br>6 rt_no_ec___6 Other
907	[ps_rt_no_ec]<br>Show the field ONLY if:<br>[rt_no_ec(6)] = '1'	Please state	text
908	[brachy_ec]	Brachytherapy administered	yesno<br>1 Yes<br>0 No

909	[rt_dos_ec_2]<br>Show the field ONLY if:<br>[brachy_ec] = '1'	Dose (Gray - Gy)	text (number)
910	[brachy_no_ec]<br>Show the field ONLY if:<br>[brachy_ec] = '0'	Reason brachytherapy not administered (Select all that apply)	checkbox<br>1 brachy_no_ec___1 Poor performance status<br>2 brachy_no_ec___2 No access<br>3 brachy_no_ec___3 Patient unable to afford<br>4 brachy_no_ec___4 Patient refused<br>5 brachy_no_ec___5 Not clinically indicated<br>6 brachy_no_ec___6 Other
911	[ps_brachy_no_ec]<br>Show the field ONLY if:<br>[brachy_no_ec(6)] = '1'	Please state	text
912	[endometrial_cancer_initial_presentation_complete]	Section Header: *Form Status*<br>Complete?	dropdown<br>0 Incomplete<br>1 Unverified<br>2 Complete
Instrument: **Uterine Cancer (recurrence)** (endometrial_cancer_recurrence)			
913	[dat_ec]	Section Header: *PREVIOUS TREATMENT RECEIVED AT PRIMARY DIAGNOSIS*<br>Date of initial/primary diagnosis of endometrial cancer	text (date_dmy)
914	[prim_tx_ec]	Previous treatment at primary/initial diagnosis (select all that apply)	checkbox<br>1 prim_tx_ec___1 Cytoreductive surgery<br>2 prim_tx_ec___2 Adjuvant chemotherapy<br>3 prim_tx_ec___3 Neoadjuvant chemotherapy<br>4 prim_tx_ec___4 External beam radiotherapy<br>5 prim_tx_ec___5 Brachytherapy<br>6 prim_tx_ec___6 Immunotherapy
915	[dat_surg1_ec]<br>Show the field ONLY if:<br>[prim_tx_ec(1)] = '1'	Date of cytoreductive surgery following initial diagnosis	text (date_dmy)
916	[dat_chem1_ec]<br>Show the field ONLY if:<br>[prim_tx_ec(2)] = '1'	Date of adjuvant chemotherapy following initial diagnosis	text (date_dmy)
917	[dat_nact_ec]<br>Show the field ONLY if:<br>[prim_tx_ec(3)] = '1'	Date of neoadjuvant chemotherapy following initial diagnosis	text (date_dmy)
918	[dat_rt1_ec]<br>Show the field ONLY if:<br>[prim_tx_ec(4)] = '1'	Date of external beam radiotherapy following initial diagnosis	text (date_dmy)
919	[dat_brachy1]<br>Show the field ONLY if:<br>[prim_tx_ec(5)] = '1'	Date of brachytherapy following initial diagnosis	text (date_dmy)
920	[dat_imm_ec]<br>Show the field ONLY if:<br>[prim_tx_ec(6)] = '1'	Date of immunotherapy following initial diagnosis	text (date_dmy)
921	[rec_ec_dat]	Date of recurrence diagnosis	text (date_dmy)
922	[age_ec_v2]	Section Header: *DEMOGRAPHICS*<br>Age of patient	text (number)

923	[eth_ec_v2]	Ethnicity	dropdown<br>1 White (British, Australian, North American, European, other white)<br>2 Asian (South East Asian, Far East Asian, other Asian)<br>3 Black (African, Caribbean, other Black)<br>4 Middle Eastern (Arab, North African, Turkish)<br>5 Mixed (multiple ethnic background)<br>6 Other
924	[ps_eth_ec_v2]<br>Show the field ONLY if:<br>[eth_ec_v2] = '6'	Please state	text
925	[bmi_ec_rec]	Body mass index (kg/m2)	text (number)
926	[hrt_ec2]	History of unopposed oestrogen use (i.e. systemic oestrogen only hormone replacement therapy)?	yesno<br>1 Yes<br>0 No
927	[ein_ec2]	History of atypical hyperplasia/endometrial intraepithelial neoplasia?	yesno<br>1 Yes<br>0 No
928	[asa_ec_v2]	Section Header: *PRE-OPERATIVE*<br>American Society of Anesthesiologists (ASA) physical status	dropdown<br>1 ASA 1: A normal healthy patient. Example: Fit, non-obese (BMI under 30), a nonsmoking patient with good exercise tolerance.<br>2 ASA 2: A patient with a mild systemic disease. Example: Patient with no functional limitations and a well-controlled disease (e.g., treated hypertension, obesity with BMI under 35, frequent social drinker or is a cigarette smoker).<br>3 ASA 3: A patient with a severe systemic disease that is not life-threatening. Example: Patient with some functional limitation as a result of disease (e.g., poorly treated hypertension or diabetes, morbid obesity, chronic renal failure, a bronchospastic disease with intermittent exacerbation, stable angina, implanted pacemaker).<br>4 ASA 4: A patient with a severe systemic disease that is a constant threat to life. Example: Patient with functional limitation from severe, life-threatening disease (e.g., unstable angina, poorly controlled COPD, symptomatic CHF, recent (less than three months ago) myocardial infarction or stroke.<br>5 ASA 5: A moribund patient who is not expected to survive without the operation. The patient is not expected to survive beyond the next 24 hours without surgery. Examples: ruptured abdominal aortic aneurysm, massive trauma, and extensive intracranial hemorrhage with mass effect.<br>6 ASA 6: A brain-dead patient whose organs are being removed with the intention of transplanting them into another patient.

929	[ecog_ec_v2]	ECOG (Eastern Cooperative Oncology Group) performance status	dropdown<br>1 Grade 0 - Fully active, able to carry on all pre-disease performance without restriction<br>2 Grade 1 - Restricted in physically strenuous activity but ambulatory and able to carry out work of a light or sedentary nature, e.g., light house work, office work<br>3 Grade 2 - Ambulatory and capable of all selfcare but unable to carry out any work activities; up and about more than 50% of waking hours<br>4 Grade 3 - Capable of only limited selfcare; confined to bed or chair more than 50% of waking hours<br>5 Grade 4 - Completely disabled; cannot carry on any selfcare; totally confined to bed or chair<br>6 Grade 5 - Dead
930	[comorb_ec2]	Co-morbidities	checkbox<br>1 comorb_ec2___1 Cardiovascular<br>2 comorb_ec2___2 Respiratory<br>3 comorb_ec2___3 Urogenital<br>4 comorb_ec2___4 Endocrine<br>5 comorb_ec2___5 Neurological<br>6 comorb_ec2___6 Haematological<br>7 comorb_ec2___7 Psychiatric<br>8 comorb_ec2___8 Gastroenterology<br>9 comorb_ec2___9 Rheumatology and musculoskeletal<br>10 comorb_ec2___10 Oncology<br>11 comorb_ec2___11 None
931	[morb_cvd_ec2]<br>Show the field ONLY if:<br>[comorb_ec2(1)] = '1'	Select all that apply	checkbox<br>1 morb_cvd_ec2___1 Hypertension<br>2 morb_cvd_ec2___2 Coronary heart disease / ischaemic heart disease<br>3 morb_cvd_ec2___3 Myocardial infarction<br>4 morb_cvd_ec2___4 Heart failure<br>5 morb_cvd_ec2___5 Arrhythmia<br>6 morb_cvd_ec2___6 Heart valve disease<br>7 morb_cvd_ec2___7 Venous insufficiency<br>8 morb_cvd_ec2___8 Peripheral vascular disease<br>9 morb_cvd_ec2___9 Stroke<br>10 morb_cvd_ec2___10 Transient ischaemic attack<br>11 morb_cvd_ec2___11 Other
932	[comorb_resp_ec2]<br>Show the field ONLY if:<br>[comorb_ec2(2)] = '1'	Select all that apply	checkbox<br>1 comorb_resp_ec2___1 Asthma<br>2 comorb_resp_ec2___2 Emphysema (COPD)<br>3 comorb_resp_ec2___3 Chronic bronchitis (COPD)<br>4 comorb_resp_ec2___4 Pulmonary fibrosis<br>5 comorb_resp_ec2___5 Sleep apnoea<br>6 comorb_resp_ec2___6 Bronchiectasis<br>7 comorb_resp_ec2___7 Cystic fibrosis<br>8 comorb_resp_ec2___8 Tuberculosis<br>9 comorb_resp_ec2___9 Pneumonia<br>10 comorb_resp_ec2___10 Other

933	[comorb_uro_ec2]<br>Show the field ONLY if:<br>[comorb_ec2(3)] = '1'	Select all that apply	checkbox<br>1 comorb_uro_ec2___1 Chronic kidney disease (not requiring haemodialysis)<br>2 comorb_uro_ec2___2 Chronic kidney disease (requiring haemodialysis)<br>3 comorb_uro_ec2___3 Polycystic kidney disease<br>4 comorb_uro_ec2___4 Glomerulonephropathy<br>5 comorb_uro_ec2___5 Glomerulonephritis<br>6 comorb_uro_ec2___6 Urinary tract infection<br>7 comorb_uro_ec2___7 Other
934	[comorb_endo_ec2]<br>Show the field ONLY if:<br>[comorb_ec2(4)] = '1'	Select all that apply	checkbox<br>1 comorb_endo_ec2___1 Type 1 diabetes mellitus<br>2 comorb_endo_ec2___2 Type 2 diabetes mellitus<br>3 comorb_endo_ec2___3 Hyperthyroidism<br>4 comorb_endo_ec2___4 Hypothyroidism<br>5 comorb_endo_ec2___5 Other
935	[comorb_neuro_ec2]<br>Show the field ONLY if:<br>[comorb_ec2(5)] = '1'	Select all that apply	checkbox<br>1 comorb_neuro_ec2___1 Parkinsons disease<br>2 comorb_neuro_ec2___2 Myasthenia gravis<br>3 comorb_neuro_ec2___3 Dementia<br>4 comorb_neuro_ec2___4 Epilepsy<br>5 comorb_neuro_ec2___5 Other
936	[comorb_haem_ec2]<br>Show the field ONLY if:<br>[comorb_ec2(6)] = '1'	Select all that apply	checkbox<br>1 comorb_haem_ec2___1 Iron deficiency anaemia<br>2 comorb_haem_ec2___2 Folate deficience anaemia<br>3 comorb_haem_ec2___3 Vitamin B12 deficiency anaemia<br>4 comorb_haem_ec2___4 Pernicious anaemia<br>5 comorb_haem_ec2___5 Haemolytic anaemia<br>6 comorb_haem_ec2___6 Aplastic anaemia<br>7 comorb_haem_ec2___7 Thalassemia<br>8 comorb_haem_ec2___8 Sickle cell disease<br>9 comorb_haem_ec2___9 Disseminated intravascular coagulation (DIC)<br>10 comorb_haem_ec2___10 Factor V Leiden<br>11 comorb_haem_ec2___11 Thrombocytosis<br>12 comorb_haem_ec2___12 Idiopathic thrombocytopenic purpura (ITP)<br>13 comorb_haem_ec2___13 Haemophilia<br>14 comorb_haem_ec2___14 Von Willebrand disease<br>15 comorb_haem_ec2___15 Antiphospholipid syndrome<br>16 comorb_haem_ec2___16 Thrombocytopenia<br>17 comorb_haem_ec2___17 Thrombotic thrombocytopenic purpura<br>18 comorb_haem_ec2___18 Deep vein thrombosis<br>19 comorb_haem_ec2___19 Pulmonary embolism<br>20 comorb_haem_ec2___20 Other

937	[comorb_pscy_ec2]<br>Show the field ONLY if:<br>[comorb_ec2(7)] = '1'	Select all that apply	checkbox<br>1 comorb_pscy_ec2___1 Anxiety<br>2 comorb_pscy_ec2___2 Depression<br>3 comorb_pscy_ec2___3 Learning difficulties<br>4 comorb_pscy_ec2___4 Personality disorder<br>5 comorb_pscy_ec2___5 Schizophrenia<br>6 comorb_pscy_ec2___6 Bipolar disorder<br>7 comorb_pscy_ec2___7 Autism<br>8 comorb_pscy_ec2___8 Other
938	[comorb_gi_ec_2]<br>Show the field ONLY if:<br>[comorb_ec2(8)] = '1'	Select all that apply	checkbox<br>1 comorb_gi_ec_2___1 Crohn's disease<br>2 comorb_gi_ec_2___2 Ulcerative colitis<br>3 comorb_gi_ec_2___3 Irritable bowel syndrome<br>4 comorb_gi_ec_2___4 Celiac Disease<br>5 comorb_gi_ec_2___5 Peptic ulcer disease<br>6 comorb_gi_ec_2___6 Gallstones<br>7 comorb_gi_ec_2___7 Pancreatitis<br>8 comorb_gi_ec_2___8 Diverticulitis<br>9 comorb_gi_ec_2___9 Non-Alcoholic Fatty Liver Disease<br>10 comorb_gi_ec_2___10 Alcoholic fatty liver disease<br>11 comorb_gi_ec_2___11 Viral hepatitis<br>12 comorb_gi_ec_2___12 Liver failure<br>13 comorb_gi_ec_2___13 Other
939	[comorb_msk_ec_2]<br>Show the field ONLY if:<br>[comorb_ec2(9)] = '1'	Select all that apply	checkbox<br>1 comorb_msk_ec_2___1 Osteoarthritis<br>2 comorb_msk_ec_2___2 Rheumatoid arthritis<br>3 comorb_msk_ec_2___3 Osteoporosis<br>4 comorb_msk_ec_2___4 Lupus<br>5 comorb_msk_ec_2___5 Spondyloarthropathies<br>6 comorb_msk_ec_2___6 Sjogren's syndrome<br>7 comorb_msk_ec_2___7 Gout<br>8 comorb_msk_ec_2___8 Other
940	[comorb_onc_ec_2]<br>Show the field ONLY if:<br>[comorb_ec2(10)] = '1'	Select all that apply	checkbox<br>1 comorb_onc_ec_2___1 Breast cancer<br>2 comorb_onc_ec_2___2 Lung cancer<br>3 comorb_onc_ec_2___3 Hepatobiliary cancer<br>4 comorb_onc_ec_2___4 Brain cancer<br>5 comorb_onc_ec_2___5 Leukemia<br>6 comorb_onc_ec_2___6 Lymphoma<br>7 comorb_onc_ec_2___7 Osteosarcoma<br>8 comorb_onc_ec_2___8 Other
941	[lap_ec2]	Previous laparoscopic abdominal surgery	yesno<br>1 Yes<br>0 No
942	[opn_ec2]	Previous laparotomy	yesno<br>1 Yes<br>0 No

943	[figo_ec_v2]	FIGO Stage	dropdown<br>1 1A Limited to endometrium or involves less than half of the myometrium<br>2 IB Invasion of half or more of the myometrium<br>3 II Invasion of the cervical stroma but no extension outside the uterus<br>4 IIIA Invasion of serosa, adnexa, or both (direct extension or metastasis)<br>5 IIIB Metastases or direct spread to the vagina and/or spread to the parametria<br>6 IIIC1 Metastases to pelvic lymph nodes<br>7 IIIC2 Metastases to para-aortic lymph nodes, with or without metastases to pelvic lymph nodes<br>8 IVA Invasion of the bladder, intestinal mucosa, or both<br>9 IVB Distant metastases, including metastases to the inguinal lymph nodes, lungs, liver, or bone and intraperitoneal disease
944	[mdt_ec_v2]	Discussed at multidisciplinary team meeting/tumour board?	yesno<br>1 Yes<br>0 No
945	[mdt_tm_ec2]<br>Show the field ONLY if:<br>[mdt_ec_v2] = '1'	Multidisciplinary team/tumour board team members (select all that apply)	checkbox<br>1 mdt_tm_ec2___1 Certified gynaecological oncology surgeon<br>2 mdt_tm_ec2___2 Trained surgeon dedicated to the management of gynaecologic cancer or having completed an ESGO-accredited fellowship<br>3 mdt_tm_ec2___3 Radiologist<br>4 mdt_tm_ec2___4 Radiation/clinical oncologist (administering radiotherapy)<br>5 mdt_tm_ec2___5 Medical oncologist (administering chemotherapy)<br>6 mdt_tm_ec2___6 Pathologist
946	[mdt_ec_no_v2]<br>Show the field ONLY if:<br>[mdt_ec_v2] = '0'	Reason not discussed (select all that apply)	checkbox<br>1 mdt_ec_no_v2___1 No multidisciplinary team meeting/tumour board<br>2 mdt_ec_no_v2___2 Other
947	[ps_mdt_ec_no_v2]<br>Show the field ONLY if:<br>[mdt_ec_no_v2(2)] = '1'	Please state	text
948	[hystero_ec_v2]	Diagnostic biopsy preformed at recurrence diagnosis?	yesno<br>1 Yes<br>0 No
949	[bx_ec_no_reas_v2]<br>Show the field ONLY if:<br>[hystero_ec_v2] = '0'	Reason diagnostic biopsy not performed at recurrence diagnosis (select all that apply)	checkbox<br>1 bx_ec_no_reas_v2___1 Biopsy not technically feasible<br>2 bx_ec_no_reas_v2___2 Patient refused<br>3 bx_ec_no_reas_v2___3 Patient unable to afford<br>4 bx_ec_no_reas_v2___4 No facilities to perform biopsy<br>5 bx_ec_no_reas_v2___5 Other
950	[ps_bx_ec_no_reas_v2]<br>Show the field ONLY if:<br>[bx_ec_no_reas_v2(5)] = '1'	Please state	text
951	[uss_ec_v2]	Ultrasound scan performed at recurrence diagnosis?	yesno<br>1 Yes<br>0 No

952	[uss_no_reas_v2]<br>Show the field ONLY if:<br>[uss_ec_v2] = '0'	Reason ultrasound not performed at diagnosis (select all that apply)	checkbox<br>1 uss_no_reas_v2___1 No access to ultrasound machine<br>2 uss_no_reas_v2___2 Patient unable to afford<br>3 uss_no_reas_v2___3 Patient refused<br>4 uss_no_reas_v2___4 Other imaging performed<br>5 uss_no_reas_v2___5 Other
953	[ps_uss_no_reas_v2]<br>Show the field ONLY if:<br>[uss_no_reas_v2(5)] = '1'	Please state	text
954	[mri_ec_v2]	Magnetic resonance imaging (MRI) pelvis/abdomen - pelvis performed at recurrence diagnosis?	yesno<br>1 Yes<br>0 No
955	[mri_find_ec_v2]<br>Show the field ONLY if:<br>[mri_ec_v2] = '1'	MRI pelvis/abdomen-pelvis findings at recurrence diagnosis (select all that apply)	checkbox<br>1 mri_find_ec_v2___1 Post-hysterectomy pelvic mass<br>2 mri_find_ec_v2___2 x1 tubo-ovarian invasion<br>3 mri_find_ec_v2___3 x2 tubo-ovarian invasion<br>4 mri_find_ec_v2___4 Vaginal invasion<br>5 mri_find_ec_v2___5 Parametrial invasion<br>6 mri_find_ec_v2___6 Pelvic sidewall invasion<br>7 mri_find_ec_v2___7 Pelvic lymphadenopathy<br>8 mri_find_ec_v2___8 Inguinal lymphadenopathy<br>9 mri_find_ec_v2___9 Para-aortic lymphadenopathy<br>10 mri_find_ec_v2___10 x1 hydroureter<br>11 mri_find_ec_v2___11 x2 hydroureter<br>12 mri_find_ec_v2___12 Ureteric invasion<br>13 mri_find_ec_v2___13 x1 hydronephrosis<br>14 mri_find_ec_v2___14 x2 hydronephrosis<br>15 mri_find_ec_v2___15 Bladder invasion<br>16 mri_find_ec_v2___16 Para rectal nodules<br>17 mri_find_ec_v2___17 Sigmoid nodules<br>18 mri_find_ec_v2___18 Rectal and sigmoid diffuse serosal invasion<br>19 mri_find_ec_v2___19 Rectal and sigmoid diffuse wall invasion<br>20 mri_find_ec_v2___20 Rectal and sigmoid diffuse luminal invasion<br>21 mri_find_ec_v2___21 Peritoneal nodules<br>22 mri_find_ec_v2___22 Omental disease<br>23 mri_find_ec_v2___23 Ascites<br>24 mri_find_ec_v2___24 Liver invasion<br>25 mri_find_ec_v2___25 Splenic invasion<br>26 mri_find_ec_v2___26 Other
956	[ps_mri_find_ec_v2]<br>Show the field ONLY if:<br>[mri_find_ec_v2(26)] = '1'	Please state	text

957	[mri_ec_no_v2]<br>Show the field ONLY if:<br>[mri_ec_v2] = '0'	Reason MRI pelvis/abdominal-pelvis not performed at recurrence diagnosis (select all that apply)	checkbox<br>1 mri_ec_no_v2___1 No access to MRI machine<br>2 mri_ec_no_v2___2 Patient unable to afford<br>3 mri_ec_no_v2___3 Patient refused<br>4 mri_ec_no_v2___4 Claustrophobia<br>5 mri_ec_no_v2___5 Other imaging performed<br>6 mri_ec_no_v2___6 Extreme morbid obesity<br>7 mri_ec_no_v2___7 Other
958	[pc_mri_ec_no_v2]<br>Show the field ONLY if:<br>[mri_ec_no_v2(7)] = '1'	Please state	text
959	[ct_ec_v2]	Staging computerised tomography (CT) performed at recurrence diagnosis?	yesno<br>1 Yes<br>0 No
960	[ct_ec_find_v2]<br>Show the field ONLY if:<br>[ct_ec_v2] = '1'	Staging CT findings at recurrence diagnosis (select all that apply)	checkbox<br>1 ct_ec_find_v2___1 Post-hysterectomy pelvic mass<br>2 ct_ec_find_v2___2 x1 tubo-ovarian invasion<br>3 ct_ec_find_v2___3 x2 tubo-ovarian invasion<br>4 ct_ec_find_v2___4 Vaginal invasion<br>5 ct_ec_find_v2___5 Parametrial invasion<br>6 ct_ec_find_v2___6 Pelvic sidewall invasion<br>7 ct_ec_find_v2___7 Pelvic lymphadenopathy<br>8 ct_ec_find_v2___8 Inguinal lymphadenopathy<br>9 ct_ec_find_v2___9 Para-aortic lymphadenopathy<br>10 ct_ec_find_v2___10 x1 hydroureter<br>11 ct_ec_find_v2___11 x2 hydroureter<br>12 ct_ec_find_v2___12 Ureteric invasion<br>13 ct_ec_find_v2___13 x1 hydronephrosis<br>14 ct_ec_find_v2___14 x2 hydronephrosis<br>15 ct_ec_find_v2___15 Bladder invasion<br>16 ct_ec_find_v2___16 Pleural effusion<br>17 ct_ec_find_v2___17 Lung nodules<br>18 ct_ec_find_v2___18 Para rectal nodules<br>19 ct_ec_find_v2___19 Sigmoid nodules<br>20 ct_ec_find_v2___20 Rectal and sigmoid diffuse serosal invasion<br>21 ct_ec_find_v2___21 Rectal and sigmoid diffuse wall invasion<br>22 ct_ec_find_v2___22 Rectal and sigmoid diffuse luminal invasion<br>23 ct_ec_find_v2___23 Peritoneal nodules<br>24 ct_ec_find_v2___24 Omental disease<br>25 ct_ec_find_v2___25 Ascites<br>26 ct_ec_find_v2___26 Liver invasion<br>27 ct_ec_find_v2___27 Splenic invasion<br>28 ct_ec_find_v2___28 Other
961	[ps_ct_ec_find_v2]<br>Show the field ONLY if:<br>[ct_ec_find_v2(28)] = '1'	Please state	text

962	[ct_ec_no_v2]<br>Show the field ONLY if:<br>[ct_ec_v2] = '0'	Reason staging CT not performed at recurrence diagnosis (select all that apply)	checkbox<br>1 ct_ec_no_v2___1 No access to CT machine<br>2 ct_ec_no_v2___2 Patient unable to afford<br>3 ct_ec_no_v2___3 Patient refused<br>4 ct_ec_no_v2___4 Other imaging performed<br>5 ct_ec_no_v2___5 Allergic reaction to contrast<br>6 ct_ec_no_v2___6 Chronic kidney disease<br>7 ct_ec_no_v2___7 Other
963	[ps_ct_ec_no_v2]<br>Show the field ONLY if:<br>[ct_ec_no_v2(7)] = '1'	Please state	text
964	[petct_ec_v2]	Positron emission tomogrophy-computerised tomography (PET-CT) performed at recurrence diagnosis?	yesno<br>1 Yes<br>0 No
965	[petct_find_v2]<br>Show the field ONLY if:<br>[petct_ec_v2] = '1'	PET-CT findings at recurrence diagnosis (select all that apply)	checkbox<br>1 petct_find_v2___1 FDG-avid uptake ovaries<br>2 petct_find_v2___2 FDG-avid uptake in vagina<br>3 petct_find_v2___3 FDG-avid uptake in parametrium<br>4 petct_find_v2___4 FDG-avid uptake in pelvic side wall<br>5 petct_find_v2___5 FDG-avid uptake in pelvic lymph nodes<br>6 petct_find_v2___6 FDG-avid uptake in inguinal lymph nodes<br>7 petct_find_v2___7 FDG-avid uptake in para-aortic nodes<br>8 petct_find_v2___8 FDG-avid uptake in x1 ureter<br>9 petct_find_v2___9 FDG-avid uptake in x2 ureters<br>10 petct_find_v2___10 FDG-avid uptake in x1 kidney<br>11 petct_find_v2___11 FDG-avid uptake in x2 kidneys<br>12 petct_find_v2___12 FDG-avid uptake in bladder<br>13 petct_find_v2___13 FDG-avid uptake in lungs<br>14 petct_find_v2___14 FDG-avid uptake in rectum<br>15 petct_find_v2___15 FDG-avid uptake in sigmoid<br>16 petct_find_v2___16 FDG-avid uptake in rectal nodules<br>17 petct_find_v2___17 FDG-avid uptake in sigmoid nodules<br>18 petct_find_v2___18 FDG-avid uptake peritoneal nodules<br>19 petct_find_v2___19 FDG-avid uptake omentum<br>20 petct_find_v2___20 FDG-avid uptake spleen<br>21 petct_find_v2___21 FDG-avid uptake liver<br>22 petct_find_v2___22 Other
966	[ps_petct_find_v2]<br>Show the field ONLY if:<br>[petct_find_v2(22)] = '1'	Please state	text

967	[petct_no_ec_v2]<br>Show the field ONLY if:<br>[petct_ec_v2] = '0'	Reason PET-CT not performed at recurrence diagnosis (select all that apply)	checkbox<br>1 petct_no_ec_v2___1 No access to PET machine<br>2 petct_no_ec_v2___2 Patient unable to afford<br>3 petct_no_ec_v2___3 Patient refused<br>4 petct_no_ec_v2___4 Uncontrolled diabetes mellitus<br>5 petct_no_ec_v2___5 Other imaging performed<br>6 petct_no_ec_v2___6 Not clinical protocol<br>7 petct_no_ec_v2___7 Other
968	[ps_petct_no_ec_v2]<br>Show the field ONLY if:<br>[petct_no_ec_v2(7)] = '1'	Please state	text
969	[cov_ec_v2]	COVID-19 status	dropdown<br>1 Positive<br>2 Negative<br>3 Not tested
970	[cov_no_ec_v2]<br>Show the field ONLY if:<br>[cov_ec_v2] = '3'	If not tested select reason (select all that apply)	checkbox<br>1 cov_no_ec_v2___1 Not clinically indicated (asymptomatic)<br>2 cov_no_ec_v2___2 Not clinical protocol to routinely test all patients prior to surgery<br>3 cov_no_ec_v2___3 Patient refused<br>4 cov_no_ec_v2___4 Patient unable to afford test<br>6 cov_no_ec_v2___6 Testing not available locally<br>5 cov_no_ec_v2___5 Other
971	[ps_cov_no_ec_v2]<br>Show the field ONLY if:<br>[cov_no_ec_v2(5)] = '1'	Please state	text
972	[bp_ec2]	Bowel preparation pre-operatively	yesno<br>1 Yes<br>0 No
973	[peri_op_ec2]	Peri-operative management plan in place? (Non- exhaustive list: pre-operative haemoglobin optimisation and iron deficit correction; correction of denutrition and immunonutrition; fluid management; pain management; prevention of post-operative nausea and vomiting).	truefalse<br>1 True<br>0 False
974	[hb_ec2]	Pre-operative Haemoglobin (g/L)	text (number)
975	[wcc_ec2]	Pre-operative White Cell Count (10^9/L)	text (number)
976	[plts_ec2]	Pre-operative Platelets (10^9/L)	text (number)
977	[pt_ec2]	Pre-operative PT (Secs)	text (number)
978	[inr_ec2]	Pre-operative INR (ratio)	text (number)
979	[aptt_ec2]	Pre-operative APTT (Secs)	text (number)
980	[fib_ec2]	Pre-operative Fibrinogen (g/L)	text (number)
981	[alb_ec2]	Pre-operative Albumin (g/L)	text (number)
982	[na_ec2]	Pre-operative Sodium (mmol/L)	text (number)
983	[k_ec2]	Pre-operative Potassium (mmol/L)	text (number)
984	[ur_ec2]	Pre-operative Urea (mmol/L)	text (number)
985	[creat_ec2]	Pre-operative Creatinine (μmol/L)	text (number)
986	[egfr_ec2]	Pre-operative eGFR - estimated glomerular filtration rate (mL/min)	text
987	[bili_ec2]	Pre-operative Bilirubin total (μmol/L)	text (number)
988	[aat_ec2]	Pre-operative Alanine Aminotransaminase (IU/L)	text (number)
989	[alp_ec2]	Pre-operative Alkaline Phosphatase (IU/L)	text (number)
990	[ast_ec2]	Pre-operative Aspartate Aminotransaminase (IU/L)	text (number)
991	[crp_ec2]	Pre-operative CRP - C-reactive protein (mg/L)	text (number)

992	[datsurg_ec_v2]	Section Header: *SURGERY*<br>Date of recurrence surgery	text (date_dmy)
993	[anaes_ec_v2]	Anaesthesia (select all that apply)	checkbox<br>1 anaes_ec_v2___1 General<br>2 anaes_ec_v2___2 Epidural<br>3 anaes_ec_v2___3 Spinal
994	[sm_ec_v2]	Surgical modality	dropdown<br>1 Laparotomy<br>2 Laparoscopic<br>3 Robotic<br>4 Vaginal<br>5 Laparoscopic converted to laparotomy<br>6 Robotic converted to laparotomy<br>7 Vaginal converted to laparotomy
995	[elem_ec_v2]	Was recurrence surgery an elective or emergency case?	dropdown<br>1 Elective<br>2 Emergency
996	[lead_surg_ec_v2]	Lead surgeon performing recurrence surgery	dropdown<br>1 Certified consultant/specialist gynaecological oncologist<br>2 Fellow/trainee gynaecological oncologist<br>3 Trained surgeon dedicated to the management of gynaecologic cancer or having completed an ESGO accredited fellowship.<br>4 General surgeon<br>5 Surgical oncologist<br>6 Other
997	[ps_lead_surg_ec_v2]<br>Show the field ONLY if:<br>[lead_surg_ec_v2] = '6'	Please state	text
998	[who_ec2]	WHO surgical safety checklist used	yesno<br>1 Yes<br>0 No
999	[cyto_ec2]	Complete macroscopic cytoreduction at recurrence surgery	yesno<br>1 Yes<br>0 No
1000	[cytored_no_ec2]<br>Show the field ONLY if:<br>[cyto_ec2] = '0'	Reason(s) complete cytoreduction not achieved at recurrence surgery (select all that apply)	checkbox<br>1 cytored_no_ec2___1 Disease more extensive than that shown on imaging<br>2 cytored_no_ec2___2 Technically unable to resect<br>3 cytored_no_ec2___3 Patient became medically unstable intra-operatively and decision made to not proceed further with surgery<br>4 cytored_no_ec2___4 Other
1001	[ps_cytored_no_ec2]<br>Show the field ONLY if:<br>[cytored_no_ec2(4)] = '1'	Please state	text
1002	[siz_resid_ec2]<br>Show the field ONLY if:<br>[cyto_ec2] = '0'	Size of residual disease (in millimetres)	dropdown<br>1 1-2.5<br>2 2.6-5<br>3 5.1-7.5<br>4 7.6-10<br>5 >10

1003	[loc_resid_ec2]<br>Show the field ONLY if:<br>[cyto_ec2] = '0'	Location of residual disease at recurrence surgery (Select all that apply)	checkbox<br>1 loc_resid_ec2___1 fallopian tube(s)<br>2 loc_resid_ec2___2 ovary(s)<br>3 loc_resid_ec2___3 cervix<br>4 loc_resid_ec2___4 vagina<br>5 loc_resid_ec2___5 parametrium<br>6 loc_resid_ec2___6 pelvic side wall<br>7 loc_resid_ec2___7 pelvic lymph nodes<br>8 loc_resid_ec2___8 inguinal lymph nodes<br>9 loc_resid_ec2___9 para-aortic nodes<br>10 loc_resid_ec2___10 ureter(s)<br>11 loc_resid_ec2___11 kidney(s)<br>12 loc_resid_ec2___12 bladder<br>13 loc_resid_ec2___13 rectum<br>14 loc_resid_ec2___14 sigmoid<br>15 loc_resid_ec2___15 rectal nodules<br>16 loc_resid_ec2___16 sigmoid nodules<br>17 loc_resid_ec2___17 peritoneal nodules<br>18 loc_resid_ec2___18 omentum<br>19 loc_resid_ec2___19 Other
1004	[ps_loc_resid_ec2]<br>Show the field ONLY if:<br>[loc_resid_ec2(19)] = '1'	Please state	text
1005	[surgfind_ec_v2]	Findings at recurrence surgery (select all that apply)	checkbox<br>1 surgfind_ec_v2___1 x1 tubo-ovarian invasion<br>2 surgfind_ec_v2___2 x2 tubo-ovarian invasion<br>3 surgfind_ec_v2___3 Parametrial invasion<br>4 surgfind_ec_v2___4 Pelvic sidewall invasion<br>5 surgfind_ec_v2___5 Pelvic lymphadenopathy<br>6 surgfind_ec_v2___6 Inguinal lymphadenopathy<br>7 surgfind_ec_v2___7 Para-aortic lymphadenopathy<br>8 surgfind_ec_v2___8 Bladder invasion<br>9 surgfind_ec_v2___9 x1 hydroureter<br>10 surgfind_ec_v2___10 x2 hydroureter<br>11 surgfind_ec_v2___11 Ureteric invasion<br>12 surgfind_ec_v2___12 x1 hydronephrosis<br>13 surgfind_ec_v2___13 x2 hydronephrosis<br>14 surgfind_ec_v2___14 Para rectal nodules<br>15 surgfind_ec_v2___15 Sigmoid nodules<br>16 surgfind_ec_v2___16 Rectal and sigmoid diffuse serosal invasion<br>17 surgfind_ec_v2___17 Rectal and sigmoid diffuse wall invasion<br>18 surgfind_ec_v2___18 Rectal and sigmoid diffuse luminal invasion<br>19 surgfind_ec_v2___19 Peritoneal nodules<br>20 surgfind_ec_v2___20 Omental disease<br>21 surgfind_ec_v2___21 Ascites<br>22 surgfind_ec_v2___22 Splenic invasion<br>23 surgfind_ec_v2___23 Liver invasion<br>24 surgfind_ec_v2___24 Other<br>25 surgfind_ec_v2___25 No visible disease

1006	[ps_surgfind_ec_v2]<br>Show the field ONLY if:<br>[surgfind_ec_v2(24)] = '1'	Please state	text
1007	[tr_ec_v2]	Tissue removed (select all that apply)	checkbox<br>1 tr_ec_v2___1 x1 fallopian tube<br>2 tr_ec_v2___2 x2 fallopian tubes<br>3 tr_ec_v2___3 x1 ovary<br>4 tr_ec_v2___4 x2 ovaries<br>5 tr_ec_v2___5 Upper vagina<br>6 tr_ec_v2___6 Parametrium<br>7 tr_ec_v2___7 Pelvic lymph nodes<br>8 tr_ec_v2___8 Para-aortic lymph nodes<br>9 tr_ec_v2___9 Inguinal lymph nodes<br>10 tr_ec_v2___10 Rectum<br>11 tr_ec_v2___11 Sigmoid<br>12 tr_ec_v2___12 Bladder<br>13 tr_ec_v2___13 Ureter<br>14 tr_ec_v2___14 Kidney<br>15 tr_ec_v2___15 Peritoneum<br>16 tr_ec_v2___16 Omentum<br>17 tr_ec_v2___17 Spleen<br>18 tr_ec_v2___18 Liver resection<br>19 tr_ec_v2___19 Other
1008	[paln_dis_ec2]<br>Show the field ONLY if:<br>[tr_ec_v2(8)] = '1'	Select all that apply	checkbox<br>1 paln_dis_ec2___1 Infra-renal<br>2 paln_dis_ec2___2 Supra-renal
1009	[ps_tr_ec_v2]<br>Show the field ONLY if:<br>[tr_ec_v2(19)] = '1'	Please state	text
1010	[si_stom_ec_v2]	Small bowel stoma	yesno<br>1 Yes<br>0 No
1011	[ss_ec2]<br>Show the field ONLY if:<br>[si_stom_ec_v2] = '1'	Stoma site	dropdown<br>1 Duodenum<br>2 Jejunum<br>3 Ileum
1012	[pb_si_stom_ec_v2]<br>Show the field ONLY if:<br>[si_stom_ec_v2] = '1'	Performed by (select all that apply)	checkbox<br>1 pb_si_stom_ec_v2___1 Gynaecological oncologist<br>2 pb_si_stom_ec_v2___2 Colorectal surgeon<br>3 pb_si_stom_ec_v2___3 Urologist<br>4 pb_si_stom_ec_v2___4 General surgeon<br>5 pb_si_stom_ec_v2___5 Other
1013	[ps_pb_si_stom_ec_v2]<br>Show the field ONLY if:<br>[pb_si_stom_ec_v2(5)] = '1'	Please state	text
1014	[si_anast_ec_v2]	Small bowel anastamosis	yesno<br>1 Yes<br>0 No

1015	[pb_si_anast_ec_v2]<br>Show the field ONLY if:<br>[si_anast_ec_v2] = '1'	Performed by (select all that apply)	checkbox<br>1 pb_si_anast_ec_v2___1 Gynaecological oncologist<br>2 pb_si_anast_ec_v2___2 Colorectal surgeon<br>3 pb_si_anast_ec_v2___3 Urologist<br>4 pb_si_anast_ec_v2___4 General surgeon<br>5 pb_si_anast_ec_v2___5 Other
1016	[ps_pb_si_anast_ec_v2]<br>Show the field ONLY if:<br>[pb_si_anast_ec_v2(5)] = '1'	Please state	text
1017	[listom_ec_v2]	Large bowel stoma	yesno<br>1 Yes<br>0 No
1018	[pb_listom_ec_v2]<br>Show the field ONLY if:<br>[listom_ec_v2] = '1'	Performed by (please state)	checkbox<br>1 pb_listom_ec_v2___1 Gynaecological oncologist<br>2 pb_listom_ec_v2___2 Colorectal surgeon<br>3 pb_listom_ec_v2___3 Urologist<br>4 pb_listom_ec_v2___4 General surgeon<br>5 pb_listom_ec_v2___5 Other
1019	[ps_pb_listom_ec_v2]<br>Show the field ONLY if:<br>[pb_listom_ec_v2(5)] = '1'	Please state	text
1020	[lianas_ec_v2]	Large bowel anastamosis	yesno<br>1 Yes<br>0 No
1021	[pb_lianas_ec_v2]<br>Show the field ONLY if:<br>[lianas_ec_v2] = '1'	Performed by (select all that apply)	checkbox<br>1 pb_lianas_ec_v2___1 Gynaecological oncologist<br>2 pb_lianas_ec_v2___2 Colorectal surgeon<br>3 pb_lianas_ec_v2___3 Urologist<br>4 pb_lianas_ec_v2___4 General surgeon<br>5 pb_lianas_ec_v2___5 Other
1022	[ps_pb_lianas_ec_v2]<br>Show the field ONLY if:<br>[pb_lianas_ec_v2(5)] = '1'	Please state	text
1023	[uro_ec_v2]	Urostomy/ileal conduit	yesno<br>1 Yes<br>0 No
1024	[pb_uro_ec_v2]<br>Show the field ONLY if:<br>[uro_ec_v2] = '1'	Performed by (select all that apply)	checkbox<br>1 pb_uro_ec_v2___1 Gynaecological oncologist<br>2 pb_uro_ec_v2___2 Colorectal surgeon<br>3 pb_uro_ec_v2___3 Urologist<br>4 pb_uro_ec_v2___4 General surgeon<br>5 pb_uro_ec_v2___5 Other
1025	[ps_pb_uro_ec_v2]<br>Show the field ONLY if:<br>[pb_uro_ec_v2(5)] = '1'	Please state	text
1026	[tuu_ec_v2]	Trans uretero-ureteric anastamosis	yesno<br>1 Yes<br>0 No

1027	[pb_tuu_ec_v2]<br>Show the field ONLY if:<br>[tuu_ec_v2] = '1'	Performed by (select all that apply)	checkbox<br>1 pb_tuu_ec_v2___1 Gynaecological oncologist<br>2 pb_tuu_ec_v2___2 Colorectal surgeon<br>3 pb_tuu_ec_v2___3 Urologist<br>4 pb_tuu_ec_v2___4 General surgeon<br>5 pb_tuu_ec_v2___5 Other
1028	[ps_pb_tuu_ec_v2]<br>Show the field ONLY if:<br>[pb_tuu_ec_v2(5)] = '1'	Please state	text
1029	[ui_ec_v2]	Ureteric reimplantation	yesno<br>1 Yes<br>0 No
1030	[pb_ui_ec_v2]<br>Show the field ONLY if:<br>[ui_ec_v2] = '1'	Performed by (select all that apply)	checkbox<br>1 pb_ui_ec_v2___1 Gynaecological oncologist<br>2 pb_ui_ec_v2___2 Colorectal surgeon<br>3 pb_ui_ec_v2___3 Urologist<br>4 pb_ui_ec_v2___4 General surgeon<br>5 pb_ui_ec_v2___5 Other
1031	[ps_pb_ui_ec_v2]<br>Show the field ONLY if:<br>[pb_ui_ec_v2(5)] = '1'	Please state	text
1032	[neph_ec_v2]	Nephrostomy	yesno<br>1 Yes<br>0 No
1033	[aliedsurg_ec_v2]<br>Show the field ONLY if:<br>[neph_ec_v2] = '1'	Performed by (select all that apply)	checkbox<br>1 aliedsurg_ec_v2___1 Gynaecological oncologist<br>2 aliedsurg_ec_v2___2 Colorectal surgeon<br>3 aliedsurg_ec_v2___3 Urologist<br>4 aliedsurg_ec_v2___4 General surgeon<br>5 aliedsurg_ec_v2___5 Other
1034	[ps_alied_surg_ec_v2]<br>Show the field ONLY if:<br>[aliedsurg_ec_v2(5)] = '1'	Please state	text
1035	[abx_ec2]	Intraoperative antibiotics	yesno<br>1 Yes<br>0 No
1036	[tim_ec_v2]	Length of time of recurrence surgery	text (time)
1037	[itu_ec2]	Section Header: *POST-OPERATIVE*<br>Post-operative recovery in intensive care unit	yesno<br>1 Yes<br>0 No
1038	[itu_dys_ec2]<br>Show the field ONLY if:<br>[itu_ec2] = '1'	Number of days in intensive care unit	text (number)
1039	[hdu_ec2]	Post-operative recovery in high dependency unit	yesno<br>1 Yes<br>0 No
1040	[hdu_dys_ec2]<br>Show the field ONLY if:<br>[hdu_ec2] = '1'	Number of days in high dependency unit	text (number)
1041	[er_ec_v2]	Enhanced recovery?	yesno<br>1 Yes<br>0 No

1042	[po_abx_ec2]	Prophylactic post operative antibiotics	yesno<br>1 Yes<br>0 No
1043	[drn_ec2]	Post operative surgical drain	yesno<br>1 Yes<br>0 No
1044	[dys_drn_ec2]<br>Show the field ONLY if:<br>[drn_ec2] = '1'	Number of days in situ	text (number)
1045	[uc_ec2]	Post operative urinary catheter	yesno<br>1 Yes<br>0 No
1046	[dys_cath_ec2]<br>Show the field ONLY if:<br>[uc_ec2] = '1'	Number of days in situ	text (number)
1047	[phb_ec_2]	Post-operative Haemoglobin (g/L) - please state first post-operative result	text (number)
1048	[pwbc_ec_2]	Post-operative White Cell Count (10^9/L) - please state first post-operative result	text (number)
1049	[pplat_ec_2]	Post-operative Platelets (10^9/L) - please state first post-operative result	text (number)
1050	[ppt_ec_2]	Post-operative PT (Secs) - please state first post-operative result	text (number)
1051	[pinr_ec_2]	Post-operative INR (Ratio) - please state first post-operative result	text (number)
1052	[paptt_ec_2]	Post-operative APTT (Secs) - please state first post-operative result	text (number)
1053	[pfib_ec_2]	Post-operative Fibrinogen (g/L) - please state first post-operative result	text (number)
1054	[palb_ec_2]	Post-operative Albumin (g/L) - please state first post-operative result	text (number)
1055	[pna_ec_2]	Post-operative Sodium (mmol/L) - please state first post-operative result	text (number)
1056	[pk_ec_2]	Post-operative Potassium (mmol/L) - please state first post-operative result	text (number)
1057	[purea_ec_2]	Post-operative Urea (mmol/L) - please state first post-operative result	text (number)
1058	[pcreat_ec_2]	Post-operative Creatinine (μmol/L) - please state first post-operative result	text
1059	[pegfr_ec_2]	Post-operative eGFR - Estimated Glomerular Filtration Rate (mL/min) - please state first post-operative result	text
1060	[pbili_ec_2]	Post-operative Bilirubin total (μmol/L) - please state first post-operative result	text (number)
1061	[paat_ec_2]	Post-operative Alanine Aminotransaminase (IU/L) - please state first post-operative result	text (number)
1062	[palp_ec_2]	Post-operative Alkaline Phosphatase (IU/L) - please state first post-operative result	text (number)
1063	[past_ec_2]	Post-operative Aspartate Aminotransaminase (IU/L) - please state first post-operative result	text (number)
1064	[pcrp_ec_2]	Post-operative CRP - C-reactive protein (mg/L) - please state first post-operative result	text (number)

1065	[hist_ec_v2]	Histologic type	dropdown<br>1 Endometrioid<br>2 Mucinous<br>3 Serous<br>4 Clear cell<br>5 Squamous cell<br>6 Undifferentiated<br>7 Mixed<br>8 Carcinosarcoma<br>9 Leiomyosarcoma
1066	[resec_ec_v2]	Histological confirmed resection margins following recurrence surgery	dropdown<br>1 < 1 mm / tumour on margin<br>2 1-5 mm (NO tumour on margin)<br>3 >5 mm Margins confirmed clear<br>4 Unknown, not available in this hospital
1067	[tg_ec2]<br>Show the field ONLY if:<br>[hist_ec_v2] = '1'	Tumour Grade	dropdown<br>1 I<br>2 II<br>3 III
1068	[lvsi_ec2]	Lymphovascular space involvement (LVSI)	dropdown<br>1 No<br>2 Focal<br>3 Substantial
1069	[molec_ec_v2]	Molecular classification	dropdown<br>1 ultramutated/with pathogenic POLE mutations<br>2 hypermutated with MSI/MMRd (loss of MMR protein immunoreactivity)<br>3 high copy number/p53abn (p53 mutant immunoreactive pattern)<br>4 low copy number/NSMP (retained MMR protein immunoreactivity, and p53 wild-type immunoreactive pattern)<br>5 multiple classifier (any combination of markers included in the previous categories)<br>6 not classified
1070	[mlh1_v2]	Section Header: *Immunohistochemistry*<br>MLH1	radio (Matrix)<br>1 Positive<br>2 Negative<br>3 Not tested
1071	[msh2_v2]	MSH2	radio (Matrix)<br>1 Positive<br>2 Negative<br>3 Not tested
1072	[msh6_v2]	MSH6	radio (Matrix)<br>1 Positive<br>2 Negative<br>3 Not tested
1073	[pms2_v2]	PMS2	radio (Matrix)<br>1 Positive<br>2 Negative<br>3 Not tested

1074	[pten_v2]	PTEN	radio (Matrix)<br>1 Positive<br>2 Negative<br>3 Not tested
1075	[er_ihc_v2]	ER	radio (Matrix)<br>1 Positive<br>2 Negative<br>3 Not tested
1076	[pr_ihc_v2]	PR	radio (Matrix)<br>1 Positive<br>2 Negative<br>3 Not tested
1077	[p53_ihc_v2]	p53	radio (Matrix)<br>1 Positive<br>2 Negative<br>3 Not tested
1078	[p16_ihc_v2]	p16	radio (Matrix)<br>1 Positive<br>2 Negative<br>3 Not tested
1079	[her2_v2]	HER2	radio (Matrix)<br>1 Positive<br>2 Negative<br>3 Not tested
1080	[hmga2_v2]	HMGA2	radio (Matrix)<br>1 Positive<br>2 Negative<br>3 Not tested
1081	[cea_ihc_v2]	CEA	radio (Matrix)<br>1 Positive<br>2 Negative<br>3 Not tested
1082	[vim_ihc_v2]	Vimentin	radio (Matrix)<br>1 Positive<br>2 Negative<br>3 Not tested
1083	[bcat_ihc_v2]	β-catenin	radio (Matrix)<br>1 Positive<br>2 Negative<br>3 Not tested
1084	[arid1a_ihc_v2]	ARID1A	radio (Matrix)<br>1 Positive<br>2 Negative<br>3 Not tested
1085	[pax2_ihc_v2]	PAX2	radio (Matrix)<br>1 Positive<br>2 Negative<br>3 Not tested
1086	[wt1_ihc_v2]	WT-1	radio (Matrix)<br>1 Positive<br>2 Negative<br>3 Not tested

1087	[gt_ec_v2]	Genetic testing performed?	yesno<br>1 Yes<br>0 No
1088	[brca1_ec_v2]<br>Show the field ONLY if:<br>[gt_ec_v2] = '1'	Section Header: *Genetic testing*<br>BRCA1	radio (Matrix)<br>1 Positive<br>2 Negative<br>3 Not tested
1089	[brca2_ec_v2]<br>Show the field ONLY if:<br>[gt_ec_v2] = '1'	BRCA2	radio (Matrix)<br>1 Positive<br>2 Negative<br>3 Not tested
1090	[mlh1_ec_v2]<br>Show the field ONLY if:<br>[gt_ec_v2] = '1'	MLH1	radio (Matrix)<br>1 Positive<br>2 Negative<br>3 Not tested
1091	[msh2_ec_v2]<br>Show the field ONLY if:<br>[gt_ec_v2] = '1'	MSH2	radio (Matrix)<br>1 Positive<br>2 Negative<br>3 Not tested
1092	[msh6_ec_v2]<br>Show the field ONLY if:<br>[gt_ec_v2] = '1'	MSH6	radio (Matrix)<br>1 Positive<br>2 Negative<br>3 Not tested
1093	[pms2_ec_v2]<br>Show the field ONLY if:<br>[gt_ec_v2] = '1'	PMS2	radio (Matrix)<br>1 Positive<br>2 Negative<br>3 Not tested
1094	[no_gt_reas_ec_v2]<br>Show the field ONLY if:<br>[gt_ec_v2] = '0'	Reason genetic testing not performed (select all that apply)	checkbox<br>1 no_gt_reas_ec_v2___1 Not available<br>5 no_gt_reas_ec_v2___5 Does not meet clinical criteria for genetic testing<br>2 no_gt_reas_ec_v2___2 Patient unable to afford<br>4 no_gt_reas_ec_v2___4 Patient refused<br>3 no_gt_reas_ec_v2___3 Other
1095	[ps_no_gt_reas_ec_v2]<br>Show the field ONLY if:<br>[no_gt_reas_ec_v2(3)] = '1'	Please state	text
1096	[immuno_ec_v2]	Immunotherapy	yesno<br>1 Yes<br>0 No
1097	[adjct_ec_v2]	Chemotherapy following recurrence diagnosis	yesno<br>1 Yes<br>0 No

1098	[chem1_ec_v2]<br>Show the field ONLY if:<br>[adjct_ec_v2] = '1'	Chemotherapy regimen (Select all that apply)	checkbox<br>1 chem1_ec_v2___1 Platinum (for example cisplatin, carboplatin)<br>2 chem1_ec_v2___2 Taxane (for example paclitaxel)<br>3 chem1_ec_v2___3 Bevacuzimab<br>4 chem1_ec_v2___4 Doxorubicin<br>5 chem1_ec_v2___5 Clinical trial regimens<br>6 chem1_ec_v2___6 Aromatase inhibitors (for example anastrazole/letrozole)<br>7 chem1_ec_v2___7 Tamoxifen<br>8 chem1_ec_v2___8 Other
1099	[ps_chem1_ec_v2]<br>Show the field ONLY if:<br>[chem1_ec_v2(8)] = '1'	Please state	text
1100	[chem_numb_ec_v2]<br>Show the field ONLY if:<br>[adjct_ec_v2] = '1'	Number of cycles planned	text (number)
1101	[no_adjct_ec_v2]<br>Show the field ONLY if:<br>[adjct_ec_v2] = '0'	Reason chemotherapy not administered (select all that apply)	checkbox<br>1 no_adjct_ec_v2___1 Poor performance status<br>2 no_adjct_ec_v2___2 No access<br>3 no_adjct_ec_v2___3 Patient unable to afford<br>4 no_adjct_ec_v2___4 Patient refused<br>5 no_adjct_ec_v2___5 Not clinically indicated<br>6 no_adjct_ec_v2___6 Other
1102	[ps_no_adj_ec_v2]<br>Show the field ONLY if:<br>[no_adjct_ec_v2(6)] = '1'	Please state	text
1103	[rt_ec_v2]	External beam radiotherapy administered following recurrence diagnosis	yesno<br>1 Yes<br>0 No
1104	[rt_dos_ec2]<br>Show the field ONLY if:<br>[rt_ec_v2] = '1'	Dose (Gray - Gy)	text (number)
1105	[rt_y_ec_v2]<br>Show the field ONLY if:<br>[rt_ec_v2] = '1'	Administered to (select all that apply)	checkbox<br>1 rt_y_ec_v2___1 pelvis<br>2 rt_y_ec_v2___2 para-aortic strip<br>3 rt_y_ec_v2___3 pelvis and para-aortic strip<br>4 rt_y_ec_v2___4 other
1106	[ps_rt_y_ec_v2]<br>Show the field ONLY if:<br>[rt_y_ec_v2(4)] = '1'	Please state	text
1107	[rt_no_ec_v2]<br>Show the field ONLY if:<br>[rt_ec_v2] = '0'	Reason external beam radiotherapy not administered following recurrence diagnosis (select all that apply)	checkbox<br>1 rt_no_ec_v2___1 Poor performance status<br>2 rt_no_ec_v2___2 No access<br>3 rt_no_ec_v2___3 Patient unable to afford<br>4 rt_no_ec_v2___4 Patient refused<br>5 rt_no_ec_v2___5 Not clinically indicated<br>6 rt_no_ec_v2___6 Other
1108	[ps_rt_no_ec_v2]<br>Show the field ONLY if:<br>[rt_no_ec_v2(6)] = '1'	Please state	text
1109	[brachy_ec_v2]	Brachytherapy administered following recurrence diagnosis	yesno<br>1 Yes<br>0 No

1110	[rt_dos_ec3]<br>Show the field ONLY if:<br>[brachy_ec_v2] = '1'	Dose (Gray - Gy)	text (number)
1111	[brachy_no_ec_v2]<br>Show the field ONLY if:<br>[brachy_ec_v2] = '0'	Reason brachytherapy not administered following recurrence diagnosis (Select all that apply)	checkbox<br>1 brachy_no_ec_v2___1 Poor performance status<br>2 brachy_no_ec_v2___2 No access<br>3 brachy_no_ec_v2___3 Patient unable to afford<br>4 brachy_no_ec_v2___4 Patient refused<br>5 brachy_no_ec_v2___5 Not clinically indicated<br>6 brachy_no_ec_v2___6 Other
1112	[ps_brachy_no_ec_v2]<br>Show the field ONLY if:<br>[brachy_no_ec_v2(6)] = '1'	Please state	text
1113	[endometrial_cancer_recurrence_complete]	Section Header: *Form Status*<br>Complete?	dropdown<br>0 Incomplete<br>1 Unverified<br>2 Complete
Instrument: **Cervical Cancer (initial presentation)** (cervical_cancer_initial_presentation)			
1114	[dat_cc]	Section Header: *BASELINE DEMOGRAPHICS*<br>Date of primary diagnosis	text (date_dmy)
1115	[age_cc]	Age of patient	text (number)
1116	[eth_cc]	Ethnicity	dropdown<br>1 White (British, Australian, North American, European, other white)<br>2 Asian (South East Asian, Far East Asian, other Asian)<br>3 Black (African, Caribbean, other Black)<br>4 Middle Eastern (Arab, North African, Turkish)<br>5 Mixed (multiple ethnic background)<br>6 Other
1117	[ps_eth_cc]<br>Show the field ONLY if:<br>[eth_cc] = '6'	Please state	text
1118	[bmi_cc]	Body mass index (kg/m2)	text (number)
1119	[smoking_cc]	Smoker	dropdown<br>1 Yes<br>2 No<br>3 Ex-smoker
1120	[hpv_cc]	HPV status	dropdown<br>1 Positive<br>2 Negative<br>3 Not tested
1121	[hpv_typ_cc]<br>Show the field ONLY if:<br>[hpv_cc] = '1'	HPV type (select all that apply)	checkbox<br>1 hpv_typ_cc___1 16<br>2 hpv_typ_cc___2 18<br>3 hpv_typ_cc___3 Other<br>4 hpv_typ_cc___4 Unknown
1122	[hpv_not_test_cc]<br>Show the field ONLY if:<br>[hpv_cc] = '3'	Reason not tested (select all that apply)	checkbox<br>1 hpv_not_test_cc___1 Not clinical protocol<br>2 hpv_not_test_cc___2 Patient refused<br>3 hpv_not_test_cc___3 Patient unable to afford test<br>4 hpv_not_test_cc___4 Testing not available locally<br>5 hpv_not_test_cc___5 Other

1123	[ps_hpv_not_test_cc]<br>Show the field ONLY if:<br>[hpv_not_test_cc(5)] = '1'	Please state	text
1124	[hpv_vac_cc]	HPV vaccine administered historically	yesno<br>1 Yes<br>0 No
1125	[hpv_vac_typ_cc]<br>Show the field ONLY if:<br>[hpv_vac_cc] = '1'	Type of HPV vaccine administered	dropdown<br>1 Bivalent<br>2 Quadrivalent<br>3 9-valent
1126	[age_vac_cc]<br>Show the field ONLY if:<br>[hpv_vac_cc] = '1'	Age vaccine administered	text (number)
1127	[hpv_vac_no_cc]<br>Show the field ONLY if:<br>[hpv_vac_cc] = '0'	Reason vaccine not administered (Select all that apply)	checkbox<br>1 hpv_vac_no_cc___1 No national vaccination programme<br>2 hpv_vac_no_cc___2 National vaccination programme introduced after age of first sexual activity<br>3 hpv_vac_no_cc___3 Patient unable to afford<br>4 hpv_vac_no_cc___4 Other
1128	[ps_hpv_vac_no_cc]<br>Show the field ONLY if:<br>[hpv_vac_no_cc(4)] = '1'	Please state	text
1129	[asa_cc]	Section Header: *PRE-OPERATIVE*<br>American Society of Anesthesiologists (ASA) physical status	dropdown<br>1 ASA 1: A normal healthy patient. Example: Fit, non-obese (BMI under 30), a nonsmoking patient with good exercise tolerance.<br>2 ASA 2: A patient with a mild systemic disease. Example: Patient with no functional limitations and a well-controlled disease (e.g., treated hypertension, obesity with BMI under 35, frequent social drinker or is a cigarette smoker).<br>3 ASA 3: A patient with a severe systemic disease that is not life-threatening. Example: Patient with some functional limitation as a result of disease (e.g., poorly treated hypertension or diabetes, morbid obesity, chronic renal failure, a bronchospastic disease with intermittent exacerbation, stable angina, implanted pacemaker).<br>4 ASA 4: A patient with a severe systemic disease that is a constant threat to life. Example: Patient with functional limitation from severe, life-threatening disease (e.g., unstable angina, poorly controlled COPD, symptomatic CHF, recent (less than three months ago) myocardial infarction or stroke.<br>5 ASA 5: A moribund patient who is not expected to survive without the operation. The patient is not expected to survive beyond the next 24 hours without surgery. Examples: ruptured abdominal aortic aneurysm, massive trauma, and extensive intracranial hemorrhage with mass effect.<br>6 ASA 6: A brain-dead patient whose organs are being removed with the intention of transplanting them into another patient.

1130	[ecog_cc]	ECOG (Eastern Cooperative Oncology Group) performance status	dropdown<br>1 Grade 0 - Fully active, able to carry on all pre-disease performance without restriction<br>2 Grade 1 - Restricted in physically strenuous activity but ambulatory and able to carry out work of a light or sedentary nature, e.g., light house work, office work<br>3 Grade 2 - Ambulatory and capable of all selfcare but unable to carry out any work activities; up and about more than 50% of waking hours<br>4 Grade 3 - Capable of only limited selfcare; confined to bed or chair more than 50% of waking hours<br>5 Grade 4 - Completely disabled; cannot carry on any selfcare; totally confined to bed or chair<br>6 Grade 5 - Dead
1131	[comorb_cc]	Co-morbidities	checkbox<br>1 comorb_cc___1 Cardiovascular<br>2 comorb_cc___2 Respiratory<br>3 comorb_cc___3 Urogenital<br>4 comorb_cc___4 Endocrine<br>5 comorb_cc___5 Neurological<br>6 comorb_cc___6 Haematological<br>7 comorb_cc___7 Psychiatric<br>8 comorb_cc___8 Gastroenterology<br>9 comorb_cc___9 Rheumatology and musculoskeletal<br>10 comorb_cc___10 Oncology<br>11 comorb_cc___11 None
1132	[morb_cvd_cc]<br>Show the field ONLY if:<br>[comorb_cc(1)] = '1'	Select all that apply	checkbox<br>1 morb_cvd_cc___1 Hypertension<br>2 morb_cvd_cc___2 Coronary heart disease / ischaemic heart disease<br>3 morb_cvd_cc___3 Myocardial infarction<br>4 morb_cvd_cc___4 Heart failure<br>5 morb_cvd_cc___5 Arrhythmia<br>6 morb_cvd_cc___6 Heart valve disease<br>7 morb_cvd_cc___7 Venous insufficiency<br>8 morb_cvd_cc___8 Peripheral vascular disease<br>9 morb_cvd_cc___9 Stroke<br>10 morb_cvd_cc___10 Transient ischaemic attack<br>11 morb_cvd_cc___11 Other
1133	[comorb_resp_cc]<br>Show the field ONLY if:<br>[comorb_cc(2)] = '1'	Select all that apply	checkbox<br>1 comorb_resp_cc___1 Asthma<br>2 comorb_resp_cc___2 Emphysema (COPD)<br>3 comorb_resp_cc___3 Chronic bronchitis (COPD)<br>4 comorb_resp_cc___4 Pulmonary fibrosis<br>5 comorb_resp_cc___5 Sleep apnoea<br>6 comorb_resp_cc___6 Bronchiectasis<br>7 comorb_resp_cc___7 Cystic fibrosis<br>8 comorb_resp_cc___8 Tuberculosis<br>9 comorb_resp_cc___9 Pneumonia<br>10 comorb_resp_cc___10 Other

1134	[comorb_uro_cc]<br>Show the field ONLY if:<br>[comorb_cc(3)] = '1'	Select all that apply	checkbox<br>1 comorb_uro_cc___1 Chronic kidney disease (not requiring haemodialysis)<br>2 comorb_uro_cc___2 Chronic kidney disease (requiring haemodialysis)<br>3 comorb_uro_cc___3 Polycystic kidney disease<br>4 comorb_uro_cc___4 Glomerulonephropathy<br>5 comorb_uro_cc___5 Glomerulonephritis<br>6 comorb_uro_cc___6 Urinary tract infection<br>7 comorb_uro_cc___7 Other
1135	[comorb_endo_cc]<br>Show the field ONLY if:<br>[comorb_cc(4)] = '1'	Select all that apply	checkbox<br>1 comorb_endo_cc___1 Type 1 diabetes mellitus<br>2 comorb_endo_cc___2 Type 2 diabetes mellitus<br>3 comorb_endo_cc___3 Hyperthyroidism<br>4 comorb_endo_cc___4 Hypothyroidism<br>5 comorb_endo_cc___5 Other
1136	[comorb_neuro_cc]<br>Show the field ONLY if:<br>[comorb_cc(5)] = '1'	Select all that apply	checkbox<br>1 comorb_neuro_cc___1 Parkinsons disease<br>2 comorb_neuro_cc___2 Myasthenia gravis<br>3 comorb_neuro_cc___3 Dementia<br>4 comorb_neuro_cc___4 Epilepsy<br>5 comorb_neuro_cc___5 Other
1137	[comorb_haem_cc]<br>Show the field ONLY if:<br>[comorb_cc(6)] = '1'	Select all that apply	checkbox<br>1 comorb_haem_cc___1 Iron deficiency anaemia<br>2 comorb_haem_cc___2 Folate deficience anaemia<br>3 comorb_haem_cc___3 Vitamin B12 deficiency anaemia<br>4 comorb_haem_cc___4 Pernicious anaemia<br>5 comorb_haem_cc___5 Haemolytic anaemia<br>6 comorb_haem_cc___6 Aplastic anaemia<br>7 comorb_haem_cc___7 Thalassemia<br>8 comorb_haem_cc___8 Sickle cell disease<br>9 comorb_haem_cc___9 Disseminated intravascular coagulation (DIC)<br>10 comorb_haem_cc___10 Factor V Leiden<br>11 comorb_haem_cc___11 Thrombocytosis<br>12 comorb_haem_cc___12 Idiopathic thrombocytopenic purpura (ITP)<br>13 comorb_haem_cc___13 Haemophilia<br>14 comorb_haem_cc___14 Von Willebrand disease<br>15 comorb_haem_cc___15 Antiphospholipid syndrome<br>16 comorb_haem_cc___16 Thrombocytopenia<br>17 comorb_haem_cc___17 Thrombotic thrombocytopenic purpura<br>18 comorb_haem_cc___18 Deep vein thrombosis<br>19 comorb_haem_cc___19 Pulmonary embolism<br>20 comorb_haem_cc___20 Other

1138	[comorb_pscy_cc]<br>Show the field ONLY if:<br>[comorb_cc(7)] = '1'	Select all that apply	checkbox<br>1 comorb_pscy_cc___1 Anxiety<br>2 comorb_pscy_cc___2 Depression<br>3 comorb_pscy_cc___3 Learning difficulties<br>4 comorb_pscy_cc___4 Personality disorder<br>5 comorb_pscy_cc___5 Schizophrenia<br>6 comorb_pscy_cc___6 Bipolar disorder<br>7 comorb_pscy_cc___7 Autism<br>8 comorb_pscy_cc___8 Other
1139	[comorb_gi_cc]<br>Show the field ONLY if:<br>[comorb_cc(8)] = '1'	Select all that apply	checkbox<br>1 comorb_gi_cc___1 Crohn's disease<br>2 comorb_gi_cc___2 Ulcerative colitis<br>3 comorb_gi_cc___3 Irritable bowel syndrome<br>4 comorb_gi_cc___4 Celiac Disease<br>5 comorb_gi_cc___5 Peptic ulcer disease<br>6 comorb_gi_cc___6 Gallstones<br>7 comorb_gi_cc___7 Pancreatitis<br>8 comorb_gi_cc___8 Diverticulitis<br>9 comorb_gi_cc___9 Non-Alcoholic Fatty Liver Disease<br>10 comorb_gi_cc___10 Alcoholic fatty liver disease<br>11 comorb_gi_cc___11 Viral hepatitis<br>12 comorb_gi_cc___12 Liver failure<br>13 comorb_gi_cc___13 Other
1140	[comorb_msk_cc]<br>Show the field ONLY if:<br>[comorb_cc(9)] = '1'	Select all that apply	checkbox<br>1 comorb_msk_cc___1 Osteoarthritis<br>2 comorb_msk_cc___2 Rheumatoid arthritis<br>3 comorb_msk_cc___3 Osteoporosis<br>4 comorb_msk_cc___4 Lupus<br>5 comorb_msk_cc___5 Spondyloarthropathies<br>6 comorb_msk_cc___6 Sjogren's syndrome<br>7 comorb_msk_cc___7 Gout<br>8 comorb_msk_cc___8 Other
1141	[comorb_onc_cc]<br>Show the field ONLY if:<br>[comorb_cc(10)] = '1'	Select all that apply	checkbox<br>1 comorb_onc_cc___1 Breast cancer<br>2 comorb_onc_cc___2 Lung cancer<br>3 comorb_onc_cc___3 Hepatobiliary cancer<br>4 comorb_onc_cc___4 Brain cancer<br>5 comorb_onc_cc___5 Leukemia<br>6 comorb_onc_cc___6 Lymphoma<br>7 comorb_onc_cc___7 Osteosarcoma<br>8 comorb_onc_cc___8 Other
1142	[lap_cc]	Previous laparoscopic abdominal surgery	yesno<br>1 Yes<br>0 No
1143	[opn_cc]	Previous laparotomy	yesno<br>1 Yes<br>0 No
1144	[mdt_cc]	Discussed at multidisciplinary team meeting/tumour board?	yesno<br>1 Yes<br>0 No

1145	[mdt_tm_cc]<br>Show the field ONLY if:<br>[mdt_cc] = '1'	Multidisciplinary team/tumour board team members (select all that apply)	checkbox<br>1 mdt_tm_cc___1 Certified gynaecological oncology surgeon<br>2 mdt_tm_cc___2 Trained surgeon dedicated to the management of gynaecologic cancer (accounting for more than 80% of his/her practice) or having completed an ESGO-accredited fellowship<br>3 mdt_tm_cc___3 Radiologist<br>4 mdt_tm_cc___4 Radiation/clinical oncologist (administering radiotherapy)<br>5 mdt_tm_cc___5 Medical oncologist (administering chemotherapy)<br>6 mdt_tm_cc___6 Pathologist
1146	[mdt_cc_no]<br>Show the field ONLY if:<br>[mdt_cc] = '0'	Reason not discussed (Select all that apply)	checkbox<br>1 mdt_cc_no___1 No multidisciplinary team meeting/tumour board<br>2 mdt_cc_no___2 Other
1147	[ps_mdt_cc_no]<br>Show the field ONLY if:<br>[mdt_cc_no(2)] = '1'	Please state	text
1148	[bx_cc]	Diagnostic biopsy performed?	yesno<br>1 Yes<br>0 No
1149	[bx_no_cc_reas]<br>Show the field ONLY if:<br>[bx_cc] = '0'	Reason diagnostic biopsy not performed (select all that apply)	checkbox<br>1 bx_no_cc_reas___1 Biopsy not technically feasible<br>2 bx_no_cc_reas___2 Patient refused<br>3 bx_no_cc_reas___3 Patient unable to afford<br>4 bx_no_cc_reas___4 No resources locally to perform biopsy<br>5 bx_no_cc_reas___5 Other
1150	[ps_bx_no_cc_reas]<br>Show the field ONLY if:<br>[bx_no_cc_reas(5)] = '1'	Please state	text
1151	[mri_cc]	Magnetic resonance imaging (MRI) pelvis/abdomen-pelvis performed at diagnosis?	yesno<br>1 Yes<br>0 No

1152	[mri_find_cc]<br>Show the field ONLY if:<br>[mri_cc] = '1'	MRI pelvis/abdominal-pelvis findings at diagnosis (select all that apply)	checkbox<br>1 mri_find_cc___1 Tumour < 4cm confined to cervix<br>2 mri_find_cc___2 Tumour >4cm confined to cervix<br>3 mri_find_cc___3 Vaginal invasion (upper two thirds)<br>4 mri_find_cc___4 Vaginal invasion (lower third)<br>5 mri_find_cc___5 Parametrial invasion<br>6 mri_find_cc___6 Uterus invasion<br>7 mri_find_cc___7 x1 tubo-ovarian invasion<br>8 mri_find_cc___8 x2 tubo-ovarian invasion<br>9 mri_find_cc___9 Pelvic side wall invasion<br>10 mri_find_cc___10 Bladder invasion<br>11 mri_find_cc___11 Rectal invasion<br>12 mri_find_cc___12 Sigmoid invasion<br>13 mri_find_cc___13 x1 hydroureter<br>14 mri_find_cc___14 x2 hydroureter<br>15 mri_find_cc___15 Ureteric invasion<br>16 mri_find_cc___16 x1 hydronephrosis<br>17 mri_find_cc___17 x2 hydronephrosis<br>18 mri_find_cc___18 Urethral invasion<br>19 mri_find_cc___19 Pelvic lymphadenopathy<br>20 mri_find_cc___20 Para-aortic lymphadenopathy<br>21 mri_find_cc___21 Other
1153	[ps_mri_find_cc]<br>Show the field ONLY if:<br>[mri_find_cc(21)] = '1'	Please state	text
1154	[mri_no_cc]<br>Show the field ONLY if:<br>[mri_cc] = '0'	Reason MRI pelvis/abdomen-pelvis not performed at diagnosis (select all that apply)	checkbox<br>1 mri_no_cc___1 No access to MRI machine<br>2 mri_no_cc___2 Patient unable to afford<br>3 mri_no_cc___3 Patient refused<br>4 mri_no_cc___4 Claustrophobia<br>5 mri_no_cc___5 Other imaging performed<br>6 mri_no_cc___6 Extreme morbid obesity<br>7 mri_no_cc___7 Other
1155	[ps_mri_no_cc]<br>Show the field ONLY if:<br>[mri_no_cc(7)] = '1'	Please state	text
1156	[ct_cc]	Staging computerised tomogrophy (CT) performed at diagnosis	yesno<br>1 Yes<br>0 No

1157	[ct_find_cc]<br>Show the field ONLY if:<br>[ct_cc] = '1'	Staging CT findings at diagnosis (select all that apply)	checkbox<br>1 ct_find_cc___1 Tumour < 4cm confined to cervix<br>2 ct_find_cc___2 Tumour >4cm confined to cervix<br>3 ct_find_cc___3 Vaginal invasion (upper two thirds)<br>4 ct_find_cc___4 Vaginal invasion (lower third)<br>5 ct_find_cc___5 Parametrial invasion<br>6 ct_find_cc___6 Uterus invasion<br>7 ct_find_cc___7 x1 tubo-ovarian involvement<br>8 ct_find_cc___8 x2 tubo-ovarian involvement<br>9 ct_find_cc___9 Pelvic side wall invasion<br>10 ct_find_cc___10 Bladder invasion<br>11 ct_find_cc___11 Rectal invasion<br>12 ct_find_cc___12 Sigmoid invasion<br>13 ct_find_cc___13 x1 hydroureter<br>14 ct_find_cc___14 x2 hydroureter<br>15 ct_find_cc___15 Ureteric invasion<br>16 ct_find_cc___16 x1 hydronephrosis<br>17 ct_find_cc___17 x2 hydronephrosis<br>18 ct_find_cc___18 Urethral invasion<br>19 ct_find_cc___19 Pelvic lymphadenopathy<br>20 ct_find_cc___20 Para-aortic lymphadenopathy<br>21 ct_find_cc___21 Lung nodules<br>22 ct_find_cc___22 Pleural effusion<br>23 ct_find_cc___23 Other
1158	[ps_ct_find_cc]<br>Show the field ONLY if:<br>[ct_find_cc(23)] = '1'	Please state	text
1159	[ct_no_cc]<br>Show the field ONLY if:<br>[ct_cc] = '0'	Reason staging CT not performed at diagnosis (select all that apply)	checkbox<br>1 ct_no_cc___1 No access to CT machine<br>2 ct_no_cc___2 Patient unable to afford<br>4 ct_no_cc___4 Patient refused<br>5 ct_no_cc___5 Other imaging performed<br>6 ct_no_cc___6 Allergic reaction to contrast<br>7 ct_no_cc___7 Chronic kidney disease<br>3 ct_no_cc___3 Other
1160	[ps_ct_no_cc]<br>Show the field ONLY if:<br>[ct_no_cc(3)] = '1'	Please state	text
1161	[petct_cc]	PET-CT performed at diagnosis?	yesno<br>1 Yes<br>0 No

<table>
<tr><td>1162</td><td>[petct_find_cc]
Show the field ONLY if:
[petct_cc] = '1'</td><td>PET-CT findings at diagnosis (select all that apply)</td><td>checkbox<table>
<tr><td>1</td><td>petct_find_cc___1</td><td>Avid-FDG uptake in cervix</td></tr>
<tr><td>2</td><td>petct_find_cc___2</td><td>Avid-FDG uptake in vagina</td></tr>
<tr><td>3</td><td>petct_find_cc___3</td><td>Avid-FDG uptake in parametrium</td></tr>
<tr><td>4</td><td>petct_find_cc___4</td><td>Avid-FDG uptake in uterus</td></tr>
<tr><td>5</td><td>petct_find_cc___5</td><td>Avid-FDG uptake ovary(s)</td></tr>
<tr><td>6</td><td>petct_find_cc___6</td><td>Avid-FDG uptake in pelvic side wall</td></tr>
<tr><td>7</td><td>petct_find_cc___7</td><td>Avid-FDG uptake in pelvic lymph nodes</td></tr>
<tr><td>8</td><td>petct_find_cc___8</td><td>Avid-FDG uptake in para-aortic nodes</td></tr>
<tr><td>9</td><td>petct_find_cc___9</td><td>Avid-FDG uptake in x1 ureter</td></tr>
<tr><td>10</td><td>petct_find_cc___10</td><td>Avid-FDG uptake in x2 ureters</td></tr>
<tr><td>11</td><td>petct_find_cc___11</td><td>Avid-FDG uptake in x1 kidney</td></tr>
<tr><td>12</td><td>petct_find_cc___12</td><td>Avid-FDG uptake in x2 kidneys</td></tr>
<tr><td>13</td><td>petct_find_cc___13</td><td>Avid-FDG uptake in bladder</td></tr>
<tr><td>14</td><td>petct_find_cc___14</td><td>Avid-FDG uptake in lung nodules</td></tr>
<tr><td>15</td><td>petct_find_cc___15</td><td>Avid-FDG uptake in para rectal nodules</td></tr>
<tr><td>16</td><td>petct_find_cc___16</td><td>Avid-FDG uptake in sigmoid nodules</td></tr>
<tr><td>17</td><td>petct_find_cc___17</td><td>Avid-FDG uptake in rectum</td></tr>
<tr><td>18</td><td>petct_find_cc___18</td><td>Avid-FDG uptake in sigmoid</td></tr>
<tr><td>19</td><td>petct_find_cc___19</td><td>Other</td></tr>
</table></td></tr>
<tr><td>1163</td><td>[ps_petct_find_cc]
Show the field ONLY if:
[petct_find_cc(19)] = '1'</td><td>Please state</td><td>text</td></tr>
<tr><td>1164</td><td>[petct_no_cc]
Show the field ONLY if:
[petct_cc] = '0'</td><td>Reason PET-CT not performed at diagnosis (select all that apply)</td><td>checkbox<table>
<tr><td>1</td><td>petct_no_cc___1</td><td>No access to PET machine</td></tr>
<tr><td>2</td><td>petct_no_cc___2</td><td>Patient unable to afford</td></tr>
<tr><td>5</td><td>petct_no_cc___5</td><td>Patient refused</td></tr>
<tr><td>6</td><td>petct_no_cc___6</td><td>Uncontrolled diabetes mellitus</td></tr>
<tr><td>4</td><td>petct_no_cc___4</td><td>Other imaging performed</td></tr>
<tr><td>7</td><td>petct_no_cc___7</td><td>Not clinical protocol</td></tr>
<tr><td>3</td><td>petct_no_cc___3</td><td>Other</td></tr>
</table></td></tr>
<tr><td>1165</td><td>[ps_petct_no_cc]
Show the field ONLY if:
[petct_no_cc(3)] = '1'</td><td>Please state</td><td>text</td></tr>
</table>

1166	[figo_cc]	Clinical FIGO Stage (i.e. preoperative staging taking into account imaging, clinical examination)	dropdown<br>1 1A1 Invasive carcinoma diagnosed only by microscopy, measured stromal invasion >/=3 mm depth<br>3 1A2 Invasive carcinoma diagnosed only by microscopy, and >/=3 mm and < 5 mm in depth<br>5 1B1 Invasive carcinoma with >/= 5 mm depth of stromal invasion, and < 2 cm in greatest dimension, lesion limited to the cervix uteri<br>7 1B2 Invasive carcinoma with >/= 5 mm depth of stromal invasion, and >/= 2 cm and < 4cm in greatest dimension, lesion limited to the cervix uteri<br>9 IB3 Invasive carcinoma with >/= 5 mm depth of stromal invasion, and >/= 4 cm in greatest dimension, lesion limited to the cervix uteri<br>11 IIA1 Involvement limited to the upper two-thirds of the vagina without parametrial involvement, invasive carcinoma < 4cm in greatest dimension<br>12 IIA2 Involvement limited to the upper two-thirds of the vagina without parametrial involvement, invasive carcinoma >/=4cm in greatest dimension<br>13 IIB With parametrial involvement but not up to the pelvic wall<br>14 IIIA Carcinoma involves the lower third of the vagina with no extension to the pelvic wall<br>15 IIIB Extension to the pelvic wall and and/or causes hydronephrosis or non-functioning kidney<br>16 IIIC1: Involvement of pelvic lymph nodes, irrespective of tumour size and extent<br>17 IIIC2: Involvement of para-aortic lymph nodes, irrespective of tumour size and extent<br>18 IVA: Carcinoma has spread to the bladder or rectum<br>19 IVB: Carcinoma has spread to distant organs outside the pelvic area, such as distant lymph nodes, lungs or bones
1167	[nact_cc]	Neoadjuvant (before surgery) chemotherapy	yesno<br>1 Yes<br>0 No
1168	[nact_no_cc]<br>Show the field ONLY if:<br>[nact_cc] = '0'	Reason neoadjuvant chemotherapy not administered (select all that apply)	checkbox<br>1 nact_no_cc___1 Poor performance status<br>2 nact_no_cc___2 No access<br>3 nact_no_cc___3 Patient unable to afford<br>4 nact_no_cc___4 Patient refused<br>5 nact_no_cc___5 Not clinically indicated<br>6 nact_no_cc___6 Pregnancy<br>7 nact_no_cc___7 Other
1169	[ps_nact_no_cc]<br>Show the field ONLY if:<br>[nact_no_cc(7)] = '1'	Please state	text

1170	[chem1_cc]<br>Show the field ONLY if:<br>[nact_cc] = '1'	Chemotherapy regimen FIRST line (select all that apply)	checkbox<br>1 chem1_cc___1 Platinum (for example cisplatin, carboplatin)<br>2 chem1_cc___2 Fluorouracil<br>3 chem1_cc___3 Taxane (for example paclitaxel)<br>4 chem1_cc___4 Bevacuzimab<br>5 chem1_cc___5 Topotecan<br>6 chem1_cc___6 Gemcitabine<br>7 chem1_cc___7 Clinical trial regimens<br>8 chem1_cc___8 Other
1171	[ps_chem1_cc]<br>Show the field ONLY if:<br>[chem1_cc(8)] = '1'	Please state	text
1172	[numb_cycl_cc]<br>Show the field ONLY if:<br>[nact_cc] = '1'	Total number of cycles received	text (number)
1173	[cov19_cc]	COVID-19 status	dropdown<br>1 Positive<br>2 Negative<br>3 Not tested
1174	[cov19_no_cc]<br>Show the field ONLY if:<br>[cov19_cc] = '3'	If not tested select reason (select all that apply)	checkbox<br>1 cov19_no_cc___1 Not clinically indicated (asymptomatic)<br>2 cov19_no_cc___2 Not clinical protocol to routinely test all patients prior to surgery<br>3 cov19_no_cc___3 Patient refused<br>4 cov19_no_cc___4 Patient unable to afford test<br>5 cov19_no_cc___5 Testing not locally available<br>6 cov19_no_cc___6 Other
1175	[ps_cov19_no]<br>Show the field ONLY if:<br>[cov19_no_cc(6)] = '1'	Please state	text
1176	[bp_cc]	Bowel preparation pre-operatively	yesno<br>1 Yes<br>0 No
1177	[peri_op_cc]	Peri-operative management plan in place? (Non- exhaustive list: pre-operative haemoglobin optimisation and iron deficit correction; correction of denutrition and immunonutrition; fluid management; pain management; prevention of post-operative nausea and vomiting).	yesno<br>1 Yes<br>0 No
1178	[hb_cc]	Pre-operative Haemoglobin (g/L)	text (number)
1179	[wcc_cc]	Pre-operative White Cell Count (10^9/L)	text (number)
1180	[plt_cc]	Pre-operative Platelets (10^9/L)	text (number)
1181	[pt_cc]	Pre-operative PT (Secs)	text (number)
1182	[inr_cc]	Pre-operative INR (Ratio)	text (number)
1183	[aptt_cc]	Pre-operative APTT (Secs)	text (number)
1184	[fib_cc]	Pre-operative Fibrinogen (g/L)	text (number)
1185	[alb_cc]	Pre-operative Albumin (g/L)	text (number)
1186	[na_cc]	Pre-operative Sodium (mmol/L)	text (number)
1187	[k_cc]	Pre-operative Potassium (mmol/L)	text (number)
1188	[ur_cc]	Pre-operative Urea (mmol/L)	text (number)
1189	[creat_cc]	Pre-operative Creatinine (μmol/L)	text (number)
1190	[egfr_cc]	Pre-operative eGFR - estimated glomerular filtration rate (mL/min)	text
1191	[bili_cc]	Pre-operative Bilirubin (μmol/L)	text (number)
1192	[alt_cc]	Pre-operative Alanine Aminotransaminase (IU/L)	text (number)

1193	[alp_cc]	Pre-operative Alkaline Phosphatase (IU/L)	text (number)
1194	[ast_cc]	Pre-operative Aspartate Aminotransaminase (IU/L)	text (number)
1195	[crp_cc]	Pre-operative CRP - C-reactive protein (mg/L)	text (number)
1196	[datsurg_cc]	Section Header: *SURGERY*<br>Date of surgery	text (date_dmy)
1197	[anaes_cc]	Anaesthesia (select all that apply)	checkbox<br>1 anaes_cc___1 General<br>2 anaes_cc___2 Epidural<br>3 anaes_cc___3 Spinal
1198	[surg_cc]	Was the surgery an elective or emergency case?	radio<br>1 Elective<br>2 Emergency
1199	[sa_cc]	Surgical aim	dropdown<br>1 Curative<br>2 Curative attempted but then abandoned (i.e. open/close laparotomy)<br>3 Palliative
1200	[sm_cc]	Surgical modality	dropdown<br>1 Laparotomy<br>2 Laparoscopic<br>3 Robotic<br>6 Vaginal<br>4 Laparoscopic converted to laparotomy<br>5 Robotic converted to laparotomy<br>7 Vaginal converted to laparotomy
1201	[ls_cc]	Lead surgeon performing surgery	dropdown<br>1 Certified consultant/specialist gynaecological oncologist<br>2 Fellow/trainee gynaecological oncologist<br>3 Trained surgeon dedicated to the management of gynaecologic cancer (accounting for more than 80% of his/her practice) or having completed an ESGO accredited fellowship.<br>4 General surgeon<br>5 Surgical oncologist<br>6 Other
1202	[ps_ls_cc]<br>Show the field ONLY if:<br>[ls_cc] = '6'	Please state	text
1203	[who_cc]	WHO surgical safety checklist used	yesno<br>1 Yes<br>0 No
1204	[hyst_cc]	Radicality of hysterectomy (according to Querleu-Morrow, 2017)	dropdown<br>1 Type A<br>2 Type B1<br>3 Type B2<br>4 Type C1<br>5 Type C2<br>6 Type D
1205	[trach_cc]	Trachelectomy/fertility sparing surgery	yesno<br>1 Yes<br>0 No
1206	[cyto_cc]	Complete macroscopic cytoreduction at surgery	yesno<br>1 Yes<br>0 No

1207	[reas_cytored_no_cc]<br>Show the field ONLY if:<br>[cyto_cc] = '0'	Reason(s) complete cytoreduction not achieved (select all that apply)	checkbox<br>1 reas_cytored_no_cc___1 Disease more extensive than that shown on imaging<br>2 reas_cytored_no_cc___2 Technically unable to resect<br>3 reas_cytored_no_cc___3 Patient became medically unstable intra-operatively and decision made to not proceed further with surgery<br>4 reas_cytored_no_cc___4 Other
1208	[ps_reas_cytored_no_cc]<br>Show the field ONLY if:<br>[reas_cytored_no_cc(4)] = '1'	Please state	text
1209	[siz_resid_cc]<br>Show the field ONLY if:<br>[cyto_cc] = '0'	Size of residual disease (in millimetres)	dropdown<br>1 1-2.5<br>2 2.6-5<br>3 5.1-7.5<br>4 7.6-10<br>5 >10
1210	[loc_resid_cc]<br>Show the field ONLY if:<br>[cyto_cc] = '0'	Location of residual disease (select all that apply)	checkbox<br>1 loc_resid_cc___1 cervix<br>2 loc_resid_cc___2 vagina<br>3 loc_resid_cc___3 parametrium<br>4 loc_resid_cc___4 uterus<br>5 loc_resid_cc___5 ovary(s)<br>6 loc_resid_cc___6 pelvic side wall<br>7 loc_resid_cc___7 pelvic lymph nodes<br>8 loc_resid_cc___8 para-aortic nodes<br>9 loc_resid_cc___9 ureter(s)<br>10 loc_resid_cc___10 kidney(s)<br>11 loc_resid_cc___11 bladder<br>12 loc_resid_cc___12 para rectal nodules<br>13 loc_resid_cc___13 sigmoid nodules<br>14 loc_resid_cc___14 rectum<br>15 loc_resid_cc___15 sigmoid<br>16 loc_resid_cc___16 Other
1211	[ps_loc_resid_cc]<br>Show the field ONLY if:<br>[loc_resid_cc(16)] = '1'	Please state	text

<table>
<tr><td>1212</td><td>[surgfind_cc]</td><td>Findings at surgery (select all that apply)</td><td>checkbox
<table>
<tr><td>1</td><td>surgfind_cc___1</td><td>Tumour < 4cm cervix</td></tr>
<tr><td>2</td><td>surgfind_cc___2</td><td>Tumour >4cm cervix</td></tr>
<tr><td>3</td><td>surgfind_cc___3</td><td>Vaginal invasion (upper two thirds)</td></tr>
<tr><td>4</td><td>surgfind_cc___4</td><td>Vaginal invasion (lower third)</td></tr>
<tr><td>5</td><td>surgfind_cc___5</td><td>Parametrial invasion</td></tr>
<tr><td>6</td><td>surgfind_cc___6</td><td>Uterus invasion</td></tr>
<tr><td>7</td><td>surgfind_cc___7</td><td>x1 tubo-ovarian invasion</td></tr>
<tr><td>8</td><td>surgfind_cc___8</td><td>x2 tubo-ovarian invasion</td></tr>
<tr><td>9</td><td>surgfind_cc___9</td><td>Pelvic side wall invasion</td></tr>
<tr><td>10</td><td>surgfind_cc___10</td><td>Rectal invasion</td></tr>
<tr><td>11</td><td>surgfind_cc___11</td><td>Sigmoid invasion</td></tr>
<tr><td>12</td><td>surgfind_cc___12</td><td>Bladder invasion</td></tr>
<tr><td>13</td><td>surgfind_cc___13</td><td>x1 hydroureter</td></tr>
<tr><td>14</td><td>surgfind_cc___14</td><td>x2 hydroureter</td></tr>
<tr><td>15</td><td>surgfind_cc___15</td><td>Ureteric invasion</td></tr>
<tr><td>16</td><td>surgfind_cc___16</td><td>x1 hydronephrosis</td></tr>
<tr><td>17</td><td>surgfind_cc___17</td><td>x2 hydronephrosis</td></tr>
<tr><td>18</td><td>surgfind_cc___18</td><td>Pelvic lymphadenopathy</td></tr>
<tr><td>19</td><td>surgfind_cc___19</td><td>Para-aortic-lymphadenopathy</td></tr>
<tr><td>20</td><td>surgfind_cc___20</td><td>Lung nodules</td></tr>
<tr><td>21</td><td>surgfind_cc___21</td><td>Pleural effusion</td></tr>
<tr><td>22</td><td>surgfind_cc___22</td><td>Other</td></tr>
<tr><td>23</td><td>surgfind_cc___23</td><td>No visible disease</td></tr>
</table></td></tr>
<tr><td>1213</td><td>[ps_surgfind_cc]
Show the field ONLY if:
[surgfind_cc(22)] = '1'</td><td>Please state</td><td>text</td></tr>
<tr><td>1214</td><td>[tr_cc]</td><td>Tissue removed (select all that apply)</td><td>checkbox
<table>
<tr><td>1</td><td>tr_cc___1</td><td>Cervix</td></tr>
<tr><td>2</td><td>tr_cc___2</td><td>Uterus</td></tr>
<tr><td>3</td><td>tr_cc___3</td><td>Parametrium</td></tr>
<tr><td>4</td><td>tr_cc___4</td><td>Upper vagina</td></tr>
<tr><td>5</td><td>tr_cc___5</td><td>x1 fallopian tube</td></tr>
<tr><td>6</td><td>tr_cc___6</td><td>x2 fallopian tubes</td></tr>
<tr><td>7</td><td>tr_cc___7</td><td>x1 ovary</td></tr>
<tr><td>8</td><td>tr_cc___8</td><td>x2 ovaries</td></tr>
<tr><td>9</td><td>tr_cc___9</td><td>Pelvic lymph nodes</td></tr>
<tr><td>10</td><td>tr_cc___10</td><td>Para-aortic lymph nodes</td></tr>
<tr><td>11</td><td>tr_cc___11</td><td>Rectum</td></tr>
<tr><td>12</td><td>tr_cc___12</td><td>Sigmoid</td></tr>
<tr><td>13</td><td>tr_cc___13</td><td>ureter</td></tr>
<tr><td>14</td><td>tr_cc___14</td><td>kidney</td></tr>
<tr><td>15</td><td>tr_cc___15</td><td>Bladder</td></tr>
<tr><td>16</td><td>tr_cc___16</td><td>Other</td></tr>
</table></td></tr>
<tr><td>1215</td><td>[paln_dis_cc]
Show the field ONLY if:
[tr_cc(10)] = '1'</td><td>Select all that apply</td><td>checkbox
<table>
<tr><td>1</td><td>paln_dis_cc___1</td><td>Infra-renal</td></tr>
<tr><td>2</td><td>paln_dis_cc___2</td><td>Supra-renal</td></tr>
</table></td></tr>
<tr><td>1216</td><td>[ps_tr_cc]
Show the field ONLY if:
[tr_cc(16)] = '1'</td><td>Please state</td><td>text</td></tr>
</table>

1217	[sln_yn_cc]	Sentinel lymph node mapping	yesno<br>1 Yes<br>0 No
1218	[sln_no_cc]<br>Show the field ONLY if:<br>[sln_yn_cc] = '0'	Reason not performed (select all that apply)	checkbox<br>1 sln_no_cc___1 No facility<br>2 sln_no_cc___2 Not local protocol<br>3 sln_no_cc___3 Clinicians not trained<br>4 sln_no_cc___4 Patient refused<br>5 sln_no_cc___5 Patient unable to afford<br>6 sln_no_cc___6 Other
1219	[ps_sln_no_cc]<br>Show the field ONLY if:<br>[sln_no_cc(6)] = '1'	Please state	text
1220	[inj_sln_cc]<br>Show the field ONLY if:<br>[sln_yn_cc] = '1'	Site of injection (select all that apply)	checkbox<br>1 inj_sln_cc___1 Cervix<br>2 inj_sln_cc___2 Hysteroscopy<br>3 inj_sln_cc___3 Fundus<br>4 inj_sln_cc___4 Other
1221	[ps_inj_sln_cc]<br>Show the field ONLY if:<br>[inj_sln_cc(4)] = '1'	Please state	text
1222	[trcr_sln_cc]<br>Show the field ONLY if:<br>[sln_yn_cc] = '1'	Tracer	dropdown<br>1 Indocyanine green<br>2 Methylene blue<br>3 Technetium<br>4 Other
1223	[ps_trcr_sln_cc]<br>Show the field ONLY if:<br>[trcr_sln_cc] = '4'	Please state	text
1224	[map_sln_cc]<br>Show the field ONLY if:<br>[sln_yn_cc] = '1'	Site of mapping (select all that apply)	checkbox<br>1 map_sln_cc___1 right pelvic lymph node<br>2 map_sln_cc___2 left pelvic lymph node<br>3 map_sln_cc___3 para-aortic<br>4 map_sln_cc___4 presacral
1225	[r_pln_cc]<br>Show the field ONLY if:<br>[map_sln_cc(1)] = '1'	Results	dropdown<br>1 Positive<br>2 Negative
1226	[l_pln_cc]<br>Show the field ONLY if:<br>[map_sln_cc(2)] = '1'	Results	dropdown<br>1 Positive<br>2 Negative
1227	[paln_cc]<br>Show the field ONLY if:<br>[map_sln_cc(3)] = '1'	Results	dropdown<br>1 Positive<br>2 Negative
1228	[psln_cc]<br>Show the field ONLY if:<br>[map_sln_cc(4)] = '1'	Results	dropdown<br>1 Positive<br>2 Negative
1229	[si_stom_cc]	Small bowel stoma	yesno<br>1 Yes<br>0 No
1230	[ss_cc]<br>Show the field ONLY if:<br>[si_stom_cc] = '1'	Stoma site	dropdown<br>1 Duodenum<br>2 Jejunum<br>3 Ileum

1231	[pb_si_stom_cc]<br>Show the field ONLY if:<br>[si_stom_cc] = '1'	Performed by (select all that apply)	checkbox<br>1 pb_si_stom_cc___1 Gynaecological oncologist<br>2 pb_si_stom_cc___2 Colorectal surgeon<br>3 pb_si_stom_cc___3 Urologist<br>4 pb_si_stom_cc___4 General surgeon<br>5 pb_si_stom_cc___5 Other
1232	[ps_pb_si_stom_cc]<br>Show the field ONLY if:<br>[pb_si_stom_cc(5)] = '1'	Please state	text
1233	[si_anast_cc]	Small bowel anastamosis	yesno<br>1 Yes<br>0 No
1234	[pb_si_anast_cc]<br>Show the field ONLY if:<br>[si_anast_cc] = '1'	Performed by (select all that apply)	checkbox<br>1 pb_si_anast_cc___1 Gynaecological oncologist<br>2 pb_si_anast_cc___2 Colorectal surgeon<br>3 pb_si_anast_cc___3 Urologist<br>4 pb_si_anast_cc___4 General surgeon<br>5 pb_si_anast_cc___5 Other
1235	[ps_pb_si_anast_cc]<br>Show the field ONLY if:<br>[pb_si_anast_cc(5)] = '1'	Please state	text
1236	[li_stom_cc]	Large bowel stoma	yesno<br>1 Yes<br>0 No
1237	[pb_li_stom_cc]<br>Show the field ONLY if:<br>[li_stom_cc] = '1'	Performed by (select all that apply)	checkbox<br>1 pb_li_stom_cc___1 Gynaecological oncologist<br>2 pb_li_stom_cc___2 Colorectal surgeon<br>3 pb_li_stom_cc___3 Urologist<br>4 pb_li_stom_cc___4 General surgeon<br>5 pb_li_stom_cc___5 Other
1238	[ps_pb_li_stom_cc]<br>Show the field ONLY if:<br>[pb_li_stom_cc(5)] = '1'	Please state	text
1239	[li_anas]	Large bowel anastamosis	yesno<br>1 Yes<br>0 No
1240	[pb_li_anast_cc]<br>Show the field ONLY if:<br>[li_anas] = '1'	Performed by (select all that apply)	checkbox<br>1 pb_li_anast_cc___1 Gynaecological oncologist<br>2 pb_li_anast_cc___2 Colorectal surgeon<br>3 pb_li_anast_cc___3 Urologist<br>4 pb_li_anast_cc___4 General surgeon<br>5 pb_li_anast_cc___5 Other
1241	[ps_pb_li_anast_cc]<br>Show the field ONLY if:<br>[pb_li_anast_cc(5)] = '1'	Please state	text
1242	[uros_cc]	Urostomy/ileal conduit	yesno<br>1 Yes<br>0 No
1243	[pb_ic_cc]<br>Show the field ONLY if:<br>[uros_cc] = '1'	Performed by (select all that apply)	checkbox<br>1 pb_ic_cc___1 Gynaecological oncologist<br>2 pb_ic_cc___2 Colorectal surgeon<br>3 pb_ic_cc___3 Urologist<br>4 pb_ic_cc___4 General surgeon<br>5 pb_ic_cc___5 Other

1244	[ps_pb_ic_cc]<br>Show the field ONLY if:<br>[pb_ic_cc(5)] = '1'	Please state	text
1245	[tuu_cc]	Trans uretero-ureteric anastamosis	yesno<br>1 Yes<br>0 No
1246	[pb_tuu_cc]<br>Show the field ONLY if:<br>[tuu_cc] = '1'	Performed by (Select all that apply)	checkbox<br>1 pb_tuu_cc___1 Gynaecological oncologist<br>2 pb_tuu_cc___2 Colorectal surgeon<br>3 pb_tuu_cc___3 Urologist<br>4 pb_tuu_cc___4 General surgeon<br>5 pb_tuu_cc___5 Other
1247	[ps_pb_tuu_cc]<br>Show the field ONLY if:<br>[pb_tuu_cc(5)] = '1'	Please state	text
1248	[ui_cc]	Ureteric reimplantation	yesno<br>1 Yes<br>0 No
1249	[pb_ui_cc]<br>Show the field ONLY if:<br>[ui_cc] = '1'	Performed by (select all that apply)	checkbox<br>1 pb_ui_cc___1 Gynaecological oncologist<br>2 pb_ui_cc___2 Colorectal surgeon<br>3 pb_ui_cc___3 Urologist<br>4 pb_ui_cc___4 General surgeon<br>5 pb_ui_cc___5 Other
1250	[ps_pb_ui_cc]<br>Show the field ONLY if:<br>[pb_ui_cc(5)] = '1'	Please state	text
1251	[neph_cc]	Nephrostomy	yesno<br>1 Yes<br>0 No
1252	[surg2_cc]<br>Show the field ONLY if:<br>[neph_cc] = '1'	Performed by (select all that apply)	checkbox<br>1 surg2_cc___1 Gynaecological oncologist<br>2 surg2_cc___2 Colorectal surgeon<br>3 surg2_cc___3 Urologist<br>4 surg2_cc___4 General surgeon<br>5 surg2_cc___5 Other
1253	[ps_surg2_cc]<br>Show the field ONLY if:<br>[surg2_cc(5)] = '1'	Please state	text
1254	[abx_cc]	Intraoperative antibiotics	yesno<br>1 Yes<br>0 No
1255	[time_cc]	Length of surgery	text (time)
1256	[itu_cc]	Section Header: *POST-OPERATIVE*<br>Post-operative recovery in intensive care unit	yesno<br>1 Yes<br>0 No
1257	[itu_dys_cc]<br>Show the field ONLY if:<br>[itu_cc] = '1'	Number of days in intensive care unit	text (number)
1258	[hdu_cc]	Post-operative recovery in high dependency unit	yesno<br>1 Yes<br>0 No
1259	[hdu_dys_cc]<br>Show the field ONLY if:<br>[hdu_cc] = '1'	Number of days in high dependency unit	text (number)

1260	[erec_cc]	Enhanced recovery?	yesno<br>1 Yes<br>0 No
1261	[po_abx_cc]	Prophylactic post operative antibiotics	yesno<br>1 Yes<br>0 No
1262	[drn_cc]	Post operative surgical drain	yesno<br>1 Yes<br>0 No
1263	[drn_dys_cc]<br>Show the field ONLY if:<br>[drn_cc] = '1'	Number of days in situ	text (number)
1264	[uc_cc]	Post operative urinary catheter	yesno<br>1 Yes<br>0 No
1265	[cath_dys_cc]<br>Show the field ONLY if:<br>[uc_cc] = '1'	Number of days in situ	text (number)
1266	[phb_cc]	Post-operative Haemoglobin (g/L) - please state first post-operative result	text (number)
1267	[pwbc_cc]	Post-operative White Cell Count (10^9/L) - please state first post-operative result	text (number)
1268	[pplat_cc]	Post-operative Platelets (10^9/L) - please state first post-operative result	text (number)
1269	[ppt_cc]	Post-operative PT (Secs) - please state first post-operative result	text (number)
1270	[pinr_cc]	Post-operative INR (Ratio) - please state first post-operative result	text (number)
1271	[paptt_cc]	Post-operative APTT (Secs) - please state first post-operative result	text (number)
1272	[pfib_cc]	Post-operative Fibrinogen (g/L) - please state first post-operative result	text (number)
1273	[palb_cc]	Post-operative Albumin (g/L) - please state first post-operative result	text (number)
1274	[pna_cc]	Post-operative Sodium (mmol/L) - please state first post-operative result	text (number)
1275	[pk_cc]	Post-operative Potassium (mmol/L) - please state first post-operative result	text (number)
1276	[purea_cc]	Post-operative Urea (mmol/L) - please state first post-operative result	text (number)
1277	[pcreat_cc]	Post-operative Creatinine (μmol/L) - please state first post-operative result	text
1278	[pegfr_cc]	Post-operative eGFR - Estimated Glomerular Filtration Rate (mL/min) - please state first post-operative result	text
1279	[pbili_cc]	Post-operative Bilirubin total (μmol/L) - please state first post-operative result	text (number)
1280	[paat_cc]	Post-operative Alanine Aminotransaminase (IU/L) - please state first post-operative result	text (number)
1281	[palp_cc]	Post-operative Alkaline Phosphatase (IU/L) - please state first post-operative result	text (number)
1282	[past_cc]	Post-operative Aspartate Aminotransaminase (IU/L) - please state first post-operative result	text (number)
1283	[pcrp_cc]	Post-operative CRP - C-reactive protein (mg/L) - please state first post-operative result	text (number)

1284	[hist_cc]	Histologic type	dropdown<br>1 Squamous cell<br>2 Adenocarcinoma (glandular)<br>3 Adenosquamous<br>4 Mesenchymal<br>5 Mixed epithelial and mesenchymal<br>6 Melanocytic<br>7 Lymphoid<br>8 Small cell undifferentiated (neuroendocrine)
1285	[resec_cc]	Histological confirmed resection margins	dropdown<br>1 < 1 mm / tumour on margin<br>2 1-5 mm (NO tumour on margin)<br>3 >5 mm Margins confirmed clear<br>4 Unknown, not available in this hospital
1286	[plnd_cc]	Pelvic lymph nodes positive	dropdown<br>1 Yes<br>2 No<br>3 Not applicable - not removed
1287	[figo_cc_2]	Final surgical FIGO stage (i.e. postoperative staging taking into account surgical and pathology findings)	dropdown<br>1 1A1 Invasive carcinoma diagnosed only by microscopy, measured stromal invasion >/=3 mm depth<br>3 1A2 Invasive carcinoma diagnosed only by microscopy, and >/=3 mm and < 5 mm in depth<br>5 1B1 Invasive carcinoma with >/= 5 mm depth of stromal invasion, and < 2 cm in greatest dimension, lesion limited to the cervix uteri<br>7 1B2 Invasive carcinoma with >/= 5 mm depth of stromal invasion, and >/= 2 cm and < 4cm in greatest dimension, lesion limited to the cervix uteri<br>9 IB3 Invasive carcinoma with >/= 5 mm depth of stromal invasion, and >/= 4 cm in greatest dimension, lesion limited to the cervix uteri<br>11 IIA1 Involvement limited to the upper two-thirds of the vagina without parametrial involvement, invasive carcinoma < 4cm in greatest dimension<br>12 IIA2 Involvement limited to the upper two-thirds of the vagina without parametrial involvement, invasive carcinoma >/=4cm in greatest dimension<br>13 IIB With parametrial involvement but not up to the pelvic wall<br>14 IIIA Carcinoma involves the lower third of the vagina with no extension to the pelvic wall<br>15 IIIB Extension to the pelvic wall and and/or causes hydronephrosis or non-functioning kidney<br>16 IIIC1: Involvement of pelvic lymph nodes, irrespective of tumour size and extent<br>17 IIIC2: Involvement of para-aortic lymph nodes, irrespective of tumour size and extent<br>18 IVA: Carcinoma has spread to the bladder or rectum<br>19 IVB: Carcinoma has spread to distant organs outside the pelvic area, such as distant lymph nodes, lungs or bones

1288	[p16_cc]	Section Header: *Immunohistochemistry*<br>p16	radio (Matrix)<br>1 Positive<br>2 Negative<br>3 Not tested
1289	[m1bi_cc]	M1B1	radio (Matrix)<br>1 Positive<br>2 Negative<br>3 Not tested
1290	[ck20_cc]	CK20	radio (Matrix)<br>1 Positive<br>2 Negative<br>3 Not tested
1291	[bcl2_cc]	bcl-2	radio (Matrix)<br>1 Positive<br>2 Negative<br>3 Not tested
1292	[cea_cc]	monoclonal CEA	radio (Matrix)<br>1 Positive<br>2 Negative<br>3 Not tested
1293	[vim_cc]	Vimentin	radio (Matrix)<br>1 Positive<br>2 Negative<br>3 Not tested
1294	[er_cc]	Oestrogen receptor (ER)	radio (Matrix)<br>1 Positive<br>2 Negative<br>3 Not tested
1295	[cd10_cc]	CD10	radio (Matrix)<br>1 Positive<br>2 Negative<br>3 Not tested
1296	[ar_cc]	Androgen receptor	radio (Matrix)<br>1 Positive<br>2 Negative<br>3 Not tested
1297	[cal_cc]	Calretinin	radio (Matrix)<br>1 Positive<br>2 Negative<br>3 Not tested
1298	[inh_cc]	Inhibin	radio (Matrix)<br>1 Positive<br>2 Negative<br>3 Not tested
1299	[pr_cc]	Progesterone receptor (PR)	radio (Matrix)<br>1 Positive<br>2 Negative<br>3 Not tested
1300	[asma_cc]	a-SMA	radio (Matrix)<br>1 Positive<br>2 Negative<br>3 Not tested

1301	[hik108_cc]	HIK108	radio (Matrix)<br>1 Positive<br>2 Negative<br>3 Not tested
1302	[abpas_cc]	alcian blue/periodic acid-Schiff (AB/PAS) stain	radio (Matrix)<br>1 Positive<br>2 Negative<br>3 Not tested
1303	[chrom_cc]	chromogranin	radio (Matrix)<br>1 Positive<br>2 Negative<br>3 Not tested
1304	[synap_cc]	synaptophysin	radio (Matrix)<br>1 Positive<br>2 Negative<br>3 Not tested
1305	[pgp_cc]	PGP9.5	radio (Matrix)<br>1 Positive<br>2 Negative<br>3 Not tested
1306	[cd56_cc]	CD56	radio (Matrix)<br>1 Positive<br>2 Negative<br>3 Not tested
1307	[ttf1_cc]	TTF1	radio (Matrix)<br>1 Positive<br>2 Negative<br>3 Not tested
1308	[cd99_cc]	CD99	radio (Matrix)<br>1 Positive<br>2 Negative<br>3 Not tested
1309	[adjchemo_cc]	Adjuvant (after surgery) chemotherapy	yesno<br>1 Yes<br>0 No
1310	[adjchemo1_cc]<br>Show the field ONLY if:<br>[adjchemo_cc] = '1'	Chemotherapy regimen FIRST line (select all that apply)	checkbox<br>1 adjchemo1_cc___1 Platinum (for example cisplatin, carboplatin)<br>2 adjchemo1_cc___2 Fluorouracil<br>3 adjchemo1_cc___3 Taxane (for example paclitaxel)<br>4 adjchemo1_cc___4 Bevacuzimab<br>5 adjchemo1_cc___5 Topotecan<br>6 adjchemo1_cc___6 Gemcitabine<br>7 adjchemo1_cc___7 Clinical trial regimens<br>8 adjchemo1_cc___8 Other
1311	[ps_adjchemo1_cc]<br>Show the field ONLY if:<br>[adjchemo1_cc(8)] = '1'	Please state	text
1312	[adjchem1_cyc_cc]<br>Show the field ONLY if:<br>[adjchemo_cc] = '1'	Number of cycles planned	text (number)

1313	[adjchemo_no_cc]<br>Show the field ONLY if:<br>[adjchemo_cc] = '0'	Reason adjuvant chemotherapy not administered (select all that apply)	checkbox<br>5 adjchemo_no_cc___5 Poor performance status<br>1 adjchemo_no_cc___1 No access<br>2 adjchemo_no_cc___2 Patient unable to afford<br>4 adjchemo_no_cc___4 Patient refused<br>6 adjchemo_no_cc___6 Not clinically indicated<br>3 adjchemo_no_cc___3 Other
1314	[ps_adjchemo_no_cc]<br>Show the field ONLY if:<br>[adjchemo_no_cc(3)] = '1'	Please state	text
1315	[rt_cc]	External beam radiotherapy administered	yesno<br>1 Yes<br>0 No
1316	[rt_dos_cc]<br>Show the field ONLY if:<br>[rt_cc] = '1'	Dose (Gray - Gy)	text (number)
1317	[rt_cc_no]<br>Show the field ONLY if:<br>[rt_cc] = '0'	Reason external beam radiotherapy not administered (Select all that apply)	checkbox<br>1 rt_cc_no___1 Poor performance status<br>2 rt_cc_no___2 Not available locally<br>3 rt_cc_no___3 Patient unable to afford<br>4 rt_cc_no___4 Patient refused<br>5 rt_cc_no___5 Not clinically indicated<br>6 rt_cc_no___6 Fistula (urinary/rectal)<br>7 rt_cc_no___7 Other
1318	[ps_rt_cc_no]<br>Show the field ONLY if:<br>[rt_cc_no(7)] = '1'	Please state	text
1319	[bt_cc]	Brachytherapy administered	yesno<br>1 Yes<br>0 No
1320	[rt_dos_cc_2]<br>Show the field ONLY if:<br>[bt_cc] = '1'	Dose (Gray - Gy)	text (number)
1321	[bt_no_cc]<br>Show the field ONLY if:<br>[bt_cc] = '0'	Reason brachytherapy not administered (Select all that apply)	checkbox<br>1 bt_no_cc___1 Poor performance status<br>2 bt_no_cc___2 Not available locally<br>3 bt_no_cc___3 Patient unable to afford<br>4 bt_no_cc___4 Patient refused<br>5 bt_no_cc___5 Not clinically indicated<br>6 bt_no_cc___6 Other
1322	[ps_bt_no_cc]<br>Show the field ONLY if:<br>[bt_no_cc(6)] = '1'	Please state	text
1323	[cervical_cancer_initial_presentation_complete]	Section Header: *Form Status*<br>Complete?	dropdown<br>0 Incomplete<br>1 Unverified<br>2 Complete
Instrument: **Cervical Cancer (recurrence)** (cervical_cancer_recurrence)			
1324	[dat_dx_cc]	Section Header: *PREVIOUS TREATMENT RECEIVED AT PRIMARY DIAGNOSIS*<br>Date of initial/primary diagnosis	text (date_dmy)

1325	[prim_tx_cc]	Previous treatment following primary/initial diagnosis (select all that apply)	checkbox<br>1 prim_tx_cc___1 Cytoreductive surgery<br>2 prim_tx_cc___2 Adjuvant chemotherapy<br>3 prim_tx_cc___3 Neoadjuvant chemotherapy<br>4 prim_tx_cc___4 External beam radiotherapy<br>5 prim_tx_cc___5 Brachytherapy<br>6 prim_tx_cc___6 Immunotherapy
1326	[dat_surg1_cc]<br>Show the field ONLY if:<br>[prim_tx_cc(1)] = '1'	Date of primary cytoreductive surgery following initial/primary diagnosis	text (date_dmy)
1327	[dat_chem1_cc]<br>Show the field ONLY if:<br>[prim_tx_cc(2)] = '1'	Date of adjuvant chemotherapy following initial diagnosis	text (date_dmy)
1328	[dat_nact1_cc]<br>Show the field ONLY if:<br>[prim_tx_cc(3)] = '1'	Date of neoadjuvant chemotherapy following initial diagnosis	text (date_dmy)
1329	[dat_rt1_cc]<br>Show the field ONLY if:<br>[prim_tx_cc(4)] = '1'	Date of external beam radiotherapy following initial diagnosis	text (date_dmy)
1330	[dat_brachy1_cc]<br>Show the field ONLY if:<br>[prim_tx_cc(5)] = '1'	Date of brachytherapy following initial diagnosis	text (date_dmy)
1331	[dat_imm_cc_2]<br>Show the field ONLY if:<br>[prim_tx_cc(6)] = '1'	Date of immunotherapy following initial diagnosis	text (date_dmy)
1332	[dat_rec_dx_cc]	Date of recurrence diagnosis	text (date_dmy)
1333	[age_cc_v2]	Section Header: *DEMOGRAPHICS*<br>Age of patient	text (number)
1334	[eth_cc_v2]	Ethnicity	dropdown<br>1 White (British, Australian, North American, European, other white)<br>2 Asian (South East Asian, Far East Asian, other Asian)<br>3 Black (African, Caribbean, other Black)<br>4 Middle Eastern (Arab, North African, Turkish)<br>5 Mixed (multiple ethnic background)<br>6 Other
1335	[ps_eth_cc_v2]<br>Show the field ONLY if:<br>[eth_cc_v2] = '6'	Please state	text
1336	[bmi_cc_v2]	Body mass index (kg/m2)	text (number)
1337	[smoking_cc_v2]	Smoker	dropdown<br>1 Yes<br>2 No<br>3 Ex-smoker
1338	[hpv_cc_v2]	HPV status	dropdown<br>1 Positive<br>2 Negative<br>3 Not tested
1339	[hpv_typ_cc2]<br>Show the field ONLY if:<br>[hpv_cc_v2] = '1'	HPV type (Select all that apply)	checkbox<br>1 hpv_typ_cc2___1 16<br>2 hpv_typ_cc2___2 18<br>3 hpv_typ_cc2___3 Other<br>4 hpv_typ_cc2___4 Unknown

1340	[hpv_not_test_cc_v2]<br>Show the field ONLY if:<br>[hpv_cc_v2] = '3'	Reason not tested (select all that apply)	checkbox<br>1 hpv_not_test_cc_v2___1 Not clinical protocol<br>2 hpv_not_test_cc_v2___2 Patient refused<br>3 hpv_not_test_cc_v2___3 Patient unable to afford test<br>4 hpv_not_test_cc_v2___4 Testing not available locally<br>5 hpv_not_test_cc_v2___5 Other
1341	[ps_hpv_not_test_cc_v2]<br>Show the field ONLY if:<br>[hpv_not_test_cc_v2(5)] = '1'	Please state	text
1342	[hpv_vac_cc_v2]	HPV vaccine administered historically	yesno<br>1 Yes<br>0 No
1343	[hpv_vac_typ_cc_v2]<br>Show the field ONLY if:<br>[hpv_vac_cc_v2] = '1'	Type of HPV vaccine administered	dropdown<br>1 Bivalent<br>2 Quadrivalent<br>3 9-valent
1344	[age_vac_cc_v2]<br>Show the field ONLY if:<br>[hpv_vac_cc_v2] = '1'	Age vaccine administered	text (number)
1345	[hpv_vac_no_cc_v2]<br>Show the field ONLY if:<br>[hpv_vac_cc_v2] = '0'	Reason vaccine not administered (Select all that apply)	checkbox<br>1 hpv_vac_no_cc_v2___1 No national vaccination programme<br>2 hpv_vac_no_cc_v2___2 National vaccination programme introduced after age of first sexual activity<br>3 hpv_vac_no_cc_v2___3 Patient unable to afford<br>4 hpv_vac_no_cc_v2___4 Other
1346	[ps_hpv_vac_no_cc_v2]<br>Show the field ONLY if:<br>[hpv_vac_no_cc_v2(4)] = '1'	Please state	text

1347	[asa_cc_v2]	Section Header: *PRE-OPERATIVE*<br>American Society of Anesthesiologists (ASA) physical status	dropdown<br>1 ASA 1: A normal healthy patient. Example: Fit, non-obese (BMI under 30), a nonsmoking patient with good exercise tolerance.<br>2 ASA 2: A patient with a mild systemic disease. Example: Patient with no functional limitations and a well-controlled disease (e.g., treated hypertension, obesity with BMI under 35, frequent social drinker or is a cigarette smoker).<br>3 ASA 3: A patient with a severe systemic disease that is not life-threatening. Example: Patient with some functional limitation as a result of disease (e.g., poorly treated hypertension or diabetes, morbid obesity, chronic renal failure, a bronchospastic disease with intermittent exacerbation, stable angina, implanted pacemaker).<br>4 ASA 4: A patient with a severe systemic disease that is a constant threat to life. Example: Patient with functional limitation from severe, life-threatening disease (e.g., unstable angina, poorly controlled COPD, symptomatic CHF, recent (less than three months ago) myocardial infarction or stroke.<br>5 ASA 5: A moribund patient who is not expected to survive without the operation. The patient is not expected to survive beyond the next 24 hours without surgery. Examples: ruptured abdominal aortic aneurysm, massive trauma, and extensive intracranial hemorrhage with mass effect.<br>6 ASA 6: A brain-dead patient whose organs are being removed with the intention of transplanting them into another patient.
1348	[ecog_cc_v2]	ECOG (Eastern Cooperative Oncology Group) performance status	dropdown<br>1 Grade 0 - Fully active, able to carry on all pre-disease performance without restriction<br>2 Grade 1 - Restricted in physically strenuous activity but ambulatory and able to carry out work of a light or sedentary nature, e.g., light house work, office work<br>3 Grade 2 - Ambulatory and capable of all selfcare but unable to carry out any work activities; up and about more than 50% of waking hours<br>4 Grade 3 - Capable of only limited selfcare; confined to bed or chair more than 50% of waking hours<br>5 Grade 4 - Completely disabled; cannot carry on any selfcare; totally confined to bed or chair<br>6 Grade 5 - Dead
1349	[comorb_cc2]	Co-morbidities	checkbox<br>1 comorb_cc2___1 Cardiovascular<br>2 comorb_cc2___2 Respiratory<br>3 comorb_cc2___3 Urogenital<br>4 comorb_cc2___4 Endocrine<br>5 comorb_cc2___5 Neurological<br>6 comorb_cc2___6 Haematological<br>7 comorb_cc2___7 Psychiatric<br>8 comorb_cc2___8 Gastroenterology<br>9 comorb_cc2___9 Rheumatology and musculoskeletal<br>10 comorb_cc2___10 Oncology<br>11 comorb_cc2___11 None

1350	[morb_cvd_cc2]<br>Show the field ONLY if:<br>[comorb_cc2(1)] = '1'	Select all that apply	checkbox<br>1 morb_cvd_cc2___1 Hypertension<br>2 morb_cvd_cc2___2 Coronary heart disease / ischaemic heart disease<br>3 morb_cvd_cc2___3 Myocardial infarction<br>4 morb_cvd_cc2___4 Heart failure<br>5 morb_cvd_cc2___5 Arrhythmia<br>6 morb_cvd_cc2___6 Heart valve disease<br>7 morb_cvd_cc2___7 Venous insufficiency<br>8 morb_cvd_cc2___8 Peripheral vascular disease<br>9 morb_cvd_cc2___9 Stroke<br>10 morb_cvd_cc2___10 Transient ischaemic attack<br>11 morb_cvd_cc2___11 Other
1351	[comorb_resp_cc2]<br>Show the field ONLY if:<br>[comorb_cc2(2)] = '1'	Select all that apply	checkbox<br>1 comorb_resp_cc2___1 Asthma<br>2 comorb_resp_cc2___2 Emphysema (COPD)<br>3 comorb_resp_cc2___3 Chronic bronchitis (COPD)<br>4 comorb_resp_cc2___4 Pulmonary fibrosis<br>5 comorb_resp_cc2___5 Sleep apnoea<br>6 comorb_resp_cc2___6 Bronchiectasis<br>7 comorb_resp_cc2___7 Cystic fibrosis<br>8 comorb_resp_cc2___8 Tuberculosis<br>9 comorb_resp_cc2___9 Pneumonia<br>10 comorb_resp_cc2___10 Other
1352	[comorb_uro_cc2]<br>Show the field ONLY if:<br>[comorb_cc2(3)] = '1'	Select all that apply	checkbox<br>1 comorb_uro_cc2___1 Chronic kidney disease (not requiring haemodialysis)<br>2 comorb_uro_cc2___2 Chronic kidney disease (requiring haemodialysis)<br>3 comorb_uro_cc2___3 Polycystic kidney disease<br>4 comorb_uro_cc2___4 Glomerulonephropathy<br>5 comorb_uro_cc2___5 Glomerulonephritis<br>6 comorb_uro_cc2___6 Urinary tract infection<br>7 comorb_uro_cc2___7 Other
1353	[comorb_endo_cc2]<br>Show the field ONLY if:<br>[comorb_cc2(4)] = '1'	Select all that apply	checkbox<br>1 comorb_endo_cc2___1 Type 1 diabetes mellitus<br>2 comorb_endo_cc2___2 Type 2 diabetes mellitus<br>3 comorb_endo_cc2___3 Hyperthyroidism<br>4 comorb_endo_cc2___4 Hypothyroidism<br>5 comorb_endo_cc2___5 Other
1354	[comorb_neuro_cc2]<br>Show the field ONLY if:<br>[comorb_cc2(5)] = '1'	Select all that apply	checkbox<br>1 comorb_neuro_cc2___1 Parkinsons disease<br>2 comorb_neuro_cc2___2 Myasthenia gravis<br>3 comorb_neuro_cc2___3 Dementia<br>4 comorb_neuro_cc2___4 Epilepsy<br>5 comorb_neuro_cc2___5 Other

1355	[comorb_haem_cc2]<br>Show the field ONLY if:<br>[comorb_cc2(6)] = '1'	Select all that apply	checkbox<br>1 comorb_haem_cc2___1 Iron deficiency anaemia<br>2 comorb_haem_cc2___2 Folate deficience anaemia<br>3 comorb_haem_cc2___3 Vitamin B12 deficiency anaemia<br>4 comorb_haem_cc2___4 Pernicious anaemia<br>5 comorb_haem_cc2___5 Haemolytic anaemia<br>6 comorb_haem_cc2___6 Aplastic anaemia<br>7 comorb_haem_cc2___7 Thalassemia<br>8 comorb_haem_cc2___8 Sickle cell disease<br>9 comorb_haem_cc2___9 Disseminated intravascular coagulation (DIC)<br>10 comorb_haem_cc2___10 Factor V Leiden<br>11 comorb_haem_cc2___11 Thrombocytosis<br>12 comorb_haem_cc2___12 Idiopathic thrombocytopenic purpura (ITP)<br>13 comorb_haem_cc2___13 Haemophilia<br>14 comorb_haem_cc2___14 Von Willebrand disease<br>15 comorb_haem_cc2___15 Antiphospholipid syndrome<br>16 comorb_haem_cc2___16 Thrombocytopenia<br>17 comorb_haem_cc2___17 Thrombotic thrombocytopenic purpura<br>18 comorb_haem_cc2___18 Deep vein thrombosis<br>19 comorb_haem_cc2___19 Pulmonary embolism<br>20 comorb_haem_cc2___20 Other
1356	[comorb_pscy_cc2]<br>Show the field ONLY if:<br>[comorb_cc2(7)] = '1'	Select all that apply	checkbox<br>1 comorb_pscy_cc2___1 Anxiety<br>2 comorb_pscy_cc2___2 Depression<br>3 comorb_pscy_cc2___3 Learning difficulties<br>4 comorb_pscy_cc2___4 Personality disorder<br>5 comorb_pscy_cc2___5 Schizophrenia<br>6 comorb_pscy_cc2___6 Bipolar disorder<br>7 comorb_pscy_cc2___7 Autism<br>8 comorb_pscy_cc2___8 Other

1357	[comorb_gi_cc_2]<br>Show the field ONLY if:<br>[comorb_cc2(8)] = '1'	Select all that apply	checkbox<br>1 comorb_gi_cc_2___1 Crohn's disease<br>2 comorb_gi_cc_2___2 Ulcerative colitis<br>3 comorb_gi_cc_2___3 Irritable bowel syndrome<br>4 comorb_gi_cc_2___4 Celiac Disease<br>5 comorb_gi_cc_2___5 Peptic ulcer disease<br>6 comorb_gi_cc_2___6 Gallstones<br>7 comorb_gi_cc_2___7 Pancreatitis<br>8 comorb_gi_cc_2___8 Diverticulitis<br>9 comorb_gi_cc_2___9 Non-Alcoholic Fatty Liver Disease<br>10 comorb_gi_cc_2___10 Alcoholic fatty liver disease<br>11 comorb_gi_cc_2___11 Viral hepatitis<br>12 comorb_gi_cc_2___12 Liver failure<br>13 comorb_gi_cc_2___13 Other
1358	[comorb_msk_cc_2]<br>Show the field ONLY if:<br>[comorb_cc2(9)] = '1'	Select all that apply	checkbox<br>1 comorb_msk_cc_2___1 Osteoarthritis<br>2 comorb_msk_cc_2___2 Rheumatoid arthritis<br>3 comorb_msk_cc_2___3 Osteoporosis<br>4 comorb_msk_cc_2___4 Lupus<br>5 comorb_msk_cc_2___5 Spondyloarthropathies<br>6 comorb_msk_cc_2___6 Sjogren's syndrome<br>7 comorb_msk_cc_2___7 Gout<br>8 comorb_msk_cc_2___8 Other
1359	[comorb_onc_cc_2]<br>Show the field ONLY if:<br>[comorb_cc2(10)] = '1'	Select all that apply	checkbox<br>1 comorb_onc_cc_2___1 Breast cancer<br>2 comorb_onc_cc_2___2 Lung cancer<br>3 comorb_onc_cc_2___3 Hepatobiliary cancer<br>4 comorb_onc_cc_2___4 Brain cancer<br>5 comorb_onc_cc_2___5 Leukemia<br>6 comorb_onc_cc_2___6 Lymphoma<br>7 comorb_onc_cc_2___7 Osteosarcoma<br>8 comorb_onc_cc_2___8 Other
1360	[lap_cc2]	Previous laparoscopic abdominal surgery	yesno<br>1 Yes<br>0 No
1361	[opn_cc2]	Previous laparotomy	yesno<br>1 Yes<br>0 No
1362	[mdt_tm_cc2]<br>Show the field ONLY if:<br>[mdt_cc_v2] = '1'	Multidisciplinary team/tumour board team members (select all that apply)	checkbox<br>1 mdt_tm_cc2___1 Certified gynaecological oncology surgeon<br>2 mdt_tm_cc2___2 Trained surgeon dedicated to the management of gynaecologic cancer (accounting for more than 80% of his/her practice) or having completed an ESGO-accredited fellowship<br>3 mdt_tm_cc2___3 Radiologist<br>4 mdt_tm_cc2___4 Radiation/clinical oncologist (administering radiotherapy)<br>5 mdt_tm_cc2___5 Medical oncologist (administering chemotherapy)<br>6 mdt_tm_cc2___6 Pathologist

1363	[mdt_cc_v2]	Discussed at multidisciplinary team meeting/tumour board?	yesno<br>1 Yes<br>0 No
1364	[mdt_cc_no_v2]<br>Show the field ONLY if:<br>[mdt_cc_v2] = '0'	Reason not discussed (Select all that apply)	checkbox<br>1 mdt_cc_no_v2___1 No multidisciplinary team meeting/tumour board<br>2 mdt_cc_no_v2___2 Other
1365	[ps_mdt_cc_no_v2]<br>Show the field ONLY if:<br>[mdt_cc_no_v2(2)] = '1'	Please state	text
1366	[figo_cc_v2]	FIGO stage	dropdown<br>1 1A1 Invasive carcinoma diagnosed only by microscopy, measured stromal invasion >/=3 mm depth<br>3 1A2 Invasive carcinoma diagnosed only by microscopy, and >/=3 mm and < 5 mm in depth<br>5 1B1 Invasive carcinoma with >/= 5 mm depth of stromal invasion, and < 2 cm in greatest dimension, lesion limited to the cervix uteri<br>7 1B2 Invasive carcinoma with >/= 5 mm depth of stromal invasion, and >/= 2 cm and < 4cm in greatest dimension, lesion limited to the cervix uteri<br>9 IB3 Invasive carcinoma with >/= 5 mm depth of stromal invasion, and >/= 4 cm in greatest dimension, lesion limited to the cervix uteri<br>11 IIA1 Involvement limited to the upper two-thirds of the vagina without parametrial involvement, invasive carcinoma < 4cm in greatest dimension<br>12 IIA2 Involvement limited to the upper two-thirds of the vagina without parametrial involvement, invasive carcinoma >/=4cm in greatest dimension<br>13 IIB With parametrial involvement but not up to the pelvic wall<br>14 IIIA Carcinoma involves the lower third of the vagina with no extension to the pelvic wall<br>15 IIIB Extension to the pelvic wall and/or causes hydronephrosis or non-functioning kidney<br>16 IIIC1: Involvement of pelvic lymph nodes, irrespective of tumour size and extent<br>17 IIIC2: Involvement of para-aortic lymph nodes, irrespective of tumour size and extent<br>18 IVA: Carcinoma has spread to the bladder or rectum.<br>19 IVB: Carcinoma has spread to distant organs outside the pelvic area, such as distant lymph nodes, lungs or bones.
1367	[bx_cc_v2]	Diagnostic biopsy performed at recurrence?	yesno<br>1 Yes<br>0 No
1368	[bx_no_cc_reas_v2]<br>Show the field ONLY if:<br>[bx_cc_v2] = '0'	Reason diagnostic biopsy not performed at recurrence (select all that apply)	checkbox<br>1 bx_no_cc_reas_v2___1 Biopsy not technically feasible<br>2 bx_no_cc_reas_v2___2 Patient refused<br>3 bx_no_cc_reas_v2___3 Patient unable to afford<br>4 bx_no_cc_reas_v2___4 No resources locally to perform biopsy<br>5 bx_no_cc_reas_v2___5 Other

1369	[ps_bx_no_cc_reas_v2]<br>Show the field ONLY if:<br>[bx_no_cc_reas_v2(5)] = '1'	Please state	text
1370	[mri_cc_v2]	Magnetic resonance imaging (MRI) pelvis/abdomen-pelvis performed at recurrence diagnosis?	yesno<br>1 Yes<br>0 No
1371	[mri_find_cc_v2]<br>Show the field ONLY if:<br>[mri_cc_v2] = '1'	MRI pelvis/abdominal-pelvis findings at recurrence diagnosis (select all that apply)	checkbox<br>1 mri_find_cc_v2___1 Vaginal invasion (upper two thirds)<br>2 mri_find_cc_v2___2 Vaginal invasion (lower third)<br>3 mri_find_cc_v2___3 Parametrial invasion<br>4 mri_find_cc_v2___4 Uterus invasion<br>5 mri_find_cc_v2___5 x1 tubo-ovarian invasion<br>6 mri_find_cc_v2___6 x2 tubo-ovarian invasion<br>7 mri_find_cc_v2___7 Psoas major invasion<br>8 mri_find_cc_v2___8 Obturator internus invasion<br>9 mri_find_cc_v2___9 Sciatic nerve invasion<br>10 mri_find_cc_v2___10 Ureter invasion<br>11 mri_find_cc_v2___11 Bladder invasion<br>12 mri_find_cc_v2___12 x1 hydroureter<br>13 mri_find_cc_v2___13 x2 hydroureter<br>14 mri_find_cc_v2___14 x1 hydronephrosis<br>15 mri_find_cc_v2___15 x2 hydronephrosis<br>16 mri_find_cc_v2___16 Urethral invasion<br>17 mri_find_cc_v2___17 Rectal invasion<br>18 mri_find_cc_v2___18 Sigmoid invasion<br>19 mri_find_cc_v2___19 Pelvic lymphadenopathy<br>20 mri_find_cc_v2___20 Para-aortic lymphadenopathy<br>21 mri_find_cc_v2___21 Other
1372	[ps_mri_find_cc_v2]<br>Show the field ONLY if:<br>[mri_find_cc_v2(21)] = '1'	Please state	text
1373	[mri_no_cc_v2]<br>Show the field ONLY if:<br>[mri_cc_v2] = '0'	Reason MRI pelvis/abdomen-pelvis not performed at recurrence diagnosis (select all that apply)	checkbox<br>1 mri_no_cc_v2___1 No access to MRI machine<br>2 mri_no_cc_v2___2 Patient unable to afford<br>3 mri_no_cc_v2___3 Patient refused<br>4 mri_no_cc_v2___4 Claustrophobia<br>5 mri_no_cc_v2___5 Other imaging performed<br>6 mri_no_cc_v2___6 Extreme morbid obesity<br>7 mri_no_cc_v2___7 Other
1374	[ps_mri_no_cc_v2]<br>Show the field ONLY if:<br>[mri_no_cc_v2(7)] = '1'	Please state	text
1375	[ct_cc_v2]	Staging computerised tomogrophy (CT) performed at recurrence diagnosis	yesno<br>1 Yes<br>0 No

1376	[ct_find_cc_v2]<br><br>Show the field ONLY if:<br>[ct_cc_v2] = '1'	Staging CT findings at recurrence diagnosis (select all that apply)	checkbox<br>1 ct_find_cc_v2___1 Vaginal invasion (upper two thirds)<br>2 ct_find_cc_v2___2 Vaginal invasion (lower third)<br>3 ct_find_cc_v2___3 Parametrial invasion<br>4 ct_find_cc_v2___4 Uterus invasion<br>5 ct_find_cc_v2___5 x1 tubo-ovarian invasion<br>6 ct_find_cc_v2___6 x2 tubo-ovarian invasion<br>7 ct_find_cc_v2___7 Psoas major invasion<br>8 ct_find_cc_v2___8 Obturator internus invasion<br>9 ct_find_cc_v2___9 Sciatic nerve invasion<br>10 ct_find_cc_v2___10 Ureter invasion<br>11 ct_find_cc_v2___11 Bladder invasion<br>12 ct_find_cc_v2___12 x1 hydroureter<br>13 ct_find_cc_v2___13 x2 hydroureter<br>14 ct_find_cc_v2___14 x1 hydronephrosis<br>15 ct_find_cc_v2___15 x2 hydronephrosis<br>16 ct_find_cc_v2___16 Urethral invasion<br>17 ct_find_cc_v2___17 Rectal invasion<br>18 ct_find_cc_v2___18 Sigmoid invasion<br>19 ct_find_cc_v2___19 Pelvic lymphadenopathy<br>20 ct_find_cc_v2___20 Para-aortic lymphadenopathy<br>21 ct_find_cc_v2___21 Pleural effusion<br>22 ct_find_cc_v2___22 Pleural nodules<br>23 ct_find_cc_v2___23 Other
1377	[ps_ct_find_cc_v2]<br><br>Show the field ONLY if:<br>[ct_find_cc_v2(23)] = '1'	Please state	text
1378	[ct_no_cc_v2]<br><br>Show the field ONLY if:<br>[ct_cc_v2] = '0'	Reason staging CT not performed at recurrence diagnosis (select all that apply)	checkbox<br>1 ct_no_cc_v2___1 No access to CT machine<br>2 ct_no_cc_v2___2 Patient unable to afford<br>4 ct_no_cc_v2___4 Patient refused<br>5 ct_no_cc_v2___5 Other imaging performed<br>6 ct_no_cc_v2___6 Allergic reaction to contrast<br>7 ct_no_cc_v2___7 Chronic kidney disease<br>3 ct_no_cc_v2___3 Other
1379	[petct_cc_v2]	PET-CT performed at recurrence diagnosis?	yesno<br>1 Yes<br>0 No
1380	[ps_ct_no_cc_v2]<br><br>Show the field ONLY if:<br>[ct_no_cc_v2(3)] = '1'	Please state	text

1381	[petct_find_cc_v2]<br>Show the field ONLY if:<br>[petct_cc_v2] = '1'	PET-CT findings at recurrence diagnosis (select all that apply)	checkbox<br>1 petct_find_cc_v2___1 Avid-FDG uptake vagina (upper two thirds)<br>2 petct_find_cc_v2___2 Avid-FDG uptake vaginal (lower third)<br>3 petct_find_cc_v2___3 Avid-FDG uptake parametrium<br>4 petct_find_cc_v2___4 Avid-FDG uptake uterus<br>5 petct_find_cc_v2___5 Avid-FDG uptake ovary(s)<br>6 petct_find_cc_v2___6 Avid-FDG uptake psoas major<br>7 petct_find_cc_v2___7 Avid-FDG uptake obturator internus<br>8 petct_find_cc_v2___8 Avid-FDG uptake sciatic nerve<br>9 petct_find_cc_v2___9 Avid-FDG uptake urethra<br>10 petct_find_cc_v2___10 Avid-FDG uptake ureter(s)<br>11 petct_find_cc_v2___11 Avid-FDG uptake bladder<br>12 petct_find_cc_v2___12 Avid-FDG uptake kidney(s)<br>13 petct_find_cc_v2___13 Avid-FDG uptake rectum<br>14 petct_find_cc_v2___14 Avid-FDG uptake sigmoid<br>15 petct_find_cc_v2___15 Avid-FDG uptake pelvic lymph nodes<br>16 petct_find_cc_v2___16 Avid-FDG uptake para-aortic lymph nodes<br>17 petct_find_cc_v2___17 Other
1382	[ps_petct_find_cc_v2]<br>Show the field ONLY if:<br>[petct_find_cc_v2(17)] = '1'	Please state	text
1383	[petct_no_cc_v2]<br>Show the field ONLY if:<br>[petct_cc_v2] = '0'	Reason PET-CT not performed at recurrence diagnosis (select all that apply)	checkbox<br>1 petct_no_cc_v2___1 No access to PET machine<br>2 petct_no_cc_v2___2 Patient unable to afford<br>5 petct_no_cc_v2___5 Patient refused<br>6 petct_no_cc_v2___6 Uncontrolled diabetes mellitus<br>4 petct_no_cc_v2___4 Other imaging performed<br>7 petct_no_cc_v2___7 Not clinical protocol<br>3 petct_no_cc_v2___3 Other
1384	[ps_petct_no_cc_v2]<br>Show the field ONLY if:<br>[petct_no_cc_v2(3)] = '1'	Please state	text
1385	[cov19_cc_v2]	COVID-19 status	dropdown<br>1 Positive<br>2 Negative<br>3 Not tested

1386	[cov19_no_cc_v2]<br>Show the field ONLY if:<br>[cov19_cc_v2] = '3'	If not tested select reason (select all that apply)	checkbox<br>1 cov19_no_cc_v2___1 Not clinically indicated (asymptomatic)<br>2 cov19_no_cc_v2___2 Not clinical protocol to routinely test all patients prior to surgery<br>3 cov19_no_cc_v2___3 Patient refused<br>4 cov19_no_cc_v2___4 Patient unable to afford test<br>5 cov19_no_cc_v2___5 Testing not locally available<br>6 cov19_no_cc_v2___6 Other
1387	[bp_cc2]	Bowel preparation pre-operatively	yesno<br>1 Yes<br>0 No
1388	[ps_cov19_no_v2]<br>Show the field ONLY if:<br>[cov19_no_cc_v2(6)] = '1'	Please state	text
1389	[peri_op_cc2]	Peri-operative management plan in place? (Non- exhaustive list: pre-operative haemoglobin optimisation and iron deficit correction; correction of denutrition and immunonutrition; fluid management; pain management; prevention of post-operative nausea and vomiting).	yesno<br>1 Yes<br>0 No
1390	[hb_cc2]	Pre-operative Haemoglobin (g/L)	text (number)
1391	[wcc_cc2]	Pre-operative White Cell Count (10^9/L)	text (number)
1392	[plt_cc2]	Pre-operative Platelets (10^9/L)	text (number)
1393	[pt_cc2]	Pre-operative PT (Secs)	text (number)
1394	[appt_cc2]	Pre-operative APPT (Secs)	text (number)
1395	[inr_cc2]	Pre-operative INR (Ratio)	text (number)
1396	[fib_cc2]	Pre-operative Fibrinogen (g/L)	text (number)
1397	[alb_cc2]	Pre-operative Albumin (g/L)	text (number)
1398	[na_cc2]	Pre-operative Sodium (mmol/L)	text (number)
1399	[k_cc2]	Pre-operative Potassium (mmol/L)	text (number)
1400	[ur_cc2]	Pre-operative Urea (mmol/L)	text (number)
1401	[creat_cc2]	Pre-operative Creatinine (µmol/L)	text (number)
1402	[egfr_cc2]	Pre-operative eGFR - estimated glomerular filtration rate (mL/min)	text
1403	[bili_cc2]	Pre-operative Bilirubin (µmol/L)	text (number)
1404	[aat_cc2]	Pre-operative Alanine Aminotransaminase (IU/L)	text (number)
1405	[alp_cc2]	Pre-operative Alkaline Phosphatase (IU/L)	text (number)
1406	[ast_cc2]	Pre-operative Aspartate Aminotransaminase (IU/L)	text (number)
1407	[crp_cc2]	Pre-operative C-RP - C-reactive protein (IU/L)	text (number)
1408	[datsurg_cc_v2]	Section Header: *SURGERY*<br>Date of recurrence surgery	text (date_dmy)
1409	[anaes_cc_v2]	Anaesthesia (select all that apply)	checkbox<br>1 anaes_cc_v2___1 General<br>2 anaes_cc_v2___2 Epidural<br>3 anaes_cc_v2___3 Spinal
1410	[surg_cc_v2]	Was recurrence surgery an elective or emergency case?	radio<br>1 Elective<br>2 Emergency
1411	[sa_cc_v2]	Surgical aim	dropdown<br>1 Curative<br>2 Curative attempted but then abandoned (i.e. open/close laparotomy)<br>3 Palliative

1412	[sm_cc_v2]	Surgical modality	dropdown<br>1 Laparotomy<br>2 Laparoscopic<br>3 Robotic<br>6 Vaginal<br>4 Laparoscopic converted to laparotomy<br>5 Robotic converted to laparotomy<br>7 Vaginal converted to laparotomy
1413	[ls_cc_v2]	Lead surgeon performing recurrence surgery	dropdown<br>1 Certified consultant/specialist gynaecological oncologist<br>2 Fellow/trainee gynaecological oncologist<br>3 Trained surgeon dedicated to the management of gynaecologic cancer (accounting for more than 80% of his/her practice) or having completed an ESGO accredited fellowship.<br>4 General surgeon<br>5 Surgical oncologist<br>6 Other
1414	[ps_ls_cc_v2]<br>Show the field ONLY if:<br>[ls_cc_v2] = '6'	Please state	text
1415	[who_cc2]	WHO surgical safety checklist used	yesno<br>1 Yes<br>0 No
1416	[cyto_cc2]	Complete macroscopic cytoreduction at recurrence surgery	yesno<br>1 Yes<br>0 No
1417	[reas_cytored_no_cc2]<br>Show the field ONLY if:<br>[cyto_cc2] = '0'	Reason(s) cytoreduction not achieved at recurrence surgery (select all that apply)	checkbox<br>1 reas_cytored_no_cc2___1 Disease more extensive than that shown on imaging<br>2 reas_cytored_no_cc2___2 Technically unable to resect<br>3 reas_cytored_no_cc2___3 Patient became medically unstable intra-operatively and decision made to not proceed further with surgery<br>4 reas_cytored_no_cc2___4 Other
1418	[ps_reas_cytored_no_cc2]<br>Show the field ONLY if:<br>[reas_cytored_no_cc2(4)] = '1'	Please state	text
1419	[siz_resid_cc2]<br>Show the field ONLY if:<br>[cyto_cc2] = '0'	Size of residual disease	dropdown<br>1 1-2.5<br>2 2.6-5<br>3 5.1-7.5<br>4 7.6-10<br>5 >10

1420	[loc_resid_cc2]<br>Show the field ONLY if:<br>[cyto_cc2] = '0'	Location of residual disease (select all that apply)	checkbox<br>1 loc_resid_cc2___1 Vagina (upper two thirds)<br>2 loc_resid_cc2___2 Vagina (lower third)<br>3 loc_resid_cc2___3 Parametrium<br>4 loc_resid_cc2___4 Uterus<br>5 loc_resid_cc2___5 fallopian tube(s)<br>6 loc_resid_cc2___6 ovary(s)<br>7 loc_resid_cc2___7 Psoas major<br>8 loc_resid_cc2___8 Obturator internus<br>9 loc_resid_cc2___9 Sciatic nerve<br>10 loc_resid_cc2___10 Ureter(s)<br>11 loc_resid_cc2___11 Bladder<br>12 loc_resid_cc2___12 Rectum<br>13 loc_resid_cc2___13 Sigmoid<br>14 loc_resid_cc2___14 Ureter(s)<br>15 loc_resid_cc2___15 Kidney(s)<br>16 loc_resid_cc2___16 Urethra<br>17 loc_resid_cc2___17 Pelvic lymphadenopathy<br>18 loc_resid_cc2___18 Para-aortic lymphadenopathy<br>19 loc_resid_cc2___19 Other
1421	[ps_loc_resid_cc2]<br>Show the field ONLY if:<br>[loc_resid_cc2(19)] = '1'	Please state	text
1422	[surgfind_cc_v2]	Findings at recurrence surgery (select all that apply)	checkbox<br>1 surgfind_cc_v2___1 Vaginal invasion (upper two thirds)<br>2 surgfind_cc_v2___2 Vaginal invasion (lower third)<br>3 surgfind_cc_v2___3 Parametrial invasion<br>4 surgfind_cc_v2___4 Uterus invasion<br>5 surgfind_cc_v2___5 x1 tubo-ovarian invasion<br>6 surgfind_cc_v2___6 x2 tubo-ovarian invasion<br>7 surgfind_cc_v2___7 Psoas major invasion<br>8 surgfind_cc_v2___8 Obturator internus invasion<br>9 surgfind_cc_v2___9 Sciatic nerve invasion<br>10 surgfind_cc_v2___10 Ureter invasion<br>11 surgfind_cc_v2___11 Bladder invasion<br>12 surgfind_cc_v2___12 Rectal invasion<br>13 surgfind_cc_v2___13 Sigmoid invasion<br>14 surgfind_cc_v2___14 x1 hydroureter<br>15 surgfind_cc_v2___15 x2 hydroureter<br>16 surgfind_cc_v2___16 Ureteric invasion<br>17 surgfind_cc_v2___17 x1 hydronephrosis<br>18 surgfind_cc_v2___18 x2 hydronephrosis<br>19 surgfind_cc_v2___19 Urethral invasion<br>20 surgfind_cc_v2___20 Pelvic lymphadenopathy<br>21 surgfind_cc_v2___21 Para-aortic lymphadenopathy<br>22 surgfind_cc_v2___22 Other<br>23 surgfind_cc_v2___23 No visible disease
1423	[ps_surgfind_cc_v2]<br>Show the field ONLY if:<br>[surgfind_cc_v2(22)] = '1'	Please state	text

1424	[tr_cc_v2]	Tissue removed (select all that apply)	checkbox<br>1 tr_cc_v2___1 Uterus<br>2 tr_cc_v2___2 Parametrium<br>3 tr_cc_v2___3 Upper vagina<br>4 tr_cc_v2___4 x1 fallopian tube<br>5 tr_cc_v2___5 x2 fallopian tubes<br>6 tr_cc_v2___6 x1 ovary<br>7 tr_cc_v2___7 x2 ovaries<br>8 tr_cc_v2___8 Psoas major<br>9 tr_cc_v2___9 Obturator internus<br>10 tr_cc_v2___10 Sciatic nerve<br>11 tr_cc_v2___11 Pelvic lymph nodes<br>12 tr_cc_v2___12 Para-aortic lymph nodes<br>13 tr_cc_v2___13 Rectum<br>14 tr_cc_v2___14 Sigmoid<br>15 tr_cc_v2___15 ureter<br>16 tr_cc_v2___16 kidney<br>17 tr_cc_v2___17 Bladder<br>18 tr_cc_v2___18 Other
1425	[ps_tr_cc_v2]<br>Show the field ONLY if:<br>[tr_cc_v2(18)] = '1'	Please state	text
1426	[paln_dis_cc2]<br>Show the field ONLY if:<br>[tr_cc_v2(12)] = '1'	Select all that apply	checkbox<br>1 paln_dis_cc2___1 Infra-renal<br>2 paln_dis_cc2___2 Supra-renal
1427	[si_stom_cc_v2]	Small bowel stoma	yesno<br>1 Yes<br>0 No
1428	[ss_cc2]<br>Show the field ONLY if:<br>[si_stom_cc_v2] = '1'	Stoma site	dropdown<br>1 Duodenum<br>2 Jejunum<br>3 Ileum
1429	[pb_si_stom_cc_v2]<br>Show the field ONLY if:<br>[si_stom_cc_v2] = '1'	Performed by (select all that apply)	checkbox<br>1 pb_si_stom_cc_v2___1 Gynaecological oncologist<br>2 pb_si_stom_cc_v2___2 Colorectal surgeon<br>3 pb_si_stom_cc_v2___3 Urologist<br>4 pb_si_stom_cc_v2___4 General surgeon<br>5 pb_si_stom_cc_v2___5 Other
1430	[ps_pb_si_stom_cc_v2]<br>Show the field ONLY if:<br>[pb_si_stom_cc_v2(5)] = '1'	Please state	text
1431	[si_anast_cc_v2]	Small bowel anastamosis	yesno<br>1 Yes<br>0 No
1432	[pb_si_anast_cc_v2]<br>Show the field ONLY if:<br>[si_anast_cc_v2] = '1'	Performed by (select all that apply)	checkbox<br>1 pb_si_anast_cc_v2___1 Gynaecological oncologist<br>2 pb_si_anast_cc_v2___2 Colorectal surgeon<br>3 pb_si_anast_cc_v2___3 Urologist<br>4 pb_si_anast_cc_v2___4 General surgeon<br>5 pb_si_anast_cc_v2___5 Other

1433	[ps_pb_si_anast_cc_v2]<br>Show the field ONLY if:<br>[pb_si_anast_cc_v2(5)] = '1'	Please state	text
1434	[li_stom_cc_v2]	Large bowel stoma	yesno<br>1 Yes<br>0 No
1435	[pb_li_stom_cc_v2]<br>Show the field ONLY if:<br>[li_stom_cc_v2] = '1'	Performed by (select all that apply)	checkbox<br>1 pb_li_stom_cc_v2___1 Gynaecological oncologist<br>2 pb_li_stom_cc_v2___2 Colorectal surgeon<br>3 pb_li_stom_cc_v2___3 Urologist<br>4 pb_li_stom_cc_v2___4 General surgeon<br>5 pb_li_stom_cc_v2___5 Other
1436	[ps_pb_li_stom_cc_v2]<br>Show the field ONLY if:<br>[pb_li_stom_cc_v2(5)] = '1'	Please state	text
1437	[li_anas_v2]	Large bowel anastamosis	yesno<br>1 Yes<br>0 No
1438	[pb_li_anast_cc_v2]<br>Show the field ONLY if:<br>[li_anas_v2] = '1'	Performed by (select all that apply)	checkbox<br>1 pb_li_anast_cc_v2___1 Gynaecological oncologist<br>2 pb_li_anast_cc_v2___2 Colorectal surgeon<br>3 pb_li_anast_cc_v2___3 Urologist<br>4 pb_li_anast_cc_v2___4 General surgeon<br>5 pb_li_anast_cc_v2___5 Other
1439	[ps_pb_li_anast_cc_v2]<br>Show the field ONLY if:<br>[pb_li_anast_cc_v2(5)] = '1'	Please state	text
1440	[uros_cc_v2]	Urostomy/ileal conduit	yesno<br>1 Yes<br>0 No
1441	[pb_ic_cc_v2]<br>Show the field ONLY if:<br>[uros_cc_v2] = '1'	Performed by (select all that apply)	checkbox<br>1 pb_ic_cc_v2___1 Gynaecological oncologist<br>2 pb_ic_cc_v2___2 Colorectal surgeon<br>3 pb_ic_cc_v2___3 Urologist<br>4 pb_ic_cc_v2___4 General surgeon<br>5 pb_ic_cc_v2___5 Other
1442	[ps_pb_ic_cc_v2]<br>Show the field ONLY if:<br>[pb_ic_cc_v2(5)] = '1'	Please state	text
1443	[tuu_cc_v2]	Trans uretero-ureteric anastamosis	yesno<br>1 Yes<br>0 No
1444	[pb_tuu_cc_v2]<br>Show the field ONLY if:<br>[tuu_cc_v2] = '1'	Performed by (Select all that apply)	checkbox<br>1 pb_tuu_cc_v2___1 Gynaecological oncologist<br>2 pb_tuu_cc_v2___2 Colorectal surgeon<br>3 pb_tuu_cc_v2___3 Urologist<br>4 pb_tuu_cc_v2___4 General surgeon<br>5 pb_tuu_cc_v2___5 Other
1445	[ps_pb_tuu_cc_v2]<br>Show the field ONLY if:<br>[pb_tuu_cc_v2(5)] = '1'	Please state	text

1446	[ui_cc_v2]	Ureteric reimplantation	yesno<br>1 Yes<br>0 No
1447	[pb_ui_cc_v2]<br>Show the field ONLY if:<br>[ui_cc_v2] = '1'	Performed by (select all that apply)	checkbox<br>1 pb_ui_cc_v2___1 Gynaecological oncologist<br>2 pb_ui_cc_v2___2 Colorectal surgeon<br>3 pb_ui_cc_v2___3 Urologist<br>4 pb_ui_cc_v2___4 General surgeon<br>5 pb_ui_cc_v2___5 Other
1448	[ps_pb_ui_cc_v2]<br>Show the field ONLY if:<br>[pb_ui_cc_v2(5)] = '1'	Please state	text
1449	[neph_cc_v2]	Nephrostomy	yesno<br>1 Yes<br>0 No
1450	[surg2_cc_v2]<br>Show the field ONLY if:<br>[neph_cc_v2] = '1'	Performed by (select all that apply)	checkbox<br>1 surg2_cc_v2___1 Gynaecological oncologist<br>2 surg2_cc_v2___2 Colorectal surgeon<br>3 surg2_cc_v2___3 Urologist<br>4 surg2_cc_v2___4 General surgeon<br>5 surg2_cc_v2___5 Other
1451	[ps_surg2_cc_v2]<br>Show the field ONLY if:<br>[surg2_cc_v2(5)] = '1'	Please state	text
1452	[abx_cc2]	Intraoperative antibiotics	yesno<br>1 Yes<br>0 No
1453	[time_cc_v2]	Length of recurrence surgery	text (time)
1454	[itu_cc2]	Section Header: *POST-OPERATIVE*<br>Post-operative recovery in intensive care unit	yesno<br>1 Yes<br>0 No
1455	[itu_dys_cc2]<br>Show the field ONLY if:<br>[itu_cc2] = '1'	Number of days in intensive care unit	text (number)
1456	[hdu_cc2]	Post-operative recovery in high dependency unit	yesno<br>1 Yes<br>0 No
1457	[hdu_dys_cc2]<br>Show the field ONLY if:<br>[hdu_cc2] = '1'	Number of days in high dependency unit	text (number)
1458	[erec_cc_v2]	Enhanced recovery?	yesno<br>1 Yes<br>0 No
1459	[po_abx_cc2]	Prophylactic post operative antibiotics	yesno<br>1 Yes<br>0 No
1460	[drn_cc2]	Post operative surgical drain	yesno<br>1 Yes<br>0 No
1461	[drn_dys_cc2]<br>Show the field ONLY if:<br>[drn_cc2] = '1'	Number of days in situ	text (number)

1462	[uc_cc2]	Post operative urinary catheter	yesno<br>1 Yes<br>0 No
1463	[cath_dys_cc2]<br>Show the field ONLY if:<br>[uc_cc2] = '1'	Number of days in situ	text (number)
1464	[phb_cc_2]	Post-operative Haemoglobin (g/L) - please state first post-operative result	text (number)
1465	[pwbc_cc_2]	Post-operative White Cell Count (10^9/L) - please state first post-operative result	text (number)
1466	[pplat_cc_2]	Post-operative Platelets (10^9/L) - please state first post-operative result	text (number)
1467	[ppt_cc_2]	Post-operative PT (Secs) - please state first post-operative result	text (number)
1468	[pinr_cc_2]	Post-operative INR (Ratio) - please state first post-operative result	text (number)
1469	[paptt_cc_2]	Post-operative APTT (Secs) - please state first post-operative result	text (number)
1470	[pfib_cc_2]	Post-operative Fibrinogen (g/L) - please state first post-operative result	text (number)
1471	[palb_cc_2]	Post-operative Albumin (g/L) - please state first post-operative result	text (number)
1472	[pna_cc_2]	Post-operative Sodium (mmol/L) - please state first post-operative result	text (number)
1473	[pk_cc_2]	Post-operative Potassium (mmol/L) - please state first post-operative result	text (number)
1474	[purea_cc_2]	Post-operative Urea (mmol/L) - please state first post-operative result	text (number)
1475	[pcreat_cc_2]	Post-operative Creatinine (μmol/L) - please state first post-operative result	text
1476	[pegfr_cc_2]	Post-operative eGFR - Estimated Glomerular Filtration Rate (mL/min) - please state first post-operative result	text
1477	[pbili_cc_2]	Post-operative Bilirubin total (μmol/L) - please state first post-operative result	text (number)
1478	[paat_cc_2]	Post-operative Alanine Aminotransaminase (IU/L) - please state first post-operative result	text (number)
1479	[palp_cc_2]	Post-operative Alkaline Phosphatase (IU/L) - please state first post-operative result	text (number)
1480	[past_cc_2]	Post-operative Aspartate Aminotransaminase (IU/L) - please state first post-operative result	text (number)
1481	[pcrp_cc_2]	Post-operative CRP - C-reactive protein (mg/L) - please state first post-operative result	text (number)
1482	[hist_cc_v2]	Histologic type	dropdown<br>1 Squamous cell<br>2 Adenocarcinoma (glandular)<br>3 Adenosquamous<br>4 Mesenchymal<br>5 Mixed epithelial and mesenchymal<br>6 Melanocytic<br>7 Lymphoid<br>8 Small cell undifferentiated (neuroendocrine)
1483	[resect_cc2]	Histological confirmed resection margins following recurrence surgery	dropdown<br>1 < 1 mm / tumour on margin<br>2 1-5 mm (NO tumour on margin)<br>3 >5 mm Margins confirmed clear<br>4 Unknown, not available in this hospital

1484	[plnd_cc_v2]	Pelvic lymph nodes positive	dropdown<br>1 Yes<br>2 No<br>3 Not applicable - not removed
1485	[p16_cc_v2]	Section Header: *Immunohistochemistry*<br>p16	radio (Matrix)<br>1 Positive<br>2 Negative<br>3 Not tested
1486	[m1bi_cc_v2]	M1B1	radio (Matrix)<br>1 Positive<br>2 Negative<br>3 Not tested
1487	[ck20_cc_v2]	CK20	radio (Matrix)<br>1 Positive<br>2 Negative<br>3 Not tested
1488	[bcl2_cc_v2]	bcl-2	radio (Matrix)<br>1 Positive<br>2 Negative<br>3 Not tested
1489	[cea_cc_v2]	monoclonal CEA	radio (Matrix)<br>1 Positive<br>2 Negative<br>3 Not tested
1490	[vim_cc_v2]	Vimentin	radio (Matrix)<br>1 Positive<br>2 Negative<br>3 Not tested
1491	[er_cc_v2]	Oestrogen receptor (ER)	radio (Matrix)<br>1 Positive<br>2 Negative<br>3 Not tested
1492	[cd10_cc_v2]	CD10	radio (Matrix)<br>1 Positive<br>2 Negative<br>3 Not tested
1493	[ar_cc_v2]	Androgen receptor	radio (Matrix)<br>1 Positive<br>2 Negative<br>3 Not tested
1494	[cal_cc_v2]	Calretinin	radio (Matrix)<br>1 Positive<br>2 Negative<br>3 Not tested
1495	[inh_cc_v2]	Inhibin	radio (Matrix)<br>1 Positive<br>2 Negative<br>3 Not tested
1496	[pr_cc_v2]	Progesterone receptor (PR)	radio (Matrix)<br>1 Positive<br>2 Negative<br>3 Not tested

1497	[hik108_cc_v2]	HIK108	radio (Matrix)<br>1 Positive<br>2 Negative<br>3 Not tested
1498	[asma_cc_v2]	a-SMA	radio (Matrix)<br>1 Positive<br>2 Negative<br>3 Not tested
1499	[abpas_cc_v2]	alcian blue/periodic acid-Schiff (AB/PAS) stain	radio (Matrix)<br>1 Positive<br>2 Negative<br>3 Not tested
1500	[chrom_cc_v2]	chromogranin	radio (Matrix)<br>1 Positive<br>2 Negative<br>3 Not tested
1501	[synap_cc_v2]	synaptophysin	radio (Matrix)<br>1 Positive<br>2 Negative<br>3 Not tested
1502	[pgp_cc_v2]	PGP9.5	radio (Matrix)<br>1 Positive<br>2 Negative<br>3 Not tested
1503	[cd56_cc_v2]	CD56	radio (Matrix)<br>1 Positive<br>2 Negative<br>3 Not tested
1504	[ttf1_cc_v2]	TTF1	radio (Matrix)<br>1 Positive<br>2 Negative<br>3 Not tested
1505	[cd99_cc_v2]	CD99	radio (Matrix)<br>1 Positive<br>2 Negative<br>3 Not tested
1506	[adjchemo_cc_v2]	Chemotherapy administered following recurrence diagnosis	yesno<br>1 Yes<br>0 No
1507	[adjchemo1_cc_v2]<br>Show the field ONLY if:<br>[adjchemo_cc_v2] = '1'	Chemotherapy regimen (select all that apply)	checkbox<br>1 adjchemo1_cc_v2___1 Platinum (for example cisplatin, carboplatin)<br>2 adjchemo1_cc_v2___2 Fluorouracil<br>3 adjchemo1_cc_v2___3 Taxane (for example paclitaxel)<br>4 adjchemo1_cc_v2___4 Bevacuzimab<br>5 adjchemo1_cc_v2___5 Topotecan<br>6 adjchemo1_cc_v2___6 Gemcitabine<br>7 adjchemo1_cc_v2___7 Clinical trial regimens<br>8 adjchemo1_cc_v2___8 Other
1508	[ps_adjchemo1_cc_v2]<br>Show the field ONLY if:<br>[adjchemo1_cc_v2(8)] = '1'	Please state	text

1509	[adjchem1_cyc_cc_v2]<br>Show the field ONLY if:<br>[adjchemo_cc_v2] = '1'	Number of cycles planned	text (number)
1510	[adjchemo_no_cc_v2]<br>Show the field ONLY if:<br>[adjchemo_cc_v2] = '0'	Reason chemotherapy not administered following recurrence diagnosis (select all that apply)	checkbox<br>5 adjchemo_no_cc_v2___5 Poor performance status<br>1 adjchemo_no_cc_v2___1 No access<br>2 adjchemo_no_cc_v2___2 Patient unable to afford<br>4 adjchemo_no_cc_v2___4 Patient refused<br>6 adjchemo_no_cc_v2___6 Not clinically indicated<br>3 adjchemo_no_cc_v2___3 Other
1511	[ps_adjchemo_no_cc_v2]<br>Show the field ONLY if:<br>[adjchemo_no_cc_v2(3)] = '1'	Please state	text
1512	[rt_cc_v2]	External beam radiotherapy administered following recurrence diagnosis	yesno<br>1 Yes<br>0 No
1513	[rt_dos_cc2]<br>Show the field ONLY if:<br>[rt_cc_v2] = '1'	Dose administered (Gray - Gy)	text (number)
1514	[rt_cc_no_v2]<br>Show the field ONLY if:<br>[rt_cc_v2] = '0'	Reason external beam radiotherapy not administered following recurrence diagnosis (select all that apply)	checkbox<br>1 rt_cc_no_v2___1 Poor performance status<br>2 rt_cc_no_v2___2 Not available locally<br>3 rt_cc_no_v2___3 Patient unable to afford<br>4 rt_cc_no_v2___4 Patient refused<br>5 rt_cc_no_v2___5 Not clinically indicated<br>6 rt_cc_no_v2___6 Fistula (urinary/rectal)<br>7 rt_cc_no_v2___7 Other
1515	[ps_rt_cc_no_v2]<br>Show the field ONLY if:<br>[rt_cc_no_v2(7)] = '1'	Please state	text
1516	[bt_cc_v2]	Brachytherapy administered following recurrence diagnosis	yesno<br>1 Yes<br>0 No
1517	[rt_dos_cc3]<br>Show the field ONLY if:<br>[bt_cc_v2] = '1'	Dose administered (Gray - Gy)	text (number)
1518	[bt_no_cc_v2]<br>Show the field ONLY if:<br>[bt_cc_v2] = '0'	Reason brachytherapy not administered following recurrence diagnosis (Select all that apply)	checkbox<br>1 bt_no_cc_v2___1 Poor performance status<br>2 bt_no_cc_v2___2 Not available locally<br>3 bt_no_cc_v2___3 Patient unable to afford<br>4 bt_no_cc_v2___4 Patient refused<br>5 bt_no_cc_v2___5 Not clinically indicated<br>6 bt_no_cc_v2___6 Other
1519	[ps_bt_no_cc_v2]<br>Show the field ONLY if:<br>[bt_no_cc_v2(6)] = '1'	Please state	text
1520	[cervical_cancer_recurrence_complete]	Section Header: *Form Status*<br>Complete?	dropdown<br>0 Incomplete<br>1 Unverified<br>2 Complete
Instrument: **Vulval cancer (initial presentation)** (vulval_cancer_initial_presentation)			
1521	[dat_vu]	Section Header: *BASELINE DEMOGRAPHICS*<br>Date of primary diagnosis	text (date_dmy)

1522	[age_vu]	Age of patient	text (number)
1523	[eth_vu]	Ethnicity	dropdown<br>1 White (British, Australian, North American, European, other white)<br>2 Asian (South East Asian, Far East Asian, other Asian)<br>3 Black (African, Caribbean, other Black)<br>4 Middle Eastern (Arab, North African, Turkish)<br>5 Mixed (multiple ethnic background)<br>6 Other
1524	[ps_eth_vu]<br>Show the field ONLY if:<br>[eth_vu] = '6'	Please state	text
1525	[bmi_vu]	Body mass index (kg/m2)	text (number)
1526	[smok_vu]	Smoker	dropdown<br>1 Yes<br>2 No<br>3 Ex-smoker
1527	[hpv_vu]	HPV status	dropdown<br>1 Positive<br>2 Negative<br>3 Not tested
1528	[hpv_typ_vu]<br>Show the field ONLY if:<br>[hpv_vu] = '1'	HPV type (select all that apply)	checkbox<br>16 hpv_typ_vu___16 16<br>18 hpv_typ_vu___18 18<br>1 hpv_typ_vu___1 other<br>19 hpv_typ_vu___19 Unknown
1529	[hpv_no_vu]<br>Show the field ONLY if:<br>[hpv_vu] = '3'	Reason not tested (select all that apply)	checkbox<br>1 hpv_no_vu___1 Not clinical protocol<br>2 hpv_no_vu___2 Patient refused<br>3 hpv_no_vu___3 Patient unable to afford test<br>4 hpv_no_vu___4 Testing not available locally<br>5 hpv_no_vu___5 Other
1530	[ps_hpv_no_vu]<br>Show the field ONLY if:<br>[hpv_no_vu(5)] = '1'	Please state	text
1531	[vac_vu]	HPV vaccine administered historically	yesno<br>1 Yes<br>0 No
1532	[vac_typ_vu]<br>Show the field ONLY if:<br>[vac_vu] = '1'	Type of vaccine administered	dropdown<br>1 Bivalent<br>2 Quadrivalent<br>3 9-valent
1533	[age_vac_vu]<br>Show the field ONLY if:<br>[vac_vu] = '1'	Age vaccine administered	text (number)
1534	[vac_no_reas_vu]<br>Show the field ONLY if:<br>[vac_vu] = '0'	Reason not administered (select all that apply)	checkbox<br>1 vac_no_reas_vu___1 No national vaccination programme<br>2 vac_no_reas_vu___2 National vaccination programme introduced after age of first sexual activity<br>3 vac_no_reas_vu___3 Patient unable to afford<br>4 vac_no_reas_vu___4 Other

1535	[ps_vac_no_reas_vu]<br>Show the field ONLY if:<br>[vac_no_reas_vu(4)] = '1'	Please state	text
1536	[asa_vu]	Section Header: *PRE-OPERATIVE*<br>American Society of Anesthesiologists (ASA) physical status	dropdown<br>1 ASA 1: A normal healthy patient. Example: Fit, non-obese (BMI under 30), a nonsmoking patient with good exercise tolerance.<br>2 ASA 2: A patient with a mild systemic disease. Example: Patient with no functional limitations and a well-controlled disease (e.g., treated hypertension, obesity with BMI under 35, frequent social drinker or is a cigarette smoker).<br>3 ASA 3: A patient with a severe systemic disease that is not life-threatening. Example: Patient with some functional limitation as a result of disease (e.g., poorly treated hypertension or diabetes, morbid obesity, chronic renal failure, a bronchospastic disease with intermittent exacerbation, stable angina, implanted pacemaker).<br>4 ASA 4: A patient with a severe systemic disease that is a constant threat to life. Example: Patient with functional limitation from severe, life-threatening disease (e.g., unstable angina, poorly controlled COPD, symptomatic CHF, recent (less than three months ago) myocardial infarction or stroke.<br>5 ASA 5: A moribund patient who is not expected to survive without the operation. The patient is not expected to survive beyond the next 24 hours without surgery. Examples: ruptured abdominal aortic aneurysm, massive trauma, and extensive intracranial hemorrhage with mass effect.<br>6 ASA 6: A brain-dead patient whose organs are being removed with the intention of transplanting them into another patient.
1537	[ecog_vu]	ECOG (Eastern Cooperative Oncology Group) performance status	dropdown<br>1 Grade 0 - Fully active, able to carry on all pre-disease performance without restriction<br>2 Grade 1 - Restricted in physically strenuous activity but ambulatory and able to carry out work of a light or sedentary nature, e.g., light house work, office work<br>3 Grade 2 - Ambulatory and capable of all selfcare but unable to carry out any work activities; up and about more than 50% of waking hours<br>4 Grade 3 - Capable of only limited selfcare; confined to bed or chair more than 50% of waking hours<br>5 Grade 4 - Completely disabled; cannot carry on any selfcare; totally confined to bed or chair<br>6 Grade 5 - Dead
1538	[comorb_vu]	Co-morbidities	checkbox<br>1 comorb_vu___1 Cardiovascular<br>2 comorb_vu___2 Respiratory<br>3 comorb_vu___3 Urogenital<br>4 comorb_vu___4 Endocrine<br>5 comorb_vu___5 Neurological<br>6 comorb_vu___6 Haematological<br>7 comorb_vu___7 Psychiatric<br>8 comorb_vu___8 Gastroenterology<br>9 comorb_vu___9 Rheumatology and musculoskeletal<br>10 comorb_vu___10 Oncology<br>11 comorb_vu___11 None

1539	[morb_cvd_vu]<br>Show the field ONLY if:<br>[comorb_vu(1)] = '1'	Select all that apply	checkbox<br>1 morb_cvd_vu___1 Hypertension<br>2 morb_cvd_vu___2 Coronary heart disease / ischaemic heart disease<br>3 morb_cvd_vu___3 Myocardial infarction<br>4 morb_cvd_vu___4 Heart failure<br>5 morb_cvd_vu___5 Arrhythmia<br>6 morb_cvd_vu___6 Heart valve disease<br>7 morb_cvd_vu___7 Venous insufficiency<br>8 morb_cvd_vu___8 Peripheral vascular disease<br>9 morb_cvd_vu___9 Stroke<br>10 morb_cvd_vu___10 Transient ischaemic attack<br>11 morb_cvd_vu___11 Other
1540	[comorb_resp_vu]<br>Show the field ONLY if:<br>[comorb_vu(2)] = '1'	Select all that apply	checkbox<br>1 comorb_resp_vu___1 Asthma<br>2 comorb_resp_vu___2 Emphysema (COPD)<br>3 comorb_resp_vu___3 Chronic bronchitis (COPD)<br>4 comorb_resp_vu___4 Pulmonary fibrosis<br>5 comorb_resp_vu___5 Sleep apnoea<br>6 comorb_resp_vu___6 Bronchiectasis<br>7 comorb_resp_vu___7 Cystic fibrosis<br>8 comorb_resp_vu___8 Tuberculosis<br>9 comorb_resp_vu___9 Pneumonia<br>10 comorb_resp_vu___10 Other
1541	[comorb_uro_vu]<br>Show the field ONLY if:<br>[comorb_vu(3)] = '1'	Select all that apply	checkbox<br>1 comorb_uro_vu___1 Chronic kidney disease (not requiring haemodialysis)<br>2 comorb_uro_vu___2 Chronic kidney disease (requiring haemodialysis)<br>3 comorb_uro_vu___3 Polycystic kidney disease<br>4 comorb_uro_vu___4 Glomerulonephropathy<br>5 comorb_uro_vu___5 Glomerulonephritis<br>6 comorb_uro_vu___6 Urinary tract infection<br>7 comorb_uro_vu___7 Other
1542	[comorb_endo_vu]<br>Show the field ONLY if:<br>[comorb_vu(4)] = '1'	Select all that apply	checkbox<br>1 comorb_endo_vu___1 Type 1 diabetes mellitus<br>2 comorb_endo_vu___2 Type 2 diabetes mellitus<br>3 comorb_endo_vu___3 Hyperthyroidism<br>4 comorb_endo_vu___4 Hypothyroidism<br>5 comorb_endo_vu___5 Other
1543	[comorb_neuro_vu]<br>Show the field ONLY if:<br>[comorb_vu(5)] = '1'	Select all that apply	checkbox<br>1 comorb_neuro_vu___1 Parkinsons disease<br>2 comorb_neuro_vu___2 Myasthenia gravis<br>3 comorb_neuro_vu___3 Dementia<br>4 comorb_neuro_vu___4 Epilepsy<br>5 comorb_neuro_vu___5 Other

1544	[comorb_haem_vu]<br>Show the field ONLY if:<br>[comorb_vu(6)] = '1'	Select all that apply	checkbox<br>1 comorb_haem_vu___1 Iron deficiency anaemia<br>2 comorb_haem_vu___2 Folate deficience anaemia<br>3 comorb_haem_vu___3 Vitamin B12 deficiency anaemia<br>4 comorb_haem_vu___4 Pernicious anaemia<br>5 comorb_haem_vu___5 Haemolytic anaemia<br>6 comorb_haem_vu___6 Aplastic anaemia<br>7 comorb_haem_vu___7 Thalassemia<br>8 comorb_haem_vu___8 Sickle cell disease<br>9 comorb_haem_vu___9 Disseminated intravascular coagulation (DIC)<br>10 comorb_haem_vu___10 Factor V Leiden<br>11 comorb_haem_vu___11 Thrombocytosis<br>12 comorb_haem_vu___12 Idiopathic thrombocytopenic purpura (ITP)<br>13 comorb_haem_vu___13 Haemophilia<br>14 comorb_haem_vu___14 Von Willebrand disease<br>15 comorb_haem_vu___15 Antiphospholipid syndrome<br>16 comorb_haem_vu___16 Thrombocytopenia<br>17 comorb_haem_vu___17 Thrombotic thrombocytopenic purpura<br>18 comorb_haem_vu___18 Deep vein thrombosis<br>19 comorb_haem_vu___19 Pulmonary embolism<br>20 comorb_haem_vu___20 Other
1545	[comorb_pscy_vu]<br>Show the field ONLY if:<br>[comorb_vu(7)] = '1'	Select all that apply	checkbox<br>1 comorb_pscy_vu___1 Anxiety<br>2 comorb_pscy_vu___2 Depression<br>3 comorb_pscy_vu___3 Learning difficulties<br>4 comorb_pscy_vu___4 Personality disorder<br>5 comorb_pscy_vu___5 Schizophrenia<br>6 comorb_pscy_vu___6 Bipolar disorder<br>7 comorb_pscy_vu___7 Autism<br>8 comorb_pscy_vu___8 Other
1546	[comorb_gi_vu]<br>Show the field ONLY if:<br>[comorb_vu(8)] = '1'	Select all that apply	checkbox<br>1 comorb_gi_vu___1 Crohn's disease<br>2 comorb_gi_vu___2 Ulcerative colitis<br>3 comorb_gi_vu___3 Irritable bowel syndrome<br>4 comorb_gi_vu___4 Celiac Disease<br>5 comorb_gi_vu___5 Peptic ulcer disease<br>6 comorb_gi_vu___6 Gallstones<br>7 comorb_gi_vu___7 Pancreatitis<br>8 comorb_gi_vu___8 Diverticulitis<br>9 comorb_gi_vu___9 Non-Alcoholic Fatty Liver Disease<br>10 comorb_gi_vu___10 Alcoholic fatty liver disease<br>11 comorb_gi_vu___11 Viral hepatitis<br>12 comorb_gi_vu___12 Liver failure<br>13 comorb_gi_vu___13 Other

1547	[comorb_msk_vu]<br>Show the field ONLY if:<br>[comorb_vu(9)] = '1'	Select all that apply	checkbox<br>1 comorb_msk_vu___1 Osteoarthritis<br>2 comorb_msk_vu___2 Rheumatoid arthritis<br>3 comorb_msk_vu___3 Osteoporosis<br>4 comorb_msk_vu___4 Lupus<br>5 comorb_msk_vu___5 Spondyloarthropathies<br>6 comorb_msk_vu___6 Sjogren's syndrome<br>7 comorb_msk_vu___7 Gout<br>8 comorb_msk_vu___8 Other
1548	[comorb_onc_vu]<br>Show the field ONLY if:<br>[comorb_vu(10)] = '1'	Select all that apply	checkbox<br>1 comorb_onc_vu___1 Breast cancer<br>2 comorb_onc_vu___2 Lung cancer<br>3 comorb_onc_vu___3 Hepatobiliary cancer<br>4 comorb_onc_vu___4 Brain cancer<br>5 comorb_onc_vu___5 Leukemia<br>6 comorb_onc_vu___6 Lymphoma<br>7 comorb_onc_vu___7 Osteosarcoma<br>8 comorb_onc_vu___8 Other
1549	[lap_vu]	Previous laparoscopic abdominal surgery	yesno<br>1 Yes<br>0 No
1550	[opn_vu]	Previous laparotomy	yesno<br>1 Yes<br>0 No
1551	[mdt_vu]	Discussed at multidisciplinary team meeting/tumour board?	yesno<br>1 Yes<br>0 No
1552	[mdt_tm_vu]<br>Show the field ONLY if:<br>[mdt_vu] = '1'	Multidisciplinary team/tumour board team members (select all that apply)	checkbox<br>1 mdt_tm_vu___1 Certified gynaecological oncology surgeon<br>2 mdt_tm_vu___2 Trained surgeon dedicated to the management of gynaecologic cancer or having completed an ESGO-accredited fellowship<br>3 mdt_tm_vu___3 Radiologist<br>4 mdt_tm_vu___4 Radiation/medical oncologist (administering radiotherapy)<br>5 mdt_tm_vu___5 Medical oncologist (administering chemotherapy)<br>6 mdt_tm_vu___6 Pathologist
1553	[mdt_vu_no]<br>Show the field ONLY if:<br>[mdt_vu] = '0'	Reason not discussed (Select all that apply)	checkbox<br>1 mdt_vu_no___1 No multidisciplinary team meeting/tumour board<br>2 mdt_vu_no___2 Other
1554	[ps_mdt_vc_no]<br>Show the field ONLY if:<br>[mdt_vu_no(2)] = '1'	Please state	text
1555	[bx_vu]	Diagnostic biopsy performed?	yesno<br>1 Yes<br>0 No

1556	[bx_no_reas_vu]<br>Show the field ONLY if:<br>[bx_vu] = '0'	Reason diagnostic biopsy not performed (select all that apply)	checkbox<br>1 bx_no_reas_vu___1 Biopsy not technically feasible<br>2 bx_no_reas_vu___2 Patient refused<br>3 bx_no_reas_vu___3 Patient unable to perform<br>4 bx_no_reas_vu___4 Not available locally<br>5 bx_no_reas_vu___5 Other
1557	[ps_bx_no_reas_vu]<br>Show the field ONLY if:<br>[bx_no_reas_vu(5)] = '1'	Please state	text
1558	[mri_vu]	Magnetic resonance imaging (MRI) pelvis/abdominal-pelvis performed at diagnosis?	yesno<br>1 Yes<br>0 No
1559	[mri_find_vu]<br>Show the field ONLY if:<br>[mri_vu] = '1'	MRI pelvis/abdominal-pelvis findings at diagnosis (select all that apply)	checkbox<br>1 mri_find_vu___1 Vulval tumour < 2cm<br>2 mri_find_vu___2 Vulval tumour >2cm<br>3 mri_find_vu___3 Perineal invasion<br>4 mri_find_vu___4 Vaginal invasion (lower third)<br>5 mri_find_vu___5 Vaginal invasion (upper two thirds)<br>6 mri_find_vu___6 Anal invasion<br>7 mri_find_vu___7 Rectal invasion<br>8 mri_find_vu___8 Sigmoid invasion<br>9 mri_find_vu___9 Urethral invasion (lower third)<br>10 mri_find_vu___10 Urethral invasion (upper two thirds)<br>11 mri_find_vu___11 Bladder invasion<br>12 mri_find_vu___12 x1 hydroureter<br>13 mri_find_vu___13 x2 hydroureter<br>14 mri_find_vu___14 Ureteric invasion<br>15 mri_find_vu___15 x1 hydronephrosis<br>16 mri_find_vu___16 x2 hydronephrosis<br>17 mri_find_vu___17 Pelvic bone invasion<br>18 mri_find_vu___18 Inguinofemoral lymphadenopathy<br>19 mri_find_vu___19 Pelvic lymphadenopathy<br>20 mri_find_vu___20 Para-aortic lymphadenopathy<br>21 mri_find_vu___21 Levator ani muscle invasion<br>22 mri_find_vu___22 Other
1560	[ps_mri_find_vu]<br>Show the field ONLY if:<br>[mri_find_vu(22)] = '1'	Please state	text
1561	[mri_no_vu]<br>Show the field ONLY if:<br>[mri_vu] = '0'	Reason MRI pelvis not performed at diagnosis (select all that apply)	checkbox<br>1 mri_no_vu___1 No access to MRI machine<br>2 mri_no_vu___2 Patient unable to afford<br>3 mri_no_vu___3 Patient refused<br>4 mri_no_vu___4 Claustrophobia<br>5 mri_no_vu___5 Other imaging performed<br>6 mri_no_vu___6 Extreme morbid obesity<br>7 mri_no_vu___7 Other
1562	[ps_mri_vu_no]<br>Show the field ONLY if:<br>[mri_no_vu(7)] = '1'	Please state	text

1563	[ct_vu]	Staging computerised tomography (CT) performed at diagnosis?	yesno<br>1 Yes<br>0 No
1564	[ct_find_vu]<br>Show the field ONLY if:<br>[ct_vu] = '1'	Staging CT findings at diagnosis (select all that apply)	checkbox<br>1 ct_find_vu___1 Vulval tumour < 2cm<br>2 ct_find_vu___2 Vulval tumour >2cm<br>3 ct_find_vu___3 Perineal invasion<br>4 ct_find_vu___4 Vaginal invasion (lower third)<br>5 ct_find_vu___5 Vaginal invasion (upper two thirds)<br>6 ct_find_vu___6 Anal invasion<br>7 ct_find_vu___7 Rectal invasion<br>8 ct_find_vu___8 Sigmoid invasion<br>9 ct_find_vu___9 Urethral invasion (lower third)<br>10 ct_find_vu___10 Urethral invasion (upper two thirds)<br>11 ct_find_vu___11 Bladder invasion<br>12 ct_find_vu___12 x1 hydroureter<br>13 ct_find_vu___13 x2 hydroureter<br>14 ct_find_vu___14 Ureteric invasion<br>15 ct_find_vu___15 x1 hydronephrosis<br>16 ct_find_vu___16 x2 hydronephrosis<br>17 ct_find_vu___17 Pelvic bone invasion<br>18 ct_find_vu___18 Inguinofemoral lymphadenopathy<br>19 ct_find_vu___19 Pelvic lymphadenopathy<br>20 ct_find_vu___20 Para-aortic lymphadenopathy<br>21 ct_find_vu___21 Levator ani muscle invasion<br>22 ct_find_vu___22 Lung nodules<br>23 ct_find_vu___23 Pleural effusion<br>24 ct_find_vu___24 Other
1565	[ps_ct_find_vu]<br>Show the field ONLY if:<br>[ct_find_vu(24)] = '1'	Please state	text
1566	[ct_vu_reas_no]<br>Show the field ONLY if:<br>[ct_vu] = '0'	Reason staging CT not performed at diagnosis	checkbox<br>1 ct_vu_reas_no___1 No access to CT machine<br>2 ct_vu_reas_no___2 Patient unable to afford<br>3 ct_vu_reas_no___3 Patient refused<br>4 ct_vu_reas_no___4 Other imaging performed<br>5 ct_vu_reas_no___5 Allergic reaction to contrast<br>6 ct_vu_reas_no___6 Chronic kidney disease<br>7 ct_vu_reas_no___7 Other
1567	[ps_ct_vu_reas_no]<br>Show the field ONLY if:<br>[ct_vu_reas_no(7)] = '1'	Please state	text
1568	[petct_vu]	PET-CT performed at diagnosis?	yesno<br>1 Yes<br>0 No

1569	[petct_find_vu]<br>Show the field ONLY if:<br>[petct_vu] = '1'	PET-CT findings at diagnosis (select all that apply)	checkbox<br>1 petct_find_vu___1 FDG-avid uptake in vulva<br>2 petct_find_vu___2 FDG-avid uptake in vagina<br>3 petct_find_vu___3 FDG-avid uptake in pelvic bones<br>4 petct_find_vu___4 FDG-avid uptake in inguinofemoral lymph nodes<br>5 petct_find_vu___5 FDG-avid uptake pelvic lymph nodes<br>6 petct_find_vu___6 FDG-avid uptake in para-aortic nodes<br>7 petct_find_vu___7 FDG-avid urethra<br>8 petct_find_vu___8 FDG-avid uptake in x1 ureter<br>9 petct_find_vu___9 FDG-avid uptake in x2 ureters<br>10 petct_find_vu___10 FDG-avid uptake in x1 kidney<br>11 petct_find_vu___11 FDG-avid uptake in x2 kidneys<br>12 petct_find_vu___12 FDG-avid uptake in bladder<br>13 petct_find_vu___13 FDG-avid uptake in para rectal nodules<br>14 petct_find_vu___14 FDG-avid uptake in sigmoid nodules<br>15 petct_find_vu___15 FDG-avid uptake in rectum<br>16 petct_find_vu___16 FDG-avid uptake in sigmoid<br>17 petct_find_vu___17 FDG-avid anus<br>18 petct_find_vu___18 FDG-avid uptake in lung nodules<br>19 petct_find_vu___19 FDG-avid uptake levator ani<br>20 petct_find_vu___20 Other
1570	[ps_petct_find_vu]<br>Show the field ONLY if:<br>[petct_find_vu(20)] = '1'	Please state	text
1571	[petct_no_reas_vu]<br>Show the field ONLY if:<br>[petct_vu] = '0'	Reason PET-CT not performed at diagnosis (select all that apply)	checkbox<br>1 petct_no_reas_vu___1 No access to PET machine<br>2 petct_no_reas_vu___2 Patient unable to afford<br>3 petct_no_reas_vu___3 Patient refused<br>4 petct_no_reas_vu___4 Uncontrolled diabetes mellitus<br>5 petct_no_reas_vu___5 Other imaging performed<br>6 petct_no_reas_vu___6 Not clinical protocol<br>7 petct_no_reas_vu___7 Other
1572	[ps_petct_no_reas_vu]<br>Show the field ONLY if:<br>[petct_no_reas_vu(7)] = '1'	Please state	text
1573	[uss_vu]	Ultrasound scan groins	yesno<br>1 Yes<br>0 No

1574	[uss_no_vu]<br>Show the field ONLY if:<br>[uss_vu] = '0'	Reason not performed (select all that apply)	checkbox<br>1 uss_no_vu___1 Not clinically indicated<br>2 uss_no_vu___2 Other imaging performed<br>3 uss_no_vu___3 Not available locally<br>4 uss_no_vu___4 Patient refused<br>5 uss_no_vu___5 Patient unable to afford<br>6 uss_no_vu___6 Other
1575	[ps_uss_no_vu]<br>Show the field ONLY if:<br>[uss_no_vu(6)] = '1'	Please state	text
1576	[ln_siz_uss_vu]<br>Show the field ONLY if:<br>[uss_vu] = '1'	Lymph nodes size	radio<br>1 < 1.5cm<br>2 ≥1.5cm
1577	[fna_uss_vu]<br>Show the field ONLY if:<br>[ln_siz_uss_vu] = '2'	Fine needle aspiration	yesno<br>1 Yes<br>0 No
1578	[fna_res_vu]<br>Show the field ONLY if:<br>[fna_uss_vu] = '1'	Fine needle aspiration results	dropdown<br>1 Positive<br>2 Negative
1579	[fna_no_vu]<br>Show the field ONLY if:<br>[fna_uss_vu] = '0'	Reason not performed (select all that apply)	checkbox<br>1 fna_no_vu___1 Not technically feasible<br>2 fna_no_vu___2 Other imaging performed<br>3 fna_no_vu___3 Not available locally<br>4 fna_no_vu___4 Patient refused<br>5 fna_no_vu___5 Patient unable to afford<br>6 fna_no_vu___6 Other
1580	[ps_fna_no_vu]<br>Show the field ONLY if:<br>[fna_no_vu(6)] = '1'	Please state	text

1581	[figo_vu]	Clinical FIGO Stage (i.e. preoperative staging taking into account imaging, clinical examination)	dropdown<br>1 — 1A Lesions ≤2 cm in size, confined to the vulva or perineum and with stromal invasion ≤1.0 mm, no nodal metastasis<br>2 — 1B Lesions >2 cm in size or with stromal invasion >1.0 mm, confined to the vulva or perineum, with negative nodes<br>3 — II Tumor of any size with extension to adjacent perineal structures (lower third of urethra, lower third of vagina, anus) with negative nodes<br>4 — IIIA Tumor of any size with or without extension to adjacent perineal structures (lower third of urethra, lower third of vagina, anus) with positive inguinofemoral nodes (with 1 lymph node metastasis (≥5 mm), or with 1-2 lymph node metastasis(es) (< 5 mm))<br>5 — IIIB Tumor of any size with or without extension to adjacent perineal structures (lower third of urethra, lower third of vagina, anus) with positive inguinofemoral nodes (with 2 or more lymph node metastases (≥5 mm), or with 3 or more lymph node metastases (< 5 mm))<br>6 — IIIC Tumor of any size with or without extension to adjacent perineal structures (lower third of urethra, lower third of vagina, anus) with positive inguinofemoral nodes with extracapsular spread<br>7 — IVA Tumor invades any of the following: upper urethral and/or vaginal mucosa, bladder mucosa, rectal mucosa, or fixed to pelvic bone, or fixed or ulcerated inguinofemoral lymph nodes<br>10 — IVB Any distant metastasis including pelvic lymph nodes
1582	[nact_vu]	Neoadjuvant (before surgery) chemotherapy	yesno<br>1 — Yes<br>0 — No
1583	[chem1_vu]<br>Show the field ONLY if:<br>[nact_vu] = '1'	Chemotherapy regimen FIRST line (Select all that apply)	checkbox<br>1 — chem1_vu___1 — Platinum (for example cisplatin, carboplatin)<br>2 — chem1_vu___2 — Fluorouracil<br>3 — chem1_vu___3 — Taxane (for example paclitaxel)<br>4 — chem1_vu___4 — Vinorelbine<br>5 — chem1_vu___5 — Erlotinib<br>6 — chem1_vu___6 — Clinical trial regimens<br>7 — chem1_vu___7 — Other
1584	[ps_chem1_vu]<br>Show the field ONLY if:<br>[chem1_vu(7)] = '1'	Please state	text
1585	[chemc_vu]<br>Show the field ONLY if:<br>[nact_vu] = '1'	Total number of chemotherapy cycles received	text (number)
1586	[nact_no_reas_vu]<br>Show the field ONLY if:<br>[nact_vu] = '0'	Reason neoadjuvant chemotherapy not administered (select all that apply)	checkbox<br>1 — nact_no_reas_vu___1 — Poor performance status<br>2 — nact_no_reas_vu___2 — No access<br>3 — nact_no_reas_vu___3 — Patient unable to afford<br>4 — nact_no_reas_vu___4 — Patient refused<br>5 — nact_no_reas_vu___5 — Not clinically indicated<br>6 — nact_no_reas_vu___6 — Other
1587	[ps_nact_no_reas_vu]<br>Show the field ONLY if:<br>[nact_no_reas_vu(6)] = '1'	Please state	text

1588	[cov19_vu]	COVID-19 status	dropdown<br>1 Positive<br>2 Negative<br>3 Not tested
1589	[cov_notst_vu]<br>Show the field ONLY if:<br>[cov19_vu] = '3'	If not tested select reason (select all that apply)	checkbox<br>1 cov_notst_vu___1 Not clinically indicated (asymptomatic)<br>2 cov_notst_vu___2 Not clinical protocol to routinely test all patients prior to surgery<br>3 cov_notst_vu___3 Patient refused<br>4 cov_notst_vu___4 Patient unable to afford test<br>5 cov_notst_vu___5 No local resources for testing<br>6 cov_notst_vu___6 Other
1590	[ps_cov_notst_vu]<br>Show the field ONLY if:<br>[cov_notst_vu(6)] = '1'	Please state	text
1591	[bp_vu]	Bowel preparation pre-operatively	yesno<br>1 Yes<br>0 No
1592	[peri_op_vu]	Peri-operative management plan in place? (Non- exhaustive list: pre-operative haemoglobin optimisation and iron deficit correction; correction of denutrition and immunonutrition; fluid management; pain management; prevention of post-operative nausea and vomiting).	yesno<br>1 Yes<br>0 No
1593	[hb_vu]	Pre-operative Haemoglobin (g/L)	text (number)
1594	[wcc_vu]	Pre-operative White Cell count (10^9/L)	text (number)
1595	[plts_vu]	Pre-operative Platelets (10^9/L)	text (number)
1596	[pt_vu]	Pre-operative PT (Secs)	text (number)
1597	[inr_vu]	Pre-operative INR (ratio)	text (number)
1598	[aptt_vu]	Pre-operative APTT (Secs)	text (number)
1599	[fib_vu]	Pre-operative Fibrinogen (g/L)	text (number)
1600	[alb_vu]	Pre-operative Albumin (g/L)	text (number)
1601	[na_vu]	Pre-operative Sodium (mmol/L)	text (number)
1602	[k_vu]	Pre-operative Potassium (mmol/L)	text (number)
1603	[ur_vu]	Pre-operative Urea (mmol/L)	text (number)
1604	[creat_vu]	Pre-operative Creatinine (μmol/L)	text (number)
1605	[egfr_vu]	Pre-operative eGFR - estimated glomerular filtration rate (mL/min)	text
1606	[bili_vu]	Pre-operative Bilirubin total (μmol/L)	text (number)
1607	[aat_vu]	Pre-operative Alanine Aminotransaminase (IU/L)	text (number)
1608	[alp_vu]	Pre-operative Alkaline Phosphatase (IU/L)	text (number)
1609	[ast_vu]	Pre-operative Aspartate Aminotransaminase (IU/L)	text (number)
1610	[crp_vu]	Pre-operative CRP - C-reactive protein (mg/L)	text (number)
1611	[surgdat_vu]	Section Header: *SURGERY*<br>Date of surgery	text (date_dmy)
1612	[anaes_vu]	Anaesthesia (select all that apply)	checkbox<br>1 anaes_vu___1 General<br>2 anaes_vu___2 Spinal<br>3 anaes_vu___3 Epidural
1613	[surg_elecem_vu]	Was the surgery an elective or emergency case?	radio<br>1 Elective<br>2 Emergency

1614	[sa_vu]	Surgical aim	dropdown<br>1 Curative<br>2 Curative attempted but then abandoned (i.e. open/close laparotomy)<br>3 Palliative
1615	[ls_vu]	Lead surgeon performing surgery	dropdown<br>1 Certified consultant/specialist gynaecological oncologist<br>2 Fellow/trainee gynaecological oncologist<br>3 Trained surgeon dedicated to the management of gynaecologic cancer or having completed an ESGO accredited fellowship.<br>4 General surgeon<br>5 Surgical oncologist<br>6 Other
1616	[ps_ls_vu]<br>Show the field ONLY if:<br>[ls_vu] = '6'	Please state	text
1617	[who_vu]	WHO surgical safety checklist used	yesno<br>1 Yes<br>0 No
1618	[sm_vu]	Surgical procedure (select all that apply)	checkbox<br>1 sm_vu___1 Wide local excision<br>2 sm_vu___2 Sentinel lymph nodes mapping<br>3 sm_vu___3 Groin lymph node dissection<br>4 sm_vu___4 Vulvectomy<br>5 sm_vu___5 Pelvic lymph node dissection<br>6 sm_vu___6 Infralevator extenteration
1619	[sit_map_sln_vu]<br>Show the field ONLY if:<br>[sm_vu(2)] = '1'	Site of mapping (select all that apply)	checkbox<br>1 sit_map_sln_vu___1 Right inguinofemoral lymph nodes<br>2 sit_map_sln_vu___2 Left inguinofemoral lymph nodes
1620	[sln_res_vu]<br>Show the field ONLY if:<br>[sit_map_sln_vu(1)] = '1'	Results	dropdown<br>1 Positive<br>2 Negative
1621	[sln_res_vu_2]<br>Show the field ONLY if:<br>[sit_map_sln_vu(2)] = '1'	Results	dropdown<br>1 Positive<br>2 Negative
1622	[trcr_sln_vu]<br>Show the field ONLY if:<br>[sm_vu(2)] = '1'	Tracer	dropdown<br>1 Indocyanine green<br>2 Methylene blue<br>3 Technetium<br>4 Other
1623	[ps_sln_vu]<br>Show the field ONLY if:<br>[trcr_sln_vu] = '4'	Please state	text
1624	[cyto_vu]	Complete macroscopic cytoreduction at surgery	yesno<br>1 Yes<br>0 No

1625	[reas_cytored_no_vu]<br>Show the field ONLY if:<br>[cyto_vu] = '0'	Reason(s) disease not fully excised at cytoreduction (select all reasons that apply)	checkbox<br>1 reas_cytored_no_vu___1 Disease more extensive than that shown on imaging<br>2 reas_cytored_no_vu___2 Technically unable to resect<br>3 reas_cytored_no_vu___3 Patient became medically unstable intra-operatively and decision made to not proceed further with surgery<br>4 reas_cytored_no_vu___4 Other
1626	[ps_reas_cytored_no_vu]<br>Show the field ONLY if:<br>[reas_cytored_no_vu(4)] = '1'	Please state	text
1627	[siz_resid_vu]<br>Show the field ONLY if:<br>[cyto_vu] = '0'	Size of residual disease (millimetres)	dropdown<br>1 1-2.5<br>2 2.6-5<br>3 5.1-7.5<br>4 7.6-10<br>5 >10
1628	[loc_resid_vu]<br>Show the field ONLY if:<br>[cyto_vu] = '0'	Location of residual disease (Select all that apply)	checkbox<br>1 loc_resid_vu___1 Vulva<br>2 loc_resid_vu___2 Vagina (lower third)<br>3 loc_resid_vu___3 Vagina (upper two thirds)<br>4 loc_resid_vu___4 Pelvic bone<br>5 loc_resid_vu___5 Urethra (lower third)<br>6 loc_resid_vu___6 Urethra (upper two thirds)<br>7 loc_resid_vu___7 Bladder<br>8 loc_resid_vu___8 ureter(s)<br>9 loc_resid_vu___9 kidney(s)<br>10 loc_resid_vu___10 Anus<br>11 loc_resid_vu___11 Rectum<br>12 loc_resid_vu___12 Sigmoid<br>13 loc_resid_vu___13 Para-aortic-lymphadenopathy<br>14 loc_resid_vu___14 Inguinofemoral lyphadenopathy<br>15 loc_resid_vu___15 Pelvic lymphadenopathy<br>16 loc_resid_vu___16 Levator ani<br>17 loc_resid_vu___17 Other
1629	[ps_loc_resid_vu]<br>Show the field ONLY if:<br>[loc_resid_vu(17)] = '1'	Please state	text

1630	[surg_find_vu]	Surgical findings (select all that apply)	checkbox<br>1 surg_find_vu___1 Vulval tumour < 2cm<br>2 surg_find_vu___2 Vulval tumour >2cm<br>3 surg_find_vu___3 Vaginal invasion (lower third)<br>4 surg_find_vu___4 Vaginal invasion (upper two thirds)<br>5 surg_find_vu___5 Pelvic bone invasion<br>6 surg_find_vu___6 Urethral invasion (lower third)<br>7 surg_find_vu___7 Urethral invasion (upper two thirds)<br>8 surg_find_vu___8 Bladder invasion<br>9 surg_find_vu___9 x1 hydroureter<br>10 surg_find_vu___10 x2 hydroureter<br>11 surg_find_vu___11 Ureteric invasion<br>12 surg_find_vu___12 x1 hydronephrosis<br>13 surg_find_vu___13 x2 hydronephrosis<br>14 surg_find_vu___14 Anal invasion<br>15 surg_find_vu___15 Rectal invasion<br>16 surg_find_vu___16 Sigmoid invasion<br>17 surg_find_vu___17 Para-aortic-lymphadenopathy<br>18 surg_find_vu___18 Inguinofemoral lyphadenopathy<br>19 surg_find_vu___19 Pelvic lymphadenopathy<br>20 surg_find_vu___20 Levator ani invasion<br>21 surg_find_vu___21 Other<br>22 surg_find_vu___22 No visible disease
1631	[ps_surg_find_vu]<br>Show the field ONLY if:<br>[surg_find_vu(21)] = '1'	Please state	text
1632	[tis_vu]	Tissue removed (Select all that apply)	checkbox<br>1 tis_vu___1 Vulva<br>2 tis_vu___2 Cervix<br>3 tis_vu___3 Uterus<br>4 tis_vu___4 x1 fallopian tube<br>5 tis_vu___5 x2 fallopian tubes<br>6 tis_vu___6 x1 ovary<br>7 tis_vu___7 x2 ovaries<br>8 tis_vu___8 Inguinofemaral lymph nodes<br>9 tis_vu___9 Pelvic lymph nodes<br>10 tis_vu___10 Para-aortic lymph nodes<br>11 tis_vu___11 Anus<br>12 tis_vu___12 Rectum<br>13 tis_vu___13 Sigmoid<br>14 tis_vu___14 Kidney<br>15 tis_vu___15 Ureter<br>16 tis_vu___16 Bladder<br>17 tis_vu___17 Levator ani<br>18 tis_vu___18 Other
1633	[paln_dis_vu]<br>Show the field ONLY if:<br>[tis_vu(10)] = '1'	Select all that apply	checkbox<br>1 paln_dis_vu___1 Infra-renal<br>2 paln_dis_vu___2 Supra-renal

1634	[ps_tis_vu]<br>Show the field ONLY if:<br>[tis_vu(18)] = '1'	Please state	text
1635	[si_stom_vu]	Small bowel stoma	yesno<br>1 Yes<br>0 No
1636	[ss_vu]<br>Show the field ONLY if:<br>[si_stom_vu] = '1'	Stoma site	dropdown<br>1 Duodenum<br>2 Jejunum<br>3 Ileum
1637	[pb_si_stom_vu]<br>Show the field ONLY if:<br>[si_stom_vu] = '1'	Performed by (select all that apply)	checkbox<br>1 pb_si_stom_vu___1 Gynaecological oncologist<br>2 pb_si_stom_vu___2 Colorectal surgeon<br>3 pb_si_stom_vu___3 Urologist<br>4 pb_si_stom_vu___4 General surgeon<br>5 pb_si_stom_vu___5 Plastic surgeon<br>6 pb_si_stom_vu___6 Other
1638	[ps_pb_si_stom_vu]<br>Show the field ONLY if:<br>[pb_si_stom_vu(6)] = '1'	Please state	text
1639	[si_anast_vu]	Small bowel anastamosis	yesno<br>1 Yes<br>0 No
1640	[pb_si_anast_vu]<br>Show the field ONLY if:<br>[si_anast_vu] = '1'	Performed by (select all that apply)	checkbox<br>1 pb_si_anast_vu___1 Gynaecological oncologist<br>2 pb_si_anast_vu___2 Colorectal surgeon<br>3 pb_si_anast_vu___3 Urologist<br>4 pb_si_anast_vu___4 General surgeon<br>5 pb_si_anast_vu___5 Plastic surgeon<br>6 pb_si_anast_vu___6 Other
1641	[ps_pb_si_anast_vu]<br>Show the field ONLY if:<br>[pb_si_anast_vu(6)] = '1'	Please state	text
1642	[li_stom_vu]	Large bowel stoma	yesno<br>1 Yes<br>0 No
1643	[pb_li_stom_vu]<br>Show the field ONLY if:<br>[li_stom_vu] = '1'	Performed by (select all that apply)	checkbox<br>1 pb_li_stom_vu___1 Gynaecological oncologist<br>2 pb_li_stom_vu___2 Colorectal surgeon<br>3 pb_li_stom_vu___3 Urologist<br>4 pb_li_stom_vu___4 General surgeon<br>5 pb_li_stom_vu___5 Plastic surgeon<br>6 pb_li_stom_vu___6 Other
1644	[ps_pb_li_stom_vu]<br>Show the field ONLY if:<br>[pb_li_stom_vu(6)] = '1'	Please state	text
1645	[li_anas_vu]	Large bowel anastamosis	yesno<br>1 Yes<br>0 No

1646	[pb_li_anast_vu]<br>Show the field ONLY if:<br>[li_anas_vu] = '1'	Performed by (select all that apply)	checkbox<br>1 pb_li_anast_vu___1 Gynaecological oncologist<br>2 pb_li_anast_vu___2 Colorectal surgeon<br>3 pb_li_anast_vu___3 Urologist<br>4 pb_li_anast_vu___4 General surgeon<br>5 pb_li_anast_vu___5 Plastic surgeon<br>6 pb_li_anast_vu___6 Other
1647	[ps_pb_li_anast_vu]<br>Show the field ONLY if:<br>[pb_li_anast_vu(6)] = '1'	Please state	text
1648	[urost_vu]	Urostomy/ileal conduit	yesno<br>1 Yes<br>0 No
1649	[pb_urost_vu]<br>Show the field ONLY if:<br>[urost_vu] = '1'	Performed by (select all that apply)	checkbox<br>1 pb_urost_vu___1 Gynaecological oncologist<br>2 pb_urost_vu___2 Colorectal surgeon<br>3 pb_urost_vu___3 Urologist<br>4 pb_urost_vu___4 General surgeon<br>5 pb_urost_vu___5 Plastic surgeon<br>6 pb_urost_vu___6 Other
1650	[ps_pb_urost_vu]<br>Show the field ONLY if:<br>[pb_urost_vu(6)] = '1'	Please state	text
1651	[tuu_vu]	Trans uretero-ureteric anastamosis	yesno<br>1 Yes<br>0 No
1652	[pb_tuu_vu]<br>Show the field ONLY if:<br>[tuu_vu] = '1'	Performed by (select all that apply)	checkbox<br>1 pb_tuu_vu___1 Gynaecological oncologist<br>2 pb_tuu_vu___2 Colorectal surgeon<br>3 pb_tuu_vu___3 Urologist<br>4 pb_tuu_vu___4 General surgeon<br>5 pb_tuu_vu___5 Plastic surgeon<br>6 pb_tuu_vu___6 Other
1653	[ps_pb_tuu_vu]<br>Show the field ONLY if:<br>[pb_tuu_vu(6)] = '1'	Please state	text
1654	[ui_vu]	Ureteric reimplantation	yesno<br>1 Yes<br>0 No
1655	[pb_ui_vu]<br>Show the field ONLY if:<br>[ui_vu] = '1'	Performed by (select all that apply)	checkbox<br>1 pb_ui_vu___1 Gynaecological oncologist<br>2 pb_ui_vu___2 Colorectal surgeon<br>3 pb_ui_vu___3 Urologist<br>4 pb_ui_vu___4 General surgeon<br>5 pb_ui_vu___5 Plastic surgeon<br>6 pb_ui_vu___6 Other
1656	[ps_pb_ui_vu]<br>Show the field ONLY if:<br>[pb_ui_vu(6)] = '1'	Please state	text
1657	[neph_vu]	Nephrostomy	yesno<br>1 Yes<br>0 No

1658	[pb_neph_vu]<br>Show the field ONLY if:<br>[neph_vu] = '1'	Performed by (select all that apply)	checkbox<br>1 pb_neph_vu___1 Gynaecological oncologist<br>2 pb_neph_vu___2 Colorectal surgeon<br>3 pb_neph_vu___3 Urologist<br>4 pb_neph_vu___4 General surgeon<br>5 pb_neph_vu___5 Plastic surgeon<br>6 pb_neph_vu___6 Other
1659	[ps_pb_neph_vu]<br>Show the field ONLY if:<br>[pb_neph_vu(6)] = '1'	Please state	text
1660	[vr_vu]	Vulval reconstruction	yesno<br>1 Yes<br>0 No
1661	[vrf_vu]<br>Show the field ONLY if:<br>[vr_vu] = '1'	Type of vulval reconstruction	radio<br>1 Rotational flap<br>2 Non rotational flap<br>3 Graft
1662	[adsurg_vu]<br>Show the field ONLY if:<br>[vr_vu] = '1'	Performed by (select all that apply)	checkbox<br>1 adsurg_vu___1 Gynaecological oncologist<br>2 adsurg_vu___2 Colorectal surgeon<br>3 adsurg_vu___3 Urologist<br>4 adsurg_vu___4 General surgeon<br>5 adsurg_vu___5 Plastic surgeon<br>6 adsurg_vu___6 Other
1663	[ps_adsurg_vu]<br>Show the field ONLY if:<br>[adsurg_vu(6)] = '1'	Please state	text
1664	[abx_vu]	Intraoperative antibiotics	yesno<br>1 Yes<br>0 No
1665	[tim_vu]	Length of surgery	text (time)
1666	[itu_vu]	Section Header: *POST-OPERATIVE*<br>Post-operative recovery in intensive care unit	yesno<br>1 Yes<br>0 No
1667	[itu_dys_vu]<br>Show the field ONLY if:<br>[itu_vu] = '1'	Number of days in intensive care unit	text (number)
1668	[hdu_vu]	Post-operative recovery in high dependency unit	yesno<br>1 Yes<br>0 No
1669	[hdu_dys_vu]<br>Show the field ONLY if:<br>[hdu_vu] = '1'	Number of days in high dependency unit	text (number)
1670	[er_vu]	Enhanced recovery?	yesno<br>1 Yes<br>0 No
1671	[po_abx_vu]	Prophylactic post operative antibiotics	yesno<br>1 Yes<br>0 No
1672	[drn_vu]	Post operative surgical drain	yesno<br>1 Yes<br>0 No

1673	[drn_dys_vu]<br>Show the field ONLY if:<br>[drn_vu] = '1'	Number of days in situ	text (number)
1674	[uc_vu]	Post operative urinary catheter	yesno<br>1 Yes<br>0 No
1675	[cath_dys_vu]<br>Show the field ONLY if:<br>[uc_vu] = '1'	Number of days in situ	text (number)
1676	[phb_vu]	Post-operative Haemoglobin (g/L) - please state first post-operative result	text (number)
1677	[pwbc_vu]	Post-operative White Cell Count (10^9/L) - please state first post-operative result	text (number)
1678	[pplat_vu]	Post-operative Platelets (10^9/L) - please state first post-operative result	text (number)
1679	[ppt_vu]	Post-operative PT (Secs) - please state first post-operative result	text (number)
1680	[pinr_vu]	Post-operative INR (Ratio) - please state first post-operative result	text (number)
1681	[paptt_vu]	Post-operative APTT (Secs) - please state first post-operative result	text (number)
1682	[pfib_vu]	Post-operative Fibrinogen (g/L) - please state first post-operative result	text (number)
1683	[palb_vu]	Post-operative Albumin (g/L) - please state first post-operative result	text (number)
1684	[pna_vu]	Post-operative Sodium (mmol/L) - please state first post-operative result	text (number)
1685	[pk_vu]	Post-operative Potassium (mmol/L) - please state first post-operative result	text (number)
1686	[purea_vu]	Post-operative Urea (mmol/L) - please state first post-operative result	text (number)
1687	[pcreat_vu]	Post-operative Creatinine (μmol/L) - please state first post-operative result	text
1688	[pegfr_vu]	Post-operative eGFR - Estimated Glomerular Filtration Rate (mL/min) - please state first post-operative result	text
1689	[pbili_vu]	Post-operative Bilirubin total (μmol/L) - please state first post-operative result	text (number)
1690	[paat_vu]	Post-operative Alanine Aminotransaminase (IU/L) - please state first post-operative result	text (number)
1691	[palp_vu]	Post-operative Alkaline Phosphatase (IU/L) - please state first post-operative result	text (number)
1692	[past_vu]	Post-operative Aspartate Aminotransaminase (IU/L) - please state first post-operative result	text (number)
1693	[pcrp_vu]	Post-operative CRP - C-reactive protein (mg/L) - please state first post-operative result	text (number)
1694	[hist_vu]	Histologic type	dropdown<br>1 Squamous cell<br>2 Malignant melanoma<br>3 Adenocarcinoma<br>4 Basal cell carcinoma<br>5 Sarcoma<br>6 Pagets with invasion
1695	[scc_uni_multi_vu]<br>Show the field ONLY if:<br>[hist_vu] = '1'	Unifocal or multifocal	dropdown<br>1 Unifocal<br>2 Multifocal
1696	[lichs_vu]	Vulval carcinoma on a background of lichen sclerosus	yesno<br>1 Yes<br>0 No

1697	[ts_ls_vu]<br>Show the field ONLY if:<br>[lichs_vu] = '1'	Maintenance dose of topical steroids (e.g. clobetasol propionate)	yesno<br>1 Yes<br>0 No
1698	[r_vu]	Histological confirmed resection margins	dropdown<br>1 < 1 mm / tumour on margin<br>2 1-5 mm (NO tumour on margin)<br>3 >5 mm Margins confirmed clear<br>4 Unknown, not available in this hospital
1699	[dis_marg_vu]	Disease at margins	dropdown<br>1 Vulval intraepithelial neoplasia I<br>2 Vulval intraepithelial neoplasia II<br>3 Vulval intraepithelial neoplasia III<br>4 Invasion<br>5 Disease free (i.e. no VIN/invasion)
1700	[figo_vu_2]	Final surgical FIGO stage (i.e. postoperative staging taking into account surgical and pathology findings)	dropdown<br>1 1A Lesions ≤2 cm in size, confined to the vulva or perineum and with stromal invasion ≤1.0 mm, no nodal metastasis<br>2 1B Lesions >2 cm in size or with stromal invasion >1.0 mm, confined to the vulva or perineum, with negative nodes<br>3 II Tumor of any size with extension to adjacent perineal structures (lower third of urethra, lower third of vagina, anus) with negative nodes<br>4 IIIA Tumor of any size with or without extension to adjacent perineal structures (lower third of urethra, lower third of vagina, anus) with positive inguinofemoral nodes (with 1 lymph node metastasis (≥5 mm), or with 1-2 lymph node metastasis(es) (< 5 mm))<br>5 IIIB Tumor of any size with or without extension to adjacent perineal structures (lower third of urethra, lower third of vagina, anus) with positive inguinofemoral nodes (with 2 or more lymph node metastases (≥5 mm), or with 3 or more lymph node metastases (< 5 mm))<br>6 IIIC Tumor of any size with or without extension to adjacent perineal structures (lower third of urethra, lower third of vagina, anus) with positive inguinofemoral nodes with extracapsular spread<br>7 IVA Tumor invades any of the following: upper urethral and/or vaginal mucosa, bladder mucosa, rectal mucosa, or fixed to pelvic bone, or fixed or ulcerated inguinofemoral lymph nodes<br>10 IVB Any distant metastasis including pelvic lymph nodes
1701	[p16_vu]	Section Header: *Immunohistochemistry*<br>p16	radio (Matrix)<br>1 Positive<br>2 Negative<br>3 Not tested
1702	[ck7_vu]	CK7	radio (Matrix)<br>1 Positive<br>2 Negative<br>3 Not tested
1703	[p53_vu]	p53	radio (Matrix)<br>1 Positive<br>2 Negative<br>3 Not tested

1704	[ck20_vu]	CK20	radio (Matrix)<br>1 Positive<br>2 Negative<br>3 Not tested
1705	[cea_vu]	CEA	radio (Matrix)<br>1 Positive<br>2 Negative<br>3 Not tested
1706	[ck8_18_vu]	CK8/18	radio (Matrix)<br>1 Positive<br>2 Negative<br>3 Not tested
1707	[s100_vu]	S100	radio (Matrix)<br>1 Positive<br>2 Negative<br>3 Not tested
1708	[melan_a_vu]	Melan A	radio (Matrix)<br>1 Positive<br>2 Negative<br>3 Not tested
1709	[cam5_2_vu]	CAM5.2	radio (Matrix)<br>1 Positive<br>2 Negative<br>3 Not tested
1710	[muc2_vu]	MUC-2	radio (Matrix)<br>1 Positive<br>2 Negative<br>3 Not tested
1711	[urop_vu]	uroplakin III	radio (Matrix)<br>1 Positive<br>2 Negative<br>3 Not tested
1712	[ac_vu]	Adjuvant (after surgery) chemotherapy	yesno<br>1 Yes<br>0 No
1713	[hmb45_vu]	HMB 45	radio (Matrix)<br>1 Positive<br>2 Negative<br>3 Not tested
1714	[adjchem1_reg_vu]<br>Show the field ONLY if:<br>[ac_vu] = '1'	Chemotherapy regimen FIRST line (Select all that apply)	checkbox<br>1 adjchem1_reg_vu___1 Platinum (for example cisplatin, carboplatin)<br>2 adjchem1_reg_vu___2 Fluorouracil<br>3 adjchem1_reg_vu___3 Taxane (for example paclitaxel)<br>4 adjchem1_reg_vu___4 Vinorelbine<br>5 adjchem1_reg_vu___5 Erlotinib<br>6 adjchem1_reg_vu___6 Clinical trial regimens<br>7 adjchem1_reg_vu___7 Other
1715	[ps_adjcem1_reg_vu]<br>Show the field ONLY if:<br>[adjchem1_reg_vu(7)] = '1'	Please state	text

<table>
<tr><td>1716</td><td>[adjchem_cycl_numb]
Show the field ONLY if:
[ac_vu] = '1'</td><td>Number of cycles planned</td><td>text (number)</td></tr>
<tr><td>1717</td><td>[adjchem_no_reas_vu]
Show the field ONLY if:
[ac_vu] = '0'</td><td>Reason adjuvant chemotherapy not administered (select all that apply)</td><td>checkbox
1 | adjchem_no_reas_vu___1 | Poor performance status
2 | adjchem_no_reas_vu___2 | No access locally
3 | adjchem_no_reas_vu___3 | Patient unable to afford
4 | adjchem_no_reas_vu___4 | Patient refused
5 | adjchem_no_reas_vu___5 | Not clinically indicated
6 | adjchem_no_reas_vu___6 | Other</td></tr>
<tr><td>1718</td><td>[ps_adjchemo_no_reas_vu]
Show the field ONLY if:
[adjchem_no_reas_vu(6)] = '1'</td><td>Please state</td><td>text</td></tr>
<tr><td>1719</td><td>[rt_vu]</td><td>External beam radiotherapy administered</td><td>yesno
1 | Yes
0 | No</td></tr>
<tr><td>1720</td><td>[rt_site_vu]
Show the field ONLY if:
[rt_vu] = '1'</td><td>External beam radiotherapy administered to</td><td>dropdown
1 | Vulva
2 | Groins
3 | Vulva and groins</td></tr>
<tr><td>1721</td><td>[rt_dos_vu]
Show the field ONLY if:
[rt_vu] = '1'</td><td>Dose administered (Gray - Gy)</td><td>text (number)</td></tr>
<tr><td>1722</td><td>[rt_no_vu]
Show the field ONLY if:
[rt_vu] = '0'</td><td>Reason radiotherapy not administered (select all that apply)</td><td>checkbox
1 | rt_no_vu___1 | Poor performance status
2 | rt_no_vu___2 | Not available locally
3 | rt_no_vu___3 | Patient unable to afford
4 | rt_no_vu___4 | Patient refused
5 | rt_no_vu___5 | Not clinically indicated
6 | rt_no_vu___6 | Other</td></tr>
<tr><td>1723</td><td>[ps_rt_no_vu]
Show the field ONLY if:
[rt_no_vu(6)] = '1'</td><td>Please state</td><td>text</td></tr>
<tr><td>1724</td><td>[brachy_vu]</td><td>Brachytherapy administered</td><td>yesno
1 | Yes
0 | No</td></tr>
<tr><td>1725</td><td>[rt_dos_vu_2]
Show the field ONLY if:
[brachy_vu] = '1'</td><td>Dose administered (Gray - Gy)</td><td>text (number)</td></tr>
<tr><td>1726</td><td>[brachy_no_reas_vu]
Show the field ONLY if:
[brachy_vu] = '0'</td><td>Reason brachytherapy not administered (select all that apply)</td><td>checkbox
1 | brachy_no_reas_vu___1 | Poor performance status
2 | brachy_no_reas_vu___2 | Not available locally
3 | brachy_no_reas_vu___3 | Patient unable to afford
4 | brachy_no_reas_vu___4 | Patient refused
5 | brachy_no_reas_vu___5 | Not clinically indicated
6 | brachy_no_reas_vu___6 | Other</td></tr>
<tr><td>1727</td><td>[ps_brachy_no_reas_vu]
Show the field ONLY if:
[brachy_no_reas_vu(6)] = '1'</td><td>Please state</td><td>text</td></tr>
</table>

1728	[vulval_cancer_initial_presentation_complete]	Section Header: *Form Status*<br>Complete?	dropdown<br>0 Incomplete<br>1 Unverified<br>2 Complete
Instrument: **Vulval cancer (recurrence)** (vulval_cancer_recurrence)			
1729	[dat_dx_vu]	Section Header: *PREVIOUS TREATMENT RECEIVED AT PRIMARY DIAGNOSIS*<br>Date of primary/initial diagnosis	text (date_dmy)
1730	[prim_tx_vu]	Previous treatment following primary/initial diagnosis	checkbox<br>1 prim_tx_vu___1 Cytoreductive surgery<br>2 prim_tx_vu___2 Adjuvant chemotherapy<br>3 prim_tx_vu___3 Neoadjuvant chemotherapy<br>4 prim_tx_vu___4 External beam radiotherapy<br>5 prim_tx_vu___5 Brachytherapy<br>6 prim_tx_vu___6 Immunotherapy
1731	[dat_surg1_vu]<br>Show the field ONLY if:<br>[prim_tx_vu(1)] = '1'	Date of primary cytoreductive surgery	text (date_dmy)
1732	[dat_chem1_vu]<br>Show the field ONLY if:<br>[prim_tx_vu(2)] = '1'	Date of adjuvant chemotherapy following initial diagnosis	text (date_dmy)
1733	[dat_nact1_vu]<br>Show the field ONLY if:<br>[prim_tx_vu(3)] = '1'	Date of neoadjuvant chemotherapy following initial diagnosis	text (date_dmy)
1734	[dat_rt1_vu]<br>Show the field ONLY if:<br>[prim_tx_vu(4)] = '1'	Date of external beam radiotherapy following initial diagnosis	text (date_dmy)
1735	[dat_imm_vu_2]<br>Show the field ONLY if:<br>[prim_tx_vu(6)] = '1'	Date of immunotherapy following initial diagnosis	text (date_dmy)
1736	[dat_brachy1_vu]<br>Show the field ONLY if:<br>[prim_tx_vu(5)] = '1'	Date of brachytherapy following initial diagnosis	text (date_dmy)
1737	[dat_rec_vu]	Date of recurrence diagnosis	text (date_dmy)
1738	[age_vu_v2]	Section Header: *BASELINE DEMOGRAPHICS*<br>Age of patient	text (number)
1739	[eth_vu_v2]	Ethnicity	dropdown<br>1 White (British, Australian, North American, European, other white)<br>2 Asian (South East Asian, Far East Asian, other Asian)<br>3 Black (African, Caribbean, other Black)<br>4 Middle Eastern (Arab, North African, Turkish)<br>5 Mixed (multiple ethnic background)<br>6 Other
1740	[ps_eth_vu_v2]<br>Show the field ONLY if:<br>[eth_vu_v2] = '6'	Please state	text
1741	[bmi_vu_v2]	Body mass index (kg/m2)	text (number)
1742	[smok_vu_v2]	Smoker	dropdown<br>1 Yes<br>2 No<br>3 Ex-smoker
1743	[hpv_vu_v2]	HPV status	dropdown<br>1 Positive<br>2 Negative<br>3 Not tested

1744	[hpv_typ_vu2]<br>Show the field ONLY if:<br>[hpv_vu_v2] = '1'	HPV type (select all that apply)	checkbox<br>1 hpv_typ_vu2___1 16<br>2 hpv_typ_vu2___2 18<br>3 hpv_typ_vu2___3 other<br>4 hpv_typ_vu2___4 Unknown
1745	[hpv_no_vu_v2]<br>Show the field ONLY if:<br>[hpv_vu_v2] = '3'	Reason not tested (select all that apply)	checkbox<br>1 hpv_no_vu_v2___1 Not clinical protocol<br>2 hpv_no_vu_v2___2 Patient refused<br>3 hpv_no_vu_v2___3 Patient unable to afford test<br>4 hpv_no_vu_v2___4 Testing not available locally<br>5 hpv_no_vu_v2___5 Other
1746	[ps_hpv_no_vu_v2]<br>Show the field ONLY if:<br>[hpv_no_vu_v2(5)] = '1'	Please state	text
1747	[vac_vu_v2]	HPV vaccine administered historically	yesno<br>1 Yes<br>0 No
1748	[vac_typ_vu_v2]<br>Show the field ONLY if:<br>[vac_vu_v2] = '1'	Type of vaccine administered	dropdown<br>1 Bivalent<br>2 Quadrivalent<br>3 9-valent
1749	[age_vac_vu_v2]<br>Show the field ONLY if:<br>[vac_vu_v2] = '1'	Age vaccine administered	text (number)
1750	[vac_no_reas_vu_v2]<br>Show the field ONLY if:<br>[vac_vu_v2] = '0'	Reason not administered (select all that apply)	checkbox<br>1 vac_no_reas_vu_v2___1 No national vaccination programme<br>2 vac_no_reas_vu_v2___2 National vaccination programme introduced after age of first sexual activity<br>3 vac_no_reas_vu_v2___3 Patient unable to afford<br>4 vac_no_reas_vu_v2___4 Other
1751	[ps_vac_no_reas_vu_v2]<br>Show the field ONLY if:<br>[vac_no_reas_vu_v2(4)] = '1'	Please state	text

1752	[asa_vu_v2]	Section Header: *PRE-OPERATIVE*<br>American Society of Anesthesiologists (ASA) physical status	dropdown<br>1 ASA 1: A normal healthy patient. Example: Fit, non-obese (BMI under 30), a nonsmoking patient with good exercise tolerance.<br>2 ASA 2: A patient with a mild systemic disease. Example: Patient with no functional limitations and a well-controlled disease (e.g., treated hypertension, obesity with BMI under 35, frequent social drinker or is a cigarette smoker).<br>3 ASA 3: A patient with a severe systemic disease that is not life-threatening. Example: Patient with some functional limitation as a result of disease (e.g., poorly treated hypertension or diabetes, morbid obesity, chronic renal failure, a bronchospastic disease with intermittent exacerbation, stable angina, implanted pacemaker).<br>4 ASA 4: A patient with a severe systemic disease that is a constant threat to life. Example: Patient with functional limitation from severe, life-threatening disease (e.g., unstable angina, poorly controlled COPD, symptomatic CHF, recent (less than three months ago) myocardial infarction or stroke.<br>5 ASA 5: A moribund patient who is not expected to survive without the operation. The patient is not expected to survive beyond the next 24 hours without surgery. Examples: ruptured abdominal aortic aneurysm, massive trauma, and extensive intracranial hemorrhage with mass effect.<br>6 ASA 6: A brain-dead patient whose organs are being removed with the intention of transplanting them into another patient.
1753	[ecog_vu_v2]	ECOG (Eastern Cooperative Oncology Group) performance status	dropdown<br>1 Grade 0 - Fully active, able to carry on all pre-disease performance without restriction<br>2 Grade 1 - Restricted in physically strenuous activity but ambulatory and able to carry out work of a light or sedentary nature, e.g., light house work, office work<br>3 Grade 2 - Ambulatory and capable of all selfcare but unable to carry out any work activities; up and about more than 50% of waking hours<br>4 Grade 3 - Capable of only limited selfcare; confined to bed or chair more than 50% of waking hours<br>5 Grade 4 - Completely disabled; cannot carry on any selfcare; totally confined to bed or chair<br>6 Grade 5 - Dead
1754	[comorb_vu2]	Co-morbidities	checkbox<br>1 comorb_vu2___1 Cardiovascular<br>2 comorb_vu2___2 Respiratory<br>3 comorb_vu2___3 Urogenital<br>4 comorb_vu2___4 Endocrine<br>5 comorb_vu2___5 Neurological<br>6 comorb_vu2___6 Haematological<br>7 comorb_vu2___7 Psychiatric<br>8 comorb_vu2___8 Gastroenterology<br>9 comorb_vu2___9 Rheumatology and musculoskeletal<br>10 comorb_vu2___10 Oncology<br>11 comorb_vu2___11 None

1755	[comorb_cvd_vu2]<br>Show the field ONLY if:<br>[comorb_vu2(1)] = '1'	Select all that apply	checkbox<br>1 comorb_cvd_vu2___1 Hypertension<br>2 comorb_cvd_vu2___2 Coronary heart disease / ischaemic heart disease<br>3 comorb_cvd_vu2___3 Myocardial infarction<br>4 comorb_cvd_vu2___4 Heart failure<br>5 comorb_cvd_vu2___5 Arrhythmia<br>6 comorb_cvd_vu2___6 Heart valve disease<br>7 comorb_cvd_vu2___7 Venous insufficiency<br>8 comorb_cvd_vu2___8 Peripheral vascular disease<br>9 comorb_cvd_vu2___9 Stroke<br>10 comorb_cvd_vu2___10 Transient ischaemic attack<br>11 comorb_cvd_vu2___11 Other
1756	[comorb_resp_vu2]<br>Show the field ONLY if:<br>[comorb_vu2(2)] = '1'	Select all that apply	checkbox<br>1 comorb_resp_vu2___1 Asthma<br>2 comorb_resp_vu2___2 Emphysema (COPD)<br>3 comorb_resp_vu2___3 Chronic bronchitis (COPD)<br>4 comorb_resp_vu2___4 Pulmonary fibrosis<br>5 comorb_resp_vu2___5 Sleep apnoea<br>6 comorb_resp_vu2___6 Bronchiectasis<br>7 comorb_resp_vu2___7 Cystic fibrosis<br>8 comorb_resp_vu2___8 Tuberculosis<br>9 comorb_resp_vu2___9 Pneumonia<br>10 comorb_resp_vu2___10 Other
1757	[comorb_uro_vu2]<br>Show the field ONLY if:<br>[comorb_vu2(3)] = '1'	Select all that apply	checkbox<br>1 comorb_uro_vu2___1 Chronic kidney disease (not requiring haemodialysis)<br>2 comorb_uro_vu2___2 Chronic kidney disease (requiring haemodialysis)<br>3 comorb_uro_vu2___3 Polycystic kidney disease<br>4 comorb_uro_vu2___4 Glomerulonephropathy<br>5 comorb_uro_vu2___5 Glomerulonephritis<br>6 comorb_uro_vu2___6 Urinary tract infection<br>7 comorb_uro_vu2___7 Other
1758	[comorb_endo_vu2]<br>Show the field ONLY if:<br>[comorb_vu2(4)] = '1'	Select all that apply	checkbox<br>1 comorb_endo_vu2___1 Type 1 diabetes mellitus<br>2 comorb_endo_vu2___2 Type 2 diabetes mellitus<br>3 comorb_endo_vu2___3 Hyperthyroidism<br>4 comorb_endo_vu2___4 Hypothyroidism<br>5 comorb_endo_vu2___5 Other
1759	[comorb_neuro_vu2]<br>Show the field ONLY if:<br>[comorb_vu2(5)] = '1'	Select all that apply	checkbox<br>1 comorb_neuro_vu2___1 Parkinsons disease<br>2 comorb_neuro_vu2___2 Myasthenia gravis<br>3 comorb_neuro_vu2___3 Dementia<br>4 comorb_neuro_vu2___4 Epilepsy<br>5 comorb_neuro_vu2___5 Other

1760	[comorb_haem_vu2]<br><br>Show the field ONLY if:<br>[comorb_vu2(6)] = '1'	Select all that apply	checkbox<br>1 comorb_haem_vu2___1 Iron deficiency anaemia<br>2 comorb_haem_vu2___2 Folate deficience anaemia<br>3 comorb_haem_vu2___3 Vitamin B12 deficiency anaemia<br>4 comorb_haem_vu2___4 Pernicious anaemia<br>5 comorb_haem_vu2___5 Haemolytic anaemia<br>6 comorb_haem_vu2___6 Aplastic anaemia<br>7 comorb_haem_vu2___7 Thalassemia<br>8 comorb_haem_vu2___8 Sickle cell disease<br>9 comorb_haem_vu2___9 Disseminated intravascular coagulation (DIC)<br>10 comorb_haem_vu2___10 Factor V Leiden<br>11 comorb_haem_vu2___11 Thrombocytosis<br>12 comorb_haem_vu2___12 Idiopathic thrombocytopenic purpura (ITP)<br>13 comorb_haem_vu2___13 Haemophilia<br>14 comorb_haem_vu2___14 Von Willebrand disease<br>15 comorb_haem_vu2___15 Antiphospholipid syndrome<br>16 comorb_haem_vu2___16 Thrombocytopenia<br>17 comorb_haem_vu2___17 Thrombotic thrombocytopenic purpura<br>18 comorb_haem_vu2___18 Deep vein thrombosis<br>19 comorb_haem_vu2___19 Pulmonary embolism<br>20 comorb_haem_vu2___20 Other
1761	[comorb_pscy_vu2]<br><br>Show the field ONLY if:<br>[comorb_vu2(7)] = '1'	Select all that apply	checkbox<br>1 comorb_pscy_vu2___1 Anxiety<br>2 comorb_pscy_vu2___2 Depression<br>3 comorb_pscy_vu2___3 Learning difficulties<br>4 comorb_pscy_vu2___4 Personality disorder<br>5 comorb_pscy_vu2___5 Schizophrenia<br>6 comorb_pscy_vu2___6 Bipolar disorder<br>7 comorb_pscy_vu2___7 Autism<br>8 comorb_pscy_vu2___8 Other

1762	[comorb_gi_vu_2]<br>Show the field ONLY if:<br>[comorb_vu2(8)] = '1'	Select all that apply	checkbox<br>1 comorb_gi_vu_2___1 Crohn's disease<br>2 comorb_gi_vu_2___2 Ulcerative colitis<br>3 comorb_gi_vu_2___3 Irritable bowel syndrome<br>4 comorb_gi_vu_2___4 Celiac Disease<br>5 comorb_gi_vu_2___5 Peptic ulcer disease<br>6 comorb_gi_vu_2___6 Gallstones<br>7 comorb_gi_vu_2___7 Pancreatitis<br>8 comorb_gi_vu_2___8 Diverticulitis<br>9 comorb_gi_vu_2___9 Non-Alcoholic Fatty Liver Disease<br>10 comorb_gi_vu_2___10 Alcoholic fatty liver disease<br>11 comorb_gi_vu_2___11 Viral hepatitis<br>12 comorb_gi_vu_2___12 Liver failure<br>13 comorb_gi_vu_2___13 Other
1763	[comorb_msk_vu_2]<br>Show the field ONLY if:<br>[comorb_vu2(9)] = '1'	Select all that apply	checkbox<br>1 comorb_msk_vu_2___1 Osteoarthritis<br>2 comorb_msk_vu_2___2 Rheumatoid arthritis<br>3 comorb_msk_vu_2___3 Osteoporosis<br>4 comorb_msk_vu_2___4 Lupus<br>5 comorb_msk_vu_2___5 Spondyloarthropathies<br>6 comorb_msk_vu_2___6 Sjogren's syndrome<br>7 comorb_msk_vu_2___7 Gout<br>8 comorb_msk_vu_2___8 Other
1764	[comorb_onc_vu_2]<br>Show the field ONLY if:<br>[comorb_vu2(10)] = '1'	Select all that apply	checkbox<br>1 comorb_onc_vu_2___1 Breast cancer<br>2 comorb_onc_vu_2___2 Lung cancer<br>3 comorb_onc_vu_2___3 Hepatobiliary cancer<br>4 comorb_onc_vu_2___4 Brain cancer<br>5 comorb_onc_vu_2___5 Leukemia<br>6 comorb_onc_vu_2___6 Lymphoma<br>7 comorb_onc_vu_2___7 Osteosarcoma<br>8 comorb_onc_vu_2___8 Other
1765	[lap_vu2]	Previous laparoscopic abdominal surgery	yesno<br>1 Yes<br>0 No
1766	[opn_vu2]	Previous laparotomy	yesno<br>1 Yes<br>0 No
1767	[mdt_vu_v2]	Discussed at multidisciplinary team meeting/tumour board?	yesno<br>1 Yes<br>0 No

1768	[mdt_tm_vu2]<br>Show the field ONLY if:<br>[mdt_vu_v2] = '1'	Multidisciplinary team/tumour board members (select all that apply)	checkbox<br>1 mdt_tm_vu2___1 Certified gynaecological oncology surgeon<br>2 mdt_tm_vu2___2 Trained surgeon dedicated to the management of gynaecologic cancer or having completed an ESGO-accredited fellowship<br>3 mdt_tm_vu2___3 Radiologist<br>4 mdt_tm_vu2___4 Radiation/medical oncologist (administering radiotherapy)<br>5 mdt_tm_vu2___5 Medical oncologist (administering chemotherapy)<br>6 mdt_tm_vu2___6 Pathologist
1769	[mdt_vu_no_v2]<br>Show the field ONLY if:<br>[mdt_vu_v2] = '0'	Reason not discussed (Select all that apply)	checkbox<br>1 mdt_vu_no_v2___1 No multidisciplinary team meeting/tumour board<br>2 mdt_vu_no_v2___2 Other
1770	[ps_mdt_vc_no_v2]<br>Show the field ONLY if:<br>[mdt_vu_no_v2(2)] = '1'	Please state	text
1771	[figo_vu_v2]	FIGO stage	dropdown<br>1 1A Lesions ≤2 cm in size, confined to the vulva or perineum and with stromal invasion ≤1.0 mm, no nodal metastasis<br>2 1B Lesions >2 cm in size or with stromal invasion >1.0 mm, confined to the vulva or perineum, with negative nodes<br>3 II Tumor of any size with extension to adjacent perineal structures (lower third of urethra, lower third of vagina, anus) with negative nodes<br>4 IIIA Tumor of any size with or without extension to adjacent perineal structures (lower third of urethra, lower third of vagina, anus) with positive inguinofemoral nodes (with 1 lymph node metastasis (≥5 mm), or with 1-2 lymph node metastasis(es) (< 5 mm))<br>5 IIIB Tumor of any size with or without extension to adjacent perineal structures (lower third of urethra, lower third of vagina, anus) with positive inguinofemoral nodes (with 2 or more lymph node metastases (≥5 mm), or with 3 or more lymph node metastases (< 5 mm))<br>6 IIIC Tumor of any size with or without extension to adjacent perineal structures (lower third of urethra, lower third of vagina, anus) with positive inguinofemoral nodes with extracapsular spread<br>7 IVA Tumor invades any of the following: upper urethral and/or vaginal mucosa, bladder mucosa, rectal mucosa, or fixed to pelvic bone, or fixed or ulcerated inguinofemoral lymph nodes<br>10 IVB Any distant metastasis including pelvic lymph nodes
1772	[bx_vu_v2]	Diagnostic biopsy performed at recurrence?	yesno<br>1 Yes<br>0 No

1773	[bx_no_reas_vu_v2]<br>Show the field ONLY if:<br>[bx_vu_v2] = '0'	Reason diagnostic biopsy not performed at recurrence (select all that apply)	checkbox<br>1 bx_no_reas_vu_v2___1 Biopsy not technically feasible<br>2 bx_no_reas_vu_v2___2 Patient refused<br>3 bx_no_reas_vu_v2___3 Patient unable to perform<br>4 bx_no_reas_vu_v2___4 Not available locally<br>5 bx_no_reas_vu_v2___5 Other
1774	[ps_bx_no_reas_vu_v2]<br>Show the field ONLY if:<br>[bx_no_reas_vu_v2(5)] = '1'	Please state	text
1775	[mri_vu_v2]	Magnetic resonance imaging (MRI) pelvis/abdominal-pelvis performed at recurrence diagnosis?	yesno<br>1 Yes<br>0 No
1776	[mri_find_vu_v2]<br>Show the field ONLY if:<br>[mri_vu_v2] = '1'	MRI pelvis/abdominal-pelvis findings at recurrence diagnosis (select all that apply)	checkbox<br>1 mri_find_vu_v2___1 Vulval tumour < 2cm<br>2 mri_find_vu_v2___2 Vulval tumour >2cm<br>3 mri_find_vu_v2___3 Perineal invasion<br>4 mri_find_vu_v2___4 Vaginal invasion (lower third)<br>5 mri_find_vu_v2___5 Vaginal invasion (upper two thirds)<br>6 mri_find_vu_v2___6 Anal invasion<br>7 mri_find_vu_v2___7 Rectal invasion<br>8 mri_find_vu_v2___8 Sigmoid invasion<br>9 mri_find_vu_v2___9 Urethral invasion (lower third)<br>10 mri_find_vu_v2___10 Urethral invasion (upper two thirds)<br>11 mri_find_vu_v2___11 Bladder invasion<br>12 mri_find_vu_v2___12 x1 hydroureter<br>13 mri_find_vu_v2___13 x2 hydroureter<br>14 mri_find_vu_v2___14 Ureteric invasion<br>15 mri_find_vu_v2___15 x1 hydronephrosis<br>16 mri_find_vu_v2___16 x2 hydronephrosis<br>17 mri_find_vu_v2___17 Pelvic bone invasion<br>18 mri_find_vu_v2___18 Inguinofemoral lymphadenopathy<br>19 mri_find_vu_v2___19 Pelvic lymphadenopathy<br>20 mri_find_vu_v2___20 Para-aortic lymphadenopathy<br>21 mri_find_vu_v2___21 Levator ani muscle invasion<br>22 mri_find_vu_v2___22 Other
1777	[ps_mri_find_vu_v2]<br>Show the field ONLY if:<br>[mri_find_vu_v2(22)] = '1'	Please state	text
1778	[mri_no_vu_v2]<br>Show the field ONLY if:<br>[mri_vu_v2] = '0'	Reason MRI pelvis not performed at recurrence diagnosis (select all that apply)	checkbox<br>1 mri_no_vu_v2___1 No access to MRI machine<br>2 mri_no_vu_v2___2 Patient unable to afford<br>3 mri_no_vu_v2___3 Patient refused<br>4 mri_no_vu_v2___4 Claustrophobia<br>5 mri_no_vu_v2___5 Other imaging performed<br>6 mri_no_vu_v2___6 Extreme morbid obesity<br>7 mri_no_vu_v2___7 Other

1779	[ps_mri_vu_no_v2]<br>Show the field ONLY if:<br>[mri_no_vu_v2(7)] = '1'	Please state	text
1780	[ct_vu_v2]	Staging computerised tomography (CT) performed at recurrence diagnosis?	yesno<br>1 Yes<br>0 No
1781	[ct_find_vu_v2]<br>Show the field ONLY if:<br>[ct_vu_v2] = '1'	Staging CT findings at diagnosis (select all that apply)	checkbox<br>1 ct_find_vu_v2___1 Vulval tumour < 2cm<br>2 ct_find_vu_v2___2 Vulval tumour >2cm<br>3 ct_find_vu_v2___3 Perineal invasion<br>4 ct_find_vu_v2___4 Vaginal invasion (lower third)<br>5 ct_find_vu_v2___5 Vaginal invasion (upper two thirds)<br>6 ct_find_vu_v2___6 Anal invasion<br>7 ct_find_vu_v2___7 Rectal invasion<br>8 ct_find_vu_v2___8 Sigmoid invasion<br>9 ct_find_vu_v2___9 Urethral invasion (lower third)<br>10 ct_find_vu_v2___10 Urethral invasion (upper two thirds)<br>11 ct_find_vu_v2___11 Bladder invasion<br>12 ct_find_vu_v2___12 x1 hydroureter<br>13 ct_find_vu_v2___13 x2 hydroureter<br>14 ct_find_vu_v2___14 Ureteric invasion<br>15 ct_find_vu_v2___15 x1 hydronephrosis<br>16 ct_find_vu_v2___16 x2 hydronephrosis<br>17 ct_find_vu_v2___17 Pelvic bone invasion<br>18 ct_find_vu_v2___18 Inguinofemoral lymphadenopathy<br>19 ct_find_vu_v2___19 Pelvic lymphadenopathy<br>20 ct_find_vu_v2___20 Para-aortic lymphadenopathy<br>21 ct_find_vu_v2___21 Levator ani muscle invasion<br>22 ct_find_vu_v2___22 Lung nodules<br>23 ct_find_vu_v2___23 Pleural effusion<br>24 ct_find_vu_v2___24 Other
1782	[ps_ct_find_vu_v2]<br>Show the field ONLY if:<br>[ct_find_vu_v2(24)] = '1'	Please state	text
1783	[ct_vu_reas_no_v2]<br>Show the field ONLY if:<br>[ct_vu_v2] = '0'	Reason staging CT not performed at recurrence diagnosis	checkbox<br>1 ct_vu_reas_no_v2___1 No access to CT machine<br>2 ct_vu_reas_no_v2___2 Patient unable to afford<br>3 ct_vu_reas_no_v2___3 Patient refused<br>4 ct_vu_reas_no_v2___4 Other imaging performed<br>5 ct_vu_reas_no_v2___5 Allergic reaction to contrast<br>6 ct_vu_reas_no_v2___6 Chronic kidney disease<br>7 ct_vu_reas_no_v2___7 Other
1784	[ps_ct_vu_reas_no_v2]<br>Show the field ONLY if:<br>[ct_vu_reas_no_v2(7)] = '1'	Please state	text
1785	[petct_vu_v2]	PET-CT performed at recurrence diagnosis?	yesno<br>1 Yes<br>0 No

1786	[petct_find_vu_v2]<br>Show the field ONLY if:<br>[petct_vu_v2] = '1'	PET-CT findings at diagnosis (select all that apply)	checkbox<br>1 petct_find_vu_v2___1 FDG-avid uptake in vulva<br>2 petct_find_vu_v2___2 FDG-avid uptake in vagina<br>3 petct_find_vu_v2___3 FDG-avid uptake in pelvic bones<br>4 petct_find_vu_v2___4 FDG-avid uptake in inguinofemoral lymph nodes<br>5 petct_find_vu_v2___5 FDG-avid uptake pelvic lymph nodes<br>6 petct_find_vu_v2___6 FDG-avid uptake in para-aortic nodes<br>7 petct_find_vu_v2___7 FDG-avid urethra<br>8 petct_find_vu_v2___8 FDG-avid uptake in x1 ureter<br>9 petct_find_vu_v2___9 FDG-avid uptake in x2 ureters<br>10 petct_find_vu_v2___10 FDG-avid uptake in x1 kidney<br>11 petct_find_vu_v2___11 FDG-avid uptake in x2 kidneys<br>12 petct_find_vu_v2___12 FDG-avid uptake in bladder<br>13 petct_find_vu_v2___13 FDG-avid uptake in para rectal nodules<br>14 petct_find_vu_v2___14 FDG-avid uptake in sigmoid nodules<br>15 petct_find_vu_v2___15 FDG-avid uptake in rectum<br>16 petct_find_vu_v2___16 FDG-avid uptake in sigmoid<br>17 petct_find_vu_v2___17 FDG-avid anus<br>18 petct_find_vu_v2___18 FDG-avid uptake in lung nodules<br>19 petct_find_vu_v2___19 FDG-avid uptake levator ani<br>20 petct_find_vu_v2___20 Other
1787	[ps_petct_find_vu_v2]<br>Show the field ONLY if:<br>[petct_find_vu_v2(20)] = '1'	Please state	text
1788	[petct_no_reas_vu_v2]<br>Show the field ONLY if:<br>[petct_vu_v2] = '0'	Reason PET-CT not performed at diagnosis (select all that apply)	checkbox<br>1 petct_no_reas_vu_v2___1 No access to PET machine<br>2 petct_no_reas_vu_v2___2 Patient unable to afford<br>3 petct_no_reas_vu_v2___3 Patient refused<br>4 petct_no_reas_vu_v2___4 Uncontrolled diabetes mellitus<br>5 petct_no_reas_vu_v2___5 Other imaging performed<br>6 petct_no_reas_vu_v2___6 Not clinical protocol<br>7 petct_no_reas_vu_v2___7 Other
1789	[ps_petct_no_reas_vu_v2]<br>Show the field ONLY if:<br>[petct_no_reas_vu_v2(7)] = '1'	Please state	text
1790	[uss_vu_v2]	Ultrasound scan groins	yesno<br>1 Yes<br>0 No

1791	[uss_no_vu_v2]<br>Show the field ONLY if:<br>[uss_vu_v2] = '0'	Reason not performed (select all that apply)	checkbox<br>1 uss_no_vu_v2___1 Not clinically indicated<br>2 uss_no_vu_v2___2 Other imaging performed<br>3 uss_no_vu_v2___3 Not available locally<br>4 uss_no_vu_v2___4 Patient refused<br>5 uss_no_vu_v2___5 Patient unable to afford<br>6 uss_no_vu_v2___6 Other
1792	[ps_uss_no_vu_v2]<br>Show the field ONLY if:<br>[uss_no_vu_v2(6)] = '1'	Please state	text
1793	[ln_siz_uss_vu_v2]<br>Show the field ONLY if:<br>[uss_vu_v2] = '1'	Lymph nodes size	radio<br>1 < 1.5cm<br>2 ≥1.5cm
1794	[fna_uss_vu_v2]<br>Show the field ONLY if:<br>[ln_siz_uss_vu_v2] = '2'	Fine needle aspiration	yesno<br>1 Yes<br>0 No
1795	[fna_res_vu_v2]<br>Show the field ONLY if:<br>[fna_uss_vu_v2] = '1'	Fine needle aspiration results	dropdown<br>1 Positive<br>2 Negative
1796	[fna_no_vu_v2]<br>Show the field ONLY if:<br>[fna_uss_vu_v2] = '0'	Reason not performed (select all that apply)	checkbox<br>1 fna_no_vu_v2___1 Not technically feasible<br>2 fna_no_vu_v2___2 Other imaging performed<br>3 fna_no_vu_v2___3 Not available locally<br>4 fna_no_vu_v2___4 Patient refused<br>5 fna_no_vu_v2___5 Patient unable to afford<br>6 fna_no_vu_v2___6 Other
1797	[ps_fna_no_vu_v2]<br>Show the field ONLY if:<br>[fna_no_vu_v2(6)] = '1'	Please state	text
1798	[cov19_vu_v2]	COVID-19 status	dropdown<br>1 Positive<br>2 Negative<br>3 Not tested
1799	[cov_notst_vu_v2]<br>Show the field ONLY if:<br>[cov19_vu_v2] = '3'	If not tested select reason (select all that apply)	checkbox<br>1 cov_notst_vu_v2___1 Not clinically indicated (asymptomatic)<br>2 cov_notst_vu_v2___2 Not clinical protocol to routinely test all patients prior to surgery<br>3 cov_notst_vu_v2___3 Patient refused<br>4 cov_notst_vu_v2___4 Patient unable to afford test<br>5 cov_notst_vu_v2___5 No local resources for testing<br>6 cov_notst_vu_v2___6 Other
1800	[ps_cov_notst_vu_v2]<br>Show the field ONLY if:<br>[cov_notst_vu_v2(6)] = '1'	Please state	text
1801	[bp_vu2]	Bowel preparation pre-operatively	yesno<br>1 Yes<br>0 No
1802	[peri_op_vu2]	Peri-operative management plan in place? (Non- exhaustive list: pre-operative haemoglobin optimisation and iron deficit correction; correction of denutrition and immunonutrition; fluid management; pain management; prevention of post-operative nausea and vomiting).	yesno<br>1 Yes<br>0 No
1803	[hb_vu2]	Pre-operative Haemoglobin (g/L)	text (number)

1804	[wcc_vu2]	Pre-operative White Cell Count (10^9/L)	text (number)
1805	[plt_vu2]	Pre-operative Platelets (10^9/L)	text (number)
1806	[pt_vu2]	Pre-operative PT (Secs)	text (number)
1807	[inr_vu2]	Pre-operative INR (ratio)	text (number)
1808	[aptt_vu2]	Pre-operative APTT (Secs)	text (number)
1809	[fib_vu2]	Pre-operative Fibrinogen (g/L)	text (number)
1810	[alb_vu2]	Pre-operative Albumin (g/L)	text (number)
1811	[na_vu2]	Pre-operative Sodium (mmol/L)	text (number)
1812	[k_vu2]	Pre-operative Potassium (mmol/L)	text (number)
1813	[ur_vu2]	Pre-operative Urea (mmol/L)	text (number)
1814	[creat_vu2]	Pre-operative Creatinine (µmol/L)	text (number)
1815	[egfr_vu2]	Pre-operative eGFR - estimated glomerular filtration rate (ml/min)	text
1816	[bili_vu2]	Pre-operative Bilirubin (µmol/L)	text (number)
1817	[aat_vu2]	Pre-operative Alanine Aminotransaminase (IU/L)	text (number)
1818	[alp_vu2]	Pre-operative Alkaline Phosphatase (IU/L)	text (number)
1819	[ast_vu2]	Pre-operative Aspartate Aminotransaminase (IU/L)	text (number)
1820	[crp_vu2]	Pre-operative CRP - C-reactive protein (mg/L)	text (number)
1821	[surgdat_vu_v2]	Section Header: *SURGERY*<br>Date of recurrence surgery	text (date_dmy)
1822	[anaes_vu_v2]	Anaesthesia (select all that apply)	checkbox<br>1 anaes_vu_v2___1 General<br>2 anaes_vu_v2___2 Spinal<br>3 anaes_vu_v2___3 Epidural
1823	[surg_elecem_vu_v2]	Was the recurrence surgery an elective or emergency case?	radio<br>1 Elective<br>2 Emergency
1824	[sa_vu_v2]	Surgical aim	dropdown<br>1 Curative<br>2 Curative attempted but then abandoned (i.e. open/close laparotomy)<br>3 Palliative
1825	[ls_vu_v2]	Lead surgeon performing recurrence surgery	dropdown<br>1 Certified consultant/specialist gynaecological oncologist<br>2 Fellow/trainee gynaecological oncologist<br>3 Trained surgeon dedicated to the management of gynaecologic cancer or having completed an ESGO accredited fellowship.<br>4 General surgeon<br>5 Surgical oncologist<br>6 Other
1826	[ps_ls_vu_v2]<br>Show the field ONLY if:<br>[ls_vu_v2] = '6'	Please state	text
1827	[who_vu2]	WHO surgical safety checklist used	yesno<br>1 Yes<br>0 No
1828	[sm_vu_v2]	Recurrence surgical procedure (select all that apply)	checkbox<br>1 sm_vu_v2___1 Wide local excision<br>2 sm_vu_v2___2 Groin lymph node dissection<br>3 sm_vu_v2___3 Vulvectomy<br>4 sm_vu_v2___4 Pelvic lymph node dissection<br>5 sm_vu_v2___5 Infralevator extenteration

1829	[cyto_vu2]	Complete macroscopic cytoreduction at surgery	yesno<br>1 Yes<br>0 No
1830	[reas_cytored_no_vu2]<br>Show the field ONLY if:<br>[cyto_vu2] = '0'	Reason(s) disease not fully removed at recurrence cytoreductive surgery (select all that apply)	checkbox<br>1 reas_cytored_no_vu2___1 Disease more extensive than that shown on imaging<br>2 reas_cytored_no_vu2___2 Technically unable to resect<br>3 reas_cytored_no_vu2___3 Patient became medically unstable intra-operatively and decision made to not proceed further with surgery<br>4 reas_cytored_no_vu2___4 Other
1831	[ps_reas_cytored_no_vu2]<br>Show the field ONLY if:<br>[reas_cytored_no_vu2(4)] = '1'	Please state	text
1832	[siz_resid_vu2]<br>Show the field ONLY if:<br>[cyto_vu2] = '0'	Size of residual disease (in millimetres)	dropdown<br>1 1-2.5<br>2 2.6-5<br>3 5.1-7.5<br>4 7.6-10<br>5 >10
1833	[loc_resid_vu2]<br>Show the field ONLY if:<br>[cyto_vu2] = '0'	Location of residual disease at recurrence surgery (Select all that apply)	checkbox<br>1 loc_resid_vu2___1 Vulva<br>2 loc_resid_vu2___2 Vagina (lower third)<br>3 loc_resid_vu2___3 Vagina (upper two thirds)<br>4 loc_resid_vu2___4 Pelvic bone<br>5 loc_resid_vu2___5 Urethra (lower third)<br>6 loc_resid_vu2___6 Urethra (upper two thirds)<br>7 loc_resid_vu2___7 Bladder<br>8 loc_resid_vu2___8 ureter(s)<br>9 loc_resid_vu2___9 kidney(s)<br>10 loc_resid_vu2___10 Anus<br>11 loc_resid_vu2___11 Rectum<br>12 loc_resid_vu2___12 Sigmoid<br>13 loc_resid_vu2___13 Para-aortic-lymphadenopathy<br>14 loc_resid_vu2___14 Inguinofemoral lyphadenopathy<br>15 loc_resid_vu2___15 Pelvic lymphadenopathy<br>16 loc_resid_vu2___16 Levator ani<br>17 loc_resid_vu2___17 Other
1834	[ps_loc_resid_vu2]<br>Show the field ONLY if:<br>[loc_resid_vu2(17)] = '1'	Please state	text

1835	[surg_find_vu_v2]	Recurrence surgical findings (select all that apply)	checkbox<br>1 surg_find_vu_v2___1 Vulval tumour < 2cm<br>2 surg_find_vu_v2___2 Vulval tumour >2cm<br>3 surg_find_vu_v2___3 Vaginal invasion (lower third)<br>4 surg_find_vu_v2___4 Vaginal invasion (upper two thirds)<br>5 surg_find_vu_v2___5 Pelvic bone invasion<br>6 surg_find_vu_v2___6 Urethral invasion (lower third)<br>7 surg_find_vu_v2___7 Urethral invasion (upper two thirds)<br>8 surg_find_vu_v2___8 Bladder invasion<br>9 surg_find_vu_v2___9 x1 hydroureter<br>10 surg_find_vu_v2___10 x2 hydroureter<br>11 surg_find_vu_v2___11 Ureteric invasion<br>12 surg_find_vu_v2___12 x1 hydronephrosis<br>13 surg_find_vu_v2___13 x2 hydronephrosis<br>14 surg_find_vu_v2___14 Anal invasion<br>15 surg_find_vu_v2___15 Rectal invasion<br>16 surg_find_vu_v2___16 Sigmoid invasion<br>17 surg_find_vu_v2___17 Para-aortic-lymphadenopathy<br>18 surg_find_vu_v2___18 Inguinofemoral lyphadenopathy<br>19 surg_find_vu_v2___19 Pelvic lymphadenopathy<br>20 surg_find_vu_v2___20 Levator ani invasion<br>21 surg_find_vu_v2___21 Other<br>22 surg_find_vu_v2___22 No visible disease
1836	[ps_surg_find_vu_v2]<br>Show the field ONLY if:<br>[surg_find_vu_v2(21)] = '1'	Please state	text
1837	[tis_vu_v2]	Tissue removed (Select all that apply)	checkbox<br>1 tis_vu_v2___1 Vulva<br>2 tis_vu_v2___2 Cervix<br>3 tis_vu_v2___3 Uterus<br>4 tis_vu_v2___4 x1 fallopian tube<br>5 tis_vu_v2___5 x2 fallopian tubes<br>6 tis_vu_v2___6 x1 ovary<br>7 tis_vu_v2___7 x2 ovaries<br>8 tis_vu_v2___8 Inguinofemaral lymph nodes<br>9 tis_vu_v2___9 Pelvic lymph nodes<br>10 tis_vu_v2___10 Para-aortic lymph nodes<br>11 tis_vu_v2___11 Anus<br>12 tis_vu_v2___12 Rectum<br>13 tis_vu_v2___13 Sigmoid<br>14 tis_vu_v2___14 Kidney<br>15 tis_vu_v2___15 Ureter<br>16 tis_vu_v2___16 Bladder<br>17 tis_vu_v2___17 Levator ani<br>18 tis_vu_v2___18 Other
1838	[paln_dis_vu2]<br>Show the field ONLY if:<br>[tis_vu_v2(10)] = '1'	Select all that apply	checkbox<br>1 paln_dis_vu2___1 Infra-renal<br>2 paln_dis_vu2___2 Supra-renal

1839	[ps_tis_vu_v2]<br>Show the field ONLY if:<br>[tis_vu_v2(18)] = '1'	Please state	text
1840	[si_stom_vu_v2]	Small bowel stoma	yesno<br>1 Yes<br>0 No
1841	[ss_vu2]<br>Show the field ONLY if:<br>[si_stom_vu_v2] = '1'	Stoma site	dropdown<br>1 Duodenum<br>2 Jejunum<br>3 Ileum
1842	[pb_si_stom_vu_v2]<br>Show the field ONLY if:<br>[si_stom_vu_v2] = '1'	Performed by (select all that apply)	checkbox<br>1 pb_si_stom_vu_v2___1 Gynaecological oncologist<br>2 pb_si_stom_vu_v2___2 Colorectal surgeon<br>3 pb_si_stom_vu_v2___3 Urologist<br>4 pb_si_stom_vu_v2___4 General surgeon<br>5 pb_si_stom_vu_v2___5 Plastic surgeon<br>6 pb_si_stom_vu_v2___6 Other
1843	[ps_pb_si_stom_vu_v2]<br>Show the field ONLY if:<br>[pb_si_stom_vu_v2(6)] = '1'	Please state	text
1844	[si_anast_vu_v2]	Small bowel anastamosis	yesno<br>1 Yes<br>0 No
1845	[pb_si_anast_vu_v2]<br>Show the field ONLY if:<br>[si_anast_vu_v2] = '1'	Performed by (select all that apply)	checkbox<br>1 pb_si_anast_vu_v2___1 Gynaecological oncologist<br>2 pb_si_anast_vu_v2___2 Colorectal surgeon<br>3 pb_si_anast_vu_v2___3 Urologist<br>4 pb_si_anast_vu_v2___4 General surgeon<br>5 pb_si_anast_vu_v2___5 Plastic surgeon<br>6 pb_si_anast_vu_v2___6 Other
1846	[ps_pb_si_anast_vu_v2]<br>Show the field ONLY if:<br>[pb_si_anast_vu_v2(6)] = '1'	Please state	text
1847	[li_stom_vu_v2]	Large bowel stoma	yesno<br>1 Yes<br>0 No
1848	[pb_li_stom_vu_v2]<br>Show the field ONLY if:<br>[li_stom_vu_v2] = '1'	Performed by (select all that apply)	checkbox<br>1 pb_li_stom_vu_v2___1 Gynaecological oncologist<br>2 pb_li_stom_vu_v2___2 Colorectal surgeon<br>3 pb_li_stom_vu_v2___3 Urologist<br>4 pb_li_stom_vu_v2___4 General surgeon<br>5 pb_li_stom_vu_v2___5 Plastic surgeon<br>6 pb_li_stom_vu_v2___6 Other
1849	[ps_pb_li_stom_vu_v2]<br>Show the field ONLY if:<br>[pb_li_stom_vu_v2(6)] = '1'	Please state	text
1850	[li_anas_vu_v2]	Large bowel anastamosis	yesno<br>1 Yes<br>0 No

1851	[pb_li_anast_vu_v2]<br>Show the field ONLY if:<br>[li_anas_vu_v2] = '1'	Performed by (select all that apply)	checkbox<br>1 pb_li_anast_vu_v2___1 Gynaecological oncologist<br>2 pb_li_anast_vu_v2___2 Colorectal surgeon<br>3 pb_li_anast_vu_v2___3 Urologist<br>4 pb_li_anast_vu_v2___4 General surgeon<br>5 pb_li_anast_vu_v2___5 Plastic surgeon<br>6 pb_li_anast_vu_v2___6 Other
1852	[ps_pb_li_anast_vu_v2]<br>Show the field ONLY if:<br>[pb_li_anast_vu_v2(6)] = '1'	Please state	text
1853	[urost_vu_v2]	Urostomy/ileal conduit	yesno<br>1 Yes<br>0 No
1854	[pb_urost_vu_v2]<br>Show the field ONLY if:<br>[urost_vu_v2] = '1'	Performed by (select all that apply)	checkbox<br>1 pb_urost_vu_v2___1 Gynaecological oncologist<br>2 pb_urost_vu_v2___2 Colorectal surgeon<br>3 pb_urost_vu_v2___3 Urologist<br>4 pb_urost_vu_v2___4 General surgeon<br>5 pb_urost_vu_v2___5 Plastic surgeon<br>6 pb_urost_vu_v2___6 Other
1855	[ps_pb_urost_vu_v2]<br>Show the field ONLY if:<br>[pb_urost_vu_v2(6)] = '1'	Please state	text
1856	[tuu_vu_v2]	Trans uretero-ureteric anastamosis	yesno<br>1 Yes<br>0 No
1857	[pb_tuu_vu_v2]<br>Show the field ONLY if:<br>[tuu_vu_v2] = '1'	Performed by (select all that apply)	checkbox<br>1 pb_tuu_vu_v2___1 Gynaecological oncologist<br>2 pb_tuu_vu_v2___2 Colorectal surgeon<br>3 pb_tuu_vu_v2___3 Urologist<br>4 pb_tuu_vu_v2___4 General surgeon<br>5 pb_tuu_vu_v2___5 Plastic surgeon<br>6 pb_tuu_vu_v2___6 Other
1858	[ps_pb_tuu_vu_v2]<br>Show the field ONLY if:<br>[pb_tuu_vu_v2(6)] = '1'	Please state	text
1859	[ui_vu_v2]	Ureteric reimplantation	yesno<br>1 Yes<br>0 No
1860	[pb_ui_vu_v2]<br>Show the field ONLY if:<br>[ui_vu_v2] = '1'	Performed by (select all that apply)	checkbox<br>1 pb_ui_vu_v2___1 Gynaecological oncologist<br>2 pb_ui_vu_v2___2 Colorectal surgeon<br>3 pb_ui_vu_v2___3 Urologist<br>4 pb_ui_vu_v2___4 General surgeon<br>5 pb_ui_vu_v2___5 Plastic surgeon<br>6 pb_ui_vu_v2___6 Other
1861	[ps_pb_ui_vu_v2]<br>Show the field ONLY if:<br>[pb_ui_vu_v2(6)] = '1'	Please state	text
1862	[neph_vu_v2]	Nephrostomy	yesno<br>1 Yes<br>0 No

1863	[pb_neph_vu_v2]<br>Show the field ONLY if:<br>[neph_vu_v2] = '1'	Performed by (select all that apply)	checkbox<br>1 pb_neph_vu_v2___1 Gynaecological oncologist<br>2 pb_neph_vu_v2___2 Colorectal surgeon<br>3 pb_neph_vu_v2___3 Urologist<br>4 pb_neph_vu_v2___4 General surgeon<br>5 pb_neph_vu_v2___5 Plastic surgeon<br>6 pb_neph_vu_v2___6 Other
1864	[ps_pb_neph_vu_v2]<br>Show the field ONLY if:<br>[pb_neph_vu_v2(6)] = '1'	Please state	text
1865	[vr_vu_v2]	Vulval reconstruction	yesno<br>1 Yes<br>0 No
1866	[vrf_vu_v2]<br>Show the field ONLY if:<br>[vr_vu_v2] = '1'	Type of vulval reconstruction	radio<br>1 Rotational flap<br>2 Non rotational flap<br>3 Graft
1867	[adsurg_vu_v2]<br>Show the field ONLY if:<br>[vr_vu_v2] = '1'	Performed by (select all that apply)	checkbox<br>1 adsurg_vu_v2___1 Gynaecological oncologist<br>2 adsurg_vu_v2___2 Colorectal surgeon<br>3 adsurg_vu_v2___3 Urologist<br>4 adsurg_vu_v2___4 General surgeon<br>5 adsurg_vu_v2___5 Plastic surgeon<br>6 adsurg_vu_v2___6 Other
1868	[ps_adsurg_vu_v2]<br>Show the field ONLY if:<br>[adsurg_vu_v2(6)] = '1'	Please state	text
1869	[abx_vu2]	Intraoperative antibiotics	yesno<br>1 Yes<br>0 No
1870	[tim_vu_v2]	Length of recurrence surgery	text (time)
1871	[itu_vu2]	Section Header: *POST-OPERATIVE*<br>Post-operative recovery in intensive care unit	yesno<br>1 Yes<br>0 No
1872	[itu_dys_vu2]<br>Show the field ONLY if:<br>[itu_vu2] = '1'	Number of days in intensive care unit	text (number)
1873	[hdu_vu2]	Post-operative recovery in high dependency unit	yesno<br>1 Yes<br>0 No
1874	[hdu_dys_vu2]<br>Show the field ONLY if:<br>[hdu_vu2] = '1'	Number of days in high dependency unit	text (number)
1875	[er_vu_v2]	Enhanced recovery?	yesno<br>1 Yes<br>0 No
1876	[po_abx_vu2]	Prophylactic post operative antibiotics	yesno<br>1 Yes<br>0 No
1877	[drn_vu2]	Post operative surgical drain	yesno<br>1 Yes<br>0 No

1878	[drn_dys_vu2]<br>Show the field ONLY if:<br>[drn_vu2] = '1'	Number of days in situ	text (number)
1879	[uc_vu2]	Post operative urinary catheter	yesno<br>1 Yes<br>0 No
1880	[cath_dys_vu2]<br>Show the field ONLY if:<br>[uc_vu2] = '1'	Number of days in situ	text (number)
1881	[phb_vu_2]	Post-operative Haemoglobin (g/L) - please state first post-operative result	text (number)
1882	[pwbc_vu_2]	Post-operative White Cell Count (10^9/L) - please state first post-operative result	text (number)
1883	[pplat_vu_2]	Post-operative Platelets (10^9/L) - please state first post-operative result	text (number)
1884	[ppt_vu_2]	Post-operative PT (Secs) - please state first post-operative result	text (number)
1885	[pinr_vu_2]	Post-operative INR (Ratio) - please state first post-operative result	text (number)
1886	[paptt_vu_2]	Post-operative APTT (Secs) - please state first post-operative result	text (number)
1887	[pfib_vu_2]	Post-operative Fibrinogen (g/L) - please state first post-operative result	text (number)
1888	[palb_vu_2]	Post-operative Albumin (g/L) - please state first post-operative result	text (number)
1889	[pna_vu_2]	Post-operative Sodium (mmol/L) - please state first post-operative result	text (number)
1890	[pk_vu_2]	Post-operative Potassium (mmol/L) - please state first post-operative result	text (number)
1891	[purea_vu_2]	Post-operative Urea (mmol/L) - please state first post-operative result	text (number)
1892	[pcreat_vu_2]	Post-operative Creatinine (µmol/L) - please state first post-operative result	text
1893	[pegfr_vu_2]	Post-operative eGFR - Estimated Glomerular Filtration Rate (mL/min) - please state first post-operative result	text
1894	[pbili_vu_2]	Post-operative Bilirubin total (µmol/L) - please state first post-operative result	text (number)
1895	[paat_vu_2]	Post-operative Alanine Aminotransaminase<br>(IU/L) - please state first post-operative result	text (number)
1896	[palp_vu_2]	Post-operative Alkaline Phosphatase (IU/L) - please state first post-operative result	text (number)
1897	[past_vu_2]	Post-operative Aspartate Aminotransaminase (IU/L) - please state first post-operative result	text (number)
1898	[pcrp_vu_2]	Post-operative CRP - C-reactive protein (mg/L) - please state first post-operative result	text (number)
1899	[hist_vu_v2]	Histologic type	dropdown<br>1 Squamous cell<br>2 Malignant melanoma<br>3 Adenocarcinoma<br>4 Basal cell carcinoma<br>5 Sarcoma<br>6 Pagets with invasion
1900	[scc_uni_multi_vu_v2]<br>Show the field ONLY if:<br>[hist_vu_v2] = '1'	Unifocal or multifocal	dropdown<br>1 Unifocal<br>2 Multifocal
1901	[lichs_vu_v2]	Vulval carcinoma on a background of lichen sclerosus	yesno<br>1 Yes<br>0 No

1902	[ts_ls_vu_v2]<br>Show the field ONLY if:<br>[lichs_vu_v2] = '1'	Maintenance dose of topical steroids (e.g. clobetasol propionate)	yesno<br>1 Yes<br>0 No
1903	[resect_vu2]	Histological confirmed resection margins following recurrence surgery	dropdown<br>1 < 1 mm / tumour on margin<br>2 1-5 mm (NO tumour on margin)<br>3 >5 mm Margins confirmed clear<br>4 Unknown, not available in this hospital
1904	[dis_marg_vu_2]	Disease at margins	dropdown<br>1 Vulval intraepithelial neoplasia I<br>2 Vulval intraepithelial neoplasia II<br>3 Vulval intraepithelial neoplasia III<br>4 Invasion<br>5 Disease free (i.e. no VIN/invasion)
1905	[p16_vu_v2]	Section Header: *Immunohistochemistry*<br>p16	radio (Matrix)<br>1 Positive<br>2 Negative<br>3 Not tested
1906	[p53_vu_v2]	p53	radio (Matrix)<br>1 Positive<br>2 Negative<br>3 Not tested
1907	[ck7_vu_v2]	CK7	radio (Matrix)<br>1 Positive<br>2 Negative<br>3 Not tested
1908	[ck20_vu_v2]	CK20	radio (Matrix)<br>1 Positive<br>2 Negative<br>3 Not tested
1909	[cea_vu_v2]	CEA	radio (Matrix)<br>1 Positive<br>2 Negative<br>3 Not tested
1910	[ck8_18_vu_v2]	CK8/18	radio (Matrix)<br>1 Positive<br>2 Negative<br>3 Not tested
1911	[s100_vu_v2]	S100	radio (Matrix)<br>1 Positive<br>2 Negative<br>3 Not tested
1912	[melan_a_vu_v2]	Melan A	radio (Matrix)<br>1 Positive<br>2 Negative<br>3 Not tested
1913	[cam5_2_vu_v2]	CAM5.2	radio (Matrix)<br>1 Positive<br>2 Negative<br>3 Not tested

1914	[muc2_vu_v2]	MUC-2	radio (Matrix)<br>1 Positive<br>2 Negative<br>3 Not tested
1915	[uro_vu_v2]	uroplakin III	radio (Matrix)<br>1 Positive<br>2 Negative<br>3 Not tested
1916	[hmb45_vu_v2]	HMB 45	radio (Matrix)<br>1 Positive<br>2 Negative<br>3 Not tested
1917	[ac_vu_v2]	Chemotherapy administered following recurrence diagnosis	yesno<br>1 Yes<br>0 No
1918	[adjchem1_reg_vu_v2]<br>Show the field ONLY if:<br>[ac_vu_v2] = '1'	Chemotherapy regimen (select all that apply)	checkbox<br>1 adjchem1_reg_vu_v2___1 Platinum (for example cisplatin, carboplatin)<br>2 adjchem1_reg_vu_v2___2 Fluorouracil<br>3 adjchem1_reg_vu_v2___3 Taxane (for example paclitaxel)<br>4 adjchem1_reg_vu_v2___4 Vinorelbine<br>5 adjchem1_reg_vu_v2___5 Erlotinib<br>6 adjchem1_reg_vu_v2___6 Clinical trial regimens<br>7 adjchem1_reg_vu_v2___7 Other
1919	[ps_adjcem1_reg_vu_v2]<br>Show the field ONLY if:<br>[adjchem1_reg_vu_v2(7)] = '1'	Please state	text
1920	[adjchem_cycl_numb_v2]<br>Show the field ONLY if:<br>[ac_vu_v2] = '1'	Number of cycles planned	text (number)
1921	[adjchem_no_reas_vu_v2]<br>Show the field ONLY if:<br>[ac_vu_v2] = '0'	Reason chemotherapy not administered following recurrence diagnosis (select all that apply)	checkbox<br>1 adjchem_no_reas_vu_v2___1 Poor performance status<br>2 adjchem_no_reas_vu_v2___2 No access locally<br>3 adjchem_no_reas_vu_v2___3 Patient unable to afford<br>4 adjchem_no_reas_vu_v2___4 Patient refused<br>5 adjchem_no_reas_vu_v2___5 Not clinically indicated<br>6 adjchem_no_reas_vu_v2___6 Other
1922	[ps_adjchemo_no_reas_vu_v2]<br>Show the field ONLY if:<br>[adjchem_no_reas_vu_v2(6)] = '1'	Please state	text
1923	[rt_vu_v2]	External beam radiotherapy administered following recurrence diagnosis	yesno<br>1 Yes<br>0 No
1924	[rt_dos_vu2]<br>Show the field ONLY if:<br>[rt_vu_v2] = '1'	Dose received (Gray - Gy)	text (number)
1925	[rt_site_vu_v2]<br>Show the field ONLY if:<br>[rt_vu_v2] = '1'	External beam radiotherapy administered to	dropdown<br>1 Vulva<br>2 Groins<br>3 Vulva and groins

1926	[rt_no_vu_v2]<br>Show the field ONLY if:<br>[rt_vu_v2] = '0'	Reason radiotherapy not administered following recurrence diagnosis (select all that apply)	checkbox<br>1 rt_no_vu_v2___1 Poor performance status<br>2 rt_no_vu_v2___2 Not available locally<br>3 rt_no_vu_v2___3 Patient unable to afford<br>4 rt_no_vu_v2___4 Patient refused<br>5 rt_no_vu_v2___5 Not clinically indicated<br>6 rt_no_vu_v2___6 Other
1927	[ps_rt_no_vu_v2]<br>Show the field ONLY if:<br>[rt_no_vu_v2(6)] = '1'	Please state	text
1928	[brachy2_vu]	Brachytherapy administered after recurrence diagnosis	yesno<br>1 Yes<br>0 No
1929	[rt_dos_vu3]<br>Show the field ONLY if:<br>[brachy2_vu] = '1'	Dose received (Gray - Gy)	text (number)
1930	[brachy_no_reas_vu2]<br>Show the field ONLY if:<br>[brachy2_vu] = '0'	Reason brachytherapy not administered after recurrence surgery	checkbox<br>1 brachy_no_reas_vu2___1 Poor performance status<br>2 brachy_no_reas_vu2___2 Not available locally<br>3 brachy_no_reas_vu2___3 Patient unable to afford<br>4 brachy_no_reas_vu2___4 Patient refused<br>5 brachy_no_reas_vu2___5 Not clinically indicated<br>6 brachy_no_reas_vu2___6 Other
1931	[ps_brachy_no_reas_vu2]<br>Show the field ONLY if:<br>[brachy_no_reas_vu2(6)] = '1'	Please state	text
1932	[vulval_cancer_recurrence_complete]	Section Header: *Form Status*<br>Complete?	dropdown<br>0 Incomplete<br>1 Unverified<br>2 Complete

Instrument: **Vaginal cancer (initial presentation)** (vaginal_cancer_initial_presentation)

1933	[dat_vag]	Section Header: *BASELINE DEMOGRAPHICS*<br>Date of primary diagnosis	text (date_dmy)
1934	[age_vag]	Age of patient	text (number)
1935	[eth_vag]	Ethnicity	dropdown<br>1 White (British, Australian, North American, European, other white)<br>2 Asian (South East Asian, Far East Asian, other Asian)<br>3 Black (African, Caribbean, other Black)<br>4 Middle Eastern (Arab, North African, Turkish)<br>5 Mixed (multiple ethnic background)<br>6 Other
1936	[ps_eth_vag]<br>Show the field ONLY if:<br>[eth_vag] = '6'	Please state	text
1937	[bmi_vag]	Body mass index (kg/m2)	text (number)
1938	[smok_vag]	Smoker	dropdown<br>1 Yes<br>2 No<br>3 Ex-smoker

1939	[hpv_vag]	HPV status	dropdown<br>1 Positive<br>2 Negative<br>3 Not tested
1940	[hpv_typ_vag]<br>Show the field ONLY if:<br>[hpv_vag] = '1'	HPV type (select all that apply)	checkbox<br>1 hpv_typ_vag___1 16<br>2 hpv_typ_vag___2 18<br>3 hpv_typ_vag___3 other<br>4 hpv_typ_vag___4 Unknown
1941	[hpv_vag_no_reas]<br>Show the field ONLY if:<br>[hpv_vag] = '3'	Reason not done (select all that apply)	checkbox<br>1 hpv_vag_no_reas___1 Not clinical protocol<br>2 hpv_vag_no_reas___2 Patient refused<br>3 hpv_vag_no_reas___3 Patient unable to afford test<br>4 hpv_vag_no_reas___4 Testing not available locally<br>5 hpv_vag_no_reas___5 Other
1942	[ps_hpv_vag_no_reas]<br>Show the field ONLY if:<br>[hpv_vag_no_reas(5)] = '1'	Please state	text
1943	[vac_vag]	HPV vaccine administered historically	yesno<br>1 Yes<br>0 No
1944	[typ_vac_vag]<br>Show the field ONLY if:<br>[vac_vag] = '1'	Type of vaccine administered	radio<br>1 Bivalent<br>2 Quadrivalent<br>3 9-valent
1945	[age_vac_vag]<br>Show the field ONLY if:<br>[vac_vag] = '1'	Age vaccine administered	text (number)
1946	[vac_no_reas_vag]<br>Show the field ONLY if:<br>[vac_vag] = '0'	Reason vaccine not administered (select all that apply)	checkbox<br>1 vac_no_reas_vag___1 No national vaccination programme<br>2 vac_no_reas_vag___2 National vaccination programme introduced after age of first sexual activity<br>3 vac_no_reas_vag___3 Patient unable to afford<br>4 vac_no_reas_vag___4 Other
1947	[ps_vac_no_reas_vag]<br>Show the field ONLY if:<br>[vac_no_reas_vag(4)] = '1'	Please state	text

1948	[asa_vag]	Section Header: *PRE-OPERATIVE*<br>American Society of Anesthesiologists (ASA) physical status	dropdown<br>1 ASA 1: A normal healthy patient. Example: Fit, non-obese (BMI under 30), a nonsmoking patient with good exercise tolerance.<br>2 ASA 2: A patient with a mild systemic disease. Example: Patient with no functional limitations and a well-controlled disease (e.g., treated hypertension, obesity with BMI under 35, frequent social drinker or is a cigarette smoker).<br>3 ASA 3: A patient with a severe systemic disease that is not life-threatening. Example: Patient with some functional limitation as a result of disease (e.g., poorly treated hypertension or diabetes, morbid obesity, chronic renal failure, a bronchospastic disease with intermittent exacerbation, stable angina, implanted pacemaker).<br>4 ASA 4: A patient with a severe systemic disease that is a constant threat to life. Example: Patient with functional limitation from severe, life-threatening disease (e.g., unstable angina, poorly controlled COPD, symptomatic CHF, recent (less than three months ago) myocardial infarction or stroke.<br>5 ASA 5: A moribund patient who is not expected to survive without the operation. The patient is not expected to survive beyond the next 24 hours without surgery. Examples: ruptured abdominal aortic aneurysm, massive trauma, and extensive intracranial hemorrhage with mass effect.<br>6 ASA 6: A brain-dead patient whose organs are being removed with the intention of transplanting them into another patient.
1949	[ecog_vag]	ECOG (Eastern Cooperative Oncology Group) performance status	dropdown<br>1 Grade 0 - Fully active, able to carry on all pre-disease performance without restriction<br>2 Grade 1 - Restricted in physically strenuous activity but ambulatory and able to carry out work of a light or sedentary nature, e.g., light house work, office work<br>3 Grade 2 - Ambulatory and capable of all selfcare but unable to carry out any work activities; up and about more than 50% of waking hours<br>4 Grade 3 - Capable of only limited selfcare; confined to bed or chair more than 50% of waking hours<br>5 Grade 4 - Completely disabled; cannot carry on any selfcare; totally confined to bed or chair<br>6 Grade 5 - Dead
1950	[comorb_vag]	Co-morbidities	checkbox<br>1 comorb_vag___1 Cardiovascular<br>2 comorb_vag___2 Respiratory<br>3 comorb_vag___3 Urogenital<br>4 comorb_vag___4 Endocrine<br>5 comorb_vag___5 Neurological<br>6 comorb_vag___6 Haematological<br>7 comorb_vag___7 Psychiatric<br>8 comorb_vag___8 Gastroenterology<br>9 comorb_vag___9 Rheumatology and musculoskeletal<br>10 comorb_vag___10 Oncology<br>11 comorb_vag___11 None

1951	[comorb_cvd_vag]<br>Show the field ONLY if:<br>[comorb_vag(1)] = '1'	Select all that apply	checkbox<br>1 comorb_cvd_vag___1 Hypertension<br>2 comorb_cvd_vag___2 Coronary heart disease / ischaemic heart disease<br>3 comorb_cvd_vag___3 Myocardial infarction<br>4 comorb_cvd_vag___4 Heart failure<br>5 comorb_cvd_vag___5 Arrhythmia<br>6 comorb_cvd_vag___6 Heart valve disease<br>7 comorb_cvd_vag___7 Venous insufficiency<br>8 comorb_cvd_vag___8 Peripheral vascular disease<br>9 comorb_cvd_vag___9 Stroke<br>10 comorb_cvd_vag___10 Transient ischaemic attack<br>11 comorb_cvd_vag___11 Other
1952	[comorb_resp_vag]<br>Show the field ONLY if:<br>[comorb_vag(2)] = '1'	Select all that apply	checkbox<br>1 comorb_resp_vag___1 Asthma<br>2 comorb_resp_vag___2 Emphysema (COPD)<br>3 comorb_resp_vag___3 Chronic bronchitis (COPD)<br>4 comorb_resp_vag___4 Pulmonary fibrosis<br>5 comorb_resp_vag___5 Sleep apnoea<br>6 comorb_resp_vag___6 Bronchiectasis<br>7 comorb_resp_vag___7 Cystic fibrosis<br>8 comorb_resp_vag___8 Tuberculosis<br>9 comorb_resp_vag___9 Pneumonia<br>10 comorb_resp_vag___10 Other
1953	[comorb_uro_vag]<br>Show the field ONLY if:<br>[comorb_vag(3)] = '1'	Select all that apply	checkbox<br>1 comorb_uro_vag___1 Chronic kidney disease (not requiring haemodialysis)<br>2 comorb_uro_vag___2 Chronic kidney disease (requiring haemodialysis)<br>3 comorb_uro_vag___3 Polycystic kidney disease<br>4 comorb_uro_vag___4 Glomerulonephropathy<br>5 comorb_uro_vag___5 Glomerulonephritis<br>6 comorb_uro_vag___6 Urinary tract infection<br>7 comorb_uro_vag___7 Other
1954	[comorb_endo_vag]<br>Show the field ONLY if:<br>[comorb_vag(4)] = '1'	Select all that apply	checkbox<br>1 comorb_endo_vag___1 Type 1 diabetes mellitus<br>2 comorb_endo_vag___2 Type 2 diabetes mellitus<br>3 comorb_endo_vag___3 Hyperthyroidism<br>4 comorb_endo_vag___4 Hypothyroidism<br>5 comorb_endo_vag___5 Other
1955	[comorb_neuro_vag]<br>Show the field ONLY if:<br>[comorb_vag(5)] = '1'	Select all that apply	checkbox<br>1 comorb_neuro_vag___1 Parkinsons disease<br>2 comorb_neuro_vag___2 Myasthenia gravis<br>3 comorb_neuro_vag___3 Dementia<br>4 comorb_neuro_vag___4 Epilepsy<br>5 comorb_neuro_vag___5 Other

1956	[comorb_haem_vag]<br>Show the field ONLY if:<br>[comorb_vag(6)] = '1'	Select all that apply	checkbox<br>1 comorb_haem_vag___1 Iron deficiency anaemia<br>2 comorb_haem_vag___2 Folate deficience anaemia<br>3 comorb_haem_vag___3 Vitamin B12 deficiency anaemia<br>4 comorb_haem_vag___4 Pernicious anaemia<br>5 comorb_haem_vag___5 Haemolytic anaemia<br>6 comorb_haem_vag___6 Aplastic anaemia<br>7 comorb_haem_vag___7 Thalassemia<br>8 comorb_haem_vag___8 Sickle cell disease<br>9 comorb_haem_vag___9 Disseminated intravascular coagulation (DIC)<br>10 comorb_haem_vag___10 Factor V Leiden<br>11 comorb_haem_vag___11 Thrombocytosis<br>12 comorb_haem_vag___12 Idiopathic thrombocytopenic purpura (ITP)<br>13 comorb_haem_vag___13 Haemophilia<br>14 comorb_haem_vag___14 Von Willebrand disease<br>15 comorb_haem_vag___15 Antiphospholipid syndrome<br>16 comorb_haem_vag___16 Thrombocytopenia<br>17 comorb_haem_vag___17 Thrombotic thrombocytopenic purpura<br>18 comorb_haem_vag___18 Deep vein thrombosis<br>19 comorb_haem_vag___19 Pulmonary embolism<br>20 comorb_haem_vag___20 Other
1957	[comorb_gi_vag]<br>Show the field ONLY if:<br>[comorb_vag(8)] = '1'	Select all that apply	checkbox<br>1 comorb_gi_vag___1 Crohn's disease<br>2 comorb_gi_vag___2 Ulcerative colitis<br>3 comorb_gi_vag___3 Irritable bowel syndrome<br>4 comorb_gi_vag___4 Celiac Disease<br>5 comorb_gi_vag___5 Peptic ulcer disease<br>6 comorb_gi_vag___6 Gallstones<br>7 comorb_gi_vag___7 Pancreatitis<br>8 comorb_gi_vag___8 Diverticulitis<br>9 comorb_gi_vag___9 Non-Alcoholic Fatty Liver Disease<br>10 comorb_gi_vag___10 Alcoholic fatty liver disease<br>11 comorb_gi_vag___11 Viral hepatitis<br>12 comorb_gi_vag___12 Liver failure<br>13 comorb_gi_vag___13 Other
1958	[comorb_pscy_vag]<br>Show the field ONLY if:<br>[comorb_vag(7)] = '1'	Select all that apply	checkbox<br>1 comorb_pscy_vag___1 Anxiety<br>2 comorb_pscy_vag___2 Depression<br>3 comorb_pscy_vag___3 Learning difficulties<br>4 comorb_pscy_vag___4 Personality disorder<br>5 comorb_pscy_vag___5 Schizophrenia<br>6 comorb_pscy_vag___6 Bipolar disorder<br>7 comorb_pscy_vag___7 Autism<br>8 comorb_pscy_vag___8 Other

1959	[comorb_msk_vag]<br>Show the field ONLY if:<br>[comorb_vag(9)] = '1'	Select all that apply	checkbox<br>1 comorb_msk_vag___1 Osteoarthritis<br>2 comorb_msk_vag___2 Rheumatoid arthritis<br>3 comorb_msk_vag___3 Osteoporosis<br>4 comorb_msk_vag___4 Lupus<br>5 comorb_msk_vag___5 Spondyloarthropathies<br>6 comorb_msk_vag___6 Sjogren's syndrome<br>7 comorb_msk_vag___7 Gout<br>8 comorb_msk_vag___8 Other
1960	[comorb_onc_vag]<br>Show the field ONLY if:<br>[comorb_vag(10)] = '1'	Select all that apply	checkbox<br>1 comorb_onc_vag___1 Breast cancer<br>2 comorb_onc_vag___2 Lung cancer<br>3 comorb_onc_vag___3 Hepatobiliary cancer<br>4 comorb_onc_vag___4 Brain cancer<br>5 comorb_onc_vag___5 Leukemia<br>6 comorb_onc_vag___6 Lymphoma<br>7 comorb_onc_vag___7 Osteosarcoma<br>8 comorb_onc_vag___8 Other
1961	[lap_vag]	Previous laparoscopic abdominal surgery	yesno<br>1 Yes<br>0 No
1962	[opn_vag]	Previous laparotomy	yesno<br>1 Yes<br>0 No
1963	[mdt_vag]	Discussed at multidisciplinary team meeting/tumour board?	yesno<br>1 Yes<br>0 No
1964	[mdt_tm_vag]<br>Show the field ONLY if:<br>[mdt_vag] = '1'	Multidisciplinary team/tumour board members (select all that apply)	checkbox<br>1 mdt_tm_vag___1 Certified gynaecological oncology surgeon<br>2 mdt_tm_vag___2 Trained surgeon dedicated to the management of gynaecologic cancer or having completed an ESGO-accredited fellowship<br>3 mdt_tm_vag___3 Radiologist<br>4 mdt_tm_vag___4 Radiation/clinical oncologist (administering radiotherapy)<br>5 mdt_tm_vag___5 Medical oncologist (administering chemotherapy)<br>6 mdt_tm_vag___6 Pathologist
1965	[mdt_vagc_no]<br>Show the field ONLY if:<br>[mdt_vag] = '0'	Reason not discussed (select all that apply)	checkbox<br>1 mdt_vagc_no___1 No multidisciplinary team meeting/tumour board<br>2 mdt_vagc_no___2 Other
1966	[ps_mdt_vagc_no]<br>Show the field ONLY if:<br>[mdt_vagc_no(2)] = '1'	Please state	text
1967	[bx_vag]	Diagnostic biopsy performed?	yesno<br>1 Yes<br>0 No

1968	[bx_vag_no_reas]<br>Show the field ONLY if:<br>[bx_vag] = '0'	Reason diagnostic biopsy not performed (Select all that apply)	checkbox<br>1 bx_vag_no_reas___1 Biopsy not technically feasible<br>2 bx_vag_no_reas___2 Patient refused<br>3 bx_vag_no_reas___3 Unable to perform<br>4 bx_vag_no_reas___4 No resources available locally to perform<br>5 bx_vag_no_reas___5 Other
1969	[ps_bx_vag_no_reas]<br>Show the field ONLY if:<br>[bx_vag_no_reas(5)] = '1'	Please state	text
1970	[mri_vag]	Magnetic resonance imaging (MRI) pelvis/abdominal-pelvis performed at diagnosis?	yesno<br>1 Yes<br>0 No
1971	[mri_find_vag]<br>Show the field ONLY if:<br>[mri_vag] = '1'	MRI pelvis/abdominal-pelvis findings at diagnosis (select all that apply)	checkbox<br>1 mri_find_vag___1 Vaginal tumour < 2cm<br>2 mri_find_vag___2 Vaginal tumour >2cm<br>3 mri_find_vag___3 Vulval invasion<br>4 mri_find_vag___4 Cervical invasion<br>5 mri_find_vag___5 Uterus invasion<br>6 mri_find_vag___6 Urethral invasion (lower third)<br>7 mri_find_vag___7 Urethral invasion (upper two thirds)<br>8 mri_find_vag___8 x1 hydroureter<br>9 mri_find_vag___9 x2 hydroureter<br>10 mri_find_vag___10 Ureteric invasion<br>11 mri_find_vag___11 x1 hydronephrosis<br>12 mri_find_vag___12 x2 hydronephrosis<br>13 mri_find_vag___13 Bladder invasion<br>14 mri_find_vag___14 Anal invasion<br>15 mri_find_vag___15 Rectal invasion<br>16 mri_find_vag___16 Sigmoid invasion<br>17 mri_find_vag___17 Pelvic bone invasion<br>18 mri_find_vag___18 Inguinal lymphadenopathy<br>19 mri_find_vag___19 Pelvic lymphadenopathy<br>20 mri_find_vag___20 Para-aortic lymphadenopathy<br>21 mri_find_vag___21 Other
1972	[ps_mri_find_vag]<br>Show the field ONLY if:<br>[mri_find_vag(21)] = '1'	Please state	text
1973	[mri_vag_no_reas]<br>Show the field ONLY if:<br>[mri_vag] = '0'	Reason MRI not performed at diagnosis (select all that apply)	checkbox<br>1 mri_vag_no_reas___1 No access to MRI machine<br>2 mri_vag_no_reas___2 Patient unable to afford<br>3 mri_vag_no_reas___3 Patient refused<br>4 mri_vag_no_reas___4 Claustrophobia<br>5 mri_vag_no_reas___5 Other imaging performed<br>6 mri_vag_no_reas___6 Extreme morbid obesity<br>7 mri_vag_no_reas___7 Other
1974	[ps_mri_vag_no_reas]<br>Show the field ONLY if:<br>[mri_vag_no_reas(7)] = '1'	Please state	text

1975	[ct_vag]	Staging computerised tomography (CT) performed at diagnosis?	yesno<br>1 Yes<br>0 No
1976	[ct_vag_find]<br>Show the field ONLY if:<br>[ct_vag] = '1'	Staging CT findings at diagnosis (Select all that apply)	checkbox<br>1 ct_vag_find___1 Vaginal tumour < 2cm<br>2 ct_vag_find___2 Vaginal tumour >2cm<br>3 ct_vag_find___3 Vulval invasion<br>4 ct_vag_find___4 Cervical invasion<br>5 ct_vag_find___5 Uterus invasion<br>6 ct_vag_find___6 Urethral invasion (lower third)<br>7 ct_vag_find___7 Urethral invasion (upper two thirds)<br>8 ct_vag_find___8 x1 hydroureter<br>9 ct_vag_find___9 x2 hydroureter<br>10 ct_vag_find___10 Ureteric invasion<br>11 ct_vag_find___11 x1 hydronephrosis<br>12 ct_vag_find___12 x2 hydronephrosis<br>13 ct_vag_find___13 Bladder invasion<br>14 ct_vag_find___14 Anal invasion<br>15 ct_vag_find___15 Rectal invasion<br>16 ct_vag_find___16 Sigmoid invasion<br>17 ct_vag_find___17 Pelvic bone invasion<br>18 ct_vag_find___18 Inguinal lymphadenopathy<br>19 ct_vag_find___19 Pelvic lymphadenopathy<br>20 ct_vag_find___20 Para-aortic lymphadenopathy<br>21 ct_vag_find___21 Lung nodules<br>22 ct_vag_find___22 Pleural effusion<br>23 ct_vag_find___23 Other
1977	[ps_ct_vag_find]<br>Show the field ONLY if:<br>[ct_vag_find(23)] = '1'	Please state	text
1978	[ct_vag_no_reas]<br>Show the field ONLY if:<br>[ct_vag] = '0'	Reason staging CT not performed at diagnosis (Select all that apply)	checkbox<br>1 ct_vag_no_reas___1 No access to CT machine<br>2 ct_vag_no_reas___2 Patient unable to afford<br>4 ct_vag_no_reas___4 Patient refused<br>5 ct_vag_no_reas___5 Other imaging performed<br>6 ct_vag_no_reas___6 Allergic reaction to contrast<br>7 ct_vag_no_reas___7 Chronic kidney disease<br>3 ct_vag_no_reas___3 Other
1979	[ps_ct_vag_no_reas]<br>Show the field ONLY if:<br>[ct_vag_no_reas(3)] = '1'	Please state	text
1980	[pet_vag]	PET-CT performed at diagnosis?	yesno<br>1 Yes<br>0 No

1981	[pet_vag_find]<br>Show the field ONLY if:<br>[pet_vag] = '1'	PET-CT findings at diagnosis (Select all that apply)	checkbox<br>1 pet_vag_find___1 FDG-avid uptake vagina<br>2 pet_vag_find___2 FDG-avid uptake vulva<br>3 pet_vag_find___3 FDG-avid uptake cervix<br>4 pet_vag_find___4 FDG-avid uptake uterus<br>5 pet_vag_find___5 FDG-avid uptake urethra<br>6 pet_vag_find___6 FDG-avid uptake ureter(s)<br>7 pet_vag_find___7 FDG-avid uptake kidney(s)<br>8 pet_vag_find___8 FDG-avid uptake bladder<br>9 pet_vag_find___9 FDG-avid uptake anus<br>10 pet_vag_find___10 FDG-avid uptake rectum<br>11 pet_vag_find___11 FDG-avid uptake sigmoid<br>12 pet_vag_find___12 FDG-avid uptake pelvic bones<br>13 pet_vag_find___13 FDG-avid uptake inguinal lymph nodes<br>14 pet_vag_find___14 FDG-avid uptake pelvic lymph nodes<br>15 pet_vag_find___15 FDG-avid uptake para-aortic lymph nodes<br>16 pet_vag_find___16 Other
1982	[ps_pet_vag_find]<br>Show the field ONLY if:<br>[pet_vag_find(16)] = '1'	Please state	text
1983	[pet_vag_no_reas]<br>Show the field ONLY if:<br>[pet_vag] = '0'	Reason PET-CT not performed (select all that apply)	checkbox<br>1 pet_vag_no_reas___1 No access to PET machine<br>2 pet_vag_no_reas___2 Patient unable to afford<br>3 pet_vag_no_reas___3 Patient refused<br>4 pet_vag_no_reas___4 Uncontrolled diabetes mellitus<br>5 pet_vag_no_reas___5 Other imaging performed<br>6 pet_vag_no_reas___6 Not clinical protocol<br>7 pet_vag_no_reas___7 Other
1984	[ps_pet_vag_no_reas]<br>Show the field ONLY if:<br>[pet_vag_no_reas(7)] = '1'	Please state	text
1985	[figo_vag]	Clinical FIGO Stage (i.e. preoperative staging taking into account imaging, clinical examination)	dropdown<br>1 1A Invasion confined to vagina, with tumour < 2cm wide and < 1mm depth of invasion<br>2 1B Invasion confined to vagina, with tumour >2cm wide and >1mm depth of invasion<br>3 Stage 2 Tumour invades para-vaginal tissues but not to the pelvic wall<br>4 Stage 3 Extension to the pelvic wall<br>5 Stage 4A Extension beyond true pelvis or invasion of bladder/rectum<br>6 Stage 4B Pelvic or inguinal lymphadenopathy or distant metastases
1986	[nact_vag]	Neoadjuvant (before surgery) chemotherapy	yesno<br>1 Yes<br>0 No

1987	[nactreg1_vag]<br>Show the field ONLY if:<br>[nact_vag] = '1'	Chemotherapy regimen FIRST line (Select all that apply)	checkbox<br>1 nactreg1_vag___1 Platinum (for example cisplatin, carboplatin)<br>2 nactreg1_vag___2 Fluorouracil<br>3 nactreg1_vag___3 Taxane (for example paclitaxel)<br>4 nactreg1_vag___4 Irenotecan<br>5 nactreg1_vag___5 Clinical trial regimens<br>6 nactreg1_vag___6 Other
1988	[ps_nactreg1_vag]<br>Show the field ONLY if:<br>[nactreg1_vag(6)] = '1'	Please state	text
1989	[nact_cycl_vag]<br>Show the field ONLY if:<br>[nact_vag] = '1'	Total number of cycles received	text (number)
1990	[nact_vag_no]<br>Show the field ONLY if:<br>[nact_vag] = '0'	Reason neoadjuvant chemotherapy not administered (Select all that apply)	checkbox<br>1 nact_vag_no___1 Poor performance status<br>2 nact_vag_no___2 No access<br>3 nact_vag_no___3 Patient unable to afford<br>4 nact_vag_no___4 Patient refused<br>5 nact_vag_no___5 Not clinically indicated<br>6 nact_vag_no___6 Other
1991	[ps_nact_vag_no]<br>Show the field ONLY if:<br>[nact_vag_no(6)] = '1'	Please state	text
1992	[cov19_vag]	COVID-19 status	dropdown<br>1 Positive<br>2 Negative<br>3 Not tested
1993	[cov19_vag_no_reas]<br>Show the field ONLY if:<br>[cov19_vag] = '3'	Reason not tested (select all that apply)	checkbox<br>1 cov19_vag_no_reas___1 Not clinically indicated (asymptomatic)<br>2 cov19_vag_no_reas___2 Not clinical protocol to routinely test all patients prior to surgery<br>3 cov19_vag_no_reas___3 Patient refused<br>4 cov19_vag_no_reas___4 Patient unable to afford test<br>5 cov19_vag_no_reas___5 No resources locally for testing<br>6 cov19_vag_no_reas___6 Other
1994	[ps_cov19_vag_no_reas]<br>Show the field ONLY if:<br>[cov19_vag_no_reas(6)] = '1'	Please state	text
1995	[bp_vag]	Bowel preparation pre-operatively	yesno<br>1 Yes<br>0 No
1996	[peri_op_vag]	Peri-operative management plan in place? (Non- exhaustive list: pre-operative haemoglobin optimisation and iron deficit correction; correction of denutrition and immunonutrition; fluid management; pain management; prevention of post-operative nausea and vomiting).	yesno<br>1 Yes<br>0 No
1997	[hb_vag]	Pre-operative Haemoglobin (g/L)	text (number)
1998	[wcc_vag]	Pre-operative White Cell Count (10^9/L)	text (number)
1999	[plt_vag]	Pre-operative Platelets (10^9/L)	text (number)
2000	[pt_vag]	Pre-operative PT (Secs)	text (number)
2001	[inr_vag]	Pre-operative INR (ratio)	text (number)

2002	[aptt_vag]	Pre-operative APTT (Secs)	text (number)
2003	[fib_vag]	Pre-operative Fibrinogen (g/L)	text (number)
2004	[alb_vag]	Pre-operative Albumin (g/L)	text (number)
2005	[na_vag]	Pre-operative Sodium (mmol/L)	text (number)
2006	[k_vag]	Pre-operative Potassium (mmol/L)	text (number)
2007	[ur_vag]	Pre-operative Urea (mmol/L)	text (number)
2008	[creat_vag]	Pre-operative Creatinine (μmol/L)	text (number)
2009	[egfr_vag]	Pre-operative eGFR - estimated glomerular filtration rate (ml/min)	text
2010	[bili_vag]	Pre-operative Bilirubin total (μmol/L)	text (number)
2011	[aat_vag]	Pre-operative Alanine Aminotransaminase (IU/L)	text (number)
2012	[alp_vag]	Pre-operative Alkaline Phosphatase (IU/L)	text (number)
2013	[ast_vag]	Pre-operative Aspartate Aminotransaminase (IU/L)	text (number)
2014	[crp_vag]	Pre-operative CRP - C-reactive protein (mg/L)	text (number)
2015	[datsurg_vag]	Section Header: *SURGERY*<br>Date of surgery	text (date_dmy)
2016	[anaes_vag]	Anaesthesia (Select all that apply)	checkbox<br>1 anaes_vag___1 General<br>2 anaes_vag___2 Spinal<br>3 anaes_vag___3 Epidural
2017	[elecem_vag]	Was the surgery an elective or emergency case?	radio<br>1 Elective<br>2 Emergency
2018	[sa_vag]	Surgical aim	dropdown<br>1 Curative<br>2 Curative attempted but then abandoned (i.e. open/close laparotomy)<br>3 Palliative
2019	[sm_vag]	Surgical modality (select all that apply)	checkbox<br>1 sm_vag___1 Vaginal<br>2 sm_vag___2 Laparotomy
2020	[ls_vag]	Lead surgeon performing surgery	dropdown<br>1 Certified consultant/specialist gynaecological oncologist<br>2 Fellow/trainee gynaecological oncologist<br>3 Trained surgeon dedicated to the management of gynaecologic cancer or having completed an ESGO accredited fellowship<br>4 General surgeon<br>5 Surgical oncologist<br>6 Other
2021	[ps_ls_vag]<br>Show the field ONLY if:<br>[ls_vag] = '6'	Please state	text
2022	[who_vag]	WHO surgical safety checklist used	yesno<br>1 Yes<br>0 No
2023	[cyto_vag]	Complete macroscopic cytoreduction at surgery	yesno<br>1 Yes<br>0 No

<table>
<tr><td>2024</td><td>[reas_cytored_no_vag]
Show the field ONLY if:
[cyto_vag] = '0'</td><td>Reason(s) disease not fully resected at cytodeuction (select all that apply)</td><td>checkbox<table><tr><td>1</td><td>reas_cytored_no_vag___1</td><td>Disease more extensive than that shown on imaging</td></tr><tr><td>2</td><td>reas_cytored_no_vag___2</td><td>Technically unable to resect</td></tr><tr><td>3</td><td>reas_cytored_no_vag___3</td><td>Patient became medically unstable intra-operatively and decision made to not proceed further with surgery</td></tr><tr><td>4</td><td>reas_cytored_no_vag___4</td><td>Other</td></tr></table></td></tr>
<tr><td>2025</td><td>[ps_reas_cytored_no_vag]
Show the field ONLY if:
[reas_cytored_no_vag(4)] = '1'</td><td>Please state</td><td>text</td></tr>
<tr><td>2026</td><td>[siz_resid_vag]
Show the field ONLY if:
[cyto_vag] = '0'</td><td>Size of residual disease (in millimetres)</td><td>dropdown<table><tr><td>1</td><td>1-2.5</td></tr><tr><td>2</td><td>2.6-5</td></tr><tr><td>3</td><td>5.1-7.5</td></tr><tr><td>4</td><td>7.6-10</td></tr><tr><td>5</td><td>>10</td></tr></table></td></tr>
<tr><td>2027</td><td>[loc_resid_vag]
Show the field ONLY if:
[cyto_vag] = '0'</td><td>Location of residual disease (select all that apply)</td><td>checkbox<table><tr><td>1</td><td>loc_resid_vag___1</td><td>Vagina</td></tr><tr><td>2</td><td>loc_resid_vag___2</td><td>Vulva</td></tr><tr><td>3</td><td>loc_resid_vag___3</td><td>Cervix</td></tr><tr><td>4</td><td>loc_resid_vag___4</td><td>Uterus</td></tr><tr><td>5</td><td>loc_resid_vag___5</td><td>Urethra (lower third)</td></tr><tr><td>6</td><td>loc_resid_vag___6</td><td>Urethra (upper two thirds)</td></tr><tr><td>7</td><td>loc_resid_vag___7</td><td>Bladder</td></tr><tr><td>8</td><td>loc_resid_vag___8</td><td>ureter(s)</td></tr><tr><td>9</td><td>loc_resid_vag___9</td><td>kidney(s)</td></tr><tr><td>10</td><td>loc_resid_vag___10</td><td>Anus</td></tr><tr><td>11</td><td>loc_resid_vag___11</td><td>Rectum</td></tr><tr><td>12</td><td>loc_resid_vag___12</td><td>Sigmoid</td></tr><tr><td>13</td><td>loc_resid_vag___13</td><td>Pelvic bone invasion</td></tr><tr><td>14</td><td>loc_resid_vag___14</td><td>Inguinal lymphadenopathy</td></tr><tr><td>15</td><td>loc_resid_vag___15</td><td>Pelvic lymphadenopathy</td></tr><tr><td>16</td><td>loc_resid_vag___16</td><td>Para-aortic-lymphadenopathy</td></tr><tr><td>17</td><td>loc_resid_vag___17</td><td>Other</td></tr></table></td></tr>
<tr><td>2028</td><td>[ps_loc_resid_vag]
Show the field ONLY if:
[loc_resid_vag(17)] = '1'</td><td>Please state</td><td>text</td></tr>
</table>

2029	[surgfind_vag]	Findings at surgery (Select all that apply)	checkbox<br>1 surgfind_vag___1 Vaginal tumour < 2cm<br>2 surgfind_vag___2 Vaginal tumour >2cm<br>3 surgfind_vag___3 Vulval invasion<br>4 surgfind_vag___4 Cervical invasion<br>5 surgfind_vag___5 Uterus invasion<br>6 surgfind_vag___6 Urethral invasion (lower third)<br>7 surgfind_vag___7 Urethral invasion (upper two thirds)<br>8 surgfind_vag___8 Bladder invasion<br>9 surgfind_vag___9 x1 hydroureter<br>10 surgfind_vag___10 x2 hydroureter<br>11 surgfind_vag___11 Ureteric invasion<br>12 surgfind_vag___12 x1 hydronephrosis<br>13 surgfind_vag___13 x2 hydronephrosis<br>14 surgfind_vag___14 Anal invasion<br>15 surgfind_vag___15 Rectal invasion<br>16 surgfind_vag___16 Sigmoid invasion<br>17 surgfind_vag___17 Pelvic bone invasion<br>18 surgfind_vag___18 Inguinal lymphadenopathy<br>19 surgfind_vag___19 Pelvic lymphadenopathy<br>20 surgfind_vag___20 Para-aortic-lymphadenopathy<br>21 surgfind_vag___21 Other<br>22 surgfind_vag___22 No visible disease
2030	[ps_surgfind_vag]<br>Show the field ONLY if: [surgfind_vag(21)] = '1'	Please state	text
2031	[tis_vag]	Tissue removed (Select all that apply)	checkbox<br>1 tis_vag___1 Vulva<br>2 tis_vag___2 Cervix<br>3 tis_vag___3 Uterus<br>4 tis_vag___4 x1 fallopian tube<br>5 tis_vag___5 x2 fallopian tubes<br>6 tis_vag___6 x1 ovary<br>7 tis_vag___7 x2 ovaries<br>8 tis_vag___8 Inguinal lymph nodes<br>9 tis_vag___9 Pelvic lymph nodes<br>10 tis_vag___10 Para-aortic lymph nodes<br>11 tis_vag___11 Anus<br>12 tis_vag___12 Rectum<br>13 tis_vag___13 Sigmoid<br>14 tis_vag___14 kidney<br>15 tis_vag___15 ureter<br>16 tis_vag___16 Bladder<br>17 tis_vag___17 Other
2032	[paln_dis_vag]<br>Show the field ONLY if: [tis_vag(10)] = '1'	Select all that apply	checkbox<br>1 paln_dis_vag___1 Infra-renal<br>2 paln_dis_vag___2 Supra-renal
2033	[ps_tis_vag]<br>Show the field ONLY if: [tis_vag(17)] = '1'	Please state	text

2034	[si_stom_vag]	Small bowel stoma	yesno<br>1 Yes<br>0 No
2035	[ss_vag]<br>Show the field ONLY if:<br>[si_stom_vag] = '1'	Stoma site	dropdown<br>1 Duodenum<br>2 Jejunum<br>3 Ileum
2036	[pb_si_stom_vag]<br>Show the field ONLY if:<br>[si_stom_vag] = '1'	Performed by (select all that apply)	checkbox<br>1 pb_si_stom_vag___1 Gynaecological oncologist<br>2 pb_si_stom_vag___2 Colorectal surgeon<br>3 pb_si_stom_vag___3 Urologist<br>4 pb_si_stom_vag___4 General surgeon<br>5 pb_si_stom_vag___5 Plastic surgeon<br>6 pb_si_stom_vag___6 Other
2037	[ps_pb_si_stom_vag]<br>Show the field ONLY if:<br>[pb_si_stom_vag(6)] = '1'	Please state	text
2038	[si_anast_vag]	Small bowel anastamosis	yesno<br>1 Yes<br>0 No
2039	[pb_si_anast_vag]<br>Show the field ONLY if:<br>[si_anast_vag] = '1'	Performed by (select all that apply)	checkbox<br>1 pb_si_anast_vag___1 Gynaecological oncologist<br>2 pb_si_anast_vag___2 Colorectal surgeon<br>3 pb_si_anast_vag___3 Urologist<br>4 pb_si_anast_vag___4 General surgeon<br>5 pb_si_anast_vag___5 Plastic surgeon<br>6 pb_si_anast_vag___6 Other
2040	[ps_pb_si_anast_vag]<br>Show the field ONLY if:<br>[pb_si_anast_vag(6)] = '1'	Please state	text
2041	[li_stom_vag]	Large bowel stoma	yesno<br>1 Yes<br>0 No
2042	[pb_li_stom_vag]<br>Show the field ONLY if:<br>[li_stom_vag] = '1'	Performed by (select all that apply)	checkbox<br>1 pb_li_stom_vag___1 Gynaecological oncologist<br>2 pb_li_stom_vag___2 Colorectal surgeon<br>3 pb_li_stom_vag___3 Urologist<br>4 pb_li_stom_vag___4 General surgeon<br>5 pb_li_stom_vag___5 Plastic surgeon<br>6 pb_li_stom_vag___6 Other
2043	[ps_pb_li_stom_vag]<br>Show the field ONLY if:<br>[pb_li_stom_vag(6)] = '1'	Please state	text
2044	[li_anast_vag]	Large bowel anastamosis	yesno<br>1 Yes<br>0 No
2045	[pb_li_anast_vag]<br>Show the field ONLY if:<br>[li_anast_vag] = '1'	Performed by (select all that apply)	checkbox<br>1 pb_li_anast_vag___1 Gynaecological oncologist<br>2 pb_li_anast_vag___2 Colorectal surgeon<br>3 pb_li_anast_vag___3 Urologist<br>4 pb_li_anast_vag___4 General surgeon<br>5 pb_li_anast_vag___5 Plastic surgeon<br>6 pb_li_anast_vag___6 Other

2046	[ps_pb_li_anast_vag]<br>Show the field ONLY if:<br>[pb_li_anast_vag(6)] = '1'	Please state	text
2047	[ic_vag]	Urostomy/ileal conduit	yesno<br>1 Yes<br>0 No
2048	[pb_ic_vag]<br>Show the field ONLY if:<br>[ic_vag] = '1'	Performed by (select all that apply)	checkbox<br>1 pb_ic_vag___1 Gynaecological oncologist<br>2 pb_ic_vag___2 Colorectal surgeon<br>3 pb_ic_vag___3 Urologist<br>4 pb_ic_vag___4 General surgeon<br>5 pb_ic_vag___5 Plastic surgeon<br>6 pb_ic_vag___6 Other
2049	[ps_pb_ic_vag]<br>Show the field ONLY if:<br>[pb_ic_vag(6)] = '1'	Please state	text
2050	[tuu_vag]	Trans uretero-ureteric anastamosis	yesno<br>1 Yes<br>0 No
2051	[pb_tuu_vag]<br>Show the field ONLY if:<br>[tuu_vag] = '1'	Performed by (select all that apply)	checkbox<br>1 pb_tuu_vag___1 Gynaecological oncologist<br>2 pb_tuu_vag___2 Colorectal surgeon<br>3 pb_tuu_vag___3 Urologist<br>4 pb_tuu_vag___4 General surgeon<br>5 pb_tuu_vag___5 Plastic surgeon<br>6 pb_tuu_vag___6 Other
2052	[ps_pb_tuu_vag]<br>Show the field ONLY if:<br>[pb_tuu_vag(6)] = '1'	Please state	text
2053	[neph_vag]	Nephrostomy	yesno<br>1 Yes<br>0 No
2054	[pb_neph_vag]<br>Show the field ONLY if:<br>[neph_vag] = '1'	Performed by (select all that apply)	checkbox<br>1 pb_neph_vag___1 Gynaecological oncologist<br>2 pb_neph_vag___2 Colorectal surgeon<br>3 pb_neph_vag___3 Urologist<br>4 pb_neph_vag___4 General surgeon<br>5 pb_neph_vag___5 Plastic surgeon<br>6 pb_neph_vag___6 Other
2055	[ps_pb_neph_vag]<br>Show the field ONLY if:<br>[pb_neph_vag(6)] = '1'	Please state	text
2056	[vag_recon]	Vaginal reconstruction	yesno<br>1 Yes<br>0 No
2057	[pb_vag_recon]<br>Show the field ONLY if:<br>[vag_recon] = '1'	Performed by (select all that apply)	checkbox<br>1 pb_vag_recon___1 Gynaecological oncologist<br>2 pb_vag_recon___2 Colorectal surgeon<br>3 pb_vag_recon___3 Urologist<br>4 pb_vag_recon___4 General surgeon<br>5 pb_vag_recon___5 Plastic surgeon<br>6 pb_vag_recon___6 Other

2058	[ps_pb_vag_recon]<br>Show the field ONLY if:<br>[pb_vag_recon(6)] = '1'	Please state	text
2059	[vulv_recon_vag]	Vulval reconstruction	yesno<br>1 Yes<br>0 No
2060	[vulvrecon_flap_vag]<br>Show the field ONLY if:<br>[vulv_recon_vag] = '1'	Type of vulval reconstruction	radio<br>1 Rotational flap<br>2 Non-rotational flap<br>3 Graft
2061	[surg_perf_vag]<br>Show the field ONLY if:<br>[vulv_recon_vag] = '1'	Performed by (Select all that apply)	checkbox<br>1 surg_perf_vag___1 Gynaecological oncologist<br>2 surg_perf_vag___2 Colorectal surgeon<br>3 surg_perf_vag___3 Urologist<br>4 surg_perf_vag___4 General surgeon<br>5 surg_perf_vag___5 Plastic surgeon<br>6 surg_perf_vag___6 Other
2062	[ps_surg_perf_vag]<br>Show the field ONLY if:<br>[surg_perf_vag(6)] = '1'	Please state	text
2063	[abx_vag]	Intraoperative antibiotics	yesno<br>1 Yes<br>0 No
2064	[tim_vag]	Length of surgery	text (time)
2065	[itu_vag]	Section Header: *POST-OPERATIVE*<br>Post-operative recovery in intensive care unit	yesno<br>1 Yes<br>0 No
2066	[itu_dys_vag]<br>Show the field ONLY if:<br>[itu_vag] = '1'	Number of days in intensive care unit	text (number)
2067	[hdu_vag]	Post-operative recovery in high dependency unit	yesno<br>1 Yes<br>0 No
2068	[hdu_dys_vag]<br>Show the field ONLY if:<br>[hdu_vag] = '1'	Number of days in high dependency unit	text (number)
2069	[er_vag]	Enhanced recovery?	yesno<br>1 Yes<br>0 No
2070	[po_abx_vag]	Prophylactic post operative antibiotics	yesno<br>1 Yes<br>0 No
2071	[drn_vag]	Post operative surgical drain	yesno<br>1 Yes<br>0 No
2072	[drn_dys_vag]<br>Show the field ONLY if:<br>[drn_vag] = '1'	Number of days in situ	text (number)
2073	[uc_vag]	Post operative urinary catheter	yesno<br>1 Yes<br>0 No
2074	[cath_dys_vag]<br>Show the field ONLY if:<br>[uc_vag] = '1'	Number of days in situ	text (number)

2075	[phb_vag]	Post-operative Haemoglobin (g/L) - please state first post-operative result	text (number)
2076	[pwbc_vag]	Post-operative White Cell Count (10^9/L) - please state first post-operative result	text (number)
2077	[pplat_vag]	Post-operative Platelets (10^9/L) - please state first post-operative result	text (number)
2078	[ppt_vag]	Post-operative PT (Secs) - please state first post-operative result	text (number)
2079	[pinr_vag]	Post-operative INR (Ratio) - please state first post-operative result	text (number)
2080	[paptt_vag]	Post-operative APTT (Secs) - please state first post-operative result	text (number)
2081	[pfib_vag]	Post-operative Fibrinogen (g/L) - please state first post-operative result	text (number)
2082	[palb_vag]	Post-operative Albumin (g/L) - please state first post-operative result	text (number)
2083	[pna_vag]	Post-operative Sodium (mmol/L) - please state first post-operative result	text (number)
2084	[pk_vag]	Post-operative Potassium (mmol/L) - please state first post-operative result	text (number)
2085	[purea_vag]	Post-operative Urea (mmol/L) - please state first post-operative result	text (number)
2086	[pcreat_vag]	Post-operative Creatinine (μmol/L) - please state first post-operative result	text
2087	[pegfr_vag]	Post-operative eGFR - Estimated Glomerular Filtration Rate (mL/min) - please state first post-operative result	text
2088	[pbili_vag]	Post-operative Bilirubin total (μmol/L) - please state first post-operative result	text (number)
2089	[paat_vag]	Post-operative Alanine Aminotransaminase (IU/L) - please state first post-operative result	text (number)
2090	[palp_vag]	Post-operative Alkaline Phosphatase (IU/L) - please state first post-operative result	text (number)
2091	[past_vag]	Post-operative Aspartate Aminotransaminase (IU/L) - please state first post-operative result	text (number)
2092	[pcrp_vag]	Post-operative CRP - C-reactive protein (mg/L) - please state first post-operative result	text (number)
2093	[histo_vag]	Histological type	dropdown<br>1 Squamous carcinoma<br>2 Adenocarcinoma<br>6 Small cell<br>3 Lymphoma<br>4 Sarcoma<br>5 Melanoma
2094	[r_vag]	Histological confirmed resection margins	dropdown<br>1 < 1 mm / tumour on margin<br>2 1-5 mm (NO tumour on margin)<br>3 >5 mm Margins confirmed clear<br>4 Unknown, not available in this hospital
2095	[p16_vag]	Section Header: *Immunohistochemistry*<br>p16	radio (Matrix)<br>1 Positive<br>2 Negative<br>3 Not tested
2096	[ki67_vag]	Ki67	radio (Matrix)<br>1 Positive<br>2 Negative<br>3 Not tested

2097	[p53_vag]	p53	radio (Matrix)<br>1 Positive<br>2 Negative<br>3 Not tested
2098	[cox2_vag]	COX-2	radio (Matrix)<br>1 Positive<br>2 Negative<br>3 Not tested
2099	[asma_vag]	α-SMA	radio (Matrix)<br>1 Positive<br>2 Negative<br>3 Not tested
2100	[cd34_vag]	CD34	radio (Matrix)<br>1 Positive<br>2 Negative<br>3 Not tested
2101	[hmga2_vag]	HMGA2	radio (Matrix)<br>1 Positive<br>2 Negative<br>3 Not tested
2102	[figo_vag_2]	Final surgical FIGO stage (i.e. postoperative staging taking into account surgical and pathology findings)	dropdown<br>1 1A Invasion confined to vagina, with tumour < 2cm wide and < 1mm depth of invasion<br>2 1B Invasion confined to vagina, with tumour >2cm wide and >1mm depth of invasion<br>3 Stage 2 Tumour invades para-vaginal tissues but not to the pelvic wall<br>4 Stage 3 Extension to the pelvic wall<br>5 Stage 4A Extension beyond true pelvis or invasion of bladder/rectum<br>6 Stage 4B Pelvic or inguinal lymphadenopathy or distant metastases
2103	[chemadj_vag]	Adjuvant (after surgery) chemotherapy	yesno<br>1 Yes<br>0 No
2104	[chemreg1_vag]<br>Show the field ONLY if:<br>[chemadj_vag] = '1'	Chemotherapy regimen FIRST line (Select all that apply)	checkbox<br>1 chemreg1_vag___1 Platinum (for example cisplatin, carboplatin)<br>2 chemreg1_vag___2 Fluorouracil<br>3 chemreg1_vag___3 Taxane (for example paclitaxel)<br>4 chemreg1_vag___4 Irenotecan<br>5 chemreg1_vag___5 Clinical trial regimens<br>6 chemreg1_vag___6 Other
2105	[ps_chemrej1_vag]<br>Show the field ONLY if:<br>[chemreg1_vag(6)] = '1'	Please state	text
2106	[chemadj_cycl_vag]<br>Show the field ONLY if:<br>[chemadj_vag] = '1'	Number of cycles planned	text (number)

2107	[chem_adj_no_vag]<br>Show the field ONLY if:<br>[chemadj_vag] = '0'	Reason adjuvant chemotherapy not administered (select all that apply)	checkbox<br>1 chem_adj_no_vag___1 Poor performance status<br>2 chem_adj_no_vag___2 No access<br>3 chem_adj_no_vag___3 Patient unable to afford<br>4 chem_adj_no_vag___4 Patient refused<br>5 chem_adj_no_vag___5 Not clinically indicated<br>6 chem_adj_no_vag___6 Other
2108	[ps_chem_adj_no_vag]<br>Show the field ONLY if:<br>[chem_adj_no_vag(6)] = '1'	Please state	text
2109	[rt_vag]	External beam radiotherapy administered?	yesno<br>1 Yes<br>0 No
2110	[rt_dos_vag]<br>Show the field ONLY if:<br>[rt_vag] = '1'	Dose received (Gray - Gy)	text (number)
2111	[rt_vag_no_reas]<br>Show the field ONLY if:<br>[rt_vag] = '0'	Reason external beam radiotherapy not administered (select all that apply)	checkbox<br>1 rt_vag_no_reas___1 Poor performance status<br>2 rt_vag_no_reas___2 No access<br>3 rt_vag_no_reas___3 Patient unable to afford<br>4 rt_vag_no_reas___4 Patient refused<br>5 rt_vag_no_reas___5 Not clinically indicated<br>6 rt_vag_no_reas___6 Other
2112	[ps_rt_vag_no_reas]<br>Show the field ONLY if:<br>[rt_vag_no_reas(6)] = '1'	Please state	text
2113	[brachy_vag]	Brachytherapy administered	yesno<br>1 Yes<br>0 No
2114	[rt_dos_vag_2]<br>Show the field ONLY if:<br>[brachy_vag] = '1'	Dose received (Gray - Gy)	text (number)
2115	[brachy_no_reas_vag]<br>Show the field ONLY if:<br>[brachy_vag] = '0'	Reason not administered (select all that apply)	checkbox<br>1 brachy_no_reas_vag___1 Poor performance status<br>2 brachy_no_reas_vag___2 No access<br>3 brachy_no_reas_vag___3 Patient unable to afford<br>4 brachy_no_reas_vag___4 Patient refused<br>5 brachy_no_reas_vag___5 Not clinically indicated<br>6 brachy_no_reas_vag___6 Other
2116	[ps_brachy_no_reas_vag]<br>Show the field ONLY if:<br>[brachy_no_reas_vag(6)] = '1'	Please state	text
2117	[vaginal_cancer_initial_presentation_complete]	Section Header: *Form Status*<br>Complete?	dropdown<br>0 Incomplete<br>1 Unverified<br>2 Complete
Instrument: **Vaginal cancer (recurrence)** (vaginal_cancer_recurrence)			
2118	[dat_dx_vag]	Section Header: *PREVIOUS TREATMENT RECEIVED AT PRIMARY DIAGNOSIS*<br>Date of initial/primary diagnosis	text (date_dmy)

2119	[prim_tx_vag]	Primary treatment following primary/initial diagnosis	checkbox<br>1 prim_tx_vag___1 Cytoreductive surgery<br>2 prim_tx_vag___2 Adjuvant chemotherapy<br>3 prim_tx_vag___3 Neoadjuvant chemotherapy<br>4 prim_tx_vag___4 External beam radiotherapy<br>5 prim_tx_vag___5 Brachytherapy<br>6 prim_tx_vag___6 Immunotherapy
2120	[dat_surg1_vag]<br>Show the field ONLY if:<br>[prim_tx_vag(1)] = '1'	Date of primary cytoreductive surgery	text (date_dmy)
2121	[dat_chem1_vag]<br>Show the field ONLY if:<br>[prim_tx_vag(2)] = '1'	Date of adjuvant chemotherapy following initial diagnosis	text (date_dmy)
2122	[dat_nact1_vag]<br>Show the field ONLY if:<br>[prim_tx_vag(3)] = '1'	Date of neoadjuvant chemotherapy following initial diagnosis	text (date_dmy)
2123	[dat_rt1_vag]<br>Show the field ONLY if:<br>[prim_tx_vag(4)] = '1'	Date of external beam radiotherapy following initial diagnosis	text (date_dmy)
2124	[dat_brachy1_vag]<br>Show the field ONLY if:<br>[prim_tx_vag(5)] = '1'	Date of brachytherapy following initial diagnosis	text (date_dmy)
2125	[dat_imm_vag_2]<br>Show the field ONLY if:<br>[prim_tx_vag(6)] = '1'	Date of immunotherapy following initial diagnosis	text (date_dmy)
2126	[dat_rec_vag]	Date of recurrence diagnosis	text (date_dmy)
2127	[age_vag_v2]	Section Header: *BASELINE DEMOGRAPHICS*<br>Age of patient	text (number)
2128	[eth_vag_v2]	Ethnicity	dropdown<br>1 White (British, Australian, North American, European, other white)<br>2 Asian (South East Asian, Far East Asian, other Asian)<br>3 Black (African, Caribbean, other Black)<br>4 Middle Eastern (Arab, North African, Turkish)<br>5 Mixed (multiple ethnic background)<br>6 Other
2129	[bmi_vag_v2]	Body mass index (kg/m2)	text (number)
2130	[ps_eth_vag_v2]<br>Show the field ONLY if:<br>[eth_vag_v2] = '6'	Please state	text
2131	[smok_vag_v2]	Smoker	dropdown<br>1 Yes<br>2 No<br>3 Ex-smoker
2132	[hpv_vag_v2]	HPV status	dropdown<br>1 Positive<br>2 Negative<br>3 Not tested
2133	[hpv_typ_vag2]<br>Show the field ONLY if:<br>[hpv_vag_v2] = '1'	HPV type (select all that apply)	checkbox<br>1 hpv_typ_vag2___1 16<br>2 hpv_typ_vag2___2 18<br>3 hpv_typ_vag2___3 other<br>4 hpv_typ_vag2___4 Unknown

2134	[hpv_vag_no_reas_v2]<br>Show the field ONLY if:<br>[hpv_vag_v2] = '3'	Reason not done (select all that apply)	checkbox<br>1 hpv_vag_no_reas_v2___1 Not clinical protocol<br>2 hpv_vag_no_reas_v2___2 Patient refused<br>3 hpv_vag_no_reas_v2___3 Patient unable to afford test<br>4 hpv_vag_no_reas_v2___4 Testing not available locally<br>5 hpv_vag_no_reas_v2___5 Other
2135	[ps_hpv_vag_no_reas_v2]<br>Show the field ONLY if:<br>[hpv_vag_no_reas_v2(5)] = '1'	Please state	text
2136	[vac_vag_v2]	HPV vaccine administered historically	yesno<br>1 Yes<br>0 No
2137	[typ_vac_vag_v2]<br>Show the field ONLY if:<br>[vac_vag_v2] = '1'	Type of vaccine administered	radio<br>1 Bivalent<br>2 Quadrivalent<br>3 9-valent
2138	[age_vac_vag_v2]<br>Show the field ONLY if:<br>[vac_vag_v2] = '1'	Age vaccine administered	text (number)
2139	[vac_no_reas_vag_v2]<br>Show the field ONLY if:<br>[vac_vag_v2] = '0'	Reason vaccine not administered (select all that apply)	checkbox<br>1 vac_no_reas_vag_v2___1 No national vaccination programme<br>2 vac_no_reas_vag_v2___2 National vaccination programme introduced after age of first sexual activity<br>3 vac_no_reas_vag_v2___3 Patient unable to afford<br>4 vac_no_reas_vag_v2___4 Other
2140	[ps_vac_no_reas_vag_v2]<br>Show the field ONLY if:<br>[vac_no_reas_vag_v2(4)] = '1'	Please state	text

2141	[asa_vag_v2]	Section Header: *PRE-OPERATIVE*<br>American Society of Anesthesiologists (ASA) physical status	dropdown<br>1 ASA 1: A normal healthy patient. Example: Fit, non-obese (BMI under 30), a nonsmoking patient with good exercise tolerance.<br>2 ASA 2: A patient with a mild systemic disease. Example: Patient with no functional limitations and a well-controlled disease (e.g., treated hypertension, obesity with BMI under 35, frequent social drinker or is a cigarette smoker).<br>3 ASA 3: A patient with a severe systemic disease that is not life-threatening. Example: Patient with some functional limitation as a result of disease (e.g., poorly treated hypertension or diabetes, morbid obesity, chronic renal failure, a bronchospastic disease with intermittent exacerbation, stable angina, implanted pacemaker).<br>4 ASA 4: A patient with a severe systemic disease that is a constant threat to life. Example: Patient with functional limitation from severe, life-threatening disease (e.g., unstable angina, poorly controlled COPD, symptomatic CHF, recent (less than three months ago) myocardial infarction or stroke.<br>5 ASA 5: A moribund patient who is not expected to survive without the operation. The patient is not expected to survive beyond the next 24 hours without surgery. Examples: ruptured abdominal aortic aneurysm, massive trauma, and extensive intracranial hemorrhage with mass effect.<br>6 ASA 6: A brain-dead patient whose organs are being removed with the intention of transplanting them into another patient.
2142	[ecog_vag_v2]	ECOG (Eastern Cooperative Oncology Group) performance status	dropdown<br>1 Grade 0 - Fully active, able to carry on all pre-disease performance without restriction<br>2 Grade 1 - Restricted in physically strenuous activity but ambulatory and able to carry out work of a light or sedentary nature, e.g., light house work, office work<br>3 Grade 2 - Ambulatory and capable of all selfcare but unable to carry out any work activities; up and about more than 50% of waking hours<br>4 Grade 3 - Capable of only limited selfcare; confined to bed or chair more than 50% of waking hours<br>5 Grade 4 - Completely disabled; cannot carry on any selfcare; totally confined to bed or chair<br>6 Grade 5 - Dead
2143	[comorb_vag2]	Co-morbidities	checkbox<br>1 comorb_vag2___1 Cardiovascular<br>2 comorb_vag2___2 Respiratory<br>3 comorb_vag2___3 Urogenital<br>4 comorb_vag2___4 Endocrine<br>5 comorb_vag2___5 Neurological<br>6 comorb_vag2___6 Haematological<br>7 comorb_vag2___7 Psychiatric<br>8 comorb_vag2___8 Gastroenterology<br>9 comorb_vag2___9 Rheumatology and musculoskeletal<br>10 comorb_vag2___10 Oncology<br>11 comorb_vag2___11 None

2144	[comorb_cvd_vag2]<br>Show the field ONLY if:<br>[comorb_vag2(1)] = '1'	Select all that apply	checkbox<br>1 comorb_cvd_vag2___1 Hypertension<br>2 comorb_cvd_vag2___2 Coronary heart disease / ischaemic heart disease<br>3 comorb_cvd_vag2___3 Myocardial infarction<br>4 comorb_cvd_vag2___4 Heart failure<br>5 comorb_cvd_vag2___5 Arrhythmia<br>6 comorb_cvd_vag2___6 Heart valve disease<br>7 comorb_cvd_vag2___7 Venous insufficiency<br>8 comorb_cvd_vag2___8 Peripheral vascular disease<br>9 comorb_cvd_vag2___9 Stroke<br>10 comorb_cvd_vag2___10 Transient ischaemic attack<br>11 comorb_cvd_vag2___11 Other
2145	[comorb_resp_vag2]<br>Show the field ONLY if:<br>[comorb_vag2(2)] = '1'	Select all that apply	checkbox<br>1 comorb_resp_vag2___1 Asthma<br>2 comorb_resp_vag2___2 Emphysema (COPD)<br>3 comorb_resp_vag2___3 Chronic bronchitis (COPD)<br>4 comorb_resp_vag2___4 Pulmonary fibrosis<br>5 comorb_resp_vag2___5 Sleep apnoea<br>6 comorb_resp_vag2___6 Bronchiectasis<br>7 comorb_resp_vag2___7 Cystic fibrosis<br>8 comorb_resp_vag2___8 Tuberculosis<br>9 comorb_resp_vag2___9 Pneumonia<br>10 comorb_resp_vag2___10 Other
2146	[comorb_uro_vag2]<br>Show the field ONLY if:<br>[comorb_vag2(3)] = '1'	Select all that apply	checkbox<br>1 comorb_uro_vag2___1 Chronic kidney disease (not requiring haemodialysis)<br>2 comorb_uro_vag2___2 Chronic kidney disease (requiring haemodialysis)<br>3 comorb_uro_vag2___3 Polycystic kidney disease<br>4 comorb_uro_vag2___4 Glomerulonephropathy<br>5 comorb_uro_vag2___5 Glomerulonephritis<br>6 comorb_uro_vag2___6 Urinary tract infection<br>7 comorb_uro_vag2___7 Other
2147	[comorb_endo_vag2]<br>Show the field ONLY if:<br>[comorb_vag2(4)] = '1'	Select all that apply	checkbox<br>1 comorb_endo_vag2___1 Type 1 diabetes mellitus<br>2 comorb_endo_vag2___2 Type 2 diabetes mellitus<br>3 comorb_endo_vag2___3 Hyperthyroidism<br>4 comorb_endo_vag2___4 Hypothyroidism<br>5 comorb_endo_vag2___5 Other
2148	[comorb_neuro_vag2]<br>Show the field ONLY if:<br>[comorb_vag2(5)] = '1'	Select all that apply	checkbox<br>1 comorb_neuro_vag2___1 Parkinsons disease<br>2 comorb_neuro_vag2___2 Myasthenia gravis<br>3 comorb_neuro_vag2___3 Dementia<br>4 comorb_neuro_vag2___4 Epilepsy<br>5 comorb_neuro_vag2___5 Other

2149	[comorb_haem_vag2]<br><br>Show the field ONLY if:<br>[comorb_vag2(6)] = '1'	Select all that apply	checkbox<br>1 comorb_haem_vag2___1 Iron deficiency anaemia<br>2 comorb_haem_vag2___2 Folate deficience anaemia<br>3 comorb_haem_vag2___3 Vitamin B12 deficiency anaemia<br>4 comorb_haem_vag2___4 Pernicious anaemia<br>5 comorb_haem_vag2___5 Haemolytic anaemia<br>6 comorb_haem_vag2___6 Aplastic anaemia<br>7 comorb_haem_vag2___7 Thalassemia<br>8 comorb_haem_vag2___8 Sickle cell disease<br>9 comorb_haem_vag2___9 Disseminated intravascular coagulation (DIC)<br>10 comorb_haem_vag2___10 Factor V Leiden<br>11 comorb_haem_vag2___11 Thrombocytosis<br>12 comorb_haem_vag2___12 Idiopathic thrombocytopenic purpura (ITP)<br>13 comorb_haem_vag2___13 Haemophilia<br>14 comorb_haem_vag2___14 Von Willebrand disease<br>15 comorb_haem_vag2___15 Antiphospholipid syndrome<br>16 comorb_haem_vag2___16 Thrombocytopenia<br>17 comorb_haem_vag2___17 Thrombotic thrombocytopenic purpura<br>18 comorb_haem_vag2___18 Deep vein thrombosis<br>19 comorb_haem_vag2___19 Pulmonary embolism<br>20 comorb_haem_vag2___20 Other
2150	[comorb_pscy_vag2]<br><br>Show the field ONLY if:<br>[comorb_vag2(7)] = '1'	Select all that apply	checkbox<br>1 comorb_pscy_vag2___1 Anxiety<br>2 comorb_pscy_vag2___2 Depression<br>3 comorb_pscy_vag2___3 Learning difficulties<br>4 comorb_pscy_vag2___4 Personality disorder<br>5 comorb_pscy_vag2___5 Schizophrenia<br>6 comorb_pscy_vag2___6 Bipolar disorder<br>7 comorb_pscy_vag2___7 Autism<br>8 comorb_pscy_vag2___8 Other

2151	[comorb_gi_vag_2]<br>Show the field ONLY if:<br>[comorb_vag2(8)] = '1'	Select all that apply	checkbox<br>1 comorb_gi_vag_2___1 Crohn's disease<br>2 comorb_gi_vag_2___2 Ulcerative colitis<br>3 comorb_gi_vag_2___3 Irritable bowel syndrome<br>4 comorb_gi_vag_2___4 Celiac Disease<br>5 comorb_gi_vag_2___5 Peptic ulcer disease<br>6 comorb_gi_vag_2___6 Gallstones<br>7 comorb_gi_vag_2___7 Pancreatitis<br>8 comorb_gi_vag_2___8 Diverticulitis<br>9 comorb_gi_vag_2___9 Non-Alcoholic Fatty Liver Disease<br>10 comorb_gi_vag_2___10 Alcoholic fatty liver disease<br>11 comorb_gi_vag_2___11 Viral hepatitis<br>12 comorb_gi_vag_2___12 Liver failure<br>13 comorb_gi_vag_2___13 Other
2152	[comorb_msk_vag_2]<br>Show the field ONLY if:<br>[comorb_vag2(9)] = '1'	Select all that apply	checkbox<br>1 comorb_msk_vag_2___1 Osteoarthritis<br>2 comorb_msk_vag_2___2 Rheumatoid arthritis<br>3 comorb_msk_vag_2___3 Osteoporosis<br>4 comorb_msk_vag_2___4 Lupus<br>5 comorb_msk_vag_2___5 Spondyloarthropathies<br>6 comorb_msk_vag_2___6 Sjogren's syndrome<br>7 comorb_msk_vag_2___7 Gout<br>8 comorb_msk_vag_2___8 Other
2153	[comorb_onc_vag_2]<br>Show the field ONLY if:<br>[comorb_vag2(10)] = '1'	Select all that apply	checkbox<br>1 comorb_onc_vag_2___1 Breast cancer<br>2 comorb_onc_vag_2___2 Lung cancer<br>3 comorb_onc_vag_2___3 Hepatobiliary cancer<br>4 comorb_onc_vag_2___4 Brain cancer<br>5 comorb_onc_vag_2___5 Leukemia<br>6 comorb_onc_vag_2___6 Lymphoma<br>7 comorb_onc_vag_2___7 Osteosarcoma<br>8 comorb_onc_vag_2___8 Other
2154	[lap_vag2]	Previous laparoscopic abdominal surgery	yesno<br>1 Yes<br>0 No
2155	[opn_vag2]	Previous laparotomy	yesno<br>1 Yes<br>0 No
2156	[mdt_vag_v2]	Discussed at multidisciplinary team meeting/tumour board?	yesno<br>1 Yes<br>0 No

2157	[mdt_tm_vag2]<br>Show the field ONLY if:<br>[mdt_vag_v2] = '1'	Members of multidisciplinary team/tumour board (select all that apply)	checkbox<br>1 mdt_tm_vag2___1 Certified gynaecological oncology surgeon<br>2 mdt_tm_vag2___2 Trained surgeon dedicated to the management of gynaecologic cancer or having completed an ESGO-accredited fellowship<br>3 mdt_tm_vag2___3 Radiologist<br>4 mdt_tm_vag2___4 Radiation/clinical oncologist (administering radiotherapy)<br>5 mdt_tm_vag2___5 Medical oncologist (administering chemotherapy)<br>6 mdt_tm_vag2___6 Pathologist
2158	[mdt_vagc_no_v2]<br>Show the field ONLY if:<br>[mdt_vag_v2] = '0'	Reason not discussed (select all that apply)	checkbox<br>1 mdt_vagc_no_v2___1 No multidisciplinary team meeting/tumour board<br>2 mdt_vagc_no_v2___2 Other
2159	[ps_mdt_vagc_no_v2]<br>Show the field ONLY if:<br>[mdt_vagc_no_v2(2)] = '1'	Please state	text
2160	[figo_vag_v2]	FIGO Stage	dropdown<br>1 1A Invasion confined to vagina, with tumour < 2cm wide and < 1mm depth of invasion<br>2 1B Invasion confined to vagina, with tumour >2cm wide and >1mm depth of invasion<br>3 Stage 2 Tumour invades para-vaginal tissues but not to the pelvic wall<br>4 Stage 3 Extension to the pelvic wall<br>5 Stage 4A Extension beyond true pelvis or invasion of bladder/rectum<br>6 Stage 4B Pelvic or inguinal lymphadenopathy or distant metastases
2161	[bx_vag_v2]	Diagnostic biopsy performed at recurrence diagnosis?	yesno<br>1 Yes<br>0 No
2162	[bx_vag_no_reas_v2]<br>Show the field ONLY if:<br>[bx_vag_v2] = '0'	Reason diagnostic biopsy not performed at recurrence diagnosis (Select all that apply)	checkbox<br>1 bx_vag_no_reas_v2___1 Biopsy not technically feasible<br>2 bx_vag_no_reas_v2___2 Patient refused<br>3 bx_vag_no_reas_v2___3 Unable to perform<br>4 bx_vag_no_reas_v2___4 No resources available locally to perform<br>5 bx_vag_no_reas_v2___5 Other
2163	[ps_bx_vag_no_reas_v2]<br>Show the field ONLY if:<br>[bx_vag_no_reas_v2(5)] = '1'	Please state	text
2164	[mri_vag_v2]	Magnetic resonance imaging (MRI) pelvis/abdominal-pelvis performed at recurrence<br>diagnosis?	yesno<br>1 Yes<br>0 No

2165	[mri_find_vag_v2]<br>Show the field ONLY if:<br>[mri_vag_v2] = '1'	MRI pelvis/abdominal-pelvis findings at recurrence diagnosis (select all that apply)	checkbox<br>1 mri_find_vag_v2___1 Vaginal tumour < 2cm<br>2 mri_find_vag_v2___2 Vaginal tumour >2cm<br>3 mri_find_vag_v2___3 Vulval invasion<br>4 mri_find_vag_v2___4 Cervical invasion<br>5 mri_find_vag_v2___5 Uterus invasion<br>6 mri_find_vag_v2___6 Urethral invasion (lower third)<br>7 mri_find_vag_v2___7 Urethral invasion (upper two thirds)<br>8 mri_find_vag_v2___8 x1 hydroureter<br>9 mri_find_vag_v2___9 x2 hydroureter<br>10 mri_find_vag_v2___10 Ureteric invasion<br>11 mri_find_vag_v2___11 x1 hydronephrosis<br>12 mri_find_vag_v2___12 x2 hydronephrosis<br>13 mri_find_vag_v2___13 Bladder invasion<br>14 mri_find_vag_v2___14 Anal invasion<br>15 mri_find_vag_v2___15 Rectal invasion<br>16 mri_find_vag_v2___16 Sigmoid invasion<br>17 mri_find_vag_v2___17 Pelvic bone invasion<br>18 mri_find_vag_v2___18 Inguinal lymphadenopathy<br>19 mri_find_vag_v2___19 Pelvic lymphadenopathy<br>20 mri_find_vag_v2___20 Para-aortic lymphadenopathy<br>21 mri_find_vag_v2___21 Other
2166	[ps_mri_find_vag_v2]<br>Show the field ONLY if:<br>[mri_find_vag_v2(21)] = '1'	Please state	text
2167	[mri_vag_no_reas_v2]<br>Show the field ONLY if:<br>[mri_vag_v2] = '0'	Reason MRI not performed at recurrence diagnosis (select all that apply)	checkbox<br>1 mri_vag_no_reas_v2___1 No access to MRI machine<br>2 mri_vag_no_reas_v2___2 Patient unable to afford<br>3 mri_vag_no_reas_v2___3 Patient refused<br>4 mri_vag_no_reas_v2___4 Claustrophobia<br>5 mri_vag_no_reas_v2___5 Other imaging performed<br>6 mri_vag_no_reas_v2___6 Extreme morbid obesity<br>7 mri_vag_no_reas_v2___7 Other
2168	[ps_mri_vag_no_reas_v2]<br>Show the field ONLY if:<br>[mri_vag_no_reas_v2(7)] = '1'	Please state	text
2169	[ct_vag_v2]	Staging computerised tomography (CT) performed at recurrence diagnosis?	yesno<br>1 Yes<br>0 No

2170	[ct_vag_find_v2]<br>Show the field ONLY if:<br>[ct_vag_v2] = '1'	Staging CT findings at recurrence diagnosis (Select all that apply)	checkbox<br>1 ct_vag_find_v2___1 Vaginal tumour < 2cm<br>2 ct_vag_find_v2___2 Vaginal tumour >2cm<br>3 ct_vag_find_v2___3 Vulval invasion<br>4 ct_vag_find_v2___4 Cervical invasion<br>5 ct_vag_find_v2___5 Uterus invasion<br>6 ct_vag_find_v2___6 Urethral invasion (lower third)<br>7 ct_vag_find_v2___7 Urethral invasion (upper two thirds)<br>8 ct_vag_find_v2___8 x1 hydroureter<br>9 ct_vag_find_v2___9 x2 hydroureter<br>10 ct_vag_find_v2___10 Ureteric invasion<br>11 ct_vag_find_v2___11 x1 hydronephrosis<br>12 ct_vag_find_v2___12 x2 hydronephrosis<br>13 ct_vag_find_v2___13 Bladder invasion<br>14 ct_vag_find_v2___14 Anal invasion<br>15 ct_vag_find_v2___15 Rectal invasion<br>16 ct_vag_find_v2___16 Sigmoid invasion<br>17 ct_vag_find_v2___17 Pelvic bone invasion<br>18 ct_vag_find_v2___18 Inguinal lymphadenopathy<br>19 ct_vag_find_v2___19 Pelvic lymphadenopathy<br>20 ct_vag_find_v2___20 Para-aortic lymphadenopathy<br>21 ct_vag_find_v2___21 Lung nodules<br>22 ct_vag_find_v2___22 Pleural effusion<br>23 ct_vag_find_v2___23 Other
2171	[ps_ct_vag_find_v2]<br>Show the field ONLY if:<br>[ct_vag_find_v2(23)] = '1'	Please state	text
2172	[ct_vag_no_reas_v2]<br>Show the field ONLY if:<br>[ct_vag_v2] = '0'	Reason staging CT not performed at recurrence diagnosis (Select all that apply)	checkbox<br>1 ct_vag_no_reas_v2___1 No access to CT machine<br>2 ct_vag_no_reas_v2___2 Patient unable to afford<br>4 ct_vag_no_reas_v2___4 Patient refused<br>5 ct_vag_no_reas_v2___5 Other imaging performed<br>6 ct_vag_no_reas_v2___6 Allergic reaction to contrast<br>7 ct_vag_no_reas_v2___7 Chronic kidney disease<br>3 ct_vag_no_reas_v2___3 Other
2173	[ps_ct_vag_no_reas_v2]<br>Show the field ONLY if:<br>[ct_vag_no_reas_v2(3)] = '1'	Please state	text
2174	[pet_vag_v2]	PET-CT performed at recurrence diagnosis?	yesno<br>1 Yes<br>0 No

2175	[pet_vag_find_v2]<br>Show the field ONLY if:<br>[pet_vag_v2] = '1'	PET-CT findings at recurrence diagnosis (Select all that apply)	checkbox<br>1 pet_vag_find_v2___1 FDG-avid uptake vagina<br>2 pet_vag_find_v2___2 FDG-avid uptake vulva<br>3 pet_vag_find_v2___3 FDG-avid uptake cervix<br>4 pet_vag_find_v2___4 FDG-avid uptake uterus<br>5 pet_vag_find_v2___5 FDG-avid uptake urethra<br>6 pet_vag_find_v2___6 FDG-avid uptake ureter(s)<br>7 pet_vag_find_v2___7 FDG-avid uptake kidney(s)<br>8 pet_vag_find_v2___8 FDG-avid uptake bladder<br>9 pet_vag_find_v2___9 FDG-avid uptake anus<br>10 pet_vag_find_v2___10 FDG-avid uptake rectum<br>11 pet_vag_find_v2___11 FDG-avid uptake sigmoid<br>12 pet_vag_find_v2___12 FDG-avid uptake pelvic bones<br>13 pet_vag_find_v2___13 FDG-avid uptake inguinal lymph nodes<br>14 pet_vag_find_v2___14 FDG-avid uptake pelvic lymph nodes<br>15 pet_vag_find_v2___15 FDG-avid uptake para-aortic lymph nodes<br>16 pet_vag_find_v2___16 Other
2176	[ps_pet_vag_find_v2]<br>Show the field ONLY if:<br>[pet_vag_find_v2(16)] = '1'	Please state	text
2177	[pet_vag_no_reas_v2]<br>Show the field ONLY if:<br>[pet_vag_v2] = '0'	Reason PET-CT not performed at recurrence diagnosis (select all that apply)	checkbox<br>1 pet_vag_no_reas_v2___1 No access to PET machine<br>2 pet_vag_no_reas_v2___2 Patient unable to afford<br>3 pet_vag_no_reas_v2___3 Patient refused<br>4 pet_vag_no_reas_v2___4 Uncontrolled diabetes mellitus<br>5 pet_vag_no_reas_v2___5 Other imaging performed<br>6 pet_vag_no_reas_v2___6 Not clinical protocol<br>7 pet_vag_no_reas_v2___7 Other
2178	[ps_pet_vag_no_reas_v2]<br>Show the field ONLY if:<br>[pet_vag_no_reas_v2(7)] = '1'	Please state	text
2179	[cov19_vag_v2]	COVID-19 status	dropdown<br>1 Positive<br>2 Negative<br>3 Not tested

2180	[cov19_vag_no_reas_v2]<br>Show the field ONLY if:<br>[cov19_vag_v2] = '3'	Reason not tested (select all that apply)	checkbox<br>1 cov19_vag_no_reas_v2___1 Not clinically indicated (asymptomatic)<br>2 cov19_vag_no_reas_v2___2 Not clinical protocol to routinely test all patients prior to surgery<br>3 cov19_vag_no_reas_v2___3 Patient refused<br>4 cov19_vag_no_reas_v2___4 Patient unable to afford test<br>5 cov19_vag_no_reas_v2___5 No resources locally for testing<br>6 cov19_vag_no_reas_v2___6 Other
2181	[ps_cov19_vag_no_reas_v2]<br>Show the field ONLY if:<br>[cov19_vag_no_reas_v2(6)] = '1'	Please state	text
2182	[bp_vag2]	Bowel preparation pre-operatively	yesno<br>1 Yes<br>0 No
2183	[peri_op_vag2]	Peri-operative management plan in place? (Non- exhaustive list: pre-operative haemoglobin optimisation and iron deficit correction; correction of denutrition and immunonutrition; fluid management; pain management; prevention of post-operative nausea and vomiting).	yesno<br>1 Yes<br>0 No
2184	[hb_vag2]	Pre-operative Haemoglobin (g/L)	text (number)
2185	[wcc_vag2]	Pre-operative White Cell Count (10^9/L)	text (number)
2186	[plt_vag2]	Pre-operative Platelets (10^9/L)	text (number)
2187	[pt_vag2]	Pre-operative PT (Secs)	text (number)
2188	[inr_vag2]	Pre-operative INR (Ratio)	text (number)
2189	[aptt_vag2]	Pre-operative APTT (Secs)	text (number)
2190	[fib_vag2]	Pre-operative Fibrinogen (g/L)	text (number)
2191	[alb_vag2]	Pre-operative Albumin (g/L)	text (number)
2192	[na_vag2]	Pre-operative Sodium (mmol/L)	text (number)
2193	[k_vag2]	Pre-operative Potassium (mmol/L)	text (number)
2194	[ur_vag2]	Pre-operative Urea (mmol/L)	text (number)
2195	[creat_vag2]	Pre-operative Creatinine (µmol/L)	text (number)
2196	[egfr_vag2]	Pre-operative eGFR - estimated glomerular filtration rate (mL/min)	text
2197	[bili_vag2]	Pre-operative Bilirubin total (µmol/L)	text (number)
2198	[aat_vag2]	Pre-operative Alanine Aminotransaminase (IU/L)	text (number)
2199	[alp_vag2]	Pre-operative Alkaline Phosphatase (IU/L)	text (number)
2200	[ast_vag2]	Pre-operative Aspartate Aminotransaminase (IU/L)	text (number)
2201	[crp_vag2]	Pre-operative CRP - C-reactive protein (mg/L)	text (number)
2202	[datsurg_vag_v2]	Section Header: *SURGERY*<br>Date of recurrence surgery	text (date_dmy)
2203	[anaes_vag_v2]	Anaesthesia (Select all that apply)	checkbox<br>1 anaes_vag_v2___1 General<br>2 anaes_vag_v2___2 Spinal<br>3 anaes_vag_v2___3 Epidural
2204	[elecem_vag_v2]	Was recurrence surgery an elective or emergency case?	radio<br>1 Elective<br>2 Emergency

2205	[sa_vag_v2]	Surgical aim	dropdown<br>1 Curative<br>2 Curative attempted but then abandoned (i.e. open/close laparotomy)<br>3 Palliative
2206	[sm_vag_v2]	Surgical modality (select all that apply)	checkbox<br>1 sm_vag_v2___1 Vaginal<br>2 sm_vag_v2___2 Laparotomy
2207	[ls_vag_v2]	Lead surgeon performing recurrence surgery	dropdown<br>1 Certified consultant/specialist gynaecological oncologist<br>2 Fellow/trainee gynaecological oncologist<br>3 Trained surgeon dedicated to the management of gynaecologic cancer or having completed an ESGO accredited fellowship.<br>4 General surgeon<br>5 Surgical oncologist<br>6 Other
2208	[ps_ls_vag_v2]<br>Show the field ONLY if:<br>[ls_vag_v2] = '6'	Please state	text
2209	[who_vag2]	WHO surgical safety checklist used	yesno<br>1 Yes<br>0 No
2210	[cyto_vag2]	Complete macroscopic cytoreduction at recurrence surgery	yesno<br>1 Yes<br>0 No
2211	[reas_cytored_no_vag2]<br>Show the field ONLY if:<br>[cyto_vag2] = '0'	Reason(s) for incomplete excision of disease at cytoreduction recurrence surgery (select all that apply)	checkbox<br>1 reas_cytored_no_vag2___1 Disease more extensive than that shown on imaging<br>2 reas_cytored_no_vag2___2 Technically unable to resect<br>3 reas_cytored_no_vag2___3 Patient became medically unstable intra-operatively and decision made to not proceed further with surgery<br>4 reas_cytored_no_vag2___4 Other
2212	[ps_reas_cytored_no_vag2]<br>Show the field ONLY if:<br>[reas_cytored_no_vag2(4)] = '1'	Please state	text
2213	[siz_resid_vag2]<br>Show the field ONLY if:<br>[cyto_vag2] = '0'	Size of residual disease (in millimetres)	dropdown<br>1 1-2.5<br>2 2.6-5<br>3 5.1-7.5<br>4 7.6-10<br>5 >10

2214	[loc_resid_vag2]<br>Show the field ONLY if:<br>[cyto_vag2] = '0'	Location of residual disease at recurrence surgery (select all that apply)	checkbox<br>1 loc_resid_vag2___1 Vagina<br>2 loc_resid_vag2___2 Vulva<br>3 loc_resid_vag2___3 Cervix<br>4 loc_resid_vag2___4 Uterus<br>5 loc_resid_vag2___5 Urethra (lower third)<br>6 loc_resid_vag2___6 Urethra (upper two thirds)<br>7 loc_resid_vag2___7 Bladder<br>8 loc_resid_vag2___8 ureter(s)<br>9 loc_resid_vag2___9 kidney(s)<br>10 loc_resid_vag2___10 Anus<br>11 loc_resid_vag2___11 Rectum<br>12 loc_resid_vag2___12 Sigmoid<br>13 loc_resid_vag2___13 Pelvic bone invasion<br>14 loc_resid_vag2___14 Inguinal lymphadenopathy<br>15 loc_resid_vag2___15 Pelvic lymphadenopathy<br>16 loc_resid_vag2___16 Para-aortic-lymphadenopathy<br>17 loc_resid_vag2___17 Other
2215	[ps_loc_resid_vag2]<br>Show the field ONLY if:<br>[loc_resid_vag2(17)] = '1'	Please state	text
2216	[surgfind_vag_v2]	Findings at recurrence surgery (Select all that apply)	checkbox<br>1 surgfind_vag_v2___1 Vaginal tumour < 2cm<br>2 surgfind_vag_v2___2 Vaginal tumour >2cm<br>3 surgfind_vag_v2___3 Vulval invasion<br>4 surgfind_vag_v2___4 Cervical invasion<br>5 surgfind_vag_v2___5 Uterus invasion<br>6 surgfind_vag_v2___6 Urethral invasion (lower third)<br>7 surgfind_vag_v2___7 Urethral invasion (upper two thirds)<br>8 surgfind_vag_v2___8 Bladder invasion<br>9 surgfind_vag_v2___9 x1 hydroureter<br>10 surgfind_vag_v2___10 x2 hydroureter<br>11 surgfind_vag_v2___11 Ureteric invasion<br>12 surgfind_vag_v2___12 x1 hydronephrosis<br>13 surgfind_vag_v2___13 x2 hydronephrosis<br>14 surgfind_vag_v2___14 Anal invasion<br>15 surgfind_vag_v2___15 Rectal invasion<br>16 surgfind_vag_v2___16 Sigmoid invasion<br>17 surgfind_vag_v2___17 Pelvic bone invasion<br>18 surgfind_vag_v2___18 Inguinal lymphadenopathy<br>19 surgfind_vag_v2___19 Pelvic lymphadenopathy<br>20 surgfind_vag_v2___20 Para-aortic-lymphadenopathy<br>21 surgfind_vag_v2___21 Other<br>22 surgfind_vag_v2___22 No visible disease
2217	[ps_surgfind_vag_v2]<br>Show the field ONLY if:<br>[surgfind_vag_v2(21)] = '1'	Please state	text

2218	[tis_vag_v2]	Tissue removed (Select all that apply)	checkbox<br>1 tis_vag_v2___1 Vagina<br>2 tis_vag_v2___2 Vulva<br>3 tis_vag_v2___3 Cervix<br>4 tis_vag_v2___4 Uterus<br>5 tis_vag_v2___5 x1 fallopian tube<br>6 tis_vag_v2___6 x2 fallopian tubes<br>7 tis_vag_v2___7 x1 ovary<br>8 tis_vag_v2___8 x2 ovaries<br>9 tis_vag_v2___9 Inguinal lymph nodes<br>10 tis_vag_v2___10 Pelvic lymph nodes<br>11 tis_vag_v2___11 Para-aortic lymph nodes<br>12 tis_vag_v2___12 Anus<br>13 tis_vag_v2___13 Rectum<br>14 tis_vag_v2___14 Sigmoid<br>15 tis_vag_v2___15 kidney<br>16 tis_vag_v2___16 ureter<br>17 tis_vag_v2___17 Bladder<br>18 tis_vag_v2___18 Other
2219	[paln_dis_vag2]<br>Show the field ONLY if:<br>[tis_vag_v2(11)] = '1'	Select all that apply	checkbox<br>1 paln_dis_vag2___1 Infra-renal<br>2 paln_dis_vag2___2 Supra-renal
2220	[ps_tis_vag_v2]<br>Show the field ONLY if:<br>[tis_vag_v2(18)] = '1'	Please state	text
2221	[si_stom_vag_v2]	Small bowel stoma	yesno<br>1 Yes<br>0 No
2222	[ss_vag2]<br>Show the field ONLY if:<br>[si_stom_vag_v2] = '1'	Stoma site	dropdown<br>1 Duodenum<br>2 Jejunum<br>3 Ileum
2223	[pb_si_stom_vag_v2]<br>Show the field ONLY if:<br>[si_stom_vag_v2] = '1'	Performed by (select all that apply)	checkbox<br>1 pb_si_stom_vag_v2___1 Gynaecological oncologist<br>2 pb_si_stom_vag_v2___2 Colorectal surgeon<br>3 pb_si_stom_vag_v2___3 Urologist<br>4 pb_si_stom_vag_v2___4 General surgeon<br>5 pb_si_stom_vag_v2___5 Plastic surgeon<br>6 pb_si_stom_vag_v2___6 Other
2224	[ps_pb_si_stom_vag_v2]<br>Show the field ONLY if:<br>[pb_si_stom_vag_v2(6)] = '1'	Please state	text
2225	[si_anast_vag_v2]	Small bowel anastamosis	yesno<br>1 Yes<br>0 No
2226	[pb_si_anast_vag_v2]<br>Show the field ONLY if:<br>[si_anast_vag_v2] = '1'	Performed by (select all that apply)	checkbox<br>1 pb_si_anast_vag_v2___1 Gynaecological oncologist<br>2 pb_si_anast_vag_v2___2 Colorectal surgeon<br>3 pb_si_anast_vag_v2___3 Urologist<br>4 pb_si_anast_vag_v2___4 General surgeon<br>5 pb_si_anast_vag_v2___5 Plastic surgeon<br>6 pb_si_anast_vag_v2___6 Other

2227	[ps_pb_si_anast_vag_v2]<br>Show the field ONLY if:<br>[pb_si_anast_vag_v2(6)] = '1'	Please state	text
2228	[li_stom_vag_v2]	Large bowel stoma	yesno<br>1 Yes<br>0 No
2229	[pb_li_stom_vag_v2]<br>Show the field ONLY if:<br>[li_stom_vag_v2] = '1'	Performed by (select all that apply)	checkbox<br>1 pb_li_stom_vag_v2___1 Gynaecological oncologist<br>2 pb_li_stom_vag_v2___2 Colorectal surgeon<br>3 pb_li_stom_vag_v2___3 Urologist<br>4 pb_li_stom_vag_v2___4 General surgeon<br>5 pb_li_stom_vag_v2___5 Plastic surgeon<br>6 pb_li_stom_vag_v2___6 Other
2230	[ps_pb_li_stom_vag_v2]<br>Show the field ONLY if:<br>[pb_li_stom_vag_v2(6)] = '1'	Please state	text
2231	[li_anast_vag_v2]	Large bowel anastamosis	yesno<br>1 Yes<br>0 No
2232	[pb_li_anast_vag_v2]<br>Show the field ONLY if:<br>[li_anast_vag_v2] = '1'	Performed by (select all that apply)	checkbox<br>1 pb_li_anast_vag_v2___1 Gynaecological oncologist<br>2 pb_li_anast_vag_v2___2 Colorectal surgeon<br>3 pb_li_anast_vag_v2___3 Urologist<br>4 pb_li_anast_vag_v2___4 General surgeon<br>5 pb_li_anast_vag_v2___5 Plastic surgeon<br>6 pb_li_anast_vag_v2___6 Other
2233	[ps_pb_li_anast_vag_v2]<br>Show the field ONLY if:<br>[pb_li_anast_vag_v2(6)] = '1'	Please state	text
2234	[ic_vag_v2]	Urostomy/ileal conduit	yesno<br>1 Yes<br>0 No
2235	[pb_ic_vag_v2]<br>Show the field ONLY if:<br>[ic_vag_v2] = '1'	Performed by (select all that apply)	checkbox<br>1 pb_ic_vag_v2___1 Gynaecological oncologist<br>2 pb_ic_vag_v2___2 Colorectal surgeon<br>3 pb_ic_vag_v2___3 Urologist<br>4 pb_ic_vag_v2___4 General surgeon<br>5 pb_ic_vag_v2___5 Plastic surgeon<br>6 pb_ic_vag_v2___6 Other
2236	[ps_pb_ic_vag_v2]<br>Show the field ONLY if:<br>[pb_ic_vag_v2(6)] = '1'	Please state	text
2237	[tuu_vag_v2]	Trans uretero-ureteric anastamosis	yesno<br>1 Yes<br>0 No
2238	[pb_tuu_vag_v2]<br>Show the field ONLY if:<br>[tuu_vag_v2] = '1'	Performed by (select all that apply)	checkbox<br>1 pb_tuu_vag_v2___1 Gynaecological oncologist<br>2 pb_tuu_vag_v2___2 Colorectal surgeon<br>3 pb_tuu_vag_v2___3 Urologist<br>4 pb_tuu_vag_v2___4 General surgeon<br>5 pb_tuu_vag_v2___5 Plastic surgeon<br>6 pb_tuu_vag_v2___6 Other

2239	[ps_pb_tuu_vag_v2]<br>Show the field ONLY if:<br>[pb_tuu_vag_v2(6)] = '1'	Please state	text
2240	[neph_vag_v2]	Nephrostomy	yesno<br>1 Yes<br>0 No
2241	[pb_neph_vag_v2]<br>Show the field ONLY if:<br>[neph_vag_v2] = '1'	Performed by (select all that apply)	checkbox<br>1 pb_neph_vag_v2___1 Gynaecological oncologist<br>2 pb_neph_vag_v2___2 Colorectal surgeon<br>3 pb_neph_vag_v2___3 Urologist<br>4 pb_neph_vag_v2___4 General surgeon<br>5 pb_neph_vag_v2___5 Plastic surgeon<br>6 pb_neph_vag_v2___6 Other
2242	[ps_pb_neph_vag_v2]<br>Show the field ONLY if:<br>[pb_neph_vag_v2(6)] = '1'	Please state	text
2243	[vag_recon_v2]	Vaginal reconstruction	yesno<br>1 Yes<br>0 No
2244	[pb_vag_recon_v2]<br>Show the field ONLY if:<br>[vag_recon_v2] = '1'	Performed by (select all that apply)	checkbox<br>1 pb_vag_recon_v2___1 Gynaecological oncologist<br>2 pb_vag_recon_v2___2 Colorectal surgeon<br>3 pb_vag_recon_v2___3 Urologist<br>4 pb_vag_recon_v2___4 General surgeon<br>5 pb_vag_recon_v2___5 Plastic surgeon<br>6 pb_vag_recon_v2___6 Other
2245	[ps_pb_vag_recon_v2]<br>Show the field ONLY if:<br>[pb_vag_recon_v2(6)] = '1'	Please state	text
2246	[vulv_recon_vag_v2]	Vulval reconstruction	yesno<br>1 Yes<br>0 No
2247	[vulvrecon_flap_vag_v2]<br>Show the field ONLY if:<br>[vulv_recon_vag_v2] = '1'	Type of vulval reconstruction	radio<br>1 Rotational flap<br>2 Non-rotational flap<br>3 Graft
2248	[surg_perf_vag_v2]<br>Show the field ONLY if:<br>[vulv_recon_vag_v2] = '1'	Performed by (Select all that apply)	checkbox<br>1 surg_perf_vag_v2___1 Gynaecological oncologist<br>2 surg_perf_vag_v2___2 Colorectal surgeon<br>3 surg_perf_vag_v2___3 Urologist<br>4 surg_perf_vag_v2___4 General surgeon<br>5 surg_perf_vag_v2___5 Plastic surgeon<br>6 surg_perf_vag_v2___6 Other
2249	[ps_surg_perf_vag_v2]<br>Show the field ONLY if:<br>[surg_perf_vag_v2(6)] = '1'	Please state	text
2250	[abx_vag2]	Intraoperative antibiotics	yesno<br>1 Yes<br>0 No
2251	[tim_vag_v2]	Length of recurrence surgery	text (time)
2252	[itu_vag2]	Section Header: *POST-OPERATIVE*<br>Post-operative recovery in intensive care unit	yesno<br>1 Yes<br>0 No

2253	[itu_dys_vag2]<br>Show the field ONLY if:<br>[itu_vag2] = '1'	Number of days in intensive care unit	text (number)
2254	[hdu_vag2]	Post-operative recovery in high dependency unit	yesno<br>1 Yes<br>0 No
2255	[hdu_dys_vag2]<br>Show the field ONLY if:<br>[hdu_vag2] = '1'	Number of days in high dependency unit	text (number)
2256	[er_vag_v2]	Enhanced recovery?	yesno<br>1 Yes<br>0 No
2257	[po_abx_vag2]	Prophylactic post operative antibiotics	yesno<br>1 Yes<br>0 No
2258	[drn_vag2]	Post operative surgical drain	yesno<br>1 Yes<br>0 No
2259	[drn_dys_vag2]<br>Show the field ONLY if:<br>[drn_vag2] = '1'	Number of days in situ	text (number)
2260	[uc_vag2]	Post operative urinary catheter	yesno<br>1 Yes<br>0 No
2261	[cath_dys_vag2]<br>Show the field ONLY if:<br>[uc_vag2] = '1'	Number of days in situ	text (number)
2262	[phb_vag_2]	Post-operative Haemoglobin (g/L) - please state first post-operative result	text (number)
2263	[pwbc_vag_2]	Post-operative White Cell Count (10^9/L) - please state first post-operative result	text (number)
2264	[pplat_vag_2]	Post-operative Platelets (10^9/L) - please state first post-operative result	text (number)
2265	[ppt_vag_2]	Post-operative PT (Secs) - please state first post-operative result	text (number)
2266	[pinr_vag_2]	Post-operative INR (Ratio) - please state first post-operative result	text (number)
2267	[paptt_vag_2]	Post-operative APTT (Secs) - please state first post-operative result	text (number)
2268	[pfib_vag_2]	Post-operative Fibrinogen (g/L) - please state first post-operative result	text (number)
2269	[palb_vag_2]	Post-operative Albumin (g/L) - please state first post-operative result	text (number)
2270	[pna_vag_2]	Post-operative Sodium (mmol/L) - please state first post-operative result	text (number)
2271	[pk_vag_2]	Post-operative Potassium (mmol/L) - please state first post-operative result	text (number)
2272	[purea_vag_2]	Post-operative Urea (mmol/L) - please state first post-operative result	text (number)
2273	[pcreat_vag_2]	Post-operative Creatinine (µmol/L) - please state first post-operative result	text
2274	[pbili_vag_2]	Post-operative Bilirubin total (µmol/L) - please state first post-operative result	text (number)
2275	[pegfr_vag_2]	Post-operative eGFR - Estimated Glomerular Filtration Rate (mL/min) - please state first post-operative result	text
2276	[paat_vag_2]	Post-operative Alanine Aminotransaminase (IU/L) - please state first post-operative result	text (number)
2277	[palp_vag_2]	Post-operative Alkaline Phosphatase (IU/L) - please state first post-operative result	text (number)

2278	[past_vag_2]	Post-operative Aspartate Aminotransaminase (IU/L) - please state first post-operative result	text (number)
2279	[pcrp_vag_2]	Post-operative CRP - C-reactive protein (mg/L) - please state first post-operative result	text (number)
2280	[histo_vag_v2]	Histological type	dropdown<br>1 Squamous carcinoma<br>2 Adenocarcinoma<br>6 Small cell<br>3 Lymphoma<br>4 Sarcoma<br>5 Melanoma
2281	[resect_vag2]	Histological confirmed resection margins	dropdown<br>1 < 1 mm / tumour on inked margin<br>2 1-5 mm (NO tumour on inked margin)<br>3 >5 mm Margins confirmed clear<br>4 Unknown, not available in this hospital
2282	[p16_vag_v2]	Section Header: *Immunohistochemistry*<br>p16	radio (Matrix)<br>1 Positive<br>2 Negative<br>3 Not tested
2283	[ki67_vag_v2]	Ki67	radio (Matrix)<br>1 Positive<br>2 Negative<br>3 Not tested
2284	[p53_vag_v2]	p53	radio (Matrix)<br>1 Positive<br>2 Negative<br>3 Not tested
2285	[cox2_vag_v2]	COX-2	radio (Matrix)<br>1 Positive<br>2 Negative<br>3 Not tested
2286	[asma_vag_v2]	α-SMA	radio (Matrix)<br>1 Positive<br>2 Negative<br>3 Not tested
2287	[cd34_vag_v2]	CD34	radio (Matrix)<br>1 Positive<br>2 Negative<br>3 Not tested
2288	[hmga2_vag_v2]	HMGA2	radio (Matrix)<br>1 Positive<br>2 Negative<br>3 Not tested
2289	[chemadj_vag_v2]	Chemotherapy administered following recurrence diagnosis	yesno<br>1 Yes<br>0 No

2290	[chemreg1_vag_v2]<br>Show the field ONLY if:<br>[chemadj_vag_v2] = '1'	Chemotherapy regimen (Select all that apply)	checkbox<br>1 chemreg1_vag_v2___1 Platinum (for example cisplatin, carboplatin)<br>2 chemreg1_vag_v2___2 Fluorouracil<br>3 chemreg1_vag_v2___3 Taxane (for example paclitaxel)<br>4 chemreg1_vag_v2___4 Irenotecan<br>5 chemreg1_vag_v2___5 Clinical trial regimens<br>6 chemreg1_vag_v2___6 Other
2291	[ps_chemrej1_vag_v2]<br>Show the field ONLY if:<br>[chemreg1_vag_v2(6)] = '1'	Please state	text
2292	[chemadj_cycl_vag_v2]<br>Show the field ONLY if:<br>[chemadj_vag_v2] = '1'	Number of cycles planned	text (number)
2293	[chem_adj_no_vag_v2]<br>Show the field ONLY if:<br>[chemadj_vag_v2] = '0'	Reason chemotherapy not administered following recurrence diagnosis (select all that apply)	checkbox<br>1 chem_adj_no_vag_v2___1 Poor performance status<br>2 chem_adj_no_vag_v2___2 No access<br>3 chem_adj_no_vag_v2___3 Patient unable to afford<br>4 chem_adj_no_vag_v2___4 Patient refused<br>5 chem_adj_no_vag_v2___5 Not clinically indicated<br>6 chem_adj_no_vag_v2___6 Other
2294	[ps_chem_adj_no_vag_v2]<br>Show the field ONLY if:<br>[chem_adj_no_vag_v2(6)] = '1'	Please state	text
2295	[rt_vag_v2]	External beam radiotherapy administered following recurrence diagnosis?	yesno<br>1 Yes<br>0 No
2296	[r_dos_vag2]<br>Show the field ONLY if:<br>[rt_vag_v2] = '1'	Dose received (Gray - Gy)	text (number)
2297	[rt_vag_no_reas_v2]<br>Show the field ONLY if:<br>[rt_vag_v2] = '0'	Reason external beam radiotherapy not administered following recurrence diagnosis (select all that apply)	checkbox<br>1 rt_vag_no_reas_v2___1 Poor performance status<br>2 rt_vag_no_reas_v2___2 No access<br>3 rt_vag_no_reas_v2___3 Patient unable to afford<br>4 rt_vag_no_reas_v2___4 Patient refused<br>5 rt_vag_no_reas_v2___5 Not clinically indicated<br>6 rt_vag_no_reas_v2___6 Other
2298	[ps_rt_vag_no_reas_v2]<br>Show the field ONLY if:<br>[rt_vag_no_reas_v2(6)] = '1'	Please state	text
2299	[brachy_vag_v2]	Brachytherapy administered following recurrence diagnosis	yesno<br>1 Yes<br>0 No
2300	[r_dos_vag3]<br>Show the field ONLY if:<br>[brachy_vag_v2] = '1'	Dose received (Gray - Gy)	text (number)

2301	[brachy_no_reas_vag_v2]<br>Show the field ONLY if:<br>[brachy_vag_v2] = '0'	Reason not administered following recurrence diagnosis (select all that apply)	checkbox<br>1 brachy_no_reas_vag_v2___1 Poor performance status<br>2 brachy_no_reas_vag_v2___2 No access<br>3 brachy_no_reas_vag_v2___3 Patient unable to afford<br>4 brachy_no_reas_vag_v2___4 Patient refused<br>5 brachy_no_reas_vag_v2___5 Not clinically indicated<br>6 brachy_no_reas_vag_v2___6 Other
2302	[ps_brachy_no_reas_vag_v2]<br>Show the field ONLY if:<br>[brachy_no_reas_vag_v2(6)] = '1'	Please state	text
2303	[vaginal_cancer_recurrence_complete]	Section Header: *Form Status*<br>Complete?	dropdown<br>0 Incomplete<br>1 Unverified<br>2 Complete

Instrument: **Malignant gestational trophoblastic disease (initial presentation)** (malignant_gestational_trophoblastic_disease_initia)

2304	[dat_pdx_gtd]	Section Header: *BASELINE DEMOGRAPHICS*<br>Date of primary diagnosis	text (date_dmy)
2305	[gtd_age]	Age of patient	text (number)
2306	[eth_gtd]	Ethnicity	dropdown<br>1 White (British, Australian, North American, European, other white)<br>2 Asian (South East Asian, Far East Asian, other Asian)<br>3 Black (African, Caribbean, other Black)<br>4 Middle Eastern (Arab, North African, Turkish)<br>5 Mixed (multiple ethnic background)<br>6 Other
2307	[ps_eth_gtd]<br>Show the field ONLY if:<br>[eth_gtd] = '6'	Please state	text
2308	[bmi_gtd]	Body mass index (kg/m2)	text (number)

2309	[asa_gtd]	Section Header: *PRE-OPERATIVE*<br>American Society of Anesthesiologists (ASA) physical status	dropdown<br>1 ASA 1: A normal healthy patient. Example: Fit, non-obese (BMI under 30), a nonsmoking patient with good exercise tolerance.<br>2 ASA 2: A patient with a mild systemic disease. Example: Patient with no functional limitations and a well-controlled disease (e.g., treated hypertension, obesity with BMI under 35, frequent social drinker or is a cigarette smoker).<br>3 ASA 3: A patient with a severe systemic disease that is not life-threatening. Example: Patient with some functional limitation as a result of disease (e.g., poorly treated hypertension or diabetes, morbid obesity, chronic renal failure, a bronchospastic disease with intermittent exacerbation, stable angina, implanted pacemaker).<br>4 ASA 4: A patient with a severe systemic disease that is a constant threat to life. Example: Patient with functional limitation from severe, life-threatening disease (e.g., unstable angina, poorly controlled COPD, symptomatic CHF, recent (less than three months ago) myocardial infarction or stroke.<br>5 ASA 5: A moribund patient who is not expected to survive without the operation. The patient is not expected to survive beyond the next 24 hours without surgery. Examples: ruptured abdominal aortic aneurysm, massive trauma, and extensive intracranial hemorrhage with mass effect.<br>6 ASA 6: A brain-dead patient whose organs are being removed with the intention of transplanting them into another patient.
2310	[ecog_gtd]	ECOG (Eastern Cooperative Oncology Group) performance status	dropdown<br>1 Grade 0 - Fully active, able to carry on all pre-disease performance without restriction<br>2 Grade 1 - Restricted in physically strenuous activity but ambulatory and able to carry out work of a light or sedentary nature, e.g., light house work, office work<br>3 Grade 2 - Ambulatory and capable of all selfcare but unable to carry out any work activities; up and about more than 50% of waking hours<br>4 Grade 3 - Capable of only limited selfcare; confined to bed or chair more than 50% of waking hours<br>5 Grade 4 - Completely disabled; cannot carry on any selfcare; totally confined to bed or chair<br>6 Grade 5 - Dead
2311	[comorb_gtd]	Co-morbidities	checkbox<br>1 comorb_gtd___1 Cardiovascular<br>2 comorb_gtd___2 Respiratory<br>3 comorb_gtd___3 Urogenital<br>4 comorb_gtd___4 Endocrine<br>5 comorb_gtd___5 Neurological<br>6 comorb_gtd___6 Haematological<br>7 comorb_gtd___7 Psychiatric<br>8 comorb_gtd___8 Gastroenterology<br>9 comorb_gtd___9 Rheumatology and musculoskeletal<br>10 comorb_gtd___10 Oncology<br>11 comorb_gtd___11 None

2312	[comorb_cvd_gtd]<br>Show the field ONLY if:<br>[comorb_gtd(1)] = '1'	Select all that apply	checkbox<br>1 comorb_cvd_gtd___1 Hypertension<br>2 comorb_cvd_gtd___2 Coronary heart disease / ischaemic heart disease<br>3 comorb_cvd_gtd___3 Myocardial infarction<br>4 comorb_cvd_gtd___4 Heart failure<br>5 comorb_cvd_gtd___5 Arrhythmia<br>6 comorb_cvd_gtd___6 Heart valve disease<br>7 comorb_cvd_gtd___7 Venous insufficiency<br>8 comorb_cvd_gtd___8 Peripheral vascular disease<br>9 comorb_cvd_gtd___9 Stroke<br>10 comorb_cvd_gtd___10 Transient ischaemic attack<br>11 comorb_cvd_gtd___11 Other
2313	[comorb_resp_gtd]<br>Show the field ONLY if:<br>[comorb_gtd(2)] = '1'	Select all that apply	checkbox<br>1 comorb_resp_gtd___1 Asthma<br>2 comorb_resp_gtd___2 Emphysema (COPD)<br>3 comorb_resp_gtd___3 Chronic bronchitis (COPD)<br>4 comorb_resp_gtd___4 Pulmonary fibrosis<br>5 comorb_resp_gtd___5 Sleep apnoea<br>6 comorb_resp_gtd___6 Bronchiectasis<br>7 comorb_resp_gtd___7 Cystic fibrosis<br>8 comorb_resp_gtd___8 Tuberculosis<br>9 comorb_resp_gtd___9 Pneumonia<br>10 comorb_resp_gtd___10 Other
2314	[comorb_uro_gtd]<br>Show the field ONLY if:<br>[comorb_gtd(3)] = '1'	Select all that apply	checkbox<br>1 comorb_uro_gtd___1 Chronic kidney disease (not requiring haemodialysis)<br>2 comorb_uro_gtd___2 Chronic kidney disease (requiring haemodialysis)<br>3 comorb_uro_gtd___3 Polycystic kidney disease<br>4 comorb_uro_gtd___4 Glomerulonephropathy<br>5 comorb_uro_gtd___5 Glomerulonephritis<br>6 comorb_uro_gtd___6 Urinary tract infection<br>7 comorb_uro_gtd___7 Other
2315	[comorb_endo_gtd]<br>Show the field ONLY if:<br>[comorb_gtd(4)] = '1'	Select all that apply	checkbox<br>1 comorb_endo_gtd___1 Type 1 diabetes mellitus<br>2 comorb_endo_gtd___2 Type 2 diabetes mellitus<br>3 comorb_endo_gtd___3 Hyperthyroidism<br>4 comorb_endo_gtd___4 Hypothyroidism<br>5 comorb_endo_gtd___5 Other
2316	[comorb_neuro_gtd]<br>Show the field ONLY if:<br>[comorb_gtd(5)] = '1'	Select all that apply	checkbox<br>1 comorb_neuro_gtd___1 Parkinsons disease<br>2 comorb_neuro_gtd___2 Myasthenia gravis<br>3 comorb_neuro_gtd___3 Dementia<br>4 comorb_neuro_gtd___4 Epilepsy<br>5 comorb_neuro_gtd___5 Other

2317	[comorb_haem_gtd]<br>Show the field ONLY if:<br>[comorb_gtd(6)] = '1'	Select all that apply	checkbox<br>1 comorb_haem_gtd___1 Iron deficiency anaemia<br>2 comorb_haem_gtd___2 Folate deficience anaemia<br>3 comorb_haem_gtd___3 Vitamin B12 deficiency anaemia<br>4 comorb_haem_gtd___4 Pernicious anaemia<br>5 comorb_haem_gtd___5 Haemolytic anaemia<br>6 comorb_haem_gtd___6 Aplastic anaemia<br>7 comorb_haem_gtd___7 Thalassemia<br>8 comorb_haem_gtd___8 Sickle cell disease<br>9 comorb_haem_gtd___9 Disseminated intravascular coagulation (DIC)<br>10 comorb_haem_gtd___10 Factor V Leiden<br>11 comorb_haem_gtd___11 Thrombocytosis<br>12 comorb_haem_gtd___12 Idiopathic thrombocytopenic purpura (ITP)<br>13 comorb_haem_gtd___13 Haemophilia<br>14 comorb_haem_gtd___14 Von Willebrand disease<br>15 comorb_haem_gtd___15 Antiphospholipid syndrome<br>16 comorb_haem_gtd___16 Thrombocytopenia<br>17 comorb_haem_gtd___17 Thrombotic thrombocytopenic purpura<br>18 comorb_haem_gtd___18 Deep vein thrombosis<br>19 comorb_haem_gtd___19 Pulmonary embolism<br>20 comorb_haem_gtd___20 Other
2318	[comorb_pscy_gtd]<br>Show the field ONLY if:<br>[comorb_gtd(7)] = '1'	Select all that apply	checkbox<br>1 comorb_pscy_gtd___1 Anxiety<br>2 comorb_pscy_gtd___2 Depression<br>3 comorb_pscy_gtd___3 Learning difficulties<br>4 comorb_pscy_gtd___4 Personality disorder<br>5 comorb_pscy_gtd___5 Schizophrenia<br>6 comorb_pscy_gtd___6 Bipolar disorder<br>7 comorb_pscy_gtd___7 Autism<br>8 comorb_pscy_gtd___8 Other
2319	[comorb_gi_gtd]<br>Show the field ONLY if:<br>[comorb_gtd(8)] = '1'	Select all that apply	checkbox<br>1 comorb_gi_gtd___1 Crohn's disease<br>2 comorb_gi_gtd___2 Ulcerative colitis<br>3 comorb_gi_gtd___3 Irritable bowel syndrome<br>4 comorb_gi_gtd___4 Celiac Disease<br>5 comorb_gi_gtd___5 Peptic ulcer disease<br>6 comorb_gi_gtd___6 Gallstones<br>7 comorb_gi_gtd___7 Pancreatitis<br>8 comorb_gi_gtd___8 Diverticulitis<br>9 comorb_gi_gtd___9 Non-Alcoholic Fatty Liver Disease<br>10 comorb_gi_gtd___10 Alcoholic fatty liver disease<br>11 comorb_gi_gtd___11 Viral hepatitis<br>12 comorb_gi_gtd___12 Liver failure<br>13 comorb_gi_gtd___13 Other

2320	[comorb_msk_gtd]<br>Show the field ONLY if:<br>[comorb_gtd(9)] = '1'	Select all that apply	checkbox<br>1 comorb_msk_gtd___1 Osteoarthritis<br>2 comorb_msk_gtd___2 Rheumatoid arthritis<br>3 comorb_msk_gtd___3 Osteoporosis<br>4 comorb_msk_gtd___4 Lupus<br>5 comorb_msk_gtd___5 Spondyloarthropathies<br>6 comorb_msk_gtd___6 Sjogren's syndrome<br>7 comorb_msk_gtd___7 Gout<br>8 comorb_msk_gtd___8 Other
2321	[comorb_onc_gtd]<br>Show the field ONLY if:<br>[comorb_gtd(10)] = '1'	Select all that apply	checkbox<br>1 comorb_onc_gtd___1 Breast cancer<br>2 comorb_onc_gtd___2 Lung cancer<br>3 comorb_onc_gtd___3 Hepatobiliary cancer<br>4 comorb_onc_gtd___4 Brain cancer<br>5 comorb_onc_gtd___5 Leukemia<br>6 comorb_onc_gtd___6 Lymphoma<br>7 comorb_onc_gtd___7 Osteosarcoma<br>8 comorb_onc_gtd___8 Other
2322	[lap_gtd]	Previous laparoscopic abdominal surgery	yesno<br>1 Yes<br>0 No
2323	[opn_gtd]	Previous laparotomy	yesno<br>1 Yes<br>0 No
2324	[mdt_gtd]	Discussed at multidisciplinary team meeting/tumour board?	yesno<br>1 Yes<br>0 No
2325	[mdt_tm_gtd]<br>Show the field ONLY if:<br>[mdt_gtd] = '1'	Members of multidisciplinary team/tumour board (select all that apply)	checkbox<br>1 mdt_tm_gtd___1 Certified gynaecological oncology surgeon<br>2 mdt_tm_gtd___2 Trained surgeon dedicated to the management of gynaecologic cancer or having completed an ESGO-accredited fellowship<br>3 mdt_tm_gtd___3 Radiologist<br>4 mdt_tm_gtd___4 Radiation/clinical oncologist (administering radiotherapy)<br>5 mdt_tm_gtd___5 Medical oncologist (administering chemotherapy)<br>6 mdt_tm_gtd___6 Pathologist
2326	[mdt_gtd_no]<br>Show the field ONLY if:<br>[mdt_gtd] = '0'	Reason not discussed (select all that apply)	checkbox<br>1 mdt_gtd_no___1 No multidisciplinary team meeting/tumour board<br>2 mdt_gtd_no___2 Other
2327	[ps_mdt_gtd_no]<br>Show the field ONLY if:<br>[mdt_gtd_no(2)] = '1'	Please state	text
2328	[bx_gtd]	Diagnostic biopsy performed?	yesno<br>1 Yes<br>0 No

2329	[bx_gtd_no]<br>Show the field ONLY if:<br>[bx_gtd] = '0'	Reason diagnostic biopsy not performed	checkbox<br>1 bx_gtd_no___1 Biopsy not technically feasible<br>2 bx_gtd_no___2 Patient refused<br>3 bx_gtd_no___3 Patient unable to afford<br>4 bx_gtd_no___4 Resources not available locally<br>5 bx_gtd_no___5 Other
2330	[ps_bx_gtd_no]<br>Show the field ONLY if:<br>[bx_gtd_no(5)] = '1'	Please state	text
2331	[gtd_mri]	Magnetic resonance imaging (MRI) pelvis/abdomen-pelvis performed at diagnosis?	yesno<br>1 Yes<br>0 No
2332	[mri_find_gtd]<br>Show the field ONLY if:<br>[gtd_mri] = '1'	MRI pelvis/abdomen-pelvis findings at diagnosis (select all that apply)	checkbox<br>1 mri_find_gtd___1 Uterus invasion<br>2 mri_find_gtd___2 Cervical invasion<br>3 mri_find_gtd___3 x1 tubo-ovarian invasion<br>4 mri_find_gtd___4 x2 tubo-ovarian invasion<br>5 mri_find_gtd___5 Vaginal invasion<br>6 mri_find_gtd___6 Urethral invasion<br>7 mri_find_gtd___7 Bladder invasion<br>8 mri_find_gtd___8 x1 hydroureter<br>9 mri_find_gtd___9 x2 hydroureter<br>10 mri_find_gtd___10 Ureteric invasion<br>11 mri_find_gtd___11 x1 hydronephrosis<br>12 mri_find_gtd___12 x2 hydronephrosis<br>13 mri_find_gtd___13 Anal invasion<br>14 mri_find_gtd___14 Rectal invasion<br>15 mri_find_gtd___15 Sigmoid invasion<br>16 mri_find_gtd___16 Pelvic bone invasion<br>17 mri_find_gtd___17 Inguinal lymphadenopathy<br>18 mri_find_gtd___18 Pelvic lymphadenopathy<br>19 mri_find_gtd___19 Para-aortic lymphadenopathy<br>20 mri_find_gtd___20 Other
2333	[ps_mri_find_gtd]<br>Show the field ONLY if:<br>[mri_find_gtd(20)] = '1'	Please state	text
2334	[mri_gtd_no]<br>Show the field ONLY if:<br>[gtd_mri] = '0'	Reason MRI pelvis not performed at diagnosis (Select all that apply)	checkbox<br>1 mri_gtd_no___1 No access to MRI machine<br>2 mri_gtd_no___2 Patient unable to afford<br>3 mri_gtd_no___3 Patient refused<br>4 mri_gtd_no___4 Claustrophobia<br>5 mri_gtd_no___5 Other imaging performed<br>6 mri_gtd_no___6 Extreme morbid obesity<br>7 mri_gtd_no___7 Other
2335	[ps_mri_gtd_no]<br>Show the field ONLY if:<br>[mri_gtd_no(7)] = '1'	Please state	text
2336	[ctcap_gtd]	Staging computerised tomography (CT) performed at diagnosis?	yesno<br>1 Yes<br>0 No

2337	[ctfind_gtd]<br>Show the field ONLY if:<br>[ctcap_gtd] = '1'	Staging CT findings at diagnosis (Select all that apply)	checkbox<br>1 ctfind_gtd___1 Uterus invasion<br>2 ctfind_gtd___2 Cervical invasion<br>3 ctfind_gtd___3 x1 tubo-ovarian invasion<br>4 ctfind_gtd___4 x2 tubo-ovarian invasion<br>5 ctfind_gtd___5 Vaginal invasion<br>6 ctfind_gtd___6 Urethral invasion<br>7 ctfind_gtd___7 Bladder invasion<br>8 ctfind_gtd___8 x1 hydroureter<br>9 ctfind_gtd___9 x2 hydroureter<br>10 ctfind_gtd___10 Ureteric invasion<br>11 ctfind_gtd___11 x1 hydronephrosis<br>12 ctfind_gtd___12 x2 hydronephrosis<br>13 ctfind_gtd___13 Anal invasion<br>14 ctfind_gtd___14 Rectal invasion<br>15 ctfind_gtd___15 Sigmoid invasion<br>16 ctfind_gtd___16 Pelvic bone invasion<br>17 ctfind_gtd___17 Inguinal lymphadenopathy<br>18 ctfind_gtd___18 Pelvic lymphadenopathy<br>19 ctfind_gtd___19 Para-aortic lymphadenopathy<br>20 ctfind_gtd___20 Lung nodules<br>21 ctfind_gtd___21 Pleural effusion<br>22 ctfind_gtd___22 Other
2338	[ps_ctfind_gtd]<br>Show the field ONLY if:<br>[ctfind_gtd(22)] = '1'	Please state	text
2339	[ctcap_gtd_no_reas]<br>Show the field ONLY if:<br>[ctcap_gtd] = '0'	Reason staging CT not performed at diagnosis (state all that apply)	checkbox<br>1 ctcap_gtd_no_reas___1 No access to CT machine<br>2 ctcap_gtd_no_reas___2 Patient unable to afford<br>3 ctcap_gtd_no_reas___3 Patient refused<br>4 ctcap_gtd_no_reas___4 Other imaging performed<br>5 ctcap_gtd_no_reas___5 Allergic reaction to contrast<br>6 ctcap_gtd_no_reas___6 Chronic kidney disease<br>7 ctcap_gtd_no_reas___7 Not clinical protocol<br>8 ctcap_gtd_no_reas___8 Other
2340	[ps_ctcap_no_reas_gtd]<br>Show the field ONLY if:<br>[ctcap_gtd_no_reas(8)] = '1'	Please state	text
2341	[pet_gtd]	PET-CT performed at diagnosis?	yesno<br>1 Yes<br>0 No

2342	[pet_find_gtd]<br>Show the field ONLY if:<br>[pet_gtd] = '1'	PET-CT findings at diagnosis (Select all that apply)	checkbox<br>1 pet_find_gtd___1 Avid -FDG uptake uterus<br>2 pet_find_gtd___2 Avid -FDG uptake cervix<br>3 pet_find_gtd___3 Avid -FDG uptake ovary(s)<br>4 pet_find_gtd___4 Avid -FDG uptake vagina<br>5 pet_find_gtd___5 Avid -FDG uptake urethra<br>6 pet_find_gtd___6 Avid -FDG uptake bladder<br>7 pet_find_gtd___7 Avid -FDG uptake ureter(s)<br>8 pet_find_gtd___8 Avid -FDG uptake kidney(s)<br>9 pet_find_gtd___9 Avid -FDG uptake anus<br>10 pet_find_gtd___10 Avid -FDG uptake rectum<br>11 pet_find_gtd___11 Avid -FDG uptake sigmoid<br>12 pet_find_gtd___12 Avid -FDG uptake para-rectal nodules<br>13 pet_find_gtd___13 Avid -FDG uptake sigmoid nodules<br>14 pet_find_gtd___14 Avid -FDG uptake pelvic bones<br>15 pet_find_gtd___15 Avid -FDG uptake inguinal lymph nodes<br>16 pet_find_gtd___16 Avid -FDG uptake pelvic lymph nodes<br>17 pet_find_gtd___17 Avid -FDG uptake para-aortic lymph nodes<br>18 pet_find_gtd___18 Avid -FDG uptake lung nodules<br>19 pet_find_gtd___19 Other
2343	[ps_pet_fing_gtd]<br>Show the field ONLY if:<br>[pet_find_gtd(19)] = '1'	Please state	text
2344	[pet_no_reaas_gtd]<br>Show the field ONLY if:<br>[pet_gtd] = '0'	Reason PET-CT not performed (Select all that apply)	checkbox<br>1 pet_no_reaas_gtd___1 No access to PET machine<br>2 pet_no_reaas_gtd___2 Patient unable to afford<br>3 pet_no_reaas_gtd___3 Patient refused<br>4 pet_no_reaas_gtd___4 Uncontrolled diabetes mellitus<br>5 pet_no_reaas_gtd___5 Other imaging performed<br>6 pet_no_reaas_gtd___6 Not clinical protocol<br>7 pet_no_reaas_gtd___7 Other
2345	[ps_pet_no_reaas_gtd]<br>Show the field ONLY if:<br>[pet_no_reaas_gtd(7)] = '1'	Please state	text
2346	[gtd_figo]	Clinical FIGO stage (i.e. preoperative staging taking into account imaging, clinical examination)	dropdown<br>1 Stage I Gestational trophoblastic tumours strictly confined to the uterine corpus<br>2 Stage II Gestational trophoblastic tumours extending to the adnexae or to the vagina, but limited to the genital structures<br>3 Stage III Gestational trophoblastic tumours extending to the lungs, with or without genital tract involvement<br>4 Stage IV All other metastatic sites
2347	[nact_gtd]	Neoadjuvant (before surgery) chemotherapy	yesno<br>1 Yes<br>0 No

2348	[gtd_chemreg]<br>Show the field ONLY if:<br>[nact_gtd] = '1'	Chemotherapy FIRST line regimen (Select all that apply)	checkbox<br>1 gtd_chemreg___1 Methotrexate<br>2 gtd_chemreg___2 Actinomycin D/dactinomycin<br>3 gtd_chemreg___3 Cyclophosphamide<br>4 gtd_chemreg___4 Vinca alkaloids (e.g. vincristine)<br>5 gtd_chemreg___5 Etoposide<br>6 gtd_chemreg___6 Other
2349	[ps_gtd_chemreg]<br>Show the field ONLY if:<br>[gtd_chemreg(6)] = '1'	Please state	text
2350	[nact_cycl_gtd]<br>Show the field ONLY if:<br>[nact_gtd] = '1'	Total number of cycles received	text (number)
2351	[nact_no_reas_gtd]<br>Show the field ONLY if:<br>[nact_gtd] = '0'	Reason neoadjuvant chemotherapy not administered (select all that apply)	checkbox<br>5 nact_no_reas_gtd___5 Poor performance status<br>1 nact_no_reas_gtd___1 No access<br>2 nact_no_reas_gtd___2 Patient unable to afford<br>4 nact_no_reas_gtd___4 Patient refused<br>6 nact_no_reas_gtd___6 Not clinically indicated<br>3 nact_no_reas_gtd___3 Other
2352	[ps_nact_no_reas_gtd]<br>Show the field ONLY if:<br>[nact_no_reas_gtd(3)] = '1'	Please state	text
2353	[covid_gtd]	COVID-19 status	dropdown<br>1 Positive<br>2 Negative<br>3 Not tested
2354	[covid_no_reas_gtd]<br>Show the field ONLY if:<br>[covid_gtd] = '3'	Reason not tested (Select all that apply)	checkbox<br>1 covid_no_reas_gtd___1 Not clinically indicated (asymptomatic)<br>2 covid_no_reas_gtd___2 Not clinical protocol to routinely test all patients prior to surgery<br>3 covid_no_reas_gtd___3 Patient refused<br>4 covid_no_reas_gtd___4 Patient unable to afford test<br>5 covid_no_reas_gtd___5 No resources available locally for testing<br>6 covid_no_reas_gtd___6 Other
2355	[ps_covid_no_reas_gtd]<br>Show the field ONLY if:<br>[covid_no_reas_gtd(6)] = '1'	Please state	text
2356	[bp_gtd]	Bowel preparation pre-operatively	yesno<br>1 Yes<br>0 No
2357	[peri_op_gtd]	Peri-operative management plan in place? (Non- exhaustive list: pre-operative haemoglobin optimisation and iron deficit correction; correction of denutrition and immunonutrition; fluid management; pain management; prevention of post-operative nausea and vomiting).	yesno<br>1 Yes<br>0 No
2358	[hb_gtd]	Pre-operative Haemoglobin (g/L)	text (number)
2359	[wcc_gtd]	Pre-operative White Cell Count (10^9/L)	text (number)
2360	[plts_gtd]	Pre-operative Platelets (10^9/L)	text (number)
2361	[pt_gtd]	Pre-operative PT (Secs)	text (number)
2362	[inr_gtd]	Pre-operative INR (ratio)	text (number)
2363	[aptt_gtd]	Pre-operative APTT (Secs)	text (number)

2364	[fib_gtd]	Pre-operative Fibrinogen (g/L)	text (number)
2365	[alb_gtd]	Pre-operative Albumin (g/L)	text (number)
2366	[na_gtd]	Pre-operative Sodium (mmol/L)	text (number)
2367	[k_gtd]	Pre-operative Potassium (mmol/L)	text (number)
2368	[ur_gtd]	Pre-operative Urea (mmol/L)	text (number)
2369	[creat_gtd]	Pre-operative Creatinine (μmol/L)	text (number)
2370	[egfr_gtd]	Pre-operative eGFR - estimated glomerular filtration rate (mL/min)	text
2371	[bili_gtd]	Pre-operative Bilirubin total (μmol/L)	text (number)
2372	[aat_gtd]	Pre-operative Alanine Aminotransaminase (IU/L)	text (number)
2373	[alp_gtd]	Pre-operative Alkaline Phosphatase (IU/L)	text (number)
2374	[ast_gtd]	Pre-operative Aspartate Aminotransaminase (IU/L)	text (number)
2375	[crp_gtd]	Pre-operative CRP - C-reactive protein (mg/L)	text (number)
2376	[dat_gtd]	Section Header: *SURGERY*<br>Date of surgery	text (date_dmy)
2377	[anaes_gtd]	Anaesthesia (Select all that apply)	checkbox<br>1 anaes_gtd___1 General<br>2 anaes_gtd___2 Spinal<br>3 anaes_gtd___3 Epidural
2378	[elecem_gtd]	Was the surgery an elective or emergency case?	radio<br>1 Elective<br>2 Emergency
2379	[sa_gtd]	Surgical aim	dropdown<br>1 Curative<br>2 Curative attempted but then abandoned (i.e. open/close laparotomy)<br>3 Palliative
2380	[sm_gtd]	Surgical modality	dropdown<br>1 Laparotomy<br>2 Laparoscopic<br>3 Robotic<br>6 Vaginal<br>4 Laparoscopic converted to laparotomy<br>5 Robotic converted to laparotomy<br>7 Vaginal converted to laparotomy
2381	[ls_gtd]	Lead surgeon performing surgery	dropdown<br>1 Certified consultant/specialist gynaecological oncologist<br>2 Fellow/trainee gynaecological oncologist<br>3 Trained surgeon dedicated to the management of gynaecologic cancer or having completed an ESGO accredited fellowship.<br>4 General surgeon<br>5 Surgical oncologist<br>6 Other
2382	[ps_ls_gtd]<br>Show the field ONLY if:<br>[ls_gtd] = '6'	Please state	text
2383	[who_gtd]	WHO surgical safety checklist used	yesno<br>1 Yes<br>0 No
2384	[cyto_gtd]	Complete macroscopic cytoreduction at surgery	yesno<br>1 Yes<br>0 No

<table>
<tr><td>2385</td><td>[reas_cytored_no_gtd]
Show the field ONLY if:
[cyto_gtd] = '0'</td><td>Reason(s) disease not fully excised at cytoreduction (select all that apply)</td><td>checkbox<table><tr><td>1</td><td>reas_cytored_no_gtd___1</td><td>Disease more extensive than that shown on imaging</td></tr><tr><td>2</td><td>reas_cytored_no_gtd___2</td><td>Technically unable to resect</td></tr><tr><td>3</td><td>reas_cytored_no_gtd___3</td><td>Patient became medically unstable intra-operatively and decision made to not proceed further with surgery</td></tr><tr><td>4</td><td>reas_cytored_no_gtd___4</td><td>Other</td></tr></table></td></tr>
<tr><td>2386</td><td>[ps_reas_cytored_no_gtd]
Show the field ONLY if:
[reas_cytored_no_gtd(4)] = '1'</td><td>Please state</td><td>text</td></tr>
<tr><td>2387</td><td>[siz_resid_gtd]
Show the field ONLY if:
[cyto_gtd] = '0'</td><td>Size of residual disease (in millimetres)</td><td>dropdown<table><tr><td>1</td><td>1-2.5</td></tr><tr><td>2</td><td>2.6-5</td></tr><tr><td>3</td><td>5.1-7.5</td></tr><tr><td>4</td><td>7.6-10</td></tr><tr><td>5</td><td>>10</td></tr></table></td></tr>
<tr><td>2388</td><td>[loc_resid_gtd]
Show the field ONLY if:
[cyto_gtd] = '0'</td><td>Location of residual disease (select all that apply)</td><td>checkbox<table><tr><td>1</td><td>loc_resid_gtd___1</td><td>Uterus</td></tr><tr><td>2</td><td>loc_resid_gtd___2</td><td>Cervix</td></tr><tr><td>3</td><td>loc_resid_gtd___3</td><td>fallopian tube(s)</td></tr><tr><td>4</td><td>loc_resid_gtd___4</td><td>ovary(s)</td></tr><tr><td>5</td><td>loc_resid_gtd___5</td><td>Vagina</td></tr><tr><td>6</td><td>loc_resid_gtd___6</td><td>Urethra</td></tr><tr><td>7</td><td>loc_resid_gtd___7</td><td>Bladder</td></tr><tr><td>8</td><td>loc_resid_gtd___8</td><td>ureter</td></tr><tr><td>9</td><td>loc_resid_gtd___9</td><td>Kidney(s)</td></tr><tr><td>10</td><td>loc_resid_gtd___10</td><td>Anus</td></tr><tr><td>11</td><td>loc_resid_gtd___11</td><td>Rectum</td></tr><tr><td>12</td><td>loc_resid_gtd___12</td><td>Sigmoid</td></tr><tr><td>13</td><td>loc_resid_gtd___13</td><td>Para rectal nodules</td></tr><tr><td>14</td><td>loc_resid_gtd___14</td><td>Sigmoid nodules</td></tr><tr><td>15</td><td>loc_resid_gtd___15</td><td>Pelvic bone</td></tr><tr><td>16</td><td>loc_resid_gtd___16</td><td>Inguinal lymphadenopathy</td></tr><tr><td>17</td><td>loc_resid_gtd___17</td><td>Pelvic lymphadenopathy</td></tr><tr><td>18</td><td>loc_resid_gtd___18</td><td>Para-aortic lymphadenopathy</td></tr><tr><td>19</td><td>loc_resid_gtd___19</td><td>Other</td></tr></table></td></tr>
<tr><td>2389</td><td>[ps_loc_resid_gtd]
Show the field ONLY if:
[loc_resid_gtd(19)] = '1'</td><td>Please state</td><td>text</td></tr>
</table>

2390	[gtd_surgfind]	Findings at surgery (Select all that apply)	checkbox<br>1 gtd_surgfind___1 Uterus invasion<br>2 gtd_surgfind___2 Cervical invasion<br>3 gtd_surgfind___3 x1 tubo-ovarian invasion<br>4 gtd_surgfind___4 x2 tubo-ovarian invasion<br>5 gtd_surgfind___5 Vaginal invasion<br>6 gtd_surgfind___6 Urethral invasion<br>7 gtd_surgfind___7 Bladder invasion<br>8 gtd_surgfind___8 x1 hydroureter<br>9 gtd_surgfind___9 x2 hydroureter<br>10 gtd_surgfind___10 Ureteric invasion<br>11 gtd_surgfind___11 x1 hydronephrosis<br>12 gtd_surgfind___12 x2 hydronephrosis<br>13 gtd_surgfind___13 Anal invasion<br>14 gtd_surgfind___14 Rectal invasion<br>15 gtd_surgfind___15 Sigmoid invasion<br>16 gtd_surgfind___16 Para rectal nodules<br>17 gtd_surgfind___17 Sigmoid nodules<br>18 gtd_surgfind___18 Pelvic bone invasion<br>19 gtd_surgfind___19 Inguinal lymphadenopathy<br>20 gtd_surgfind___20 Pelvic lymphadenopathy<br>21 gtd_surgfind___21 Para-aortic lymphadenopathy<br>22 gtd_surgfind___22 Other<br>23 gtd_surgfind___23 No visible disease
2391	[ps_gtd_surgfind]<br>Show the field ONLY if:<br>[gtd_surgfind(22)] = '1'	Please state	text
2392	[tis_surg_gtd]	Tissue removed at surgery (Select all that apply)	checkbox<br>1 tis_surg_gtd___1 Uterus<br>2 tis_surg_gtd___2 Cervix<br>3 tis_surg_gtd___3 x1 ovary<br>4 tis_surg_gtd___4 x2 ovaries<br>5 tis_surg_gtd___5 Vagina<br>6 tis_surg_gtd___6 Urethra<br>7 tis_surg_gtd___7 ureter<br>8 tis_surg_gtd___8 kidney<br>9 tis_surg_gtd___9 Anus<br>10 tis_surg_gtd___10 Rectum<br>11 tis_surg_gtd___11 Sigmoid<br>12 tis_surg_gtd___12 Inguinal nodes<br>13 tis_surg_gtd___13 Pelvic lymph nodes<br>14 tis_surg_gtd___14 Para-aortic nodes<br>15 tis_surg_gtd___15 Other
2393	[paln_dis_gtd]<br>Show the field ONLY if:<br>[tis_surg_gtd(14)] = '1'	Select all that apply	checkbox<br>1 paln_dis_gtd___1 Infra-renal<br>2 paln_dis_gtd___2 Supra-renal
2394	[ps_tis_surg_gtd]<br>Show the field ONLY if:<br>[tis_surg_gtd(15)] = '1'	Please state	text
2395	[si_stom_gtd]	Small bowel stoma	yesno<br>1 Yes<br>0 No

2396	[ss_gtd]<br>Show the field ONLY if:<br>[si_stom_gtd] = '1'	Stoma site	dropdown<br>1 Duodenum<br>2 Jejunum<br>3 Ileum
2397	[pb_si_stom_gtd]<br>Show the field ONLY if:<br>[si_stom_gtd] = '1'	Performed by (select all that apply)	checkbox<br>1 pb_si_stom_gtd___1 Gynaecological oncologist<br>2 pb_si_stom_gtd___2 Colorectal surgeon<br>3 pb_si_stom_gtd___3 Urologist<br>4 pb_si_stom_gtd___4 General surgeon<br>5 pb_si_stom_gtd___5 Plastic surgeon<br>6 pb_si_stom_gtd___6 Other
2398	[ps_pb_si_stom_gtd]<br>Show the field ONLY if:<br>[pb_si_stom_gtd(6)] = '1'	Please state	text
2399	[si_anast_gtd]	Small bowel anastamosis	yesno<br>1 Yes<br>0 No
2400	[pb_si_anast_gtd]<br>Show the field ONLY if:<br>[si_anast_gtd] = '1'	Performed by (select all that apply)	checkbox<br>1 pb_si_anast_gtd___1 Gynaecological oncologist<br>2 pb_si_anast_gtd___2 Colorectal surgeon<br>3 pb_si_anast_gtd___3 Urologist<br>4 pb_si_anast_gtd___4 General surgeon<br>5 pb_si_anast_gtd___5 Plastic surgeon<br>6 pb_si_anast_gtd___6 Other
2401	[ps_pb_si_anast_gtd]<br>Show the field ONLY if:<br>[pb_si_anast_gtd(6)] = '1'	Please state	text
2402	[li_stoma_gtd]	Large bowel stoma	yesno<br>1 Yes<br>0 No
2403	[pb_li_stom_gtd]<br>Show the field ONLY if:<br>[li_stoma_gtd] = '1'	Performed by (select all that apply)	checkbox<br>1 pb_li_stom_gtd___1 Gynaecological oncologist<br>2 pb_li_stom_gtd___2 Colorectal surgeon<br>3 pb_li_stom_gtd___3 Urologist<br>4 pb_li_stom_gtd___4 General surgeon<br>5 pb_li_stom_gtd___5 Plastic surgeon<br>6 pb_li_stom_gtd___6 Other
2404	[ps_pb_li_stom_gtd]<br>Show the field ONLY if:<br>[pb_li_stom_gtd(6)] = '1'	Please state	text
2405	[li_anast_gtd]	Large bowel anastamosis	yesno<br>1 Yes<br>0 No
2406	[pb_li_anast_gtd]<br>Show the field ONLY if:<br>[li_anast_gtd] = '1'	Performed by (select all that apply)	checkbox<br>1 pb_li_anast_gtd___1 Gynaecological oncologist<br>2 pb_li_anast_gtd___2 Colorectal surgeon<br>3 pb_li_anast_gtd___3 Urologist<br>4 pb_li_anast_gtd___4 General surgeon<br>5 pb_li_anast_gtd___5 Plastic surgeon<br>6 pb_li_anast_gtd___6 Other
2407	[ps_pb_li_anast_gtd]<br>Show the field ONLY if:<br>[pb_li_anast_gtd(6)] = '1'	Please state	text

2408	[ui_gtd]	Urostomy/ileal conduit	yesno<br>1 Yes<br>0 No
2409	[pb_ui_gtd]<br>Show the field ONLY if:<br>[ui_gtd] = '1'	Performed by (select all that apply)	checkbox<br>1 pb_ui_gtd___1 Gynaecological oncologist<br>2 pb_ui_gtd___2 Colorectal surgeon<br>3 pb_ui_gtd___3 Urologist<br>4 pb_ui_gtd___4 General surgeon<br>5 pb_ui_gtd___5 Plastic surgeon<br>6 pb_ui_gtd___6 Other
2410	[ps_pb_ui_gtd]<br>Show the field ONLY if:<br>[pb_ui_gtd(6)] = '1'	Please state	text
2411	[tuu_gtd]	Trans uretero-ureteric anastamosis	yesno<br>1 Yes<br>0 No
2412	[pb_tuu_gtd]<br>Show the field ONLY if:<br>[tuu_gtd] = '1'	Performed by (select all that apply)	checkbox<br>1 pb_tuu_gtd___1 Gynaecological oncologist<br>2 pb_tuu_gtd___2 Colorectal surgeon<br>3 pb_tuu_gtd___3 Urologist<br>4 pb_tuu_gtd___4 General surgeon<br>5 pb_tuu_gtd___5 Plastic surgeon<br>6 pb_tuu_gtd___6 Other
2413	[ps_pb_tuu_gtd]<br>Show the field ONLY if:<br>[pb_tuu_gtd(6)] = '1'	Please state	text
2414	[neph_gtd]	Nephrostomy	yesno<br>1 Yes<br>0 No
2415	[surgeon_gtd_add]<br>Show the field ONLY if:<br>[neph_gtd] = '1'	Performed by (Select all that apply)	checkbox<br>1 surgeon_gtd_add___1 Gynaecological oncologist<br>2 surgeon_gtd_add___2 Colorectal surgeon<br>3 surgeon_gtd_add___3 Urologist<br>4 surgeon_gtd_add___4 General surgeon<br>5 surgeon_gtd_add___5 Plastic surgeon<br>6 surgeon_gtd_add___6 Other
2416	[ps_surgeon_gtd_add]<br>Show the field ONLY if:<br>[surgeon_gtd_add(6)] = '1'	Please state	text
2417	[abx_gtd]	Intraoperative antibiotics	yesno<br>1 Yes<br>0 No
2418	[tim_gtd]	Length of surgery	text (time)
2419	[itu_gtd]	Section Header: *POST-OPERATIVE*<br>Post-operative recovery in intensive care unit	yesno<br>1 Yes<br>0 No
2420	[itu_dys_gtd]<br>Show the field ONLY if:<br>[itu_gtd] = '1'	Number of days in intensive care unit	text (number)
2421	[hdu_gtd]	Post-operative recovery in high dependency unit	yesno<br>1 Yes<br>0 No

2422	[hdu_dys_gtd]<br>Show the field ONLY if:<br>[hdu_gtd] = '1'	Number of days in high dependency unit	text (number)
2423	[er_gtd]	Enhanced recovery?	yesno<br>1 Yes<br>0 No
2424	[po_abx_gtd2]	Prophylactic post operative antibiotics	yesno<br>1 Yes<br>0 No
2425	[drn_gtd]	Post operative surgical drain	yesno<br>1 Yes<br>0 No
2426	[drn_dys_gtd]<br>Show the field ONLY if:<br>[drn_gtd] = '1'	Number of days in situ	text (number)
2427	[uc_gtd]	Post operative urinary catheter	yesno<br>1 Yes<br>0 No
2428	[cath_dys_gtd]<br>Show the field ONLY if:<br>[uc_gtd] = '1'	Number of days in situ	text (number)
2429	[phb_gtd]	Post-operative Haemoglobin (g/L) - please state first post-operative result	text (number)
2430	[pwbc_gtd]	Post-operative White Cell Count (10^9/L) - please state first post-operative result	text (number)
2431	[pplat_gtd]	Post-operative Platelets (10^9/L) - please state first post-operative result	text (number)
2432	[ppt_gtd]	Post-operative PT (Secs) - please state first post-operative result	text (number)
2433	[pinr_gtd]	Post-operative INR (Ratio) - please state first post-operative result	text (number)
2434	[paptt_gtd]	Post-operative APTT (Secs) - please state first post-operative result	text (number)
2435	[pfib_gtd]	Post-operative Fibrinogen (g/L) - please state first post-operative result	text (number)
2436	[palb_gtd]	Post-operative Albumin (g/L) - please state first post-operative result	text (number)
2437	[pna_gtd]	Post-operative Sodium (mmol/L) - please state first post-operative result	text (number)
2438	[pk_gtd]	Post-operative Potassium (mmol/L) - please state first post-operative result	text (number)
2439	[purea_gtd]	Post-operative Urea (mmol/L) - please state first post-operative result	text (number)
2440	[pcreat_gtd]	Post-operative Creatinine (µmol/L) - please state first post-operative result	text
2441	[pegfr_gtd]	Post-operative eGFR - Estimated Glomerular Filtration Rate (mL/min) - please state first post-operative result	text
2442	[pbili_gtd]	Post-operative Bilirubin total (µmol/L) - please state first post-operative result	text (number)
2443	[paat_gtd]	Post-operative Alanine Aminotransaminase (IU/L) - please state first post-operative result	text (number)
2444	[palp_gtd]	Post-operative Alkaline Phosphatase (IU/L) - please state first post-operative result	text (number)
2445	[past_gtd]	Post-operative Aspartate Aminotransaminase (IU/L) - please state first post-operative result	text (number)
2446	[pcrp_gtd]	Post-operative CRP - C-reactive protein (mg/L) - please state first post-operative result	text (number)

2447	[histo_gtd]	Histopathology	dropdown<br>2 Choriocarcinoma<br>3 Placenta site trophoblastic tumour<br>4 Epithelioid trophoblastic tumour
2448	[resec_gtd]	Histological confirmed resection margins	dropdown<br>1 < 1 mm / tumour on margin<br>2 1-5 mm (NO tumour on margin)<br>3 >5 mm Margins confirmed clear<br>4 Unknown, not available in this hospital
2449	[past_gtd_2]	Post-operative Aspartate Aminotransferase (IU/L) - please state first post-operative result	text (number)
2450	[ckae1_gtd]	Section Header: *Immunohistochemistry*<br>CK AE1	radio (Matrix)<br>1 Positive<br>2 Negative<br>3 Not tested
2451	[ckae3_gtd]	CK AE3	radio (Matrix)<br>1 Positive<br>2 Negative<br>3 Not tested
2452	[p63_gtd]	p63	radio (Matrix)<br>1 Positive<br>2 Negative<br>3 Not tested
2453	[hpl_gtd]	hPL	radio (Matrix)<br>1 Positive<br>2 Negative<br>3 Not tested
2454	[melcam_gtd]	Mel-CAM	radio (Matrix)<br>1 Positive<br>2 Negative<br>3 Not tested
2455	[ki67_gtd]	Ki-67	radio (Matrix)<br>1 Positive<br>2 Negative<br>3 Not tested
2456	[gtd_figo_2]	Final surgical FIGO Stage (i.e. postoperative staging taking into account surgical and pathology findings)	dropdown<br>1 Stage I Gestational trophoblastic tumours strictly confined to the uterine corpus<br>2 Stage II Gestational trophoblastic tumours extending to the adnexae or to the vagina, but limited to the genital structures<br>3 Stage III Gestational trophoblastic tumours extending to the lungs, with or without genital tract involvement<br>4 Stage IV All other metastatic sites
2457	[adjchem_gtd]	Adjuvant (after surgery) chemotherapy	yesno<br>1 Yes<br>0 No

2458	[adjchem1_gtd]<br>Show the field ONLY if:<br>[adjchem_gtd] = '1'	Chemotherapy FIRST line regimen (select all that apply)	checkbox<br>1 adjchem1_gtd___1 Methotrexate<br>2 adjchem1_gtd___2 Actinomycin D/dactinomycin<br>3 adjchem1_gtd___3 Cyclophosphamide<br>4 adjchem1_gtd___4 Vinca alkaloids (e.g. vincristine)<br>5 adjchem1_gtd___5 Etoposide<br>6 adjchem1_gtd___6 Other
2459	[ps_adjchem1_gtd]<br>Show the field ONLY if:<br>[adjchem1_gtd(6)] = '1'	Please state	text
2460	[adjchem_cycl_gtd]<br>Show the field ONLY if:<br>[adjchem_gtd] = '1'	Number of cycles planned	text (number)
2461	[adjchem_no_reas_gtd]<br>Show the field ONLY if:<br>[adjchem_gtd] = '0'	Reason adjuvant chemotherapy not administered (Select all that apply)	checkbox<br>1 adjchem_no_reas_gtd___1 Poor performance status<br>2 adjchem_no_reas_gtd___2 No access<br>3 adjchem_no_reas_gtd___3 Patient unable to afford<br>4 adjchem_no_reas_gtd___4 Patient refused<br>5 adjchem_no_reas_gtd___5 Not clinically indicated<br>6 adjchem_no_reas_gtd___6 Other
2462	[ps_adjchem_no_reas_gtd]<br>Show the field ONLY if:<br>[adjchem_no_reas_gtd(6)] = '1'	Please state	text
2463	[rt_gtd]	External beam radiotherapy administered	yesno<br>1 Yes<br>0 No
2464	[rt_no_reas_gtd]<br>Show the field ONLY if:<br>[rt_gtd] = '0'	Reason external beam radiotherapy not administered (Select all that apply)	checkbox<br>1 rt_no_reas_gtd___1 Poor performance status<br>2 rt_no_reas_gtd___2 No access<br>3 rt_no_reas_gtd___3 Patient unable to afford<br>4 rt_no_reas_gtd___4 Patient refused<br>5 rt_no_reas_gtd___5 Not clinically indicated<br>6 rt_no_reas_gtd___6 Other
2465	[rt_dos_gtd]<br>Show the field ONLY if:<br>[rt_gtd] = '1'	Dose received (Gray - Gy)	text (number)
2466	[ps_rt_no_reas_gtd]<br>Show the field ONLY if:<br>[rt_no_reas_gtd(6)] = '1'	Please state	text
2467	[brachy_gtd]	Brachytherapy administered	yesno<br>1 Yes<br>0 No
2468	[rt_dos_gtd_2]<br>Show the field ONLY if:<br>[brachy_gtd] = '1'	Dose received (Gray - Gy)	text (number)

2469	[brachy_no_reas_gtd]<br>Show the field ONLY if:<br>[brachy_gtd] = '0'	Reason brachytherapy not administered (Select all that apply)	checkbox<br>1 brachy_no_reas_gtd___1 Poor performance status<br>2 brachy_no_reas_gtd___2 No access<br>3 brachy_no_reas_gtd___3 Patient unable to afford<br>4 brachy_no_reas_gtd___4 Patient refused<br>5 brachy_no_reas_gtd___5 Not clinically indicated<br>6 brachy_no_reas_gtd___6 Other
2470	[ps_brachy_no_reas_gtd]<br>Show the field ONLY if:<br>[brachy_no_reas_gtd(6)] = '1'	Please state	text
2471	[malignant_gestational_trophoblastic_disease_initia_complete]	Section Header: *Form Status*<br>Complete?	dropdown<br>0 Incomplete<br>1 Unverified<br>2 Complete
Instrument: **Malignant gestational trophoblastic disease (recurrence)** (malignant_gestational_trophoblastic_disease_recurr)			
2472	[dat_dx_gtd]	Section Header: *PREVIOUS TREATMENT RECEIVED AT PRIMARY DIAGNOSIS*<br>Date of primary/initial diagnosis	text (date_dmy)
2473	[prim_tx_gtd]	Primary treatment following primary/initial diagnosis (select all that apply)	checkbox<br>1 prim_tx_gtd___1 Cytoreductive surgery<br>2 prim_tx_gtd___2 Adjuvant chemotherapy<br>3 prim_tx_gtd___3 Neoadjuvant chemotherapy<br>4 prim_tx_gtd___4 External beam radiotherapy<br>5 prim_tx_gtd___5 Brachytherapy<br>6 prim_tx_gtd___6 Immunotherapy
2474	[dat_surg1_gtd]<br>Show the field ONLY if:<br>[prim_tx_gtd(1)] = '1'	Date of initial cytoreductive surgery	text (date_dmy)
2475	[dat_chem1_gtd]<br>Show the field ONLY if:<br>[prim_tx_gtd(2)] = '1'	Date of adjuvant chemotherapy following initial diagnosis	text (date_dmy)
2476	[dat_nact1_gtd]<br>Show the field ONLY if:<br>[prim_tx_gtd(3)] = '1'	Date of neoadjuvant chemotherapy following initial diagnosis	text (date_dmy)
2477	[dat_rt1_gtd]<br>Show the field ONLY if:<br>[prim_tx_gtd(4)] = '1'	Date of external beam radiotherapy following initial diagnosis	text (date_dmy)
2478	[dat_brachy1_gtd]<br>Show the field ONLY if:<br>[prim_tx_gtd(5)] = '1'	Date of brachytherapy following initial diagnosis	text (date_dmy)
2479	[dat_imm_gtd_2]<br>Show the field ONLY if:<br>[prim_tx_gtd(6)] = '1'	Date of immunotherapy following initial diagnosis	text (date_dmy)
2480	[dat_rec_dx_gtd]	Date of recurrence diagnosis	text (date_dmy)
2481	[gtd_age_v2]	Section Header: *BASELINE DEMOGRAPHICS*<br>Age of patient	text (number)
2482	[eth_gtd_v2]	Ethnicity	dropdown<br>1 White (British, Australian, North American, European, other white)<br>2 Asian (South East Asian, Far East Asian, other Asian)<br>3 Black (African, Caribbean, other Black)<br>4 Middle Eastern (Arab, North African, Turkish)<br>5 Mixed (multiple ethnic background)<br>6 Other

2483	[ps_eth_gtd_v2]<br>Show the field ONLY if:<br>[eth_gtd_v2] = '6'	Please state	text
2484	[bmi_gtd_v2]	Body mass index (kg/m2)	text (number)
2485	[asa_gtd_v2]	Section Header: *PRE-OPERATIVE*<br>American Society of Anesthesiologists (ASA) physical status	dropdown<br>1 ASA 1: A normal healthy patient. Example: Fit, non-obese (BMI under 30), a nonsmoking patient with good exercise tolerance.<br>2 ASA 2: A patient with a mild systemic disease. Example: Patient with no functional limitations and a well-controlled disease (e.g., treated hypertension, obesity with BMI under 35, frequent social drinker or is a cigarette smoker).<br>3 ASA 3: A patient with a severe systemic disease that is not life-threatening. Example: Patient with some functional limitation as a result of disease (e.g., poorly treated hypertension or diabetes, morbid obesity, chronic renal failure, a bronchospastic disease with intermittent exacerbation, stable angina, implanted pacemaker).<br>4 ASA 4: A patient with a severe systemic disease that is a constant threat to life. Example: Patient with functional limitation from severe, life-threatening disease (e.g., unstable angina, poorly controlled COPD, symptomatic CHF, recent (less than three months ago) myocardial infarction or stroke.<br>5 ASA 5: A moribund patient who is not expected to survive without the operation. The patient is not expected to survive beyond the next 24 hours without surgery. Examples: ruptured abdominal aortic aneurysm, massive trauma, and extensive intracranial hemorrhage with mass effect.<br>6 ASA 6: A brain-dead patient whose organs are being removed with the intention of transplanting them into another patient.
2486	[ecog_gtd_v2]	ECOG (Eastern Cooperative Oncology Group) performance status	dropdown<br>1 Grade 0 - Fully active, able to carry on all pre-disease performance without restriction<br>2 Grade 1 - Restricted in physically strenuous activity but ambulatory and able to carry out work of a light or sedentary nature, e.g., light house work, office work<br>3 Grade 2 - Ambulatory and capable of all selfcare but unable to carry out any work activities; up and about more than 50% of waking hours<br>4 Grade 3 - Capable of only limited selfcare; confined to bed or chair more than 50% of waking hours<br>5 Grade 4 - Completely disabled; cannot carry on any selfcare; totally confined to bed or chair<br>6 Grade 5 - Dead

2487	[comorb_gtd2]	Co-morbidities	checkbox<br>1 comorb_gtd2___1 Cardiovascular<br>2 comorb_gtd2___2 Respiratory<br>3 comorb_gtd2___3 Urogenital<br>4 comorb_gtd2___4 Endocrine<br>5 comorb_gtd2___5 Neurological<br>6 comorb_gtd2___6 Haematological<br>7 comorb_gtd2___7 Psychiatric<br>8 comorb_gtd2___8 Gastroenterology<br>9 comorb_gtd2___9 Rheumatology and musculoskeletal<br>10 comorb_gtd2___10 Oncology<br>11 comorb_gtd2___11 None
2488	[comorb_cvd_gtd2]<br>Show the field ONLY if:<br>[comorb_gtd2(1)] = '1'	Select all that apply	checkbox<br>1 comorb_cvd_gtd2___1 Hypertension<br>2 comorb_cvd_gtd2___2 Coronary heart disease / ischaemic heart disease<br>3 comorb_cvd_gtd2___3 Myocardial infarction<br>4 comorb_cvd_gtd2___4 Heart failure<br>5 comorb_cvd_gtd2___5 Arrhythmia<br>6 comorb_cvd_gtd2___6 Heart valve disease<br>7 comorb_cvd_gtd2___7 Venous insufficiency<br>8 comorb_cvd_gtd2___8 Peripheral vascular disease<br>9 comorb_cvd_gtd2___9 Stroke<br>10 comorb_cvd_gtd2___10 Transient ischaemic attack<br>11 comorb_cvd_gtd2___11 Other
2489	[comorb_resp_gtd2]<br>Show the field ONLY if:<br>[comorb_gtd2(2)] = '1'	Select all that apply	checkbox<br>1 comorb_resp_gtd2___1 Asthma<br>2 comorb_resp_gtd2___2 Emphysema (COPD)<br>3 comorb_resp_gtd2___3 Chronic bronchitis (COPD)<br>4 comorb_resp_gtd2___4 Pulmonary fibrosis<br>5 comorb_resp_gtd2___5 Sleep apnoea<br>6 comorb_resp_gtd2___6 Bronchiectasis<br>7 comorb_resp_gtd2___7 Cystic fibrosis<br>8 comorb_resp_gtd2___8 Tuberculosis<br>9 comorb_resp_gtd2___9 Pneumonia<br>10 comorb_resp_gtd2___10 Other
2490	[comorb_uro_gtd2]<br>Show the field ONLY if:<br>[comorb_gtd2(3)] = '1'	Select all that apply	checkbox<br>1 comorb_uro_gtd2___1 Chronic kidney disease (not requiring haemodialysis)<br>2 comorb_uro_gtd2___2 Chronic kidney disease (requiring haemodialysis)<br>3 comorb_uro_gtd2___3 Polycystic kidney disease<br>4 comorb_uro_gtd2___4 Glomerulonephropathy<br>5 comorb_uro_gtd2___5 Glomerulonephritis<br>6 comorb_uro_gtd2___6 Urinary tract infection<br>7 comorb_uro_gtd2___7 Other

2491	[comorb_endo_gtd2]<br>Show the field ONLY if:<br>[comorb_gtd2(4)] = '1'	Select all that apply	checkbox<br>1 comorb_endo_gtd2___1 Type 1 diabetes mellitus<br>2 comorb_endo_gtd2___2 Type 2 diabetes mellitus<br>3 comorb_endo_gtd2___3 Hyperthyroidism<br>4 comorb_endo_gtd2___4 Hypothyroidism<br>5 comorb_endo_gtd2___5 Other
2492	[comorb_neuro_gtd2]<br>Show the field ONLY if:<br>[comorb_gtd2(5)] = '1'	Select all that apply	checkbox<br>1 comorb_neuro_gtd2___1 Parkinsons disease<br>2 comorb_neuro_gtd2___2 Myasthenia gravis<br>3 comorb_neuro_gtd2___3 Dementia<br>4 comorb_neuro_gtd2___4 Epilepsy<br>5 comorb_neuro_gtd2___5 Other
2493	[comorb_haem_gtd2]<br>Show the field ONLY if:<br>[comorb_gtd2(6)] = '1'	Select all that apply	checkbox<br>1 comorb_haem_gtd2___1 Iron deficiency anaemia<br>2 comorb_haem_gtd2___2 Folate deficience anaemia<br>3 comorb_haem_gtd2___3 Vitamin B12 deficiency anaemia<br>4 comorb_haem_gtd2___4 Pernicious anaemia<br>5 comorb_haem_gtd2___5 Haemolytic anaemia<br>6 comorb_haem_gtd2___6 Aplastic anaemia<br>7 comorb_haem_gtd2___7 Thalassemia<br>8 comorb_haem_gtd2___8 Sickle cell disease<br>9 comorb_haem_gtd2___9 Disseminated intravascular coagulation (DIC)<br>10 comorb_haem_gtd2___10 Factor V Leiden<br>11 comorb_haem_gtd2___11 Thrombocytosis<br>12 comorb_haem_gtd2___12 Idiopathic thrombocytopenic purpura (ITP)<br>13 comorb_haem_gtd2___13 Haemophilia<br>14 comorb_haem_gtd2___14 Von Willebrand disease<br>15 comorb_haem_gtd2___15 Antiphospholipid syndrome<br>16 comorb_haem_gtd2___16 Thrombocytopenia<br>17 comorb_haem_gtd2___17 Thrombotic thrombocytopenic purpura<br>18 comorb_haem_gtd2___18 Deep vein thrombosis<br>19 comorb_haem_gtd2___19 Pulmonary embolism<br>20 comorb_haem_gtd2___20 Other
2494	[comorb_pscy_gtd2]<br>Show the field ONLY if:<br>[comorb_gtd2(7)] = '1'	Select all that apply	checkbox<br>1 comorb_pscy_gtd2___1 Anxiety<br>2 comorb_pscy_gtd2___2 Depression<br>3 comorb_pscy_gtd2___3 Learning difficulties<br>4 comorb_pscy_gtd2___4 Personality disorder<br>5 comorb_pscy_gtd2___5 Schizophrenia<br>6 comorb_pscy_gtd2___6 Bipolar disorder<br>7 comorb_pscy_gtd2___7 Autism<br>8 comorb_pscy_gtd2___8 Other

2495	[comorb_gi_gtd_2]<br>Show the field ONLY if:<br>[comorb_gtd2(8)] = '1'	Select all that apply	checkbox<br>1 comorb_gi_gtd_2___1 Crohn's disease<br>2 comorb_gi_gtd_2___2 Ulcerative colitis<br>3 comorb_gi_gtd_2___3 Irritable bowel syndrome<br>4 comorb_gi_gtd_2___4 Celiac Disease<br>5 comorb_gi_gtd_2___5 Peptic ulcer disease<br>6 comorb_gi_gtd_2___6 Gallstones<br>7 comorb_gi_gtd_2___7 Pancreatitis<br>8 comorb_gi_gtd_2___8 Diverticulitis<br>9 comorb_gi_gtd_2___9 Non-Alcoholic Fatty Liver Disease<br>10 comorb_gi_gtd_2___10 Alcoholic fatty liver disease<br>11 comorb_gi_gtd_2___11 Viral hepatitis<br>12 comorb_gi_gtd_2___12 Liver failure<br>13 comorb_gi_gtd_2___13 Other
2496	[comorb_msk_gtd_2]<br>Show the field ONLY if:<br>[comorb_gtd2(9)] = '1'	Select all that apply	checkbox<br>1 comorb_msk_gtd_2___1 Osteoarthritis<br>2 comorb_msk_gtd_2___2 Rheumatoid arthritis<br>3 comorb_msk_gtd_2___3 Osteoporosis<br>4 comorb_msk_gtd_2___4 Lupus<br>5 comorb_msk_gtd_2___5 Spondyloarthropathies<br>6 comorb_msk_gtd_2___6 Sjogren's syndrome<br>7 comorb_msk_gtd_2___7 Gout<br>8 comorb_msk_gtd_2___8 Other
2497	[comorb_onc_gtd_2]<br>Show the field ONLY if:<br>[comorb_gtd2(10)] = '1'	Select all that apply	checkbox<br>1 comorb_onc_gtd_2___1 Breast cancer<br>2 comorb_onc_gtd_2___2 Lung cancer<br>3 comorb_onc_gtd_2___3 Hepatobiliary cancer<br>4 comorb_onc_gtd_2___4 Brain cancer<br>5 comorb_onc_gtd_2___5 Leukemia<br>6 comorb_onc_gtd_2___6 Lymphoma<br>7 comorb_onc_gtd_2___7 Osteosarcoma<br>8 comorb_onc_gtd_2___8 Other
2498	[lap_gtd2]	Previous laparoscopic abdominal surgery	yesno<br>1 Yes<br>0 No
2499	[opn_gtd2]	Previous laparotomy	yesno<br>1 Yes<br>0 No
2500	[mdt_gtd_v2]	Discussed at multidisciplinary team meeting/tumour board?	yesno<br>1 Yes<br>0 No

2501	[mdt_tm_gtd2]<br>Show the field ONLY if:<br>[mdt_gtd_v2] = '1'	Members of multidisciplinary team/tumour board (select all that apply)	checkbox<br>1 mdt_tm_gtd2___1 Certified gynaecological oncology surgeon<br>2 mdt_tm_gtd2___2 Trained surgeon dedicated to the management of gynaecologic cancer or having completed an ESGO-accredited fellowship<br>3 mdt_tm_gtd2___3 Radiologist<br>4 mdt_tm_gtd2___4 Radiation/clinical oncologist (administering radiotherapy)<br>5 mdt_tm_gtd2___5 Medical oncologist (administering chemotherapy)<br>6 mdt_tm_gtd2___6 Pathologist
2502	[mdt_gtd_no_v2]<br>Show the field ONLY if:<br>[mdt_gtd_v2] = '0'	Reason not discussed (select all that apply)	checkbox<br>1 mdt_gtd_no_v2___1 No multidisciplinary team meeting/tumour board<br>2 mdt_gtd_no_v2___2 Other
2503	[ps_mdt_gtd_no_v2]<br>Show the field ONLY if:<br>[mdt_gtd_no_v2(2)] = '1'	Please state	text
2504	[gtd_figo_v2]	FIGO Stage	dropdown<br>1 Stage I Gestational trophoblastic tumours strictly confined to the uterine corpus<br>2 Stage II Gestational trophoblastic tumours extending to the adnexae or to the vagina, but limited to the genital structures<br>3 Stage III Gestational trophoblastic tumours extending to the lungs, with or without genital tract involvement<br>4 Stage IV All other metastatic sites
2505	[bx_gtd_v2]	Diagnostic biopsy performed at recurrence diagnosis?	yesno<br>1 Yes<br>0 No
2506	[bx_gtd_no_v2]<br>Show the field ONLY if:<br>[bx_gtd_v2] = '0'	Reason diagnostic biopsy not performed at recurrence	checkbox<br>1 bx_gtd_no_v2___1 Biopsy not technically feasible<br>2 bx_gtd_no_v2___2 Patient refused<br>3 bx_gtd_no_v2___3 Patient unable to afford<br>4 bx_gtd_no_v2___4 Resources not available locally<br>5 bx_gtd_no_v2___5 Other
2507	[ps_bx_gtd_no_v2]<br>Show the field ONLY if:<br>[bx_gtd_no_v2(5)] = '1'	Please state	text
2508	[gtd_mri_v2]	Magnetic resonance imaging (MRI) pelvis/abdomen-pelvis performed at recurrence diagnosis?	yesno<br>1 Yes<br>0 No

2509	[mri_find_gtd_v2] Show the field ONLY if: [gtd_mri_v2] = '1'	MRI pelvis/abdomen-pelvis findings at recurrence diagnosis (select all that apply)	checkbox<br>1 mri_find_gtd_v2___1 Post hysterectomy pelvic mass<br>2 mri_find_gtd_v2___2 x1 tubo-ovarian invasion<br>3 mri_find_gtd_v2___3 x2 tubo-ovarian invasion<br>4 mri_find_gtd_v2___4 Vaginal invasion<br>5 mri_find_gtd_v2___5 Urethral invasion<br>6 mri_find_gtd_v2___6 Bladder invasion<br>7 mri_find_gtd_v2___7 x1 hydroureter<br>8 mri_find_gtd_v2___8 x2 hydroureter<br>9 mri_find_gtd_v2___9 Ureteric invasion<br>10 mri_find_gtd_v2___10 x1 hydronephrosis<br>11 mri_find_gtd_v2___11 x2 hydronephrosis<br>12 mri_find_gtd_v2___12 Anal invasion<br>13 mri_find_gtd_v2___13 Rectal invasion<br>14 mri_find_gtd_v2___14 Sigmoid invasion<br>15 mri_find_gtd_v2___15 Pelvic bone invasion<br>16 mri_find_gtd_v2___16 Inguinal lymphadenopathy<br>17 mri_find_gtd_v2___17 Pelvic lymphadenopathy<br>18 mri_find_gtd_v2___18 Para-aortic lymphadenopathy<br>19 mri_find_gtd_v2___19 Other
2510	[ps_mri_find_gtd_v2] Show the field ONLY if: [mri_find_gtd_v2(19)] = '1'	Please state	text
2511	[mri_gtd_no_v2] Show the field ONLY if: [gtd_mri_v2] = '0'	Reason MRI pelvis not performed at recurrence diagnosis (Select all that apply)	checkbox<br>1 mri_gtd_no_v2___1 No access to MRI machine<br>2 mri_gtd_no_v2___2 Patient unable to afford<br>3 mri_gtd_no_v2___3 Patient refused<br>4 mri_gtd_no_v2___4 Claustrophobia<br>5 mri_gtd_no_v2___5 Other imaging performed<br>6 mri_gtd_no_v2___6 Extreme morbid obesity<br>7 mri_gtd_no_v2___7 Other
2512	[ps_mri_gtd_no_v2] Show the field ONLY if: [mri_gtd_no_v2(7)] = '1'	Please state	text
2513	[ctcap_gtd_v2]	Staging computerised tomography (CT) performed at recurrence diagnosis?	yesno<br>1 Yes<br>0 No

2514	[ctfind_gtd_v2]<br>Show the field ONLY if:<br>[ctcap_gtd_v2] = '1'	Staging CT findings at recurrence diagnosis (Select all that apply)	checkbox<br>1 ctfind_gtd_v2___1 Post hysterectomy pelvic mass<br>2 ctfind_gtd_v2___2 x1 tubo-ovarian invasion<br>3 ctfind_gtd_v2___3 x2 tubo-ovarian invasion<br>4 ctfind_gtd_v2___4 Vaginal invasion<br>5 ctfind_gtd_v2___5 Urethral invasion<br>6 ctfind_gtd_v2___6 Bladder invasion<br>7 ctfind_gtd_v2___7 x1 hydroureter<br>8 ctfind_gtd_v2___8 x2 hydroureter<br>9 ctfind_gtd_v2___9 Ureteric invasion<br>10 ctfind_gtd_v2___10 x1 hydronephrosis<br>11 ctfind_gtd_v2___11 x2 hydronephrosis<br>12 ctfind_gtd_v2___12 Anal invasion<br>13 ctfind_gtd_v2___13 Rectal invasion<br>14 ctfind_gtd_v2___14 Sigmoid invasion<br>15 ctfind_gtd_v2___15 Pelvic bone invasion<br>16 ctfind_gtd_v2___16 Inguinal lymphadenopathy<br>17 ctfind_gtd_v2___17 Pelvic lymphadenopathy<br>18 ctfind_gtd_v2___18 Para-aortic lymphadenopathy<br>19 ctfind_gtd_v2___19 Lung nodules<br>20 ctfind_gtd_v2___20 Pleural effusion<br>21 ctfind_gtd_v2___21 Other
2515	[ps_ctfind_gtd_v2]<br>Show the field ONLY if:<br>[ctfind_gtd_v2(21)] = '1'	Please state	text
2516	[ctcap_gtd_no_reas_v2]<br>Show the field ONLY if:<br>[ctcap_gtd_v2] = '0'	Reason staging CT not performed at recurrence diagnosis (state all that apply)	checkbox<br>1 ctcap_gtd_no_reas_v2___1 No access to CT machine<br>2 ctcap_gtd_no_reas_v2___2 Patient unable to afford<br>3 ctcap_gtd_no_reas_v2___3 Patient refused<br>4 ctcap_gtd_no_reas_v2___4 Other imaging performed<br>5 ctcap_gtd_no_reas_v2___5 Allergic reaction to contrast<br>6 ctcap_gtd_no_reas_v2___6 Chronic kidney disease<br>7 ctcap_gtd_no_reas_v2___7 Not clinical protocol<br>8 ctcap_gtd_no_reas_v2___8 Other
2517	[ps_ctcap_no_reas_gtd_v2]<br>Show the field ONLY if:<br>[ctcap_gtd_no_reas_v2(8)] = '1'	Please state	text
2518	[pet_gtd_v2]	PET-CT performed at diagnosis?	yesno<br>1 Yes<br>0 No

2519	[pet_find_gtd_v2]<br>Show the field ONLY if:<br>[pet_gtd_v2] = '1'	PET-CT findings at recurrence diagnosis (Select all that apply)	checkbox<br>1 pet_find_gtd_v2___1 Avid -FDG uptake ovary(s)<br>2 pet_find_gtd_v2___2 Avid -FDG uptake vagina<br>3 pet_find_gtd_v2___3 Avid -FDG uptake urethra<br>4 pet_find_gtd_v2___4 Avid -FDG uptake bladder<br>5 pet_find_gtd_v2___5 Avid -FDG uptake ureter(s)<br>6 pet_find_gtd_v2___6 Avid -FDG uptake kidney(s)<br>7 pet_find_gtd_v2___7 Avid -FDG uptake anus<br>8 pet_find_gtd_v2___8 Avid -FDG uptake rectum<br>9 pet_find_gtd_v2___9 Avid -FDG uptake sigmoid<br>10 pet_find_gtd_v2___10 Avid -FDG uptake para-rectal nodules<br>11 pet_find_gtd_v2___11 Avid -FDG uptake sigmoid nodules<br>12 pet_find_gtd_v2___12 Avid -FDG uptake pelvic bones<br>13 pet_find_gtd_v2___13 Avid -FDG uptake inguinal lymph nodes<br>14 pet_find_gtd_v2___14 Avid -FDG uptake pelvic lymph nodes<br>15 pet_find_gtd_v2___15 Avid -FDG uptake para-aortic lymph nodes<br>16 pet_find_gtd_v2___16 Avid -FDG uptake lung nodules<br>17 pet_find_gtd_v2___17 Other
2520	[ps_pet_fing_gtd_v2]<br>Show the field ONLY if:<br>[pet_find_gtd_v2(17)] = '1'	Please state	text
2521	[pet_no_reaas_gtd_v2]<br>Show the field ONLY if:<br>[pet_gtd_v2] = '0'	Reason PET-CT not performed (Select all that apply)	checkbox<br>1 pet_no_reaas_gtd_v2___1 No access to PET machine<br>2 pet_no_reaas_gtd_v2___2 Patient unable to afford<br>3 pet_no_reaas_gtd_v2___3 Patient refused<br>4 pet_no_reaas_gtd_v2___4 Uncontrolled diabetes mellitus<br>5 pet_no_reaas_gtd_v2___5 Other imaging performed<br>6 pet_no_reaas_gtd_v2___6 Not clinical protocol<br>7 pet_no_reaas_gtd_v2___7 Other
2522	[ps_pet_no_reaas_gtd_v2]<br>Show the field ONLY if:<br>[pet_no_reaas_gtd_v2(7)] = '1'	Please state	text
2523	[covid_gtd_v2]	COVID-19 status	dropdown<br>1 Positive<br>2 Negative<br>3 Not tested

2524	[covid_no_reas_gtd_v2]<br>Show the field ONLY if:<br>[covid_gtd_v2] = '3'	Reason not tested (Select all that apply)	checkbox<br>1 covid_no_reas_gtd_v2___1 Not clinically indicated (asymptomatic)<br>2 covid_no_reas_gtd_v2___2 Not clinical protocol to routinely test all patients prior to surgery<br>3 covid_no_reas_gtd_v2___3 Patient refused<br>4 covid_no_reas_gtd_v2___4 Patient unable to afford test<br>5 covid_no_reas_gtd_v2___5 No resources available locally for testing<br>6 covid_no_reas_gtd_v2___6 Other
2525	[ps_covid_no_reas_gtd_v2]<br>Show the field ONLY if:<br>[covid_no_reas_gtd_v2(6)] = '1'	Please state	text
2526	[bp_gtd2]	Bowel preparation pre-operatively	yesno<br>1 Yes<br>0 No
2527	[peri_op_gtd2]	Peri-operative management plan in place? (Non- exhaustive list: pre-operative haemoglobin optimisation and iron deficit correction; correction of denutrition and immunonutrition; fluid management; pain management; prevention of post-operative nausea and vomiting).	yesno<br>1 Yes<br>0 No
2528	[hb_gtd2]	Pre-operative Haemoglobin (g/L)	text (number)
2529	[wcc_gtd2]	Pre-operative White Cell Count (10^9/L)	text (number)
2530	[plt_gtd2]	Pre-operative Platelets (10^9/L)	text (number)
2531	[pt_gtd2]	Pre-operative PT (Secs)	text (number)
2532	[inr_gtd2]	Pre-operative INR (ratio)	text (number)
2533	[aptt_gtd2]	Pre-operative APTT (Secs)	text (number)
2534	[fib_gtd2]	Pre-operative Fibrinogen (g/L)	text (number)
2535	[alb_gtd2]	Pre-operative Albumin (g/L)	text (number)
2536	[na_gtd2]	Pre-operative Sodium (mmol/L)	text (number)
2537	[k_gtd2]	Pre-operative Potassium (mmol/L)	text (number)
2538	[ur_gtd2]	Pre-operative Urea (mmol/L)	text (number)
2539	[creat_gtd2]	Pre-operative Creatinine (µmol/L)	text (number)
2540	[egfr_gtd2]	Pre-operative eGFR - estimated glomerular filtration rate (mL/min)	text
2541	[bili_gtd2]	Pre-operative Bilirubin total (µmol/L)	text (number)
2542	[aat_gtd2]	Pre-operative Alanine Aminotransaminase (IU/L)	text (number)
2543	[alp_gtd2]	Pre-operative Alkaline Phosphatase (IU/L)	text (number)
2544	[ast_gtd2]	Pre-operative Aspartate Aminotransaminase (IU/L)	text (number)
2545	[crp_gtd2]	Pre-operative CRP - C-reactive protein (mg/L)	text (number)
2546	[dat_gtd_v2]	Section Header: *SURGERY*<br>Date of recurrence surgery	text (date_dmy)
2547	[anaes_gtd_v2]	Anaesthesia (Select all that apply)	checkbox<br>1 anaes_gtd_v2___1 General<br>2 anaes_gtd_v2___2 Spinal<br>3 anaes_gtd_v2___3 Epidural
2548	[elecem_gtd_v2]	Was recurrence surgery an elective or emergency case?	radio<br>1 Elective<br>2 Emergency

2549	[sa_gtd_v2]	Surgical aim	dropdown<br>1 Curative<br>2 Curative attempted but then abandoned (i.e. open/close laparotomy)<br>3 Palliative
2550	[sm_gtd_v2]	Surgical modality	dropdown<br>1 Laparotomy<br>2 Laparoscopic<br>3 Robotic<br>6 Vaginal<br>4 Laparoscopic converted to laparotomy<br>5 Robotic converted to laparotomy<br>7 Vaginal converted to laparotomy
2551	[ls_gtd_v2]	Lead surgeon performing recurrence surgery	dropdown<br>1 Certified consultant/specialist gynaecological oncologist<br>2 Fellow/trainee gynaecological oncologist<br>3 Trained surgeon dedicated to the management of gynaecologic cancer or having completed an ESGO accredited fellowship<br>4 General surgeon<br>5 Surgical oncologist<br>6 Other
2552	[ps_ls_gtd_v2]<br>Show the field ONLY if:<br>[ls_gtd_v2] = '6'	Please state	text
2553	[who_gtd2]	WHO surgical safety checklist used	yesno<br>1 Yes<br>0 No
2554	[cyto_gtd2]	Complete macroscopic cytoreduction at recurrence surgery	yesno<br>1 Yes<br>0 No
2555	[reas_cytored_no_gtd2]<br>Show the field ONLY if:<br>[cyto_gtd2] = '0'	Reason(s) disease not fully excised at recurrence cytoreduction surgery (select all that apply)	checkbox<br>1 reas_cytored_no_gtd2___1 Disease more extensive than that shown on imaging<br>2 reas_cytored_no_gtd2___2 Technically unable to resect<br>3 reas_cytored_no_gtd2___3 Patient became medically unstable intra-operatively and decision made to not proceed further with surgery<br>4 reas_cytored_no_gtd2___4 Other
2556	[ps_reas_cytored_no_gtd2]<br>Show the field ONLY if:<br>[reas_cytored_no_gtd2(4)] = '1'	Please state	text
2557	[siz_rec_gtd2]<br>Show the field ONLY if:<br>[cyto_gtd2] = '0'	Size of recurrence disease (in millimetres)	dropdown<br>1 1-2.5<br>2 2.6-5<br>3 5.1-7.5<br>4 7.6-10<br>5 >10

2558	[loc_resid_gtd2]<br>Show the field ONLY if:<br>[cyto_gtd2] = '0'	Location of residual disease at recurrence surgery (Select all that apply)	checkbox<br>1 loc_resid_gtd2___1 ovary(s)<br>2 loc_resid_gtd2___2 Vagina<br>3 loc_resid_gtd2___3 Urethra<br>4 loc_resid_gtd2___4 Bladder<br>5 loc_resid_gtd2___5 ureter<br>6 loc_resid_gtd2___6 Kidney(s)<br>7 loc_resid_gtd2___7 Anus<br>8 loc_resid_gtd2___8 Rectum<br>9 loc_resid_gtd2___9 Sigmoid<br>10 loc_resid_gtd2___10 Para rectal nodules<br>11 loc_resid_gtd2___11 Sigmoid nodules<br>12 loc_resid_gtd2___12 Pelvic bone<br>13 loc_resid_gtd2___13 Inguinal lymphadenopathy<br>14 loc_resid_gtd2___14 Pelvic lymphadenopathy<br>15 loc_resid_gtd2___15 Para-aortic lymphadenopathy<br>16 loc_resid_gtd2___16 Other
2559	[ps_loc_resid_gtd2]<br>Show the field ONLY if:<br>[loc_resid_gtd2(16)] = '1'	Please state	text
2560	[gtd_surgfind_v2]	Findings at recurrence surgery (Select all that apply)	checkbox<br>1 gtd_surgfind_v2___1 Post hysterectomy pelvic mass<br>2 gtd_surgfind_v2___2 x1 tubo-ovarian invasion<br>3 gtd_surgfind_v2___3 x2 tubo-ovarian invasion<br>4 gtd_surgfind_v2___4 Vaginal invasion<br>5 gtd_surgfind_v2___5 Urethral invasion<br>6 gtd_surgfind_v2___6 Bladder invasion<br>7 gtd_surgfind_v2___7 x1 hydroureter<br>8 gtd_surgfind_v2___8 x2 hydroureter<br>9 gtd_surgfind_v2___9 Ureteric invasion<br>10 gtd_surgfind_v2___10 x1 hydronephrosis<br>11 gtd_surgfind_v2___11 x2 hydronephrosis<br>12 gtd_surgfind_v2___12 Anal invasion<br>13 gtd_surgfind_v2___13 Rectal invasion<br>14 gtd_surgfind_v2___14 Sigmoid invasion<br>15 gtd_surgfind_v2___15 Para rectal nodules<br>16 gtd_surgfind_v2___16 Sigmoid nodules<br>17 gtd_surgfind_v2___17 Pelvic bone invasion<br>18 gtd_surgfind_v2___18 Inguinal lymphadenopathy<br>19 gtd_surgfind_v2___19 Pelvic lymphadenopathy<br>20 gtd_surgfind_v2___20 Para-aortic lymphadenopathy<br>21 gtd_surgfind_v2___21 Other<br>22 gtd_surgfind_v2___22 No visible disease
2561	[ps_gtd_surgfind_v2]<br>Show the field ONLY if:<br>[gtd_surgfind_v2(21)] = '1'	Please state	text

2562	[tis_surg_gtd_v2]	Tissue removed at recurrence surgery (Select all that apply)	checkbox<br>1 tis_surg_gtd_v2___1 x1 ovary<br>2 tis_surg_gtd_v2___2 x2 ovaries<br>3 tis_surg_gtd_v2___3 Vagina<br>4 tis_surg_gtd_v2___4 Urethra<br>5 tis_surg_gtd_v2___5 ureter<br>6 tis_surg_gtd_v2___6 kidney<br>7 tis_surg_gtd_v2___7 Anus<br>8 tis_surg_gtd_v2___8 Rectum<br>9 tis_surg_gtd_v2___9 Sigmoid<br>10 tis_surg_gtd_v2___10 Inguinal nodes<br>11 tis_surg_gtd_v2___11 Pelvic lymph nodes<br>12 tis_surg_gtd_v2___12 Para-aortic nodes<br>13 tis_surg_gtd_v2___13 Other
2563	[paln_dis_gtd2]<br>Show the field ONLY if:<br>[tis_surg_gtd_v2(12)] = '1'	Select all that apply	checkbox<br>1 paln_dis_gtd2___1 Infra-renal<br>2 paln_dis_gtd2___2 Supra-renal
2564	[ps_tis_surg_gtd_v2]<br>Show the field ONLY if:<br>[tis_surg_gtd_v2(13)] = '1'	Please state	text
2565	[si_stom_gtd_v2]	Small bowel stoma	yesno<br>1 Yes<br>0 No
2566	[ss_gtd2]<br>Show the field ONLY if:<br>[si_stom_gtd_v2] = '1'	Stoma site	dropdown<br>1 Duodenum<br>2 Jejunum<br>3 Ileum
2567	[pb_si_stom_gtd_v2]<br>Show the field ONLY if:<br>[si_stom_gtd_v2] = '1'	Performed by (select all that apply)	checkbox<br>1 pb_si_stom_gtd_v2___1 Gynaecological oncologist<br>2 pb_si_stom_gtd_v2___2 Colorectal surgeon<br>3 pb_si_stom_gtd_v2___3 Urologist<br>4 pb_si_stom_gtd_v2___4 General surgeon<br>5 pb_si_stom_gtd_v2___5 Plastic surgeon<br>6 pb_si_stom_gtd_v2___6 Other
2568	[ps_pb_si_stom_gtd_v2]<br>Show the field ONLY if:<br>[pb_si_stom_gtd_v2(6)] = '1'	Please state	text
2569	[si_anast_gtd_v2]	Small bowel anastamosis	yesno<br>1 Yes<br>0 No
2570	[pb_si_anast_gtd_v2]<br>Show the field ONLY if:<br>[si_anast_gtd_v2] = '1'	Performed by (select all that apply)	checkbox<br>1 pb_si_anast_gtd_v2___1 Gynaecological oncologist<br>2 pb_si_anast_gtd_v2___2 Colorectal surgeon<br>3 pb_si_anast_gtd_v2___3 Urologist<br>4 pb_si_anast_gtd_v2___4 General surgeon<br>5 pb_si_anast_gtd_v2___5 Plastic surgeon<br>6 pb_si_anast_gtd_v2___6 Other
2571	[ps_pb_si_anast_gtd_v2]<br>Show the field ONLY if:<br>[pb_si_anast_gtd_v2(6)] = '1'	Please state	text

2572	[li_stoma_gtd_v2]	Large bowel stoma	yesno<br>1 Yes<br>0 No
2573	[pb_li_stom_gtd_v2]<br>Show the field ONLY if:<br>[li_stoma_gtd_v2] = '1'	Performed by (select all that apply)	checkbox<br>1 pb_li_stom_gtd_v2___1 Gynaecological oncologist<br>2 pb_li_stom_gtd_v2___2 Colorectal surgeon<br>3 pb_li_stom_gtd_v2___3 Urologist<br>4 pb_li_stom_gtd_v2___4 General surgeon<br>5 pb_li_stom_gtd_v2___5 Plastic surgeon<br>6 pb_li_stom_gtd_v2___6 Other
2574	[ps_pb_li_stom_gtd_v2]<br>Show the field ONLY if:<br>[pb_li_stom_gtd_v2(6)] = '1'	Please state	text
2575	[li_anast_gtd_v2]	Large bowel anastamosis	yesno<br>1 Yes<br>0 No
2576	[pb_li_anast_gtd_v2]<br>Show the field ONLY if:<br>[li_anast_gtd_v2] = '1'	Performed by (select all that apply)	checkbox<br>1 pb_li_anast_gtd_v2___1 Gynaecological oncologist<br>2 pb_li_anast_gtd_v2___2 Colorectal surgeon<br>3 pb_li_anast_gtd_v2___3 Urologist<br>4 pb_li_anast_gtd_v2___4 General surgeon<br>5 pb_li_anast_gtd_v2___5 Plastic surgeon<br>6 pb_li_anast_gtd_v2___6 Other
2577	[ps_pb_li_anast_gtd_v2]<br>Show the field ONLY if:<br>[pb_li_anast_gtd_v2(6)] = '1'	Please state	text
2578	[ui_gtd_v2]	Urostomy/ileal conduit	yesno<br>1 Yes<br>0 No
2579	[pb_ui_gtd_v2]<br>Show the field ONLY if:<br>[ui_gtd_v2] = '1'	Performed by (select all that apply)	checkbox<br>1 pb_ui_gtd_v2___1 Gynaecological oncologist<br>2 pb_ui_gtd_v2___2 Colorectal surgeon<br>3 pb_ui_gtd_v2___3 Urologist<br>4 pb_ui_gtd_v2___4 General surgeon<br>5 pb_ui_gtd_v2___5 Plastic surgeon<br>6 pb_ui_gtd_v2___6 Other
2580	[ps_pb_ui_gtd_v2]<br>Show the field ONLY if:<br>[pb_ui_gtd_v2(6)] = '1'	Please state	text
2581	[tuu_gtd_v2]	Trans uretero-ureteric anastamosis	yesno<br>1 Yes<br>0 No
2582	[pb_tuu_gtd_v2]<br>Show the field ONLY if:<br>[tuu_gtd_v2] = '1'	Performed by (select all that apply)	checkbox<br>1 pb_tuu_gtd_v2___1 Gynaecological oncologist<br>2 pb_tuu_gtd_v2___2 Colorectal surgeon<br>3 pb_tuu_gtd_v2___3 Urologist<br>4 pb_tuu_gtd_v2___4 General surgeon<br>5 pb_tuu_gtd_v2___5 Plastic surgeon<br>6 pb_tuu_gtd_v2___6 Other
2583	[ps_pb_tuu_gtd_v2]<br>Show the field ONLY if:<br>[pb_tuu_gtd_v2(6)] = '1'	Please state	text

2584	[neph_gtd_v2]	Nephrostomy	yesno<br>1 Yes<br>0 No
2585	[surgeon_gtd_add_v2]<br>Show the field ONLY if:<br>[neph_gtd_v2] = '1'	Performed by (Select all that apply)	checkbox<br>1 surgeon_gtd_add_v2___1 Gynaecological oncologist<br>2 surgeon_gtd_add_v2___2 Colorectal surgeon<br>3 surgeon_gtd_add_v2___3 Urologist<br>4 surgeon_gtd_add_v2___4 General surgeon<br>5 surgeon_gtd_add_v2___5 Plastic surgeon<br>6 surgeon_gtd_add_v2___6 Other
2586	[ps_surgeon_gtd_add_v2]<br>Show the field ONLY if:<br>[surgeon_gtd_add_v2(6)] = '1'	Please state	text
2587	[abx_gtd2]	Intraoperative antibiotics	yesno<br>1 Yes<br>0 No
2588	[tim_gtd_v2]	Length of recurrence surgery	text (time)
2589	[itu_gtd2]	Section Header: *POST-OPERATIVE*<br>Post-operative recovery in intensive care unit	yesno<br>1 Yes<br>0 No
2590	[itu_dys_gtd2]<br>Show the field ONLY if:<br>[itu_gtd2] = '1'	Number of days in intensive care unit	text (number)
2591	[hdu_gtd2]	Post-operative recovery in high dependency unit	yesno<br>1 Yes<br>0 No
2592	[hdu_dys_gtd2]<br>Show the field ONLY if:<br>[hdu_gtd2] = '1'	Number of days in high dependency unit	text (number)
2593	[er_gtd_v2]	Enhanced recovery?	yesno<br>1 Yes<br>0 No
2594	[po_abx_gtd3]	Prophylactic post operative antibiotics	yesno<br>1 Yes<br>0 No
2595	[drn_gtd2]	Post operative surgical drain	yesno<br>1 Yes<br>0 No
2596	[drn_dys_gtd2]<br>Show the field ONLY if:<br>[drn_gtd2] = '1'	Number of days in situ	text (number)
2597	[uc_gtd2]	Post operative urinary catheter	yesno<br>1 Yes<br>0 No
2598	[cath_dys_gtd2]<br>Show the field ONLY if:<br>[uc_gtd2] = '1'	Number of days in situ	text (number)
2599	[phb_gtd_2]	Post-operative Haemoglobin (g/L) - please state first post-operative result	text (number)
2600	[pwbc_gtd_2]	Post-operative White Cell Count (10^9/L) - please state first post-operative result	text (number)
2601	[pplat_gtd_2]	Post-operative Platelets (10^9/L) - please state first post-operative result	text (number)
2602	[ppt_gtd_2]	Post-operative PT (Secs) - please state first post-operative result	text (number)

2603	[pinr_gtd_2]	Post-operative INR (Ratio) - please state first post-operative result	text (number)
2604	[paptt_gtd_2]	Post-operative APTT (Secs) - please state first post-operative result	text (number)
2605	[pfib_gtd_2]	Post-operative Fibrinogen (g/L) - please state first post-operative result	text (number)
2606	[palb_gtd_2]	Post-operative Albumin (g/L) - please state first post-operative result	text (number)
2607	[pna_gtd_2]	Post-operative Sodium (mmol/L) - please state first post-operative result	text (number)
2608	[pk_gtd_2]	Post-operative Potassium (mmol/L) - please state first post-operative result	text (number)
2609	[purea_gtd_2]	Post-operative Urea (mmol/L) - please state first post-operative result	text (number)
2610	[pcreat_gtd_2]	Post-operative Creatinine (μmol/L) - please state first post-operative result	text
2611	[pegfr_gtd_2]	Post-operative eGFR - Estimated Glomerular Filtration Rate (mL/min) - please state first post-operative result	text
2612	[pbili_gtd_2]	Post-operative Bilirubin total (μmol/L) - please state first post-operative result	text (number)
2613	[paat_gtd_2]	Post-operative Alanine Aminotransaminase (IU/L) - please state first post-operative result	text (number)
2614	[palp_gtd_2]	Post-operative Alkaline Phosphatase (IU/L) - please state first post-operative result	text (number)
2615	[past_gtd2]	Post-operative Aspartate Aminotransaminase (IU/L) - please state first post-operative result	text (number)
2616	[pcrp_gtd_2]	Post-operative CRP - C-reactive protein (mg/L) - please state first post-operative result	text (number)
2617	[histo_gtd_v2]	Histopathology	dropdown<br>2 Choriocarcinoma<br>3 Placenta site trophoblastic tumour<br>4 Epithelioid trophoblastic tumour
2618	[resec_gtd_v2]	Histological confirmed resection margins	dropdown<br>1 < 1 mm / tumour on margin<br>2 1-5 mm (NO tumour on margin)<br>3 >5 mm Margins confirmed clear<br>4 Unknown, not available in this hospital
2619	[ckae1_gtd_v2]	Section Header: *Immunohistochemistry*<br>CK AE1	radio (Matrix)<br>1 Positive<br>2 Negative<br>3 Not tested
2620	[ckae3_gtd_v2]	CK AE3	radio (Matrix)<br>1 Positive<br>2 Negative<br>3 Not tested
2621	[p63_gtd_v2]	p63	radio (Matrix)<br>1 Positive<br>2 Negative<br>3 Not tested
2622	[hpl_gtd_v2]	hPL	radio (Matrix)<br>1 Positive<br>2 Negative<br>3 Not tested
2623	[melcam_gtd_v2]	Mel-CAM	radio (Matrix)<br>1 Positive<br>2 Negative<br>3 Not tested

2624	[ki67_gtd_v2]	Ki-67	radio (Matrix)<br>1 Positive<br>2 Negative<br>3 Not tested
2625	[adjchem_gtd_v2]	Chemotherapy following recurrence diagnosis	yesno<br>1 Yes<br>0 No
2626	[adjchem1_gtd_v2]<br>Show the field ONLY if:<br>[adjchem_gtd_v2] = '1'	Chemotherapy regimen (select all that apply)	checkbox<br>1 adjchem1_gtd_v2___1 Methotrexate<br>2 adjchem1_gtd_v2___2 Actinomycin D/dactinomycin<br>3 adjchem1_gtd_v2___3 Cyclophosphamide<br>4 adjchem1_gtd_v2___4 Vinca alkaloids (e.g. vincristine)<br>5 adjchem1_gtd_v2___5 Etoposide<br>6 adjchem1_gtd_v2___6 Other
2627	[ps_adjchem1_gtd_v2]<br>Show the field ONLY if:<br>[adjchem1_gtd_v2(6)] = '1'	Please state	text
2628	[adjchem_cycl_gtd_v2]<br>Show the field ONLY if:<br>[adjchem_gtd_v2] = '1'	Number of cycles planned	text (number)
2629	[adjchem_no_reas_gtd_v2]<br>Show the field ONLY if:<br>[adjchem_gtd_v2] = '0'	Reason chemotherapy not administered following recurrence diagnosis (Select all that apply)	checkbox<br>1 adjchem_no_reas_gtd_v2___1 Poor performance status<br>2 adjchem_no_reas_gtd_v2___2 No access<br>3 adjchem_no_reas_gtd_v2___3 Patient unable to afford<br>4 adjchem_no_reas_gtd_v2___4 Patient refused<br>5 adjchem_no_reas_gtd_v2___5 Not clinically indicated<br>6 adjchem_no_reas_gtd_v2___6 Other
2630	[ps_adjchem_no_reas_gtd_v2]<br>Show the field ONLY if:<br>[adjchem_no_reas_gtd_v2(6)] = '1'	Please state	text
2631	[rt_gtd_v2]	External beam radiotherapy administered following recurrence diagnosis	yesno<br>1 Yes<br>0 No
2632	[dos_rt_gtd2]<br>Show the field ONLY if:<br>[rt_gtd_v2] = '1'	Dose received (Gray - Gy)	text (number)
2633	[rt_no_reas_gtd_v2]<br>Show the field ONLY if:<br>[rt_gtd_v2] = '0'	Reason external beam radiotherapy not administered following recurrence diagnosis (select all that apply)	checkbox<br>1 rt_no_reas_gtd_v2___1 Poor performance status<br>2 rt_no_reas_gtd_v2___2 No access<br>3 rt_no_reas_gtd_v2___3 Patient unable to afford<br>4 rt_no_reas_gtd_v2___4 Patient refused<br>5 rt_no_reas_gtd_v2___5 Not clinically indicated<br>6 rt_no_reas_gtd_v2___6 Other
2634	[ps_rt_no_reas_gtd_v2]<br>Show the field ONLY if:<br>[rt_no_reas_gtd_v2(6)] = '1'	Please state	text

2635	[brachy_gtd_v2]	Brachytherapy administered following recurrence diagnosis	yesno<br>1 Yes<br>0 No
2636	[dos_rt_gtd3]<br>Show the field ONLY if:<br>[brachy_gtd_v2] = '1'	Dose received (Gray - Gy)	text (number)
2637	[brachy_no_reas_gtd_v2]<br>Show the field ONLY if:<br>[brachy_gtd_v2] = '0'	Reason brachytherapy not administered following recurrence diagnosis (select all that apply)	checkbox<br>1 brachy_no_reas_gtd_v2___1 Poor performance status<br>2 brachy_no_reas_gtd_v2___2 No access<br>3 brachy_no_reas_gtd_v2___3 Patient unable to afford<br>4 brachy_no_reas_gtd_v2___4 Patient refused<br>5 brachy_no_reas_gtd_v2___5 Not clinically indicated<br>6 brachy_no_reas_gtd_v2___6 Other
2638	[ps_brachy_no_reas_gtd_v2]<br>Show the field ONLY if:<br>[brachy_no_reas_gtd_v2(6)] = '1'	Please state	text
2639	[malignant_gestational_trophoblastic_disease_recurr_complete]	Section Header: *Form Status*<br>Complete?	dropdown<br>0 Incomplete<br>1 Unverified<br>2 Complete

Instrument: **Minor/day case surgical procedures** (minorday_case_surgical_procedures)

2640	[mnr_surg]	Surgical procedure	dropdown<br>1 Prophylactic/risk reducing salpingo-oophorectomy<br>2 Prophylactic/risk reducing salpingectomy<br>3 Hysteroscopy<br>4 Examination under anaesthesia +/- biopsy
2641	[mnr_surg_mod]<br>Show the field ONLY if:<br>[mnr_surg] = '1' and [mnr_surg] = '2'	Surgical modality	dropdown<br>1 Laparoscopic<br>2 Robotic<br>3 Laparoscopy converted to open<br>4 Robotic converted to open
2642	[hyst_mod]<br>Show the field ONLY if:<br>[mnr_surg] = '3'	Setting	radio<br>1 general anaesthesia<br>2 outpatient
2643	[sd_disch]	Same day discharge	yesno<br>1 Yes<br>0 No
2644	[minorday_case_surgical_procedures_complete]	Section Header: *Form Status*<br>Complete?	dropdown<br>0 Incomplete<br>1 Unverified<br>2 Complete

Instrument: **Intra-operative complications** (intraoperative_complications)

2645	[iop]	Intra-operative complications (select all that apply)	checkbox<br>1 iop___1 Significant intra-opertative blood loss requiring blood transfusion<br>2 iop___2 Ureter injury<br>3 iop___3 Bladder injury<br>4 iop___4 Large bowel injury<br>5 iop___5 Small bowel injury<br>6 iop___6 Liver injury<br>7 iop___7 Splenic injury<br>8 iop___8 Pancreatic injury<br>9 iop___9 Gastric injury<br>10 iop___10 Vascular injury<br>11 iop___11 Nerve injury<br>12 iop___12 Death<br>13 iop___13 Other<br>14 iop___14 None
2646	[bld_prod]<br>Show the field ONLY if:<br>[iop(1)] = '1'	Blood products transfused (select all that apply)	checkbox<br>1 bld_prod___1 red cell concentrate<br>2 bld_prod___2 platelet concentrate<br>3 bld_prod___3 fresh frozen plasma<br>4 bld_prod___4 cryopercipitate<br>5 bld_prod___5 whole blood
2647	[numb_unit]<br>Show the field ONLY if:<br>[bld_prod(1)] = '1'	Number of units	text (number)
2648	[numb_unit_2]<br>Show the field ONLY if:<br>[bld_prod(2)] = '1'	Number of units	text (number)
2649	[numb_unit_3]<br>Show the field ONLY if:<br>[bld_prod(3)] = '1'	Number of units	text (number)
2650	[numb_unit_4]<br>Show the field ONLY if:<br>[bld_prod(4)] = '1'	Number of units	text (number)
2651	[numb_unit_5]<br>Show the field ONLY if:<br>[bld_prod(5)] = '1'	Number of units	text (number)
2652	[ebl_iop]	State intraoperative blood loss (millilitres/mls)	text (number), Required
2653	[li_comp]<br>Show the field ONLY if:<br>[iop(4)] = '1'	Large bowel injury (select all that apply)	checkbox<br>1 li_comp___1 Caecum<br>2 li_comp___2 Ascending colon<br>3 li_comp___3 Transverse colon<br>4 li_comp___4 Descending colon<br>5 li_comp___5 Sigmoid colon<br>6 li_comp___6 Rectum
2654	[typ_inj_io_1]<br>Show the field ONLY if:<br>[li_comp(1)] = '1'	Type of injury (select all that apply)	checkbox<br>1 typ_inj_io_1___1 serosal<br>2 typ_inj_io_1___2 full thickness<br>3 typ_inj_io_1___3 thermal
2655	[typ_inj_io_2]<br>Show the field ONLY if:<br>[li_comp(2)] = '1'	Type of injury (select all that apply)	checkbox<br>1 typ_inj_io_2___1 serosal<br>2 typ_inj_io_2___2 full thickness<br>3 typ_inj_io_2___3 thermal

2656	[typ_inj_io_3]<br>Show the field ONLY if:<br>[li_comp(3)] = '1'	Type of injury (select all that apply)	checkbox<br>1 typ_inj_io_3___1 serosal<br>2 typ_inj_io_3___2 full thickness<br>3 typ_inj_io_3___3 thermal
2657	[typ_inj_io_4]<br>Show the field ONLY if:<br>[li_comp(4)] = '1'	Type of injury (select all that apply)	checkbox<br>1 typ_inj_io_4___1 serosal<br>2 typ_inj_io_4___2 full thickness<br>3 typ_inj_io_4___3 thermal
2658	[typ_inj_io_5]<br>Show the field ONLY if:<br>[li_comp(5)] = '1'	Type of injury (select all that apply)	checkbox<br>1 typ_inj_io_5___1 serosal<br>2 typ_inj_io_5___2 full thickness<br>3 typ_inj_io_5___3 thermal
2659	[typ_inj_io_6]<br>Show the field ONLY if:<br>[li_comp(6)] = '1'	Type of injury (select all that apply)	checkbox<br>1 typ_inj_io_6___1 serosal<br>2 typ_inj_io_6___2 full thickness<br>3 typ_inj_io_6___3 thermal
2660	[rpr_li_io_1]<br>Show the field ONLY if:<br>[li_comp(1)] = '1'	Repair	dropdown<br>1 none<br>2 primary repair<br>3 segmental resection<br>4 colostomy
2661	[rpr_li_io_2]<br>Show the field ONLY if:<br>[li_comp(2)] = '1'	Repair	dropdown<br>1 none<br>2 primary repair<br>3 segmental resection<br>4 colostomy
2662	[rpr_li_io_3]<br>Show the field ONLY if:<br>[li_comp(3)] = '1'	Repair	dropdown<br>1 none<br>2 primary repair<br>3 segmental resection<br>4 colostomy
2663	[rpr_li_io_4]<br>Show the field ONLY if:<br>[li_comp(4)] = '1'	Repair	dropdown<br>1 none<br>2 primary repair<br>3 segmental resection<br>4 colostomy
2664	[rpr_li_io_5]<br>Show the field ONLY if:<br>[li_comp(5)] = '1'	Repair	dropdown<br>1 none<br>2 primary repair<br>3 segmental resection<br>4 colostomy
2665	[rpr_li_io_6]<br>Show the field ONLY if:<br>[li_comp(6)] = '1'	Repair	dropdown<br>1 none<br>2 primary repair<br>3 segmental resection<br>4 colostomy
2666	[si_inj_comp]<br>Show the field ONLY if:<br>[iop(5)] = '1'	Small bowel injury (select all that apply)	checkbox<br>1 si_inj_comp___1 Duodenum<br>2 si_inj_comp___2 Jejunum<br>3 si_inj_comp___3 Ileum

2667	[si_inj_typ_io_1]<br>Show the field ONLY if:<br>[si_inj_comp(1)] = '1'	Type of injury (select all that apply)	checkbox<br>1 si_inj_typ_io_1___1 serosal<br>2 si_inj_typ_io_1___2 full thickness<br>3 si_inj_typ_io_1___3 thermal
2668	[si_inj_typ_io_2]<br>Show the field ONLY if:<br>[si_inj_comp(2)] = '1'	Type of injury (select all that apply)	checkbox<br>1 si_inj_typ_io_2___1 serosal<br>2 si_inj_typ_io_2___2 full thickness<br>3 si_inj_typ_io_2___3 thermal
2669	[si_inj_typ_io_3]<br>Show the field ONLY if:<br>[si_inj_comp(3)] = '1'	Type of injury (select all that apply)	checkbox<br>1 si_inj_typ_io_3___1 serosal<br>2 si_inj_typ_io_3___2 full thickness<br>3 si_inj_typ_io_3___3 thermal
2670	[ps_iop]<br>Show the field ONLY if:<br>[iop(13)] = '1'	Please state	text
2671	[rpr_si_io_1]<br>Show the field ONLY if:<br>[si_inj_comp(1)] = '1'	Repair	dropdown<br>1 none<br>2 primary repair<br>3 segmental resection<br>4 stoma
2672	[rpr_si_io_2]<br>Show the field ONLY if:<br>[si_inj_comp(2)] = '1'	Repair	dropdown<br>1 none<br>2 primary repair<br>3 segmental resection<br>4 stoma
2673	[rpr_si_io_3]<br>Show the field ONLY if:<br>[si_inj_comp(3)] = '1'	Repair	dropdown<br>1 none<br>2 primary repair<br>3 segmental resection<br>4 stoma

2674	[io_comp_vess]<br>Show the field ONLY if:<br>[iop(10)] = '1'	Blood vessels injured (select all that apply)	checkbox<br>1 io_comp_vess___1 Aorta<br>2 io_comp_vess___2 Common iliac artery<br>3 io_comp_vess___3 Internal iliac artery<br>4 io_comp_vess___4 External iliac artery<br>5 io_comp_vess___5 Femoral artery<br>6 io_comp_vess___6 Obturator artery<br>7 io_comp_vess___7 Umbilical artery<br>8 io_comp_vess___8 Vaginal artery<br>9 io_comp_vess___9 Uterine artery<br>10 io_comp_vess___10 Ovarian artery<br>11 io_comp_vess___11 Internal pudendal artery<br>12 io_comp_vess___12 Inferior gluteal artery<br>13 io_comp_vess___13 Superior gluteal artery<br>14 io_comp_vess___14 Superior sacral artery<br>15 io_comp_vess___15 Inferior sacral artery<br>16 io_comp_vess___16 Median sacral artery<br>17 io_comp_vess___17 Iliolumbar artery<br>18 io_comp_vess___18 Superior vesical artery<br>19 io_comp_vess___19 Inferior vesical artery<br>20 io_comp_vess___20 Superior mesenteric artery<br>21 io_comp_vess___21 Inferior mesenteric artery<br>22 io_comp_vess___22 Sigmoid artery<br>23 io_comp_vess___23 Superior rectal artery<br>24 io_comp_vess___24 Inferior rectal artery<br>25 io_comp_vess___25 Middle rectal artery<br>26 io_comp_vess___26 Hepatic artery<br>27 io_comp_vess___27 Inferior vena cava<br>28 io_comp_vess___28 Common iliac vein<br>29 io_comp_vess___29 External iliac vein<br>30 io_comp_vess___30 Internal iliac vein<br>31 io_comp_vess___31 Femoral vein<br>32 io_comp_vess___32 Ovarian vein<br>33 io_comp_vess___33 Superior mesenteric vein<br>34 io_comp_vess___34 Inferior mesenteric vein<br>35 io_comp_vess___35 Sigmoid vein<br>36 io_comp_vess___36 Splenic vein<br>37 io_comp_vess___37 Hepatic vein<br>38 io_comp_vess___38 Hepatic portal vein<br>39 io_comp_vess___39 Other
2675	[ps_io_comp_vess]<br>Show the field ONLY if:<br>[io_comp_vess(39)] = '1'	Please state	text
2676	[io_comp_nerv]<br>Show the field ONLY if:<br>[iop(11)] = '1'	Nerves injured (select all that apply)	checkbox<br>1 io_comp_nerv___1 Iliohypogastric nerve<br>2 io_comp_nerv___2 Ilioinguinal nerve<br>3 io_comp_nerv___3 Genitofemoral nerve<br>4 io_comp_nerv___4 Obturator nerve<br>5 io_comp_nerv___5 Femoral nerve<br>6 io_comp_nerv___6 Other
2677	[ps_ios_comp_nerve]<br>Show the field ONLY if:<br>[io_comp_nerv(6)] = '1'	Please state	text

2678	[intraoperative_complications_complete]	Section Header: *Form Status*<br>Complete?	dropdown<br>0 Incomplete<br>1 Unverified<br>2 Complete

Instrument: **Grade I Clavien-Dindo post-operative surgical complications** (grade_i_claviendindo_postoperative_surgical_compli)

2679	[g1cd]	Grade I Clavien-Dindo post-operative complications	checkbox<br>1 g1cd___1 Wound infection managed conservatively (antibiotics, wounds opened at bedside)<br>2 g1cd___2 Atrial fibrillation which resolves after correction of electrolytes<br>3 g1cd___3 Atelectasis requiring physiotherapy<br>4 g1cd___4 Transient confusion not requiring therapy<br>5 g1cd___5 Non-infectious diarrhoea<br>6 g1cd___6 Transient elevation of serum creatinine<br>7 g1cd___7 Lymphoedema<br>8 g1cd___8 Ileus requiring intravenous fluids<br>9 g1cd___9 Urinary retention requiring catheterisation<br>10 g1cd___10 High output stoma managed with intravenous fluids for electrolyte replacement<br>11 g1cd___11 Chyloascites managed with diet modifications<br>12 g1cd___12 Other<br>13 g1cd___13 None
2680	[dat_wound]<br>Show the field ONLY if:<br>[g1cd(1)] = '1'	Date of complication	text (date_dmy)
2681	[dat_af]<br>Show the field ONLY if:<br>[g1cd(2)] = '1'	Date of complication	text (date_dmy)
2682	[dat_atelec]<br>Show the field ONLY if:<br>[g1cd(3)] = '1'	Date of complication	text (date_dmy)
2683	[dat_conf]<br>Show the field ONLY if:<br>[g1cd(4)] = '1'	Date of complication	text (date_dmy)
2684	[dat_diar]<br>Show the field ONLY if:<br>[g1cd(5)] = '1'	Date of complication	text (date_dmy)
2685	[dat_creat]<br>Show the field ONLY if:<br>[g1cd(6)] = '1'	Date of complication	text (date_dmy)
2686	[dat_lymph]<br>Show the field ONLY if:<br>[g1cd(7)] = '1'	Date of complication	text (date_dmy)
2687	[dat_il]<br>Show the field ONLY if:<br>[g1cd(8)] = '1'	Date of complication	text (date_dmy)
2688	[dat_urinret]<br>Show the field ONLY if:<br>[g1cd(9)] = '1'	Date of complication	text (date_dmy)
2689	[dat_hos]<br>Show the field ONLY if:<br>[g1cd(10)] = '1'	Date of complication	text (date_dmy)

2690	[dat_ca]<br>Show the field ONLY if:<br>[g1cd(11)] = '1'	Date of complication	text (date_dmy)
2691	[dat_other]<br>Show the field ONLY if:<br>[g1cd(12)] = '1'	Date of complication	text (date_dmy)
2692	[ps_g1cd]<br>Show the field ONLY if:<br>[g1cd(12)] = '1'	Please state	text
2693	[grade_i_claviendindo_postoperative_surgical_compli_complete]	Section Header: *Form Status*<br>Complete?	dropdown<br>0 Incomplete<br>1 Unverified<br>2 Complete

Instrument: **Grade II Clavien-Dindo post-operative surgical complications** (grade_ii_claviendindo_postoperative_surgical_compl)

2694	[g2cd]	Grade II Clavien-Dindo post-operative complications (select all that apply)	checkbox<br>1 g2cd___1 Tachyarrythmia requiring medical treatment<br>2 g2cd___2 Pneumonia treated with antibiotics on the ward<br>3 g2cd___3 Transient ischaemic attack (TIA) requiring treatment with anticoagulants<br>4 g2cd___4 Ileus requiring insertion of nasogastric tube<br>5 g2cd___5 Infectious diarrhoea requiring antibiotics<br>6 g2cd___6 Urinary tract infection requiring antibiotics<br>7 g2cd___7 Anemia following intra-operative blood loss requiring post operative blood transfusion<br>8 g2cd___8 Bowel obstruction managed conservatively on the ward (nasogastric tube, antiemetics, laxatives, IV fluids)<br>9 g2cd___9 Deep vein thrombosis<br>10 g2cd___10 Pulmonary embolism<br>11 g2cd___11 High out put stoma managed pharmacologically with anti-secretory / diarrhoeal medication<br>12 g2cd___12 Chyloascites managed pharmacologically with or without total parenteral nutrition<br>13 g2cd___13 Other<br>14 g2cd___14 None
2695	[blt_un_g2]<br>Show the field ONLY if:<br>[g2cd(7)] = '1'	Blood products transfused (select all that apply)	checkbox<br>1 blt_un_g2___1 red cell concentrate<br>2 blt_un_g2___2 platelet concentrate<br>3 blt_un_g2___3 fresh frozen plasma<br>4 blt_un_g2___4 cryopercipitate<br>5 blt_un_g2___5 whole blood
2696	[numb_un_g2]<br>Show the field ONLY if:<br>[blt_un_g2(1)] = '1'	Number of units	text (number)
2697	[numb_un_g3]<br>Show the field ONLY if:<br>[blt_un_g2(2)] = '1'	Number of units	text (number)
2698	[numb_un_g4]<br>Show the field ONLY if:<br>[blt_un_g2(3)] = '1'	Number of units	text (number)

2699	[numb_un_g5]<br>Show the field ONLY if:<br>[blt_un_g2(4)] = '1'	Number of units	text (number)
2700	[numb_un_g6]<br>Show the field ONLY if:<br>[blt_un_g2(5)] = '1'	Number of units	text (number)
2701	[dat_tachy]<br>Show the field ONLY if:<br>[g2cd(1)] = '1'	Date of complication	text (date_dmy)
2702	[dat_hap]<br>Show the field ONLY if:<br>[g2cd(2)] = '1'	Date of complication	text (date_dmy)
2703	[dat_tia]<br>Show the field ONLY if:<br>[g2cd(3)] = '1'	Date of complication	text (date_dmy)
2704	[dat_ileus]<br>Show the field ONLY if:<br>[g2cd(4)] = '1'	Date of complication	text (date_dmy)
2705	[dat_infec_diar]<br>Show the field ONLY if:<br>[g2cd(5)] = '1'	Date of complication	text (date_dmy)
2706	[dat_uti]<br>Show the field ONLY if:<br>[g2cd(6)] = '1'	Date of complication	text (date_dmy)
2707	[dat_bld_tx]<br>Show the field ONLY if:<br>[g2cd(7)] = '1'	Date of complication	text (date_dmy)
2708	[dat_bld_tx_2]<br>Show the field ONLY if:<br>[g2cd(8)] = '1'	Date of complication	text (date_dmy)
2709	[dat_bld_tx_3]<br>Show the field ONLY if:<br>[g2cd(9)] = '1'	Date of complication	text (date_dmy)
2710	[dat_bld_tx_4]<br>Show the field ONLY if:<br>[g2cd(10)] = '1'	Date of complication	text (date_dmy)
2711	[dat_hos_2]<br>Show the field ONLY if:<br>[g2cd(11)] = '1'	Date of complication	text (date_dmy)
2712	[dat_ca_cd2]<br>Show the field ONLY if:<br>[g2cd(12)] = '1'	Date of complication	text (date_dmy)
2713	[ps_g2cd]<br>Show the field ONLY if:<br>[g2cd(13)] = '1'	Please state	text
2714	[dat_other2]<br>Show the field ONLY if:<br>[g2cd(13)] = '1'	Date of complication	text (date_dmy)
2715	[grade_ii_claviendindo_postoperative_surgical_compl_complete]	Section Header: *Form Status*<br>Complete?	dropdown<br>0 Incomplete<br>1 Unverified<br>2 Complete

Instrument: **Grade IIIa Clavien-Dindo post-operative surgical complications** (grade_iiia_claviendindo_postoperative_surgical_com)

2716	[g3acd]	Grade IIIA Clavien-Dindo post-operative surgical complications (select all that apply)	checkbox<br>1 g3acd___1 Bradyarrythmia requiring pacemaker insertion<br>2 g3acd___2 Biloma after liver resection requiring percutaneous drainage<br>3 g3acd___3 Ureteric stenting following ureteric injury<br>4 g3acd___4 Closure of dehisced surgical wound in the operating room/theatre<br>5 g3acd___5 Percutaneous drainage of lymph ascites<br>6 g3acd___6 Percutaneous drainage of chyloascites<br>7 g3acd___7 Other<br>8 g3acd___8 None
2717	[dat_brady]<br>Show the field ONLY if:<br>[g3acd(1)] = '1'	Date of complication	text (date_dmy)
2718	[dat_bil]<br>Show the field ONLY if:<br>[g3acd(2)] = '1'	Date of complication	text (date_dmy)
2719	[dat_us]<br>Show the field ONLY if:<br>[g3acd(3)] = '1'	Date of complication	text (date_dmy)
2720	[dat_dehis]<br>Show the field ONLY if:<br>[g3acd(4)] = '1'	Date of complication	text (date_dmy)
2721	[dat_la]<br>Show the field ONLY if:<br>[g3acd(5)] = '1'	Date of complication	text (date_dmy)
2722	[dat_ca_cd3a]<br>Show the field ONLY if:<br>[g3acd(6)] = '1'	Date of complication	text (date_dmy)
2723	[ps_g3acd]<br>Show the field ONLY if:<br>[g3acd(7)] = '1'	Please state	text
2724	[dat_other3a]<br>Show the field ONLY if:<br>[g3acd(7)] = '1'	Date of complication	text (date_dmy)
2725	[grade_iiia_claviendindo_postoperative_surgical_com_complete]	Section Header: *Form Status*<br>Complete?	dropdown<br>0 Incomplete<br>1 Unverified<br>2 Complete
Instrument: **Grade IIIb Clavien-Dindo post-operative surgical complications** (grade_iiib_claviendindo_postoperative_surgical_com)			
2726	[g3bcd]	Grade IIIB Clavien-Dindo post-operative surgical complications	checkbox<br>1 g3bcd___1 Bowel anastamotic leak requiring return to theatre<br>2 g3bcd___2 Bowel obstruction requiring return to theatre<br>3 g3bcd___3 Bowel perforation requiring return to theatre<br>4 g3bcd___4 Fistula requiring return to theatre<br>5 g3bcd___5 Hernia requiring surgical intervention<br>6 g3bcd___6 Lymphocyst requiring surgical intervention<br>7 g3bcd___7 Other<br>8 g3bcd___8 None

2727	[fist_3b]<br>Show the field ONLY if:<br>[g3bcd(4)] = '1'	Type of fistula (select all that apply)	checkbox<br>1 fist_3b___1 Vesico-vaginal<br>2 fist_3b___2 Recto-vaginal<br>3 fist_3b___3 Ureterovaginal<br>4 fist_3b___4 Enterovesical<br>5 fist_3b___5 Enterocutaneous fistulas<br>6 fist_3b___6 Pancreatic<br>7 fist_3b___7 Other
2728	[ps_fist_3b]<br>Show the field ONLY if:<br>[fist_3b(7)] = '1'	Please state	text
2729	[dat_anast_lk]<br>Show the field ONLY if:<br>[g3bcd(1)] = '1'	Date of complication	text (date_dmy)
2730	[dat_anast_lk_2]<br>Show the field ONLY if:<br>[g3bcd(2)] = '1'	Date of complication	text (date_dmy)
2731	[dat_anast_lk_3]<br>Show the field ONLY if:<br>[g3bcd(3)] = '1'	Date of complication	text (date_dmy)
2732	[dat_anast_lk_4]<br>Show the field ONLY if:<br>[g3bcd(4)] = '1'	Date of complication	text (date_dmy)
2733	[dat_anast_lk_5]<br>Show the field ONLY if:<br>[g3bcd(5)] = '1'	Date of complication	text (date_dmy)
2734	[dat_anast_lk_6]<br>Show the field ONLY if:<br>[g3bcd(6)] = '1'	Date of complication	text (date_dmy)
2735	[ps_g3bcd]<br>Show the field ONLY if:<br>[g3bcd(7)] = '1'	Please state	text
2736	[doc_g3bcd]<br>Show the field ONLY if:<br>[g3bcd(7)] = '1'	Date of complication	text (date_dmy)
2737	[grade_iiib_claviendindo_postoperative_surgical_com_complete]	Section Header: *Form Status*<br>Complete?	dropdown<br>0 Incomplete<br>1 Unverified<br>2 Complete
	Instrument: **Grade IVa Clavien-Dindo post-operative surgical complications** (grade_iva_claviendindo_postoperative_surgical_comp)		
2738	[g4acd]	Grade IVA Clavien-Dindo post-operative complications (select all that apply)	checkbox<br>1 g4acd___1 Myocardial infarction<br>2 g4acd___2 Heart failure leading to low output<br>3 g4acd___3 Respiratory failure requiring intubation<br>4 g4acd___4 Stroke (ischemic/haemmorhagic)<br>5 g4acd___5 Renal insufficiency requiring dialysis<br>6 g4acd___6 Necrotising pancreatitis<br>7 g4acd___7 Other<br>8 g4acd___8 None
2739	[dat_mi]<br>Show the field ONLY if:<br>[g4acd(1)] = '1'	Date of complication	text (date_dmy)
2740	[dat_rf]<br>Show the field ONLY if:<br>[g4acd(2)] = '1'	Date of complication	text (date_dmy)

2741	[dat_respf]<br>Show the field ONLY if:<br>[g4acd(3)] = '1'	Date of complication	text (date_dmy)
2742	[dat_stroke]<br>Show the field ONLY if:<br>[g4acd(4)] = '1'	Date of complication	text (date_dmy)
2743	[dat_dial]<br>Show the field ONLY if:<br>[g4acd(5)] = '1'	Date of complication	text (date_dmy)
2744	[dat_panc]<br>Show the field ONLY if:<br>[g4acd(6)] = '1'	Date of complication	text (date_dmy)
2745	[ps_g4acd]<br>Show the field ONLY if:<br>[g4acd(7)] = '1'	Please state	text
2746	[dat_other4a]<br>Show the field ONLY if:<br>[g4acd(7)] = '1'	Date of complication	text (date_dmy)
2747	[grade_iva_claviendindo_postoperative_surgical_comp_complete]	Section Header: *Form Status*<br>Complete?	dropdown<br>0 Incomplete<br>1 Unverified<br>2 Complete

Instrument: **Grade IVb Clavien-Dindo post-operative surgical complications** (grade_ivb_claviendindo_postoperative_surgical_comp)

2748	[g4bcd]	Grade IVB Clavien-Dindo post-operative complications	checkbox<br>1 g4bcd___1 Multi-organ failure<br>2 g4bcd___2 Bowel anastamotic leak resulting in haemodynamic instability and return to theatre<br>3 g4bcd___3 Other<br>4 g4bcd___4 None
2749	[dat_mof]<br>Show the field ONLY if:<br>[g4bcd(1)] = '1'	Date of complication	text (date_dmy)
2750	[dat_anast_hdi]<br>Show the field ONLY if:<br>[g4bcd(2)] = '1'	Date of complication	text (date_dmy)
2751	[ps_g4bcd]<br>Show the field ONLY if:<br>[g4bcd(3)] = '1'	Please state	text
2752	[dat_other4b]<br>Show the field ONLY if:<br>[g4bcd(3)] = '1'	Date of complication	text (date_dmy)
2753	[grade_ivb_claviendindo_postoperative_surgical_comp_complete]	Section Header: *Form Status*<br>Complete?	dropdown<br>0 Incomplete<br>1 Unverified<br>2 Complete

Instrument: **Grade V Clavien-Dindo post-operative surgical complications** (grade_v_claviendindo_postoperative_surgical_compli)

2754	[cd_5]	Grade V Clavien-Dindo post-opertative complication	radio<br>1 Death<br>2 Not applicable (patient alive)
2755	[rip]<br>Show the field ONLY if:<br>[cd_5] = '1'	Date of death	text (date_dmy, Min: 0000.00.00)
2756	[grade_v_claviendindo_postoperative_surgical_compli_complete]	Section Header: *Form Status*<br>Complete?	dropdown<br>0 Incomplete<br>1 Unverified<br>2 Complete

Instrument: **Readmissions and return to theatre** (readmissions_and_return_to_theatre)

2757	[read]	Readmission	yesno<br>1 Yes<br>0 No
2758	[read_y_dat]<br>Show the field ONLY if:<br>[read] = '1'	Date of readmission	text (date_dmy)
2759	[read_reas]<br>Show the field ONLY if:<br>[read] = '1'	Reason for readmission (select all that apply)	checkbox<br>1 read_reas___1 operative site pain<br>2 read_reas___2 wound related complication (e.g. collection, infection, dehiscence, bleeding)<br>3 read_reas___3 Small bowel obstruction<br>4 read_reas___4 Large bowel obstruction<br>5 read_reas___5 Anastomotic leak<br>6 read_reas___6 Stoma related complication (e.g. vascular compromise, retraction, peristomal skin irritation, peristomal infection/abscess/fistula, parastomal herniation, bowel obstruction)<br>7 read_reas___7 Haemorrhage<br>8 read_reas___8 Pneumonia<br>9 read_reas___9 Urosepsis<br>10 read_reas___10 Cardiovascular (e.g. myocardial infarction, arrhythmia, stroke)<br>11 read_reas___11 Electrolyte imbalance<br>12 read_reas___12 Urinary retention<br>13 read_reas___13 Other
2760	[ps_reas_other]<br>Show the field ONLY if:<br>[read_reas(13)] = '1'	Please state	text
2761	[numb_read_dys]<br>Show the field ONLY if:<br>[read] = '1'	Number of days as inpatient during readmission	text (number, Min: 1)
2762	[rtt_yn]	Return to theatre	yesno<br>1 Yes<br>0 No
2763	[reas_rtt_y]<br>Show the field ONLY if:<br>[rtt_yn] = '1'	Reason for return to theatre	checkbox<br>1 reas_rtt_y___1 Haemorrhage<br>2 reas_rtt_y___2 Surgical wound dehiscence<br>3 reas_rtt_y___3 Anastomotic leak<br>4 reas_rtt_y___4 Small bowel obstruction<br>5 reas_rtt_y___5 Large bowel obstruction<br>6 reas_rtt_y___6 Stoma related complication<br>7 reas_rtt_y___7 To drain a collection<br>8 reas_rtt_y___8 Other
2764	[dat_rtt]<br>Show the field ONLY if:<br>[rtt_yn] = '1'	Date of return to theatre	text (date_dmy)<br>Field Annotation: d
2765	[ps_rtt]<br>Show the field ONLY if:<br>[reas_rtt_y(8)] = '1'	Please state	text

2766	[readmissions_and_return_to_theatre_complete]	Section Header: *Form Status*<br>Complete?	dropdown<br>0 Incomplete<br>1 Unverified<br>2 Complete
Instrument: **Death** (death)			
2767	[rip_cause]	Cause of death	dropdown<br>1 Cancer related<br>2 Non-cancer related
2768	[rip_dat_c]<br>Show the field ONLY if:<br>[rip_cause] = '1'	Date of death	text (date_dmy)
2769	[rip_dat_nc]<br>Show the field ONLY if:<br>[rip_cause] = '2'	Date of death	text (date_dmy)
2770	[death_complete]	Section Header: *Form Status*<br>Complete?	dropdown<br>0 Incomplete<br>1 Unverified<br>2 Complete
Instrument: **30 day follow up** (day_follow_up)			
2771	[fu_meth]	Method of follow up over the 30 day period from date of surgery (select all that apply)	checkbox, Required<br>1 fu_meth___1 Clinic review<br>2 fu_meth___2 Community/home review<br>3 fu_meth___3 Lost to follow up<br>4 fu_meth___4 Still inpatient<br>5 fu_meth___5 Telephone review<br>6 fu_meth___6 Reviewing hospital records for readmissions<br>7 fu_meth___7 Reviewing emergency department lists for readmissions<br>8 fu_meth___8 Contacting general/family practitioners to enquire about complications managed in primary care<br>9 fu_meth___9 Other
2772	[ps_fu_meth]<br>Show the field ONLY if:<br>[fu_meth(9)] = '1'	Please state	text
2773	[fu_dat]	Date of last follow up	text (date_dmy), Required
2774	[day_follow_up_complete]	Section Header: *Form Status*<br>Complete?	dropdown<br>0 Incomplete<br>1 Unverified<br>2 Complete
Instrument: **Patient reported outcomes (pre-op)** (patient_reported_outcomes)			
2775	[prom_mob]	Please click the ONE box that best describes your health TODAY.<br><br>MOBILITY	radio<br>1 I have no problems in walking about<br>2 I have slight problems in walking about<br>3 I have moderate problems in walking about<br>4 I have severe problems in walking about<br>5 I am unable to walk about

2776	[prom_sc]	Please click the ONE box that best describes your health TODAY.<br><br>SELF-CARE	radio<br>1 I have no problems washing or dressing myself<br>2 I have slight problems washing or dressing myself<br>3 I have moderate problems washing or dressing myself<br>4 I have severe problems washing or dressing myself<br>5 I am unable to wash or dress myself
2777	[prom_act]	Please click the ONE box that best describes your health TODAY.<br><br>USUAL ACTIVITIES (e.g. work, study, housework, family or leisure activities)	radio<br>1 I have no problems doing my usual activities<br>2 I have slight problems doing my usual activities<br>3 I have moderate problems doing my usual activities<br>4 I have severe problems doing my usual activities<br>5 I am unable to do my usual activities
2778	[prom_pain]	Please click the ONE box that best describes your health TODAY.<br><br>PAIN / DISCOMFORT	radio<br>1 I have no pain or discomfort<br>2 I have slight pain or discomfort<br>3 I have moderate pain or discomfort<br>4 I have severe pain or discomfort<br>5 I have extreme pain or discomfort
2779	[prom_dep]	Please click the ONE box that best describes your health TODAY.<br><br>ANXIETY / DEPRESSION	radio<br>1 I am not anxious or depressed<br>2 I am slightly anxious or depressed<br>3 I am moderately anxious or depressed<br>4 I am severely anxious or depressed<br>5 I am extremely anxious or depressed
2780	[prom_sca]	We would like to know how good or bad your health is TODAY.<br><br>This scale is numbered from 0 to 100.<br><br>100 means the best health you can imagine.<br>0 means the worst health you can imagine.<br><br>Please click on the scale to indicate how your health is TODAY.	slider<br>Slider labels: 100 - The best health you can imagine, 50, 0 - The worst health you can imagine<br>Custom alignment: RH
2781	[patient_reported_outcomes_complete]	Section Header: *Form Status*<br>Complete?	dropdown<br>0 Incomplete<br>1 Unverified<br>2 Complete
Instrument: **Patient reported outcomes (post- op day 1)** (patient_reported_outcomes_post_op_day_1_2)			
2782	[prom_mob_v2]	Please click the ONE box that best describes your health TODAY.<br><br>MOBILITY	radio<br>1 I have no problems in walking about<br>2 I have slight problems in walking about<br>3 I have moderate problems in walking about<br>4 I have severe problems in walking about<br>5 I am unable to walk about
2783	[prom_sc_v2]	Please click the ONE box that best describes your health TODAY.<br><br>SELF-CARE	radio<br>1 I have no problems washing or dressing myself<br>2 I have slight problems washing or dressing myself<br>3 I have moderate problems washing or dressing myself<br>4 I have severe problems washing or dressing myself<br>5 I am unable to wash or dress myself

2784	[prom_act_v2]	Please click the ONE box that best describes your health TODAY.<br><br>USUAL ACTIVITIES (e.g. work, study, housework, family or leisure activities)	radio<br>1 I have no problems doing my usual activities<br>2 I have slight problems doing my usual activities<br>3 I have moderate problems doing my usual activities<br>4 I have severe problems doing my usual activities<br>5 I am unable to do my usual activities
2785	[prom_pain_v2]	Please click the ONE box that best describes your health TODAY.<br><br>PAIN / DISCOMFORT	radio<br>1 I have no pain or discomfort<br>2 I have slight pain or discomfort<br>3 I have moderate pain or discomfort<br>4 I have severe pain or discomfort<br>5 I have extreme pain or discomfort
2786	[prom_dep_v2]	Please click the ONE box that best describes your health TODAY.<br><br>ANXIETY / DEPRESSION	radio<br>1 I am not anxious or depressed<br>2 I am slightly anxious or depressed<br>3 I am moderately anxious or depressed<br>4 I am severely anxious or depressed<br>5 I am extremely anxious or depressed
2787	[prom_sca_v2]	We would like to know how good or bad your health is TODAY.<br><br>This scale is numbered from 0 to 100.<br><br>100 means the best health you can imagine.<br>0 means the worst health you can imagine.<br><br>Please click on the scale to indicate how your health is TODAY.	slider<br>Slider labels: 100 - The best health you can imagine, 50, 0 - The worst health you can imagine<br>Custom alignment: RH
2788	[patient_reported_outcomes_post_op_day_1_2_complete]	Section Header: *Form Status*<br>Complete?	dropdown<br>0 Incomplete<br>1 Unverified<br>2 Complete

Instrument: **Patient reported outcomes (post- op on day of discharge from hospital)**
(patient_reported_outcomes_post_op_day_1_2_43ef)

2789	[prom_mob_v2_v2]	Please click the ONE box that best describes your health TODAY.<br><br>MOBILITY	radio<br>1 I have no problems in walking about<br>2 I have slight problems in walking about<br>3 I have moderate problems in walking about<br>4 I have severe problems in walking about<br>5 I am unable to walk about
2790	[prom_sc_v2_v2]	Please click the ONE box that best describes your health TODAY.<br><br>SELF-CARE	radio<br>1 I have no problems washing or dressing myself<br>2 I have slight problems washing or dressing myself<br>3 I have moderate problems washing or dressing myself<br>4 I have severe problems washing or dressing myself<br>5 I am unable to wash or dress myself
2791	[prom_act_v2_v2]	Please click the ONE box that best describes your health TODAY.<br><br>USUAL ACTIVITIES (e.g. work, study, housework, family or leisure activities)	radio<br>1 I have no problems doing my usual activities<br>2 I have slight problems doing my usual activities<br>3 I have moderate problems doing my usual activities<br>4 I have severe problems doing my usual activities<br>5 I am unable to do my usual activities

2792	[prom_pain_v2_v2]	Please click the ONE box that best describes your health TODAY.<br><br>PAIN / DISCOMFORT	radio<br>1 I have no pain or discomfort<br>2 I have slight pain or discomfort<br>3 I have moderate pain or discomfort<br>4 I have severe pain or discomfort<br>5 I have extreme pain or discomfort
2793	[prom_dep_v2_v2]	Please click the ONE box that best describes your health TODAY.<br><br>ANXIETY / DEPRESSION	radio<br>1 I am not anxious or depressed<br>2 I am slightly anxious or depressed<br>3 I am moderately anxious or depressed<br>4 I am severely anxious or depressed<br>5 I am extremely anxious or depressed
2794	[prom_sca_v2_v2]	We would like to know how good or bad your health is TODAY.<br><br>This scale is numbered from 0 to 100.<br><br>100 means the best health you can imagine.<br>0 means the worst health you can imagine.<br><br>Please click on the scale to indicate how your health is TODAY.	slider<br>Slider labels: 100 - The best health you can imagine, 50, 0 - The worst health you can imagine<br>Custom alignment: RH
2795	[patient_reported_outcomes_post_op_day_1_2_43ef_complete]	Section Header: *Form Status*<br>Complete?	dropdown<br>0 Incomplete<br>1 Unverified<br>2 Complete
Instrument: **Patient reported outcomes (post- op 3 months)** (patient_reported_outcomes_post_op_3_months)			
2796	[prom_mob_v2_v2_v2]	Please click the ONE box that best describes your health TODAY.<br><br>MOBILITY	radio<br>1 I have no problems in walking about<br>2 I have slight problems in walking about<br>3 I have moderate problems in walking about<br>4 I have severe problems in walking about<br>5 I am unable to walk about
2797	[prom_sc_v2_v2_v2]	Please click the ONE box that best describes your health TODAY.<br><br>SELF-CARE	radio<br>1 I have no problems washing or dressing myself<br>2 I have slight problems washing or dressing myself<br>3 I have moderate problems washing or dressing myself<br>4 I have severe problems washing or dressing myself<br>5 I am unable to wash or dress myself
2798	[prom_act_v2_v2_v2]	Please click the ONE box that best describes your health TODAY.<br><br>USUAL ACTIVITIES (e.g. work, study, housework, family or leisure activities)	radio<br>1 I have no problems doing my usual activities<br>2 I have slight problems doing my usual activities<br>3 I have moderate problems doing my usual activities<br>4 I have severe problems doing my usual activities<br>5 I am unable to do my usual activities
2799	[prom_pain_v2_v2_v2]	Please click the ONE box that best describes your health TODAY.<br><br>PAIN / DISCOMFORT	radio<br>1 I have no pain or discomfort<br>2 I have slight pain or discomfort<br>3 I have moderate pain or discomfort<br>4 I have severe pain or discomfort<br>5 I have extreme pain or discomfort

2800	[prom_dep_v2_v2_v2]	Please click the ONE box that best describes your health TODAY.<br><br>ANXIETY / DEPRESSION	radio<br>1 I am not anxious or depressed<br>2 I am slightly anxious or depressed<br>3 I am moderately anxious or depressed<br>4 I am severely anxious or depressed<br>5 I am extremely anxious or depressed
2801	[prom_sca_v2_v2_v2]	We would like to know how good or bad your health is TODAY.<br><br>This scale is numbered from 0 to 100.<br><br>100 means the best health you can imagine.<br>0 means the worst health you can imagine.<br><br>Please click on the scale to indicate how your health is TODAY.	slider<br>Slider labels: 100 - The best health you can imagine, 50, 0 - The worst health you can imagine<br>Custom alignment: RH
2802	[patient_reported_outcomes_post_op_3_months_complete]	Section Header: *Form Status*<br>Complete?	dropdown<br>0 Incomplete<br>1 Unverified<br>2 Complete
Instrument: **Patient reported outcomes (post- op 6 months)** (patient_reported_outcomes_post_op_6_months)			
2803	[prom_mob_v2_v2_v2_v2]	Please click the ONE box that best describes your health TODAY.<br><br>MOBILITY	radio<br>1 I have no problems in walking about<br>2 I have slight problems in walking about<br>3 I have moderate problems in walking about<br>4 I have severe problems in walking about<br>5 I am unable to walk about
2804	[prom_sc_v2_v2_v2_v2]	Please click the ONE box that best describes your health TODAY.<br><br>SELF-CARE	radio<br>1 I have no problems washing or dressing myself<br>2 I have slight problems washing or dressing myself<br>3 I have moderate problems washing or dressing myself<br>4 I have severe problems washing or dressing myself<br>5 I am unable to wash or dress myself
2805	[prom_act_v2_v2_v2_v2]	Please click the ONE box that best describes your health TODAY.<br><br>USUAL ACTIVITIES (e.g. work, study, housework, family or leisure activities)	radio<br>1 I have no problems doing my usual activities<br>2 I have slight problems doing my usual activities<br>3 I have moderate problems doing my usual activities<br>4 I have severe problems doing my usual activities<br>5 I am unable to do my usual activities
2806	[prom_pain_v2_v2_v2_v2]	Please click the ONE box that best describes your health TODAY.<br><br>PAIN / DISCOMFORT	radio<br>1 I have no pain or discomfort<br>2 I have slight pain or discomfort<br>3 I have moderate pain or discomfort<br>4 I have severe pain or discomfort<br>5 I have extreme pain or discomfort
2807	[prom_dep_v2_v2_v2_v2]	Please click the ONE box that best describes your health TODAY.<br><br>ANXIETY / DEPRESSION	radio<br>1 I am not anxious or depressed<br>2 I am slightly anxious or depressed<br>3 I am moderately anxious or depressed<br>4 I am severely anxious or depressed<br>5 I am extremely anxious or depressed

2808	[prom_sca_v2_v2_v2_v2]	We would like to know how good or bad your health is TODAY.<br><br>This scale is numbered from 0 to 100.<br><br>100 means the best health you can imagine.<br>0 means the worst health you can imagine.<br><br>Please click on the scale to indicate how your health is TODAY.	slider<br>Slider labels: 100 - The best health you can imagine, 50, 0 - The worst health you can imagine<br>Custom alignment: RH
2809	[patient_reported_outcomes_post_op_6_months_complete]	Section Header: *Form Status*<br>Complete?	dropdown<br>0 Incomplete<br>1 Unverified<br>2 Complete
Instrument: **Patient reported outcomes (post- op 12 months)** (patient_reported_outcomes_post_op_12_months)			
2810	[prom_mob_v2_v2_v2_v2_v2]	Please click the ONE box that best describes your health TODAY.<br><br>MOBILITY	radio<br>1 I have no problems in walking about<br>2 I have slight problems in walking about<br>3 I have moderate problems in walking about<br>4 I have severe problems in walking about<br>5 I am unable to walk about
2811	[prom_sc_v2_v2_v2_v2_v2]	Please click the ONE box that best describes your health TODAY.<br><br>SELF-CARE	radio<br>1 I have no problems washing or dressing myself<br>2 I have slight problems washing or dressing myself<br>3 I have moderate problems washing or dressing myself<br>4 I have severe problems washing or dressing myself<br>5 I am unable to wash or dress myself
2812	[prom_act_v2_v2_v2_v2_v2]	Please click the ONE box that best describes your health TODAY.<br><br>USUAL ACTIVITIES (e.g. work, study, housework, family or leisure activities)	radio<br>1 I have no problems doing my usual activities<br>2 I have slight problems doing my usual activities<br>3 I have moderate problems doing my usual activities<br>4 I have severe problems doing my usual activities<br>5 I am unable to do my usual activities
2813	[prom_pain_v2_v2_v2_v2_v2]	Please click the ONE box that best describes your health TODAY.<br><br>PAIN / DISCOMFORT	radio<br>1 I have no pain or discomfort<br>2 I have slight pain or discomfort<br>3 I have moderate pain or discomfort<br>4 I have severe pain or discomfort<br>5 I have extreme pain or discomfort
2814	[prom_dep_v2_v2_v2_v2_v2]	Please click the ONE box that best describes your health TODAY.<br><br>ANXIETY / DEPRESSION	radio<br>1 I am not anxious or depressed<br>2 I am slightly anxious or depressed<br>3 I am moderately anxious or depressed<br>4 I am severely anxious or depressed<br>5 I am extremely anxious or depressed
2815	[prom_sca_v2_v2_v2_v2_v2]	We would like to know how good or bad your health is TODAY.<br><br>This scale is numbered from 0 to 100.<br><br>100 means the best health you can imagine.<br>0 means the worst health you can imagine.<br><br>Please click on the scale to indicate how your health is TODAY.	slider<br>Slider labels: 100 - The best health you can imagine, 50, 0 - The worst health you can imagine<br>Custom alignment: RH
2816	[patient_reported_outcomes_post_op_12_months_complete]	Section Header: *Form Status*<br>Complete?	dropdown<br>0 Incomplete<br>1 Unverified<br>2 Complete
