# Supplementary material for: International Variations in Surgical Morbidity and Mortality Post Gynaecological Oncology Surgery: A Global Gynaecological Oncology Surgical Outcomes Collaborative Led Study (GO SOAR1)
Source: Cancers (Basel). 2023 Oct 16;15(20):5001. doi: 10.3390/cancers15205001 (PMC10605858; doi:10.3390/cancers15205001)
Supplement: Supplementary file 1 [file cancers-15-05001-s001.zip › Supplementary_File_S2.pdf]

Topic guide for qualitative sub-study: GO SOAR1

**Research Aims to explore**<br>Participating in research.<br>Gynaecological Oncology Training.<br>Surgical morbidity and mortality.

1. **Introduction**

- Introduce self, research study funded by Grampian Endowment Fund, Medtronic.
- Purpose of the research.
- Introduce audio recorder.
- Stress all identifiable information will be kept confidential, but anonymised quotes may be published/presented.

2. **Background**

- Demographics: name, age, gender, country, centre name, rural or urban setting, government funded or non-government funded institution
- Job
  Q: What is your role in the surgical team?
  P: consultant/attending, fellow/registrar/resident, medical student, nurse
  Q: What is your role in the research team?
  P: principle investigator (PI), research fellow
- Experience
  Q: Would you consider yourself an experienced researcher?
  P: number of years, formal training/qualification, take part in research studies, set up and lead studies, national vs international participation

3. **Research participation**

- Facilitators (individual, organisational, policy related)
  Q: *Based on your experience, what motivates you to participate in research?*
  P: authorship, making new contacts, want to improve care, recognition from the scientific/clinical community, financial gain
- Barriers
  Q: *Based on your experience, what deters you from participating in research?*
  P: *clinical commitments/time, complicated/time consuming ethical approval process, not being invited to join studies i.e. no opportunities, research naïve, working environment/job plan not conducive to research activities, no research training, lack of resources (infrastructure, money), motivation, culture/language, research culture – low priority, COVID pandemic*
- Change
  Q: *What changes would make you more likely to take part in research?*
  P: *local, international*

4. **Gynaecological oncology training**

- Q: *Is there formal gynae onc training?*
  P: local or national training programme, international fellowships, no training, do other surgical specialities perform gynae onc surgery, who performs bowel/urology/reconstruction surgery – gynae onc or other specialities or gynae onc with support of other specialities

5. **Surgical morbidity/mortality**

- *Q: Is there anything as part of your routine clinical practice which negatively impacts on morbidity/mortality?*

P: lack of training/knowledge on best practice guidelines, resources (eg lack of surgical expertise for complex surgery, not enough nursing staff for post op recovery, lack of medicines e.g. antibiotics), surgical training, hospital/national policies, does government consider womens health a national priority, health insurance/finances i.e. no money no care/limited care,

- *Q: Is there anything as part of your routine clinical practice which positively impacts on morbidity/mortality?*
  P: formal training, sufficient resources, hospital/national guidelines/policies, government consider womens health a national priority, government funded health care for all – free at point of need, enhanced recovery
- *Q: What clinical practice guidelines do you follow?*
  P: none, local, national, international (ask which ones e.g. ESGO/SGO/NCCN/BGCS/IGCS/any other)

Final steps:

Thank the participant. Check whether they have remaining questions or comments about the topic
Reassurance about confidentiality and anonymity
Research team contact details should they want further information

Q-question; P-probe.
