# Supplementary material for: International Variations in Surgical Morbidity and Mortality Post Gynaecological Oncology Surgery: A Global Gynaecological Oncology Surgical Outcomes Collaborative Led Study (GO SOAR1)
Source: Cancers (Basel). 2023 Oct 16;15(20):5001. doi: 10.3390/cancers15205001 (PMC10605858; doi:10.3390/cancers15205001)
Supplement: Supplementary file 1 [file cancers-15-05001-s001.zip › Supplementary_File_S3.pdf]

GO SOAR Collaborators (Group Authorship)

Mohamedraed Elshami, Islamic Univesity of Gaza, Gaza City, Palestine
Muhammed Elhadi, Tripoli University Hospital, Tripoli, Libya
Salah Eddine Oussama Kacimi, Abou Bakr Belkaid University of Tlemcen, Tlemcen, Algeria
Domenico Ferraioli, Cancer Center Leon Berard, Lyon, France
Noor Abualdahab Yarmouk University, Irbid, Jordan
Lina Karout, American University of Beirut, Beirut, Lebanon
Alaa Ismail, Islamic University of Gaza, Gaza City, Palestine
Monika Sobocan, UMC Maribor, Maribor, Slovenia
Mustafa Mohamed Ali Hussein, Omdurman military hospital, Khartoum, Sudan
Aya Zazo, Aleppo University Hospital, Aleppo, Syria
Glauco Baiocchi, AC Camargo Cancer Center, São Paulo, Brazil
Bruna Triapelli Goncalves, AC Camargo Cancer Center, São Paulo, Brazil
Ademola Adeyeye, Afe Babalola University, Ado-Ekiti, Nigeria
Ahmad Al Houri, Al-Bairouni University Hospital, Damascus University, Damascus, Syria
Hasan Al Houri, Al-Bairouni University Hospital, Damascus University, Damascus, Syria
Marah Al Matar, Al-Bairouni University Hospital, Damascus University, Damascus, Syria
Almu'atasim Khamees, Al-Basheer Hospital, Amman, Jordan
Mustafa Sabri Massadi, Al-Basheer Hospital, Amman, Jordan
Mohamad Banat, Al-Makassed Hospital, East Jerusalem, Palestine
Omar Badaren, Al-Makassed Hospital, East Jerusalem, Palestine
Dema Adwan, Maternity Hospital Damascus university (Al-Tawleed Hospital), Damascus, Syria
Mustafa Albokai, Maternity Hospital Damascus university (Al-Tawleed Hospital), Damascus, Syria
Halima.A.Sh.Alhasie, Albida Medical Centre, Faculty of Medicine, Omar Al-Mukhtar University, Al Bayda, Libya
Wisam Abraheem Hasan, Faculty of Medicine, Omar Al-Mukhtar University, Al Bayda, Libya
Hamdi Nawfal, Aleppo University Hospital of Obstetrics and Gynecology, Aleppo, Syria
Amr Hamza, Aleppo University Hospital, Aleppo, Syria
Rema Hassan Otman, Al Khadra Hospital, Tripoli, Libya
Reyam Abdalla Naana, Al Khadra Hospital, Tripoli, Libya
Igor Govorov, Almazov National Medical Research Centre, St Petersburg, Russia
Elena Dikareva, Almazov National Medical Research Centre, St Petersburg, Russia
Elena Ulrikh, Almazov National Medical Research Centre, St Petersburg, Russia
Baraa Hajjaj, Al Shifa Hospital, Gaza City, Palestine
Shukri Odeh Yousef, Augusta Victoria Hospital, East Jerusalem, Palestine
Ali Al-Isawi, Babylon Maternity and Children's Hospital, Babylon, Iraq
Alyaa Raad Al-Khafaji, Babylon Maternity and Children's Hospital, Babylon, Iraq
Ahmed M. Abbas, Faculty of Medicine, Assiut University, Assiut, Egypt
Mohammed F. Ramadan, Faculty of Medicine, Assiut University, Assiut, Egypt
Mustafa M. Khodry, South Valley University, Qena, Egypt
Mohammad Awad, Beit Jala Governmental Hospital, Beit Jala City, West Bank, Palestine
Swali Fundaunda, Cancer Diseases Hospital, Lusaka, Zambia
Mukatimui Munalula Kalima, Cancer Diseases Hospital, Lusaka, Zambia
Anisse Tidjane, University of Oran 1 EHU-1st November 1954, Oran, Algeria
Farah Shahin, Government Hospital Hebron, Hebron, Palestine
David Atallah, Hotel Dieu de France University Hospital, Beirut, Lebanon
Samir Tarchichi, Hotel Dieu de France University Hospital, Beirut, Lebanon
Iman Feghali, Hotel Dieu de France University Hospital, Beirut, Lebanon
Nadine El Kassis, Hotel Dieu de France University Hospital, Beirut, Lebanon
Claudia Bessa Pereira Chaves, Brazilian National Cancer Institute, Rio de Janerio, Brazil
Daniel de Souza Fernandes, Brazilian National Cancer Institute, Rio de Janerio, Brazil
Ana Claudia Frazao Paiva, Brazilian National Cancer Institute, Rio de Janerio, Brazil
Rafaela Silva de Oliveira, Brazilian National Cancer Institute, Rio de Janerio, Brazil
Maen Alsumadi, Jordan Hospital, Amman, Jordan: Bushra Al Mikhi, Jordan Hospital, Amman, Jordan
Ziena Obiedat, Jordan Hospital, Amman, Jordan
Seda Şahin Aker, Kayseri City Education and Research Hospital, Kayseri, Türkiye
Hüseyin Aksoy, Kayseri City Education and Research Hospital, Kayseri, Türkiye

Amer Mahmoud Sindiani, Jordan University of Science and Technology, Irbid, Jordan
Wafa' Thani Alsardieh, Jordan University of Science and Technology, Irbid, Jordan
Cagatay Taskiran, Koc University School of Medicine, Istanbul, Türkiye
Burak Giray, Koc University School of Medicine, Istanbul, Türkiye
Dogan Vatansever, Koc University School of Medicine, Istanbul, Türkiye
Ayodeji Kayode Adefemi, Lagos State University Teaching Hospital, Lagos, Nigeria
Ayokunle Moses Olumodeji, Lagos State University Teaching Hospital, Lagos, Nigeria
Basel Refky, Mansoura University, Mansoura, Egypt
Waleed Elamin, Mansoura University, Mansoura, Egypt
Hassan Alsalman, Mansoura University, Mansoura, Egypt
Svetlana Kulikova, Moscow City Oncology Hospital #62, Moscow, Russia
Pavel Sorokin, Moscow City Oncology Hospital #62, Moscow, Russia
Dmitrii Utkin, Moscow City Oncology Hospital #62, Moscow, Russia
Mikhail Novozhilov, Moscow City Oncology Hospital #62, Moscow, Russia
Zarema Abduragimova, Moscow City Oncology Hospital #62, Moscow, Russia
Zhirayr Malakyan, Nairi MC, Yerevan, Armenia
Artem Stepanyan, Nairi MC, Yerevan, Armenia
Areg Alaverdyan, Nairi MC, Yerevan, Armenia
Mustafa A. Abu Jayyab, Islamic University of Gaza, Gaza City, Palestine
Husameddin Ashraf Alattar, Islamic University of Gaza, Gaza City, Palestine
Abdulhadi Othman Alshatshat, National Cancer Institute (Misurata), Misrata, Libya
Hammad Ali Fadlalmola, National Ribat University Hospital, Khartoum, Sudan
Zouhair Amarin, Jordan University of Science and Technology, Irbid, Jordan
Hazim Ababneh, Jordan University of Science and Technology, Irbid, Jordan
Amar Al-Jarrah, Jordan University of Science and Technology, Irbid, Jordan
Mohammad AM Ahmed, South Valley University, Qena, Egypt
Sara Hussein, South Valley University, Qena, Egypt
V Annapurna, Sri Shankara cancer Hospital and research centre, Bangalore, India
Rekha BR, Sri Shankara cancer Hospital and research centre, Bangalore, India
Jaydip Bhaumik, Tata Medical Center, Kolkata, India
Anik Ghosh,Tata Medical Center, Kolkata, India
Jagannath Mishra, Tata Medical Center, Kolkata, India
Zuheir Al-Shehabi, Tishreen University Hospital, Latakia, Syria
Bassam Hassan, Tishreen University Hospital, Latakia, Syria
Maherah Kamarudin, Universiti Malaya, Kuala Lumpur, Malaysia
Chee Yang Tan, Universiti Malaya, Kuala Lumpur, Malaysia
Mariam Anjum Ifthikar, Yenepoya Medical College Hospital, Mangalore, India
Kiran Kumar K M, Yenepoya Medical College Hospital, Mangalore, India
M Vijaykumar, Yenepoya Medical College Hospital, Mangalore, India
Rohan Shetty, Yenepoya Medical College Hospital, Mangalore, India
Jallaluddin Akbar, Yenepoya Medical College Hospital, Mangalore, India
Ali Elimam, East Nile (Sharg Al-Neel) Hospital, Khartoum, Sudan
Shatha Husain, The Islamic Hospital, Amman, Jordan
Martina Arcieri, University of Messina, Messina, Italy
Alfredo Ercoli, University of Messina, Messina, Italy
Davide Arrigo, University of Messina, Messina, Italy
Stefano Restaino, University Hospital of Udine, Azienda Ospedaliera Universitaria Friuli Centrale, Udine, Italy
Giuseppe Vizzielli, University of Udine, Azienda Ospedaliera Universitaria Friuli Centrale, Udine, Italy
Giulia Pellecchia, University of Udine, Udine, Italy
Lorenza Driu, University of Udine, Azienda Ospedaliera Universitaria Friuli Centrale, Udine, Italy
Matilde Degano, University of Udine, Udine, Italy
Joao Cassanova, Champalimaud Foundation, Lisbon, Portugal
Hospital da Luz Lisboa, Lisbon, Portugal
Sara Proença, Champalimaud Foundation, Lisbon, Portugal
Basile Pache, CHUV, Lausanne, Switzerland
Patrice Mathevet, CHUV, Lausanne, Switzerland
Renata Poncová, First Faculty of Medicine, Charles University in Prague, Czech Republic:

Christine Ang, Queen Elizabeth Hospital, Gateshead Health NHS Foundation Trust, Gateshead, UK
Viktor Ivan Cassar, Queen Elizabeth Hospital, Gateshead Health NHS Foundation Trust, Gateshead, UK:
Lorraine Pearce, Queen Elizabeth Hospital, Gateshead Health NHS Foundation Trust, Gateshead, UK
Manuela Undurraga, Hôpitaux Universitaire de Genève, Geneva, Switzerland
Benedetta Guani, MEC Centre du Sein Fribourg, Fribourg, Switzerland
Anis Feki, MEC Centre du Sein Fribourg, Fribourg, Switzerland
Gemma Mancebo, Hospital del Mar. PSMAR. Barcelona, Barcelona, Spain
Berta Fabregó, Hospital del Mar. PSMAR. Barcelona, Barcelona, Spain
Ester Miralpeix, Hospital del Mar. PSMAR. Barcelona, Spain
Blanca Gil-Ibáñez, University Hospital 12 de Octubre, Madrid, Spain
Alvaro Tejerizo, University Hospital 12 de Octubre, Madrid, Spain
Gregorio López González, University Hospital 12 de Octubre, Madrid, Spain
Manuel Pantoja-Garrido, University Hospital Virgen Macarena, Seville, Spain
Alfredo Polo-Velasco, University Hospital Virgen Macarena, Seville, Spain
Julia Rodríguez-Sánchez-Reyman, University Hospital Virgen Macarena, Seville, Spain
Fabio Martinelli, Fondazione IRCCS Istituto Nazionale dei Tumori di Milano, Milan, Italy
Chiara Cimmino, Fondazione IRCCS Istituto Nazionale dei Tumori di Milano, Milan, Italy
Francesco Raspagliesi, Fondazione IRCCS Istituto Nazionale dei Tumori di Milano, Milan, Italy
Martina Aida Angeles, Institut Claudius Regaud, Institut Universitaire du Cancer de Toulouse-Oncopole, Toulouse, France
Mathilde Del, Institut Claudius Regaud, Institut Universitaire du Cancer de Toulouse-Oncopole, Toulouse, France
Alejandra Martinez, Institut Claudius Regaud, Institut Universitaire du Cancer de Toulouse-Oncopole, Toulouse, France
Marc Fischer, Kantosspital St. Gallen, St Gallen, Switzerland
Henrik Falconer, Karolinska University Hospital, Stockholm, Sweden
Kajsa Lidman, Karolinska University Hospital, Stockholm, Sweden
Agnieszka Rychlik, Maria Skłodowska-Curie National Research Institute of Oncology, Warsaw, Poland
Katarzyna Derda, Maria Skłodowska-Curie National Research Institute of Oncology, Warsaw, Poland
Mahalakshmi Gurumurthy, Aberdeen Royal Infirmary, NHS Grampian, UK
Mary Cairns, Aberdeen Royal Infirmary, NHS Grampian, UK
Lori McPherson, Aberdeen Royal Infirmary, NHS Grampian, UK
Olivia W. Foley, Northwestern University Feinberg School of Medicine, Chicago, USA
Dario R. Roque, Northwestern University Feinberg School of Medicine, Chicago, USA
Edoardo Cola, Macerata Hospital, Macerata, Spain
Mauro Pelagalli, Macerata Hospital, Macerata, Spain
Andrea Papadia, Ente Ospedaliero Cantonale of Lugano (EOC), Lugano, Switzerland
Maria Luisa Gasparri, Ente Ospedaliero Cantonale of Lugano (EOC), Lugano, Switzerland
Anna Fagotti, Fondazione Policlinico Universitario A. Gemelli, IRCCS, Rome, Italy
Alice Zampolini Faustini, Fondazione Policlinico Universitario A. Gemelli, IRCCS, Rome, Italy
Giulia Ferrante, Fondazione Policlinico Universitario A. Gemelli, IRCCS, Rome, Italy
Giovanni Scambia, Fondazione Policlinico Universitario A. Gemelli, IRCCS, Rome, Italy
Thumuluru Kavitha Madhuri, Royal Surrey County Hospital, Royal Surrey NHS Foundation Trust, UK
Hasanthi Assa Laarachchi, Royal Surrey County Hospital, Royal Surrey NHS Foundation Trust, UK
Aqsa Aslam, Royal Surrey County Hospital, Royal Surrey NHS Foundation Trust, UK
Saurabh Phadnis, The Royal London Hospital, Barts Health NHS Trust, London, UK
Elly Brockbank, The Royal London Hospital, Barts Health NHS Trust, London, UK
Alexandra Lawrence, The Royal London Hospital, Barts Health NHS Trust, London, UK
Arjun Jeyarajah, The Royal London Hospital, Barts Health NHS Trust, London, UK
James Dilley, The Royal London Hospital, Barts Health NHS Trust, London, UK
Ranjit Manchanda, The Royal London Hospital, Barts Health NHS Trust, London, UK
Ana Patricia Louise Alvarado, The Royal London Hospital, Barts Health NHS Trust, London, UK
John Butler, The Royal Marsden Hospital, The Royal Marsden NHS Foundation Trust, London, UK
Nana Gomes, The Royal Marsden Hospital, The Royal Marsden NHS Foundation Trust, London, UK
Owen Heath, The Royal Marsden Hospital, The Royal Marsden NHS Foundation Trust, London, UK
Marielle Nobbenhuis, The Royal Marsden Hospital, The Royal Marsden NHS Foundation Trust, London, UK
Thomas Ind, The Royal Marsden Hospital, The Royal Marsden NHS Foundation Trust, London, UK

Desmond Barton, The Royal Marsden Hospital, The Royal Marsden NHS Foundation Trust, London, UK
Víctor Lago, University Hospital La Fe Valencia, Valencia, Spain
Aarti Sharma, University Hospital of Wales, Cardiff and Vale University Health Board, Cardiff, UK
Hassan Zeinah, University Hospital of Wales, Cardiff and Vale University Health Board, Cardiff, UK
Catherine Smith, University Hospital of Wales, Cardiff and Vale University Health Board, Cardiff, UK
Andrej Cokan, UMC Maribor, Maribor, Slovenia
Eva Timošek, UMC Maribor, Maribor, Slovenia
Alyssa Wield, Magee-Womens Hospital, University of Pittsburgh, Pittsburgh, USA
Isabel Janmey, Magee-Womens Hospital, University of Pittsburgh, Pittsburgh, USA
Natalia R. Gomez-Hidalgo, Vall d'Hebron University hospital, Barcelona, Spain
Ana Luzarraga Aznar, Vall d'Hebron University hospital, Barcelona, Spain
