# Supplementary material for: International Variations in Surgical Morbidity and Mortality Post Gynaecological Oncology Surgery: A Global Gynaecological Oncology Surgical Outcomes Collaborative Led Study (GO SOAR1)
Source: Cancers (Basel). 2023 Oct 16;15(20):5001. doi: 10.3390/cancers15205001 (PMC10605858; doi:10.3390/cancers15205001)
Supplement: Supplementary file 1 [file cancers-15-05001-s001.zip › supplementary_tables.pdf]

Supplementary Table S1: Proportion of patients by cancer stage per tumour and country income group

	Ovary		Uterus		Cervix		Vulva		Vagina		GTD	
	HIC	LMIC	HIC	LMIC	HIC	LMIC	HIC	LMIC	HIC	LMIC	HIC	LMIC
**FIGO stage I**	29.2% (131/448)	31.8% (85/267)	69.2% (306/442)	60.9% (184/302)	69% (69/100)	63.2% (36/57)	73% (46/63)	67.8% (40/59)	50% (2/4)	60% (6/10)	66.7% (2/3)	83.3% (5/6)
**FIGO stage II**	7.1% (32/448)	14.2% (38/267)	8.6% (38/442)	9.9% (30/302)	8% (8/100)	10.5% (6/57)	1.6% (1/63)	1.7% (1/59)	25% (1/4)	20% (2/10)	0% (0/3)	16.7% (1/6)
**FIGO stage III**	46.4% (208/448)	40.8% (109/267)	15.8% (70/442)	21.9% (66/302)	14% (14/100)	24.6% (14/57)	22.2% (14/63)	28.8% (17/59)	0% (0/4)	10% (1/10)	33.3% (1/3)	0% (0/6)
**FIGO stage IV**	17.2% (77/448)	13.1% (35/267)	6.3% (28/442)	7.3% (22/302)	9% (9/100)	1.8% (1/57)	3.2% (2/63)	1.7% (1/59)	25% (1/4)	10% (1/10)	0% (0/3)	0% (0/6)

LMIC-low and middle income country; HIC-high income country; GTD-gestational trophoblastic disease; FIGO- The International Federation of Gynecology and Obstetrics.

Supplementary Table S2: Linear correlation between cancer stage per tumour group and operative risk and performance status

Correlation of stage	Ovary	Uterus	Cervix	Vulva	Vagina	GTD
**with ECOG**	0.063	0.141	0.019	-0.064	-0.055	-0.167
**with ASA**	0.114	0.059	0.085	0.225	0.624	-0.258

ASA-American Society of Anesthesiologists physical status classification system; ECOG- Eastern Cooperative Oncology Group performance status scale; GTD-gestational trophoblastic disease.

Linear correlation coefficient >0.5 = strong correlation.

Linear correlation coefficient >0.7 = very strong correlation.

Supplementary Table S3: Adjusted 3-level models for predictors of intra-operative morbidity

	Univariable OR (95%CI)	Multivariable OR (95%CI)	Multivariable reduced OR (95%CI)	Multilevel OR (95%CI)
**Age**	0.971 (0.834-1.134), p=0.712	1.076 (0.885-1.312), p=0.465		
**Ethnicity**	0.776 (0.566-1.07), p=0.118			
**BMI**	0.799 (0.669-0.945), p=0.011	0.946 (0.769-1.152), p=0.591		
**ASA**	1.046 (0.822-1.33), p=0.714			
**ECOG**	1.143 (0.914-1.417), p=0.231	0.911 (0.685-1.198), p=0.51		
**Co-morbidities**	1.089 (0.784-1.502), p=0.606			
**Previous laparotomy**	1.303 (0.951-1.779), p=0.098			
**Previous laparoscopic surgery**	1.602 (1.13-2.247), p=0.007	1.571 (1.048-2.337), p=0.027	1.552 (1.045-2.286), p=0.028	1.6 (1.045-2.45), p=0.031
**MDM discussion**	0.745 (0.495-1.153), p=0.172			
**Pre-operative imaging**	1.238 (0.69-2.424), p=0.502			
**COVID 19**	1.88 (0.419-6.212), p=0.341	0.874 (0.14-4.287), p=0.875		
**FIGO stage**	0.298 (0.216-0.41), p<0.001	0.678 (0.451-1.02), p=0.062	0.671 (0.457-0.987), p=0.042	0.721 (0.477-1.09), p=0.12
**Pre-operative mechanical bowel prophylaxis**	2.217 (1.616-3.065), p<0.001	1.4 (0.967-2.034), p=0.076	1.442 (1.003-2.082), p=0.049	1.245 (0.776-1.997), p=0.363
**Pre-operative haemaglobin**	0.46 (0.216-0.796), p=0.023	0.544 (0.252-0.888), p=0.052	0.536 (0.244-0.885), p=0.053	0.633 (0.348-1.15), p=0.133
**GO surgeon vs non-GO surgeon**	1.265 (0.732-2.353), p=0.427			
**Trainee vs consultant**	1.013 (0.541-1.767), p=0.964			
**Elective vs emergency**	0.111 (0.033-0.352), p<0.001	0.121 (0.027-0.474), p=0.003	0.147 (0.04-0.505), p=0.002	0.179 (0.042-0.758), p=0.019
**WHO checklist (yes vs no)**	1.188 (0.686-2.213), p=0.561			
**Length of surgery**	1.778 (1.559-2.031), p<0.001	1.271 (1.072-1.5), p=0.005	1.271 (1.08-1.489), p=0.003	1.48 (1.201-1.824), p<0.001
**Estimated blood loss**	2.429 (2.061-2.888), p<0.001	1.987 (1.654-2.417), p=0	1.977 (1.653-2.393), p<0.001	2.226 (1.784-2.778), p<0.001
**Complete cytoreduction**	0.415 (0.252-0.707), p=0.001	0.802 (0.432-1.541), p=0.494		
**WBI**	0.898 (0.657-1.231), p=0.5	0.902 (0.597-1.366), p=0.625	0.97 (0.657-1.436), p=0.878	0.693 (0.36-1.333), p=0.272

**Surgical modality**	0.398 (0.275-0.564), p<0.001	0.695 (0.438-1.093), p=0.118	0.719 (0.463-1.105), p=0.136	0.552 (0.331-0.921), p=0.023
**Centre size**	1.071 (0.763-1.487), p=0.688			
**Recurrence vs primary surgery**	1.758 (1.121-2.687), p=0.011	1.621 (0.975-2.641), p=0.057	1.562 (0.945-2.523), p=0.074	1.59 (0.947-2.671), p=0.08
**Primary tumour**				
**Cervix**	0.84 (0.491-0.311), p=0.507	1.617 (0.881-2.891), p=0.111		
**Uterus**	0.445 (0.491-0.311), p<0.001	1.111 (0.699-1.762), p=0.656		
**GTD**	0.707 (0.491-0.311), p=0.749	0.321 (0.011-3.777), p=0.43		
**Vagina**	0.848 (0.491-0.311), p=0.833	1.073 (0.144-5.025), p=0.936		
**Vulva**	0.249 (0.491-0.311), p=0.003	0.533 (0.169-1.367), p=0.23		

Adjusted 3-level models (univariable, multivariable, multilevel) for predictors of intra-operative complications. N=1350 with 187 events.

LMIC-low and middle income country; HIC-high income country; BMI-body mass index; ASA-American Society of Anesthesiologists physical status classification system; ECOG- Eastern Cooperative Oncology Group performance status scale; GTD-gestational trophoblastic disease; COVID 19-Coronavirus disease; WHO-World Health Organization; MIS: Minimally invasive surgery (laparoscopy/robotic surgery); FIGO- The International Federation of Gynecology and Obstetrics; WBI-World Bank Institute.

Age: linear variable; ethnicity: Caucasian vs non-Caucasian; BMI: linear variable; ASA: linear variable; ECOG: linear variable; comorbidities: no comorbidity vs presence of 1 or more comorbidity; previous laparotomy: yes vs no; previous laparoscopic abdominal surgery: yes vs no; MDM discussion: yes vs no; pre-operative imaging: yes vs no; pre-operative COVID-19 status: positive vs negative/not tested; FIGO stage: I-II vs III-IV; pre-operative mechanical prophylaxis: yes vs no; intra-operative antibiotics: yes vs no; peri-operative management plan: yes vs no; pre-operative haemoglobin: linear variable; GO surgeon vs non-GO surgeon; trainee vs consultant: registrar/resident vs attending/consultant; elective vs emergency; WHO checklist: yes vs no; length of surgery: linear variable; estimated blood loss: linear variable; ITU recovery: yes vs no; HDU recovery: yes vs no; enhanced recovery: yes vs no; prophylactic post-operative antibiotics: yes vs no; surgical drain: yes vs no; indwelling urinary catheter: yes vs no; complete macroscopic cytoreduction: yes vs no; WBI: HIC vs LMIC; surgical modality: laparoscopic/robotic vs laparotomy/MIS converted to laparotomy; centre size: small/medium vs large; intra-operative complication: yes vs no; recurrence surgery vs primary surgery; cervix: cervix vs ovary; uterus: uterus vs ovary; GTD: GTD vs ovary; vagina: vagina vs ovary; vulva: vulva vs ovary.

Supplementary Table S4: Adherence to tumour specific audit standards by country income group

	LMIC	HIC	P value
**Ovary**			
Surgery performed by a gynaecologic oncologist or a trained surgeon (formal/informal training)	89.2% (256/287)	88.1% (399/453)	0.723
Treatment planned and reviewed at MDT	81.8% (238/291)	92.7% (418/451)	<0.001
**Uterus**			
Surgery performed by a gynaecologic oncologist or a trained surgeon (formal/informal training)	95.1% (291/306)	91.3% (407/446)	0.045
Treatment planned and reviewed at MDT	80.8% (249/308)	80.1% (354/442)	0.852
**Cervix**			
Surgery performed by a gynaecologic oncologist or a trained surgeon (formal/informal training)	95% (57/60)	85.2% (87/102)	0.071
Treatment planned and reviewed at MDT	84.1% (53/63)	86.1% (87/101)	0.821
**Vulva**			
Surgery performed by a gynaecologic oncologist or a trained surgeon (formal/informal training)	100% (63/63)	95.6% (65/68)	0.245
Treatment planned and reviewed at MDT	87.3% (55/63)	80.9% (55/68)	0.35
**Vagina**			
Surgery performed by a gynaecologic oncologist or a trained surgeon (formal/informal training)	100% (10/10)	75% (3/4)	0.286
Treatment planned and reviewed at MDT	100% (10/10)	100% (4/4)	
**Gestational trophoblastic malignancies**			
Surgery performed by a gynaecologic oncologist or a trained surgeon (formal/informal training)	100% (6/6)	100% (3/3)	
Treatment planned and reviewed at MDT	66.7% (4/6)	100% (3/3)	

Audit standards defined as per the European Society of Gynaecological Oncology (ESGO).

LMIC-low and middle income country; HIC-high income country; MDT-multi-disciplinary team/tumour board.

Supplementary Table S5: Centre demographics per income country setting and study participation status

	Centres participating in study(n=73)				Centres that did not participating in study (n=121)			
	HIC (n=30)	UMIC (n=17)	LMIC (n=19)	LIC (n=7)	HIC (n=25)	UMIC (n=35)	LMIC (n=39)	LIC (n=19)
**Institution type**								
Government health centre	4	4	5	1	2	4	4	0
Private health centre	0	2	1	0	1	1	2	3
Government district/rural hospital	2	0	0	0	0	20	21	12
Private district/rural hospital	0	0	0	0	1	0	0	0
Government academic/university hospital	18	6	4	5	17	9	8	3
Private academic/university hospital	5	2	5	1	1	0	3	0
Government provincial tertiary hospital	1	2	1	0	2	0	0	1
Private provincial tertiary hospital	0	1	1	0	1	1	0	0
NGO/Mission Hospital	0	0	2	0	0	0	1	0
**Mean number of new referrals per year (SD, range)**	mean=409.9 (SD=344.4, range 60-1230)	mean=338.1 (SD=290.8, range 20-1000)	mean=427.8 (SD=577.1, range 23-2000)	mean=598.9 (SD=790.9, range 36-2200)	mean=374.5 (SD =406.7, range 50-1500)	mean=390.8 (SD=488.3, range 5-1500)	mean=112.6 (SD=191.4, range 14-700)	mean=243.7 (SD=255.3, range 13-518)
**Mean number of GO surgeries performed per year (SD, range)**	mean=330.2 (SD=270, range 60-1100)	mean=209.8 (SD=235.6, range 25-1000)	mean=161.9 (SD=192.2, range 20-715)	mean=101.6 (SD=85.7, range 24-270)	mean=190.5 (SD =161, range 50-600)	mean=154.1 (SD=126.1, range 4-400)	mean=24.7 (SD=50.1, range 10-200)	mean=45.3 (SD=39.3, range 16-90)

**mean number of ovary cancer surgeries performed per year (SD, range)**	mean=98.5 (SD=106.3, range 10-520)	mean=41.5 (SD=41.2, range 2-150)	mean=66.7 (SD=82.4, range 0-315)	mean=19.1 (SD=17.5, range 0-50)	mean=68.6 (SD= 101.1, range 20-350)	mean=35.6 (SD=19.1, range 1-70)	mean=17.9 (SD=4, range 6-18)	mean=10.7 (SD=4, range 7-15)
**mean number of uterine cancer surgeries performed per year (SD, range)**	mean=118.3 (SD=103.5, range 15-420)	mean=89.6 (SD=117.2, range 10-500)	mean=54.3 (SD=77, range 10-320)	mean=42.6 (SD=45.9, range 10-140)	mean=62.5 (SD= 68.1, range 20-250)	mean=49.3 (SD=33.9, range 2-100)	mean=13.4 (SD=39.2, range 5-150)	mean=23 (SD=15.7, range 9-40)
**mean number of cervical cancer surgeries performed per year (SD, range)**	mean=35.4 (SD=33.2, range 5-140)	mean=32.2 (SD=29.9, range 3-100)	mean=32 (SD=67.5, range 0-286)	mean=26.3 (SD=36.9, range 1-105)	mean=25.2 (SD= 19.3, range 5-67)	mean=25.9 (SD=15.5, range 1-50)	mean=8.2 (SD=23, range 1-90)	mean=11 (SD=7.9, range 5-20)
**mean number of vulva cancer surgeries performed per year (SD, range)**	mean=26.4 (SD=28.6, range 5-120)	mean=10.2 (SD=15, range 2-65)	mean=9.7 (SD=14.5, range 0-50)	mean=10.7 (SD=14.7, range 0-33)	mean=15.9 (SD= 14.6, range 3-50)	mean=8.7 (SD=5.7, range 0-20)	mean=6.1 (SD=19.8, range 0-75)	mean=2.3 (SD=2.5, range 0-5)
**mean number of vagina cancer surgeries performed per year (SD, range)**	mean=4.7 (SD=7.1, range 0-35)	mean=3.2 (SD=2.9, range 0-10)	mean=6.1 (SD=13.6, range 0-55)	mean=3.9 (SD=4.1, range 0-12)	mean=9.3 (SD=17.6, range 0-56)	mean=5.1 (SD=3.2, range 0-10)	mean=5.4 (SD=18.4, range 0-67)	mean=2.3 (SD=2.5, range 0-5)
**mean number of GTD surgeries performed per year (SD, range)**	mean=1.9 (SD=2.4, range 0-10)	mean=6.3 (SD=7.4, range 0-25)	mean=15.4 (SD=24.9, range 0-102)	mean=10.9 (SD=14, range 0-40)	mean=13.7 (SD= 29.3, range 0-100)	mean=11.5 (SD=27.9, range 0-100)	mean=4.9 (SD=14.8, range 1-58)	mean=0.3 (SD=0.6, range 0-1)
**Level 2/3 critical care unit facilities available on site**	30	17	19	7	25	12	13	3
**Centres participating in gynaecological oncology research studies**	30	13	12	5	25	10	8	2

HIC-high income country; UMIC-upper middle income country; LMIC-low middle income country; LIC-low income country; GO-gynaecological oncology.

Supplementary Table S6: Themes associated with research, training, surgical morbidity and mortality

Theme	Quote
**Individual**	
**Altruism**	*"This mission must not end, when good results come out we must continue to get more. We are a war-torn country but we cannot say we don't have time, we cannot say we don't have money, electricity. I cannot say I am not available; this is not for me. We are committed to doing the best we can – we owe it to our patients. The world has forgotten us. We only have each other."* – LMIC centre
**Burnout**	*"With ever increasing clinical work and the ever increasing research studies we are invited to participate in, it can be hard to have the enthusiasm to participate new research. We are all so overwhelmed with work and burnt out."* - HIC
**Culture**	"The lack of heath awareness amongst our patients is difficult. Often they do not prioritise their health – this is a common problem in our culture where people do not like to talk out their health. We as clinicians are then faced with treating advance stage disease often presenting for the first time in the emergency room." – LMIC centre
**Organisational**	
**Resource limitations**	*"Many of our critical care specialists and surgeons have left the country. This is a financially poor nation and our limited resources negatively impact on surgical morbidity and mortality. For example, drugs are not always available and not always affordable to obtain privately if not available at a government hospital, late diagnosis due to lack of screening and symptom awareness, patients understanding of drugs they may not take the correct dose, they may take lesser dose to make the drug last longer which has consequences and drugs not always available."* – LMIC centre<br><br>*"Shortage of beds, lack of theatre time, long waiting list impact on morbidity and mortality as treatment delays due to limited resources result in a greater proportion of our patients presenting with advanced stage disease."* – HIC centre
**Logistics**	*"The ethical approval board is a complicated process, time consuming, lengthy and frustrating."* – HIC centre
**Education**	*"Gynaecological Oncology is not a recognised speciality in our country and in many countries that neighbour us. Surgeries are performed by surgical oncologists or general surgeons who have informal training in gynaecological oncology. We do not have any specific training programmes."* – LMIC centre<br><br>*"Yes I am a certified Gynaecological Oncologist who underwent formal training as part of a national training programme as did all of my colleagues."* – HIC centre
**National**	
**War**	*"We are a country at war. It has been like this for many years. Our patients and our staff are all living under extreme conditions. Daily bombings and war casualties are a daily occurrence for us. This constant decay in infrastructure has a very bad effect on our patients*

	health as well as ours. Patients and staff alike risk their lives coming to hospital. We are all so scared that this might be our last day and we may never see our families again.” – LMIC centre
**Pandemic**	*“COVID 19 has been tough on us all. We are working through waiting list backlogs caused by the pandemic. COVID 19 resulted in a pause in national screening programmes and delayed diagnosis resulting in a stage shift and patients presenting with more advanced stage disease. More complex surgery then increases the risk of a post-operative complication.”* – HIC centre
**Policy**	“Unfortunately women health (including gynaecological cancers) is not a national priority in our country. This results in ongoing gender inequalities.” – LMIC centre

LMIC-low and middle income country; HIC-high income country.

Supplementary Table S7: Facilitators and barriers to research participation

**Facilitators**	**Quote**
Altruism	*"I like taking part in research because I really want to make a difference to the patients under my care and to humanity as a whole."* – LMIC centre
Authorship	*"Authorship is also important as it gives you recognition for work often performed out with contracted hours."* – HIC centre
Scientific value	*"It is always exciting to take part in a study that is well designed to answer an important research question."* – HIC centre
Time and oragnisational support	"Having the time and staff to take part in studies helps make participation easier. Also, the support of your institute to take part helps." – HIC centre
**Barriers**	**Quote**
Exclusion	*"Quite often LMIC centres are excluded from participating in international studies. We are simply not invited to take part. For our patients to benefit, they must be included so that change can come about."* – LMIC centre
Complex regulatory approvals	*"Getting approval is a long, complicated and bureaucratic process that is very slow. The system is unnecessarily complicated."* – HIC centre
Clinical commitments	*"Clinical work often needs to take priority. It is difficult to find time when we have so much clinical work."* – HIC centre
Lack of resources	*"We often have power cuts due to the ongoing war in our country and often don't have the appropriate infrastructure to take part."* – LMIC centre

LMIC-low and middle income country; HIC-high income country.
